# Integrating Bulk RNA and Single‐Cell RNA Sequencing Identifies and Validates Lactylation‐Related Signatures for Intervertebral Disc Degeneration

**DOI:** 10.1111/jcmm.70262

**Published:** 2024-12-05

**Authors:** Yangyang Shi, Fudong Li, Wenbo Lin, Linhui Han, Jinyu Wang, Chen Yan, Jingchuan Sun, Chenglong Ji, Jiangang Shi, Kaiqiang Sun

**Affiliations:** ^1^ Department of Orthopedic Surgery, Changzheng Hospital Navy Medical University Shanghai China; ^2^ Department of Orthopedics Naval Medical Center of PLA Shanghai China

**Keywords:** intervertebral disc degeneration, lactylation, molecular mechanism

## Abstract

Glycolysis‐related lactylation has gained wide attention for regulating various cellular functions and diseases. Nevertheless, its intricate involvement in intervertebral disc degeneration (IVDD) is not yet fully understood. In this study, we unrevealed the intricate association between elevated lactylation levels and the development of IVDD. Subsequently, we harvested the lactylation‐related genes (LRGs) and systematically analysed the expression levels of these genes to establish a gene signature related to IVDD through multiple bulk RNA sequencing data. Six hub LRGs were determined and closely associated with the increased severity of IVDD. Among the six genes, CBX3 was the most upregulated in both in vivo and in vitro experiments. Furthermore, molecular docking identified atosiban acetate as a specific inhibitor for CBX3, and inhibiting the expression of CBX3 using atosiban acetate significantly repressed the glycolysis activity and global lactylation level, thus alleviating the progression of IVDD. In conclusion, the lactylation correlates positively with IVDD and the LRG signature could be used as a biomarker for the effective clinical treatment of IVDD. CBX3 emerged as one of the key LRGs in IVDD, and atosiban acetate, as a specific inhibitor for CBX3, may be a promising therapeutic candidate for IVDD by affecting lactylation.

## 1 Introduction

Low back pain (LBP), the predominant symptom of spinal degeneration, is a leading cause of disability worldwide and imposes a huge economic burden on society. Over 80% of individuals are likely to experience LBP at some point during their lifetime [1, 2]. Intervertebral disc degeneration (IVDD) has been identified as the primary pathological cause of LBP [3]. Despite significant advances in understanding the pathophysiology of IVDD, the molecular mechanisms underlying this complex disease remain incompletely understood.

The intervertebral disc (IVD) is an avascular structure composed of the inner nucleus pulposus (NP), outer annulus fibrosus and upper and lower cartilage end plates. Given low oxygen tension in IVDs, anaerobic glycolysis serves as the primary energy source for NP cells (NPCs) [4, 5]. The maintenance of glycolysis is vital for NPC survival in IVDs. Disruption in the balance between glucose utilisation and lactate production can significantly impact NP cell function. Previous research has shown that in degenerative NPCs, the expression of key glycolytic enzymes is upregulated, while inhibiting glycolysis has been found to promote autophagy, enhance extracellular matrix synthesis and suppress cellular senescence [6]. Moreover, NPCs display metabolic flexibility. When lactate accumulates significantly due to monocarboxylate transporter 4 (MCT4) inhibition, the level of glucose‐6‐phosphate, a glycolytic intermediate, is found to increase twofold, indicating that lactate further stimulates glycolysis [7]. Unfortunately, the precise cellular mechanisms by which lactate affects NPCs during IVDD are still elusive.

Lactylation is a recently discovered posttranslational modification (PTM) of proteins, which involves the covalent attachment of lactate moieties to target proteins mediated by lactyltransferases and delactylases [8]. Lactylation has gained wide attention for its role in regulating various cellular functions and diseases, including inflammatory response, tumour invasion and tissue regeneration [8, 9, 10]. Lactylation occurs in both histone and nonhistone proteins. Histone lactylation, for instance, at H3K18la sites, has been demonstrated to directly stimulate downstream gene transcription, and lactylated nonhistone proteins would undergo alternations of function [8, 11, 12]. Previous studies have reported that IVDD was accompanied by excessively increased glycolysis and lactate accumulation within IVD, which may disturb the repair and regeneration of NPCs [13, 14, 15]. Therefore, it is inferred that lactylation and its related genes or pathways may also participate in the progression of IVDD and that lactylation‐related genes and biomarkers could pave the way for novel therapeutic strategies. However, research on the relationship between lactylation and IVDD remains scarce.

**FIGURE 1:**
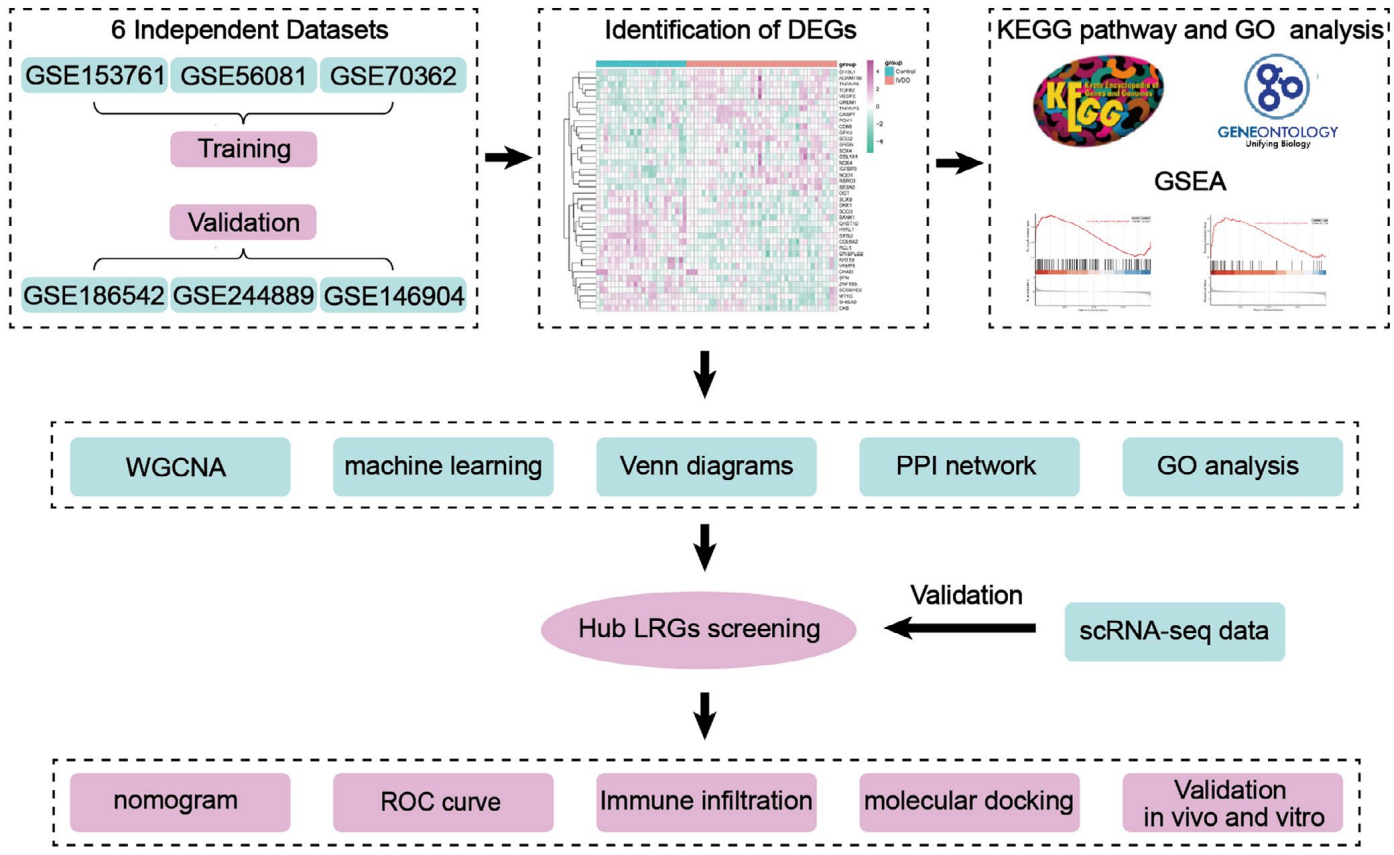
The overall flow of this study.

Consequently, in this study, we first discovered the intricate relationship between elevated lactylation levels and the development and progression of IVDD. Further, we identified the lactylation‐related genes (LRGs) and systematically analysed their expression levels to establish a gene signature related to IVDD through multiple bulk RNA sequencing data. Subsequently, the glycolysis alternations and the expression of the hub genes were validated based on single‐cell RNA sequencing (scRNA‐seq) data. Additionally, molecular docking targeting the critical hub gene was performed to explore the therapeutical effects of regulating glycolysis and lactylation levels on IVDD. The flow chart is shown in Figure 1.

## 2 Materials and Methods

### 2.1 Data Acquisition and Processing

In the study, both the bulk RNA sequencing data and scRNA‐seq data were obtained from the Gene Expression Omnibus (GEO) database (http://www.ncbi.nlm.nih.gov/geo). Specifically, the gene expression profiles of bulk RNA sequencing data (GSE153761 [16], GSE56081 [17], GSE70362 [18], GSE244889 [19], GSE186542 [20] and GSE146904 [21]) were downloaded. Data from GSE153761, GSE56081 and GSE70362 were used to establish the training set, and the remaining data were treated as validation sets. Totally, 332 LRGs were selected according to prior research (Table S1) [22]. The underlying R code for bioinformatic analysis is shown in Table S2.

### 2.2 Differential Expression Gene Analysis and Functional Analysis

The ‘limma’ R package [23] was utilised to detect differentially expressed genes (DEGs) in GE153761, GSE56081 and GSE70362 using *p* < 0.05 as the threshold value. The ‘pheatmap’ package drew the volcano plot and the heat map. The R packages ‘org.Hs.eg.db’ and ‘clusterProfiler’ were employed to elucidate the biological mechanisms of DEGs [24]. Additionally, gene ontology (GO) and Kyoto Encyclopedia of Genes and Genomes (KEGG) enrichment analyses were conducted to identify significant enrichments with a threshold of *p* < 0.05. The GO analysis encompassed biological process (BP), molecular function (MF) and cellular component (CC).

### 2.3 Weighted Gene Coexpression Network Analysis

Weighted gene coexpression network analysis (WGCNA) was utilised by the WGCNA R package to construct gene coexpression networks linked to clinical characteristics [25]. Hierarchical clustering analysis was performed to eliminate outliers. Following this, an optimal soft threshold was chosen to build the weighted adjacency matrix, which was then converted to a topological overlap matrix (TOM). Finally, Pearson correlation coefficients were calculated to assess the relationships between modules and traits.

### 2.4 Construction and Validation of Nomogram Models

Eigengenes were integrated to construct nomograms by the R package ‘rms’. The accuracy of the nomogram was evaluated by calibration curves, and its clinical applicability was assessed through decision curve analysis. In order to evaluate the efficacy of the identified LRGs, the receiver operating characteristic (ROC) curves for each gene were separately plotted using the ‘pROC’ R package [26].

### 2.5 Identification of Hub LRGs via Machine Learning Algorithms

Two machine learning algorithms, LASSO and RF, were used to systematically detect hub LRGs associated with IVDD. LASSO analysis was conducted using the ‘glmnet’ R package [27]. The RF algorithm was executed using the ‘randomForest’ package. Subsequently, Venn diagram was employed to depict the intersection of the hub LRGs identified by both algorithms. To explore the interplay among LRGs, GeneMANIA [28] was utilised to analyse protein coexpression patterns and construct the PPI networks for hub genes identified in our study.

### 2.6 Immune Infiltration Analysis

The CIBERSORT analysis was utilised to quantify the proportion of 22 distinct immune cell types in both IVDD patients and healthy controls [29]. The findings were plotted using the ‘ggboxplot’ R package. Furthermore, R package ‘ggplot2’ was utilised to analyse the correlation between the immune cells and the hub genes.

### 2.7 Single‐Cell RNA‐Seq Data Analysis

The Seurat package was employed to analyse scRNA‐seq data [30]. Briefly, we filtered out low‐quality cells by removing barcodes exhibiting outlier gene counts, which may indicate dying cells, cell doublets and cells with compromised membranes. After removal of low‐quality data, the remaining high‐quality cells were normalised and scaled to achieve linear conversion by using the ‘ScaleData’ and ‘NormalizeData’ functions. The clusters were then plotted by using Uniform Manifold Approximation and Projection (UMAP). Cells were grouped according to the classical marker expression profiles.

### 2.8 Pseudotime Trajectory Construction

Monocle 2 or cell differentiation trajectory analysis was used. This analysis commenced with data normalisation to mitigate technical biases, followed by differential expression testing to identify genes marking cellular transitions. Utilising Monocle 2's unsupervised learning, we constructed cellular trajectories, revealing the dynamism of differentiation within NPC subsets. The pseudotime‐dependent gene expression across all NPCs was further identified using an unsupervised approach.

### 2.9 Score According to Metabolism‐Related Hallmark Genes

Module scores and the enrichment fraction for glycolysis‐related gene expression in single cells were determined using AUCell. The glycolysis‐related hallmark gene was obtained from https://www.gsea‐msigdb.org/. We assessed the glycolysis score and AUC value in each NPC type with the glycolysis‐related hallmark genes.

### 2.10 Small‐Molecule Therapeutics Screened and Docking

Autodock for molecular docking was performed to investigate interactions between small molecule compounds and identified genes [31]. Initially, we compiled a list of small molecule compounds that interact with selected genes using the CTD database (http://ctdbase.org/). Subsequently, the primary protein structures of the target genes were downloaded from the PDB database (https://rcsb.org/). Following this, we obtained the biological macromolecular structures corresponding to the selected features from the Uniprot database (https://uniprot.org/). Finally, the automatic docking of small molecular compounds and biological macromolecules was conducted. The Pymol software was used to visualise the results.

### 2.11 The Acquisition of Human NP Tissue

This study received ethical approval from Shanghai Changzheng Hospital (No. 2022SL018‐2), and all participants provided written informed consent. The NP tissues were classified into two groups based on the Pfirrmann grades: moderate degeneration (Grade II or III) and severe degeneration (Grade IV or V). All procedures involving human samples adhered to the principles outlined in the Helsinki Declaration.

### 2.12 Cell Culture

The NPCs were derived from Pfirrmann Grade II patients. Briefly, NP tissues obtained during surgery were transported to an ultra‐clean laboratory in 0.9% sodium chloride solution. After being cut into pieces and washed three times with sterilised PBS (G0002; Servicebio, China), the NP tissues were digested with 0.25% Trypsin–EDTA (G4001; Servicebio, China) for 30 min, followed by incubation with an equal amount of complete DMED/F‐12 medium (containing 1% penicillin–streptomycin and 10% foetal bovine serum) and collagenase type II (0.2%; Invitrogen, USA) for 1 h. After centrifugation at 1200 rpm for 3 min, the NPCs were resuspended in fresh complete DMEM/F‐12 medium. Subsequently, the NPCs were counted and replanted in a T25 culture flask under sterile conditions with 5% CO_2_ at 37°C. The medium was refreshed every 3–4 days. NPCs were grown to 80%–90% confluence for subsequent experiments.

### 2.13 Construction of a Mouse Model of IVDD

All animal procedures were conducted in accordance with the Animal Research: Reporting of in vivo Experiments (ARRIVE). In the present study, 8‐week‐old C57BL/6 mice were used to establish AF needle puncture–induced IVDD model, following a previously described protocol [32]. Briefly, after preparing the skin, the mice were anaesthetised with isoflurane. A 22 G needle was vertically inserted into the coccygeal 5/6 (Co5/6) disc, and rotated for 8 s. For IVDD treatment, atosiban acetate was injected intraperitoneally and the dose was calculated in reference to previous studies using the metrological conversion formula between rat and mouse [33]. The dose in mice is = 0.1 mg/kg × 200 g × 0.14/20 g = 0.14 mg/kg. Four weeks after the operation, the disc tissues were harvested for subsequent experiments.

### 2.14 Quantitative Reverse Transcription Polymerase Chain Reaction Analysis

The quantitative reverse transcription PCR (qRT‐PCR) procedure adhered to previously established methods [34]. Initially, total RNAs were extracted using Rapture Universal RNA Plus Kit (R4013‐02; Magen, China) as per the manufacturer's instructions. After purification, total RNAs were reverse‐transcribed into cDNAs, followed by amplified using ChamQ Universal SYBR qPCR Master Mix (Q711‐03; Vazyme, China). GAPDH was used as the internal control, and the results were calculated using the ΔΔCt method. The following primers were used: IGFBP3, (F:5′‐CAGCCTTCTGTGGTGTCATT‐3′, R:5′‐TAGTCCCCAAGCAGTACAGGT‐3′); THUMPD1, (F:5′‐AGCTCTCCTGTCCCATTGTG‐3′, R:5′‐TCAAGTGGCTGTGATTTATTGTTAC‐3′); CBX3, (F:5′‐GATGATACCAGTAAGGCATTACAGT‐3′, R:5′‐TCCTGTGGAAATACATATTCAAGTT‐3′), RBM10, (F:5′‐ACAATGGTGACCCGCTTCAA‐3′, R:5′‐GTCCCAGCCATCCAACACTCT‐3′); DDIT4, (F:5′‐CCTCCAAGACAGAGACGACTG‐3′, R:5′‐CTCAGTTTTCCAACCACAGGA‐3′); CHST1, (F:5′‐GTGGCTCAAGGGCAAGTACAT‐3′, R:5′‐CCTCGGTCTTCTTCATAGGGTTC‐3′); ACAN, (F:5′‐TGGAGACAAGGATGAGTT TCC‐3′, R:5′‐GGCGAAGCAGTACACATCATA‐3′); COL2A1, (F:5′‐CCAGAAACAACACAATCCGTT‐3′, R:5′‐ATGGACATCAGGTCAGGTCAG‐3′); ADAMTS5, (F:5′‐CTGTGACCAAAAGAGGATGTG‐3′, R:5′‐AGTGTTTCTTGTAAGCCCAGG‐3′); MMP3, (F:5′‐GCAGTCTTTCTTCGGCTTAGA‐3′, R:5′‐TTGTATTCACCCACATCAGGA‐3′), PKM2, (F:5′‐ATGTCGAAGCCCCATAGTGAA‐3′, R:5′‐TGGGTGGTGAATCAATGTCCA‐3′); LDHA, (F:5′‐ATGGCAACTCTAAAGGATCAGC‐3′, R:5′‐CCAACCCCAACAACTGTAATCT‐3′).

### 2.15 Immunofluorescence (IF) Analysis

The NPCs were subjected to immunofluorescent staining for Pan Kla (PTM‐1401; PTM BIO, China, 1:100). Initially, the samples were fixed with 4% paraformaldehyde for 15 min and then permeabilised with Triton X‐100 (0.1% vol/vol) for an additional 10 min. Next, the samples were blocked with 5% BSA at room temperature for 1 h and subsequently incubated with respective primary antibodies overnight at 4°C. After washing three times, the samples were treated with secondary antibody (550076; Zen Bio, China, 1:500) for 1 h.

### 2.16 Immunohistochemical (IHC) Assay

The mice NP sections were deparaffinised (G1128; Servicebio, China) and dehydrated using a gradient of alcohol. After incubation with 3% BSA for 25 min, the sections were then treated with primary antibody against CBX3 (11650‐2‐AP; Proteintech, Chian, 1:100) at 4°C for 12 h. The next day, the sections were exposed to secondary antibody (511203; ZENBIO, China, 1:300) for 60 min. Stained section images were observed using light microscopy (BX43; Olympus, Japan).

### 2.17 Haematoxylin and Eosin (HE), and Safranin O and Fast Green (SF) Staining

The mice NP sections underwent deparaffinisation, followed by dehydration in a gradient of alcohol. Subsequently, the sections were stained with HE or SF, following standard protocols. Images of stained section were acquired using light microscopy (Olympus, Japan).

### 2.18 Western Blot Analysis

NPCs were lysed using RIPA buffer containing phosphatase and protease inhibitors and then centrifuged at 12,000 *g* for 15 min at 4°C. The protein concentrations were quantified by BCA Protein Assay Kit (P0010s; Beyotime, China). Loading buffer (LT101S; Epizyme Biomedical Technology Co. Ltd., China) was added to the samples, and the samples were heated at 95°C for 10 min. Equivalent amounts of protein were separated using 10% SDS‐PAGE, followed by transfer onto 0.45‐μm pore size PVDF membranes (IPVH00010; Millipore, USA). The membranes were blocked in 5% skimmed milk dissolved in TBST for 2 h and then incubated with the following primary antibodies at 4°C overnight: ACAN (DF7561; Affinity, China, 1:1000), COL2A1 (AF0135; Affinity, China, 1:1000), ADAMTS5 (DF13268; Affinity, China, 1:1000), MMP3 (AF0217; Affinity, China, 1:1000), LDHA (DF6280; Affinity, China, 1:1000), PKM2 (AF5234; Affinity, China, 1:1000), Pan Kla (PTM‐1401; PTM BIO, China, 1:500), β‐actin (AF7018; Affinity, China, 1:3000). After washed with TBST, the membranes were incubated with the secondary antibody (511203; ZENBIO, China, 1:3000) for 2 h. The protein bands were imaged using Omni‐ECL Femto Light Chemiluminescence Kit (SQ201L; Epizyme Biomedical Technology Co. Ltd., China) and an automatic chemiluminescence/fluorescence image analysis system (5200; Tanon, China).

### 2.19 Cell Counting Kit‐8 (CCK‐8) Assay

NPCs were seeded into a 96‐well plate at a density of 4 × 10^3^ cells. Then, a range of atosiban acetate (1, 5, 10, 20, 50 μM) was added to the plates for 24 h. CCK‐8 detection solution (prepared by mixing 10 μL CCK‐8 reagent with 90 μL DMEM/F‐12 medium) was added to each well and incubated at 37°C for 2 h. The optical density (OD) was measured at a wavelength of 450 nm using a microplate reader.

### 2.20 Statistical Analysis

All the quantitative data were shown as means ± SD. For normally distributed variables between two groups, unpaired Student's *t*‐tests were utilised. When variables were not normally distributed, Mann–Whitney *U* tests were applied. For comparisons involving more than two groups, one‐way ANOVA tests were used. Statistical analysis was performed using Graphpad Prism (Version 9.0), with statistical significance defined at *p* < 0.05.

## 3 Results

### 3.1 Global Lactylation Level Was Upregulated in Human Degenerative NP Tissue

**FIGURE 2:**
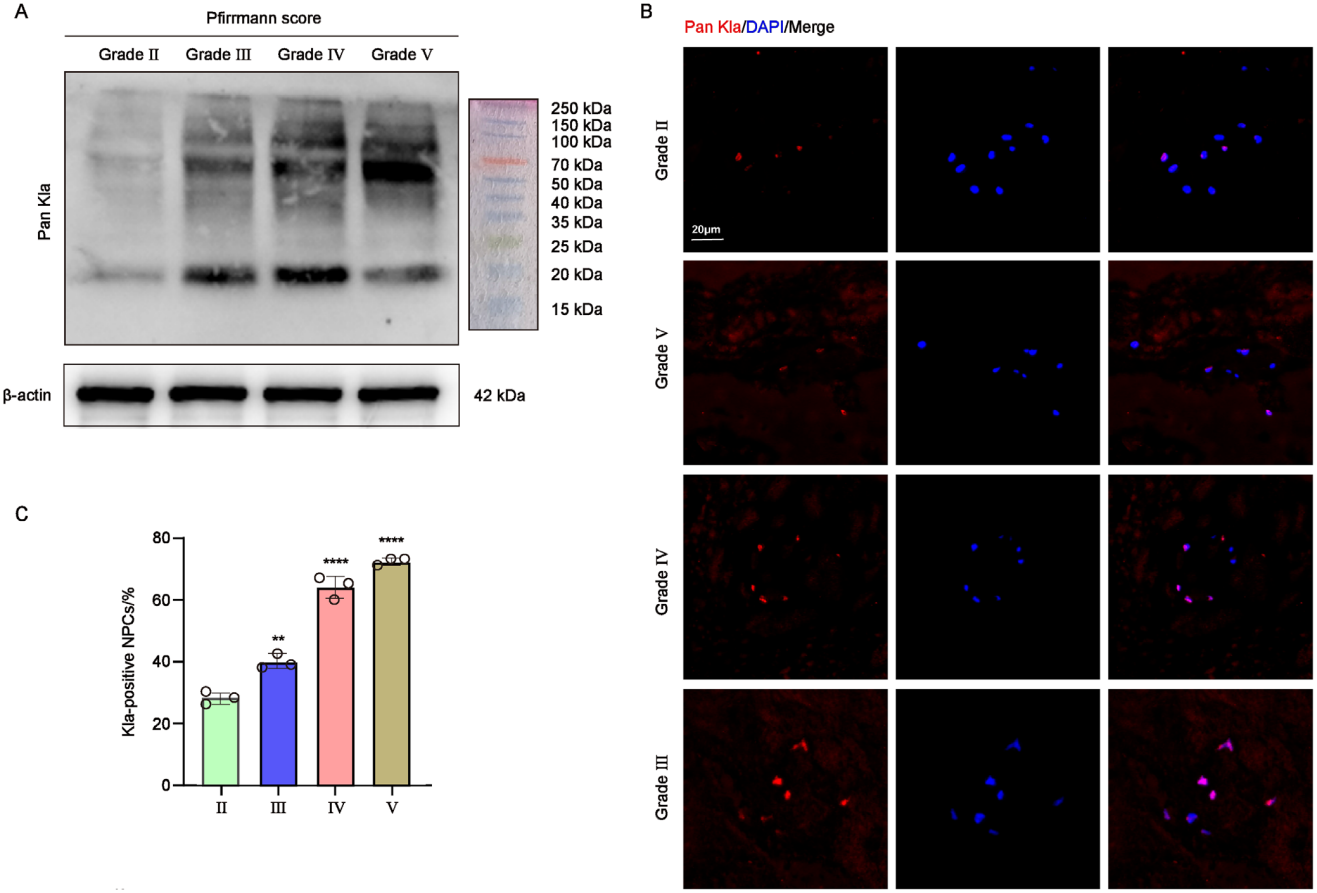
Elevated lactylation level in degenerated human nucleus pulposus tissue. (A) Western blot analysis showing the protein expression of Pan Kla in human degeneration NP tissues. (B) IF staining of Pan Kla expression in human degeneration NP tissues. Scale bar = 20 μm. (C) Statistical analysis showing the percentage of Kla‐positive NPCs. **p < 0.01, ****p < 0.0001 (Pan Kla was the symbol of lactylation).

Given the fact that glycolysis intensifies with the progression of IVDD and that glycolysis‐derived lactate is the crucial substrate for lactylation, we initially investigated the change of global lactylation in human NP tissue across varying degeneration degrees as classified by the Pfirrmann grades. The result of WB demonstrated that the lactylation level was upregulated with the progression of IVDD (Figure 2A). In addition, the change of lactylation in NP tissue was further examined using IF staining. As shown in Figure 2B,C, the IF and quantified results indicated an increase in lactylation‐positive NPCs, positively correlated to the degree of IVDD. Therefore, these findings indicated a direct relationship correlation between the lactylation level and the severity degree of IVDD.

### 3.2 Identification of DEGs and Enrichment Analysis

**FIGURE 3:**
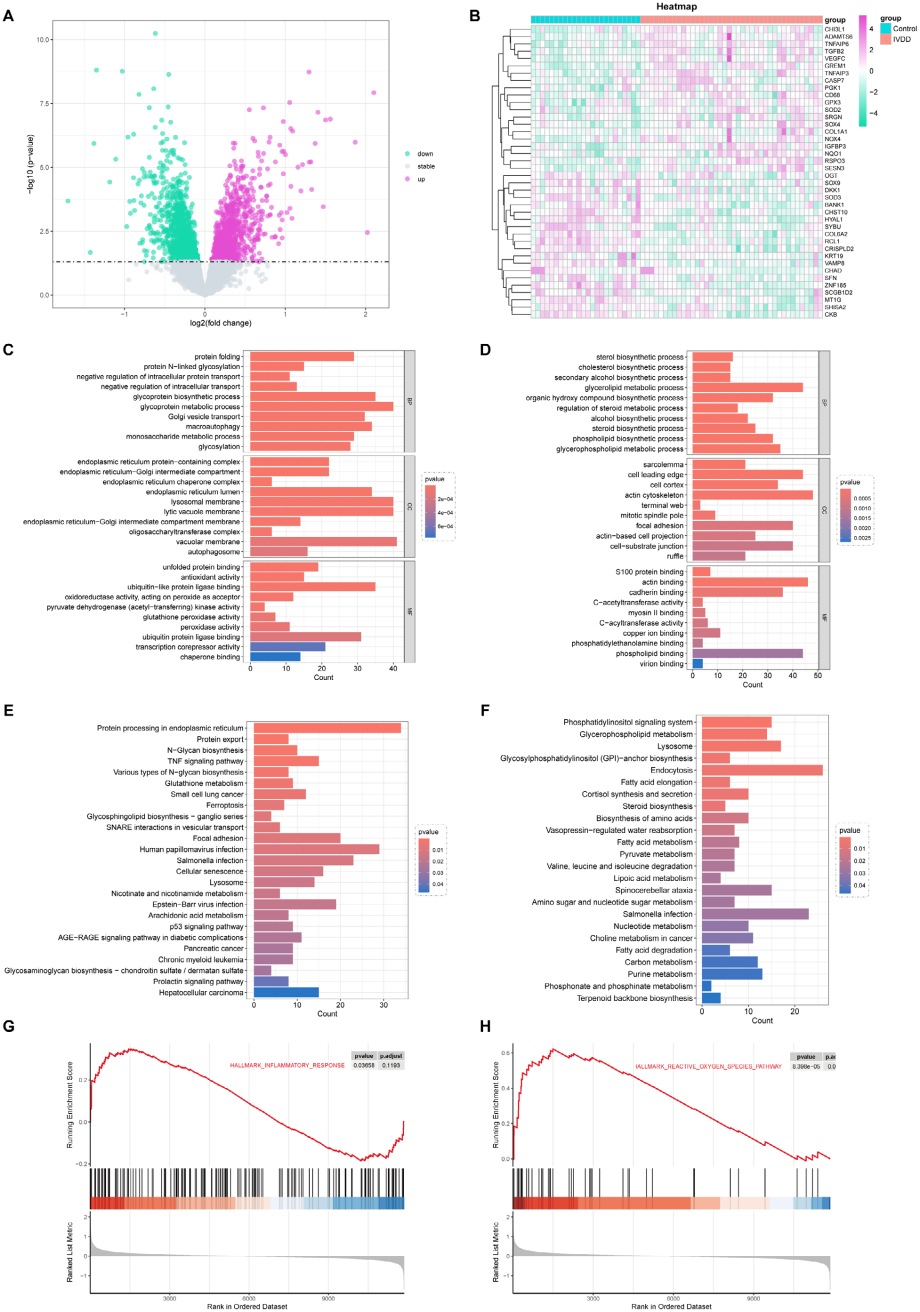
Different expression analysis and function enrichment analysis in normal and degenerated human IVD tissue. (A) Volcano plot of DEGs between IVDD tissues and normal tissues. (B) Clustering heatmap of the expression of DEGs in IVDD samples and normal samples. (C) GO enrichment analysis of upregulated DEGs. (D) GO enrichment analysis of downregulated DEGs. (E) KEGG pathway analysis based on upregulated DEGs. (F) KEGG pathway analysis based on downregulated DEGs. (G, H) The biological functions were associated with the development of IVDD.

We employed the ‘limma’ package to analyse the DEGs in the healthy controls and IVDD samples, identifying 2179 DEGs (1006 upregulated and 1173 downregulated) with *p* < 0.05 as threshold, as depicted in the volcano map and heat map (Figure 3A,B). Furthermore, GO and KEGG enrichment analyses on both upregulated and downregulated genes were conducted to investigate the molecular mechanisms and functions in IVDD. The main changes of upregulated genes in BP were glycoprotein metabolic process, macroautophagy and monosaccharide metabolic process. The primary changes in CC included endoplasmic reticulum protein, Golgi intermediate compartment and lytic vacuole membrane. The significant variations in MF involved unfolded protein binding, antioxidant activity and ubiquitin‐like protein ligase binding (Figure 3C). As for downregulated genes, GO analysis in the BP category showed enrichment in cholesterol biosynthetic process, sterol biosynthetic process and glycerolipid metabolic process. In the CC category, DEGs were involved in sarcolemma, cell leading edge and cell cortex. In the MF category, DEGs were related to actin binding and cadherin binding (Figure 3D). KEGG analysis of upregulated genes was mainly associated with TNF signalling pathway, glutathione metabolism and ferroptosis (Figure 3E). The KEGG pathways of downregulated genes were predominantly involved in phosphatidylinositol signalling system, glycerophospholipid metabolism and endocytosis (Figure 3F). The detailed list of GO and KEGG analyses is shown in Table S3. Furthermore, gene set enrichment analysis (GSEA) was conducted to assess the activated status of pathways related to inflammatory and reactive oxygen species (ROS) in the degenerative NP. The results demonstrated the activation of inflammation and ROS during the development of IVDD (Figure 3G,H).

### 3.3 Construction of Weighted Coexpression Network and Identification of Critical Modules

**FIGURE 4:**
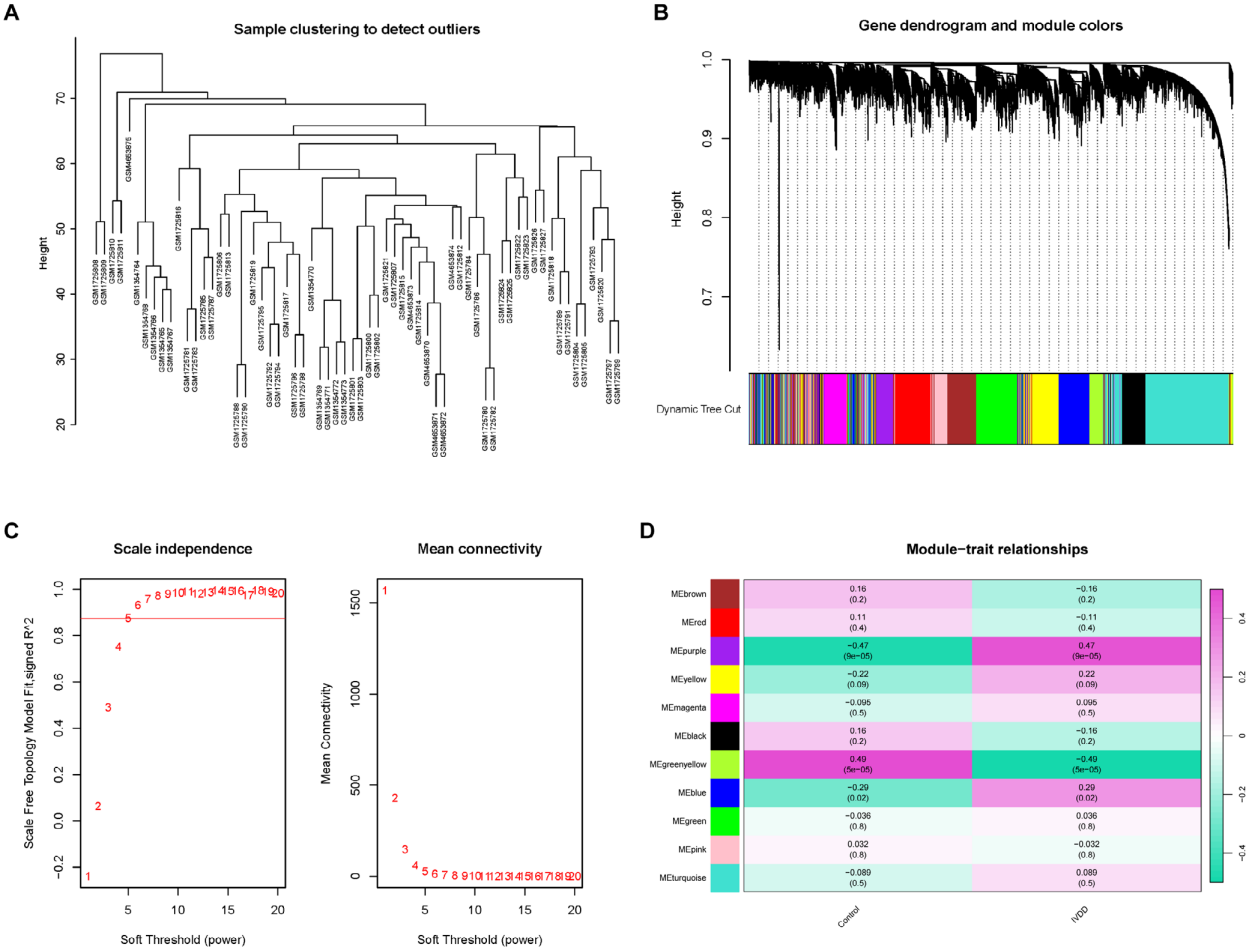
Construction of weighted coexpression network and identification of critical modules. (A) Cluster dendrogram of IVDD. (B) Cluster tree dendrogram of coexpression modules. Different colours represent distinct coexpression modules. (C) The selection of soft threshold power. (D) Heatmap of the correlation between gene modules and clinical traits of IVDD.

Utilising the WGCNA package alongside clinical information, we constructed a network that incorporated the top 50% of genes based on variance (Figure 4A). The samples exhibited robust clustering with no evident outliers. Average linkage hierarchical clustering was utilised to categorise gene modules, each containing at least 200 genes (Figure 4B). The optimal soft threshold for the model was identified to be 5, with the scale‐free R2 set to 0.85 (Figure 4C). Subsequent merging of similar gene modules resulted in the identification of 11 coexpression gene modules (Figure 4D). The results indicated that the green–yellow module was significantly negatively correlated with IVDD (Cor = 0.48, *p* = 1.6e‐20), suggesting a potential protective role against IVDD. Conversely, the purple module demonstrated a strong positive correlation with IVDD (Cor = 0.23, *p* = 2.9e‐06, Figure 4D), indicating its potential contribution to the development of IVDD. Consequently, hub genes within both modules were subjected to further analysis.

### 3.4 Identification of Lactylation‐Related Genes

**FIGURE 5:**
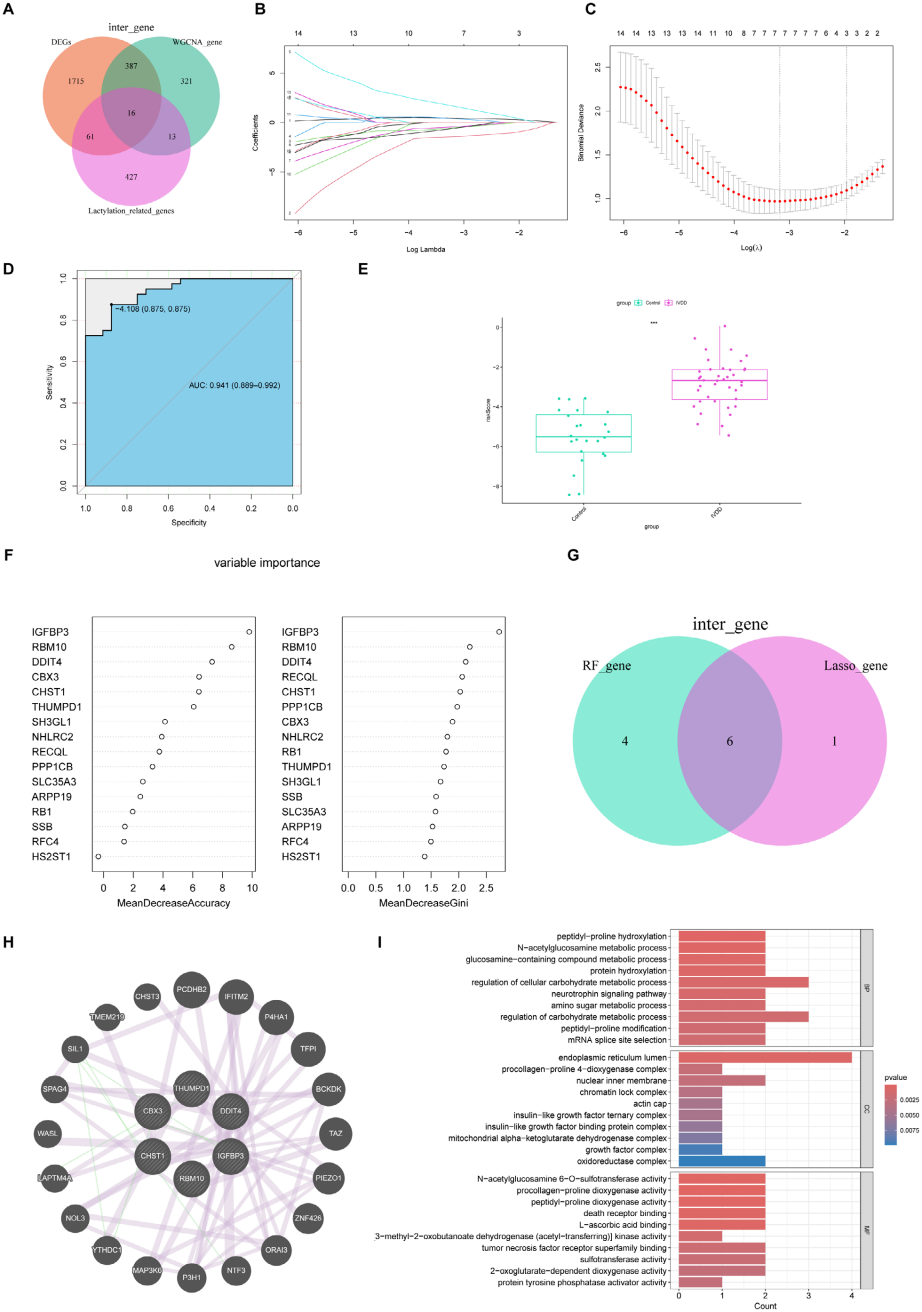
Hub LRGs screening based on machine learning algorithms. (A) The Venn diagram illustrated the common genes by DEGs, WGCNA and LRGs. (B) Plot of LASSO coefficient profiles. (C) Plot of LASSO partial likelihood deviance. (D) ROC curve of the LASSO model. (E) Risk Score of the control group and the IVDD group. (F) RF model showed the top 16 LRGs in terms of importance. (G) Venn diagram showed the six intersected hub LRGs shared by LASSO and RF algorithms. (H) PPI network of 6 hub LRGs and 20 highly related genes. (I) GO enrichment analysis based on the 26 screened genes.

To refine the LRGs, we identified 16 genes via Venn diagrams (Figure 5A). LASSO regression was subsequently employed to further eliminate redundancy, resulting in the selection of seven potential genes (Figure 5B,C). Furthermore, the LASSO machine learning model was assessed using ROC analysis, with an area under the curve (AUC) value of 0.941 (Figure 5D). Moreover, the risk score for IVDD was found to be significantly higher than that of the control group, suggesting that these genes have potential as diagnostic markers for IVDD (Figure 5E). The RF algorithm, which ranks genes based on their importance scores, identified the top 16 candidate genes (Figure 5F). By intersecting the results from both algorithms, six genes (CBX3, THUMPD1, DDIT4, IGFBP3, RBM10 and CHST1) were identified as potential core genes (Figure 5G). Figure 5H illustrates the interaction between 6 hub genes and 20 highly related genes. Finally, GO analysis was carried out to explore the GO terms enriched by these 26 genes. The GO enrichment analysis suggested that these genes are enriched in peptidyl‐proline hydroxylation, regulation of cellular carbohydrate metabolic process and neurotrophin signalling pathway in the BP category. In the CC category, hub genes are involved in endoplasmic reticulum lumen and nuclear inner membrane. The main changes in MF were sulfotransferase activity, procollagen‐proline dioxygenase activity and death receptor binding (Figure 5I).

### 3.5 Construction of a Predictive Nomogram and Validation of LRGs

**FIGURE 6:**
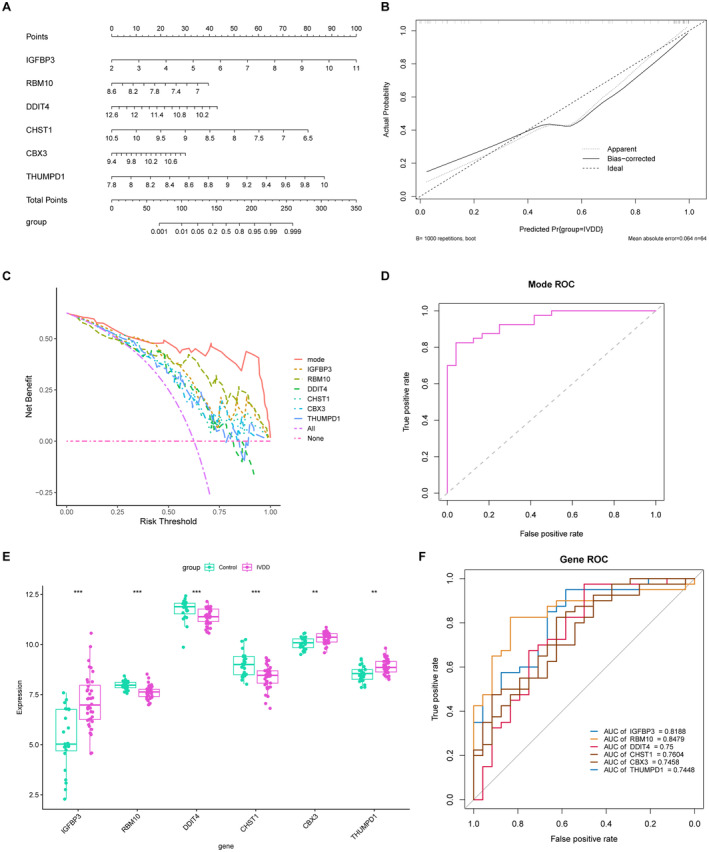
Construction of a predictive nomogram and validation of LRGs. (A) Construction of a nomogram to predicting the risk of IVDD based on six LRGs. (B) Calibration curves of the nomogram prediction. (C) DCA curves of the nomogram prediction. (D) ROC curve of nomogram model. (E) Expression of six hub LRGs in IVDD samples and control samples in the GSE13904 datasets. (F) ROC curves of the six LRGs.

To further confirm the clinical relevance of the identified six LRGs, a nomogram model was performed (Figure 6A). The calibration curve illustrated excellent concordance between predicted and observed outcomes (Figure 6B). Additionally, the decision curve analysis (DCA) for the integrated model of six genes is depicted in Figure 6C. Additionally, the area under the model ROC curve was 0.814, suggesting that the predictive model performs well (Figure 6D). In the established training set, the expression of IGFBP3, CBX3 and THUMPD1 was significantly elevated in the IVDD group, whereas the expression of RBM10, DDIT4 and CHST1 was notably lower than those in the control group (Figure 6E). The ROC curves for these hub genes exhibited AUC values of 0.819, 0.848, 0.75, 0.760, 0.746 and 0.745, respectively, underscoring their potential as valuable biomarkers for IVDD (Figure 6F).

### 3.6 Immune Characteristics and Interaction Function of LACRGs

**FIGURE 7:**
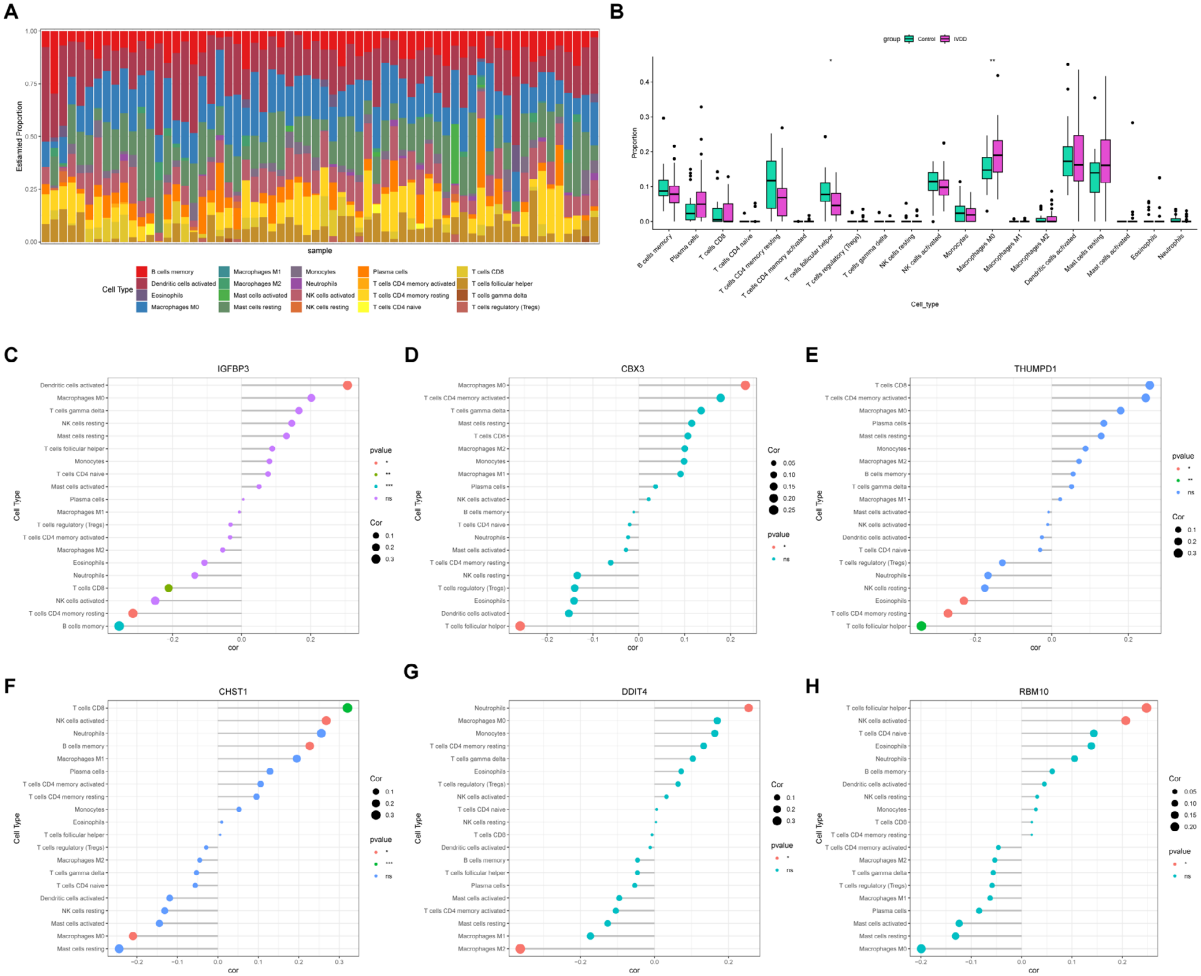
Immune characteristics and interaction function of LRGs. (A) The bar plot shows the distribution of 20 distinct immune cells across various IVDD samples. (B) The box plot illustrates the expression profiles of 20 immune cells in the IVDD group and the control group. (C–H) Correlations of C IGFBP3, D CBX3, E THUMPD1, F CHST1, G DDIT4 and H RBM10 with immune cell infiltration.

The progression of IVDD escalates inflammatory cytokine levels within IVD tissues, promoting immune cell infiltration and thereby amplifying the inflammatory cascade. To further assess the immune characteristics in IVDD, the CIBERSORT algorithm was performed. Figure 7A depicts the distribution of 20 immune cell types per sample, whereas Figure 7B displays the differential levels of immune cells between the control and IVDD groups. Among them, follicular helper T cells were underexpressed in the IVDD group, whereas Macrophages M0 was notably elevated compared to the control group. Subsequently, correlation analysis was conducted to investigate the relationships between infiltrating immune cells and hub genes. The results revealed that IGFBP3 had positive correlation with dendritic cells activated and negative correlations with CD8 T cells, NK cells activated, resting memory CD4 T cells and memory B cells (Figure 7C). CBX3 positively correlated with Macrophages M0 but negatively with follicular helper T cells (Figure 7D). THUMPD1 was found to be negatively correlated with eosinophils, resting memory CD4 T cells and follicular helper T cells (Figure 7E). CHST1 showed the most positively significant correlation with CD8 T cells, but displayed a negative correlation with macrophages M0 (Figure 7F). DDIT4 had the strongest positive link with neutrophils but negatively with macrophages M2 (Figure 7G). RBM10 correlated positively with follicular helper T cells and NK cells activated (Figure 7H). Taken together, these findings suggested that the six hub LRGs significantly influence the immune microenvironment associated with the progression of IVDD.

### 3.7 Glycolysis Was Excessively Activated During IVDD

**FIGURE 8:**
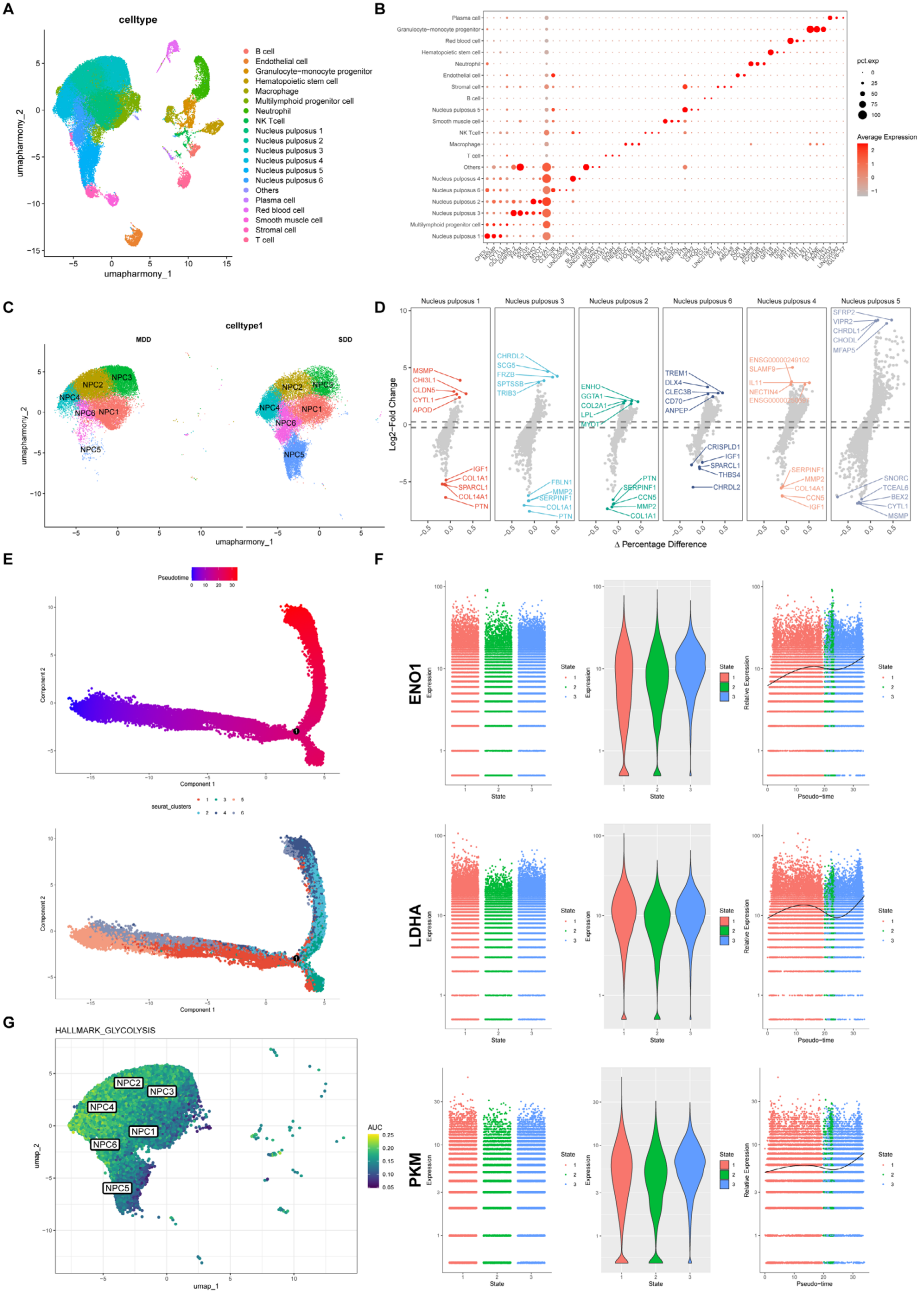
Single‐cell RNA sequence analysis revealed the increased glycolysis level in degenerated nucleus pulposus tissue. (A) UMAP visualisation of the scRNA‐seq profiles revealed 20 manually annotated clusters. (B) Dot plot shows the expression levels of selected representative genes among clusters. (C) NPCs were separated into five clusters by UMAP. (D) Differentially expressed gene per cluster (top 5). (E) Monocle pseudotime trajectory axis revealed the progress of NPC. (F) Expression of key glycolytic enzymes in developmental stages. (G) UMAP plots shows the score of glycolysis in different NPC clusters.

Utilising publicly available scRNA‐seq data of IVDD, a total of 20 major cell clusters were identified (Figure 8A). The expression profile of the representative markers of the cell type was displayed in a dot plot, and the cellular subtypes could be distinguished (Figure 8B). Simultaneously, the distribution of each NPC cluster in MDD and SDD tissues was analysed, and the NPC5 and NPC6 clusters were mainly distributed in the SDD group and closely related to IVDD (Figure 8C). Differential expression analysis was conducted, and the top five genes are shown in Figure 8D.

To further explore the cellular trajectories of distinct NPCs clusters, we utilised the Monocle pseudotime algorithm for pseudotime analysis, as depicted in Figure 8E. In the pseudotime analysis, shades of red represent the progression of cell differentiation over time, with purple shades indicating earlier stages of differentiation. Figure 8F shows enhanced expressions of glycolytic enzymes in the SDD group, including ENO1, LDHA and PKM, especially in late‐stage NPCs. In addition, the UMAP distribution of glycolysis in NPCs is shown in the scatter plot, revealing the most statistically significant alterations in glycolysis for NPC5 (Figure 8G). These findings robustly confirmed the upregulation of glycolysis in late‐stage NPCs during the progression of IVDD.

### 3.8 The Expression of CBX3 Was Upregulated During IVDD

**FIGURE 9:**
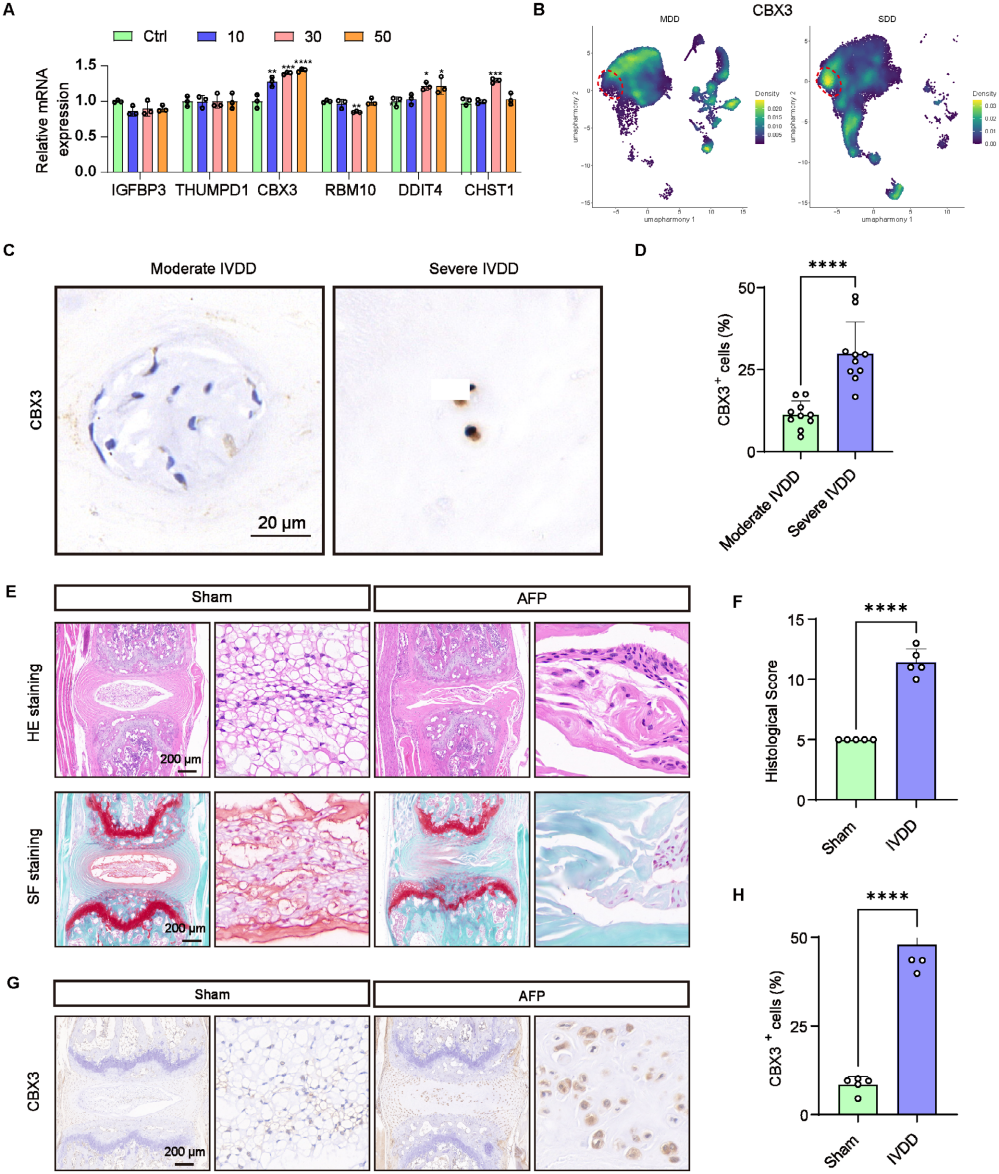
Validation of the expression of six hub LRGs. (A) Relative mRNA expression levels of six LRGs of NPCs treated with different dose IL‐1β (0, 10 30 50 ng/mL, 24 h). (B) UMAP visualisation displayed the expression of CBX3 in the MDD and SDD groups. (C, D) IHC staining and qualification analysis showing the expression of CBX3 in human disc degeneration tissues. Scale bar = 20 μm. (E, F) Representative images of HE staining, SF staining and histological scores in sham and AFP groups. Scale bar = 200 μm. (G, H) IHC staining and qualification analysis of CBX3 in the sham and AFP groups. Scale bar = 200 μm. *p < 0.05, **p < 0.01, ***p < 0.001 and ****p < 0.000.

To further validate the expression of the six LRGs, we established an NPCs degeneration model induced by Il‐1β. Among the hub genes examined, only CBX3 exhibited consistent expression patterns with our DEGs analysis, which is increased significantly in a dose‐dependent (0, 10, 30, 50, ng/mL, 24 h, Figure 9A). In addition, the UMAP visualisation displayed that CBX3 expression was significantly upregulated in the SDD group (Figure 9B). To further validate these findings, we measured the expression of CBX3 in moderately and severely degenerated human IVDs. Consistently, IHC staining confirmed elevated level of CBX3 in severely degenerated discs (Figure 9C,D). Furthermore, the change of CBX3 expression was evaluated in IVDD mouse model induced by AF needle puncture. The HE and SF staining revealed indistinct boundary between the NP and AF, indicating that the IVDD model was successfully established (Figure 9E,F). IHC staining demonstrated significantly higher CBX3 expression in the AFP group compared to the sham group (Figure 9G,H). Taken together, these comprehensive findings validated CBX3 as a critical hub gene in the progression of IVDD.

### 3.9 Screening for Potentially Effective Molecules Targeting CBX3 Through Molecular Docking

To identify the potential molecules that effectively target CBX3, we conducted a search for protein structures from PDB using CBX3 as a keyword. Specifically, we filtered for human species and identified CBX3_HUMAN (PDB ID:3TZD) as the relevant protein structure. Concurrently, we downloaded 3158 small molecule structures from FDA‐Approved & Pharmacopeia Drug Library (L1010; Opscience Co. Ltd.). In each molecular docking simulation, we generated 10 binding conformations and selected the best binding score for sorting. After eliminating duplicates, we identified the top 10 small molecules with robust binding effects.

**FIGURE 10:**
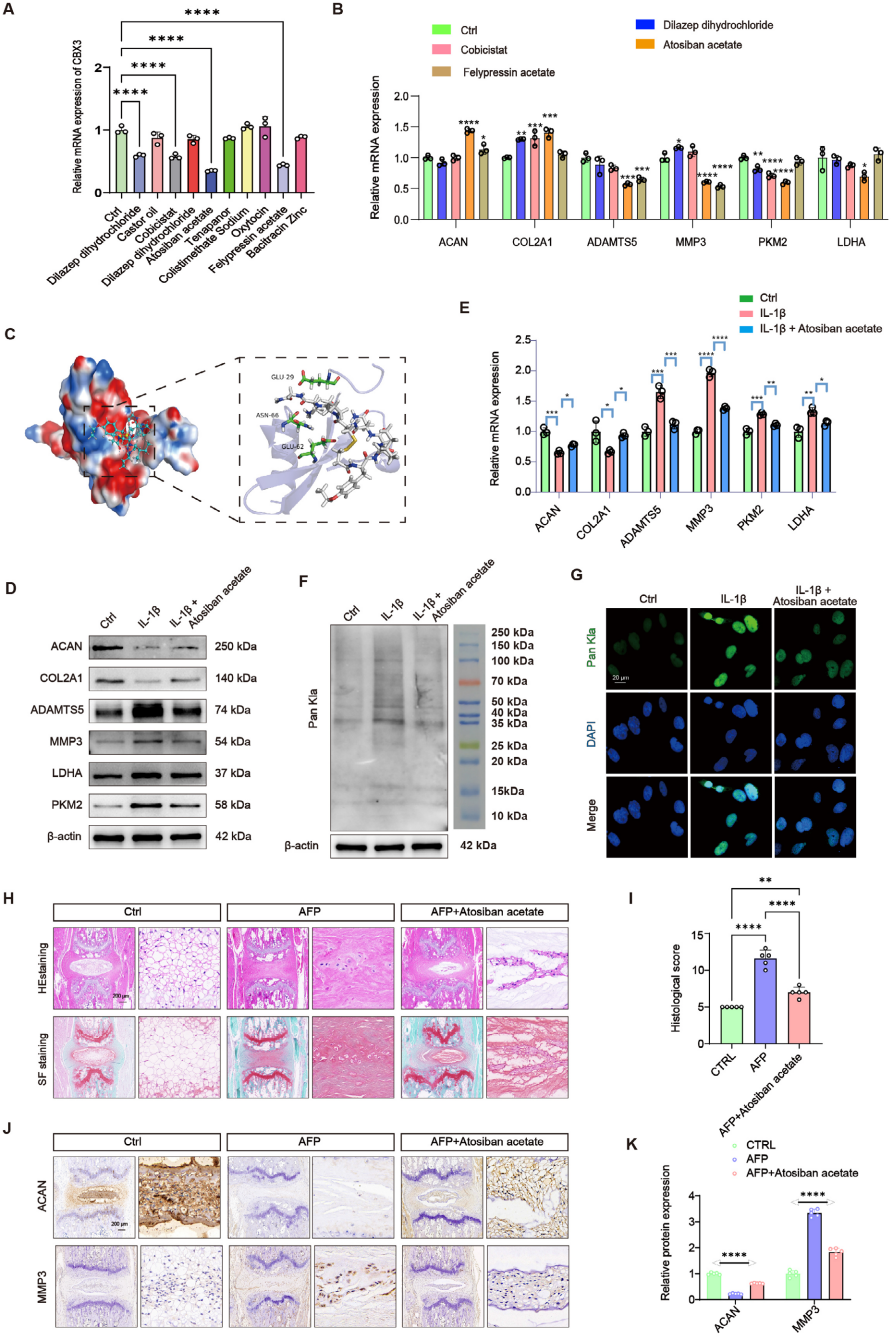
Atosiban acetate as effective molecule alleviated NPC degeneration via repressing the glycolysis activity and global lactylation level. (A) Relative mRNA expression levels of CBX3 in NPCs treated with different molecules (10 μM, 24 h). (B) Relative mRNA expression levels of ACAN, COL2A1, ADAMTS5, MMP3, LDHA and PKM2 in NPCs after dilazep dihydrochloride, cobicistat, atosiban acetate and felypressin acetate. (C) Molecular docking of CBX3 and atosiban acetate. (D) Western blot analysis and quantification showing the protein expression of ACAN, COL2A1, ADAMTS5, MMP3, LDHA and PKM2 in NPCs in response to IL‐1β and atosiban acetate. (E) Relative mRNA expression levels of ACAN, COL2A1, ADAMTS5, MMP3, LDHA and PKM2 in NPCs in response to IL‐1β and atosiban acetate. (F) Western blot analysis showing the protein expression of Pan Kla in NPCs in response to IL‐1β and atosiban acetate. (G) IF staining showing the expression of Pan Kla in NPCs in response to IL‐1β and atosiban acetate. Scale bar = 20 μm. (H, I) Representative images of HE staining, SF staining and histological scores in different groups. Scale bar = 200 μm. (J, K) IHC staining and qualification analysis of ACAN and MMP3 in different groups. Scale bar = 200 μm. *p < 0.05, **p < 0.01, ***p < 0.001 and ****p < 0.0001.

To further explore the interaction between these small molecules and CBX3, the mRNA expression of CBX3 in NPCs treated with these molecules was assessed. As the results showed, dilazep dihydrochloride, cobicistat, atosiban acetate and felypressin acetate decreased the mRNA expression of CBX3, with atosiban acetate exhibiting the most significant decrease (Figure 10A). In addition, unlike the other three small molecule compounds, only atosiban acetate simultaneously increased the expression of ACAN and COL2A1 and decreased ATAMTS5, MMP3, PKM2 and LDHA (Figure 10B). The molecular docking indicated that atosiban acetate bound to GLU‐29, ASN‐66 and GLU‐62 of CBX3. Notably, the docking energy between atosiban acetate and CBX3 was −9.3434 kcal/mol, indicating a strong binding capability to CBX3 (Figure 10C).

### 3.10 Atosiban Acetate Restored the Balance of Lactylation and Alleviated IVDD

To determine the effects of atosiban acetate on IVDD, various concentrations of atosiban acetate (1, 5, 10, 20 and 50 μM) were added into NPCs, followed by the CCK8 assay to assess the cell viability. The results indicated that atosiban acetate most noticeably improved the viability of NPCs at a concentration of 10 μM (Figure S1A). Then, we established an IL‐1β‐induced NPCs model and treated with atosiban acetate (10 μM, 24 h). As expected, the WB results revealed that the ACAN and COL2A1 were decreased while MMP3 and ADAMTS5 were increased after IL‐1β treatment, a phenomenon that was mitigated by atosiban acetate (Figure 10D), which was quantified in Figure S1B–E. Moreover, glycolysis was excessively activated by IL‐1β, including LDHA and PKM2, and this effect could be partially ameliorated by atosiban acetate (Figure 10D), which was quantified in Figure S1F,G. The RT‐qPCR results showed the same trend (Figure 10E). The effect of atosiban acetate on lactylation was tested subsequently. As demonstrated in WB, IL‐1β treatment resulted in elevated lactylation level, which was reversed partly by atosiban acetate (Figure 10F). IF staining (Figure 10G) further verified the results of WB, which was quantified in Figure S1H. Furthermore, to transcend the limitations of the above in vitro experiments, we administered atosiban acetate to an IVDD mouse model to verify its role in alleviating the degeneration of IVDs through in vivo experiments. The histological results, including HE and SF staining, showed that the boundary between the NP and the AF tissues was significantly disrupted in the AFP group, and this effect was alleviated by atosiban acetate (Figure 10H,I). The IHC results demonstrated that atosiban acetate could reversed the decreased ACAN and the increased MMP3 in the IVDD mouse model (Figure 10J,K). Collectively, this part data confirmed that atosiban acetate could ameliorate IVDD and restore the balance of lactylation.

## 4 Discussion

Recently, growing evidence has shown that lactate is not only one of the most important direct metabolic products from cells, it also functions as a signalling regulator to mediate protein lactylation for the progression of diseases. We initially established a clear link between lactylation and IVDD, revealing an increase in lactylation levels correlating with the severity of IVDD. Then, six hub LRGs were determined and a lactylation score model was constructed, which was closely associated with increased severity of IVDD. Analysis of scRNA‐seq data further confirmed the activation of glycolysis and the expression changes of these hub genes. Among the six genes, CBX3 was the most upregulated in both in vivo and in vitro experiments. Notably, inhibiting the expression of CBX3 significantly repressed the glycolysis activity and global lactylation level, thus alleviating NPCs degeneration. Therefore, the identification and validation of these hub genes and biomarkers offered critical insights into the molecular mechanisms underlying IVDD and potentially led to the development of novel therapeutic strategies from the perspective of protein lactylation.

Due to the hypoxic conditions of IVDs, anaerobic glycolysis serves as the primary energy source for intervertebral disc cells, particularly NPCs. Unlike oxidative phosphorylation (OxPhos), which requires oxygen and is extremely efficient, glycolysis provides a rapid yet less efficient way to generate ATP [35]. Studies have highlighted the role of hypoxia‐inducible factor (HIF‐1α) in upregulating glycolysis and suppressing OxPhos in NPCs, suggesting their adaptation to low oxygen microenvironment [36]. Energy metabolism plays a crucial role in cellular function and is often disrupted during disease states, notably under chronic inflammatory conditions. Recent studies have shown that NPCs can undergo metabolic alterations in response to various stimuli [15, 37]. The glycolytic metabolism of NPCs in a hypoxia microenvironment is further intensified due to diminished glucose and oxygen supply, a consequence of reduced CEP permeability during IVDD [6, 38], which increases the lactate production. Elevated lactate levels in turn significantly induce anabolic downregulation and catabolic upregulation in acid‐sensitive NPCs, leading to cell senescence, ECM imbalance and degradation [39]. Consistent with previous research, we found that the expression of key glycolytic enzymes was upregulated in degenerative NPCs, including ENO1, LDHA and PKM2 based on the results of scRNA‐seq. Therefore, reprogramming glycolysis in NPCs may be a promising strategy to maintain or restore their physiological functions, thus potentially ameliorating the progression of IVDD.

Lactate, as the main product of glycolysis, has been found in association with IVDD. Bartels et al. [40] demonstrated that the lactate concentration peaked at the centre of the disc in patients experiencing back pain, typically ranging from 2 to 6 mM. Accumulation of lactate could acidify the disc microenvironment, impairing cell viability and function. Ohshima and Urban [39] found that elevated lactate concentration inhibited the synthesis of proteoglycan in discs, resulting in a reduction in proteoglycan content and ultimately to IVDD. Through in vitro experiments, Wu et al. [41] revealed that NPCs underwent apoptosis in 6 mM lactate (a high lactate concentration), whereas 2 mM lactate (a low lactate concentration) did not induce apoptosis. Additionally, they found higher expression of type II collagens in NPCs with 2 mM lactate compared with the control group, whereas higher expression of type I collagens was observed in 6 mM lactate group. These findings suggest that while low lactate concentrations may protect NPCs, high concentrations of lactate present a significant disadvantage for tissue repair.

Recently, PTMs have been shown to be involved in IVDD [34, 42, 43]. Lactylation, a critical PTM driven by glycolysis‐derived lactate, could directly activate gene transcription [8]. To date, research on lactylation has examined both histone and nonhistone proteins. Histone Kla exhibits unique temporal dynamics distinct from histone acetylation. Functionally, histone lactylation is important for various biological processes, such as macrophage M1/2 polarisation [44]. Histone Kla levels remarkably raised at the promoters of M2‐like genes during the later stages of M1 macrophage polarisation, suggesting that it may act as a ‘lactate clock’ facilitating the transition from an inflammatory to a steady‐state phenotype in macrophages. Beyond histones, lactylation also modifies nonhistone proteins. Yang et al. [12] confirmed that lactate could mediate the nuclear‐to‐cytoplasmic transfer of HMGB1 via MCTs and GPR81, increasing HMGB1 lactylation, thereby leading to endothelial barrier dysfunction and promoting sepsis. In a recent study, Zhang et al. [6] proved that glutamine could prevent IVDD by inhibiting glycolysis and decreasing AMPKα lactylation, which promotes autophagy and suppresses the senescence of NPCs. These findings suggest that lactylation is integral to the pathogenesis of IVDD and that targeting metabolic disorders could offer potential strategies for preventing IVDD.

In this study, we extracted human NP tissues with varying degeneration degrees based on the Pfirrmann grades. Our results revealed that the lactylation level was increased with the progression of IVDD, suggesting that lactylation was closely involved in IVDD. These findings furnish us with the groundwork and assurance to embark on data‐mining strategies and comprehensive bioinformatics analysis in next steps. Using a combination of the WGCNA model, LASSO analysis and RF algorithms, we identified six characteristic LRGs, CBX3, THUMPD1, DDIT4, IGFBP3, RBM10 and CHST1, which are linked to carbohydrate metabolic process, sulfotransferase activity and death receptor binding. CBX3, a chromobox homologue 3, is a highly conserved nonhistone chromosomal protein. High expression of CBX3 has been found in various cancers, where it functions as an oncogene by regulating multiple biological processes in cancer cells [45, 46, 47]. Chen et al. [48] revealed that CBX3 positively regulates aerobic glycolysis by downregulating the glycolysis genes FBP1 in pancreatic cancer cells. DDIT4, as a mammalian target of rapamycin (mTOR) inhibitor, could inhibit lipopolysaccharide‐induced glycolysis and promote oxidative phosphorylation [49]. Previous studies have identified DDIT4 as a contributor to IVDD, with its expression notably elevated in severely degenerated intervertebral disc tissues [50, 51]. Ma et al. [51] demonstrated that DDIT4 was upregulated under H_2_O_2_‐induced mitochondrial damage, and its interaction with TXNIP activated the classic pyroptosis pathway. THUMPD1 is a specific adaptor protein, which could modulate tRNA acetylation through interacting with NAT10, a human acetyltransferase known for modifying histone and microtubule [52]. Havugimana et al. [53] indicated that THUMPD1 interacted with Yes‐associated protein (YAP), which is essential for tumour proliferation and invasion. IGFBP3, known as the insulin‐like growth factor binding protein 3 in yeast, is implicated in various cellular processes, including cell differentiation, proliferation and apoptosis [54, 55]. Recent research has confirmed that IGFBP3 acts as a key regulator in cancer development and progression, which could regulate aerobic glycolysis through the PI3K/AKT pathway [56]. Kazezian et al. [57] identified that IGFBP3 was upregulated in human degenerated AF via activation of IFNα2β signalling. RBM10 (S1‐1), one of the most important members of the RNA binding motif gene family, functions as an alternative RNA splicing factor involved in regulating gene expression. Recent studies indicated that RBM10 was involved in many tumours, highlighting its potential as a target for new therapeutics [58, 59]. CHST genes encode Golgi‐localised enzymes, such as sulfotransferase and keratan sulfotransferase, which instal sulphate at specific sites in glycans. While some literature demonstrated that enhanced CHST1 was related to tumour development [60, 61], its role in IVDD remains unexplored. In this study, a predictive nomogram based on the six hub LRGs was developed, which demonstrated a good discrimination and calibration capacity, suggesting their potential as novel diagnostic markers for IVDD.

Given the significance of the immune response in IVDD, we employed the CIBERSORT algorithm to analyse the composition and proportion of infiltrating immune cells in both control and IVDD samples. Our findings revealed significant changes in immune cell infiltration in IVDD samples, characterised by increased M0 macrophages and decreased follicular helper T cells. Additionally, a correlation analysis between six hub LRGs and the relative abundance of 20 immune cell types revealed significant associations. These hub genes exhibited notable correlations with multiple immune cell types, indicating their involvement in the immunological processes. Thus, these findings suggested a vital role for these hub LRGs in modulating the immune response associated with IVDD, potentially opening up avenues for immunometabolic therapies.

Finally, we designed an IL‐1β‐induced degeneration model in NPCs to validate the differential expression of six hub LRGs. Based on the RT‐qPCR results, unlike other hub genes, the expression of CBX3 was dose‐dependently upregulated, and the expression trend was consistent with the result of bioinformatics analysis. Thus, CBX3 was identified as a key gene and showed a promising clinical diagnostic value through ROC curve analysis. Numerous studies have demonstrated that CBX3 played significant roles in various cancers by regulating multiple mechanisms including heterochromatin formation, gene silencing and DNA replication. However, to date, there has been no report on the function of CBX3 in IVDD. In the current study, we demonstrated that CBX3 was significantly upregulated in IVDD by analysing clinical samples, animal models and cellular models, indicating the association between CBX3 and IVDD. As far as we are aware, this study pioneers the identification of CBX3 as a diagnostic biomarker for IVDD utilising a publicly accessible GEO dataset and an extensive bioinformatics strategy. This adds a new dimension to the pathophysiological understanding of IVDD by connecting metabolic regulation and lactylation.

More importantly, through molecular docking, we identified atosiban acetate as a potentially therapeutic small molecule. Atosiban acetate, an oxytocin receptor antagonist, was considered a first‐line tocolytic agent for the treatment of preterm birth. Additionally, a previous study has demonstrated that atosiban could regulate proinflammatory cytokine expressions and gastric lipid peroxidation in the stomachs of exercised rats with ulcers [33]. As a small molecular compound, atosiban acetate showed good binding affinity with CBX3 and decreased CBX3 expression, indicating that atosiban acetate may be a potential drug through suppressing CBX3. Treatment with atosiban acetate will prevent ECM degeneration, including increased expression of ACAN and COL2A1, and decreased expression of MMP3 and ADAMTS5, suggesting that atosiban acetate could ameliorate IVDD. Previous research has reported that silencing CBX3 could inhibit aerobic glycolysis in pancreatic cancer by enhancing the expression of FBP1, a negative regulator of aerobic glycolysis [48]. In this study, we indicated that the expression of key glycolytic enzymes was increased in degenerative NPCs and atosiban acetate could decrease the expression of glycolytic enzymes, indicating that the functional effect of atosiban acetate on inhibiting glycolysis through downregulating the expression of CBX3. Lactate, an end product of glycolysis, is involved in lactylation process, and decreases in lactate could alleviate lactylation. Zhang et al. [6] proved that lactylation was involved in IVDD and reducing lactylation could inhibit senescence and promote cellular matrix synthesis. Consistently, our results showed that lactylation level was upregulated with the progression of IVDD, and atosiban acetate could significantly inhibit lactylation, thereby alleviating IVDD. Collectively, the identification of the linkages between CBX3, atosiban acetate, glycolysis and lactylation offers insights into the pathogenesis of IVDD and suggests potential strategies for IVDD prevention. However, further research is needed to fully elucidate the mechanism of lactylation.

This study has limitations. Firstly, we did not conduct lactylation modification proteomic sequencing, which prevents a comprehensive description of the changes in lactylation modifications of various proteins during the process of IVDD. This will be addressed in future studies. Secondly, we did not establish CBX3 gene knockout mice to investigate its effects on IVDD and lactylation modification. Further animal and clinical studies are needed to elucidate the intrinsic molecular mechanisms.

## 5 Conclusions

In conclusion, we performed a comprehensive analysis to explore the impact of LRGs on IVDD and identified six hub genes that could serve as novel diagnostic markers for IVDD. Moreover, CBX3 was identified as a key characteristic LRG in IVDD, which was upregulated and tested in the validation set. Furthermore, new targets for small molecules targeting CBX3 in IVDD treatment were identified. The results demonstrated that lactylation plays a crucial role in IVDD pathogenesis, and atosiban acetate may be a promising therapeutic option for IVDD by modulating lactylation.

## Author Contributions

**Yangyang Shi:** project administration (equal), writing – original draft (equal). **Fudong Li:** data curation (equal). **Wenbo Lin:** funding acquisition (equal), methodology (equal). **Linhui Han:** software (equal). **Jinyu Wang:** validation (equal). **Chen Yan:** formal analysis (equal). **Jingchuan Sun:** investigation (equal). **Chenglong Ji:** writing – review and editing (equal). **Jiangang Shi:** funding acquisition (equal), visualization (equal). **Kaiqiang Sun:** conceptualization (equal), funding acquisition (equal).

## Ethics Statement

All procedures performed involving human patients were approved by the ethics committee of Shanghai Changzheng Hospital. All animal studies were performed following protocols approved by the Laboratory Animal Center, Naval Medical University.

## Consent

Informed consent was obtained from all subjects involved in the study.

## Conflicts of Interest

The authors declare no conflicts of interest.

## Supporting information


**Figure S1.** (A) CCK8 assay showing the cell viability in NPCs stimulated with various concentration of atosiban acetate. (B–G) Semiquantitative analysis and statistical analysis showing the protein expression of ACNA, COL2A1, ADAMTS5, MMP3, LDHA and PKM2. (H) Statistical analysis showing relative fluorescence intensity of Pan Kla.

**Table S1.** List of 332 LRGs selected based on previous research.

**Table S2.** The underlying R code for bioinformatic analysis.

**Table S3.** The detailed list of GO and KEGG analysis in normal and degenerated human IVD tissue.

## Data Availability

The authors are able to provide the data generated by the analysis of this study upon reasonable request.
