# Correction to ‘Integrating Bulk RNA and Single‐Cell RNA Sequencing Identifies and Validates Lactylation‐Related Signatures for Intervertebral Disc Degeneration’

**DOI:** 10.1111/jcmm.71061

**Published:** 2026-03-17

Y. Shi, F. Li, W. Lin, L. Han, J. Wang, C. Yan, J. Sun, C. Ji, J. Shi, and K. Sun, “Integrating Bulk RNA and Single‐Cell RNA Sequencing Identifies and Validates Lactylation‐Related Signatures for Intervertebral Disc Degeneration,” *Journal of Cellular and Molecular Medicine* 28, no. 23 (2024): e70262. https://doi.org/10.1111/jcmm.70262

In the article, there was an error in Figure 10H. The hematoxylin‐eosin (HE) and safranin O and fast green (SF) image of the Ctrl group in Figure 10H are incorrect. This error arose from an inadvertent typesetting oversight during the figure assembly process. Specifically, due to file naming similarities, an incorrect image was accidentally selected and placed in the ctrl panel instead of the correct representative image. These corrections do not alter the results or conclusions of the article.

The correct Figure 10H is shown below.

**FIGURE 10:**
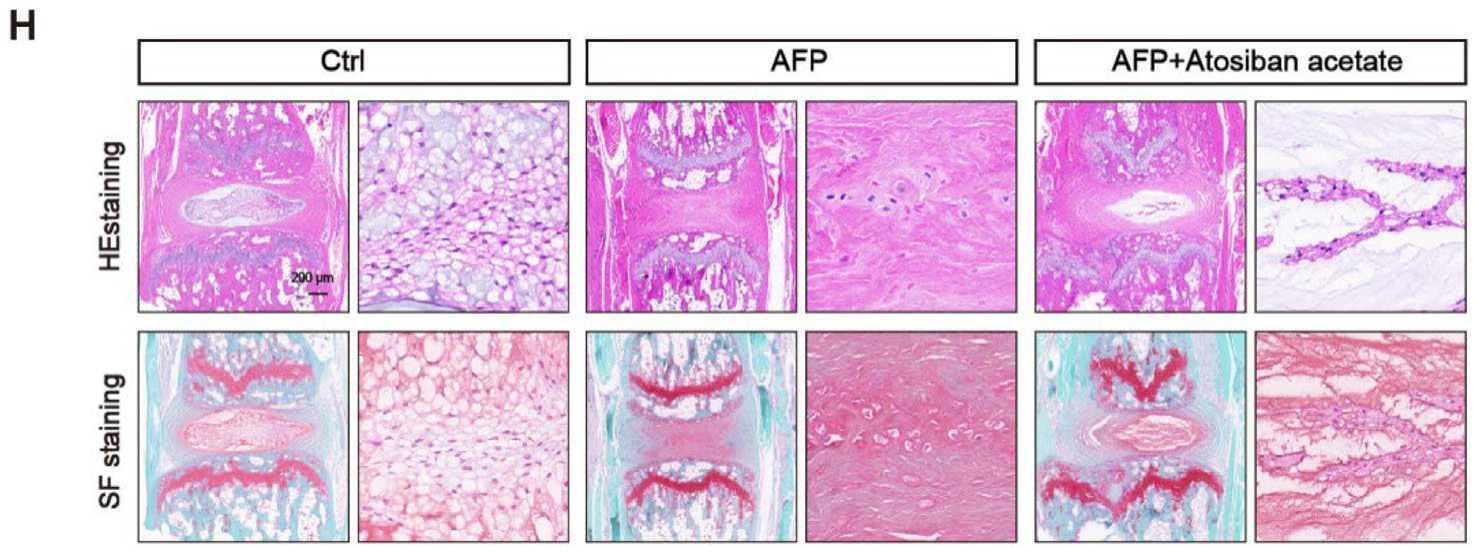
(H) Atosiban acetate as an effective molecule alleviated NPC degeneration via repressing the glycolysis activity and global lactylation level. (H, I) Representative images of HE staining, SF staining, and histological scores in different groups. Scale bar = 200 μm.

We sincerely apologise for this error.

