# Supplementary material for: Integrating Bulk RNA and Single‐Cell RNA Sequencing Identifies and Validates Lactylation‐Related Signatures for Intervertebral Disc Degeneration
Source: J Cell Mol Med. 2024 Dec 5;28(23):e70262. doi: 10.1111/jcmm.70262 (PMC11619158; doi:10.1111/jcmm.70262)
Supplement: Supplementary file 1 — Figure S1. (A) CCK8 assay showing the cell viability in NPCs stimulated with various concentration of atosiban acetate. (B–G) Semiquantitative analysis and statistical analysis showing the protein expression of ACNA, COL2A1, ADAMTS5, MMP3, LDHA and PKM2. (H) Statistical analysis showing relative fluorescence intensity of Pan Kla. [file JCMM-28-e70262-s001.docx]

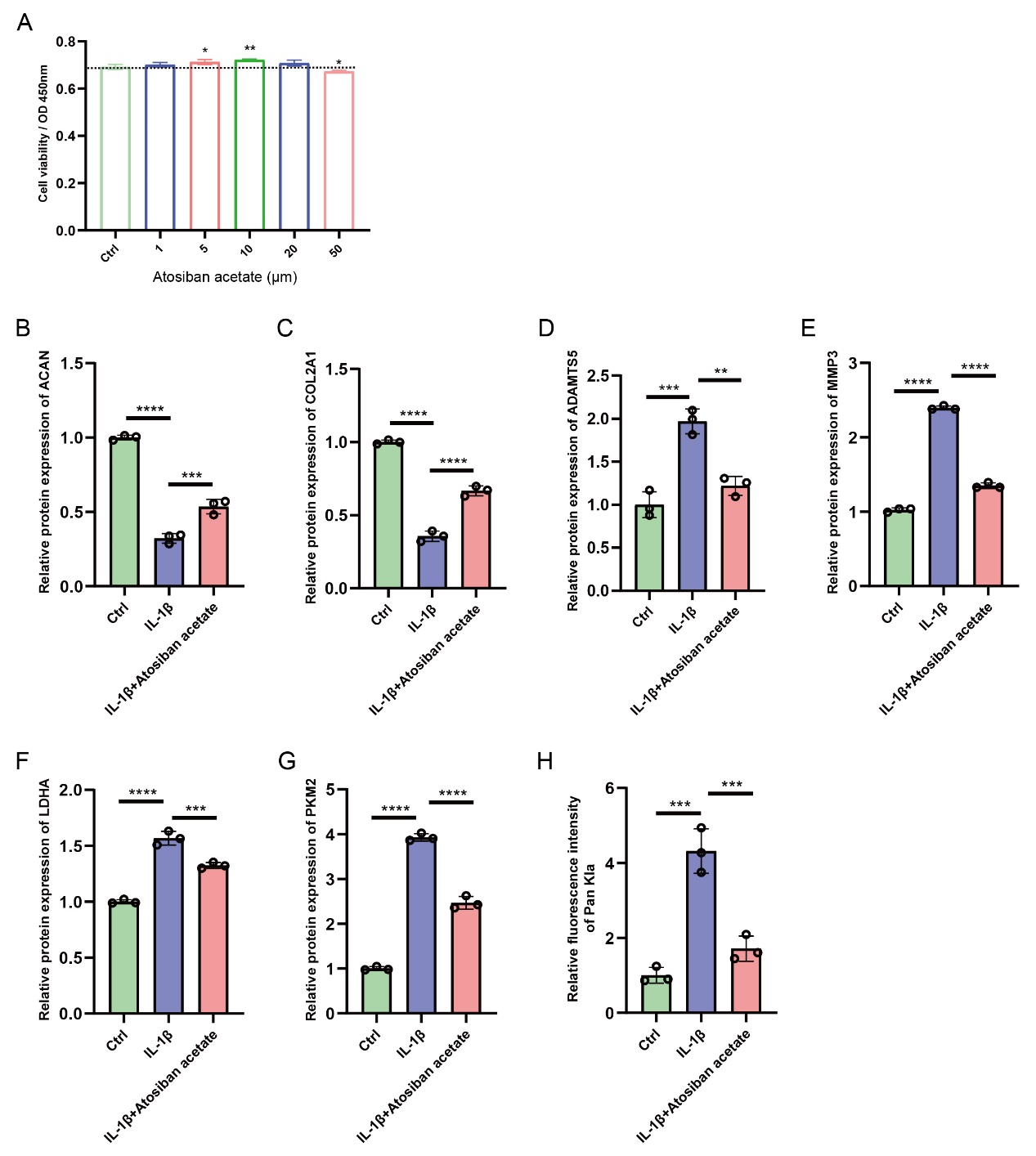


**Figure S1.** (**A**) CCK8 assay showing the cell viability in NPCs stimulated with various concentration of atosiban acetate. (**B-G**) Semiquantitative analysis and statistical analysis showing the protein expression of ACNA, COL2A1, ADAMTS5, MMP3, LDHA, and PKM2. (**H**) Statistical analysis showing relative fluorescence intensity of Pan Kla.
