# Supplementary material for: Integrating Bulk RNA and Single‐Cell RNA Sequencing Identifies and Validates Lactylation‐Related Signatures for Intervertebral Disc Degeneration
Source: J Cell Mol Med. 2024 Dec 5;28(23):e70262. doi: 10.1111/jcmm.70262 (PMC11619158; doi:10.1111/jcmm.70262)
Supplement: Supplementary file 3 — Table S2. The underlying R code for bioinformatic analysis. [file JCMM-28-e70262-s004.docx]

#################################################################

rm(list=ls())

library(Seurat)

library(tidyverse)

library(dplyr)

x <- list.files("data/GSE174219_RAW/")

library(tibble)

scRNAlist <- list()

for (i in x) {

scRNAlist[[i]] <- read.table(file = paste0("data/GSE174219_RAW/",i))

print(i)

}

x <- list.files("data/GSE210898_RAW/")

for (i in x) {

scRNAlist[[i]] <- Read10X(data.dir = paste0("data/GSE210898_RAW/",i,

"/"))

print(i)

}

names(scRNAlist) <- substring(names(scRNAlist),1,10)

for (i in names(scRNAlist)) {

scRNAlist[[i]] <- CreateSeuratObject(counts = scRNAlist[[i]],

project = i,

min.cells = 3, min.features = 200)

}

###

scRNA <- scRNAlist[[1]]

for (i in 2:length(scRNAlist)) {

scRNA <- merge(scRNA, scRNAlist[[i]])

}

scRNA[["percent.mt"]] <- PercentageFeatureSet(scRNA, pattern = "^MT-")

scRNA[["percent.rb"]] <- PercentageFeatureSet(scRNA, pattern = "^RP[SL]")

##质scRNA <- subset(scRNA, subset = nFeature_RNA > 300& nFeature_RNA < 7000 & percent.mt < 20 & nCount_RNA < 100000)

##

violin <- c("nFeature_RNA", "nCount_RNA", "percent.mt","percent.rb")

library(ggplot2)

for (i in violin) {

col.num <- length(levels(scRNA@active.ident))

p <- VlnPlot(scRNA,#

features = c("nFeature_RNA"),

cols =rainbow(col.num),

pt.size = 0.01)

ggsave(filename = paste0("output/质控/",i,".pdf"),plot = p,width = 8,height = 6)

print(i)

}

FeatureScatter(scRNA, feature1 = "nCount_RNA", feature2 = "percent.mt")

FeatureScatter(scRNA, feature1 = "nCount_RNA", feature2 = "nFeature_RNA")

#####

scRNA <- NormalizeData(scRNA, normalization.method = "LogNormalize", scale.factor = 10000)

##挑

scRNA <- FindVariableFeatures(scRNA, selection.method = "vst", nfeatures = 3000)

top10 <- head(VariableFeatures(scRNA), 10)

# plot variable features with and without labels

plot1 <- VariableFeaturePlot(scRNA)

###把top10的基因加到图中

plot2 <- LabelPoints(plot = plot1, points = top10, repel = TRUE, size=2.5)

plot <- CombinePlots(plots = list(plot1, plot2),legend="bottom")

###

plot

scale.genes <- rownames(scRNA)

scRNA <- ScaleData(scRNA, features = scale.genes)

##

scRNA <- RunPCA(scRNA, features = VariableFeatures(scRNA))

##

ElbowPlot(scRNA, ndims=50, reduction="pca")

VizDimLoadings(scRNA, dims = 1:4, reduction = "pca",ncol = 2)

DimHeatmap(scRNA, dims = 1:4, cells = 1000, balanced = TRUE,ncol = 2)

library(harmony)

##

scRNA <- RunHarmony(scRNA,

group.by.vars = "orig.ident")

######################################################################################

##

scRNA <- FindNeighbors(scRNA,

reduction = "harmony", ###一定要指定harmony

dims = 1:30) %>%

FindClusters(resolution = 0.5)

##umap

scRNA <- RunUMAP(scRNA,

reduction = "harmony", ###一定要指定harmony

dims = 1:30)

scRNA <- scRNA %>%

RunTSNE(reduction = "harmony",

dims=1:30) %>%

RunUMAP(reduction = "harmony",

dims=1:30)

DimPlot(scRNA, group.by = "orig.ident")

DimPlot(scRNA, group.by = "seurat_clusters")

markers <- FindAllMarkers(object = scRNA,

test.use="wilcox" ,

min.pct=0.1,

only.pos = TRUE,

logfc.threshold = 0.25)

all.markers =markers %>%

dplyr::select(gene, everything()) %>%

subset(p_val<0.05)

top20 = all.markers %>%

group_by(cluster) %>%

top_n(n = 20, wt = avg_log2FC)

celltype <- read.csv(file = "data/单细胞.csv")

#

scRNA@meta.data$celltype ="NA"

for(i in 1:nrow(celltype)){

scRNA@meta.data[which(scRNA@meta.data$seurat_clusters == celltype$cluster[i]),'celltype'] <- celltype$celltype[i]

}

table(scRNA@meta.data$group)

scRNA@meta.data$group <- ifelse(scRNA@meta.data$orig.ident=="GSM5289542","Control","HTN")

DimPlot(scRNA, group.by="celltype", label=T, label.size=5)

colnames(scRNA@meta.data)

p1 = DimPlot(scRNA, group.by="seurat_clusters", label=F, label.size=5, reduction = "umap")

p1

p2 = DimPlot(scRNA, group.by="celltype", label=F, label.size=5, reduction='umap')

p2

p3 = DimPlot(scRNA, group.by="group", label=F, label.size=5, reduction='umap')

p3

top10 = all.markers %>%

group_by(cluster) %>%

top_n(n = 10, wt = avg_log2FC)

DoHeatmap(scRNA,

group.by = "celltype",

label = F,

features = top10$gene) + NoLegend()

##每个分簇

Idents(scRNA) <- "celltype"

DoHeatmap(subset(scRNA, downsample = 30),

features = top10$gene,

group.by = "celltype",

label = F)

###

gene <- top10$gene[1:10]

library(reshape2)

##小提琴图

VlnPlot(scRNA, features = gene)

##

vln.df=as.data.frame(scRNA[["RNA"]]@data[gene,])

vln.df$gene=rownames(vln.df)

vln.df=melt(vln.df,id="gene")

colnames(vln.df)[c(2,3)]=c("CB","exp")

colnames(scRNA@meta.data)

anno=scRNA@meta.data

anno$CB <- rownames(anno)

##

vln.df=inner_join(vln.df,anno,by="CB")

vln.df$gene=factor(vln.df$gene,levels = gene)

##绘图

vln.df%>%ggplot(aes(seurat_clusters,exp))+

geom_violin(aes(fill=gene),scale = "width")+

facet_grid(vln.df$gene~.,scales = "free_y")+

scale_fill_brewer(palette = "Set3",direction = 1)+

scale_x_discrete("")+scale_y_continuous("")+

theme_bw()+

theme(

axis.text.x.bottom = element_text(angle = 45,hjust = 1,vjust = 1),

panel.grid.major = element_blank(),panel.grid.minor = element_blank(),

legend.position = "none"

)

#

FeaturePlot(scRNA, features = gene, ncol = 4)

##

DotPlot(scRNA,

features = gene,

scale=T) +

RotatedAxis()

colnames(scRNA@meta.data)

Idents(scRNA) <- "celltype"

table(scRNA$celltype)#

prop.table(table(Idents(scRNA)))

table(Idents(scRNA), scRNA$group)#

Cellratio <- prop.table(table(Idents(scRNA), scRNA$orig.ident), margin = 2)#计算各组样本不同细胞群比例

Cellratio

Cellratio <- as.data.frame(Cellratio)

colourCount = length(unique(Cellratio$Var1))

library(ggplot2)

ggplot(Cellratio) +

geom_bar(aes(x =Var2, y= Freq, fill = Var1),stat = "identity",width = 0.7,size = 0.5,colour = '#222222')+

theme_classic() +

labs(x='Sample',y = 'Ratio')+

#coord_flip()+

theme(panel.border = element_rect(fill=NA,color="black", size=0.5, linetype="solid"))

table(scRNA@meta.data$celltype)

table(scRNA@meta.data$group)

scRNAlist <- SplitObject(scRNA, split.by = "celltype")

names(scRNAlist)

scRNA1 <- scRNAlist[["Proximal tubule cells"]]

options(future.globals.maxSize= 10*891289600)

##

table(scRNA1@meta.data$group)

degdf <- FindMarkers(scRNA1,

ident.1 = "HTN",#实验组

ident.2 = "Control", #对照组

logfc.threshold = 0,

group.by = "group",

verbose = FALSE)

logFC=0.1

P.Value = 0.05

k1 = (degdf$p_val < P.Value)&(degdf$avg_log2FC < -logFC)

k2 = (degdf$p_val < P.Value)&(degdf$avg_log2FC > logFC)

degdf$change = ifelse(k1,"down",ifelse(k2,"up","stable"))

table(degdf$change)

##################################################################

library(Seurat)

#####

load(file = "../01_juleizhushi/output/scRNA.Rdata")

#

expr <- scRNA@assays$RNA@counts

expr <- as.matrix(expr)

expr[1:10,1:10]

meta <- scRNA@meta.data

meta[1:10,]

##

expr <- matr.filter(expr, min.cells = 10, min.genes = 10)

ncol(expr)

##

meta <- meta[colnames(expr),]

#

ent.res <- SE_fun(expr)

head(ent.res)

SEplot(ent.res)

rogue.value <- CalculateRogue(ent.res, platform = "UMI")

rogue.value

rogue.res <- rogue(expr,

labels = meta$celltype,

samples = meta$time,

platform = "UMI",

span = 0.9)

rogue.res

rogue.boxplot(rogue.res)

p <- rogue.boxplot(rogue.res)

p + theme(axis.text.x = element_text(angle = 45, hjust = 1))

##################################################################

load("/output/scRNA.Rdata")

##BiocManager::install("AUCell")

library(AUCell)

library(ggplot2)

table(scRNA@meta.data$celltype)

table(scRNA@meta.data$orig.ident)

scRNA@meta.data$group <- ifelse((scRNA@meta.data$orig.ident=="SRR11038989")|(scRNA@meta.data$orig.ident=="SRR11038990")|(scRNA@meta.data$orig.ident=="SRR11038991"),"PBMC_TB",

ifelse((scRNA@meta.data$orig.ident=="SRR11038992")|(scRNA@meta.data$orig.ident=="SRR11038993"),"PBMC_LTBI", "PBMC_HC"))

table(scRNA@meta.data$group)

scRNAlist <- SplitObject(scRNA, split.by = "celltype") #

scRNA1 <- scRNAlist[["Macrophage"]]

#

scRNA1@meta.data$type <- ifelse(scRNA1@meta.data$group==project[i],"Other","all")

scRNAlist <- SplitObject(scRNA1, split.by = "type") #也可以按别的指标（metadata中的）来进行拆分，比如可以按不同的分组来拆分样本，再进行整合。

names(scRNAlist)

scRNA2 <- scRNAlist[["all"]]

options(future.globals.maxSize= 10*891289600)

table(scRNA2@meta.data$celltype)

table(scRNA2@meta.data$group)

unique(scRNA2@meta.data$group)[1]

degdf <- FindMarkers(scRNA2,

ident.1 = unique(scRNA2@meta.data$group)[1],#

ident.2 = unique(scRNA2@meta.data$group)[2],

logfc.threshold = 0,

group.by = "group",

verbose = FALSE)

logFC=1

P.Value = 0.05

k1 = (degdf$p_val < P.Value)&(degdf$avg_log2FC < -logFC)

k2 = (degdf$p_val < P.Value)&(degdf$avg_log2FC > logFC)

degdf$change = ifelse(k1,"down",ifelse(k2,"up","stable"))

table(degdf$change)

library(ggplot2)

library(ggrepel)

colnames(degdf)

data <- degdf

p <- ggplot(

# 数据、映射、颜色

data, aes(x = avg_log2FC, y = -log10(p_val), colour=change)) +

geom_point(alpha=0.6, size=2.5) +

scale_color_manual(values=c("#45b97c", "#d2dae2","#f15a22"))+

# 辅助线

geom_vline(xintercept=c(-1,1),lty=4,col="black",lwd=0.6,alpha=0.8) +

geom_hline(yintercept = -log10(0.05),lty=4,col="black",lwd=0.6,alpha=0.8) +

# 坐标轴

labs(x="log2(fold change)",

y="-log10 (p-value)")+

theme_bw()+

# 图例

theme(plot.title = element_text(hjust = 0.5),

legend.position="right",

legend.title = element_blank())

##BiocManager::install("AUCell")

library(AUCell)

library(ggplot2)

cells_rankings <- AUCell_buildRankings(scRNAlist[["all"]]@assays$RNA@data, nCores=6, plotStats=TRUE, splitByBlocks = TRUE)

cells_rankings

library(msigdbr)

msigdbr_show_species()

kk <- msigdbr_collections()

pathway <- msigdbr(species = "Mus musculus",

category = "H",

)

#

fgsea_sets<- pathway %>% split(x = .$gene_symbol, f = .$gs_name)

cells_AUC <- AUCell_calcAUC(fgsea_sets,

cells_rankings,

nCores =1,

aucMaxRank=nrow(cells_rankings)*0.1)

aucs <- as.numeric(getAUC(cells_AUC)[i, ])

scRNA1$AUC <- aucs

umap_mat <- Embeddings(scRNA1, reduction = "umap")

df<- data.frame(scRNA1@meta.data, umap_mat)

colnames(df)

class_avg <- df %>%

group_by( celltype) %>%#按照细胞类型分组

summarise(

UMAP_1 = median(UMAP_1),

UMAP_2 = median(UMAP_2)

)

class(df$UMAP_2)

p1 <- ggplot(df, aes(UMAP_1, UMAP_2)) +

geom_point(aes(colour = AUC)) +

viridis::scale_color_viridis(option="A") +#

ggrepel::geom_label_repel(aes(label = celltype),

data = class_avg,#

size = 5,

label.size = 1,

segment.color = NA

)+

theme(legend.position = "none") +

theme_bw()+

labs(title = i)

library(org.Hs.eg.db)

library(org.Mm.eg.db)

library(clusterProfiler)

colnames(degdf)

DEGs <- degdf %>%

filter(p_val < 0.05) %>%

filter(abs(avg_log2FC) >0.1)

degs.list=rownames(DEGs)

ego = enrichGO(gene =degs.list,#

OrgDb = org.Mm.eg.db,#

keyType = "SYMBOL",

ont = "all",

minGSSize = 1,

pvalueCutoff =0.05,

qvalueCutoff =0.1,

readable = TRUE)

ego_res <- ego@result

barplot(ego, showCategory = 30,color = "pvalue",label_format=100)#展示前二十个条目

y <- ego_res$Description[30:60]

barplot(ego, showCategory = y,color = "pvalue",label_format=100)#展示前二十个条目

dotplot(ego, showCategory = 30,color = "pvalue",label_format=100)

barplot(ego, drop = TRUE, showCategory =10,color = "pvalue",split="ONTOLOGY",label_format=100) +

facet_grid(ONTOLOGY~., scale='free')

dotplot(ego,showCategory = 10,split="ONTOLOGY",color = "pvalue",label_format=100) +

facet_grid(ONTOLOGY~., scale='free')

keytypes(org.Hs.eg.db)

DEG.entrez_id = mapIds(x = org.Mm.eg.db,

keys = degs.list,

keytype = "SYMBOL",

column = "ENTREZID")

erich.kegg.res <- enrichKEGG(gene = DEG.entrez_id,

organism = "mmu",

keyType = "kegg")

EGG <- enrichKEGG(gene = DEG.entrez_id,

organism = 'mmu',

pvalueCutoff = 0.1,

use_internal_data =F)

barplot(EGG, showCategory = 30,color = "pvalue",label_format=100)#展示前二十个条目

dotplot(EGG, showCategory = 30,label_format=100)

##################################################################

if (!requireNamespace("BiocManager", quietly = TRUE))

install.packages("BiocManager")

BiocManager::install("monocle")

library(Seurat)

load(file="../01_juleizhushi/output/Astrocyte/scRNA_Astrocyte.Rdata")

#

pbmc <- scRNA1

rm(scRNA1)

#

library(monocle)

expr_matrix <- as(as.matrix(pbmc@assays$RNA@counts))

p_data$celltype <- pbmc@active.ident

DefaultAssay(pbmc) <- "RNA"

f_data <- data.frame(gene_short_name = row.names(pbmc),

row.names = row.names(pbmc))

pd <- new('AnnotatedDataFrame',

data = p_data)

fd <- new('AnnotatedDataFrame',

data = f_data)

cds <- newCellDataSet(expr_matrix,

phenoData = pd,

featureData = fd,

lowerDetectionLimit = 0.5,

expressionFamily = negbinomial.size())

cds <- estimateSizeFactors(cds)

cds <- estimateDispersions(cds)

cds <- detectGenes(cds, min_expr = 0.1)

expressed_genes <- row.names(subset(fData(cds),

num_cells_expressed >= 10))

library(igraph)

express_genes <- VariableFeatures(pbmc)

cds <- setOrderingFilter(cds, express_genes)

plot_ordering_genes(cds)

deg.cluster <- FindAllMarkers(pbmc)

express_genes <- subset(deg.cluster,p_val_adj<0.05)$gene

cds <- setOrderingFilter(cds, express_genes)

disp_table <- dispersionTable(cds)

disp.genes <- subset(disp_table, mean_expression >= 0.1 & dispersion_empirical >= 1 * dispersion_fit)$gene_id

cds <- setOrderingFilter(cds, disp.genes)

plot_ordering_genes(cds)

#

colnames(pbmc@meta.data)

table(pbmc@meta.data$Patient)

diff <- differentialGeneTest(cds[expressed_genes,],

fullModelFormulaStr="~Patient",

cores=30)

deg <- subset(diff, qval < 0.01)

deg <- deg[order(deg$qval,decreasing=F),]

head(deg)

write.table(deg,file="train.monocle.DEG.xls",

col.names=T,

row.names=F,

sep="\t",

quote=F)

##

ordergene <- rownames(deg)

ordergene <- row.names(deg)[order(deg$qval)][1:2000]

cds <- setOrderingFilter(cds, ordergene)

colnames(pbmc@meta.data)

table(pbmc@meta.data$time)

cds <- reduceDimension(cds,

max_components = 2,

num_dim = 2,

method = 'DDRTree',

sigma = 0.5,

verbose = T)

cds <- orderCells(cds)

library(ggsci)

plot_cell_trajectory(cds,color_by="Pseudotime", size=1,show_backbone=TRUE) +

scale_color_gradient(low = "blue", high = "red")

#################################################################

library(Biobase)

library(genefilter)

library(limma)

library(RColorBrewer)

library(GSVA) #need R 3.6

library(pheatmap)

library(GSEABase)

geneSets <- getGmt('resource/genesets.v2023.2.Hs.gmt')

exp <- read.csv(file = "data/exp.csv",check.names = F)

colnames(exp)

grepl("count",colnames(exp))

exp <- exp[,!grepl("count",colnames(exp))]

exp[,2:26] <- apply(exp[,2:26],1,as.numeric)

library(tibble)

library(stringr)

library(dplyr)

colnames(exp) <- gsub("_FPKM","",colnames(exp))

range(exp)

exp <- log2(exp+1)

save(exp,file = "output/exp.Rdata")

GSVA_hall <- gsva(expr=as.matrix(exp),

gset.idx.list=geneSets, #

method="gsva", #

mx.diff=T,

kcdf="Gaussian",

parallel.sz=4,

min.sz=2)

GSVA <- as.data.frame(GSVA_hall)

library(limma)

library(stringr)

group <- trimws(str_split(colnames(GSVA),'-',simplify = T)[,1])

group <- factor(group,levels = c("S0","S3","S7","S14","S28"))

metadata <- data.frame(sample=colnames(exp),group=group)

library(ggsci)

library(tidyr)

library(ggpubr)

identical(rownames(a),metadata$sample)

a <- as.data.frame(t(GSVA))

a$group <- group

b <- gather(a,key=pathway,value = GSVA_score,-c(group))

ggboxplot(b, x = "pathway", y = "GSVA_score",

fill = "group", palette = "lancet")+

stat_compare_means(aes(group = group),

label = "p.signif",

symnum.args=list(cutpoints = c(0, 0.001, 0.01, 0.05, 1),

symbols = c("***", "**", "*", "ns")))+

theme(text = element_text(size=10),

axis.text.x = element_text(angle=45, hjust=1))

dev.off()

gene <- c("Lgals1","Vim","Fabp5","Nefl")

a <- as.data.frame(t(exp[gene,]))

a$group <- group

b <- gather(a,key=gene,value = expression,-c(group))

ggboxplot(b, x = "gene", y = "expression",

fill = "group", palette = "lancet")+

stat_compare_means(aes(group = group),

label = "p.signif",

symnum.args=list(cutpoints = c(0, 0.001, 0.01, 0.05, 1),

symbols = c("***", "**", "*", "ns")))+

theme(text = element_text(size=10),

axis.text.x = element_text(angle=45, hjust=1))

dev.off()

gene <- intersect(rownames(exp),geneSets[["HALLMARK_OXIDATIVE_PHOSPHORYLATION"]]@geneIds)

gene <- c("Lgals1","Vim","Fabp5","Nefl")

Fatty_gene <- read.csv(file = "data/Fatty acid metabolism.csv",header = F)

gene <- intersect(rownames(exp),Fatty_gene$V2)

Lactylase <- read.csv(file = "data/Lactylated proteins.csv",header = F)

gene <- intersect(rownames(exp),Lactylase$V2)

diff <- exp[gene,]

labels_gene <- c("Eno1","pdk3","Eno2","pgk1", "Ldhc","hk2", "pfkp", "pfkfb1","Gapdhs")

labels_gene <- intersect(labels_gene,rownames(diff))

#install.packages('pheatmap')

group

k <- c(which(group=="S0"),which(group=="S3"),which(group=="S7"),which(group=="S14"),which(group=="S28"))

set.seed(123)

library(ComplexHeatmap)

library(pheatmap)

annotation_col=data.frame(group=group[k])

rownames(annotation_col)=colnames(diff[k])

pheatmap(diff[,k],

annotation_col=annotation_col,

scale = "row",

border_color = "grey",

main = "Heatmap",#

cluster_rows=T,#

cluster_cols=F,#

show_rownames =F,#

show_colnames =T,#

color = colorRampPalette(c("navy", "white", "red"))(50),

fontsize = 10,

fontsize_row=8,

fontsize_col=8)

+

rowAnnotation(link = anno_mark(at = which(rownames(diff) %in% labels_gene),

labels = labels_gene, labels_gp = gpar(fontsize = 10)))

dev.off()

library("KEGGREST")

library("EnrichmentBrowser")

hsapathway <- downloadPathways("mmu") #

### retrieve gene sets

hsa <- getGenesets(org = "mmu", db = "kegg", gene.id.type = "SYMBOL",cache = TRUE, return.type="list") ##只有在第一次运行这句代码时，耗时较长

writeGMT(hsa, gmt.file = "20230113_kegg_mmu.gmt")

################################################################

load(file = "..//output/exp.Rdata")

load(file = "..//output/metadata.Rdata")

exp <- na.omit(exp)

library(e1071)

library(parallel)

library(preprocessCore)

source("resource/CIBERSORT.R")

#

sig_matrix <- "resource/ImmuCC.txt"

exprSet <- tibble::rownames_to_column(exp)

mixture_file <- 'output/exp.txt'

res_cibersort <- CIBERSORT(sig_matrix, mixture_file, perm=1000, QN=TRUE)#perm值越高，计算时间越久，结果越稳定

res_cibersort <- res_cibersort[,1:22]

ciber.res <- res_cibersort[,colSums(res_cibersort) > 0]

group <- metadata$group

library(pheatmap)

re2 <- as.data.frame(t(ciber.res))

identical(colnames(re2),colnames(exp))

an = data.frame(group = group,

row.names =colnames(exp))

pheatmap(re2,

scale = "row",

show_colnames = T, #

show_rownames = T,

cluster_rows = TRUE,

cluster_cols = F, #

annotation_col = an,

color = colorRampPalette(c("navy", "white", "firebrick3"))(50))

library(RColorBrewer)

library(dplyr)

library(tidyr)

library(ggplot2)

library(tibble)

dd1 <- res_cibersort %>%

as.data.frame() %>%

rownames_to_column("sample") %>%

mutate(group) %>%

pivot_longer(cols=2:23,

names_to= "celltype",

values_to = "Proportion")

library(ggplot2)

ggplot(dd1,aes(sample,Proportion,fill = celltype)) +

geom_bar(position = "fill",stat = "identity")+

theme_bw()+

guides(fill=guide_legend(ncol=1))+

facet_wrap(~group, scales = "free_x", nrow = 1)+

theme(axis.text.x = element_text(angle = 45, hjust = 1,vjust = 1, colour = "black"))

library(tibble)

library(ggpubr)

re <-ciber.res

dat <- re %>% as.data.frame() %>%

rownames_to_column("sample") %>%

gather(key = Cell_type,value = Proportion,-sample)

dat <- inner_join(dat,metadata,by="sample")

ggboxplot(dat, x = "Cell_type", y = "Proportion",

fill = "group", palette = "lancet")+

stat_compare_means(aes(group = group),

#method = "wilcox.test",

label = "p.signif",

symnum.args=list(cutpoints = c(0, 0.001, 0.01, 0.05, 1),

symbols = c("***", "**", "*", " ")))+

theme(text = element_text(size=10),

axis.text.x = element_text(angle=45, hjust=1))

#################################################################

library(corrplot)

library(tidyverse)

load(file = "../output/res_cibersort.Rdata")

res_cibersort <- res_cibersort[,1:22]

ciber.res <- res_cibersort[,colSums(res_cibersort) > 0]

b <- as.data.frame(ciber.res)

cor<-sapply(b,function(x,y) cor(x,y,method="spearman"),b)

rownames(cor)<-colnames(b)

write.csv(cor,file = "output/cor.csv")

library(psych)

cortest_psy <- corr.test(b,b,method = "spearman")

p <- cortest_psy[["p"]]

write.csv(p,file = "output/p.csv")

#

corrplot(cor,

method = "color",

col=colorRampPalette(c("#01468b","white","#ee0000"))(100),#颜色

addCoef.col = "black",

tl.col="black",

type = "full",

number.cex = 0.01,

pch.cex = 1,

tl.cex = 0.8,#

cl.align = "l",

p.mat = p,

insig = "label_sig",sig.level = c(.001, .01, .05))#

##################################################################

####

library(corrplot)

library(tidyverse)

load(file = "../output/exp.Rdata")

gene <- c("Lgals1","Vim","Fabp5","Nefl")

data <- as.data.frame(t(exp[gene,]))

data[,1:4] <- apply(data[,1:4],1,as.numeric)

load(file = "../05_CIBERSORT分析(人和小鼠）/output/res_cibersort.Rdata")

res_cibersort <- res_cibersort[,1:22]

ciber.res <- as.data.frame(res_cibersort[,colSums(res_cibersort) > 0] ) #去除丰度全为0的细胞

identical(rownames(data),rownames(ciber.res))

cor<- as.data.frame(cor(ciber.res,data[,i]))

colnames(cor) <- "cor"

library(psych)

cortest_psy <- corr.test(ciber.res,data[,i],method = "spearman")

p <- as.data.frame( cortest_psy[["p"]])

identical(rownames(cor),rownames(p))

cor$pvalue <- p$V1

write.csv(cor,file = paste0("output/",i,"_cor.csv"))

library(ggplot2)

dfm <- cor

dfm$pvalue <- ifelse((dfm$pvalue < 0.05)&(dfm$pvalue >= 0.01),"*",

ifelse((dfm$pvalue < 0.01)&(dfm$pvalue >= 0.001),"**",

ifelse((dfm$pvalue < 0.001),"***","ns")))

dfm$pvalue <- as.factor(dfm$pvalue)

dfm$Cor <- abs(dfm$cor)

dfm$name <- factor(rownames(dfm))

dfm <- dfm[order(dfm$cor), ]

dfm$name <- factor(dfm$name, levels = rownames(dfm))

g1 <- ggplot(dfm, aes(x=cor, y=name, color = pvalue)) +

geom_segment(aes(y = name, #

x = 0,

yend = name,

xend = cor),

color = "gray", size=1) +

geom_point(stat='identity',aes(size= Cor)) +

labs(x="cor",y="Cell Type")+

theme_bw()

g1 <- g1 + ggtitle(i) +

theme(plot.title = element_text(hjust = 0.5))

###

library(ggpubr)

colnames(data)

library(ggstatsplot)#

ggscatterstats(data,

y =CD73.FAP.vs.FAP, #

x =CD8.vs.TOTAL,#

# ylab = i,

type = "pearson",

centrality.para = "mean",

margins = "both",

marginal.type = "density",

title = "COR")

###############################################################

load(file = "..//output/exp.Rdata")

colnames(exp)

library(stringr)

group <- trimws(str_split(colnames(exp),'-',simplify = T)[,1])

group <- factor(group,levels = c("S0","S3","S7","S14","S28"))

library(limma)

boxplot(exp,outline=FALSE, notch=T,col=group, las=2)

dev.off()

exp=normalizeBetweenArrays(exp)

boxplot(exp,outline=FALSE, notch=T,col=group, las=2)

exp <- as.data.frame(exp)

range(exp)

dev.off()

project <- c("S3","S7","S14","S28")

colnames(exp)

table(group)

exp_01 <- exp[,c(grepl("S0",colnames(exp))|grepl(i,colnames(exp)))]

colnames(exp_01)

group <- trimws(str_split(colnames(exp_01),'-',simplify = T)[,1])

group <- factor(group,levels = c("S0",i))

library(tidyverse)

library(GEOquery)

library(limma)

design=model.matrix(~group)

fit=lmFit(exp_01,design)

fit=eBayes(fit)

deg=topTable(fit,coef=2,number = Inf)

logFC=1

P.Value = 0.05

k1 = (deg$P.Value < P.Value)&(deg$logFC < -logFC)

k2 = (deg$P.Value < P.Value)&(deg$logFC > logFC)

deg$change = ifelse(k1,"down",ifelse(k2,"up","stable"))

table(deg$change)

save(deg,file = paste0("output/",i,"_vs_S0_deg.Rdata"))

write.csv(deg,file = paste0("output/",i,"_vs_S0_deg.csv"))

cg = rownames(deg)[deg$change !="stable"]

diff=exp_01[cg,]

k <- c(which(group=="S0"),which(group==i))

diff <- diff[,k]

#install.packages('pheatmap')

library(pheatmap)

annotation_col=data.frame(group=group[k])

rownames(annotation_col)=colnames(diff)

pdf(width = 8,height = 6,file = paste0("output/",i,"_vs_S0差异分析热图.pdf"))

p <- pheatmap(diff,

annotation_col=annotation_col,

scale = "row",#

main = "Heatmap",#

cluster_rows=T,

cluster_cols=F,

show_rownames = F,

show_colnames =T,

color = colorRampPalette(c("navy", "white", "red"))(50),

fontsize = 10,

fontsize_row=8,

fontsize_col=8)

print(p)

dev.off()

library(ggplot2)

library(ggrepel)

data <- deg

data$gene <- rownames(data)#

p <- ggplot(

data, aes(x = logFC, y = -log10(P.Value), colour=change)) +

geom_vline(xintercept=c(-1,1),lty=4,col="black",lwd=0.6,alpha=0.8) +

geom_hline(yintercept = -log10(0.05),lty=4,col="black",lwd=0.6,alpha=0.8) +

# 坐标轴

labs(x="log2(fold change)",

y="-log10 (p-value)")+

theme_bw()+

# 图例

theme(plot.title = element_text(hjust = 0.5),

legend.position="right",

legend.title = element_blank())#+
