# Supplementary material for: Integrating Bulk RNA and Single‐Cell RNA Sequencing Identifies and Validates Lactylation‐Related Signatures for Intervertebral Disc Degeneration
Source: J Cell Mol Med. 2024 Dec 5;28(23):e70262. doi: 10.1111/jcmm.70262 (PMC11619158; doi:10.1111/jcmm.70262)
Supplement: Supplementary file 4 — Table S3. The detailed list of GO and KEGG analysis in normal and degenerated human IVD tissue. [file JCMM-28-e70262-s002.pdf]

GO enrichment analysis of downregulated DEGs

	ONTOLOG	ID	Descriptior	GeneRatio	BgRatio	pvalue	p.adjust	qvalue	geneID	Count
GO:001612	BP	GO:001612	sterol biosy	16/1035	63/18614	2.30E-07	0.0006	0.000575	CYB5R2/EE	16
GO:000669	BP	GO:000669	cholesterol	15/1035	56/18614	2.57E-07	0.0006	0.000575	EBP/CYB5F	15
GO:190265	BP	GO:190265	secondary	15/1035	56/18614	2.57E-07	0.0006	0.000575	EBP/CYB5F	15
GO:004648	BP	GO:004648	glycerolipic	44/1035	385/18614	4.71E-06	0.006705	0.006427	SLC44A4/II	44
GO:190161	BP	GO:190161	organic hy	32/1035	242/18614	4.78E-06	0.006705	0.006427	CYB5R2/EE	32
GO:001921	BP	GO:001921	regulation	18/1035	99/18614	7.75E-06	0.009067	0.008692	ACADL/FM	18
GO:004616	BP	GO:004616	alcohol bic	22/1035	140/18614	9.48E-06	0.009509	0.009116	EBP/CYB5F	22
GO:000669	BP	GO:000669	steroid bio	25/1035	175/18614	1.35E-05	0.011119	0.010658	CYB5R2/EE	25
GO:000865	BP	GO:000865	phospholip	32/1035	255/18614	1.43E-05	0.011119	0.010658	SLC44A4/II	32
GO:000665	BP	GO:000665	glycerophc	35/1035	297/18614	2.27E-05	0.015601	0.014956	SLC44A4/II	35
GO:004501	BP	GO:004501	glycerolipic	31/1035	250/18614	2.45E-05	0.015601	0.014956	SLC44A4/II	31
GO:000664	BP	GO:000664	phospholip	41/1035	375/18614	2.82E-05	0.0165	0.015816	SLC44A4/II	41
GO:003502	BP	GO:003502	regulation	7/1035	21/18614	9.40E-05	0.045157	0.043288	ARHGAP17	7
GO:000820	BP	GO:000820	cholesterol	20/1035	140/18614	9.52E-05	0.045157	0.043288	EBP/CYB5F	20
GO:001660	BP	GO:001660	Rac proteir	10/1035	43/18614	9.65E-05	0.045157	0.043288	ARHGAP17	10
GO:004647	BP	GO:004647	glycerophc	26/1035	210/18614	0.00011	0.048391	0.046387	SLC44A4/II	26
GO:001612	BP	GO:001612	sterol meta	21/1035	155/18614	0.000139	0.052698	0.050516	CYB5R2/EE	21
GO:001605	BP	GO:001605	organic aci	29/1035	250/18614	0.000146	0.052698	0.050516	GSTZ1/ASF	29
GO:004639	BP	GO:004639	carboxylic	29/1035	250/18614	0.000146	0.052698	0.050516	GSTZ1/ASF	29
GO:000716	BP	GO:000716	establishm	27/1035	226/18614	0.00015	0.052698	0.050516	KANK1/MS	27
GO:000606	BP	GO:000606	alcohol me	38/1035	366/18614	0.000162	0.054232	0.051987	INPP1/EBP	38
GO:190002	BP	GO:190002	regulation	8/1035	30/18614	0.000174	0.055401	0.053107	COBL/KAN	8
GO:001921	BP	GO:001921	regulation	36/1035	344/18614	0.000204	0.062019	0.059452	PRKD1/MT	36
GO:001611	BP	GO:001611	terpenoid I	6/1035	17/18614	0.000212	0.062019	0.059452	HMGCS1/F	6
GO:002153	BP	GO:002153	neural tube	9/1035	39/18614	0.000229	0.063668	0.061032	TCTN1/PA	9
GO:003252	BP	GO:003252	microvillus	7/1035	24/18614	0.000242	0.063668	0.061032	TNIK/PLD1	7
GO:190265	BP	GO:190265	secondary	20/1035	150/18614	0.000247	0.063668	0.061032	EBP/CYB5F	20
GO:005081	BP	GO:005081	regulation	13/1035	76/18614	0.000262	0.063668	0.061032	NR1D1/FG	13
GO:000726	BP	GO:000726	small GTPa	46/1035	483/18614	0.000266	0.063668	0.061032	TIAM2/ARI	46
GO:000820	BP	GO:000820	steroid me	34/1035	323/18614	0.000272	0.063668	0.061032	CYB5R2/CI	34
GO:005105	BP	GO:005105	regulation	32/1035	298/18614	0.000285	0.063719	0.061081	TIAM2/ARI	32
GO:009706	BP	GO:009706	anterior he	4/1035	7/18614	0.00029	0.063719	0.061081	PFAS/SOX9	4
GO:190372	BP	GO:190372	regulation	6/1035	18/18614	0.000303	0.064487	0.061817	ASAP1/SYT	6
GO:003281	BP	GO:003281	positive re	7/1035	26/18614	0.000417	0.085983	0.082423	LAMP1/AX	7
GO:009717	BP	GO:009717	ruffle asser	9/1035	43/18614	0.000499	0.098366	0.094293	COBL/KAN	9
GO:004689	BP	GO:004689	regulation	22/1035	182/18614	0.000505	0.098366	0.094293	ACADL/NR	22
GO:005165	BP	GO:005165	establishm	42/1035	445/18614	0.00058	0.110001	0.105448	VAMP8/PII	42
GO:009003	BP	GO:009003	negative re	3/1035	4/18614	0.000657	0.118286	0.113389	GPD1L/DG	3
GO:200074	BP	GO:200074	positive re	3/1035	4/18614	0.000657	0.118286	0.113389	SOX9/SOX	3
GO:004802	BP	GO:004802	regulation	15/1035	108/18614	0.000925	0.158857	0.152281	FAM172A/	15
GO:000038	BP	GO:000038	regulation	10/1035	56/18614	0.00093	0.158857	0.152281	FAM172A/	10
GO:200064	BP	GO:200064	positive re	4/1035	9/18614	0.000956	0.158857	0.152281	MTMR2/M	4
GO:004428	BP	GO:004428	small mole	37/1035	388/18614	0.000995	0.158857	0.152281	GSTZ1/ASF	37
GO:005087	BP	GO:005087	regulation	35/1035	361/18614	0.001008	0.158857	0.152281	SERPINA1/	35
GO:003214	BP	GO:003214	activation	15/1035	109/18614	0.001018	0.158857	0.152281	ADRB2/JAI	15
GO:000829	BP	GO:000829	isoprenoid	7/1035	30/18614	0.001061	0.161884	0.155182	HMGCS1/F	7
GO:004000	BP	GO:004000	establishm	8/1035	39/18614	0.00117	0.174738	0.167504	NSFL1C/D	8
GO:004348	BP	GO:004348	regulation	21/1035	182/18614	0.001224	0.178917	0.17151	FAM172A/	21
GO:005108	BP	GO:005108	nuclear me	4/1035	10/18614	0.001523	0.214225	0.205356	VRK1/DCT	4
GO:009758	BP	GO:009758	lamellipodi	13/1035	91/18614	0.001526	0.214225	0.205356	KANK1/SP	13
GO:190475	BP	GO:190475	positive re	3/1035	5/18614	0.001575	0.214861	0.205966	PINX1/NM	3
GO:000650	BP	GO:000650	GPI anchor	7/1035	32/18614	0.001592	0.214861	0.205966	PIGT/PIGK	7
GO:006201	BP	GO:006201	regulation	32/1035	331/18614	0.001699	0.224979	0.215665	LCMT1/AC	32
GO:002191	BP	GO:002191	neural tube	19/1035	163/18614	0.001816	0.228981	0.219501	COBL/TCTI	19
GO:005068	BP	GO:005068	regulation	17/1035	139/18614	0.001845	0.228981	0.219501	FAM172A/	17
GO:190257	BP	GO:190257	protein loc	5/1035	17/18614	0.001857	0.228981	0.219501	PINX1/RRS	5
GO:190160	BP	GO:190160	alpha-ami	13/1035	93/18614	0.001865	0.228981	0.219501	GSTZ1/ASF	13
GO:001969	BP	GO:001969	ribose pho	42/1035	473/18614	0.001903	0.228981	0.219501	PPT1/JMJD	42
GO:000650	BP	GO:000650	GPI anchor	7/1035	33/18614	0.001925	0.228981	0.219501	PIGT/PIGK	7
GO:000038	BP	GO:000038	alternative	11/1035	72/18614	0.001991	0.232899	0.223258	FAM172A/	11
GO:002261	BP	GO:002261	ribonucleo	42/1035	475/18614	0.002058	0.236804	0.227001	RCL1/NIFK	42
GO:003281	BP	GO:003281	regulation	8/1035	43/18614	0.002266	0.238802	0.228916	LAMP1/AX	8
GO:003039	BP	GO:003039	membrane	4/1035	11/18614	0.002288	0.238802	0.228916	VRK1/DCT	4
GO:003350	BP	GO:003350	floor plate	4/1035	11/18614	0.002288	0.238802	0.228916	COBL/CBY	4
GO:004648	BP	GO:004648	phosphatic	18/1035	154/18614	0.002293	0.238802	0.228916	INPP1/ITPK	18
GO:000013	BP	GO:000013	establishm	7/1035	34/18614	0.002309	0.238802	0.228916	NSFL1C/D	7
GO:001593	BP	GO:001593	coenzyme	5/1035	18/18614	0.002455	0.238802	0.228916	ACOT7/AC	5
GO:007139	BP	GO:007139	cellular res	5/1035	18/18614	0.002455	0.238802	0.228916	CKB/AR/TF	5
GO:000672	BP	GO:000672	isoprenoid	15/1035	119/18614	0.002481	0.238802	0.228916	HMGCS1/F	15
GO:190440	BP	GO:190440	positive re	8/1035	44/18614	0.002636	0.238802	0.228916	JAK2/ASL/I	8
GO:008016	BP	GO:008016	regulation	10/1035	65/18614	0.002979	0.238802	0.228916	JAK2/ASL/I	10
GO:000091	BP	GO:000091	assembly c	3/1035	6/18614	0.00302	0.238802	0.228916	PLEC/PDCI	3
GO:000091	BP	GO:000091	actomyosii	3/1035	6/18614	0.00302	0.238802	0.228916	PLEC/PDCI	3
GO:000601	BP	GO:000601	5-phospho	3/1035	6/18614	0.00302	0.238802	0.228916	PYGL/PRPS	3
GO:004533	BP	GO:004533	farnesyl di	3/1035	6/18614	0.00302	0.238802	0.228916	HMGCS1/F	3
GO:004639	BP	GO:004639	5-phospho	3/1035	6/18614	0.00302	0.238802	0.228916	PYGL/PRPS	3
GO:190162	BP	GO:190162	negative re	3/1035	6/18614	0.00302	0.238802	0.228916	GPR161/SU	3
GO:190355	BP	GO:190355	regulation	3/1035	6/18614	0.00302	0.238802	0.228916	SDC4/PDC	3
GO:000701	BP	GO:000701	actin filam	39/1035	442/18614	0.003022	0.238802	0.228916	COBL/ARH	39
GO:000754	BP	GO:000754	primary se	2/1035	2/18614	0.003089	0.238802	0.228916	MAP3K4/S	2
GO:000835	BP	GO:000835	olfactory le	2/1035	2/18614	0.003089	0.238802	0.228916	TPBG/ADC	2
GO:001899	BP	GO:001899	germ-line	2/1035	2/18614	0.003089	0.238802	0.228916	MAP3K4/S	2
GO:001910	BP	GO:001910	male germ	2/1035	2/18614	0.003089	0.238802	0.228916	MAP3K4/S	2
GO:003102	BP	GO:003102	nuclear mi	2/1035	2/18614	0.003089	0.238802	0.228916	SUN2/SYN	2
GO:190014	BP	GO:190014	regulation	2/1035	2/18614	0.003089	0.238802	0.228916	GAS6/AKA	2
GO:190363	BP	GO:190363	regulation	2/1035	2/18614	0.003089	0.238802	0.228916	RGN/AIMP	2
GO:190456	BP	GO:190456	response t	2/1035	2/18614	0.003089	0.238802	0.228916	CKB/SP1	2
GO:190456	BP	GO:190456	cellular res	2/1035	2/18614	0.003089	0.238802	0.228916	CKB/SP1	2
GO:200039	BP	GO:200039	regulation	2/1035	2/18614	0.003089	0.238802	0.228916	VPS28/TSG	2
GO:200039	BP	GO:200039	positive re	2/1035	2/18614	0.003089	0.238802	0.228916	VPS28/TSG	2
GO:003152	BP	GO:003152	ruffle orga	9/1035	55/18614	0.003096	0.238802	0.228916	COBL/KAN	9
GO:009003	BP	GO:009003	regulation	5/1035	19/18614	0.003181	0.242699	0.232651	GPD1L/DG	5
GO:000672	BP	GO:000672	terpenoid	13/1035	99/18614	0.003275	0.245109	0.234962	HMGCS1/F	13
GO:003511	BP	GO:003511	genitalia m	4/1035	12/18614	0.003283	0.245109	0.234962	AR/RBP4/T	4
GO:000679	BP	GO:000679	sulfur comp	30/1035	319/18614	0.003507	0.259137	0.248409	HYAL1/PPT	30
GO:003444	BP	GO:003444	substrate a	13/1035	100/18614	0.003577	0.261545	0.250717	ST6GAL1/K	13
GO:004206	BP	GO:004206	gliogenesis	30/1035	320/18614	0.003671	0.265628	0.254632	APCDD1/P	30
GO:000906	BP	GO:000906	cellular am	14/1035	113/18614	0.003985	0.285429	0.273613	GSTZ1/ASF	14
GO:000925	BP	GO:000925	ribonucleo	40/1035	464/18614	0.004034	0.285991	0.274151	PPT1/JMJD	40
GO:000333	BP	GO:000333	keratinocyt	4/1035	13/18614	0.004535	0.311493	0.298597	PALLD/SFN	4
GO:000673	BP	GO:000673	NADH met	4/1035	13/18614	0.004535	0.311493	0.298597	GPD1L/OG	4
GO:003253	BP	GO:003253	regulation	4/1035	13/18614	0.004535	0.311493	0.298597	PLD1/EZR/	4
GO:000046	BP	GO:000046	cleavage ir	6/1035	29/18614	0.004593	0.311493	0.298597	RCL1/RRS1	6
GO:003086	BP	GO:003086	cortical cyt	8/1035	48/18614	0.004615	0.311493	0.298597	EZR/PAFAI	8
GO:000759	BP	GO:000759	hemostasis	23/1035	230/18614	0.004737	0.312494	0.299558	SERPINA1/	23
GO:000663	BP	GO:000663	acylglycerc	15/1035	128/18614	0.004991	0.312494	0.299558	DGKG/AVI	15
GO:000865	BP	GO:000865	pyrimidine	3/1035	7/18614	0.005067	0.312494	0.299558	CDA/TK2/U	3
GO:001619	BP	GO:001619	axon choic	3/1035	7/18614	0.005067	0.312494	0.299558	ROBO3/RC	3
GO:003253	BP	GO:003253	regulation	3/1035	7/18614	0.005067	0.312494	0.299558	EZR/PLS1/	3
GO:003280	BP	GO:003280	lacrimal gla	3/1035	7/18614	0.005067	0.312494	0.299558	FOXC1/SO	3
GO:004483	BP	GO:004483	actomyosii	3/1035	7/18614	0.005067	0.312494	0.299558	PLEC/PDCI	3
GO:007197	BP	GO:007197	extracellula	3/1035	7/18614	0.005067	0.312494	0.299558	SDC4/PDC	3
GO:190568	BP	GO:190568	regulation	3/1035	7/18614	0.005067	0.312494	0.299558	SYTL4/ANX	3
GO:200073	BP	GO:200073	positive re	5/1035	21/18614	0.005075	0.312494	0.299558	FOXC1/PW	5
GO:003235	BP	GO:003235	regulation	7/1035	39/18614	0.005186	0.316556	0.303451	PAX8/NR3	7
GO:000663	BP	GO:000663	neutral lipi	15/1035	129/18614	0.005366	0.324684	0.311243	DGKG/AVI	15
GO:190331	BP	GO:190331	regulation	30/1035	329/18614	0.005456	0.327316	0.313765	FAM172A/	30
GO:003001	BP	GO:003001	establishm	17/1035	155/18614	0.005737	0.339088	0.32505	KANK1/MS	17
GO:000915	BP	GO:000915	purine ribo	38/1035	445/18614	0.005806	0.339088	0.32505	PPT1/JMJD	38
GO:003158	BP	GO:003158	cell-substr	32/1035	359/18614	0.005868	0.339088	0.32505	ST6GAL1/S	32
GO:001607	BP	GO:001607	tRNA catal	4/1035	14/18614	0.006072	0.339088	0.32505	EXOSC10/I	4
GO:001820	BP	GO:001820	peptidyl-m	4/1035	14/18614	0.006072	0.339088	0.32505	NAA30/ME	4
GO:003023	BP	GO:003023	male sex d	4/1035	14/18614	0.006072	0.339088	0.32505	MAP3K4/A	4
GO:190365	BP	GO:190365	positive re	4/1035	14/18614	0.006072	0.339088	0.32505	MTMR2/M	4
GO:004620	BP	GO:004620	nitric oxide	11/1035	83/18614	0.006116	0.339088	0.32505	CYB5R3/JA	11
GO:012003	BP	GO:012003	regulation	20/1035	195/18614	0.006148	0.339088	0.32505	COBL/KAN	20
GO:000666	BP	GO:000666	phosphatic	15/1035	131/18614	0.006184	0.339088	0.32505	INPP1/ITPK	15
GO:007232	BP	GO:007232	monocarbo	15/1035	131/18614	0.006184	0.339088	0.32505	ACADL/DC	15
GO:200105	BP	GO:200105	reactive nit	11/1035	84/18614	0.006694	0.364206	0.349128	CYB5R3/JA	11
GO:006049	BP	GO:006049	regulation	20/1035	197/18614	0.006868	0.366191	0.351031	COBL/KAN	20
GO:005129	BP	GO:005129	establishm	7/1035	41/18614	0.006886	0.366191	0.351031	NSFL1C/D	7
GO:003003	BP	GO:003003	lamellipodi	10/1035	73/18614	0.006941	0.366191	0.351031	SPATA13/	10
GO:000759	BP	GO:000759	blood coag	22/1035	224/18614	0.006963	0.366191	0.351031	SERPINA1/	22
GO:004542	BP	GO:004542	regulation	9/1035	62/18614	0.006991	0.366191	0.351031	JAK2/ASL/I	9
GO:200067	BP	GO:200067	regulation	8/1035	52/18614	0.007573	0.366692	0.351511	FOXC1/SRI	8
GO:000665	BP	GO:000665	phosphatic	6/1035	32/18614	0.007605	0.366692	0.351511	PLD1/DGK	6
GO:002190	BP	GO:002190	dorsal/ven	5/1035	23/18614	0.007654	0.366692	0.351511	TCTN1/GP	5
GO:003809	BP	GO:003809	Fc-epsilon	5/1035	23/18614	0.007654	0.366692	0.351511	PLCG2/VA	5
GO:003369	BP	GO:003369	cellular pol	9/1035	63/18614	0.007764	0.366692	0.351511	UGP2/HAS	9
GO:004396	BP	GO:004396	histone H4	9/1035	63/18614	0.007764	0.366692	0.351511	MUC1/RU	9
GO:001719	BP	GO:001719	N-termina	3/1035	8/18614	0.007773	0.366692	0.351511	NAA30/NA	3
GO:200073	BP	GO:200073	regulation	3/1035	8/18614	0.007773	0.366692	0.351511	SOX9/SOX	3
GO:190382	BP	GO:190382	positive re	39/1035	468/18614	0.007774	0.366692	0.351511	ARHGEF5/	39
GO:000912	BP	GO:000912	nucleoside	7/1035	42/18614	0.007876	0.366692	0.351511	PAICS/PFA	7
GO:004542	BP	GO:004542	positive re	7/1035	42/18614	0.007876	0.366692	0.351511	JAK2/ASL/I	7
GO:004650	BP	GO:004650	S-adenosy	4/1035	15/18614	0.00792	0.366692	0.351511	METTL16/I	4
GO:005101	BP	GO:005101	actin filam	17/1035	161/18614	0.008321	0.366692	0.351511	ARHGEF5/I	17
GO:003253	BP	GO:003253	regulation	31/1035	354/18614	0.008469	0.366692	0.351511	KANK1/DA	31
GO:000336	BP	GO:000336	establishm	2/1035	3/18614	0.008924	0.366692	0.351511	AMOT/AM	2
GO:000666	BP	GO:000666	sphinganin	2/1035	3/18614	0.008924	0.366692	0.351511	SPTLC2/DE	2
GO:000753	BP	GO:000753	primary se	2/1035	3/18614	0.008924	0.366692	0.351511	MAP3K4/S	2
GO:001405	BP	GO:001405	negative re	2/1035	3/18614	0.008924	0.366692	0.351511	ABAT/GAB	2
GO:002181	BP	GO:002181	nucleokine	2/1035	3/18614	0.008924	0.366692	0.351511	SUN2/SYN	2
GO:003129	BP	GO:003129	Ran proteir	2/1035	3/18614	0.008924	0.366692	0.351511	GPSM2/RA	2
GO:003348	BP	GO:003348	nitric oxide	2/1035	3/18614	0.008924	0.366692	0.351511	GSTP1/ADI	2
GO:004519	BP	GO:004519	establishm	2/1035	3/18614	0.008924	0.366692	0.351511	FGF13/DO	2
GO:004520	BP	GO:004520	establishm	2/1035	3/18614	0.008924	0.366692	0.351511	FGF13/DO	2
GO:004520	BP	GO:004520	MAPK expo	2/1035	3/18614	0.008924	0.366692	0.351511	DUSP16/D	2
GO:004606	BP	GO:004606	dGDP meta	2/1035	3/18614	0.008924	0.366692	0.351511	NUDT18/G	2
GO:007085	BP	GO:007085	positive re	2/1035	3/18614	0.008924	0.366692	0.351511	NR1D1/ST	2
GO:007162	BP	GO:007162	masticatior	2/1035	3/18614	0.008924	0.366692	0.351511	TIFAB/DCA	2
GO:009858	BP	GO:009858	learned vo	2/1035	3/18614	0.008924	0.366692	0.351511	TIFAB/DCA	2
GO:190289	BP	GO:190289	terminal w	2/1035	3/18614	0.008924	0.366692	0.351511	EZR/PLS1	2
GO:190377	BP	GO:190377	positive re	2/1035	3/18614	0.008924	0.366692	0.351511	VPS37B/TS	2
GO:190425	BP	GO:190425	positive re	2/1035	3/18614	0.008924	0.366692	0.351511	NR1D1/ST	2
GO:190495	BP	GO:190495	positive re	2/1035	3/18614	0.008924	0.366692	0.351511	CSNK1D/D	2
GO:190574	BP	GO:190574	negative re	2/1035	3/18614	0.008924	0.366692	0.351511	TIFAB/DCA	2
GO:190574	BP	GO:190574	hard palate	2/1035	3/18614	0.008924	0.366692	0.351511	TIFAB/DCA	2
GO:200064	BP	GO:200064	negative re	2/1035	3/18614	0.008924	0.366692	0.351511	MTMR2/AI	2
GO:200085	BP	GO:200085	regulation	2/1035	3/18614	0.008924	0.366692	0.351511	POMC/TSF	2
GO:005081	BP	GO:005081	coagulatio	22/1035	229/18614	0.008934	0.366692	0.351511	SERPINA1/	22
GO:000912	BP	GO:000912	nucleoside	10/1035	76/18614	0.009188	0.374639	0.35913	AK7/PAICS	10
GO:000709	BP	GO:000709	nuclear mi	5/1035	24/18614	0.009234	0.374639	0.35913	DCTN1/PA	5
GO:004885	BP	GO:004885	neural nucl	9/1035	65/18614	0.009505	0.383411	0.367538	CKB/SYNG	9
GO:003162	BP	GO:003162	receptor in	14/1035	125/18614	0.00966	0.387447	0.371408	MTMR2/PL	14
GO:000836	BP	GO:000836	regulation	15/1035	138/18614	0.009865	0.393426	0.377138	ARHGAP18	15
GO:000680	BP	GO:000680	nitric oxide	10/1035	77/18614	0.010048	0.39615	0.37975	CYB5R3/JA	10
GO:000318	BP	GO:000318	heart valve	4/1035	16/18614	0.010103	0.39615	0.37975	SOX9/EFN	4
GO:003003	BP	GO:003003	microvillus	4/1035	16/18614	0.010103	0.39615	0.37975	TNIK/PLD1	4
GO:000915	BP	GO:000915	ribonucleo	6/1035	34/18614	0.010272	0.400553	0.38397	PAICS/PFA	6
GO:003465	BP	GO:003465	nucleobase	37/1035	447/18614	0.010342	0.401066	0.384462	RBM10/RB	37
GO:006157	BP	GO:006157	actin filam	17/1035	165/18614	0.01051	0.405345	0.388564	ARHGEF5/I	17
GO:190160	BP	GO:190160	alpha-ami	20/1035	206/18614	0.011011	0.406666	0.389831	GSTZ1/ASF	20
GO:000046	BP	GO:000046	maturation	5/1035	25/18614	0.011025	0.406666	0.389831	RCL1/RRS1	5
GO:000821	BP	GO:000821	glucocortic	5/1035	25/18614	0.011025	0.406666	0.389831	NR3C1/YW	5
GO:003157	BP	GO:003157	membrane	5/1035	25/18614	0.011025	0.406666	0.389831	PPT1/ANX	5
GO:003530	BP	GO:003530	regulation	14/1035	127/18614	0.011034	0.406666	0.389831	JAK2/MTM	14
GO:000045	BP	GO:000045	exonucleol	3/1035	9/18614	0.011182	0.406666	0.389831	EXOSC10/I	3
GO:000046	BP	GO:000046	exonucleol	3/1035	9/18614	0.011182	0.406666	0.389831	EXOSC10/I	3
GO:001587	BP	GO:001587	choline tra	3/1035	9/18614	0.011182	0.406666	0.389831	SLC44A4/S	3
GO:190296	BP	GO:190296	regulation	3/1035	9/18614	0.011182	0.406666	0.389831	MSN/RDX/	3
GO:190296	BP	GO:190296	positive re	3/1035	9/18614	0.011182	0.406666	0.389831	MSN/RDX/	3
GO:190474	BP	GO:190474	regulation	3/1035	9/18614	0.011182	0.406666	0.389831	PINX1/NM	3
GO:000597	BP	GO:000597	glycogen b	7/1035	45/18614	0.011474	0.40922	0.392279	UGP2/NR1	7
GO:000925	BP	GO:000925	glucan bios	7/1035	45/18614	0.011474	0.40922	0.392279	UGP2/NR1	7
GO:190230	BP	GO:190230	regulation	9/1035	67/18614	0.011524	0.40922	0.392279	GPD1L/AN	9
GO:005129	BP	GO:005129	establishm	8/1035	56/18614	0.01177	0.40922	0.392279	NSFL1C/D	8
GO:190437	BP	GO:190437	regulation	14/1035	128/18614	0.011777	0.40922	0.392279	VAMP8/AR	14
GO:007052	BP	GO:007052	protein kin	6/1035	35/18614	0.011828	0.40922	0.392279	GPD1L/DG	6
GO:000838	BP	GO:000838	RNA splicir	38/1035	466/18614	0.011846	0.40922	0.392279	FAM172A/	38
GO:001000	BP	GO:001000	glial cell di	22/1035	235/18614	0.011872	0.40922	0.392279	PRPF19/DA	22
GO:000906	BP	GO:000906	glutamine	10/1035	79/18614	0.011952	0.40922	0.392279	ASRGL1/N	10
GO:001936	BP	GO:001936	pyridine nu	10/1035	79/18614	0.011952	0.40922	0.392279	GPD1L/DC	10
GO:004649	BP	GO:004649	nicotinami	10/1035	79/18614	0.011952	0.40922	0.392279	GPD1L/DC	10
GO:200073	BP	GO:200073	regulation	10/1035	79/18614	0.011952	0.40922	0.392279	FOXC1/PW	10
GO:190165	BP	GO:190165	response t	20/1035	208/18614	0.01216	0.41324	0.396132	SMYD3/CK	20
GO:001097	BP	GO:001097	regulation	37/1035	452/18614	0.012187	0.41324	0.396132	TIAM2/CO	37
GO:009739	BP	GO:009739	response t	4/1035	17/18614	0.01264	0.413448	0.396332	NFKBIZ/TR	4
GO:009739	BP	GO:009739	cellular res	4/1035	17/18614	0.01264	0.413448	0.396332	NFKBIZ/TR	4
GO:190120	BP	GO:190120	regulation	4/1035	17/18614	0.01264	0.413448	0.396332	HAS2/SOX	4
GO:190203	BP	GO:190203	regulation	4/1035	17/18614	0.01264	0.413448	0.396332	FOXC1/KA	4
GO:003496	BP	GO:003496	histone lysi	12/1035	104/18614	0.01273	0.413448	0.396332	SETD4/SM	12
GO:004693	BP	GO:004693	nucleotide	12/1035	104/18614	0.01273	0.413448	0.396332	JMJD8/NM	12
GO:003225	BP	GO:003225	methylatio	31/1035	365/18614	0.012771	0.413448	0.396332	SETD4/SM	31
GO:000202	BP	GO:000202	regulation	11/1035	92/18614	0.012989	0.413448	0.396332	GPD1L/AD	11
GO:004518	BP	GO:004518	maintenan	11/1035	92/18614	0.012989	0.413448	0.396332	FAF1/ANK	11
GO:003133	BP	GO:003133	negative re	23/1035	251/18614	0.013031	0.413448	0.396332	RBM10/MT	23
GO:003386	BP	GO:003386	nucleoside	8/1035	57/18614	0.013044	0.413448	0.396332	PPT1/ACO	8
GO:003403	BP	GO:003403	ribonucleo	8/1035	57/18614	0.013044	0.413448	0.396332	PPT1/ACO	8
GO:003403	BP	GO:003403	purine nucl	8/1035	57/18614	0.013044	0.413448	0.396332	PPT1/ACO	8
GO:000046	BP	GO:000046	maturation	6/1035	36/18614	0.013543	0.413448	0.396332	RCL1/RRS1	6
GO:001073	BP	GO:001073	protein kin	6/1035	36/18614	0.013543	0.413448	0.396332	ADRB2/RD	6
GO:004647	BP	GO:004647	phosphatic	6/1035	36/18614	0.013543	0.413448	0.396332	PLD1/DGK	6
GO:004639	BP	GO:004639	ribose pho	22/1035	238/18614	0.013607	0.413448	0.396332	PPT1/NME	22
GO:000906	BP	GO:000906	fatty acid c	12/1035	105/18614	0.013669	0.413448	0.396332	ACADL/EC	12
GO:005165	BP	GO:005165	establishm	19/1035	197/18614	0.013853	0.413448	0.396332	TRAPPC2L/	19
GO:004427	BP	GO:004427	cellular nitr	39/1035	486/18614	0.013867	0.413448	0.396332	RBM10/AC	39
GO:007182	BP	GO:007182	ribonucleo	22/1035	239/18614	0.014228	0.413448	0.396332	PRPF19/SN	22
GO:200064	BP	GO:200064	regulation	8/1035	58/18614	0.014416	0.413448	0.396332	GPD1L/AN	8
GO:000175	BP	GO:000175	eye photor	7/1035	47/18614	0.014452	0.413448	0.396332	SOX9/PAX	7
GO:000037	BP	GO:000037	RNA splicir	28/1035	325/18614	0.014566	0.413448	0.396332	FAM172A/	28
GO:000039	BP	GO:000039	mRNA spli	28/1035	325/18614	0.014566	0.413448	0.396332	FAM172A/	28
GO:004670	BP	GO:004670	heterocycle	39/1035	488/18614	0.014735	0.413448	0.396332	RBM10/RB	39
GO:009050	BP	GO:009050	RNA phosp	16/1035	158/18614	0.014909	0.413448	0.396332	RCL1/RRS1	16
GO:000663	BP	GO:000663	acyl-CoA r	11/1035	94/18614	0.015102	0.413448	0.396332	PPT1/HMG	11
GO:003538	BP	GO:003538	thioester m	11/1035	94/18614	0.015102	0.413448	0.396332	PPT1/HMG	11
GO:000027	BP	GO:000027	polysaccha	9/1035	70/18614	0.015133	0.413448	0.396332	UGP2/HAS	9
GO:004684	BP	GO:004684	filopodium	9/1035	70/18614	0.015133	0.413448	0.396332	DAAM2/SF	9
GO:003952	BP	GO:003952	RIG-I signa	5/1035	27/18614	0.015287	0.413448	0.396332	PHB2/ZCC	5
GO:004633	BP	GO:004633	diacylglyce	5/1035	27/18614	0.015287	0.413448	0.396332	DGKG/AVI	5
GO:000151	BP	GO:000151	selenocyst	3/1035	10/18614	0.015322	0.413448	0.396332	C6orf52/SE	3
GO:000213	BP	GO:000213	retinoic aci	3/1035	10/18614	0.015322	0.413448	0.396332	ALDH8A1/	3
GO:000645	BP	GO:000645	translation	3/1035	10/18614	0.015322	0.413448	0.396332	C6orf52/SE	3
GO:003112	BP	GO:003112	rRNA 3'-er	3/1035	10/18614	0.015322	0.413448	0.396332	EXOSC10/I	3
GO:003164	BP	GO:003164	negative re	3/1035	10/18614	0.015322	0.413448	0.396332	MTMR2/C	3
GO:190024	BP	GO:190024	positive re	3/1035	10/18614	0.015322	0.413448	0.396332	ZCCHC3/P	3
GO:190162	BP	GO:190162	regulation	3/1035	10/18614	0.015322	0.413448	0.396332	GPR161/SU	3
GO:001009	BP	GO:001009	specificatic	6/1035	37/18614	0.015425	0.413448	0.396332	AR/TBR1/F	6
GO:003817	BP	GO:003817	neurotroph	6/1035	37/18614	0.015425	0.413448	0.396332	MAGI2/DD	6
GO:190274	BP	GO:190274	positive re	6/1035	37/18614	0.015425	0.413448	0.396332	AUTS2/AV	6
GO:000697	BP	GO:000697	DNA dama	4/1035	18/18614	0.01555	0.413448	0.396332	MUC1/KAT	4
GO:003649	BP	GO:003649	PERK-med	4/1035	18/18614	0.01555	0.413448	0.396332	EIF2S1/BO	4
GO:004225	BP	GO:004225	ribosome b	26/1035	298/18614	0.015631	0.413448	0.396332	RCL1/NIFK	26
GO:000597	BP	GO:000597	polysaccha	12/1035	107/18614	0.015706	0.413448	0.396332	CHST1/UG	12
GO:006201	BP	GO:006201	negative re	12/1035	107/18614	0.015706	0.413448	0.396332	ACADL/DD	12
GO:190165	BP	GO:190165	cellular res	12/1035	107/18614	0.015706	0.413448	0.396332	SMYD3/CK	12
GO:000834	BP	GO:000834	glial cell m	8/1035	59/18614	0.01589	0.413448	0.396332	APCDD1/C	8
GO:000916	BP	GO:000916	ribonucleo	8/1035	59/18614	0.01589	0.413448	0.396332	PAICS/PFA	8
GO:001657	BP	GO:001657	histone me	14/1035	133/18614	0.016087	0.413448	0.396332	SETD4/SM	14
GO:003016	BP	GO:003016	platelet act	14/1035	133/18614	0.016087	0.413448	0.396332	AXL/JAK2/	14
GO:004618	BP	GO:004618	phenol-co	7/1035	48/18614	0.016132	0.413448	0.396332	MC1R/MO	7
GO:000599	BP	GO:000599	monosaccl	23/1035	256/18614	0.016149	0.413448	0.396332	GALE/CHS	23
GO:001605	BP	GO:001605	Wnt signali	37/1035	461/18614	0.016182	0.413448	0.396332	APCDD1/T	37
GO:009006	BP	GO:009006	regulation	39/1035	492/18614	0.016605	0.413448	0.396332	ADRB2/SO	39
GO:001607	BP	GO:001607	rRNA meta	23/1035	257/18614	0.016839	0.413448	0.396332	RCL1/NIFK	23
GO:000037	BP	GO:000037	RNA splicir	28/1035	329/18614	0.016887	0.413448	0.396332	FAM172A/	28
GO:005164	BP	GO:005164	vesicle loca	20/1035	215/18614	0.016946	0.413448	0.396332	TRAPPC2L/	20
GO:000195	BP	GO:000195	intestinal D	2/1035	4/18614	0.01719	0.413448	0.396332	EZR/PLS1	2
GO:000655	BP	GO:000655	isoleucine	2/1035	4/18614	0.01719	0.413448	0.396332	ACAT1/BC	2
GO:000918	BP	GO:000918	purine deo	2/1035	4/18614	0.01719	0.413448	0.396332	NUDT18/G	2
GO:003348	BP	GO:003348	cholesterol	2/1035	4/18614	0.01719	0.413448	0.396332	EBP/DHCR	2
GO:003349	BP	GO:003349	cholesterol	2/1035	4/18614	0.01719	0.413448	0.396332	EBP/DHCR	2
GO:003446	BP	GO:003446	response t	2/1035	4/18614	0.01719	0.413448	0.396332	KCNMA1/I	2
GO:003502	BP	GO:003502	negative re	2/1035	4/18614	0.01719	0.413448	0.396332	ARHGAP44	2
GO:004273	BP	GO:004273	D-xylose n	2/1035	4/18614	0.01719	0.413448	0.396332	DCXR/UXS	2
GO:004332	BP	GO:004332	regulation	2/1035	4/18614	0.01719	0.413448	0.396332	LAMP1/HL	2

GO enrichmenet analysis of upregulated DEGs

	ONTOLOG	ID	Descriptior	GeneRatio	BgRatio	pvalue	p.adjust	qvalue	geneID	Count
GO:000645	BP	GO:000645	protein fol	29/902	220/18614	9.62E-07	0.004916	0.004413	BAG2/RP2	29
GO:000648	BP	GO:000648	protein N-	15/902	72/18614	1.47E-06	0.004916	0.004413	DPAGT1/R	15
GO:009031	BP	GO:009031	negative re	11/902	44/18614	5.73E-06	0.010834	0.009725	SUMO1/B	11
GO:003238	BP	GO:003238	negative re	13/902	62/18614	6.80E-06	0.010834	0.009725	SUMO1/B	13
GO:000910	BP	GO:000910	glycoprote	35/902	324/18614	8.10E-06	0.010834	0.009725	DSE/DPAG	35
GO:000910	BP	GO:000910	glycoprote	40/902	401/18614	1.29E-05	0.014354	0.012884	DSE/DPAG	40
GO:004819	BP	GO:004819	Golgi vesic	32/902	294/18614	1.68E-05	0.014544	0.013054	RP2/RAB3	32
GO:001623	BP	GO:001623	macroauto	34/902	322/18614	1.76E-05	0.014544	0.013054	SESN3/SN	34
GO:000599	BP	GO:000599	monosacc	29/902	256/18614	1.96E-05	0.014544	0.013054	IGFBP3/DS	29
GO:007008	BP	GO:007008	glycosylati	28/902	245/18614	2.30E-05	0.014675	0.013171	DPAGT1/R	28
GO:003497	BP	GO:003497	response t	29/902	259/18614	2.44E-05	0.014675	0.013171	TRIB3/CCN	29
GO:005081	BP	GO:005081	regulation	4/902	5/18614	2.63E-05	0.014675	0.013171	PDK2/SNC	4
GO:000648	BP	GO:000648	protein gly	26/902	226/18614	3.98E-05	0.019017	0.017069	DPAGT1/R	26
GO:004341	BP	GO:004341	macromol	26/902	226/18614	3.98E-05	0.019017	0.017069	DPAGT1/R	26
GO:190015	BP	GO:190015	positive re	6/902	15/18614	4.37E-05	0.019482	0.017486	ZFP36L1/T	6
GO:001072	BP	GO:001072	negative re	30/902	286/18614	6.10E-05	0.025487	0.022876	TNFAIP6/I	30
GO:004887	BP	GO:004887	homeostas	31/902	304/18614	7.87E-05	0.029155	0.026169	MAFB/ZFP	31
GO:009988	BP	GO:009988	cellular oxi	15/902	99/18614	8.09E-05	0.029155	0.026169	CYGB/GPX	15
GO:001050	BP	GO:001050	positive re	19/902	146/18614	8.29E-05	0.029155	0.026169	SESN3/SN	19
GO:000215	BP	GO:000215	osteoclast	5/902	11/18614	9.56E-05	0.031953	0.028679	GREM1/TN	5
GO:001827	BP	GO:001827	protein N-	7/902	24/18614	0.000103	0.032687	0.029338	RPN1/MG	7
GO:000600	BP	GO:000600	glucose m	22/902	187/18614	0.00011	0.032889	0.02952	IGFBP3/DY	22
GO:000257	BP	GO:000257	myeloid le	25/902	227/18614	0.000113	0.032889	0.02952	TNFAIP6/N	25
GO:001090	BP	GO:001090	regulation	15/902	103/18614	0.000128	0.034882	0.031309	IGFBP3/DY	15
GO:199074	BP	GO:199074	cellular det	16/902	115/18614	0.000134	0.034882	0.031309	CYGB/GPX	16
GO:003359	BP	GO:003359	response t	20/902	164/18614	0.000136	0.034882	0.031309	CCND1/FI	20
GO:003043	BP	GO:003043	ubiquitin-	13/902	83/18614	0.000169	0.041869	0.03758	SYVN1/HS	13
GO:000698	BP	GO:000698	response t	18/902	142/18614	0.000177	0.042257	0.037928	CCND1/FI	18
GO:004636	BP	GO:004636	monosacc	14/902	96/18614	0.00021	0.048368	0.043413	DSE/DSEL	14
GO:003019	BP	GO:003019	collagen fil	11/902	64/18614	0.000229	0.050852	0.045642	GREM1/EN	11
GO:003096	BP	GO:003096	endoplasm	12/902	75/18614	0.000243	0.050852	0.045642	CCND1/FI	12
GO:003650	BP	GO:003650	ERAD path	15/902	109/18614	0.000243	0.050852	0.045642	SYVN1/HS	15
GO:004684	BP	GO:004684	bone remc	13/902	88/18614	0.000307	0.062221	0.055847	GREM1/ZN	13
GO:009723	BP	GO:009723	cellular res	16/902	124/18614	0.000325	0.063816	0.057279	CYGB/GPX	16
GO:003009	BP	GO:003009	myeloid ce	37/902	421/18614	0.000361	0.065893	0.059143	TNFAIP6/N	37
GO:004276	BP	GO:004276	regulation	5/902	14/18614	0.000367	0.065893	0.059143	PDK2/SNC	5
GO:003097	BP	GO:003097	retrograde	7/902	29/18614	0.000375	0.065893	0.059143	SYVN1/SV	7
GO:190351	BP	GO:190351	endoplasm	7/902	29/18614	0.000375	0.065893	0.059143	SYVN1/SV	7
GO:001050	BP	GO:001050	regulation	32/902	348/18614	0.000405	0.067992	0.061026	TRIB3/SES	32
GO:190210	BP	GO:190210	regulation	30/902	319/18614	0.000417	0.067992	0.061026	TNFAIP6/I	30
GO:001051	BP	GO:001051	regulation	3/902	4/18614	0.000437	0.067992	0.061026	PDK2/PDK	3
GO:190476	BP	GO:190476	negative re	3/902	4/18614	0.000437	0.067992	0.061026	RB1/TP53I	3
GO:200069	BP	GO:200069	negative re	3/902	4/18614	0.000437	0.067992	0.061026	STAT1/CT	3
GO:004870	BP	GO:004870	embryonic	16/902	129/18614	0.000508	0.072199	0.064802	IRX5/SOX1	16
GO:190370	BP	GO:190370	regulation	36/902	415/18614	0.000549	0.072199	0.064802	TNFAIP6/I	36
GO:003021	BP	GO:003021	erythrocyt	16/902	130/18614	0.000554	0.072199	0.064802	MAFB/ZFP	16
GO:000758	BP	GO:000758	response t	18/902	156/18614	0.000567	0.072199	0.064802	CCND1/CY	18
GO:004828	BP	GO:004828	organelle f	18/902	156/18614	0.000567	0.072199	0.064802	CHMP5/G	18
GO:003120	BP	GO:003120	post-trans	4/902	9/18614	0.000567	0.072199	0.064802	SEC61G/H	4
GO:190415	BP	GO:190415	negative re	4/902	9/18614	0.000567	0.072199	0.064802	SVIP/ERLE	4
GO:003257	BP	GO:003257	response t	8/902	40/18614	0.000568	0.072199	0.064802	TGFB2/CY	8
GO:007163	BP	GO:007163	regulation	8/902	40/18614	0.000568	0.072199	0.064802	GATA6/TG	8
GO:003359	BP	GO:003359	cellular res	15/902	118/18614	0.000579	0.072199	0.064802	CCND1/FI	15
GO:000610	BP	GO:000610	regulation	20/902	183/18614	0.000583	0.072199	0.064802	IGFBP3/DY	20
GO:000697	BP	GO:000697	response t	37/902	434/18614	0.00064	0.077132	0.06923	SESN3/CY	37
GO:000226	BP	GO:000226	myeloid ce	19/902	171/18614	0.000646	0.077132	0.06923	MAFB/ZFP	19
GO:003250	BP	GO:003250	endosome	8/902	41/18614	0.000677	0.079375	0.071244	CHMP5/ST	8
GO:000690	BP	GO:000690	vesicle fusi	15/902	120/18614	0.000692	0.079798	0.071623	CHMP5/G	15
GO:001908	BP	GO:001908	viral protei	7/902	32/18614	0.000711	0.08062	0.072361	SPCS3/ST6	7
GO:004856	BP	GO:004856	embryonic	38/902	453/18614	0.00074	0.082445	0.073999	MAFB/ZFP	38
GO:004870	BP	GO:004870	skeletal sy	23/902	229/18614	0.000778	0.08315	0.074632	GREM1/IR	23
GO:003320	BP	GO:003320	tumor necr	14/902	109/18614	0.000786	0.08315	0.074632	ZNF675/LI	14
GO:190210	BP	GO:190210	negative re	14/902	109/18614	0.000786	0.08315	0.074632	TNFAIP6/I	14
GO:000276	BP	GO:000276	negative re	9/902	52/18614	0.000796	0.08315	0.074632	TNFAIP6/N	9
GO:009017	BP	GO:009017	organelle f	15/902	122/18614	0.000824	0.084736	0.076055	CHMP5/G	15
GO:004856	BP	GO:004856	embryonic	7/902	33/18614	0.000866	0.086866	0.077967	TGFB2/SO	7
GO:003462	BP	GO:003462	cellular res	13/902	98/18614	0.000881	0.086866	0.077967	CCND1/FI	13
GO:000276	BP	GO:000276	regulation	15/902	123/18614	0.000897	0.086866	0.077967	TNFAIP6/N	15
GO:000608	BP	GO:000608	acetyl-Co/	4/902	10/18614	0.000909	0.086866	0.077967	PDK2/DLA	4
GO:007008	BP	GO:007008	negative re	4/902	10/18614	0.000909	0.086866	0.077967	SVIP/ERLE	4
GO:007160	BP	GO:007160	transformi	8/902	43/18614	0.000943	0.087952	0.078942	GATA6/TG	8
GO:000662	BP	GO:000662	post-trans	5/902	17/18614	0.001004	0.087952	0.078942	SEC61G/H	5
GO:006033	BP	GO:006033	interferon-	6/902	25/18614	0.00102	0.087952	0.078942	IRF1/NR1H	6
GO:003166	BP	GO:003166	response t	39/902	477/18614	0.001029	0.087952	0.078942	CCND1/SE	39
GO:006035	BP	GO:006035	cartilage d	7/902	34/18614	0.001045	0.087952	0.078942	SHOX2/SE	7
GO:003020	BP	GO:003020	dermatan	3/902	5/18614	0.001054	0.087952	0.078942	DSE/DSEL	3
GO:009067	BP	GO:009067	cell differe	3/902	5/18614	0.001054	0.087952	0.078942	SOD2/FGF	3
GO:190542	BP	GO:190542	vascular as	3/902	5/18614	0.001054	0.087952	0.078942	SOD2/FGF	3
GO:190591	BP	GO:190591	regulation	3/902	5/18614	0.001054	0.087952	0.078942	SOD2/FGF	3
GO:190593	BP	GO:190593	regulation	3/902	5/18614	0.001054	0.087952	0.078942	SOD2/FGF	3
GO:190495	BP	GO:190495	negative re	16/902	138/18614	0.001066	0.087952	0.078942	SUMO1/NI	16
GO:007135	BP	GO:007135	cellular res	23/902	235/18614	0.001106	0.090172	0.080935	ZFP36L1/Z	23
GO:003410	BP	GO:003410	erythrocyt	16/902	139/18614	0.001151	0.092742	0.083241	MAFB/ZFP	16
GO:000182	BP	GO:000182	kidney dev	28/902	310/18614	0.001191	0.09481	0.085098	GREM1/IR	28
GO:190015	BP	GO:190015	regulation	6/902	26/18614	0.001272	0.09888	0.08875	ZFP36L1/T	6
GO:003278	BP	GO:003278	positive re	8/902	45/18614	0.001287	0.09888	0.08875	SUMO1/D	8
GO:009700	BP	GO:009700	energy hor	8/902	45/18614	0.001287	0.09888	0.08875	IRX3/MFSD	8
GO:190370	BP	GO:190370	negative re	14/902	115/18614	0.001336	0.101513	0.091114	TNFAIP6/I	14
GO:003497	BP	GO:003497	protein fol	4/902	11/18614	0.001375	0.103259	0.092681	HSPA5/DN	4
GO:005086	BP	GO:005086	negative re	22/902	225/18614	0.001421	0.105539	0.094727	EMILIN1/I	22
GO:003022	BP	GO:003022	monocyte	7/902	36/18614	0.001491	0.109344	0.098142	ZFP36L1/P	7
GO:003021	BP	GO:003021	T cell diffe	27/902	300/18614	0.001521	0.109344	0.098142	IRF1/MAFE	27
GO:003247	BP	GO:003247	regulation	13/902	104/18614	0.001537	0.109344	0.098142	IRF1/ITCH	13
GO:003260	BP	GO:003260	type I inter	13/902	104/18614	0.001537	0.109344	0.098142	IRF1/ITCH	13
GO:006201	BP	GO:006201	regulation	29/902	331/18614	0.001569	0.110405	0.099095	TRIB3/IGFE	29
GO:007097	BP	GO:007097	protein loc	11/902	80/18614	0.001598	0.110607	0.099276	SPCS3/SE	11
GO:003461	BP	GO:003461	response t	24/902	257/18614	0.00166	0.110607	0.099276	ZFP36L1/Z	24
GO:004269	BP	GO:004269	muscle cell	34/902	410/18614	0.001663	0.110607	0.099276	GREM1/G	34
GO:009875	BP	GO:009875	detoxificati	16/902	144/18614	0.001673	0.110607	0.099276	CYGB/GPX	16
GO:001603	BP	GO:001603	viral proce	35/902	426/18614	0.001677	0.110607	0.099276	ITCH/SPCS	35
GO:001931	BP	GO:001931	hexose me	22/902	228/18614	0.001683	0.110607	0.099276	IGFBP3/DY	22
GO:190313	BP	GO:190313	mononucle	38/902	474/18614	0.001687	0.110607	0.099276	IRF1/MAFE	38
GO:000688	BP	GO:000688	endoplasm	15/902	131/18614	0.001708	0.110802	0.099451	GOLT1B/LI	15
GO:007196	BP	GO:007196	multivesicu	8/902	47/18614	0.001724	0.110802	0.099451	CHMP5/ST	8
GO:003450	BP	GO:003450	protein loc	27/902	304/18614	0.001842	0.113777	0.102121	TRIM8/SU	27
GO:001470	BP	GO:001470	striated mu	24/902	259/18614	0.001842	0.113777	0.102121	GREM1/G	24
GO:007200	BP	GO:007200	renal syste	28/902	320/18614	0.001908	0.113777	0.102121	GREM1/IR	28
GO:007207	BP	GO:007207	kidney epit	16/902	146/18614	0.001931	0.113777	0.102121	GREM1/IR	16
GO:004218	BP	GO:004218	cellular ket	21/902	216/18614	0.001933	0.113777	0.102121	TRIB3/GPD	21
GO:003252	BP	GO:003252	protein exi	8/902	48/18614	0.001982	0.113777	0.102121	SYVN1/SV	8
GO:004504	BP	GO:004504	protein tar	8/902	48/18614	0.001982	0.113777	0.102121	SPCS3/SE	8
GO:004810	BP	GO:004810	somatic st	4/902	12/18614	0.001983	0.113777	0.102121	TGFB2/FZ	4
GO:004682	BP	GO:004682	regulation	13/902	107/18614	0.001993	0.113777	0.102121	SUMO1/H	13
GO:003569	BP	GO:003569	mitochond	3/902	6/18614	0.002031	0.113777	0.102121	SPATA18/	3
GO:190476	BP	GO:190476	regulation	3/902	6/18614	0.002031	0.113777	0.102121	RB1/TP53I	3
GO:001605	BP	GO:001605	vesicle org	31/902	368/18614	0.002048	0.113777	0.102121	BLOC1S6/	31
GO:004870	BP	GO:004870	embryonic	12/902	95/18614	0.002101	0.113777	0.102121	IRX5/SOX1	12
GO:005122	BP	GO:005122	negative re	15/902	134/18614	0.00214	0.113777	0.102121	SUMO1/NI	15
GO:007208	BP	GO:007208	stem cell p	14/902	121/18614	0.002176	0.113777	0.102121	ZFP36L1/V	14
GO:004513	BP	GO:004513	developme	22/902	233/18614	0.002212	0.113777	0.102121	TNFAIP6/C	22
GO:003649	BP	GO:003649	IRE1-medi	5/902	20/18614	0.002228	0.113777	0.102121	FICD/HSPA	5
GO:004565	BP	GO:004565	regulation	5/902	20/18614	0.002228	0.113777	0.102121	ZFP36L1/H	5
GO:007137	BP	GO:007137	cellular res	5/902	20/18614	0.002228	0.113777	0.102121	GATA6/CY	5
GO:007207	BP	GO:007207	nephron tu	5/902	20/18614	0.002228	0.113777	0.102121	GREM1/IR	5
GO:001619	BP	GO:001619	endosoma	23/902	248/18614	0.002242	0.113777	0.102121	SNX18/CH	23
GO:003133	BP	GO:003133	positive re	36/902	450/18614	0.002283	0.113777	0.102121	TRIB3/ITCI	36
GO:000334	BP	GO:000334	negative re	2/902	2/18614	0.002346	0.113777	0.102121	STAT1/CT	2
GO:001932	BP	GO:001932	pentose bi	2/902	2/18614	0.002346	0.113777	0.102121	G6PD/PGD	2
GO:003330	BP	GO:003330	phytol met	2/902	2/18614	0.002346	0.113777	0.102121	PECR/ALDI	2
GO:009029	BP	GO:009029	negative re	2/902	2/18614	0.002346	0.113777	0.102121	GREM1/TN	2
GO:009706	BP	GO:009706	cellular res	2/902	2/18614	0.002346	0.113777	0.102121	GCLM/GCL	2
GO:190024	BP	GO:190024	negative re	2/902	2/18614	0.002346	0.113777	0.102121	FGF9/DNM	2
GO:190317	BP	GO:190317	fatty alcoh	2/902	2/18614	0.002346	0.113777	0.102121	PECR/ALDI	2
GO:190438	BP	GO:190438	cellular res	2/902	2/18614	0.002346	0.113777	0.102121	GCLM/GCL	2
GO:190591	BP	GO:190591	negative re	2/902	2/18614	0.002346	0.113777	0.102121	FGF9/DNM	2
GO:190593	BP	GO:190593	negative re	2/902	2/18614	0.002346	0.113777	0.102121	FGF9/DNM	2
GO:200049	BP	GO:200049	negative re	2/902	2/18614	0.002346	0.113777	0.102121	CYGB/GCL	2
GO:000030	BP	GO:000030	response t	20/902	205/18614	0.002348	0.113777	0.102121	SESN3/CY	20
GO:000165	BP	GO:000165	ureteric bu	12/902	97/18614	0.002509	0.120684	0.108321	GREM1/RA	12
GO:007216	BP	GO:007216	mesoneph	12/902	98/18614	0.002736	0.129738	0.116447	GREM1/RA	12
GO:007216	BP	GO:007216	mesoneph	12/902	98/18614	0.002736	0.129738	0.116447	GREM1/RA	12
GO:006021	BP	GO:006021	positive re	4/902	13/18614	0.002755	0.129738	0.116447	TNRC6B/C	4
GO:001067	BP	GO:001067	regulation	16/902	152/18614	0.002912	0.136145	0.122198	IGFBP3/DY	16
GO:004325	BP	GO:004325	regulation	33/902	409/18614	0.00298	0.138163	0.124008	CYFIP2/SU	33
GO:003315	BP	GO:003315	regulation	21/902	224/18614	0.002996	0.138163	0.124008	SUMO1/B	21
GO:000663	BP	GO:000663	fatty acid r	32/902	394/18614	0.003092	0.141578	0.127074	TRIB3/PTG	32
GO:000206	BP	GO:000206	chondrocy	13/902	113/18614	0.003241	0.14701	0.131949	GREM1/SH	13
GO:014046	BP	GO:014046	integrated	7/902	41/18614	0.003254	0.14701	0.131949	MAFB/HSP	7
GO:007029	BP	GO:007029	renal abso	6/902	31/18614	0.00331	0.148529	0.133313	AMN/HBB	6
GO:007259	BP	GO:007259	establishm	8/902	52/18614	0.003334	0.148613	0.133388	SPCS3/SE	8
GO:004276	BP	GO:004276	very long-	3/902	7/18614	0.003427	0.150619	0.135189	SLC27A2/	3
GO:005094	BP	GO:005094	positive re	3/902	7/18614	0.003427	0.150619	0.135189	BLOC1S6/	3
GO:004561	BP	GO:004561	regulation	20/902	212/18614	0.003461	0.150619	0.135189	IRF1/ZFP3	20
GO:000008	BP	GO:000008	regulation	5/902	22/18614	0.003492	0.150619	0.135189	ID2/RB1/C	5
GO:006071	BP	GO:006071	labyrinthin	5/902	22/18614	0.003492	0.150619	0.135189	ZFP36L1/R	5
GO:000963	BP	GO:000963	response t	22/902	242/18614	0.003521	0.150901	0.135442	GATA6/AH	22
GO:004545	BP	GO:004545	bone resor	9/902	64/18614	0.003553	0.151309	0.135808	ZNF675/TN	9
GO:000084	BP	GO:000084	gonad dev	21/902	228/18614	0.00369	0.154108	0.138321	TNFAIP6/C	21
GO:004873	BP	GO:004873	cardiac mu	22/902	243/18614	0.0037	0.154108	0.138321	GREM1/G	22
GO:000604	BP	GO:000604	UDP-N-ac	4/902	14/18614	0.003711	0.154108	0.138321	DPAGT1/S	4
GO:190415	BP	GO:190415	regulation	4/902	14/18614	0.003711	0.154108	0.138321	SVIP/ERLE	4
GO:005515	BP	GO:005515	response t	11/902	89/18614	0.003759	0.154378	0.138562	GATA6/ZF	11
GO:200064	BP	GO:200064	positive re	8/902	53/18614	0.003764	0.154378	0.138562	VEGFC/SO	8
GO:000182	BP	GO:000182	mesoneph	12/902	102/18614	0.003818	0.154676	0.13883	GREM1/RA	12
GO:000689	BP	GO:000689	post-Golgi	12/902	102/18614	0.003818	0.154676	0.13883	RP2/RAB3	12
GO:001605	BP	GO:001605	carbohydra	20/902	214/18614	0.00385	0.154676	0.13883	DSE/DYRK	20
GO:005140	BP	GO:005140	neuron apo	23/902	259/18614	0.003865	0.154676	0.13883	CCND1/TG	23
GO:004567	BP	GO:004567	negative re	6/902	32/18614	0.00391	0.154676	0.13883	TNFAIP6/N	6
GO:006103	BP	GO:006103	negative re	6/902	32/18614	0.00391	0.154676	0.13883	GREM1/PT	6
GO:003233	BP	GO:003233	regulation	8/902	54/18614	0.004235	0.16389	0.147101	GREM1/SH	8
GO:000858	BP	GO:000858	male gona	15/902	144/18614	0.004278	0.16389	0.147101	CCND1/G	15
GO:004544	BP	GO:004544	fat cell diff	22/902	246/18614	0.004283	0.16389	0.147101	TRIB3/CCN	22
GO:004682	BP	GO:004682	negative re	5/902	23/18614	0.004286	0.16389	0.147101	SUMO1/SI	5
GO:000673	BP	GO:000673	NADP met	7/902	43/18614	0.00429	0.16389	0.147101	ME1/NADI	7
GO:007136	BP	GO:007136	cellular res	7/902	43/18614	0.00429	0.16389	0.147101	ZFP36L1/B	7
GO:007209	BP	GO:007209	regulation	11/902	91/18614	0.004465	0.169627	0.15225	ZFP36L1/V	11
GO:007259	BP	GO:007259	establishm	35/902	453/18614	0.004539	0.171302	0.153753	SPCS3/SU	35
GO:004654	BP	GO:004654	developme	15/902	145/18614	0.004564	0.171302	0.153753	CCND1/G	15
GO:004814	BP	GO:004814	negative re	6/902	33/18614	0.004586	0.171302	0.153753	MED31/TP	6
GO:005121	BP	GO:005121	cartilage d	19/902	203/18614	0.004696	0.172258	0.154611	GREM1/CH	19
GO:000266	BP	GO:000266	negative re	37/902	487/18614	0.004712	0.172258	0.154611	GREM1/EN	37
GO:001936	BP	GO:001936	pyridine nu	10/902	79/18614	0.004724	0.172258	0.154611	ME1/NADI	10
GO:004649	BP	GO:004649	nicotinami	10/902	79/18614	0.004724	0.172258	0.154611	ME1/NADI	10
GO:200123	BP	GO:200123	negative re	21/902	233/18614	0.004741	0.172258	0.154611	SYVN1/TN	21
GO:004555	BP	GO:004555	regulation	15/902	146/18614	0.004866	0.173071	0.15534	TRIB3/ZFP	15
GO:004230	BP	GO:004230	negative re	4/902	15/18614	0.004868	0.173071	0.15534	SUMO1/SI	4
GO:006021	BP	GO:006021	regulation	4/902	15/18614	0.004868	0.173071	0.15534	TNRC6B/C	4
GO:007252	BP	GO:007252	seminifero	4/902	15/18614	0.004868	0.173071	0.15534	WDR48/AD	4
GO:190357	BP	GO:190357	negative re	7/902	44/18614	0.004892	0.173071	0.15534	SYVN1/NR	7
GO:004316	BP	GO:004316	proteasom	34/902	439/18614	0.004922	0.173215	0.15547	TRIB3/ITCI	34
GO:000941	BP	GO:000941	response t	34/902	440/18614	0.005095	0.175807	0.157796	CCND1/G	34
GO:000205	BP	GO:000205	positive re	5/902	24/18614	0.005202	0.175807	0.157796	SHOX2/ST	5
GO:000921	BP	GO:000921	pyrimidine	5/902	24/18614	0.005202	0.175807	0.157796	NTHL1/ME	5
GO:003545	BP	GO:003545	cellular res	5/902	24/18614	0.005202	0.175807	0.157796	IRF1/IFI16/	5
GO:001611	BP	GO:001611	terpenoid	3/902	8/18614	0.005287	0.175807	0.157796	CYP26B1/	3
GO:003644	BP	GO:003644	myofibrobl	3/902	8/18614	0.005287	0.175807	0.157796	RB1/TP53I	3
GO:004500	BP	GO:004500	depyrimidi	3/902	8/18614	0.005287	0.175807	0.157796	NTHL1/ME	3
GO:004810	BP	GO:004810	autophagic	3/902	8/18614	0.005287	0.175807	0.157796	TP53INP1/	3
GO:005065	BP	GO:005065	dermatan	3/902	8/18614	0.005287	0.175807	0.157796	DSE/DSEL	3
GO:005093	BP	GO:005093	regulation	3/902	8/18614	0.005287	0.175807	0.157796	BLOC1S6/	3
GO:006002	BP	GO:006002	hard palat	3/902	8/18614	0.005287	0.175807	0.157796	SOX11/ITG	3
GO:001080	BP	GO:001080	regulation	8/902	56/18614	0.005312	0.175807	0.157796	ZNF675/TN	8
GO:003235	BP	GO:003235	response t	13/902	120/18614	0.00543	0.178857	0.160534	CCND1/CY	13
GO:004856	BP	GO:004856	digestive t	14/902	134/18614	0.005521	0.180949	0.162412	GATA6/TG	14
GO:007259	BP	GO:007259	reactive ox	21/902	237/18614	0.00575	0.187521	0.16831	PMAIP1/C	21
GO:006107	BP	GO:006107	chaperone	9/902	69/18614	0.005901	0.191535	0.171913	HSPB6/DN	9
GO:003238	BP	GO:003238	regulation	27/902	332/18614	0.006135	0.195341	0.17533	SUMO1/B	27
GO:190589	BP	GO:190589	regulation	10/902	82/18614	0.006163	0.195341	0.17533	FICD/PMAI	10
GO:004327	BP	GO:004327	anoikis	6/902	35/18614	0.006192	0.195341	0.17533	BMF/NTRK	6
GO:007265	BP	GO:007265	protein loc	24/902	285/18614	0.006218	0.195341	0.17533	RAP2A/RA	24
GO:005076	BP	GO:005076	negative re	15/902	150/18614	0.006238	0.195341	0.17533	DUSP10/D	15
GO:003233	BP	GO:003233	negative re	5/902	25/18614	0.006247	0.195341	0.17533	GREM1/PT	5
GO:200020	BP	GO:200020	regulation	5/902	25/18614	0.006247	0.195341	0.17533	NTRK2/BR	5
GO:004478	BP	GO:004478	modulatior	7/902	46/18614	0.006286	0.195341	0.17533	PPIB/ZDH	7
GO:004860	BP	GO:004860	reproductiv	25/902	301/18614	0.006301	0.195341	0.17533	TNFAIP6/C	25
GO:004343	BP	GO:004343	negative re	18/902	194/18614	0.006369	0.195341	0.17533	ITCH/ZNF6	18
GO:006219	BP	GO:006219	cellular res	28/902	349/18614	0.006381	0.195341	0.17533	ZFP36L1/C	28
GO:000195	BP	GO:000195	regulation	16/902	165/18614	0.006479	0.195341	0.17533	ZNF675/N	16
GO:000703	BP	GO:000703	vacuolar tr	16/902	165/18614	0.006479	0.195341	0.17533	CHMP5/PL	16
GO:004230	BP	GO:004230	regulation	8/902	58/18614	0.006586	0.195341	0.17533	SUMO1/H	8
GO:003208	BP	GO:003208	negative re	11/902	96/18614	0.006694	0.195341	0.17533	ITCH/ZNF6	11
GO:003133	BP	GO:003133	positive re	18/902	195/18614	0.00671	0.195341	0.17533	SUMO1/B	18
GO:190382	BP	GO:190382	negative re	19/902	210/18614	0.006726	0.195341	0.17533	MAP1A/SU	19
GO:000640	BP	GO:000640	tRNA expo	2/902	3/18614	0.00681	0.195341	0.17533	XPOT/SSB	2
GO:000698	BP	GO:000698	response t	2/902	3/18614	0.00681	0.195341	0.17533	GPX3/MGS	2
GO:000905	BP	GO:000905	pentose-p	2/902	3/18614	0.00681	0.195341	0.17533	G6PD/PGD	2
GO:001967	BP	GO:001967	NAD catab	2/902	3/18614	0.00681	0.195341	0.17533	NUDT12/S	2
GO:002157	BP	GO:002157	rhombome	2/902	3/18614	0.00681	0.195341	0.17533	MAFB/HO	2
GO:003570	BP	GO:003570	monocyte	2/902	3/18614	0.00681	0.195341	0.17533	SH2B3/CS	2
GO:006040	BP	GO:006040	negative re	2/902	3/18614	0.00681	0.195341	0.17533	GDF15/LEF	2
GO:007193	BP	GO:007193	positive re	2/902	3/18614	0.00681	0.195341	0.17533	ID2/ZPR1	2
GO:009732	BP	GO:009732	melanocyt	2/902	3/18614	0.00681	0.195341	0.17533	ZEB2/KITL	2
GO:190409	BP	GO:190409	regulation	2/902	3/18614	0.00681	0.195341	0.17533	DAPK1/LA	2
GO:004851	BP	GO:004851	rhythmic p	25/902	303/18614	0.006837	0.195341	0.17533	TNFAIP6/P	25
GO:003328	BP	GO:003328	response t	23/902	272/18614	0.006949	0.196453	0.176327	TRIB3/ZFP	23
GO:001633	BP	GO:001633	morphoge	15/902	152/18614	0.00698	0.196453	0.176327	GREM1/IR	15
GO:003328	BP	GO:003328	cellular res	19/902	211/18614	0.007068	0.196453	0.176327	TRIB3/ZFP	19
GO:001820	BP	GO:001820	peptidyl-p	7/902	47/18614	0.007084	0.196453	0.176327	FKBP11/PP	7
GO:007084	BP	GO:007084	response t	7/902	47/18614	0.007084	0.196453	0.176327	ZFP36L1/B	7
GO:003292	BP	GO:003292	circadian r	9/902	71/18614	0.00712	0.196453	0.176327	AHR/PPP1	9
GO:000316	BP	GO:000316	cardiac co	6/902	36/18614	0.007132	0.196453	0.176327	GATA6/IR	6
GO:005093	BP	GO:005093	pigment c	6/902	36/18614	0.007132	0.196453	0.176327	BLOC1S6/	6
GO:001607	BP	GO:001607	synaptic ve	11/902	97/18614	0.00723	0.196453	0.176327	CADPS2/B	11
GO:009719	BP	GO:009719	extrinsic ap	20/902	227/18614	0.007359	0.196453	0.176327	TGFB2/PM	20
GO:006145	BP	GO:006145	reproductiv	25/902	305/18614	0.00741	0.196453	0.176327	TNFAIP6/C	25
GO:000863	BP	GO:000863	apoptotic	12/902	111/18614	0.007515	0.196453	0.176327	PMAIP1/IF	12
GO:004632	BP	GO:004632	regulation	14/902	139/18614	0.007589	0.196453	0.176327	MAP4K4/I	14
GO:000283	BP	GO:000283	negative re	13/902	125/18614	0.00762	0.196453	0.176327	ITCH/AHR	13
GO:001985	BP	GO:001985	L-ascorbic	3/902	9/18614	0.007646	0.196453	0.176327	SLC2A3/G	3
GO:003020	BP	GO:003020	dermatan	3/902	9/18614	0.007646	0.196453	0.176327	DSE/DSEL	3
GO:003290	BP	GO:003290	transformi	3/902	9/18614	0.007646	0.196453	0.176327	GATA6/TG	3
GO:003290	BP	GO:003290	regulation	3/902	9/18614	0.007646	0.196453	0.176327	GATA6/TG	3
GO:003573	BP	GO:003573	hepatic ste	3/902	9/18614	0.007646	0.196453	0.176327	CYGB/GCL	3
GO:004808	BP	GO:004808	positive re	3/902	9/18614	0.007646	0.196453	0.176327	BLOC1S6/	3
GO:005277	BP	GO:005277	amino disa	3/902	9/18614	0.007646	0.196453	0.176327	DSE/DSEL	3
GO:007144	BP	GO:007144	cellular res	3/902	9/18614	0.007646	0.196453	0.176327	MGST1/TP	3
GO:009706	BP	GO:009706	ncRNA exp	3/902	9/18614	0.007646	0.196453	0.176327	PHAX/XPO	3
GO:190133	BP	GO:190133	lactone me	3/902	9/18614	0.007646	0.196453	0.176327	SLC2A3/G	3
GO:190138	BP	GO:190138	regulation	3/902	9/18614	0.007646	0.196453	0.176327	GATA6/ITG	3
GO:004666	BP	GO:004666	male sex d	16/902	168/18614	0.007669	0.196453	0.176327	CCND1/G	16
GO:005125	BP	GO:005125	negative re	16/902	168/18614	0.007669	0.196453	0.176327	IRF1/ITCH	16
GO:003164	BP	GO:003164	regulation	26/902	322/18614	0.007731	0.197296	0.177084	BAG2/SYV	26
GO:000608	BP	GO:000608	acetyl-Co/	4/902	17/18614	0.007858	0.198697	0.178342	PDK2/DLA	4
GO:004855	BP	GO:004855	embryonic	4/902	17/18614	0.007858	0.198697	0.178342	SOX11/SH	4
GO:006144	BP	GO:006144	connective	23/902	275/18614	0.007892	0.198697	0.178342	GREM1/AF	23
GO:007252	BP	GO:007252	pyridine-c	10/902	85/18614	0.007921	0.198697	0.178342	ME1/NADI	10
GO:004854	BP	GO:004854	response t	27/902	339/18614	0.008018	0.198697	0.178342	TFPI/CCNE	27
GO:000609	BP	GO:000609	pyruvate m	12/902	112/18614	0.008057	0.198697	0.178342	ME1/PDK2	12
GO:000722	BP	GO:000722	integrin-m	12/902	112/18614	0.008057	0.198697	0.178342	ITGBL1/LI	12
GO:006035	BP	GO:006035	endochon	8/902	60/18614	0.008079	0.198697	0.178342	SHOX2/SE	8
GO:000183	BP	GO:000183	embryonic	13/902	126/18614	0.008132	0.198697	0.178342	GREM1/IR	13
GO:000092	BP	GO:000092	nucleotide	6/902	37/18614	0.008171	0.198697	0.178342	DPAGT1/S	6
GO:004532	BP	GO:004532	late endos	6/902	37/18614	0.008171	0.198697	0.178342	CHMP5/ST	6
GO:004563	BP	GO:004563	regulation	19/902	214/18614	0.008176	0.198697	0.178342	TNFAIP6/N	19
GO:190210	BP	GO:190210	positive re	17/902	184/18614	0.008202	0.198697	0.178342	ZFP36L1/D	17
GO:190370	BP	GO:190370	positive re	17/902	184/18614	0.008202	0.198697	0.178342	ZFP36L1/D	17

KEGG pathway analysis based on downregulated DEGs

	ID	Descriptior	GeneRatio	BgRatio	pvalue	p.adjust	qvalue	geneID	Count
hsa04070	hsa04070	Phosphatic	15/523	97/8662	0.000656	0.217707	0.212599	3628/8027	15
hsa00564	hsa00564	Glycerophc	14/523	99/8662	0.002365	0.237356	0.231787	9468/2317	14
hsa04142	hsa04142	Lysosome	17/523	132/8662	0.002396	0.237356	0.231787	3373/5538	17
hsa00563	hsa00563	Glycosylph	6/523	26/8662	0.003838	0.237356	0.231787	51604/100	6
hsa04144	hsa04144	Endocytosi	26/523	250/8662	0.00456	0.237356	0.231787	381/50807	26
hsa00062	hsa00062	Fatty acid	6/523	27/8662	0.004688	0.237356	0.231787	5538/1133	6
hsa04927	hsa04927	Cortisol sy	10/523	65/8662	0.005241	0.237356	0.231787	84876/544	10
hsa00100	hsa00100	Steroid bic	5/523	20/8662	0.005719	0.237356	0.231787	10682/710	5
hsa01230	hsa01230	Biosynthes	10/523	75/8662	0.014187	0.509811	0.497849	435/5091/	10
hsa04962	hsa04962	Vasopress	7/523	44/8662	0.015356	0.509811	0.497849	1639/109/	7
hsa01212	hsa01212	Fatty acid	8/523	57/8662	0.020273	0.531014	0.518555	5538/33/4	8
hsa00620	hsa00620	Pyruvate m	7/523	47/8662	0.021588	0.531014	0.518555	5162/5091	7
hsa00280	hsa00280	Valine, leu	7/523	48/8662	0.024006	0.531014	0.518555	3157/18/3	7
hsa00785	hsa00785	Lipoic acid	4/523	19/8662	0.024656	0.531014	0.518555	4967/5162	4
hsa05017	hsa05017	Spinocereb	15/523	143/8662	0.025496	0.531014	0.518555	3710/3423	15
hsa00520	hsa00520	Amino sug	7/523	49/8662	0.026604	0.531014	0.518555	2582/5170	7
hsa05132	hsa05132	Salmonella	23/523	249/8662	0.02719	0.531014	0.518555	302/1639/	23
hsa01232	hsa01232	Nucleotide	10/523	85/8662	0.031438	0.579851	0.566246	51251/299	10
hsa05231	hsa05231	Choline me	11/523	98/8662	0.033591	0.586954	0.573183	80736/946	11
hsa00071	hsa00071	Fatty acid	6/523	43/8662	0.0429	0.644819	0.62969	33/38/104	6
hsa01200	hsa01200	Carbon me	12/523	115/8662	0.044279	0.644819	0.62969	4967/5162	12
hsa00230	hsa00230	Purine met	13/523	128/8662	0.044885	0.644819	0.62969	29922/122	13
hsa00440	hsa00440	Phosphona	2/523	6/8662	0.046401	0.644819	0.62969	9468/5833	2
hsa00900	hsa00900	Terpenoid	4/523	23/8662	0.046613	0.644819	0.62969	3157/2224	4
hsa04710	hsa04710	Circadian r	5/523	34/8662	0.05143	0.682997	0.666972	1453/9572	5
hsa03050	hsa03050	Proteasom	6/523	46/8662	0.056739	0.723227	0.706258	5705/5687	6
hsa00240	hsa00240	Pyrimidine	7/523	58/8662	0.058817	0.723227	0.706258	51251/299	7
hsa03030	hsa03030	DNA replic	5/523	36/8662	0.063226	0.724574	0.707574	5984/5780	5
hsa00360	hsa00360	Phenylalan	3/523	16/8662	0.068135	0.724574	0.707574	8639/1644	3
hsa01250	hsa01250	Biosynthes	5/523	37/8662	0.069648	0.724574	0.707574	2582/7360	5
hsa04925	hsa04925	Aldosteror	10/523	98/8662	0.070882	0.724574	0.707574	5587/8487	10
hsa04062	hsa04062	Chemokine	17/523	192/8662	0.072007	0.724574	0.707574	3717/2788	17
hsa00562	hsa00562	Inositol ph	8/523	73/8662	0.072021	0.724574	0.707574	3628/8027	8
hsa00650	hsa00650	Butanoate	4/523	27/8662	0.076602	0.747996	0.730447	3157/18/3	4
hsa04360	hsa04360	Axon guida	16/523	182/8662	0.083163	0.773966	0.755807	64221/533	16
hsa04934	hsa04934	Cushing sy	14/523	155/8662	0.084847	0.773966	0.755807	84876/544	14
hsa04512	hsa04512	ECM-recep	9/523	89/8662	0.087591	0.773966	0.755807	1292/1299	9
hsa05130	hsa05130	Pathogenic	17/523	198/8662	0.089662	0.773966	0.755807	55971/469	17
hsa00511	hsa00511	Other glyc	3/523	18/8662	0.090918	0.773966	0.755807	23324/412	3
hsa01521	hsa01521	EGFR tyros	8/523	79/8662	0.102876	0.842768	0.822995	558/3717/	8
hsa00020	hsa00020	Citrate cyc	4/523	30/8662	0.104077	0.842768	0.822995	4967/5162	4
hsa00983	hsa00983	Drug meta	8/523	80/8662	0.10864	0.856267	0.836177	2941/2992	8
hsa04072	hsa04072	Phospholip	13/523	148/8662	0.110902	0.856267	0.836177	5336/5337	13
hsa04931	hsa04931	Insulin resi	10/523	108/8662	0.11632	0.872591	0.852118	5507/5836	10
hsa04340	hsa04340	Hedgehog	6/523	56/8662	0.120008	0.872591	0.852118	1453/2343	6
hsa03410	hsa03410	Base excisi	5/523	44/8662	0.123984	0.872591	0.852118	4350/5984	5
hsa04915	hsa04915	Estrogen s	12/523	137/8662	0.123987	0.872591	0.852118	5443/3710	12
hsa05167	hsa05167	Kaposi sarc	16/523	194/8662	0.126158	0.872591	0.852118	3717/2788	16
hsa04666	hsa04666	Fc gamma	9/523	97/8662	0.130484	0.884094	0.863351	50807/533	9
hsa04930	hsa04930	Type II dia	5/523	46/8662	0.142299	0.934246	0.912327	5590/2645	5
hsa04911	hsa04911	Insulin secr	8/523	86/8662	0.146783	0.934246	0.912327	3710/3778	8
hsa03430	hsa03430	Mismatch r	3/523	23/8662	0.15881	0.934246	0.912327	5984/5780	3
hsa04137	hsa04137	Mitophagy	9/523	103/8662	0.168724	0.934246	0.912327	25813/196	9
hsa00040	hsa00040	Pentose ar	4/523	36/8662	0.169963	0.934246	0.912327	7360/5118	4
hsa00500	hsa00500	Starch and	4/523	36/8662	0.169963	0.934246	0.912327	7360/5836	4
hsa00780	hsa00780	Biotin meta	1/523	3/8662	0.170438	0.934246	0.912327	3141	1
hsa00310	hsa00310	Lysine deg	6/523	63/8662	0.178694	0.934246	0.912327	64754/569	6
hsa00561	hsa00561	Glycerolipi	6/523	63/8662	0.178694	0.934246	0.912327	1608/1606	6
hsa03250	hsa03250	Viral life cy	6/523	63/8662	0.178694	0.934246	0.912327	51497/459	6
hsa00330	hsa00330	Arginine ar	5/523	50/8662	0.182111	0.934246	0.912327	1152/2832	5
hsa00565	hsa00565	Ether lipid	5/523	50/8662	0.182111	0.934246	0.912327	5337/2364	5
hsa05012	hsa05012	Parkinson	20/523	266/8662	0.182121	0.934246	0.912327	7332/4723	20
hsa00250	hsa00250	Alanine, as	4/523	37/8662	0.182122	0.934246	0.912327	80150/569	4
hsa05216	hsa05216	Thyroid ca	4/523	37/8662	0.182122	0.934246	0.912327	7849/8031	4
hsa05032	hsa05032	Morphine a	8/523	91/8662	0.18291	0.934246	0.912327	2788/109/	8
hsa04928	hsa04928	Parathyroi	9/523	106/8662	0.189613	0.941324	0.919238	5337/3710	9
hsa05330	hsa05330	Allograft re	4/523	38/8662	0.194558	0.941324	0.919238	3106/3134	4
hsa05165	hsa05165	Human pa	24/523	331/8662	0.201107	0.941324	0.919238	1292/4599	24
hsa00270	hsa00270	Cysteine a	5/523	52/8662	0.203404	0.941324	0.919238	3945/2622	5
hsa05014	hsa05014	Amyotropl	26/523	364/8662	0.210828	0.941324	0.919238	4723/1639	26
hsa04611	hsa04611	Platelet act	10/523	124/8662	0.215296	0.941324	0.919238	8673/5336	10
hsa05221	hsa05221	Acute mye	6/523	67/8662	0.216542	0.941324	0.919238	83439/693	6
hsa05416	hsa05416	Viral myoc	6/523	67/8662	0.216542	0.941324	0.919238	3106/8082	6
hsa00260	hsa00260	Glycine, se	4/523	40/8662	0.220167	0.941324	0.919238	2731/2346	4
hsa00290	hsa00290	Valine, leu	1/523	4/8662	0.220543	0.941324	0.919238	587	1
hsa03267	hsa03267	Virion - Ac	1/523	4/8662	0.220543	0.941324	0.919238	4179	1
hsa04810	hsa04810	Regulation	17/523	229/8662	0.220893	0.941324	0.919238	221178/44	17
hsa01040	hsa01040	Biosynthes	3/523	27/8662	0.221154	0.941324	0.919238	11332/401	3
hsa04530	hsa04530	Tight juncti	13/523	170/8662	0.226849	0.953342	0.930974	55114/447	13
hsa05164	hsa05164	Influenza A	13/523	171/8662	0.233054	0.960419	0.937885	4599/3717	13
hsa04120	hsa04120	Ubiquitin m	11/523	142/8662	0.238357	0.960419	0.937885	27339/550	11
hsa04920	hsa04920	Adipocytol	6/523	70/8662	0.246555	0.960419	0.937885	3717/5443	6
hsa00380	hsa00380	Tryptophar	4/523	42/8662	0.246603	0.960419	0.937885	64577/38/	4
hsa05332	hsa05332	Graft-versu	4/523	42/8662	0.246603	0.960419	0.937885	3106/3134	4
hsa00603	hsa00603	Glycosphin	2/523	16/8662	0.251206	0.960419	0.937885	6489/4668	2
hsa04310	hsa04310	Wnt signali	13/523	174/8662	0.252064	0.960419	0.937885	147495/23	13
hsa05205	hsa05205	Proteoglyc	15/523	205/8662	0.256248	0.960419	0.937885	5336/4478	15
hsa04940	hsa04940	Type I diab	4/523	43/8662	0.260074	0.960419	0.937885	3106/3134	4
hsa00524	hsa00524	Neomycin,	1/523	5/8662	0.267627	0.960419	0.937885	2645	1
hsa04923	hsa04923	Regulation	5/523	58/8662	0.271518	0.960419	0.937885	154/109/1	5
hsa05208	hsa05208	Chemical c	16/523	223/8662	0.272238	0.960419	0.937885	5587/3463	16
hsa00910	hsa00910	Nitrogen m	2/523	17/8662	0.274145	0.960419	0.937885	11238/768	2
hsa01524	hsa01524	Platinum d	6/523	73/8662	0.277674	0.960419	0.937885	2941/1193	6
hsa05163	hsa05163	Human cyt	16/523	225/8662	0.284092	0.960419	0.937885	2788/3710	16
hsa00030	hsa00030	Pentose ph	3/523	31/8662	0.287375	0.960419	0.937885	9104/5631	3
hsa00410	hsa00410	beta-Alani	3/523	31/8662	0.287375	0.960419	0.937885	54498/18/	3
hsa04270	hsa04270	Vascular sr	10/523	134/8662	0.290071	0.960419	0.937885	3710/3778	10
hsa04727	hsa04727	GABAergic	7/523	89/8662	0.290551	0.960419	0.937885	2788/109/	7
hsa05235	hsa05235	PD-L1 exp	7/523	89/8662	0.290551	0.960419	0.937885	3717/2743	7
hsa04918	hsa04918	Thyroid ho	6/523	75/8662	0.298903	0.960419	0.937885	3710/7849	6
hsa05170	hsa05170	Human imi	15/523	212/8662	0.299188	0.960419	0.937885	2788/5336	15
hsa04935	hsa04935	Growth ho	9/523	120/8662	0.299382	0.960419	0.937885	3717/5336	9
hsa00052	hsa00052	Galactose	3/523	32/8662	0.304219	0.960419	0.937885	2582/7360	3
hsa04971	hsa04971	Gastric aci	6/523	76/8662	0.309633	0.960419	0.937885	3710/7430	6
hsa04145	hsa04145	Phagosom	11/523	152/8662	0.311106	0.960419	0.937885	3916/3106	11
hsa00400	hsa00400	Phenylalan	1/523	6/8662	0.311873	0.960419	0.937885	5053	1
hsa03265	hsa03265	Virion - Eb	1/523	6/8662	0.311873	0.960419	0.937885	1134	1
hsa05225	hsa05225	Hepatocell	12/523	168/8662	0.315439	0.960419	0.937885	5336/2941	12
hsa00531	hsa00531	Glycosami	2/523	19/8662	0.319837	0.960419	0.937885	3373/1085	2
hsa01210	hsa01210	2-Oxocarb	3/523	33/8662	0.321104	0.960419	0.937885	4967/5162	3
hsa04130	hsa04130	SNARE inte	3/523	33/8662	0.321104	0.960419	0.937885	8673/5127	3
hsa04970	hsa04970	Salivary se	7/523	93/8662	0.329775	0.964133	0.941512	154/3347/	7
hsa04151	hsa04151	PI3K-Akt s	24/523	359/8662	0.330733	0.964133	0.941512	1292/3717	24
hsa03420	hsa03420	Nucleotide	5/523	63/8662	0.331445	0.964133	0.941512	2968/5984	5
hsa04932	hsa04932	Non-alcoh	11/523	155/8662	0.333962	0.964133	0.941512	4723/1965	11
hsa05022	hsa05022	Pathways c	31/523	476/8662	0.35536	0.991485	0.968223	7332/5138	31
hsa05166	hsa05166	Human T-c	15/523	222/8662	0.363737	0.991485	0.968223	3106/293/	15
hsa00770	hsa00770	Pantothen	2/523	21/8662	0.364802	0.991485	0.968223	53354/587	2
hsa05215	hsa05215	Prostate ca	7/523	97/8662	0.369727	0.991485	0.968223	367/2950/	7
hsa04913	hsa04913	Ovarian ste	4/523	51/8662	0.371034	0.991485	0.968223	3949/109/	4
hsa00350	hsa00350	Tyrosine m	3/523	36/8662	0.371668	0.991485	0.968223	2954/8639	3
hsa04725	hsa04725	Cholinergi	8/523	113/8662	0.373777	0.991485	0.968223	3717/2788	8
hsa05020	hsa05020	Prion disea	18/523	272/8662	0.37782	0.991485	0.968223	4723/3710	18
hsa00010	hsa00010	Glycolysis	5/523	67/8662	0.380231	0.991485	0.968223	5162/2027	5
hsa04024	hsa04024	cAMP signa	15/523	225/8662	0.383585	0.991485	0.968223	154/5020/	15
hsa05016	hsa05016	Huntingtor	20/523	306/8662	0.389222	0.991485	0.968223	4723/1639	20
hsa04724	hsa04724	Glutamate	8/523	115/8662	0.392438	0.991485	0.968223	2788/5337	8
hsa00740	hsa00740	Riboflavin	1/523	8/8662	0.392519	0.991485	0.968223	645	1
hsa05412	hsa05412	Arrhythmo	6/523	84/8662	0.396988	0.991485	0.968223	8082/8343	6
hsa05320	hsa05320	Autoimmu	4/523	53/8662	0.398934	0.991485	0.968223	3106/3134	4
hsa04933	hsa04933	AGE-RAGE	7/523	100/8662	0.399897	0.991485	0.968223	3717/5336	7
hsa04723	hsa04723	Retrograde	10/523	148/8662	0.403707	0.991485	0.968223	2788/4723	10
hsa04924	hsa04924	Renin secr	5/523	69/8662	0.404629	0.991485	0.968223	154/84876	5
hsa04916	hsa04916	Melanoger	7/523	101/8662	0.409956	0.991485	0.968223	5443/5138	7
hsa03082	hsa03082	ATP-deper	8/523	117/8662	0.411124	0.991485	0.968223	10856/125	8
hsa00600	hsa00600	Sphingolip	4/523	54/8662	0.412811	0.991485	0.968223	9517/7368	4
hsa04917	hsa04917	Prolactin s	5/523	70/8662	0.416789	0.991485	0.968223	3717/2309	5
hsa05230	hsa05230	Central car	5/523	70/8662	0.416789	0.991485	0.968223	5162/3945	5
hsa04972	hsa04972	Pancreatic	7/523	102/8662	0.420007	0.991485	0.968223	3710/6522	7
hsa03266	hsa03266	Virion - He	1/523	9/8662	0.429232	0.991485	0.968223	29990	1
hsa04722	hsa04722	Neurotrop	8/523	119/8662	0.429792	0.991485	0.968223	5336/7189	8
hsa00534	hsa00534	Glycosami	2/523	24/8662	0.429922	0.991485	0.968223	2132/9957	2
hsa04974	hsa04974	Protein dig	7/523	103/8662	0.430042	0.991485	0.968223	1292/1299	7
hsa05414	hsa05414	Dilated car	7/523	103/8662	0.430042	0.991485	0.968223	8082/109/	7
hsa04540	hsa04540	Gap junctic	6/523	88/8662	0.440743	0.994912	0.971569	1453/3710	6
hsa00982	hsa00982	Drug meta	5/523	72/8662	0.440976	0.994912	0.971569	2941/2330	5
hsa05223	hsa05223	Non-small	5/523	72/8662	0.440976	0.994912	0.971569	5336/2743	5
hsa05169	hsa05169	Epstein-Ba	13/523	202/8662	0.447354	0.994912	0.971569	5336/3106	13
hsa04211	hsa04211	Longevity	6/523	89/8662	0.4516	0.994912	0.971569	2309/109/	6
hsa03440	hsa03440	Homologo	3/523	41/8662	0.453956	0.994912	0.971569	641/57804	3
hsa00480	hsa00480	Glutathion	4/523	57/8662	0.453966	0.994912	0.971569	2941/1193	4
hsa05213	hsa05213	Endometri	4/523	58/8662	0.467481	0.994912	0.971569	2309/8343	4
hsa04922	hsa04922	Glucagon	7/523	107/8662	0.469906	0.994912	0.971569	3710/5162	7
hsa00592	hsa00592	alpha-Linc	2/523	26/8662	0.471323	0.994912	0.971569	30/151056	2
hsa04950	hsa04950	Maturity o	2/523	26/8662	0.471323	0.994912	0.971569	5080/2645	2
hsa03320	hsa03320	PPAR signa	5/523	75/8662	0.476779	0.994912	0.971569	33/3157/1	5
hsa04390	hsa04390	Hippo sign	10/523	157/8662	0.477948	0.994912	0.971569	1453/5138	10
hsa05160	hsa05160	Hepatitis C	10/523	158/8662	0.48611	0.994912	0.971569	4599/1965	10
hsa04066	hsa04066	HIF-1 signa	7/523	109/8662	0.489593	0.994912	0.971569	5336/3570	7
hsa04936	hsa04936	Alcoholic li	9/523	142/8662	0.490056	0.994912	0.971569	7189/2309	9
hsa00790	hsa00790	Folate bios	2/523	27/8662	0.491337	0.994912	0.971569	8836/5053	2
hsa04520	hsa04520	Adherens j	6/523	93/8662	0.494486	0.994912	0.971569	55698/105	6
hsa04912	hsa04912	GnRH signa	6/523	93/8662	0.494486	0.994912	0.971569	4216/5337	6
hsa00130	hsa00130	Ubiquinon	1/523	11/8662	0.496147	0.994912	0.971569	51004	1
hsa05100	hsa05100	Bacterial in	5/523	77/8662	0.500225	0.994912	0.971569	79658/178	5
hsa04213	hsa04213	Longevity	4/523	61/8662	0.507245	0.994912	0.971569	2309/109/	4
hsa00980	hsa00980	Metabolisr	5/523	78/8662	0.511797	0.994912	0.971569	2941/1193	5
hsa04612	hsa04612	Antigen pr	5/523	78/8662	0.511797	0.994912	0.971569	926/3106/	5
hsa03022	hsa03022	Basal trans	3/523	45/8662	0.516421	0.994912	0.971569	9569/2968	3
hsa05150	hsa05150	Staphylocc	6/523	96/8662	0.5259	0.994912	0.971569	629/3880/	6
hsa05217	hsa05217	Basal cell c	4/523	63/8662	0.533003	0.994912	0.971569	51384/834	4
hsa03015	hsa03015	mRNA sun	6/523	97/8662	0.536197	0.994912	0.971569	8761/2521	6
hsa04713	hsa04713	Circadian e	6/523	97/8662	0.536197	0.994912	0.971569	2788/3710	6
hsa04114	hsa04114	Oocyte me	8/523	131/8662	0.539305	0.994912	0.971569	367/3710/	8
hsa04929	hsa04929	GnRH secr	4/523	64/8662	0.545629	0.994912	0.971569	3710/2550	4
hsa00514	hsa00514	Other type	3/523	47/8662	0.546183	0.994912	0.971569	6480/3743	3
hsa04670	hsa04670	Leukocyte	7/523	115/8662	0.547187	0.994912	0.971569	5336/4478	7
hsa00630	hsa00630	Glyoxylate	2/523	30/8662	0.548417	0.994912	0.971569	2731/38	2
hsa01523	hsa01523	Antifolate	2/523	30/8662	0.548417	0.994912	0.971569	8836/3551	2
hsa03450	hsa03450	Non-homo	1/523	13/8662	0.55523	0.994912	0.971569	7518	1
hsa04014	hsa04014	Ras signali	14/523	236/8662	0.567561	0.994912	0.971569	2788/5336	14
hsa04510	hsa04510	Focal adhe	12/523	203/8662	0.573476	0.994912	0.971569	1292/1299	12
hsa05415	hsa05415	Diabetic ca	12/523	203/8662	0.573476	0.994912	0.971569	4723/1340	12
hsa04662	hsa04662	B cell recep	5/523	84/8662	0.578684	0.994912	0.971569	5336/5502	5
hsa05203	hsa05203	Viral carcir	12/523	204/8662	0.58046	0.994912	0.971569	3106/2968	12
hsa01240	hsa01240	Biosynthes	9/523	153/8662	0.581602	0.994912	0.971569	7360/2992	9
hsa00533	hsa00533	Glycosami	1/523	14/8662	0.582125	0.994912	0.971569	8534	1
hsa04210	hsa04210	Apoptosis	8/523	136/8662	0.582567	0.994912	0.971569	3710/1965	8
hsa00640	hsa00640	Propanoat	2/523	32/8662	0.5839	0.994912	0.971569	18/3945	2
hsa05142	hsa05142	Chagas dis	6/523	102/8662	0.586155	0.994912	0.971569	7189/107/	6
hsa05110	hsa05110	Vibrio chol	3/523	50/8662	0.588752	0.994912	0.971569	5336/109/	3
hsa04910	hsa04910	Insulin sign	8/523	137/8662	0.590991	0.994912	0.971569	10580/550	8
hsa05135	hsa05135	Yersinia inf	8/523	137/8662	0.590991	0.994912	0.971569	926/10451	8
hsa05162	hsa05162	Measles	8/523	138/8662	0.599331	0.994912	0.971569	4599/4478	8
hsa04610	hsa04610	Compleme	5/523	86/8662	0.599875	0.994912	0.971569	5265/722/	5
hsa04071	hsa04071	Sphingolip	7/523	121/8662	0.601868	0.994912	0.971569	5337/9517	7
hsa04660	hsa04660	T cell recep	7/523	121/8662	0.601868	0.994912	0.971569	926/10451	7
hsa04979	hsa04979	Cholestero	3/523	51/8662	0.602359	0.994912	0.971569	3949/706/	3
hsa04150	hsa04150	mTOR sign	9/523	156/8662	0.605274	0.994912	0.971569	51384/545	9
hsa00604	hsa00604	Glycosphin	1/523	15/8662	0.607396	0.994912	0.971569	6489	1
hsa00730	hsa00730	Thiamine n	1/523	15/8662	0.607396	0.994912	0.971569	122481	1
hsa05418	hsa05418	Fluid shear	8/523	139/8662	0.607587	0.994912	0.971569	2941/5385	8
hsa05204	hsa05204	Chemical c	4/523	70/8662	0.617417	0.994912	0.971569	2941/1193	4
hsa04514	hsa04514	Cell adhesi	9/523	158/8662	0.620682	0.994912	0.971569	9369/926/	9
hsa04814	hsa04814	Motor prot	11/523	193/8662	0.623155	0.994912	0.971569	1639/4645	11
hsa04976	hsa04976	Bile secreti	5/523	89/8662	0.630504	0.994912	0.971569	6522/3949	5
hsa00430	hsa00430	Taurine an	1/523	16/8662	0.631142	0.994912	0.971569	2330	1
hsa05010	hsa05010	Alzheimer	22/523	384/8662	0.634426	0.994912	0.971569	51384/472	22
hsa05207	hsa05207	Chemical c	12/523	212/8662	0.634477	0.994912	0.971569	154/3710/	12
hsa04550	hsa04550	Signaling p	8/523	143/8662	0.639714	0.994912	0.971569	3717/5138	8
hsa03013	hsa03013	Nucleocyt	6/523	108/8662	0.642229	0.994912	0.971569	51068/848	6
hsa04350	hsa04350	TGF-beta s	6/523	108/8662	0.642229	0.994912	0.971569	4756/5696	6
hsa04659	hsa04659	Th17 cell d	6/523	108/8662	0.642229	0.994912	0.971569	3717/3570	6
hsa05161	hsa05161	Hepatitis B	9/523	162/8662	0.650535	0.994912	0.971569	3717/7189	9
hsa04115	hsa04115	p53 signali	4/523	74/8662	0.661144	0.994912	0.971569	5268/2810	4
hsa04960	hsa04960	Aldosteror	2/523	37/8662	0.663439	0.994912	0.971569	2810/5293	2
hsa03040	hsa03040	Spliceosor	12/523	217/8662	0.666353	0.994912	0.971569	27339/663	12
hsa05145	hsa05145	Toxoplasm	6/523	111/8662	0.668494	0.994912	0.971569	3717/7189	6
hsa04926	hsa04926	Relaxin sig	7/523	129/8662	0.669054	0.994912	0.971569	2788/109/	7
hsa00061	hsa00061	Fatty acid	1/523	18/8662	0.67442	0.994912	0.971569	51102	1
hsa05340	hsa05340	Primary im	2/523	38/8662	0.677796	0.994912	0.971569	926/84876	2
hsa05133	hsa05133	Pertussis	4/523	76/8662	0.681696	0.994912	0.971569	722/7189/	4
hsa05212	hsa05212	Pancreatic	4/523	76/8662	0.681696	0.994912	0.971569	5337/5293	4
hsa03008	hsa03008	Ribosome	9/523	167/8662	0.685919	0.994912	0.971569	10171/209	9
hsa04022	hsa04022	cGMP-PKG	9/523	167/8662	0.685919	0.994912	0.971569	154/9569/	9
hsa04728	hsa04728	Dopamine	7/523	132/8662	0.692363	0.994912	0.971569	2788/3710	7
hsa04726	hsa04726	Serotoner	6/523	115/8662	0.701562	0.994912	0.971569	2788/3710	6
hsa05410	hsa05410	Hypertropl	5/523	97/8662	0.704875	0.994912	0.971569	8082/1837	5
hsa00190	hsa00190	Oxidative	7/523	134/8662	0.707301	0.994912	0.971569	4723/1340	7
hsa03018	hsa03018	RNA degra	4/523	79/8662	0.71086	0.994912	0.971569	5394/8761	4
hsa04978	hsa04978	Mineral ab	3/523	60/8662	0.71107	0.994912	0.971569	4495/4494	3
hsa04750	hsa04750	Inflammat	5/523	98/8662	0.713395	0.994912	0.971569	5336/3710	5
hsa04216	hsa04216	Ferroptosis	2/523	41/8662	0.717885	0.994912	0.971569	8031/4407	2
hsa04261	hsa04261	Adrenergic	8/523	154/8662	0.719935	0.994912	0.971569	154/109/5	8
hsa04015	hsa04015	Rap1 signa	11/523	210/8662	0.73052	0.994912	0.971569	5587/9863	11
hsa04218	hsa04218	Cellular se	8/523	156/8662	0.733159	0.994912	0.971569	3710/3106	8
hsa04975	hsa04975	Fat digesti	2/523	43/8662	0.742218	0.994912	0.971569	38/4547	2
hsa04371	hsa04371	Apelin sigr	7/523	139/8662	0.742505	0.994912	0.971569	2788/3710	7
hsa05146	hsa05146	Amoebiasi	5/523	102/8662	0.74573	0.994912	0.971569	5273/107/	5
hsa00220	hsa00220	Arginine bi	1/523	22/8662	0.74636	0.994912	0.971569	435	1
hsa04146	hsa04146	Peroxisom	4/523	83/8662	0.746643	0.994912	0.971569	4358/30/1	4
hsa04152	hsa04152	AMPK sign	6/523	121/8662	0.746867	0.994912	0.971569	2309/5525	6
hsa04714	hsa04714	Thermoge	12/523	232/8662	0.751953	0.994912	0.971569	4723/1340	12
hsa05417	hsa05417	Lipid and a	11/523	215/8662	0.757952	0.994912	0.971569	3717/1045	11
hsa05321	hsa05321	Inflammat	3/523	65/8662	0.7608	0.994912	0.971569	6097/3108	3
hsa04064	hsa04064	NF-kappa	5/523	104/8662	0.760855	0.994912	0.971569	5336/7189	5
hsa04625	hsa04625	C-type lec	5/523	104/8662	0.760855	0.994912	0.971569	5336/3710	5
hsa00515	hsa00515	Mannose t	1/523	23/8662	0.761714	0.994912	0.971569	9486	1
hsa04614	hsa04614	Renin-ang	1/523	23/8662	0.761714	0.994912	0.971569	183	1
hsa04012	hsa04012	ErbB signa	4/523	85/8662	0.763223	0.994912	0.971569	5336/8440	4
hsa02010	hsa02010	ABC transp	2/523	45/8662	0.764733	0.994912	0.971569	23457/22	2
hsa00970	hsa00970	Aminoacyl	3/523	66/8662	0.76987	0.994912	0.971569	10667/283	3
hsa04977	hsa04977	Vitamin di	1/523	24/8662	0.776139	0.994912	0.971569	151056	1
hsa04260	hsa04260	Cardiac mu	4/523	87/8662	0.778947	0.994912	0.971569	1340/1351	4
hsa04664	hsa04664	Fc epsilon	3/523	68/8662	0.787167	0.994912	0.971569	5336/1045	3
hsa04140	hsa04140	Autophagy	8/523	165/8662	0.787336	0.994912	0.971569	8673/3916	8
hsa04080	hsa04080	Neuroactiv	19/523	367/8662	0.790976	0.994912	0.971569	154/5020/	19
hsa05224	hsa05224	Breast can	7/523	147/8662	0.792438	0.994912	0.971569	51384/224	7
hsa05120	hsa05120	Epithelial c	3/523	70/8662	0.803373	0.994912	0.971569	5336/537/	3
hsa05030	hsa05030	Cocaine ac	2/523	49/8662	0.80468	0.994912	0.971569	9586/1644	2
hsa04622	hsa04622	RIG-I-like	3/523	71/8662	0.81108	0.994912	0.971569	7189/4214	3
hsa04141	hsa04141	Protein prc	8/523	170/8662	0.813708	0.994912	0.971569	55968/196	8
hsa04650	hsa04650	Natural kill	6/523	132/8662	0.816597	0.994912	0.971569	5336/1045	6
hsa00601	hsa00601	Glycosphin	1/523	28/8662	0.825635	0.994912	0.971569	6489	1
hsa04657	hsa04657	IL-17 signa	4/523	94/8662	0.827542	0.994912	0.971569	7189/1075	4
hsa04744	hsa04744	Phototrans	1/523	29/8662	0.836197	0.994912	0.971569	2792	1
hsa00510	hsa00510	N-Glycan I	2/523	53/8662	0.838485	0.994912	0.971569	6480/5434	2
hsa04961	hsa04961	Endocrine	2/523	53/8662	0.838485	0.994912	0.971569	1785/1759	2
hsa04148	hsa04148	Efferocyto	7/523	156/8662	0.839493	0.994912	0.971569	558/3717/	7
hsa04110	hsa04110	Cell cycle	7/523	157/8662	0.844154	0.994912	0.971569	2810/1032	7
hsa03460	hsa03460	Fanconi an	2/523	54/8662	0.846067	0.994912	0.971569	116028/64	2
hsa00053	hsa00053	Ascorbate	1/523	30/8662	0.84612	0.994912	0.971569	9104	1
hsa00591	hsa00591	Linoleic ac	1/523	30/8662	0.84612	0.994912	0.971569	151056	1
hsa01522	hsa01522	Endocrine	4/523	98/8662	0.851104	0.994912	0.971569	109/107/5	4
hsa05140	hsa05140	Leishmania	3/523	77/8662	0.852134	0.994912	0.971569	3717/7189	3
hsa04217	hsa04217	Necroptos	7/523	159/8662	0.853152	0.994912	0.971569	3717/5478	7

KEGG pathway analysis based on upregulated DEGs

	ID	Descriptior	GeneRatio	BgRatio	pvalue	p.adjust	qvalue	geneID	Count
hsa04141	hsa04141	Protein prc	34/482	170/8662	4.09E-11	1.27E-08	1.23E-08	9532/6184	34
hsa03060	hsa03060	Protein exp	8/482	31/8662	0.000219	0.034002	0.032905	60559/234	8
hsa00510	hsa00510	N-Glycan I	10/482	53/8662	0.00058	0.059957	0.058023	1798/6184	10
hsa04668	hsa04668	TNF signali	15/482	114/8662	0.00156	0.118669	0.114841	3659/8373	15
hsa00513	hsa00513	Various typ	8/482	42/8662	0.001914	0.118669	0.114841	6184/4247	8
hsa00480	hsa00480	Glutathion	9/482	57/8662	0.003916	0.202352	0.195824	2879/2539	9
hsa05222	hsa05222	Small cell l	12/482	92/8662	0.00479	0.212125	0.205283	595/1164/	12
hsa04216	hsa04216	Ferroptosis	7/482	41/8662	0.006836	0.256519	0.248245	2879/3006	7
hsa00604	hsa00604	Glycosphin	4/482	15/8662	0.007907	0.256519	0.248245	27090/870	4
hsa04130	hsa04130	SNARE inte	6/482	33/8662	0.008824	0.256519	0.248245	9570/6811	6
hsa04510	hsa04510	Focal adhe	20/482	203/8662	0.009102	0.256519	0.248245	595/22801	20
hsa05165	hsa05165	Human pa	29/482	331/8662	0.009931	0.256558	0.248282	595/3659/	29
hsa05132	hsa05132	Salmonella	23/482	249/8662	0.011423	0.272394	0.263607	7277/2364	23
hsa04218	hsa04218	Cellular se	16/482	156/8662	0.013001	0.287889	0.278603	595/3486/	16
hsa04142	hsa04142	Lysosome	14/482	132/8662	0.014848	0.297618	0.288018	968/23545	14
hsa00760	hsa00760	Nicotinate	6/482	37/8662	0.015361	0.297618	0.288018	133686/51	6
hsa05169	hsa05169	Epstein-Ba	19/482	202/8662	0.017129	0.312359	0.302283	595/7128/	19
hsa00590	hsa00590	Arachidoni	8/482	61/8662	0.019026	0.327663	0.317093	5730/2879	8
hsa04115	hsa04115	p53 signali	9/482	74/8662	0.020978	0.342272	0.331231	595/3486/	9
hsa04933	hsa04933	AGE-RAGE	11/482	100/8662	0.022735	0.345831	0.334675	595/7042/	11
hsa05212	hsa05212	Pancreatic	9/482	76/8662	0.024543	0.345831	0.334675	595/7042/	9
hsa05220	hsa05220	Chronic my	9/482	76/8662	0.024543	0.345831	0.334675	595/7042/	9
hsa00532	hsa00532	Glycosami	4/482	21/8662	0.026633	0.358966	0.347387	29940/921	4
hsa04917	hsa04917	Prolactin si	8/482	70/8662	0.039663	0.512312	0.495786	595/3659/	8
hsa05225	hsa05225	Hepatocell	15/482	168/8662	0.047077	0.583753	0.564922	595/7042/	15
hsa01200	hsa01200	Carbon me	11/482	115/8662	0.054752	0.652812	0.631753	50/4199/6	11
hsa05110	hsa05110	Vibrio chol	6/482	50/8662	0.057577	0.661068	0.639743	23480/235	6
hsa04068	hsa04068	FoxO signa	12/482	131/8662	0.060509	0.669923	0.648312	595/7042/	12
hsa04722	hsa04722	Neurotropl	11/482	119/8662	0.066774	0.696749	0.674273	10019/590	11
hsa04612	hsa04612	Antigen pr	8/482	78/8662	0.067427	0.696749	0.674273	3309/5720	8
hsa04010	hsa04010	MAPK sign	23/482	301/8662	0.075198	0.730509	0.706944	9448/1122	23
hsa05219	hsa05219	Bladder ca	5/482	41/8662	0.075407	0.730509	0.706944	595/5925/	5
hsa04630	hsa04630	JAK-STAT	14/482	166/8662	0.078446	0.735239	0.711522	595/9306/	14
hsa05145	hsa05145	Toxoplasm	10/482	111/8662	0.089192	0.735239	0.711522	23643/704	10
hsa04146	hsa04146	Peroxisom	8/482	83/8662	0.089654	0.735239	0.711522	84188/110	8
hsa04623	hsa04623	Cytosolic D	8/482	83/8662	0.089654	0.735239	0.711522	1147/3428	8
hsa05215	hsa05215	Prostate ca	9/482	97/8662	0.089924	0.735239	0.711522	595/1147/	9
hsa00410	hsa00410	beta-Alani	4/482	31/8662	0.090951	0.735239	0.711522	26275/189	4
hsa04120	hsa04120	Ubiquitin n	12/482	142/8662	0.096994	0.735239	0.711522	83737/140	12
hsa04936	hsa04936	Alcoholic li	12/482	142/8662	0.096994	0.735239	0.711522	595/23643	12
hsa04390	hsa04390	Hippo sign	13/482	157/8662	0.097707	0.735239	0.711522	595/7042/	13
hsa04215	hsa04215	Apoptosis	4/482	32/8662	0.099613	0.735239	0.711522	5366/637/	4
hsa04144	hsa04144	Endocytosi	19/482	250/8662	0.102945	0.742163	0.718223	83737/110	19
hsa00130	hsa00130	Ubiquinon	2/482	11/8662	0.121921	0.810744	0.784591	1728/2677	2
hsa04064	hsa04064	NF-kappa	9/482	104/8662	0.124155	0.810744	0.784591	23643/712	9
hsa05226	hsa05226	Gastric car	12/482	149/8662	0.125926	0.810744	0.784591	595/7042/	12
hsa04140	hsa04140	Autophagy	13/482	165/8662	0.129437	0.810744	0.784591	3920/2998	13
hsa05100	hsa05100	Bacterial in	7/482	77/8662	0.135562	0.810744	0.784591	63916/984	7
hsa04919	hsa04919	Thyroid ho	10/482	121/8662	0.13586	0.810744	0.784591	595/29079	10
hsa05130	hsa05130	Pathogenic	15/482	198/8662	0.138397	0.810744	0.784591	7277/2699	15
hsa04145	hsa04145	Phagosom	12/482	152/8662	0.139661	0.810744	0.784591	7277/2348	12
hsa05417	hsa05417	Lipid and a	16/482	215/8662	0.143896	0.810744	0.784591	23643/330	16
hsa04151	hsa04151	PI3K-Akt s	25/482	359/8662	0.144247	0.810744	0.784591	595/22801	25
hsa05162	hsa05162	Measles	11/482	138/8662	0.145745	0.810744	0.784591	595/7128/	11
hsa00534	hsa00534	Glycosami	3/482	24/8662	0.146289	0.810744	0.784591	2135/1128	3
hsa04659	hsa04659	Th17 cell d	9/482	108/8662	0.146457	0.810744	0.784591	196/1147/	9
hsa05418	hsa05418	Fluid shear	11/482	139/8662	0.150883	0.820594	0.794123	7341/1147	11
hsa04932	hsa04932	Non-alcoh	12/482	155/8662	0.154173	0.824027	0.797446	83737/469	12
hsa05415	hsa05415	Diabetic ca	15/482	203/8662	0.159625	0.838709	0.811653	4698/7042	15
hsa05205	hsa05205	Proteoglyc	15/482	205/8662	0.168574	0.870964	0.842868	595/7042/	15
hsa00533	hsa00533	Glycosami	2/482	14/8662	0.18119	0.9208	0.891097	10678/930	2
hsa00564	hsa00564	Glycerophc	8/482	99/8662	0.185123	0.924206	0.894393	2820/1103	8
hsa00790	hsa00790	Folate bios	3/482	27/8662	0.187823	0.924206	0.894393	8644/6697	3
hsa04920	hsa04920	Adipocytol	6/482	70/8662	0.193069	0.935177	0.90501	1147/4794	6
hsa04072	hsa04072	Phospholip	11/482	148/8662	0.201106	0.936597	0.906384	9267/2846	11
hsa05210	hsa05210	Colorectal	7/482	86/8662	0.201332	0.936597	0.906384	595/7042/	7
hsa05152	hsa05152	Tuberculos	13/482	180/8662	0.202426	0.936597	0.906384	7042/2354	13
hsa05218	hsa05218	Melanoma	6/482	72/8662	0.210624	0.960199	0.929225	595/5925/	6
hsa00071	hsa00071	Fatty acid	4/482	43/8662	0.215665	0.96059	0.929603	1892/224/	4
hsa04380	hsa04380	Osteoclast	10/482	135/8662	0.218587	0.96059	0.929603	7042/1147	10
hsa04210	hsa04210	Apoptosis	10/482	136/8662	0.225161	0.96059	0.929603	7277/5366	10
hsa04512	hsa04512	ECM-recep	7/482	89/8662	0.225611	0.96059	0.929603	22801/369	7
hsa03410	hsa03410	Base excisi	4/482	44/8662	0.227662	0.96059	0.929603	4913/8930	4
hsa00020	hsa00020	Citrate cyc	3/482	30/8662	0.231925	0.96059	0.929603	50/6392/1	3
hsa05214	hsa05214	Glioma	6/482	75/8662	0.23794	0.96059	0.929603	595/5925/	6
hsa02010	hsa02010	ABC transp	4/482	45/8662	0.239824	0.96059	0.929603	1672/6890	4
hsa04014	hsa04014	Ras signali	16/482	236/8662	0.240854	0.96059	0.929603	7424/5906	16
hsa00450	hsa00450	Selenocom	2/482	17/8662	0.243651	0.96059	0.929603	22928/105	2
hsa04148	hsa04148	Efferocyto	11/482	156/8662	0.25105	0.96059	0.929603	10062/573	11
hsa03050	hsa03050	Proteasom	4/482	46/8662	0.25213	0.96059	0.929603	5720/5137	4
hsa04931	hsa04931	Insulin resi	8/482	108/8662	0.252427	0.96059	0.929603	57761/100	8
hsa05166	hsa05166	Human T-c	15/482	222/8662	0.254092	0.96059	0.929603	595/7042/	15
hsa00640	hsa00640	Propanoat	3/482	32/8662	0.262279	0.964886	0.93376	26275/189	3
hsa05160	hsa05160	Hepatitis C	11/482	158/8662	0.264185	0.964886	0.93376	595/10062	11
hsa00620	hsa00620	Pyruvate m	4/482	47/8662	0.264565	0.964886	0.93376	4199/1737	4
hsa04217	hsa04217	Necroptos	11/482	159/8662	0.270838	0.976276	0.944783	51510/712	11
hsa05202	hsa05202	Transcripti	13/482	193/8662	0.27762	0.989219	0.957309	3486/3398	13
hsa03020	hsa03020	RNA polyn	3/482	34/8662	0.29309	1	0.967742	5440/9533	3
hsa05321	hsa05321	Inflammat	5/482	65/8662	0.294291	1	0.967742	7042/3117	5
hsa05224	hsa05224	Breast can	10/482	147/8662	0.301899	1	0.967742	595/8324/	10
hsa01522	hsa01522	Endocrine	7/482	98/8662	0.303552	1	0.967742	595/5925/	7
hsa00512	hsa00512	Mucin type	3/482	36/8662	0.324119	1	0.967742	27090/112	3
hsa05211	hsa05211	Renal cell	5/482	69/8662	0.338709	1	0.967742	7042/5906	5
hsa05143	hsa05143	African try	3/482	37/8662	0.339648	1	0.967742	3043/8542	3
hsa05216	hsa05216	Thyroid ca	3/482	37/8662	0.339648	1	0.967742	595/1647/	3
hsa01240	hsa01240	Biosynthes	10/482	153/8662	0.346288	1	0.967742	133686/51	10
hsa00340	hsa00340	Histidine m	2/482	22/8662	0.34844	1	0.967742	224/3176	2
hsa05120	hsa05120	Epithelial c	5/482	70/8662	0.349913	1	0.967742	23545/114	5
hsa00600	hsa00600	Sphingolip	4/482	54/8662	0.353788	1	0.967742	2531/2717	4
hsa04625	hsa04625	C-type lec	7/482	104/8662	0.358243	1	0.967742	3659/602/	7
hsa05223	hsa05223	Non-small	5/482	72/8662	0.372367	1	0.967742	595/5925/	5
hsa04211	hsa04211	Longevity	6/482	89/8662	0.375305	1	0.967742	143686/84	6
hsa05235	hsa05235	PD-L1 exp	6/482	89/8662	0.375305	1	0.967742	1147/4794	6
hsa04110	hsa04110	Cell cycle	10/482	157/8662	0.376455	1	0.967742	595/23244	10
hsa05134	hsa05134	Legionellos	4/482	56/8662	0.379509	1	0.967742	3304/840/	4
hsa01524	hsa01524	Platinum d	5/482	73/8662	0.3836	1	0.967742	5366/637/	5
hsa03013	hsa03013	Nucleocyt	7/482	108/8662	0.395207	1	0.967742	7341/5180	7
hsa04620	hsa04620	Toll-like re	7/482	108/8662	0.395207	1	0.967742	23643/114	7
hsa05131	hsa05131	Shigellosis	15/482	247/8662	0.40118	1	0.967742	11335/926	15
hsa00240	hsa00240	Pyrimidine	4/482	58/8662	0.405123	1	0.967742	51727/124	4
hsa05213	hsa05213	Endometri	4/482	58/8662	0.405123	1	0.967742	595/1647/	4
hsa04658	hsa04658	Th1 and Th	6/482	92/8662	0.405596	1	0.967742	1147/3117	6
hsa03320	hsa03320	PPAR signa	5/482	75/8662	0.406029	1	0.967742	4199/1006	5
hsa04918	hsa04918	Thyroid ho	5/482	75/8662	0.406029	1	0.967742	6528/3309	5
hsa05161	hsa05161	Hepatitis B	10/482	162/8662	0.414472	1	0.967742	7042/637/	10
hsa00380	hsa00380	Tryptopha	3/482	42/8662	0.41648	1	0.967742	1545/1892	3
hsa05133	hsa05133	Pertussis	5/482	76/8662	0.417209	1	0.967742	3659/2364	5
hsa04657	hsa04657	IL-17 signa	6/482	94/8662	0.425737	1	0.967742	7128/1147	6
hsa00592	hsa00592	alpha-Linc	2/482	26/8662	0.428752	1	0.967742	81579/51	2
hsa04978	hsa04978	Mineral ab	4/482	60/8662	0.430532	1	0.967742	26872/300	4
hsa00980	hsa00980	Metabolisr	5/482	78/8662	0.439457	1	0.967742	1545/1555	5
hsa04721	hsa04721	Synaptic ve	5/482	78/8662	0.439457	1	0.967742	23545/117	5
hsa04213	hsa04213	Longevity	4/482	61/8662	0.44313	1	0.967742	3304/5290	4
hsa05410	hsa05410	Hypertropl	6/482	97/8662	0.455746	1	0.967742	7042/2280	6
hsa04621	hsa04621	NOD-like r	11/482	186/8662	0.462303	1	0.967742	7128/1147	11
hsa00561	hsa00561	Glycerolipi	4/482	63/8662	0.468063	1	0.967742	1609/2717	4
hsa05217	hsa05217	Basal cell c	4/482	63/8662	0.468063	1	0.967742	8324/8323	4
hsa04392	hsa04392	Hippo sign	2/482	29/8662	0.485443	1	0.967742	55233/126	2
hsa04915	hsa04915	Estrogen s	8/482	137/8662	0.496625	1	0.967742	3320/3304	8
hsa01523	hsa01523	Antifolate	2/482	30/8662	0.503551	1	0.967742	1147/7298	2
hsa00280	hsa00280	Valine, leu	3/482	48/8662	0.504416	1	0.967742	26275/189	3
hsa04662	hsa04662	B cell recep	5/482	84/8662	0.504773	1	0.967742	1147/4794	5
hsa05146	hsa05146	Amoebiasi	6/482	102/8662	0.504846	1	0.967742	7042/3911	6
hsa04150	hsa04150	mTOR sign	9/482	156/8662	0.505035	1	0.967742	8324/1147	9
hsa04814	hsa04814	Motor prot	11/482	193/8662	0.51223	1	0.967742	7277/8655	11
hsa04152	hsa04152	AMPK sign	7/482	121/8662	0.513805	1	0.967742	595/8408/	7
hsa04137	hsa04137	Mitophagy	6/482	103/8662	0.514484	1	0.967742	84315/840	6
hsa04974	hsa04974	Protein dig	6/482	103/8662	0.514484	1	0.967742	1303/8157	6
hsa05414	hsa05414	Dilated car	6/482	103/8662	0.514484	1	0.967742	7042/2280	6
hsa01232	hsa01232	Nucleotide	5/482	85/8662	0.515381	1	0.967742	51727/132	5
hsa00010	hsa00010	Glycolysis	4/482	67/8662	0.516615	1	0.967742	1737/5230	4
hsa05221	hsa05221	Acute mye	4/482	67/8662	0.516615	1	0.967742	595/1147/	4
hsa00030	hsa00030	Pentose ph	2/482	31/8662	0.521242	1	0.967742	2539/5226	2
hsa00330	hsa00330	Arginine ar	3/482	50/8662	0.532199	1	0.967742	5033/224/	3
hsa05144	hsa05144	Malaria	3/482	50/8662	0.532199	1	0.967742	7042/3043	3
hsa04260	hsa04260	Cardiac mu	5/482	87/8662	0.536305	1	0.967742	3270/444/	5
hsa04550	hsa04550	Signaling p	8/482	143/8662	0.546004	1	0.967742	8324/3977	8
hsa05204	hsa05204	Chemical c	4/482	70/8662	0.551635	1	0.967742	1545/4257	4
hsa05230	hsa05230	Central car	4/482	70/8662	0.551635	1	0.967742	2539/5154	4
hsa01210	hsa01210	2-Oxocarb	2/482	33/8662	0.555337	1	0.967742	50/1737	2
hsa00270	hsa00270	Cysteine ar	3/482	52/8662	0.559087	1	0.967742	2730/2729	3
hsa05206	hsa05206	MicroRNAs	17/482	310/8662	0.561686	1	0.967742	595/7042/	17
hsa04622	hsa04622	RIG-I-like	4/482	71/8662	0.563008	1	0.967742	80143/114	4
[illegible]	[illegible]	[illegible]	[illegible]	[illegible]	[illegible]	[illegible]	[illegible]	[illegible]	[illegible]

GO:004332	BP	GO:004332	positive re	2/1035	4/18614	0.01719	0.413448	0.396332	LAMP1/HL	2
GO:004362	BP	GO:004362	cortical mi	2/1035	4/18614	0.01719	0.413448	0.396332	EZR/PAFAH	2
GO:004500	BP	GO:004500	depurinatio	2/1035	4/18614	0.01719	0.413448	0.396332	MPG/MUTY	2
GO:004533	BP	GO:004533	farnesyl dip	2/1035	4/18614	0.01719	0.413448	0.396332	HMGCS1/F	2
GO:006031	BP	GO:006031	definitive e	2/1035	4/18614	0.01719	0.413448	0.396332	TGFBR3/ZF	2
GO:007038	BP	GO:007038	Harderian g	2/1035	4/18614	0.01719	0.413448	0.396332	SOX9/ALD	2
GO:007228	BP	GO:007228	metanephr	2/1035	4/18614	0.01719	0.413448	0.396332	PAX8/SOX9	2
GO:009050	BP	GO:009050	endocardia	2/1035	4/18614	0.01719	0.413448	0.396332	HAS2/EFN	2
GO:009725	BP	GO:009725	oligodendr	2/1035	4/18614	0.01719	0.413448	0.396332	GAS6/AKA	2
GO:190168	BP	GO:190168	glutathione	2/1035	4/18614	0.01719	0.413448	0.396332	GSTP1/GST	2
GO:190168	BP	GO:190168	glutathione	2/1035	4/18614	0.01719	0.413448	0.396332	GSTP1/GST	2
GO:190355	BP	GO:190355	positive re	2/1035	4/18614	0.01719	0.413448	0.396332	SDC4/PDC	2
GO:190362	BP	GO:190362	negative re	2/1035	4/18614	0.01719	0.413448	0.396332	RGN/DFFA	2
GO:190377	BP	GO:190377	regulation	2/1035	4/18614	0.01719	0.413448	0.396332	VPS37B/TS	2
GO:190568	BP	GO:190568	positive re	2/1035	4/18614	0.01719	0.413448	0.396332	ANXA2/AH	2
GO:019873	BP	GO:019873	cell-cell sig	37/1035	463/18614	0.0172	0.413448	0.396332	APCDD1/T	37
GO:004426	BP	GO:004426	cellular pol	11/1035	96/18614	0.017464	0.416938	0.399677	UGP2/HAS	11
GO:190331	BP	GO:190331	negative re	11/1035	96/18614	0.017464	0.416938	0.399677	RBM10/SR	11
GO:005156	BP	GO:005156	regulation	5/1035	28/18614	0.01778	0.420186	0.402791	AUTS2/KA	5
GO:005164	BP	GO:005164	nucleus loc	5/1035	28/18614	0.01778	0.420186	0.402791	DCTN1/PA	5
GO:006021	BP	GO:006021	camera-ty	5/1035	28/18614	0.01778	0.420186	0.402791	SOX9/SDK	5
GO:001056	BP	GO:001056	regulation	14/1035	135/18614	0.018114	0.423369	0.405842	ACADL/NR	14
GO:003386	BP	GO:003386	nucleoside	13/1035	122/18614	0.018156	0.423369	0.405842	PPT1/HMG	13
GO:003387	BP	GO:003387	ribonucleo	13/1035	122/18614	0.018156	0.423369	0.405842	PPT1/HMG	13
GO:003403	BP	GO:003403	purine nucl	13/1035	122/18614	0.018156	0.423369	0.405842	PPT1/HMG	13
GO:002261	BP	GO:002261	ribonucleo	21/1035	231/18614	0.018536	0.429523	0.411742	PRPF19/SN	21
GO:000649	BP	GO:000649	protein lipi	11/1035	97/18614	0.018743	0.429523	0.411742	PIGT/ZDHH	11
GO:001073	BP	GO:001073	regulation	4/1035	19/18614	0.018848	0.429523	0.411742	ADRB2/MC	4
GO:004277	BP	GO:004277	DNA dama	4/1035	19/18614	0.018848	0.429523	0.411742	MUC1/KAT	4
GO:004362	BP	GO:004362	ncRNA 3'-	4/1035	19/18614	0.018848	0.429523	0.411742	FBL/EXOSC	4
GO:004398	BP	GO:004398	histone H4	4/1035	19/18614	0.018848	0.429523	0.411742	AUTS2/KA	4
GO:200064	BP	GO:200064	regulation	4/1035	19/18614	0.018848	0.429523	0.411742	MTMR2/M	4
GO:005165	BP	GO:005165	spindle loc	8/1035	61/18614	0.019159	0.433784	0.415826	NSFL1C/D	8
GO:190002	BP	GO:190002	regulation	8/1035	61/18614	0.019159	0.433784	0.415826	ST6GAL1/K	8
GO:001993	BP	GO:001993	cyclic-nucl	10/1035	85/18614	0.019304	0.433784	0.415826	POMC/KCN	10
GO:007252	BP	GO:007252	pyridine-c	10/1035	85/18614	0.019304	0.433784	0.415826	GPD1L/DC	10
GO:000636	BP	GO:000636	rRNA proc	20/1035	218/18614	0.019402	0.433784	0.415826	RCL1/NIFK	20
GO:000726	BP	GO:000726	Ras protei	28/1035	333/18614	0.019493	0.433784	0.415826	ARHGAP17	28
GO:190237	BP	GO:190237	negative re	9/1035	73/18614	0.01951	0.433784	0.415826	RBM10/RB	9
GO:190516	BP	GO:190516	positive re	6/1035	39/18614	0.019715	0.433784	0.415826	POGZ/RUV	6
GO:000652	BP	GO:000652	cellular am	25/1035	290/18614	0.020001	0.433784	0.415826	GSTZ1/ASP	25
GO:004426	BP	GO:004426	cellular car	25/1035	290/18614	0.020001	0.433784	0.415826	INPP1/UGP	25
GO:001073	BP	GO:001073	positive re	3/1035	11/18614	0.020209	0.433784	0.415826	ADRB2/MC	3
GO:001593	BP	GO:001593	coenzyme	3/1035	11/18614	0.020209	0.433784	0.415826	ACOT7/AC	3
GO:001610	BP	GO:001610	diterpenoi	3/1035	11/18614	0.020209	0.433784	0.415826	ALDH8A1/	3
GO:003194	BP	GO:003194	regulation	3/1035	11/18614	0.020209	0.433784	0.415826	NR3C1/DK	3
GO:006073	BP	GO:006073	prostate gl	3/1035	11/18614	0.020209	0.433784	0.415826	AR/SOX9/	3
GO:007047	BP	GO:007047	uterine sm	3/1035	11/18614	0.020209	0.433784	0.415826	OXT/ABAT	3
GO:007269	BP	GO:007269	protein loc	3/1035	11/18614	0.020209	0.433784	0.415826	EZR/NUM	3
GO:007552	BP	GO:007552	IRES-depe	3/1035	11/18614	0.020209	0.433784	0.415826	EIF3D/EIF3	3
GO:200039	BP	GO:200039	regulation	3/1035	11/18614	0.020209	0.433784	0.415826	KANK1/CC	3
GO:000761	BP	GO:000761	learning or	24/1035	276/18614	0.02034	0.434259	0.416281	NRXN3/PP	24
GO:000726	BP	GO:000726	nitric oxide	5/1035	29/18614	0.020527	0.434259	0.416281	RASD1/KC	5
GO:001907	BP	GO:001907	viral releas	5/1035	29/18614	0.020527	0.434259	0.416281	VPS37B/PF	5
GO:003589	BP	GO:003589	exit from h	5/1035	29/18614	0.020527	0.434259	0.416281	VPS37B/PF	5
GO:004308	BP	GO:004308	regulation	30/1035	364/18614	0.020541	0.434259	0.416281	TIAM2/ARH	30
GO:007200	BP	GO:007200	renal syste	27/1035	320/18614	0.020669	0.43439	0.416407	HAS2/CNT	27
GO:004206	BP	GO:004206	wound hea	35/1035	439/18614	0.020708	0.43439	0.416407	SERPINA1/	35
GO:004325	BP	GO:004325	regulation	33/1035	409/18614	0.020743	0.43439	0.416407	ARHGEF5/	33
GO:000697	BP	GO:000697	response t	10/1035	86/18614	0.020794	0.43439	0.416407	PAPPA2/O	10
GO:004653	BP	GO:004653	photorecep	8/1035	62/18614	0.020961	0.436579	0.418505	SOX9/PAX	8
GO:000924	BP	GO:000924	glycolipid b	9/1035	74/18614	0.021154	0.439294	0.421107	PIGT/PIGK	9
GO:000609	BP	GO:000609	pyruvate m	12/1035	112/18614	0.021796	0.449489	0.430881	JMJD8/OG	12
GO:003086	BP	GO:003086	cortical act	6/1035	40/18614	0.022136	0.449489	0.430881	EZR/PLEC/	6
GO:009018	BP	GO:009018	regulation	6/1035	40/18614	0.022136	0.449489	0.430881	ACADL/FM	6
GO:001633	BP	GO:001633	morphoge	15/1035	152/18614	0.022235	0.449489	0.430881	COBL/TCTE	15
GO:009050	BP	GO:009050	RNA phosp	10/1035	87/18614	0.022367	0.449489	0.430881	RCL1/RRS1	10
GO:000652	BP	GO:000652	arginine m	4/1035	20/18614	0.022547	0.449489	0.430881	ASL/DDAH	4
GO:003221	BP	GO:003221	negative re	4/1035	20/18614	0.022547	0.449489	0.430881	PINX1/EXO	4
GO:003953	BP	GO:003953	regulation	4/1035	20/18614	0.022547	0.449489	0.430881	ZCCHC3/P	4
GO:190022	BP	GO:190022	positive re	4/1035	20/18614	0.022547	0.449489	0.430881	PRKD1/PL	4
GO:004354	BP	GO:004354	protein acy	22/1035	250/18614	0.022651	0.449489	0.430881	MUC1/NA	22
GO:000740	BP	GO:000740	axonogene	34/1035	427/18614	0.022727	0.449489	0.430881	TIAM2/CO	34
GO:000209	BP	GO:000209	regulation	8/1035	63/18614	0.022881	0.449489	0.430881	MTMR2/PL	8
GO:004825	BP	GO:004825	mRNA stab	8/1035	63/18614	0.022881	0.449489	0.430881	RBM10/RB	8
GO:000597	BP	GO:000597	glycogen m	9/1035	75/18614	0.022896	0.449489	0.430881	UGP2/POM	9
GO:003096	BP	GO:003096	endoplasm	9/1035	75/18614	0.022896	0.449489	0.430881	EIF2S1/BO	9
GO:003463	BP	GO:003463	cellular car	9/1035	75/18614	0.022896	0.449489	0.430881	UGP2/HAS	9
GO:003136	BP	GO:003136	N-termina	5/1035	30/18614	0.023537	0.449489	0.430881	NAA30/ME	5
GO:005122	BP	GO:005122	positive re	26/1035	309/18614	0.023541	0.449489	0.430881	ARHGEF5/	26
GO:003530	BP	GO:003530	regulation	10/1035	88/18614	0.024024	0.449489	0.430881	JAK2/MAG	10
GO:000916	BP	GO:000916	nucleotide	25/1035	295/18614	0.024081	0.449489	0.430881	PPT1/NME	25
GO:001993	BP	GO:001993	cAMP-med	7/1035	52/18614	0.024235	0.449489	0.430881	POMC/SOX	7
GO:003134	BP	GO:003134	negative re	18/1035	195/18614	0.024259	0.449489	0.430881	PTPRG/KA	18
GO:000182	BP	GO:000182	kidney dev	26/1035	310/18614	0.024391	0.449489	0.430881	HAS2/CNT	26
GO:000607	BP	GO:000607	cellular glu	9/1035	76/18614	0.024737	0.449489	0.430881	UGP2/POM	9
GO:004404	BP	GO:004404	glucan met	9/1035	76/18614	0.024737	0.449489	0.430881	UGP2/POM	9
GO:003305	BP	GO:003305	cellular pig	8/1035	64/18614	0.024921	0.449489	0.430881	SHROOM3	8
GO:005156	BP	GO:005156	histone H3	8/1035	64/18614	0.024921	0.449489	0.430881	SETD4/AU	8
GO:000647	BP	GO:000647	protein me	17/1035	182/18614	0.025319	0.449489	0.430881	SETD4/SM	17
GO:000821	BP	GO:000821	protein alk	17/1035	182/18614	0.025319	0.449489	0.430881	SETD4/SM	17
GO:000965	BP	GO:000965	UV protect	3/1035	12/18614	0.025851	0.449489	0.430881	SDF4/MC1	3
GO:001405	BP	GO:001405	gamma-ar	3/1035	12/18614	0.025851	0.449489	0.430881	ABAT/GAB	3
GO:001932	BP	GO:001932	pentose m	3/1035	12/18614	0.025851	0.449489	0.430881	DCXR/UXS	3
GO:004347	BP	GO:004347	pigment ac	3/1035	12/18614	0.025851	0.449489	0.430881	SHROOM3	3
GO:004348	BP	GO:004348	cellular pig	3/1035	12/18614	0.025851	0.449489	0.430881	SHROOM3	3
GO:005134	BP	GO:005134	regulation	3/1035	12/18614	0.025851	0.449489	0.430881	RGN/XRCC	3
GO:006022	BP	GO:006022	retinal rod	3/1035	12/18614	0.025851	0.449489	0.430881	SOX9/RPG	3
GO:006083	BP	GO:006083	smoothene	3/1035	12/18614	0.025851	0.449489	0.430881	GPR161/SU	3
GO:007085	BP	GO:007085	regulation	3/1035	12/18614	0.025851	0.449489	0.430881	NR1D1/MA	3
GO:007139	BP	GO:007139	cellular res	3/1035	12/18614	0.025851	0.449489	0.430881	AR/MSN/N	3
GO:007249	BP	GO:007249	mesenchyr	3/1035	12/18614	0.025851	0.449489	0.430881	SOX9/SOX	3
GO:190294	BP	GO:190294	protein loc	3/1035	12/18614	0.025851	0.449489	0.430881	MSN/RDX/	3
GO:190433	BP	GO:190433	regulation	3/1035	12/18614	0.025851	0.449489	0.430881	CSNK1D/T	3
GO:190566	BP	GO:190566	positive re	3/1035	12/18614	0.025851	0.449489	0.430881	MSN/RDX/	3
GO:190129	BP	GO:190129	nucleoside	25/1035	297/18614	0.025882	0.449489	0.430881	PPT1/NME	25
GO:000616	BP	GO:000616	nucleoside	11/1035	102/18614	0.0262	0.449489	0.430881	JMJD8/NM	11
GO:004215	BP	GO:004215	lipoprotein	11/1035	102/18614	0.0262	0.449489	0.430881	PIGT/ZDHH	11
GO:004247	BP	GO:004247	inner ear m	11/1035	102/18614	0.0262	0.449489	0.430881	SLC44A4/F	11
GO:004825	BP	GO:004825	regulation	12/1035	115/18614	0.026201	0.449489	0.430881	PPT1/MTM	12
GO:003238	BP	GO:003238	positive re	18/1035	197/18614	0.026539	0.449489	0.430881	PRKD1/JAK	18
GO:001607	BP	GO:001607	rRNA catal	4/1035	21/18614	0.026658	0.449489	0.430881	EXOSC10/	4
GO:003233	BP	GO:003233	positive re	4/1035	21/18614	0.026658	0.449489	0.430881	LOXL2/SOX	4
GO:003580	BP	GO:003580	regulation	4/1035	21/18614	0.026658	0.449489	0.430881	OXT/HAS2	4
GO:005157	BP	GO:005157	positive re	4/1035	21/18614	0.026658	0.449489	0.430881	AUTS2/KA	4
GO:003313	BP	GO:003313	negative re	5/1035	31/18614	0.026818	0.449489	0.430881	GPD1L/PW	5
GO:003625	BP	GO:003625	multivesicu	5/1035	31/18614	0.026818	0.449489	0.430881	VPS37B/VF	5
GO:007029	BP	GO:007029	renal absor	5/1035	31/18614	0.026818	0.449489	0.430881	HAS2/KLHI	5
GO:190022	BP	GO:190022	regulation	5/1035	31/18614	0.026818	0.449489	0.430881	PRKD1/PL	5
GO:001922	BP	GO:001922	transmissio	8/1035	65/18614	0.027084	0.449489	0.430881	ANK3/GRI	8
GO:003250	BP	GO:003250	maintenan	8/1035	65/18614	0.027084	0.449489	0.430881	ANK3/RIT2	8
GO:190533	BP	GO:190533	regulation	8/1035	65/18614	0.027084	0.449489	0.430881	AR/PHB2/F	8
GO:006156	BP	GO:006156	axon devel	37/1035	479/18614	0.027318	0.449489	0.430881	TIAM2/CO	37
GO:000606	BP	GO:000606	glucuronat	2/1035	5/18614	0.027601	0.449489	0.430881	DCXR/CRY	2
GO:000652	BP	GO:000652	asparagine	2/1035	5/18614	0.027601	0.449489	0.430881	ASRGL1/N	2
GO:000656	BP	GO:000656	threonine r	2/1035	5/18614	0.027601	0.449489	0.430881	GCAT/PHG	2
GO:000735	BP	GO:000735	thorax and	2/1035	5/18614	0.027601	0.449489	0.430881	TIFAB/DCA	2
GO:000821	BP	GO:000821	spermine r	2/1035	5/18614	0.027601	0.449489	0.430881	SMOX/AM	2
GO:001619	BP	GO:001619	axon midli	2/1035	5/18614	0.027601	0.449489	0.430881	ROBO3/RO	2
GO:001805	BP	GO:001805	peptidyl-ly	2/1035	5/18614	0.027601	0.449489	0.430881	LOXL2/LO	2
GO:001964	BP	GO:001964	glucuronat	2/1035	5/18614	0.027601	0.449489	0.430881	DCXR/CRY	2
GO:002165	BP	GO:002165	vestibuloc	2/1035	5/18614	0.027601	0.449489	0.430881	TIFAB/DCA	2
GO:002178	BP	GO:002178	glial cell fa	2/1035	5/18614	0.027601	0.449489	0.430881	SOX9/PAX	2
GO:002181	BP	GO:002181	modulatio	2/1035	5/18614	0.027601	0.449489	0.430881	SUN2/SYN	2
GO:002261	BP	GO:002261	membrane	2/1035	5/18614	0.027601	0.449489	0.430881	MSN/EZR	2
GO:003047	BP	GO:003047	nuclear mi	2/1035	5/18614	0.027601	0.449489	0.430881	SUN2/SYN	2
GO:003247	BP	GO:003247	otolith mo	2/1035	5/18614	0.027601	0.449489	0.430881	SLC44A4/T	2
GO:003360	BP	GO:003360	negative re	2/1035	5/18614	0.027601	0.449489	0.430881	ABAT/GAB	2
GO:003581	BP	GO:003581	negative re	2/1035	5/18614	0.027601	0.449489	0.430881	OXT/MLLT	2
GO:003593	BP	GO:003593	corticoster	2/1035	5/18614	0.027601	0.449489	0.430881	POMC/TSP	2
GO:005116	BP	GO:005116	xylulose 5-	2/1035	5/18614	0.027601	0.449489	0.430881	DCXR/CRY	2
GO:005117	BP	GO:005117	positive re	2/1035	5/18614	0.027601	0.449489	0.430881	ABCB7/SP	2
GO:006181	BP	GO:006181	endoplasm	2/1035	5/18614	0.027601	0.449489	0.430881	ESYT3/ESY	2
GO:006188	BP	GO:006188	negative re	2/1035	5/18614	0.027601	0.449489	0.430881	NR1D1/LD	2
GO:007029	BP	GO:007029	renal wate	2/1035	5/18614	0.027601	0.449489	0.430881	HAS2/MLL	2
GO:007552	BP	GO:007552	viral transl	2/1035	5/18614	0.027601	0.449489	0.430881	EIF3D/EIF3	2
GO:010600	BP	GO:010600	intestinal h	2/1035	5/18614	0.027601	0.449489	0.430881	EZR/PLS1	2
GO:190082	BP	GO:190082	regulation	2/1035	5/18614	0.027601	0.449489	0.430881	ANK3/RAN	2
GO:190115	BP	GO:190115	xylulose 5-	2/1035	5/18614	0.027601	0.449489	0.430881	DCXR/CRY	2
GO:190246	BP	GO:190246	positive re	2/1035	5/18614	0.027601	0.449489	0.430881	LTBP3/CITI	2
GO:190434	BP	GO:190434	positive re	2/1035	5/18614	0.027601	0.449489	0.430881	CSNK1D/D	2
GO:190450	BP	GO:190450	regulation	2/1035	5/18614	0.027601	0.449489	0.430881	ADRB2/SP	2
GO:190450	BP	GO:190450	positive re	2/1035	5/18614	0.027601	0.449489	0.430881	ADRB2/SP	2
GO:190495	BP	GO:190495	regulation	2/1035	5/18614	0.027601	0.449489	0.430881	CSNK1D/D	2
GO:200037	BP	GO:200037	positive re	2/1035	5/18614	0.027601	0.449489	0.430881	DNM2/DG	2
GO:001097	BP	GO:001097	negative re	14/1035	143/18614	0.028204	0.45089	0.432223	PTPRG/KA	14
GO:001820	BP	GO:001820	peptidyl-ly	30/1035	374/18614	0.028365	0.45089	0.432223	MUC1/SET	30
GO:000166	BP	GO:000166	ameboidal	38/1035	496/18614	0.028566	0.45089	0.432223	HYAL1/PTP	38
GO:000689	BP	GO:000689	receptor-n	22/1035	256/18614	0.028637	0.45089	0.432223	PPT1/ADRI	22
GO:004400	BP	GO:004400	movement	17/1035	185/18614	0.029063	0.45089	0.432223	VAMP8/LA	17
GO:003233	BP	GO:003233	regulation	7/1035	54/18614	0.029181	0.45089	0.432223	LOXL2/SOX	7
GO:190274	BP	GO:190274	regulation	7/1035	54/18614	0.029181	0.45089	0.432223	KANK1/AU	7
GO:009063	BP	GO:009063	activation	12/1035	117/18614	0.029478	0.45089	0.432223	TIAM2/ARI	12
GO:003090	BP	GO:003090	midbrain d	10/1035	91/18614	0.029523	0.45089	0.432223	CKB/CSNK	10
GO:006099	BP	GO:006099	dendritic s	10/1035	91/18614	0.029523	0.45089	0.432223	ASAP1/ARI	10
GO:007238	BP	GO:007238	organelle t	10/1035	91/18614	0.029523	0.45089	0.432223	LAMP1/SYI	10
GO:001931	BP	GO:001931	hexose me	20/1035	228/18614	0.029611	0.45089	0.432223	GALE/CHS	20
GO:003220	BP	GO:003220	regulation	11/1035	104/18614	0.029713	0.45089	0.432223	MAP3K4/P	11
GO:003010	BP	GO:003010	regulation	19/1035	214/18614	0.029985	0.45089	0.432223	PPT1/PRKD	19
GO:000608	BP	GO:000608	acetyl-CoA	5/1035	32/18614	0.030376	0.45089	0.432223	HMGCS1/F	5
GO:001076	BP	GO:001076	positive re	5/1035	32/18614	0.030376	0.45089	0.432223	GPD1L/AN	5
GO:003625	BP	GO:003625	multivesicu	5/1035	32/18614	0.030376	0.45089	0.432223	VPS37B/VF	5
GO:005164	BP	GO:005164	centrosom	5/1035	32/18614	0.030376	0.45089	0.432223	EZR/PAFAH	5
GO:006184	BP	GO:006184	microtubul	5/1035	32/18614	0.030376	0.45089	0.432223	EZR/PAFAH	5
GO:000915	BP	GO:000915	purine ribo	6/1035	43/18614	0.030579	0.45089	0.432223	ACOT7/NU	6
GO:001967	BP	GO:001967	NAD meta	6/1035	43/18614	0.030579	0.45089	0.432223	GPD1L/OG	6
GO:007176	BP	GO:007176	nuclear me	6/1035	43/18614	0.030579	0.45089	0.432223	NSFL1C/VF	6
GO:005089	BP	GO:005089	cognition	26/1035	317/18614	0.031026	0.45089	0.432223	NRXN3/PP	26
GO:000222	BP	GO:000222	stimulatory	4/1035	22/18614	0.031189	0.45089	0.432223	PLCG2/TR	4
GO:000753	BP	GO:000753	sex determ	4/1035	22/18614	0.031189	0.45089	0.432223	MAP3K4/A	4
GO:199084	BP	GO:199084	response t	4/1035	22/18614	0.031189	0.45089	0.432223	PLCG2/TR	4
GO:199085	BP	GO:199085	cellular res	4/1035	22/18614	0.031189	0.45089	0.432223	PLCG2/TR	4
GO:004364	BP	GO:004364	inositol ph	7/1035	55/18614	0.031892	0.45089	0.432223	INPP1/ITPK	7
GO:004593	BP	GO:004593	negative re	34/1035	439/18614	0.032218	0.45089	0.432223	CTDSP2/GI	34
GO:003253	BP	GO:003253	regulation	3/1035	13/18614	0.032246	0.45089	0.432223	EZR/PLS1/	3
GO:003533	BP	GO:003533	negative re	3/1035	13/18614	0.032246	0.45089	0.432223	WWC3/DL	3
GO:007180	BP	GO:007180	regulation	3/1035	13/18614	0.032246	0.45089	0.432223	ARHGEF5/	3
GO:009023	BP	GO:009023	regulation	3/1035	13/18614	0.032246	0.45089	0.432223	KAT5/NUM	3
GO:009869	BP	GO:009869	regulation	3/1035	13/18614	0.032246	0.45089	0.432223	SCRN1/PLD	3
GO:190009	BP	GO:190009	regulation	3/1035	13/18614	0.032246	0.45089	0.432223	KANSL3/O	3
GO:190026	BP	GO:190026	regulation	3/1035	13/18614	0.032246	0.45089	0.432223	GINS2/RFC	3
GO:190026	BP	GO:190026	positive re	3/1035	13/18614	0.032246	0.45089	0.432223	GINS2/RFC	3
GO:190305	BP	GO:190305	negative re	3/1035	13/18614	0.032246	0.45089	0.432223	ADTRP/CH	3
GO:190362	BP	GO:190362	regulation	3/1035	13/18614	0.032246	0.45089	0.432223	RGN/CIDE	3
GO:190566	BP	GO:190566	regulation	3/1035	13/18614	0.032246	0.45089	0.432223	MSN/RDX/	3
GO:002260	BP	GO:002260	regulation	21/1035	245/18614	0.032726	0.45089	0.432223	KANK1/AR	21
GO:190353	BP	GO:190353	positive re	24/1035	289/18614	0.03279	0.45089	0.432223	VAMP8/OX	24
GO:000727	BP	GO:000727	ensheathm	14/1035	146/18614	0.032891	0.45089	0.432223	MTMR2/CD	14
GO:000836	BP	GO:000836	axon enshe	14/1035	146/18614	0.032891	0.45089	0.432223	MTMR2/CD	14
GO:007207	BP	GO:007207	kidney epit	14/1035	146/18614	0.032891	0.45089	0.432223	MAGI2/FO	14
GO:003103	BP	GO:003103	actomyosir	18/1035	202/18614	0.03294	0.45089	0.432223	ARHGEF5/	18
GO:001056	BP	GO:001056	negative re	34/1035	440/18614	0.033129	0.45089	0.432223	CTDSP2/GI	34
GO:000640	BP	GO:000640	RNA catab	26/1035	319/18614	0.033153	0.45089	0.432223	RBM10/RB	26
GO:004592	BP	GO:004592	positive re	9/1035	80/18614	0.033155	0.45089	0.432223	VAMP8/LA	9
GO:006056	BP	GO:006056	epithelial t	27/1035	334/18614	0.033219	0.45089	0.432223	COBL/TCTI	27
GO:000926	BP	GO:000926	ribonucleo	20/1035	231/18614	0.033348	0.45089	0.432223	PPT1/NME	20
GO:000913	BP	GO:000913	purine nucl	11/1035	106/18614	0.033551	0.45089	0.432223	JMJD8/OG	11
GO:000917	BP	GO:000917	purine ribo	11/1035	106/18614	0.033551	0.45089	0.432223	JMJD8/OG	11
GO:002176	BP	GO:002176	substantia	6/1035	44/18614	0.033803	0.45089	0.432223	CKB/SYNG	6
GO:003288	BP	GO:003288	regulation	6/1035	44/18614	0.033803	0.45089	0.432223	HAS2/POM	6
GO:007253	BP	GO:007253	T-helper 1	6/1035	44/18614	0.033803	0.45089	0.432223	JAK2/NFKE	6
GO:004643	BP	GO:004643	organopho	15/1035	161/18614	0.034835	0.45089	0.432223	INPP1/GPD	15
GO:005125	BP	GO:005125	protein pol	23/1035	276/18614	0.03486	0.45089	0.432223	COBL/KAN	23
GO:000666	BP	GO:000666	glycolipid r	11/1035	107/18614	0.035596	0.45089	0.432223	SUMF1/PIG	11
GO:000913	BP	GO:000913	nucleoside	13/1035	134/18614	0.035819	0.45089	0.432223	JMJD8/NM	13
GO:000024	BP	GO:000024	spliceosom	10/1035	94/18614	0.035859	0.45089	0.432223	PRPF19/SN	10
GO:001086	BP	GO:001086	regulation	4/1035	23/18614	0.036143	0.45089	0.432223	LDLR/RGN	4
GO:001089	BP	GO:001089	positive re	4/1035	23/18614	0.036143	0.45089	0.432223	NR1D1/FG	4
GO:003970	BP	GO:003970	viral buddi	4/1035	23/18614	0.036143	0.45089	0.432223	VPS37B/VF	4
GO:004396	BP	GO:004396	histone H2	4/1035	23/18614	0.036143	0.45089	0.432223	RUVBL2/KA	4
GO:004559	BP	GO:004559	positive re	4/1035	23/18614	0.036143	0.45089	0.432223	FOXO3/KA	4
GO:004605	BP	GO:004605	cAMP meta	4/1035	23/18614	0.036143	0.45089	0.432223	ADCY3/AD	4
GO:004671	BP	GO:004671	muscle cell	4/1035	23/18614	0.036143	0.45089	0.432223	CAPN3/CF	4
GO:004816	BP	GO:004816	regulation	4/1035	23/18614	0.036143	0.45089	0.432223	GRIK2/AGT	4
GO:005149	BP	GO:005149	positive re	17/1035	190/18614	0.036197	0.45089	0.432223	ARHGEF5/	17
GO:001605	BP	GO:001605	carbohydra	15/1035	162/18614	0.036501	0.45089	0.432223	GALE/JMJD	15
GO:003004	BP	GO:003004	actin filame	15/1035	162/18614	0.036501	0.45089	0.432223	COBL/KAN	15
GO:004346	BP	GO:004346	regulation	13/1035	135/18614	0.037705	0.45089	0.432223	JMJD8/POM	13
GO:190350	BP	GO:190350	liposaccha	11/1035	108/18614	0.037727	0.45089	0.432223	SUMF1/PIG	11
GO:001607	BP	GO:001607	snRNA me	7/1035	57/18614	0.037808	0.45089	0.432223	INTS10/EX	7
GO:003106	BP	GO:003106	regulation	7/1035	57/18614	0.037808	0.45089	0.432223	AUTS2/KA	7
GO:004347	BP	GO:004347	regulation	7/1035	57/18614	0.037808	0.45089	0.432223	JMJD8/DDI	7
GO:004454	BP	GO:004454	NLRP3 infla	5/1035	34/18614	0.038345	0.45089	0.432223	PRKD1/PL	5
GO:006031	BP	GO:006031	cardiac epi	5/1035	34/18614	0.038345	0.45089	0.432223	HAS2/TGFI	5
GO:011011	BP	GO:011011	positive re	5/1035	34/18614	0.038345	0.45089	0.432223	AR/FGF1/S	5
GO:190550	BP	GO:190550	protein loc	5/1035	34/18614	0.038345	0.45089	0.432223	CSNK1D/N	5
GO:005170	BP	GO:005170	biological p	18/1035	206/18614	0.038832	0.45089	0.432223	VAMP8/LA	18
GO:000815	BP	GO:000815	actin polyn	17/1035	192/18614	0.039381	0.45089	0.432223	COBL/KAN	17
GO:000616	BP	GO:000616	AMP biosy	3/1035	14/18614	0.039385	0.45089	0.432223	PAICS/PFA	3
GO:000717	BP	GO:000717	negative re	3/1035	14/18614	0.039385	0.45089	0.432223	CBLC/GPR	3
GO:001815	BP	GO:001815	protein oxi	3/1035	14/18614	0.039385	0.45089	0.432223	SUMF1/LO	3
GO:003195	BP	GO:003195	corticoster	3/1035	14/18614	0.039385	0.45089	0.432223	JAK2/NR3C	3
GO:003411	BP	GO:003411	negative re	3/1035	14/18614	0.039385	0.45089	0.432223	CD9/RDX/	3
GO:004213	BP	GO:004213	neurotrans	3/1035	14/18614	0.039385	0.45089	0.432223	SLC44A4/N	3
GO:005112	BP	GO:005112	positive re	3/1035	14/18614	0.039385	0.45089	0.432223	WASF3/GS	3
GO:190102	BP	GO:190102	negative re	3/1035	14/18614	0.039385	0.45089	0.432223	SLC25A6/E	3
GO:190107	BP	GO:190107	regulation	3/1035	14/18614	0.039385	0.45089	0.432223	SRI/TIFAB/	3
GO:190302	BP	GO:190302	regulation	3/1035	14/18614	0.039385	0.45089	0.432223	HAND1/ZN	3
GO:000836	BP	GO:000836	regulation	16/1035	178/18614	0.039786	0.45089	0.432223	RAP1GAP2	16
GO:004362	BP	GO:004362	response t	8/1035	70/18614	0.039863	0.45089	0.432223	CKB/AR/CI	8
GO:000618	BP	GO:000618	'de novo' I	2/1035	6/18614	0.039891	0.45089	0.432223	PAICS/PFA	2
GO:000633	BP	GO:000633	DNA replic	2/1035	6/18614	0.039891	0.45089	0.432223	CHAF1B/C	2
GO:000735	BP	GO:000735	zygotic de	2/1035	6/18614	0.039891	0.45089	0.432223	TIFAB/DCA	2
GO:000918	BP	GO:000918	deoxyribor	2/1035	6/18614	0.039891	0.45089	0.432223	NUDT18/G	2
GO:001013	BP	GO:001013	pyrimidine	2/1035	6/18614	0.039891	0.45089	0.432223	CDA/UCK2	2
GO:001625	BP	GO:001625	attachmen	2/1035	6/18614	0.039891	0.45089	0.432223	PIGT/PIGK	2
GO:002190	BP	GO:002190	commitme	2/1035	6/18614	0.039891	0.45089	0.432223	TBR1/PAX6	2
GO:003057	BP	GO:003057	ubiquitin-c	2/1035	6/18614	0.039891	0.45089	0.432223	RNF111/ST	2
GO:003091	BP	GO:003091	paranodal	2/1035	6/18614	0.039891	0.45089	0.432223	CD9/UGT8	2
GO:003104	BP	GO:003104	heterochro	2/1035	6/18614	0.039891	0.45089	0.432223	FAM172A/	2
GO:003158	BP	GO:003158	activation	2/1035	6/18614	0.039891	0.45089	0.432223	PRKCZ/GN	2
GO:003226	BP	GO:003226	pyrimidine	2/1035	6/18614	0.039891	0.45089	0.432223	CDA/UCK2	2
GO:004227	BP	GO:004227	protection	2/1035	6/18614	0.039891	0.45089	0.432223	HLA-B/HL	2
GO:004341	BP	GO:004341	positive re	2/1035	6/18614	0.039891	0.45089	0.432223	CAPN3/PP	2
GO:004420	BP	GO:004420	UMP salva	2/1035	6/18614	0.039891	0.45089	0.432223	CDA/UCK2	2
GO:005166	BP	GO:005166	maintenan	2/1035	6/18614	0.039891	0.45089	0.432223	PAFAH1B1	2
GO:005505	BP	GO:005505	asymmetri	2/1035	6/18614	0.039891	0.45089	0.432223	FGF13/DO	2
GO:006017	BP	GO:006017	male matir	2/1035	6/18614	0.039891	0.45089	0.432223	OXT/NHLH	2
GO:006030	BP	GO:006030	regulation	2/1035	6/18614	0.039891	0.45089	0.432223	MTMR2/M	2
GO:006037	BP	GO:006037	mast cell d	2/1035	6/18614	0.039891	0.45089	0.432223	ZFPM1/PIK	2
GO:006051	BP	GO:006051	type II pne	2/1035	6/18614	0.039891	0.45089	0.432223	NFIB/AIMP	2
GO:006203	BP	GO:006203	positive re	2/1035	6/18614	0.039891	0.45089	0.432223	KAT5/NUM	2
GO:007008	BP	GO:007008	ubiquitin-c	2/1035	6/18614	0.039891	0.45089	0.432223	VPS28/TSG	2
GO:007113	BP	GO:007113	resolution	2/1035	6/18614	0.039891	0.45089	0.432223	RMI2/BLM	2
GO:007217	BP	GO:007217	metanephr	2/1035	6/18614	0.039891	0.45089	0.432223	PAX8/SOX	2
GO:009024	BP	GO:009024	positive re	2/1035	6/18614	0.039891	0.45089	0.432223	MUC1/AU	2
GO:009890	BP	GO:009890	regulation	2/1035	6/18614	0.039891	0.45089	0.432223	ANK3/RAN	2
GO:190216	BP	GO:190216	regulation	2/1035	6/18614	0.039891	0.45089	0.432223	COPS3/ZN	2

GO:004427	BP	GO:004427	sulfur com	15/902	155/18614	0.008373	0.20209	0.181386	DSE/DSEL/	15
GO:003009	BP	GO:003009	lymphocyt	32/902	422/18614	0.008416	0.202411	0.181675	IRF1/MAFE	32
GO:003166	BP	GO:003166	cellular res	20/902	230/18614	0.008458	0.202692	0.181927	SESN3/PM	20
GO:006103	BP	GO:006103	regulation	9/902	73/18614	0.008521	0.203094	0.182288	GREM1/SH	9
GO:004593	BP	GO:004593	negative re	33/902	439/18614	0.008536	0.203094	0.182288	GREM1/EN	33
GO:005500	BP	GO:005500	cardiac mu	13/902	127/18614	0.00867	0.204875	0.183886	GREM1/GA	13
GO:003251	BP	GO:003251	late endos	5/902	27/18614	0.008764	0.204875	0.183886	CHMP5/ST	5
GO:003629	BP	GO:003629	response t	5/902	27/18614	0.008764	0.204875	0.183886	ATP6V0A2	5
GO:006066	BP	GO:006066	embryonic	5/902	27/18614	0.008764	0.204875	0.183886	ZFP36L1/R	5
GO:007086	BP	GO:007086	regulation	5/902	27/18614	0.008764	0.204875	0.183886	SVIP/ERLE	5
GO:001056	BP	GO:001056	negative re	33/902	440/18614	0.008815	0.205266	0.184238	GREM1/EN	33
GO:005196	BP	GO:005196	negative re	15/902	156/18614	0.008862	0.205266	0.184238	DUSP10/D	15
GO:004214	BP	GO:004214	cellular res	7/902	49/18614	0.008903	0.205266	0.184238	SESN3/PM	7
GO:004685	BP	GO:004685	regulation	7/902	49/18614	0.008903	0.205266	0.184238	GREM1/TN	7
GO:004886	BP	GO:004886	stem cell d	21/902	247/18614	0.009063	0.207632	0.186361	GREM1/GA	21
GO:005189	BP	GO:005189	regulation	17/902	186/18614	0.009093	0.207632	0.186361	SESN3/PHI	17
GO:003806	BP	GO:003806	NIK/NF-ka	14/902	142/18614	0.009099	0.207632	0.186361	GREM1/LIN	14
GO:001988	BP	GO:001988	antigen pro	12/902	114/18614	0.009233	0.209963	0.188454	ERAP1/CD	12
GO:000858	BP	GO:000858	regulation	10/902	87/18614	0.009292	0.210591	0.189017	GLIS2/ARM	10
GO:006053	BP	GO:006053	muscle tiss	32/902	426/18614	0.009589	0.213118	0.191285	GREM1/GA	32
GO:000691	BP	GO:000691	nucleocyto	26/902	328/18614	0.0097	0.213118	0.191285	SUMO1/BC	26
GO:005116	BP	GO:005116	nuclear tra	26/902	328/18614	0.0097	0.213118	0.191285	SUMO1/BC	26
GO:000725	BP	GO:000725	activation	4/902	18/18614	0.009722	0.213118	0.191285	CHI3L1/TIF	4
GO:003319	BP	GO:003319	response t	4/902	18/18614	0.009722	0.213118	0.191285	GPX3/MGS	4
GO:004506	BP	GO:004506	T-helper 2	4/902	18/18614	0.009722	0.213118	0.191285	BCL3/IL4R	4
GO:004685	BP	GO:004685	negative re	4/902	18/18614	0.009722	0.213118	0.191285	GREM1/TN	4
GO:007256	BP	GO:007256	chemokine	4/902	18/18614	0.009722	0.213118	0.191285	FOXP1/TIR	4
GO:200034	BP	GO:200034	regulation	4/902	18/18614	0.009722	0.213118	0.191285	FOXP1/TIR	4
GO:200081	BP	GO:200081	negative re	4/902	18/18614	0.009722	0.213118	0.191285	NTRK2/PIK	4
GO:005086	BP	GO:005086	regulation	29/902	377/18614	0.00976	0.213246	0.1914	IRF1/ITCH/	29
GO:007266	BP	GO:007266	establishm	8/902	62/18614	0.009812	0.213694	0.191802	TNFAIP3/L	8
GO:004440	BP	GO:004440	biological	25/902	313/18614	0.010111	0.219041	0.196602	ITCH/TRIM	25
GO:001623	BP	GO:001623	positive re	9/902	75/18614	0.010123	0.219041	0.196602	SESN3/SN	9
GO:004263	BP	GO:004263	regulation	5/902	28/18614	0.010252	0.221117	0.198465	TGFB2/MY	5
GO:000830	BP	GO:000830	isoprenoid	3/902	10/18614	0.010534	0.224294	0.201316	CYP26B1/	3
GO:005140	BP	GO:005140	response t	3/902	10/18614	0.010534	0.224294	0.201316	GCLM/GCL	3
GO:007217	BP	GO:007217	mesoneph	3/902	10/18614	0.010534	0.224294	0.201316	GREM1/HN	3
GO:200034	BP	GO:200034	positive re	3/902	10/18614	0.010534	0.224294	0.201316	TNFAIP3/P	3
GO:000754	BP	GO:000754	sex differe	23/902	283/18614	0.010927	0.231447	0.207736	TNFAIP6/C	23
GO:006033	BP	GO:006033	type I inter	9/902	76/18614	0.011004	0.231447	0.207736	PTPN2/ISG	9
GO:000611	BP	GO:000611	regulation	7/902	51/18614	0.011043	0.231447	0.207736	PDK2/DGK	7
GO:003287	BP	GO:003287	negative re	7/902	51/18614	0.011043	0.231447	0.207736	ITCH/ZNF6	7
GO:007030	BP	GO:007030	negative re	7/902	51/18614	0.011043	0.231447	0.207736	ITCH/ZNF6	7
GO:005512	BP	GO:005512	digestive s	14/902	146/18614	0.011469	0.239633	0.215084	GATA6/TG	14
GO:000660	BP	GO:000660	protein tar	26/902	333/18614	0.011637	0.240547	0.215905	SPCS3/TRA	26
GO:003505	BP	GO:003505	cardiocyte	15/902	161/18614	0.01166	0.240547	0.215905	GREM1/GA	15
GO:004866	BP	GO:004866	regulation	16/902	176/18614	0.011714	0.240547	0.215905	IGFBP3/TN	16
GO:003016	BP	GO:003016	proteoglyc	8/902	64/18614	0.011808	0.240547	0.215905	DSE/DSEL/	8
GO:000275	BP	GO:000275	MyD88-de	4/902	19/18614	0.011851	0.240547	0.215905	IRF1/TIRAF	4
GO:000922	BP	GO:000922	pyrimidine	4/902	19/18614	0.011851	0.240547	0.215905	NTHL1/ME	4
GO:001608	BP	GO:001608	synaptic ve	4/902	19/18614	0.011851	0.240547	0.215905	SNAP23/SN	4
GO:004545	BP	GO:004545	cell redox	6/902	40/18614	0.011936	0.240547	0.215905	NQO1/GCI	6
GO:000680	BP	GO:000680	nitric oxide	9/902	77/18614	0.011942	0.240547	0.215905	CYP1B1/GI	9
GO:007135	BP	GO:007135	cellular res	9/902	77/18614	0.011942	0.240547	0.215905	PTPN2/ISG	9
GO:007208	BP	GO:007208	nephron ep	9/902	77/18614	0.011942	0.240547	0.215905	GREM1/IRX	9
GO:004232	BP	GO:004232	negative re	29/902	383/18614	0.011947	0.240547	0.215905	GREM1/EN	29
GO:000206	BP	GO:000206	columnar/	12/902	118/18614	0.011981	0.240547	0.215905	GATA6/DL	12
GO:001648	BP	GO:001648	cytosolic tr	16/902	177/18614	0.012319	0.245866	0.220678	GOLT1B/C	16
GO:006075	BP	GO:006075	regulation	16/902	177/18614	0.012319	0.245866	0.220678	ZNF675/N	16
GO:190332	BP	GO:190332	regulation	21/902	255/18614	0.012702	0.251121	0.225395	TRIB3/BAG	21
GO:003287	BP	GO:003287	regulation	17/902	193/18614	0.012833	0.251121	0.225395	MAP4K4/IT	17
GO:004885	BP	GO:004885	neural nucl	8/902	65/18614	0.01291	0.251121	0.225395	DYNLL1/H	8
GO:004582	BP	GO:004582	negative re	9/902	78/18614	0.012937	0.251121	0.225395	DUSP10/N	9
GO:000155	BP	GO:000155	ovarian cu	2/902	4/18614	0.013183	0.251121	0.225395	TNFAIP6/P	2
GO:000661	BP	GO:000661	ribosomal	2/902	4/18614	0.013183	0.251121	0.225395	RPL23/IPO	2
GO:001936	BP	GO:001936	pyridine nu	2/902	4/18614	0.013183	0.251121	0.225395	NUDT12/S	2
GO:002200	BP	GO:002200	central ner	2/902	4/18614	0.013183	0.251121	0.225395	ENG/CTNN	2
GO:003590	BP	GO:003590	response t	2/902	4/18614	0.013183	0.251121	0.225395	ACO2/SOD	2
GO:003591	BP	GO:003591	ascending	2/902	4/18614	0.013183	0.251121	0.225395	TGFB2/SOX	2
GO:003802	BP	GO:003802	insulin rece	2/902	4/18614	0.013183	0.251121	0.225395	PTPN2/CTS	2
GO:007233	BP	GO:007233	UDP-galac	2/902	4/18614	0.013183	0.251121	0.225395	SLC35A3/S	2
GO:011002	BP	GO:011002	positive re	2/902	4/18614	0.013183	0.251121	0.225395	GATA6/MA	2
GO:190222	BP	GO:190222	regulation	2/902	4/18614	0.013183	0.251121	0.225395	PTPN2/CSF	2
GO:199016	BP	GO:199016	protein K3	2/902	4/18614	0.013183	0.251121	0.225395	TNFAIP3/Y	2
GO:200058	BP	GO:200058	negative re	2/902	4/18614	0.013183	0.251121	0.225395	PTPN2/PTF	2
GO:007259	BP	GO:007259	maintenan	6/902	41/18614	0.013425	0.254997	0.228873	HSPA5/KD	6
GO:004518	BP	GO:004518	maintenan	10/902	92/18614	0.01351	0.255893	0.229678	HSPA5/NF	10
GO:005067	BP	GO:005067	epithelial c	35/902	488/18614	0.013604	0.256938	0.230616	CCND1/IG	35
GO:003469	BP	GO:003469	response t	5/902	30/18614	0.01373	0.257133	0.230791	GATA6/CY	5
GO:004274	BP	GO:004274	hydrogen p	5/902	30/18614	0.01373	0.257133	0.230791	HBB/GPX3	5
GO:190010	BP	GO:190010	regulation	5/902	30/18614	0.01373	0.257133	0.230791	FICD/HSPA	5
GO:004562	BP	GO:004562	regulation	3/902	11/18614	0.013968	0.260458	0.233776	IL4R/RARA	3
GO:001056	BP	GO:001056	regulation	13/902	135/18614	0.014048	0.260458	0.233776	TRIB3/NR1	13
GO:000689	BP	GO:000689	Golgi to va	4/902	20/18614	0.014256	0.260458	0.233776	CCDC91/V	4
GO:000834	BP	GO:000834	determinat	4/902	20/18614	0.014256	0.260458	0.233776	SIRT6/TRE	4
GO:005509	BP	GO:005509	response t	4/902	20/18614	0.014256	0.260458	0.233776	SOD2/FOX	4
GO:006023	BP	GO:006023	mesenchyr	4/902	20/18614	0.014256	0.260458	0.233776	GREM1/FZ	4
GO:006033	BP	GO:006033	negative re	4/902	20/18614	0.014256	0.260458	0.233776	PTPN2/ISG	4
GO:004558	BP	GO:004558	regulation	16/902	180/18614	0.014284	0.260458	0.233776	IRF1/DUSP	16
GO:004865	BP	GO:004865	smooth mu	16/902	180/18614	0.014284	0.260458	0.233776	IGFBP3/TN	16
GO:003459	BP	GO:003459	cellular res	23/902	290/18614	0.014297	0.260458	0.233776	CYP1B1/PD	23
GO:007156	BP	GO:007156	cellular res	22/902	274/18614	0.014346	0.260647	0.233945	EMILIN1/Z	22
GO:006191	BP	GO:006191	selective a	10/902	93/18614	0.014503	0.262071	0.235223	SNX18/LGA	10
GO:190054	BP	GO:190054	regulation	10/902	93/18614	0.014503	0.262071	0.235223	ME1/PDK2	10
GO:000327	BP	GO:000327	cardiac sep	11/902	107/18614	0.014605	0.263211	0.236246	GATA6/TG	11
GO:007030	BP	GO:007030	regulation	17/902	196/18614	0.014766	0.265385	0.238198	MAP4K4/IT	17
GO:000301	BP	GO:000301	heart proc	21/902	259/18614	0.014915	0.266665	0.239347	GATA6/IRX	21
GO:001400	BP	GO:001400	glial cell pr	7/902	54/18614	0.014917	0.266665	0.239347	VEGFC/SO	7
GO:000604	BP	GO:000604	amino sug	6/902	42/18614	0.015038	0.267999	0.240544	DPAGT1/C	6
GO:000320	BP	GO:000320	cardiac cha	15/902	166/18614	0.015105	0.267999	0.240544	GATA6/TG	15
GO:007202	BP	GO:007202	nephron m	9/902	80/18614	0.015112	0.267999	0.240544	GREM1/IRX	9
GO:005165	BP	GO:005165	establishm	17/902	197/18614	0.015458	0.272522	0.244604	BLOC1S6/	17
GO:000614	BP	GO:000614	regulation	10/902	94/18614	0.015549	0.272522	0.244604	ME1/PDK2	10
GO:000961	BP	GO:000961	response t	30/902	408/18614	0.01558	0.272522	0.244604	IRF1/ITCH/	30
GO:004873	BP	GO:004873	appendage	16/902	182/18614	0.015723	0.272522	0.244604	GREM1/TG	16
GO:006017	BP	GO:006017	limb develo	16/902	182/18614	0.015723	0.272522	0.244604	GREM1/TG	16
GO:001003	BP	GO:001003	response t	5/902	31/18614	0.015733	0.272522	0.244604	CCND1/G6	5
GO:001046	BP	GO:001046	regulation	5/902	31/18614	0.015733	0.272522	0.244604	SHOX2/ST	5
GO:003533	BP	GO:003533	peptidyl-ty	5/902	31/18614	0.015733	0.272522	0.244604	DUSP10/D	5
GO:006067	BP	GO:006067	placenta b	5/902	31/18614	0.015733	0.272522	0.244604	TMED2/ITG	5
GO:005082	BP	GO:005082	protein sta	18/902	213/18614	0.015787	0.272742	0.244801	BAG2/SYV	18
GO:003461	BP	GO:003461	cellular res	14/902	152/18614	0.015895	0.273899	0.24584	CYP1B1/PD	14
GO:000724	BP	GO:000724	I-kappaB	23/902	293/18614	0.015973	0.27454	0.246415	ZNF675/TF	23
GO:007266	BP	GO:007266	protein loc	9/902	81/18614	0.016294	0.279342	0.250725	TNFAIP3/L	9
GO:004274	BP	GO:004274	hydrogen p	7/902	55/18614	0.016399	0.280271	0.251559	HBB/GPX3	7
GO:006102	BP	GO:006102	membrane	16/902	183/18614	0.016484	0.280271	0.251559	BLOC1S6/	16
GO:004856	BP	GO:004856	embryonic	23/902	294/18614	0.016565	0.280271	0.251559	MAFB/IRX5	23
GO:000004	BP	GO:000004	autophago	11/902	109/18614	0.016591	0.280271	0.251559	SNX18/IFT	11
GO:007217	BP	GO:007217	epithelial t	13/902	138/18614	0.016618	0.280271	0.251559	GREM1/IRX	13
GO:001921	BP	GO:001921	regulation	10/902	95/18614	0.01665	0.280271	0.251559	TRIB3/NR1	10
GO:004563	BP	GO:004563	negative re	10/902	95/18614	0.01665	0.280271	0.251559	TNFAIP6/N	10
GO:007149	BP	GO:007149	cellular res	25/902	327/18614	0.016725	0.280271	0.251559	IRF1/SESN	25
GO:000939	BP	GO:000939	2'-deoxyrib	6/902	43/18614	0.016781	0.280271	0.251559	NTHL1/ME	6
GO:001967	BP	GO:001967	NAD meta	6/902	43/18614	0.016781	0.280271	0.251559	ME1/NADK	6
GO:000208	BP	GO:000208	lens morph	4/902	21/18614	0.01695	0.280271	0.251559	SOX11/HIF	4
GO:001088	BP	GO:001088	negative re	4/902	21/18614	0.01695	0.280271	0.251559	NR1H3/PT	4
GO:200073	BP	GO:200073	positive re	4/902	21/18614	0.01695	0.280271	0.251559	TGFB2/SIR	4
GO:004349	BP	GO:004349	protein kin	18/902	215/18614	0.017209	0.280271	0.251559	ZFP36L1/S	18
GO:004217	BP	GO:004217	regulation	27/902	361/18614	0.017224	0.280271	0.251559	TRIB3/BAG	27
GO:004657	BP	GO:004657	regulation	16/902	184/18614	0.017273	0.280271	0.251559	MAP4K4/T	16
GO:005068	BP	GO:005068	regulation	13/902	139/18614	0.01755	0.280271	0.251559	ZFP36L1/R	13
GO:199077	BP	GO:199077	protein loc	26/902	345/18614	0.017575	0.280271	0.251559	RAP2A/RA	26
GO:000283	BP	GO:000283	regulation	33/902	463/18614	0.017613	0.280271	0.251559	EMILIN1/G	33
GO:003514	BP	GO:003514	tube forma	14/902	154/18614	0.017629	0.280271	0.251559	GREM1/IRX	14
GO:000609	BP	GO:000609	tricarboxyli	5/902	32/18614	0.017923	0.280271	0.251559	ACO2/SDH	5
GO:000661	BP	GO:000661	cotranslati	5/902	32/18614	0.017923	0.280271	0.251559	SRP9/SSR2	5
GO:003545	BP	GO:003545	response t	5/902	32/18614	0.017923	0.280271	0.251559	IRF1/IFI16/	5
GO:190223	BP	GO:190223	regulation	5/902	32/18614	0.017923	0.280271	0.251559	PMAIP1/SY	5
GO:000333	BP	GO:000333	mesenchyr	3/902	12/18614	0.017963	0.280271	0.251559	GREM1/ST	3
GO:000628	BP	GO:000628	base-excis	3/902	12/18614	0.017963	0.280271	0.251559	NTHL1/ME	3
GO:003165	BP	GO:003165	regulation	3/902	12/18614	0.017963	0.280271	0.251559	ARRDC3/P	3
GO:003290	BP	GO:003290	regulation	3/902	12/18614	0.017963	0.280271	0.251559	GATA6/LU	3
GO:003414	BP	GO:003414	positive re	3/902	12/18614	0.017963	0.280271	0.251559	NR1H3/TIR	3
GO:003636	BP	GO:003636	transformi	3/902	12/18614	0.017963	0.280271	0.251559	GATA6/ITG	3
GO:003818	BP	GO:003818	nerve grow	3/902	12/18614	0.017963	0.280271	0.251559	RAP1A/SO	3
GO:005065	BP	GO:005065	dermatan	3/902	12/18614	0.017963	0.280271	0.251559	DSE/DSEL/	3
GO:007163	BP	GO:007163	negative re	3/902	12/18614	0.017963	0.280271	0.251559	GATA6/TSI	3
GO:009008	BP	GO:009008	negative re	3/902	12/18614	0.017963	0.280271	0.251559	DNAJB6/H	3
GO:009016	BP	GO:009016	Golgi to lys	3/902	12/18614	0.017963	0.280271	0.251559	CCDC91/S	3
GO:009067	BP	GO:009067	reversible	3/902	12/18614	0.017963	0.280271	0.251559	SOD2/FGF	3
GO:007155	BP	GO:007155	response t	22/902	280/18614	0.017979	0.280271	0.251559	EMILIN1/Z	22
GO:003260	BP	GO:003260	interferon-	7/902	56/18614	0.017983	0.280271	0.251559	IRF1/ISG15	7
GO:003264	BP	GO:003264	regulation	7/902	56/18614	0.017983	0.280271	0.251559	IRF1/ISG15	7
GO:004576	BP	GO:004576	positive re	16/902	185/18614	0.01809	0.280627	0.251878	GREM1/EN	16
GO:190401	BP	GO:190401	positive re	16/902	185/18614	0.01809	0.280627	0.251878	GREM1/EN	16
GO:000725	BP	GO:000725	JNK cascad	15/902	170/18614	0.018389	0.284601	0.255445	MAP4K4/IT	15
GO:005114	BP	GO:005114	striated mu	23/902	297/18614	0.018446	0.284822	0.255644	GREM1/GA	23
GO:000926	BP	GO:000926	deoxyribor	6/902	44/18614	0.018658	0.285301	0.256074	NTHL1/ME	6
GO:001969	BP	GO:001969	deoxyribos	6/902	44/18614	0.018658	0.285301	0.256074	NTHL1/ME	6
GO:002176	BP	GO:002176	substantia	6/902	44/18614	0.018658	0.285301	0.256074	DYNLL1/H	6
GO:004277	BP	GO:004277	intrinsic ap	6/902	44/18614	0.018658	0.285301	0.256074	PHLDA3/R	6
GO:000703	BP	GO:000703	vacuole org	18/902	217/18614	0.018728	0.285301	0.256074	SNX18/CH	18
GO:000157	BP	GO:000157	vasculogen	9/902	83/18614	0.018861	0.285301	0.256074	ZFP36L1/R	9
GO:000749	BP	GO:000749	endoderm	9/902	83/18614	0.018861	0.285301	0.256074	GATA6/ZF	9
GO:003434	BP	GO:003434	response t	9/902	83/18614	0.018861	0.285301	0.256074	PTPN2/ISG	9
GO:004620	BP	GO:004620	nitric oxide	9/902	83/18614	0.018861	0.285301	0.256074	CYP1B1/GI	9
GO:200004	BP	GO:200004	regulation	16/902	186/18614	0.018937	0.285618	0.256358	CCND1/RF	16
GO:000703	BP	GO:000703	endosome	10/902	97/18614	0.019023	0.285618	0.256358	CHMP5/PL	10
GO:006099	BP	GO:006099	kidney mor	10/902	97/18614	0.019023	0.285618	0.256358	GREM1/IRX	10
GO:000726	BP	GO:000726	small GTPa	34/902	483/18614	0.019068	0.285618	0.256358	MAP4K4/R	34
GO:200123	BP	GO:200123	regulation	28/902	381/18614	0.019095	0.285618	0.256358	PMAIP1/SY	28
GO:000269	BP	GO:000269	negative re	17/902	202/18614	0.019306	0.287597	0.258134	IRF1/ITCH/	17
GO:000602	BP	GO:000602	aminoglyca	12/902	126/18614	0.019314	0.287597	0.258134	TNFAIP6/D	12
GO:000726	BP	GO:000726	neurotrans	13/902	141/18614	0.019531	0.288457	0.258906	CADPS2/B	13
GO:009964	BP	GO:009964	signal relea	13/902	141/18614	0.019531	0.288457	0.258906	CADPS2/B	13
GO:000170	BP	GO:000170	endoderm	7/902	57/18614	0.019672	0.288457	0.258906	GATA6/CC	7
GO:005160	BP	GO:005160	protein ma	25/902	332/18614	0.019784	0.288457	0.258906	BAG2/MAF	25
GO:003052	BP	GO:003052	intracellula	23/902	299/18614	0.01979	0.288457	0.258906	ITCH/AHR	23
GO:000821	BP	GO:000821	regulation	16/902	187/18614	0.019814	0.288457	0.258906	EMILIN1/E	16
GO:000322	BP	GO:000322	atrial cardi	4/902	22/18614	0.01994	0.288457	0.258906	GATA6/SH	4
GO:000624	BP	GO:000624	pyrimidine	4/902	22/18614	0.01994	0.288457	0.258906	NTHL1/ME	4
GO:003144	BP	GO:003144	regulation	4/902	22/18614	0.01994	0.288457	0.258906	ZFP36L1/C	4
GO:003162	BP	GO:003162	synaptic ve	4/902	22/18614	0.01994	0.288457	0.258906	SNAP23/C	4
GO:003410	BP	GO:003410	negative re	4/902	22/18614	0.01994	0.288457	0.258906	GREM1/TN	4
GO:004659	BP	GO:004659	negative re	4/902	22/18614	0.01994	0.288457	0.258906	TRIM8/PTX	4
GO:000609	BP	GO:000609	gluconeog	9/902	84/18614	0.020248	0.288457	0.258906	PDK2/DGK	9
GO:200105	BP	GO:200105	reactive nit	9/902	84/18614	0.020248	0.288457	0.258906	CYP1B1/GI	9
GO:009730	BP	GO:009730	cellular res	10/902	98/18614	0.020299	0.288457	0.258906	AHR/CYP1	10
GO:000206	BP	GO:000206	chondrocy	5/902	33/18614	0.020305	0.288457	0.258906	SHOX2/PT	5
GO:004838	BP	GO:004838	retinoic aci	5/902	33/18614	0.020305	0.288457	0.258906	CYP26B1/	5
GO:004352	BP	GO:004352	regulation	18/902	219/18614	0.020348	0.288457	0.258906	CCND1/TG	18
GO:006034	BP	GO:006034	bone deve	19/902	235/18614	0.020413	0.288457	0.258906	GREM1/PP	19
GO:000726	BP	GO:000726	Ras protei	25/902	333/18614	0.020445	0.288457	0.258906	MAP4K4/R	25
GO:007048	BP	GO:007048	response t	25/902	333/18614	0.020445	0.288457	0.258906	GATA6/ZF	25
GO:000188	BP	GO:000188	liver devel	13/902	142/18614	0.020582	0.288457	0.258906	CCND1/GA	13
GO:007233	BP	GO:007233	signal trans	15/902	173/18614	0.021189	0.288457	0.258906	PHLDA3/R	15
GO:009719	BP	GO:009719	intrinsic ap	23/902	301/18614	0.021209	0.288457	0.258906	TRIB3/PHL	23
GO:000245	BP	GO:000245	T cell antig	2/902	5/18614	0.02127	0.288457	0.258906	TREX1/RFT	2
GO:000328	BP	GO:000328	septum pri	2/902	5/18614	0.02127	0.288457	0.258906	TGFB2/SOX	2
GO:000328	BP	GO:000328	atrial sept	2/902	5/18614	0.02127	0.288457	0.258906	TGFB2/SOX	2
GO:000611	BP	GO:000611	NADH oxi	2/902	5/18614	0.02127	0.288457	0.258906	GPD2/NQ	2
GO:000612	BP	GO:000612	mitochond	2/902	5/18614	0.02127	0.288457	0.258906	SDHD/SDH	2
GO:001028	BP	GO:001028	heat acclin	2/902	5/18614	0.02127	0.288457	0.258906	HSBP1L1/H	2
GO:001051	BP	GO:001051	regulation	2/902	5/18614	0.02127	0.288457	0.258906	HTR2A/PD	2
GO:003552	BP	GO:003552	protein K2	2/902	5/18614	0.02127	0.288457	0.258906	TNFAIP3/Y	2
GO:003590	BP	GO:003590	ascending	2/902	5/18614	0.02127	0.288457	0.258906	TGFB2/SOX	2
GO:004475	BP	GO:004475	response t	2/902	5/18614	0.02127	0.288457	0.258906	GCLM/GCL	2
GO:004563	BP	GO:004563	positive re	2/902	5/18614	0.02127	0.288457	0.258906	ZEB2/KITL	2
GO:004590	BP	GO:004590	negative re	2/902	5/18614	0.02127	0.288457	0.258906	CPEB3/SRF	2
GO:007037	BP	GO:007037	cellular hea	2/902	5/18614	0.02127	0.288457	0.258906	HSBP1L1/H	2
GO:009018	BP	GO:009018	negative re	2/902	5/18614	0.02127	0.288457	0.258906	STAT1/HN	2
GO:009706	BP	GO:009706	response t	2/902	5/18614	0.02127	0.288457	0.258906	GCLM/GCL	2
GO:009870	BP	GO:009870	glucose im	2/902	5/18614	0.02127	0.288457	0.258906	SLC2A3/SL	2
GO:190329	BP	GO:190329	protein loc	2/902	5/18614	0.02127	0.288457	0.258906	ARL5A/ARI	2
GO:190389	BP	GO:190389	negative re	2/902	5/18614	0.02127	0.288457	0.258906	HSPA5/DN	2
GO:190497	BP	GO:190497	regulation	2/902	5/18614	0.02127	0.288457	0.258906	TOM1/RAB	2
GO:190500	BP	GO:190500	positive re	2/902	5/18614	0.02127	0.288457	0.258906	TGFB2/EN	2
GO:004800	BP	GO:004800	platelet-de	7/902	58/18614	0.021468	0.290556	0.26079	PIK3C2A/IF	7
GO:000649	BP	GO:000649	protein O-	10/902	99/18614	0.021636	0.292238	0.262299	ST6GALNA	10
GO:005140	BP	GO:005140	stress-acti	19/902	237/18614	0.022077	0.297597	0.26711	MAP4K4/IT	19
GO:000235	BP	GO:000235	defense re	3/902	13/18614	0.022526	0.299419	0.268745	DAPK1/PRI	3
GO:001064	BP	GO:001064	negative re	3/902	13/18614	0.022526	0.299419	0.268745	PTPN2/SN	3
GO:003015	BP	GO:003015	pancreatic	3/902	13/18614	0.022526	0.299419	0.268745	NR1H3/NP	3
GO:003543	BP	GO:003543	maintenan	3/902	13/18614	0.022526	0.299419	0.268745	HSPA5/KD	3
GO:006067	BP	GO:006067	branching	3/902	13/18614	0.022526	0.299419	0.268745	RSPO3/TM	3
GO:007042	BP	GO:007042	regulation	3/902	13/18614	0.022526	0.299419	0.268745	TNFAIP3/B	3
GO:007253	BP	GO:007253	fibroblast	3/902	13/18614	0.022526	0.299419	0.268745	CYGB/GCL	3
GO:004668	BP	GO:004668	response t	12/902	129/18614	0.022787	0.300002	0.269269	GATA6/ZF	12
GO:000602	BP	GO:000602	glycosamir	8/902	72/18614	0.022831	0.300002	0.269269	DSE/DSEL/	8
GO:003538	BP	GO:003538	thioester b	6/902	46/18614	0.022832	0.300002	0.269269	PDK2/DLA	6
GO:007161	BP	GO:007161	acyl-CoA	6/902	46/18614	0.022832	0.300002	0.269269	PDK2/DLA	6
GO:004557	BP	GO:004557	regulation	5/902	34/18614	0.022884	0.300002	0.269269	ZFP36L1/I	5
GO:005086	BP	GO:005086	negative re	5/902	34/18614	0.022884	0.300002	0.269269	TNFAIP3/I	5
GO:190018	BP	GO:190018	negative re	5/902	34/18614	0.022884	0.300002	0.269269	SUMO1/SI	5
GO:001905	BP	GO:001905	viral life cy	24/902	320/18614	0.023026	0.300228	0.269471	ITCH/TRIM	24
GO:004206	BP	GO:004206	gliogenesis	24/902	320/18614	0.023026	0.300228	0.269471	DUSP10/T	24
GO:001908	BP	GO:001908	viral gene	10/902	100/18614	0.023036	0.300228	0.269471	SPCS3/TRI	10
GO:009950	BP	GO:009950	vesicle fusi	4/902	23/18614	0.023236	0.302249	0.271285	SNAP23/C	4
GO:009015	BP	GO:009015	establishm	21/902	271/18614	0.023414	0.30397	0.27283	EMC2/RAB	21
GO:004230	BP	GO:004230	molting cy	11/902	115/18614	0.023762	0.306656	0.27524	TGFB2/DL	11
GO:004263	BP	GO:004263	hair cycle	11/902	115/18614	0.023762	0.306656	0.27524	TGFB2/DL	11
GO:190503	BP	GO:190503	autophago	11/902	115/18614	0.023762	0.306656	0.27524	SNX18/IFT	11
GO:000762	BP	GO:000762	circadian r	17/902	207/18614	0.02385	0.306656	0.27524	PTGDS/AH	17
GO:004259	BP	GO:004259	response t	17/902	207/18614	0.02385	0.306656	0.27524	SESN3/PM	17
GO:003526	BP	GO:003526	multicellula	13/902	145/18614	0.02399	0.306754	0.275329	ZFP36L1/G	13
GO:006100	BP	GO:006100	hepaticobi	13/902	145/18614	0.02399	0.306754	0.275329	CCND1/GA	13
GO:003011	BP	GO:003011	regulation	25/902	338/18614	0.024015	0.306754	0.275329	GREM1/RS	25
GO:004562	BP	GO:004562	positive re	12/902	130/18614	0.024041	0.306754	0.275329	DUSP10/TI	12
GO:004346	BP	GO:004346	regulation	8/902	73/18614	0.024592	0.313183	0.281099	SUMO1/DI	8
GO:190165	BP	GO:190165	response t	17/902	208/18614	0.024848	0.314696	0.282457	CCND1/AH	17
GO:001931	BP	GO:001931	hexose bio	9/902	87/18614	0.024852	0.314696	0.282457	PDK2/DGK	9
GO:004814	BP	GO:004814	regulation	9/902	87/18614	0.024852	0.314696	0.282457	MED31/SIF	9
GO:004564	BP	GO:004564	regulation	6/902	47/18614	0.025137	0.316636	0.284198	MAFB/ZFP	6
GO:006071	BP	GO:006071	labyrinthin	6/902	47/18614	0.025137	0.316636	0.284198	ZFP36L1/R	6
GO:004558	BP	GO:004558	positive re	11/902	116/18614	0.025147	0.316636	0.284198	DUSP10/TI	11
GO:000722	BP	GO:000722	smoothene	13/902	146/18614	0.025214	0.316886	0.284422	DYRK2/GLI	13
GO:004682	BP	GO:004682	positive re	7/902	60/18614	0.025395	0.31814	0.285548	HSP90AA1	7
GO:002290	BP	GO:002290	electron tra	15/902	177/18614	0.025409	0.31814	0.285548	GPD2/NDU	15
GO:004426	BP	GO:004426	cellular car	22/902	290/18614	0.025592	0.31983	0.287065	IGFBP3/GP	22
GO:004573	BP	GO:004573	positive re	17/902	209/18614	0.025878	0.3228	0.289731	TRIB3/BAG	17
GO:003031	BP	GO:003031	osteoclast	10/902	102/18614	0.02603	0.324088	0.290887	TNFAIP6/N	10
GO:000193	BP	GO:000193	negative re	25/902	341/18614	0.026377	0.327381	0.293843	GREM1/EN	25
GO:000602	BP	GO:000602	aminoglyca	8/902	74/18614	0.026446	0.327381	0.293843	DSE/DSEL/	8
GO:003033	BP	GO:003033	negative re	27/902	375/18614	0.026515	0.327381	0.293843	GREM1/EN	27
GO:003227	BP	GO:003227	positive re	9/902	88/18614	0.026539	0.327381	0.293843	BAG4/HSP	9
GO:003327	BP	GO:003327	response t	9/902	88/18614	0.026539	0.327381	0.293843	CCND1/CY	9
GO:003020	BP	GO:003020	glycosamir	11/902	117/18614	0.02659	0.327407	0.293866	TNFAIP6/D	11
GO:004358	BP	GO:004358	skin develo	23/902	308/18614	0.026807	0.327503	0.293952	GATA6/MA	23
GO:007137	BP	GO:007137	cellular res	23/902	308/18614	0.026807	0.327503	0.293952	TRIB3/ZFP	23
GO:001064	BP	GO:001064	regulation	4/902	24/18614	0.026843	0.327503	0.293952	IFT20/PTPN	4
GO:003133	BP	GO:003133	regulation	4/902	24/18614	0.026843	0.327503	0.293952	SNCA/CPL	4
GO:006148	BP	GO:006148	hematopoi	4/902	24/18614	0.026843	0.327503	0.293952	ARMCX1/G	4
GO:004343	BP	GO:004343	response t	30/902	427/18614	0.027039	0.328448	0.2948	TRIB3/ZFP	30
GO:004559	BP	GO:004559	negative re	7/902	61/18614	0.027531	0.328448	0.2948	TRIB3/SOD	7
GO:003164	BP	GO:003164	regulation	6/902	48/18614	0.027591	0.328448	0.2948	ZPR1/PTN	6
GO:003598	BP	GO:003598	endoderm	6/902	48/18614	0.027591	0.328448	0.2948	GATA6/CC	6
GO:004806	BP	GO:004806	developme	6/902	48/18614	0.027591	0.328448	0.2948	BLOC1S6/	6
GO:000177	BP	GO:000177	leukocyte	10/902	103/18614	0.027627	0.328448	0.2948	PMAIP1/SH	10

hsa05310	hsa05310	Asthma	1/523	31/8662	0.855444	0.994912	0.971569	3108	1
hsa04640	hsa04640	Hematopo	4/523	99/8662	0.856551	0.994912	0.971569	926/928/3	4
hsa05134	hsa05134	Legionella	2/523	56/8662	0.860268	0.994912	0.971569	56681/741	2
hsa04215	hsa04215	Apoptosis	1/523	32/8662	0.864203	0.994912	0.971569	666	1
hsa04914	hsa04914	Progester	4/523	102/8662	0.871885	0.994912	0.971569	109/107/8	4
hsa04370	hsa04370	VEGF sign	2/523	59/8662	0.879333	0.994912	0.971569	5336/5293	2
hsa03020	hsa03020	RNA polyr	1/523	34/8662	0.880165	0.994912	0.971569	10623	1
hsa04730	hsa04730	Long-term	2/523	60/8662	0.885135	0.994912	0.971569	3710/1067	2
hsa04623	hsa04623	Cytosolic D	3/523	83/8662	0.885192	0.994912	0.971569	10623/853	3
hsa03083	hsa03083	Polycomb	3/523	84/8662	0.890013	0.994912	0.971569	22955/260	3
hsa05226	hsa05226	Gastric car	6/523	149/8662	0.893584	0.994912	0.971569	51384/224	6
hsa00512	hsa00512	Mucin type	1/523	36/8662	0.894253	0.994912	0.971569	374378	1
hsa04330	hsa04330	Notch sign	2/523	62/8662	0.895937	0.994912	0.971569	342371/83	2
hsa04620	hsa04620	Toll-like re	4/523	108/8662	0.898357	0.994912	0.971569	7189/1480	4
hsa05210	hsa05210	Colorectal	3/523	86/8662	0.899114	0.994912	0.971569	83439/693	3
hsa00760	hsa00760	Nicotinate	1/523	37/8662	0.900665	0.994912	0.971569	22978	1
hsa05143	hsa05143	African try	1/523	37/8662	0.900665	0.994912	0.971569	3250	1
hsa05171	hsa05171	Coronavir	10/523	232/8662	0.90132	0.994912	0.971569	4599/629/	10
hsa04068	hsa04068	FoxO signa	5/523	131/8662	0.902951	0.994912	0.971569	1032/2309	5
hsa04020	hsa04020	Calcium si	11/523	253/8662	0.904833	0.994912	0.971569	154/80271	11
hsa04380	hsa04380	Osteoclast	5/523	135/8662	0.917071	0.994912	0.971569	5336/3710	5
hsa05033	hsa05033	Nicotine a	1/523	40/8662	0.917663	0.994912	0.971569	2560	1
hsa04720	hsa04720	Long-term	2/523	67/8662	0.919041	0.994912	0.971569	3710/107	2
hsa04658	hsa04658	Th1 and Th	3/523	92/8662	0.922487	0.994912	0.971569	3717/3108	3
hsa05222	hsa05222	Small cell l	3/523	92/8662	0.922487	0.994912	0.971569	7189/5293	3
hsa00830	hsa00830	Retinol me	2/523	68/8662	0.923037	0.994912	0.971569	220/8228	2
hsa05031	hsa05031	Amphetam	2/523	69/8662	0.926847	0.994912	0.971569	9586/1644	2
hsa05211	hsa05211	Renal cell c	2/523	69/8662	0.926847	0.994912	0.971569	5062/5293	2
hsa00860	hsa00860	Porphyrin r	1/523	43/8662	0.931758	0.994912	0.971569	645	1
hsa05218	hsa05218	Melanoma	2/523	72/8662	0.937237	0.994912	0.971569	2246/5293	2
hsa04621	hsa04621	NOD-like r	7/523	186/8662	0.938226	0.994912	0.971569	3710/7189	7
hsa04630	hsa04630	JAK-STAT	6/523	166/8662	0.941186	0.994912	0.971569	3717/2352	6
hsa04061	hsa04061	Viral prote	3/523	100/8662	0.945968	0.994912	0.971569	3570/8797	3
hsa05214	hsa05214	Glioma	2/523	75/8662	0.946218	0.994912	0.971569	5336/5293	2
hsa04973	hsa04973	Carbohydr	1/523	47/8662	0.946876	0.994912	0.971569	5293	1
hsa05220	hsa05220	Chronic my	2/523	76/8662	0.94893	0.994912	0.971569	5293/3551	2
hsa05202	hsa05202	Transcripti	7/523	193/8662	0.9515	0.994912	0.971569	51384/643	7
hsa04672	hsa04672	Intestinal i	1/523	49/8662	0.953131	0.994912	0.971569	3108	1
hsa04721	hsa04721	Synaptic ve	2/523	78/8662	0.953968	0.994912	0.971569	1785/1759	2
hsa05144	hsa05144	Malaria	1/523	50/8662	0.955977	0.994912	0.971569	7060	1
hsa04010	hsa04010	MAPK sign	12/523	301/8662	0.956891	0.994912	0.971569	4216/5129	12
hsa04921	hsa04921	Oxytocin si	5/523	154/8662	0.960111	0.994912	0.971569	5020/3710	5
hsa05152	hsa05152	Tuberculos	6/523	180/8662	0.965028	0.994912	0.971569	3916/3717	6
hsa05131	hsa05131	Shigellosis	9/523	247/8662	0.966895	0.994912	0.971569	5336/3710	9
hsa04742	hsa04742	Taste trans	2/523	86/8662	0.969758	0.994912	0.971569	3710/2550	2
hsa04668	hsa04668	TNF signali	3/523	114/8662	0.971905	0.994912	0.971569	9586/5293	3
hsa05034	hsa05034	Alcoholism	6/523	188/8662	0.974309	0.994912	0.971569	2788/9586	6
hsa00590	hsa00590	Arachidoni	1/523	61/8662	0.97791	0.994912	0.971569	151056	1
hsa03010	hsa03010	Ribosome	5/523	169/8662	0.97839	0.994912	0.971569	29088/649	5
hsa05323	hsa05323	Rheumatoi	2/523	93/8662	0.979176	0.994912	0.971569	537/3108	2
hsa04919	hsa04919	Thyroid ho	3/523	121/8662	0.979928	0.994912	0.971569	5336/5333	3
hsa05206	hsa05206	MicroRNAs	9/523	310/8662	0.996698	1	0.976538	5336/5268	9
hsa04613	hsa04613	Neutrophil	4/523	191/8662	0.997527	1	0.976538	5336/293/	4
hsa05322	hsa05322	Systemic lu	2/523	137/8662	0.998171	1	0.976538	6632/3108	2
hsa04060	hsa04060	Cytokine-c	6/523	297/8662	0.999789	1	0.976538	23529/581	6
hsa04740	hsa04740	Olfactory t	4/523	439/8662	1	1	0.976538	2788/109/	4

hsa04725	hsa04725	Cholinergi	3/482	113/8662	0.955058	1	0.967742	1103/5647	3
hsa04610	hsa04610	Compleme	2/482	86/8662	0.956594	1	0.967742	7035/1675	2
hsa04911	hsa04911	Insulin sec	2/482	86/8662	0.956594	1	0.967742	5864/6513	2
hsa04923	hsa04923	Regulation	1/482	58/8662	0.964278	1	0.967742	5290	1
hsa04929	hsa04929	GnRH secr	1/482	64/8662	0.974727	1	0.967742	5290	1
hsa00970	hsa00970	Aminoacyl	1/482	66/8662	0.977481	1	0.967742	79587	1
hsa04114	hsa04114	Oocyte me	3/482	131/8662	0.979502	1	0.967742	22849/550	3
hsa04650	hsa04650	Natural kill	3/482	132/8662	0.980396	1	0.967742	637/5290/	3
hsa04924	hsa04924	Renin secr	1/482	69/8662	0.981061	1	0.967742	5732	1
hsa05031	hsa05031	Amphetam	1/482	69/8662	0.981061	1	0.967742	5500	1
hsa05020	hsa05020	Prion disea	8/482	272/8662	0.986596	1	0.967742	7277/4698	8
hsa03010	hsa03010	Ribosome	4/482	169/8662	0.986715	1	0.967742	51065/618	4
hsa04060	hsa04060	Cytokine-c	8/482	297/8662	0.986886	1	0.967742	9518/7042	8
hsa04971	hsa04971	Gastric aci	1/482	76/8662	0.987358	1	0.967742	3772	1
hsa04024	hsa04024	cAMP sign	6/482	225/8662	0.9881	1	0.967742	5732/5500	6
hsa04724	hsa04724	Glutamate	2/482	115/8662	0.989599	1	0.967742	2912/9454	2
hsa04723	hsa04723	Retrograde	3/482	148/8662	0.990509	1	0.967742	4698/2570	3
hsa04360	hsa04360	Axon guid	4/482	182/8662	0.992375	1	0.967742	23380/529	4
hsa04976	hsa04976	Bile secret	1/482	89/8662	0.994008	1	0.967742	6513	1
hsa05034	hsa05034	Alcoholism	4/482	188/8662	0.994126	1	0.967742	5500/4915	4
hsa00230	hsa00230	Purine met	2/482	128/8662	0.994622	1	0.967742	132/12458	2
hsa05032	hsa05032	Morphine	1/482	91/8662	0.99469	1	0.967742	2570	1
hsa04912	hsa04912	GnRH sign	1/482	93/8662	0.99527	1	0.967742	998	1
hsa04270	hsa04270	Vascular s	2/482	134/8662	0.996046	1	0.967742	5500/8157	2
hsa05322	hsa05322	Systemic lu	2/482	137/8662	0.996612	1	0.967742	3117/6741	2
hsa04061	hsa04061	Viral prote	1/482	100/8662	0.996847	1	0.967742	1435	1
hsa04921	hsa04921	Oxytocin si	2/482	154/8662	0.9986	1	0.967742	595/5500	2
hsa04726	hsa04726	Serotonerg	1/482	115/8662	0.998679	1	0.967742	3356	1
hsa04022	hsa04022	cGMP-PKC	2/482	167/8662	0.999293	1	0.967742	5500/1910	2
hsa04728	hsa04728	Dopamine	1/482	132/8662	0.999508	1	0.967742	5500	1
hsa04080	hsa04080	Neuroactiv	8/482	367/8662	0.999636	1	0.967742	2743/5732	8
hsa03008	hsa03008	Ribosome	1/482	167/8662	0.999936	1	0.967742	54913	1
hsa04613	hsa04613	Neutrophil	1/482	191/8662	0.999984	1	0.967742	5290	1
hsa04740	hsa04740	Olfactory t	1/482	439/8662	1	1	0.967742	390892	1

GO:190233	BP	GO:190233	negative re	2/1035	6/18614	0.039891	0.45089	0.432223	FOXC1/PA	2
GO:190302	BP	GO:190302	negative re	2/1035	6/18614	0.039891	0.45089	0.432223	HAND1/ZN	2
GO:190352	BP	GO:190352	regulation	2/1035	6/18614	0.039891	0.45089	0.432223	ASAP1/DN	2
GO:190474	BP	GO:190474	negative re	2/1035	6/18614	0.039891	0.45089	0.432223	FOXC1/PA	2
GO:190477	BP	GO:190477	positive reg	2/1035	6/18614	0.039891	0.45089	0.432223	NUMA1/GI	2
GO:190479	BP	GO:190479	positive reg	2/1035	6/18614	0.039891	0.45089	0.432223	FOXC1/PA	2
GO:190495	BP	GO:190495	positive reg	26/1035	325/18614	0.040198	0.45089	0.432223	ARHGEF5/	26
GO:003538	BP	GO:003538	thioester b	6/1035	46/18614	0.040888	0.45089	0.432223	PPT1/PDHE	6
GO:007161	BP	GO:007161	acyl-CoA b	6/1035	46/18614	0.040888	0.45089	0.432223	PPT1/PDHE	6
GO:000699	BP	GO:000699	nuclear env	7/1035	58/18614	0.041019	0.45089	0.432223	NSFL1C/VI	7
GO:004361	BP	GO:004361	keratinocyt	7/1035	58/18614	0.041019	0.45089	0.432223	PRKD1/HA	7
GO:004883	BP	GO:004883	inner ear d	17/1035	193/18614	0.041048	0.45089	0.432223	SLC44A4/N	17
GO:000641	BP	GO:000641	translation	12/1035	123/18614	0.041069	0.45089	0.432223	BANK1/EIF	12
GO:001802	BP	GO:001802	peptidyl-ly	12/1035	123/18614	0.041069	0.45089	0.432223	SETD4/SM	12
GO:004424	BP	GO:004424	cellular lipi	19/1035	222/18614	0.041233	0.45089	0.432223	PPT1/ACAI	19
GO:000175	BP	GO:000175	organ indu	4/1035	24/18614	0.041525	0.45089	0.432223	AR/FGF1/R	4
GO:003612	BP	GO:003612	cellular res	4/1035	24/18614	0.041525	0.45089	0.432223	HYAL1/HA	4
GO:004243	BP	GO:004243	melanin bi	4/1035	24/18614	0.041525	0.45089	0.432223	MC1R/TRP	4
GO:004801	BP	GO:004801	neurotroph	4/1035	24/18614	0.041525	0.45089	0.432223	DDIT4/AG	4
GO:006015	BP	GO:006015	platelet de	4/1035	24/18614	0.041525	0.45089	0.432223	BLOC1S3/	4
GO:190390	BP	GO:190390	positive reg	4/1035	24/18614	0.041525	0.45089	0.432223	AXL/VPS37	4
GO:000647	BP	GO:000647	protein ace	18/1035	208/18614	0.04205	0.45089	0.432223	MUC1/NA	18
GO:003112	BP	GO:003112	developme	5/1035	35/18614	0.042764	0.45089	0.432223	AR/FGF1/S	5
GO:003220	BP	GO:003220	negative re	5/1035	35/18614	0.042764	0.45089	0.432223	PINX1/EXC	5
GO:004587	BP	GO:004587	negative re	5/1035	35/18614	0.042764	0.45089	0.432223	GPR161/SU	5
GO:005125	BP	GO:005125	positive reg	26/1035	327/18614	0.042777	0.45089	0.432223	LAMP1/AX	26
GO:007217	BP	GO:007217	epithelial tu	13/1035	138/18614	0.043787	0.45089	0.432223	COBL/TCTI	13
GO:007053	BP	GO:007053	protein K6	7/1035	59/18614	0.044401	0.45089	0.432223	PRPF19/TR	7
GO:190305	BP	GO:190305	regulation	7/1035	59/18614	0.044401	0.45089	0.432223	HAS2/DDR	7
GO:001895	BP	GO:001895	phenol-co	11/1035	111/18614	0.044649	0.45089	0.432223	PAX8/MC1	11
GO:000698	BP	GO:000698	ER-nucleus	6/1035	47/18614	0.044757	0.45089	0.432223	EIF2S1/BO	6
GO:004880	BP	GO:004880	genitalia d	6/1035	47/18614	0.044757	0.45089	0.432223	AXL/AR/DH	6
GO:000206	BP	GO:000206	epithelial c	18/1035	210/18614	0.045456	0.45089	0.432223	PALLD/MA	18
GO:001081	BP	GO:001081	positive reg	12/1035	125/18614	0.045555	0.45089	0.432223	JAK2/HAS2	12
GO:004348	BP	GO:004348	RNA stabili	8/1035	72/18614	0.045939	0.45089	0.432223	RBM10/RB	8
GO:004215	BP	GO:004215	lipoprotein	13/1035	139/18614	0.045958	0.45089	0.432223	PPT1/PIGT	13
GO:001657	BP	GO:001657	histone ace	14/1035	153/18614	0.045977	0.45089	0.432223	MUC1/RUV	14
GO:000152	BP	GO:000152	retinoid me	9/1035	85/18614	0.04622	0.45089	0.432223	ALDH8A1/	9
GO:000609	BP	GO:000609	glycolytic p	9/1035	85/18614	0.04622	0.45089	0.432223	JMJD8/OGI	9
GO:190236	BP	GO:190236	negative re	9/1035	85/18614	0.04622	0.45089	0.432223	RBM10/RB	9
GO:000639	BP	GO:000639	mRNA pro	37/1035	500/18614	0.047029	0.45089	0.432223	FAM172A/	37
GO:000005	BP	GO:000005	ribosomal	3/1035	15/18614	0.047251	0.45089	0.432223	RRS1/NMD	3
GO:001086	BP	GO:001086	positive reg	3/1035	15/18614	0.047251	0.45089	0.432223	LDLR/RGN	3
GO:003375	BP	GO:003375	ribosome l	3/1035	15/18614	0.047251	0.45089	0.432223	RRS1/NMD	3
GO:004297	BP	GO:004297	activation	3/1035	15/18614	0.047251	0.45089	0.432223	JAK2/IL6R/	3
GO:004299	BP	GO:004299	cytoplasmi	3/1035	15/18614	0.047251	0.45089	0.432223	FAF1/SRI/S	3
GO:004332	BP	GO:004332	protein tra	3/1035	15/18614	0.047251	0.45089	0.432223	VPS37B/VI	3
GO:004517	BP	GO:004517	apical prot	3/1035	15/18614	0.047251	0.45089	0.432223	SHROOM3	3
GO:190002	BP	GO:190002	positive reg	3/1035	15/18614	0.047251	0.45089	0.432223	COBL/EPS8	3
GO:190354	BP	GO:190354	positive reg	3/1035	15/18614	0.047251	0.45089	0.432223	SDC4/PDC	3
GO:190425	BP	GO:190425	regulation	3/1035	15/18614	0.047251	0.45089	0.432223	NR1D1/MA	3
GO:190450	BP	GO:190450	positive reg	3/1035	15/18614	0.047251	0.45089	0.432223	RUVBL2/IN	3
GO:000002	BP	GO:000002	ribosomal	4/1035	25/18614	0.047335	0.45089	0.432223	RRS1/RPF2	4
GO:000658	BP	GO:000658	melanin m	4/1035	25/18614	0.047335	0.45089	0.432223	MC1R/TRP	4
GO:002203	BP	GO:002203	corpus call	4/1035	25/18614	0.047335	0.45089	0.432223	CORO1C/F	4
GO:004598	BP	GO:004598	negative re	4/1035	25/18614	0.047335	0.45089	0.432223	DDIT4/CD	4
GO:004669	BP	GO:004669	decidualiza	4/1035	25/18614	0.047335	0.45089	0.432223	TPPP3/STC	4
GO:004688	BP	GO:004688	regulation	4/1035	25/18614	0.047335	0.45089	0.432223	NR3C1/ST	4
GO:005101	BP	GO:005101	barbed-en	4/1035	25/18614	0.047335	0.45089	0.432223	AVIL/RDX/	4
GO:007093	BP	GO:007093	3'-UTR-me	4/1035	25/18614	0.047335	0.45089	0.432223	RBM10/RB	4
GO:008601	BP	GO:008601	membrane	4/1035	25/18614	0.047335	0.45089	0.432223	ANK3/SCN	4
GO:001906	BP	GO:001906	virion asse	5/1035	36/18614	0.047477	0.45089	0.432223	VPS37B/PC	5
GO:007087	BP	GO:007087	regulation	5/1035	36/18614	0.047477	0.45089	0.432223	POMC/SOF	5
GO:014063	BP	GO:014063	inflammaso	5/1035	36/18614	0.047477	0.45089	0.432223	PRKD1/PLC	5
GO:190372	BP	GO:190372	regulation	5/1035	36/18614	0.047477	0.45089	0.432223	MTMR2/PH	5
GO:190533	BP	GO:190533	positive reg	5/1035	36/18614	0.047477	0.45089	0.432223	AR/PAX8/S	5
GO:000183	BP	GO:000183	embryonic	12/1035	126/18614	0.04792	0.45089	0.432223	COBL/TCTI	12
GO:003514	BP	GO:003514	tube forma	14/1035	154/18614	0.048107	0.45089	0.432223	COBL/TCTI	14
GO:003025	BP	GO:003025	lipid modif	17/1035	197/18614	0.048221	0.45089	0.432223	MTMR2/AC	17
GO:000611	BP	GO:000611	regulation	6/1035	48/18614	0.048846	0.45089	0.432223	JMJD8/DDI	6
GO:003252	BP	GO:003252	protein exi	6/1035	48/18614	0.048846	0.45089	0.432223	TMEM129/	6
GO:000610	BP	GO:000610	regulation	16/1035	183/18614	0.049093	0.45089	0.432223	LCMT1/HA	16
GO:000675	BP	GO:000675	ATP genera	9/1035	86/18614	0.049192	0.45089	0.432223	JMJD8/OGI	9
GO:007189	BP	GO:007189	DNA biosy	17/1035	198/18614	0.050145	0.45089	0.432223	MAP3K4/P	17
GO:004427	BP	GO:004427	sulfur comp	14/1035	155/18614	0.050304	0.45089	0.432223	PPT1/CHST	14
GO:000664	BP	GO:000664	triglyceride	10/1035	100/18614	0.051237	0.45089	0.432223	LDLR/RGN	10
GO:003530	BP	GO:003530	positive reg	7/1035	61/18614	0.051692	0.45089	0.432223	JAK2/MAG	7
GO:000700	BP	GO:000700	plasma me	15/1035	170/18614	0.051949	0.45089	0.432223	DMKN/AS	15
GO:004312	BP	GO:004312	regulation	21/1035	258/18614	0.052038	0.45089	0.432223	PRKD1/CA	21
GO:000858	BP	GO:000858	regulation	9/1035	87/18614	0.052288	0.45089	0.432223	TCTN1/IFT	9
GO:004870	BP	GO:004870	astrocyte c	9/1035	87/18614	0.052288	0.45089	0.432223	PRPF19/CL	9
GO:002261	BP	GO:002261	DNA strand	5/1035	37/18614	0.052486	0.45089	0.432223	RUVBL2/IN	5
GO:003107	BP	GO:003107	embryonic	5/1035	37/18614	0.052486	0.45089	0.432223	WNT16/PA	5
GO:003411	BP	GO:003411	regulation	5/1035	37/18614	0.052486	0.45089	0.432223	JAK2/ANK3	5
GO:004427	BP	GO:004427	sulfur comp	5/1035	37/18614	0.052486	0.45089	0.432223	HYAL1/AC	5
GO:004853	BP	GO:004853	spleen dev	5/1035	37/18614	0.052486	0.45089	0.432223	NFKBIZ/TR	5
GO:006014	BP	GO:006014	regulation	5/1035	37/18614	0.052486	0.45089	0.432223	FOCAD/PU	5
GO:007252	BP	GO:007252	pyrimidine	5/1035	37/18614	0.052486	0.45089	0.432223	NME7/CD	5
GO:200027	BP	GO:200027	negative re	5/1035	37/18614	0.052486	0.45089	0.432223	PINX1/EXC	5
GO:190357	BP	GO:190357	regulation	8/1035	74/18614	0.05259	0.45089	0.432223	JMJD8/DDI	8
GO:000698	BP	GO:000698	response t	13/1035	142/18614	0.052918	0.45089	0.432223	EIF2S1/UBI	13
GO:000926	BP	GO:000926	ribonucleo	6/1035	49/18614	0.053158	0.45089	0.432223	ACOT7/NU	6
GO:004646	BP	GO:004646	neutral lipi	6/1035	49/18614	0.053158	0.45089	0.432223	AVIL/LDLR	6
GO:004646	BP	GO:004646	acylglycero	6/1035	49/18614	0.053158	0.45089	0.432223	AVIL/LDLR	6
GO:007147	BP	GO:007147	cellular res	6/1035	49/18614	0.053158	0.45089	0.432223	CAPN3/TSI	6
GO:000654	BP	GO:000654	glutamine	4/1035	26/18614	0.053571	0.45089	0.432223	NIT2/ASL/	4
GO:003611	BP	GO:003611	response t	4/1035	26/18614	0.053571	0.45089	0.432223	HYAL1/HA	4
GO:004455	BP	GO:004455	secondary	4/1035	26/18614	0.053571	0.45089	0.432223	MC1R/TRP	4
GO:200005	BP	GO:200005	regulation	4/1035	26/18614	0.053571	0.45089	0.432223	DAAM2/CS	4
GO:000207	BP	GO:000207	optic cup r	2/1035	7/18614	0.053821	0.45089	0.432223	WNT16/AL	2
GO:000654	BP	GO:000654	isoleucine	2/1035	7/18614	0.053821	0.45089	0.432223	ACAT1/BC	2
GO:000665	BP	GO:000665	CDP-choli	2/1035	7/18614	0.053821	0.45089	0.432223	PCYT1B/CI	2
GO:000707	BP	GO:000707	mitotic nuc	2/1035	7/18614	0.053821	0.45089	0.432223	VRK1/LPIN	2
GO:000717	BP	GO:000717	activation	2/1035	7/18614	0.053821	0.45089	0.432223	ADRB2/PIL	2
GO:001053	BP	GO:001053	positive reg	2/1035	7/18614	0.053821	0.45089	0.432223	IL6R/AGT	2
GO:001718	BP	GO:001718	peptidyl-d	2/1035	7/18614	0.053821	0.45089	0.432223	DPH5/DPH	2
GO:001718	BP	GO:001718	peptidyl-d	2/1035	7/18614	0.053821	0.45089	0.432223	DPH5/DPH	2
GO:002164	BP	GO:002164	vestibuloc	2/1035	7/18614	0.053821	0.45089	0.432223	TIFAB/DCA	2
GO:002187	BP	GO:002187	forebrain n	2/1035	7/18614	0.053821	0.45089	0.432223	TBR1/PAX6	2
GO:003257	BP	GO:003257	response t	2/1035	7/18614	0.053821	0.45089	0.432223	F5/GAS6	2
GO:003335	BP	GO:003335	S-adenosy	2/1035	7/18614	0.053821	0.45089	0.432223	MTRR/AHC	2
GO:003414	BP	GO:003414	positive reg	2/1035	7/18614	0.053821	0.45089	0.432223	PTPN22/PE	2
GO:004003	BP	GO:004003	snRNA mo	2/1035	7/18614	0.053821	0.45089	0.432223	NHP2/MET	2
GO:004591	BP	GO:004591	positive reg	2/1035	7/18614	0.053821	0.45089	0.432223	ABAT/NPY	2
GO:004596	BP	GO:004596	positive reg	2/1035	7/18614	0.053821	0.45089	0.432223	ABAT/NPY	2
GO:004683	BP	GO:004683	glycolipid t	2/1035	7/18614	0.053821	0.45089	0.432223	RFT1/PSAP	2
GO:004854	BP	GO:004854	positive reg	2/1035	7/18614	0.053821	0.45089	0.432223	PPT1/AXL	2
GO:004885	BP	GO:004885	formation	2/1035	7/18614	0.053821	0.45089	0.432223	PAX6/SSBF	2
GO:006172	BP	GO:006172	lipophagy	2/1035	7/18614	0.053821	0.45089	0.432223	ADRB2/SP	2
GO:007102	BP	GO:007102	nuclear ncl	2/1035	7/18614	0.053821	0.45089	0.432223	EXOSC10/	2
GO:007103	BP	GO:007103	nuclear po	2/1035	7/18614	0.053821	0.45089	0.432223	EXOSC10/	2
GO:007103	BP	GO:007103	nuclear po	2/1035	7/18614	0.053821	0.45089	0.432223	EXOSC10/	2
GO:007104	BP	GO:007104	nuclear po	2/1035	7/18614	0.053821	0.45089	0.432223	EXOSC10/	2
GO:009052	BP	GO:009052	glomerular	2/1035	7/18614	0.053821	0.45089	0.432223	KANK1/DA	2
GO:009701	BP	GO:009701	renal prote	2/1035	7/18614	0.053821	0.45089	0.432223	GAS6/GSN	2
GO:009729	BP	GO:009729	XMP metal	2/1035	7/18614	0.053821	0.45089	0.432223	PAICS/PFA	2
GO:009729	BP	GO:009729	XMP biosy	2/1035	7/18614	0.053821	0.45089	0.432223	PAICS/PFA	2
GO:009729	BP	GO:009729	'de novo' X	2/1035	7/18614	0.053821	0.45089	0.432223	PAICS/PFA	2
GO:010635	BP	GO:010635	tRNA surve	2/1035	7/18614	0.053821	0.45089	0.432223	EXOSC10/	2
GO:190002	BP	GO:190002	negative re	2/1035	7/18614	0.053821	0.45089	0.432223	KANK1/AR	2
GO:190107	BP	GO:190107	negative re	2/1035	7/18614	0.053821	0.45089	0.432223	TIFAB/DCA	2
GO:190246	BP	GO:190246	regulation	2/1035	7/18614	0.053821	0.45089	0.432223	LTBP3/CITI	2
GO:190479	BP	GO:190479	regulation	2/1035	7/18614	0.053821	0.45089	0.432223	FOXC1/PA	2
GO:200039	BP	GO:200039	positive reg	2/1035	7/18614	0.053821	0.45089	0.432223	CORO1C/R	2
GO:200079	BP	GO:200079	regulation	2/1035	7/18614	0.053821	0.45089	0.432223	SOX9/NFIE	2
GO:190290	BP	GO:190290	positive reg	15/1035	171/18614	0.054157	0.45089	0.432223	ARHGEF5/	15
GO:001610	BP	GO:001610	diterpenoi	9/1035	88/18614	0.055508	0.45089	0.432223	ALDH8A1/	9
GO:003105	BP	GO:003105	positive reg	9/1035	88/18614	0.055508	0.45089	0.432223	MUC1/PRK	9
GO:003327	BP	GO:003327	response t	9/1035	88/18614	0.055508	0.45089	0.432223	F5/NFKBIZ	9
GO:009030	BP	GO:009030	nucleic aci	20/1035	245/18614	0.055521	0.45089	0.432223	RCL1/RRS1	20
GO:004350	BP	GO:004350	regulation	7/1035	62/18614	0.055603	0.45089	0.432223	ARHGEF5/	7
GO:000016	BP	GO:000016	phosphore	1/1035	1/18614	0.055603	0.45089	0.432223	MMS19	1
GO:000071	BP	GO:000071	nucleotide-	1/1035	1/18614	0.055603	0.45089	0.432223	XPA	1
GO:000210	BP	GO:000210	tRNA wobl	1/1035	1/18614	0.055603	0.45089	0.432223	ADAT2	1
GO:000233	BP	GO:000233	transitional	1/1035	1/18614	0.055603	0.45089	0.432223	TRAF3IP2	1
GO:000245	BP	GO:000245	peripheral	1/1035	1/18614	0.055603	0.45089	0.432223	HLA-G	1
GO:000247	BP	GO:000247	antigen pro	1/1035	1/18614	0.055603	0.45089	0.432223	IKBKB	1
GO:000314	BP	GO:000314	embryonic	1/1035	1/18614	0.055603	0.45089	0.432223	HAND1	1
GO:000332	BP	GO:000332	pancreatic	1/1035	1/18614	0.055603	0.45089	0.432223	PAX6	1
GO:000618	BP	GO:000618	dGDP bios	1/1035	1/18614	0.055603	0.45089	0.432223	GUK1	1
GO:000653	BP	GO:000653	asparagine	1/1035	1/18614	0.055603	0.45089	0.432223	ASRGL1	1
GO:000653	BP	GO:000653	glutamate	1/1035	1/18614	0.055603	0.45089	0.432223	ADHFE1	1
GO:000671	BP	GO:000671	glucocortic	1/1035	1/18614	0.055603	0.45089	0.432223	YWHAH	1
GO:000859	BP	GO:000859	regulation	1/1035	1/18614	0.055603	0.45089	0.432223	DKK1	1
GO:000918	BP	GO:000918	purine deo	1/1035	1/18614	0.055603	0.45089	0.432223	NUDT18	1
GO:000919	BP	GO:000919	deoxyribor	1/1035	1/18614	0.055603	0.45089	0.432223	NUDT18	1
GO:001488	BP	GO:001488	transition b	1/1035	1/18614	0.055603	0.45089	0.432223	GTF2IRD1	1
GO:001610	BP	GO:001610	triterpenoi	1/1035	1/18614	0.055603	0.45089	0.432223	LSS	1
GO:001910	BP	GO:001910	male soma	1/1035	1/18614	0.055603	0.45089	0.432223	AR	1
GO:001925	BP	GO:001925	glucose 1-	1/1035	1/18614	0.055603	0.45089	0.432223	UGP2	1
GO:002150	BP	GO:002150	prechordal	1/1035	1/18614	0.055603	0.45089	0.432223	SSBP3	1
GO:002154	BP	GO:002154	midbrain-h	1/1035	1/18614	0.055603	0.45089	0.432223	SSBP3	1
GO:002176	BP	GO:002176	nucleus ac	1/1035	1/18614	0.055603	0.45089	0.432223	ALDH1A3	1
GO:002190	BP	GO:002190	forebrain-r	1/1035	1/18614	0.055603	0.45089	0.432223	PAX6	1
GO:002191	BP	GO:002191	BMP signal	1/1035	1/18614	0.055603	0.45089	0.432223	CHRD	1
GO:002195	BP	GO:002195	central ner	1/1035	1/18614	0.055603	0.45089	0.432223	TCTN1	1
GO:003195	BP	GO:003195	mineralocc	1/1035	1/18614	0.055603	0.45089	0.432223	JAK2	1
GO:003247	BP	GO:003247	otolith forr	1/1035	1/18614	0.055603	0.45089	0.432223	SLC44A4	1
GO:003288	BP	GO:003288	regulation	1/1035	1/18614	0.055603	0.45089	0.432223	NUMA1	1
GO:003288	BP	GO:003288	regulation	1/1035	1/18614	0.055603	0.45089	0.432223	NUMA1	1
GO:003290	BP	GO:003290	regulation	1/1035	1/18614	0.055603	0.45089	0.432223	NPY	1
GO:003331	BP	GO:003331	UDP-D-xy	1/1035	1/18614	0.055603	0.45089	0.432223	UXS1	1
GO:003332	BP	GO:003332	UDP-D-xy	1/1035	1/18614	0.055603	0.45089	0.432223	UXS1	1
GO:003334	BP	GO:003334	asparagine	1/1035	1/18614	0.055603	0.45089	0.432223	ASRGL1	1
GO:003405	BP	GO:003405	programm	1/1035	1/18614	0.055603	0.45089	0.432223	JAK2	1
GO:003420	BP	GO:003420	glycolipid t	1/1035	1/18614	0.055603	0.45089	0.432223	RFT1	1
GO:003564	BP	GO:003564	phosphoar	1/1035	1/18614	0.055603	0.45089	0.432223	PTPN22	1
GO:003573	BP	GO:003573	nitric oxide	1/1035	1/18614	0.055603	0.45089	0.432223	GSTP1	1
GO:003611	BP	GO:003611	long-chain	1/1035	1/18614	0.055603	0.45089	0.432223	ACOT7	1
GO:003616	BP	GO:003616	oxytocin pr	1/1035	1/18614	0.055603	0.45089	0.432223	POMC	1
GO:003625	BP	GO:003625	response t	1/1035	1/18614	0.055603	0.45089	0.432223	GLDC	1
GO:003817	BP	GO:003817	interleukin-	1/1035	1/18614	0.055603	0.45089	0.432223	TRAF3IP2	1
GO:004241	BP	GO:004241	carnitine ca	1/1035	1/18614	0.055603	0.45089	0.432223	ACADL	1
GO:004272	BP	GO:004272	flavin-cont	1/1035	1/18614	0.055603	0.45089	0.432223	VCP	1
GO:004286	BP	GO:004286	antisense R	1/1035	1/18614	0.055603	0.45089	0.432223	EXOSC10	1
GO:004332	BP	GO:004332	negative re	1/1035	1/18614	0.055603	0.45089	0.432223	HLA-F	1
GO:004340	BP	GO:004340	glucocortic	1/1035	1/18614	0.055603	0.45089	0.432223	NR3C1	1
GO:004485	BP	GO:004485	plasma me	1/1035	1/18614	0.055603	0.45089	0.432223	GSN	1
GO:004518	BP	GO:004518	zonula adh	1/1035	1/18614	0.055603	0.45089	0.432223	DLG5	1
GO:004581	BP	GO:004581	positive reg	1/1035	1/18614	0.055603	0.45089	0.432223	DKK1	1
GO:004596	BP	GO:004596	negative re	1/1035	1/18614	0.055603	0.45089	0.432223	TMPRSS4	1
GO:004605	BP	GO:004605	dADP cata	1/1035	1/18614	0.055603	0.45089	0.432223	NUDT18	1
GO:004606	BP	GO:004606	dGDP cata	1/1035	1/18614	0.055603	0.45089	0.432223	NUDT18	1
GO:004609	BP	GO:004609	deoxycytid	1/1035	1/18614	0.055603	0.45089	0.432223	TK2	1
GO:004658	BP	GO:004658	negative re	1/1035	1/18614	0.055603	0.45089	0.432223	FXYD5	1
GO:004674	BP	GO:004674	viral capsic	1/1035	1/18614	0.055603	0.45089	0.432223	IST1	1
GO:004807	BP	GO:004807	positive reg	1/1035	1/18614	0.055603	0.45089	0.432223	HPS4	1
GO:004857	BP	GO:004857	short-day	1/1035	1/18614	0.055603	0.45089	0.432223	NPY	1
GO:005504	BP	GO:005504	anastral sp	1/1035	1/18614	0.055603	0.45089	0.432223	NUMA1	1
GO:006011	BP	GO:006011	vestibular r	1/1035	1/18614	0.055603	0.45089	0.432223	PLS1	1
GO:006012	BP	GO:006012	vestibular r	1/1035	1/18614	0.055603	0.45089	0.432223	PLS1	1
GO:006037	BP	GO:006037	negative re	1/1035	1/18614	0.055603	0.45089	0.432223	ZFPM1	1
GO:006045	BP	GO:006045	positive reg	1/1035	1/18614	0.055603	0.45089	0.432223	OXT	1
GO:006051	BP	GO:006051	prostate in	1/1035	1/18614	0.055603	0.45089	0.432223	AR	1
GO:006051	BP	GO:006051	prostate fie	1/1035	1/18614	0.055603	0.45089	0.432223	AR	1
GO:006051	BP	GO:006051	epithelial c	1/1035	1/18614	0.055603	0.45089	0.432223	SOX9	1
GO:006052	BP	GO:006052	activation	1/1035	1/18614	0.055603	0.45089	0.432223	AR	1
GO:006078	BP	GO:006078	regulation	1/1035	1/18614	0.055603	0.45089	0.432223	SOX9	1
GO:006193	BP	GO:006193	protein loc	1/1035	1/18614	0.055603	0.45089	0.432223	AP4B1	1
GO:006203	BP	GO:006203	sensory pe	1/1035	1/18614	0.055603	0.45089	0.432223	GRIK2	1
GO:007018	BP	GO:007018	mitochond	1/1035	1/18614	0.055603	0.45089	0.432223	YARS2	1
GO:007048	BP	GO:007048	nuclear-tra	1/1035	1/18614	0.055603	0.45089	0.432223	PELO	1
GO:007062	BP	GO:007062	zymogen g	1/1035	1/18614	0.055603	0.45089	0.432223	SDF4	1
GO:007065	BP	GO:007065	nonfunctio	1/1035	1/18614	0.055603	0.45089	0.432223	PELO	1
GO:007075	BP	GO:007075	interleukin-	1/1035	1/18614	0.055603	0.45089	0.432223	JAK2	1
GO:007089	BP	GO:007089	RNA polym	1/1035	1/18614	0.055603	0.45089	0.432223	BDP1	1
GO:007104	BP	GO:007104	nuclear po	1/1035	1/18614	0.055603	0.45089	0.432223	EXOSC10	1
GO:007104	BP	GO:007104	antisense R	1/1035	1/18614	0.055603	0.45089	0.432223	EXOSC10	1
GO:007121	BP	GO:007121	cellular res	1/1035	1/18614	0.055603	0.45089	0.432223	CDA	1
GO:007124	BP	GO:007124	cellular res	1/1035	1/18614	0.055603	0.45089	0.432223	DNM2	1
GO:007170	BP	GO:007170	regulation	1/1035	1/18614	0.055603	0.45089	0.432223	DNAJC27	1
GO:007238	BP	GO:007238	flavin aden	1/1035	1/18614	0.055603	0.45089	0.432223	VCP	1
GO:007270	BP	GO:007270	response t	1/1035	1/18614	0.055603	0.45089	0.432223	ZNF16	1
GO:007270	BP	GO:007270	cellular res	1/1035	1/18614	0.055603	0.45089	0.432223	ZNF16	1
GO:009003	BP	GO:009003	regulation	1/1035	1/18614	0.055603	0.45089	0.432223	STUB1	1
GO:009003	BP	GO:009003	positive reg	1/1035	1/18614	0.055603	0.45089	0.432223	STUB1	1
GO:009022	BP	GO:009022	regulation	1/1035	1/18614	0.055603	0.45089	0.432223	RANGRF	1
GO:010608	BP	GO:010608	negative re	1/1035	1/18614	0.055603	0.45089	0.432223	SEMA6A	1
GO:011001	BP	GO:011001	protein loc	1/1035	1/18614	0.055603	0.45089	0.432223	TRAF3IP2	1
GO:012016	BP	GO:012016	detection	1/1035	1/18614	0.055603	0.45089	0.432223	GRIK2	1
GO:014045	BP	GO:014045	protein agg	1/1035	1/18614	0.055603	0.45089	0.432223	WSB1	1
GO:014046	BP	GO:014046	iron-sulfur	1/1035	1/18614	0.055603	0.45089	0.432223	ABCB7	1
GO:014066	BP	GO:014066	regulation	1/1035	1/18614	0.055603	0.45089	0.432223	POMC	1
GO:014066	BP	GO:014066	positive reg	1/1035	1/18614	0.055603	0.45089	0.432223	POMC	1
GO:190014	BP	GO:190014	negative re	1/1035	1/18614	0.055603	0.45089	0.432223	GAS6	1
GO:190014	BP	GO:190014	positive reg	1/1035	1/18614	0.055603	0.45089	0.432223	AKAP12	1
GO:190048	BP	GO:190048	regulation	1/1035	1/18614	0.055603	0.45089	0.432223	AVIL	1
GO:190082	BP	GO:190082	positive reg	1/1035	1/18614	0.055603	0.45089	0.432223	ANK3	1
GO:190129	BP	GO:190129	negative re	1/1035	1/18614	0.055603	0.45089	0.432223	DKK1	1
GO:190130	BP	GO:190130	negative re	1/1035	1/18614	0.055603	0.45089	0.432223	INSIG1	1
GO:190203	BP	GO:190203	positive reg	1/1035	1/18614	0.055603	0.45089	0.432223	FOXC1	1
GO:190236	BP	GO:190236	regulation	1/1035	1/18614	0.055603	0.45089	0.432223	NUMA1	1
GO:190236	BP	GO:190236	positive reg	1/1035	1/18614	0.055603	0.45089	0.432223	NUMA1	1
GO:190249	BP	GO:190249	iron-sulfur	1/1035	1/18614	0.055603	0.45089	0.432223	ABCB7	1
GO:190261	BP	GO:190261	regulation	1/1035	1/18614	0.055603	0.45089	0.432223	DKK3	1
GO:190261	BP	GO:190261	negative re	1/1035	1/18614	0.055603	0.45089	0.432223	DKK3	1
GO:190262	BP	GO:190262	assembly c	1/1035	1/18614	0.055603	0.45089	0.432223	EIF6	1
GO:190272	BP	GO:190272	positive reg	1/1035	1/18614	0.055603	0.45089	0.432223	ABAT	1
GO:190272	BP	GO:190272	positive reg	1/1035	1/18614	0.055603	0.45089	0.432223	JAK2	1
GO:190272	BP	GO:190272	positive reg	1/1035	1/18614	0.055603	0.45089	0.432223	JAK2	1
GO:190284	BP	GO:190284	positive reg	1/1035	1/18614	0.055603	0.45089	0.432223	NUMA1	1
GO:190286	BP	GO:190286	propionyl-	1/1035	1/18614	0.055603	0.45089	0.432223	ACAT1	1
GO:190300	BP	GO:190300	positive reg	1/1035	1/18614	0.055603	0.45089	0.432223	VCP	1
GO:190301	BP	GO:190301	negative re	1/1035	1/18614	0.055603	0.45089	0.432223	RGN	1
GO:190330	BP	GO:190330	regulation	1/1035	1/18614	0.055603	0.45089	0.432223	JMJD8	1
GO:190332	BP	GO:190332	regulation	1/1035	1/18614	0.055603	0.45089	0.432223	ABCB7	1
GO:190333	BP	GO:190333	positive reg	1/1035	1/18614	0.055603	0.45089	0.432223	ABCB7	1
GO:190344	BP	GO:190344	response t	1/1035	1/18614	0.055603	0.45089	0.432223	GLDC	1
GO:190345	BP	GO:190345	lactate cat	1/1035	1/18614	0.055603	0.45089	0.432223	LDHD	1
GO:190361	BP	GO:190361	negative re	1/1035	1/18614	0.055603	0.45089	0.432223	RGN	1
GO:190362	BP	GO:190362	protein loc	1/1035	1/18614	0.055603	0.45089	0.432223	SPATA7	1
GO:190362	BP	GO:190362	regulation	1/1035	1/18614	0.055603	0.45089	0.432223	RGN	1
GO:190362	BP	GO:190362	positive reg	1/1035	1/18614	0.055603	0.45089	0.432223	RGN	1
GO:190363	BP	GO:190363	negative re	1/1035	1/18614	0.055603	0.45089	0.432223	RGN	1
GO:190363	BP	GO:190363	positive reg	1/1035	1/18614	0.055603	0.45089	0.432223	AIMP2	1
GO:190363	BP	GO:190363	regulation	1/1035	1/18614	0.055603	0.45089	0.432223	RGN	1
GO:190363	BP	GO:190363	negative re	1/1035	1/18614	0.055603	0.45089	0.432223	RGN	1
GO:190373	BP	GO:190373	regulation	1/1035	1/18614	0.055603	0.45089	0.432223	LPIN1	1
GO:190374	BP	GO:190374	negative re	1/1035	1/18614	0.055603	0.45089	0.432223	LPIN1	1
GO:190377	BP	GO:190377	positive reg	1/1035	1/18614	0.055603	0.45089	0.432223	PSAP	1
GO:190390	BP	GO:190390	regulation	1/1035	1/18614	0.055603	0.45089	0.432223	GSN	1
GO:190394	BP	GO:190394	negative re	1/1035	1/18614	0.055603	0.45089	0.432223	DEPTOR	1
GO:190404	BP	GO:190404	regulation	1/1035	1/18614	0.055603	0.45089	0.432223	MTRR	1
GO:190404	BP	GO:190404	negative re	1/1035	1/18614	0.055603	0.45089	0.432223	MTRR	1
GO:190410	BP	GO:190410	protein loc	1/1035	1/18614	0.055603	0.45089	0.432223	INVS	1
GO:190456	BP	GO:190456	response t	1/1035	1/18614	0.055603	0.45089	0.432223	LPAR1	1
GO:190456	BP	GO:190456	cellular res	1/1035	1/18614	0.055603	0.45089	0.432223	LPAR1	1
GO:190470	BP	GO:190470	Wnt-Frizzle	1/1035	1/18614	0.055603	0.45089	0.432223	DKK1	1

GO:000863	BP	GO:000863	intrinsic ap	10/902	103/18614	0.027627	0.328448	0.2948	PHLDA3/R	10
GO:004563	BP	GO:004563	positive reg	10/902	103/18614	0.027627	0.328448	0.2948	ZFP36L1/R	10
GO:004814	BP	GO:004814	fibroblast p	10/902	103/18614	0.027627	0.328448	0.2948	CKS2/MED	10
GO:003579	BP	GO:003579	platelet-de	3/902	14/18614	0.027657	0.328448	0.2948	PTPN2/PD	3
GO:005093	BP	GO:005093	induction c	3/902	14/18614	0.027657	0.328448	0.2948	VEGFC/VE	3
GO:006033	BP	GO:006033	regulation	3/902	14/18614	0.027657	0.328448	0.2948	NR1H3/PT	3
GO:006033	BP	GO:006033	regulation	3/902	14/18614	0.027657	0.328448	0.2948	NR1H3/PT	3
GO:190389	BP	GO:190389	regulation	3/902	14/18614	0.027657	0.328448	0.2948	FICD/HSPA	3
GO:200028	BP	GO:200028	positive reg	3/902	14/18614	0.027657	0.328448	0.2948	GATA6/CT	3
GO:001701	BP	GO:001701	regulation	13/902	148/18614	0.027799	0.329549	0.295789	EMILIN1/Z	13
GO:005170	BP	GO:005170	biological	11/902	118/18614	0.028092	0.331497	0.297537	PPIB/ZDHH	11
GO:005189	BP	GO:005189	positive reg	11/902	118/18614	0.028092	0.331497	0.297537	GDF15/FA	11
GO:007122	BP	GO:007122	cellular res	9/902	89/18614	0.028306	0.331497	0.297537	SESN3/CPE	9
GO:007207	BP	GO:007207	nephron tu	8/902	75/18614	0.028395	0.331497	0.297537	GREM1/IRX	8
GO:003368	BP	GO:003368	osteoblast	5/902	36/18614	0.028655	0.331497	0.297537	GREM1/NF	5
GO:003367	BP	GO:003367	positive reg	30/902	430/18614	0.02933	0.331497	0.297537	GREM1/CC	30
GO:003166	BP	GO:003166	cellular res	20/902	261/18614	0.029364	0.331497	0.297537	SESN3/PM	20
GO:190018	BP	GO:190018	regulation	12/902	134/18614	0.029568	0.331497	0.297537	TRIM8/SU	12
GO:003248	BP	GO:003248	positive reg	7/902	62/18614	0.029786	0.331497	0.297537	IRF1/CHUK	7
GO:003109	BP	GO:003109	stress-acti	19/902	245/18614	0.029789	0.331497	0.297537	MAP4K4/IT	19
GO:005079	BP	GO:005079	regulation	14/902	165/18614	0.029844	0.331497	0.297537	TRIM8/TRI	14
GO:004852	BP	GO:004852	negative re	9/902	90/18614	0.030153	0.331497	0.297537	TRIM8/OAS	9
GO:006039	BP	GO:006039	SMAD prot	9/902	90/18614	0.030153	0.331497	0.297537	GDF15/TG	9
GO:004877	BP	GO:004877	tissue remo	15/902	181/18614	0.030227	0.331497	0.297537	GREM1/ZN	15
GO:009872	BP	GO:009872	maintenan	15/902	181/18614	0.030227	0.331497	0.297537	TRIM8/FZD	15
GO:003139	BP	GO:003139	regulation	17/902	213/18614	0.03032	0.331497	0.297537	TRIB3/BAG	17
GO:004647	BP	GO:004647	phosphatic	8/902	76/18614	0.030441	0.331497	0.297537	NR1H3/MF	8
GO:006135	BP	GO:006135	neural prec	13/902	150/18614	0.030573	0.331497	0.297537	VEGFC/WD	13
GO:003031	BP	GO:003031	melanocyt	4/902	25/18614	0.030766	0.331497	0.297537	BLOC1S6/	4
GO:004564	BP	GO:004564	regulation	4/902	25/18614	0.030766	0.331497	0.297537	ID2/PTPN2	4
GO:005092	BP	GO:005092	positive reg	4/902	25/18614	0.030766	0.331497	0.297537	VEGFC/VE	4
GO:007042	BP	GO:007042	nucleotide	4/902	25/18614	0.030766	0.331497	0.297537	ITCH/TNFA	4
GO:190506	BP	GO:190506	regulation	4/902	25/18614	0.030766	0.331497	0.297537	ENG/SOD2	4
GO:000316	BP	GO:000316	bundle of	2/902	6/18614	0.030889	0.331497	0.297537	IRX3/ID2	2
GO:000670	BP	GO:000670	progestero	2/902	6/18614	0.030889	0.331497	0.297537	STARD3/D	2
GO:001003	BP	GO:001003	meiotic ch	2/902	6/18614	0.030889	0.331497	0.297537	SMC4/SMC	2
GO:001570	BP	GO:001570	iodide tran	2/902	6/18614	0.030889	0.331497	0.297537	SLC5A5/SL	2
GO:003202	BP	GO:003202	ISG15-pro	2/902	6/18614	0.030889	0.331497	0.297537	UBE2L6/IS	2
GO:003291	BP	GO:003291	negative re	2/902	6/18614	0.030889	0.331497	0.297537	GATA6/TSI	2
GO:003359	BP	GO:003359	response t	2/902	6/18614	0.030889	0.331497	0.297537	SOD2/RUN	2
GO:003596	BP	GO:003596	COPI-coat	2/902	6/18614	0.030889	0.331497	0.297537	TMED2/AR	2
GO:004563	BP	GO:004563	regulation	2/902	6/18614	0.030889	0.331497	0.297537	ZEB2/KITL	2
GO:004607	BP	GO:004607	dTMP meta	2/902	6/18614	0.030889	0.331497	0.297537	TYMS/TYM	2
GO:004816	BP	GO:004816	fused antru	2/902	6/18614	0.030889	0.331497	0.297537	TNFAIP6/P	2
GO:004820	BP	GO:004820	Golgi trans	2/902	6/18614	0.030889	0.331497	0.297537	TMED2/AR	2
GO:004820	BP	GO:004820	COPI coati	2/902	6/18614	0.030889	0.331497	0.297537	TMED2/AR	2
GO:005103	BP	GO:005103	tRNA trans	2/902	6/18614	0.030889	0.331497	0.297537	XPOT/SSB	2
GO:006039	BP	GO:006039	regulation	2/902	6/18614	0.030889	0.331497	0.297537	GDF15/LEF	2
GO:006051	BP	GO:006051	type II pne	2/902	6/18614	0.030889	0.331497	0.297537	GATA6/RB	2
GO:006075	BP	GO:006075	positive reg	2/902	6/18614	0.030889	0.331497	0.297537	VEGFC/VE	2
GO:006134	BP	GO:006134	cell adhesi	2/902	6/18614	0.030889	0.331497	0.297537	TGFB2/FLR	2
GO:007168	BP	GO:007168	response t	2/902	6/18614	0.030889	0.331497	0.297537	CYP1B1/CT	2
GO:007256	BP	GO:007256	chemokine	2/902	6/18614	0.030889	0.331497	0.297537	TIRAP/MYD	2
GO:009028	BP	GO:009028	regulation	2/902	6/18614	0.030889	0.331497	0.297537	GREM1/TN	2
GO:009740	BP	GO:009740	neuroblast	2/902	6/18614	0.030889	0.331497	0.297537	EDNRB/TN	2
GO:009870	BP	GO:009870	carbohydra	2/902	6/18614	0.030889	0.331497	0.297537	SLC2A3/SL	2
GO:012018	BP	GO:012018	regulation	2/902	6/18614	0.030889	0.331497	0.297537	MAP4K4/D	2
GO:012018	BP	GO:012018	positive reg	2/902	6/18614	0.030889	0.331497	0.297537	MAP4K4/D	2
GO:014027	BP	GO:014027	hexose imp	2/902	6/18614	0.030889	0.331497	0.297537	SLC2A3/SL	2
GO:190023	BP	GO:190023	regulation	2/902	6/18614	0.030889	0.331497	0.297537	FGF9/DNM	2
GO:190153	BP	GO:190153	positive reg	2/902	6/18614	0.030889	0.331497	0.297537	ZNHIT1/KI	2
GO:190295	BP	GO:190295	regulation	2/902	6/18614	0.030889	0.331497	0.297537	ISCU/SNC	2
GO:190321	BP	GO:190321	negative re	2/902	6/18614	0.030889	0.331497	0.297537	BAG4/LMA	2
GO:190396	BP	GO:190396	regulation	2/902	6/18614	0.030889	0.331497	0.297537	PTPN2/CSF	2
GO:190397	BP	GO:190397	regulation	2/902	6/18614	0.030889	0.331497	0.297537	PTPN2/CSF	2
GO:190438	BP	GO:190438	response t	2/902	6/18614	0.030889	0.331497	0.297537	GCLM/GCL	2
GO:190500	BP	GO:190500	regulation	2/902	6/18614	0.030889	0.331497	0.297537	TGFB2/EN	2
GO:199011	BP	GO:199011	ribosome-	2/902	6/18614	0.030889	0.331497	0.297537	ASCC3/LTN	2
GO:200033	BP	GO:200033	regulation	2/902	6/18614	0.030889	0.331497	0.297537	TIRAP/MYD	2
GO:004320	BP	GO:004320	response t	11/902	120/18614	0.031277	0.334591	0.300313	SESN3/CPE	11
GO:005110	BP	GO:005110	regulation	11/902	120/18614	0.031277	0.334591	0.300313	ZNF675/SU	11
GO:190188	BP	GO:190188	regulation	17/902	214/18614	0.031513	0.335742	0.301347	GREM1/M	17
GO:009950	BP	GO:009950	synaptic ve	16/902	198/18614	0.031617	0.335742	0.301347	CADPS2/B	16
GO:005123	BP	GO:005123	maintenan	25/902	347/18614	0.031634	0.335742	0.301347	NR1H3/LM	25
GO:001405	BP	GO:001405	regulation	5/902	37/18614	0.031856	0.335742	0.301347	SYT8/HTR2	5
GO:003817	BP	GO:003817	neurotroph	5/902	37/18614	0.031856	0.335742	0.301347	CYFIP2/RA	5
GO:004316	BP	GO:004316	ubiquitin-c	5/902	37/18614	0.031856	0.335742	0.301347	CHMP5/ST	5
GO:004592	BP	GO:004592	negative re	5/902	37/18614	0.031856	0.335742	0.301347	RAP1A/SN	5
GO:190384	BP	GO:190384	regulation	13/902	151/18614	0.032032	0.335742	0.301347	EMILIN1/Z	13
GO:000194	BP	GO:000194	hair follicle	9/902	91/18614	0.032083	0.335742	0.301347	TGFB2/DL	9
GO:006039	BP	GO:006039	regulation	7/902	63/18614	0.032163	0.335742	0.301347	GREM1/EN	7
GO:007005	BP	GO:007005	intrinsic ap	7/902	63/18614	0.032163	0.335742	0.301347	TRIB3/PMA	7
GO:003019	BP	GO:003019	extracellula	23/902	314/18614	0.032448	0.335742	0.301347	GREM1/EN	23
GO:000961	BP	GO:000961	response t	17/902	215/18614	0.032742	0.335742	0.301347	IRF1/CHI3L	17
GO:005164	BP	GO:005164	vesicle loca	17/902	215/18614	0.032742	0.335742	0.301347	BLOC1S6/	17
GO:009877	BP	GO:009877	skin epider	11/902	121/18614	0.032963	0.335742	0.301347	GATA6/TG	11
GO:000669	BP	GO:000669	prostanoid	6/902	50/18614	0.032963	0.335742	0.301347	PTGDS/PT	6
GO:000669	BP	GO:000669	prostaglan	6/902	50/18614	0.032963	0.335742	0.301347	PTGDS/PT	6
GO:004408	BP	GO:004408	regulation	6/902	50/18614	0.032963	0.335742	0.301347	SNX18/IFT	6
GO:004854	BP	GO:004854	digestive tr	6/902	50/18614	0.032963	0.335742	0.301347	SOX11/SH	6
GO:000171	BP	GO:000171	endoderma	3/902	15/18614	0.033354	0.335742	0.301347	GATA6/CT	3
GO:000341	BP	GO:000341	chondrocy	3/902	15/18614	0.033354	0.335742	0.301347	SERPINH1/	3
GO:000620	BP	GO:000620	pyrimidine	3/902	15/18614	0.033354	0.335742	0.301347	CMPK1/TY	3
GO:003290	BP	GO:003290	transformi	3/902	15/18614	0.033354	0.335742	0.301347	GATA6/LU	3
GO:004253	BP	GO:004253	negative re	3/902	15/18614	0.033354	0.335742	0.301347	SH2B3/PTF	3
GO:004299	BP	GO:004299	cytoplasmi	3/902	15/18614	0.033354	0.335742	0.301347	NFKBIE/PS	3
GO:004807	BP	GO:004807	regulation	3/902	15/18614	0.033354	0.335742	0.301347	BLOC1S6/	3
GO:007228	BP	GO:007228	metanephr	3/902	15/18614	0.033354	0.335742	0.301347	GREM1/ST	3
GO:009721	BP	GO:009721	lysosomal	3/902	15/18614	0.033354	0.335742	0.301347	CHMP5/CH	3
GO:009753	BP	GO:009753	mast cell m	3/902	15/18614	0.033354	0.335742	0.301347	VEGFC/VE	3
GO:012030	BP	GO:012030	regulation	3/902	15/18614	0.033354	0.335742	0.301347	BLOC1S6/	3
GO:004306	BP	GO:004306	extracellula	23/902	315/18614	0.033469	0.335742	0.301347	GREM1/EN	23
GO:005067	BP	GO:005067	regulation	29/902	419/18614	0.034692	0.335742	0.301347	CCND1/ZF	29
GO:006220	BP	GO:006220	regulation	10/902	107/18614	0.034715	0.335742	0.301347	IRF1/NR1H	10
GO:000221	BP	GO:000221	activation	18/902	233/18614	0.034873	0.335742	0.301347	IRF1/ITCH/	18
GO:003587	BP	GO:003587	nucleotide	4/902	26/18614	0.035009	0.335742	0.301347	ITCH/TNFA	4
GO:004642	BP	GO:004642	negative re	4/902	26/18614	0.035009	0.335742	0.301347	SH2B3/BCI	4
GO:005092	BP	GO:005092	regulation	4/902	26/18614	0.035009	0.335742	0.301347	VEGFC/VE	4
GO:006200	BP	GO:006200	secondary	4/902	26/18614	0.035009	0.335742	0.301347	TGFB2/SOX	4
GO:190245	BP	GO:190245	negative re	4/902	26/18614	0.035009	0.335742	0.301347	SAP30/BRN	4
GO:190390	BP	GO:190390	negative re	4/902	26/18614	0.035009	0.335742	0.301347	TRIM8/PTX	4
GO:001404	BP	GO:001404	dopamine	5/902	38/18614	0.035271	0.335742	0.301347	SYT8/HTR2	5
GO:004588	BP	GO:004588	positive reg	5/902	38/18614	0.035271	0.335742	0.301347	ARMC9/SH	5
GO:001648	BP	GO:001648	protein pro	19/902	250/18614	0.035539	0.335742	0.301347	BAG2/MAF	19
GO:004501	BP	GO:004501	glycerolipi	19/902	250/18614	0.035539	0.335742	0.301347	SOCS6/NR	19
GO:004522	BP	GO:004522	external en	23/902	317/18614	0.035584	0.335742	0.301347	GREM1/EN	23
GO:003296	BP	GO:003296	collagen bi	6/902	51/18614	0.035885	0.335742	0.301347	EMILIN1/C	6
GO:004339	BP	GO:004339	negative re	6/902	51/18614	0.035885	0.335742	0.301347	ZNF675/SU	6
GO:006068	BP	GO:006068	regulation	6/902	51/18614	0.035885	0.335742	0.301347	GREM1/SH	6
GO:006004	BP	GO:006004	cardiac mu	12/902	138/18614	0.035957	0.335742	0.301347	SUMO1/HF	12
GO:000974	BP	GO:000974	response t	18/902	234/18614	0.036144	0.335742	0.301347	ZFP36L1/M	18
GO:009009	BP	GO:009009	regulation	22/902	301/18614	0.03658	0.335742	0.301347	GREM1/EN	22
GO:000717	BP	GO:000717	transformi	17/902	218/18614	0.036638	0.335742	0.301347	EMILIN1/G	17
GO:001401	BP	GO:001401	regulation	10/902	108/18614	0.036668	0.335742	0.301347	DUSP10/VI	10
GO:000008	BP	GO:000008	G1/S transi	19/902	251/18614	0.036781	0.335742	0.301347	CCND1/RP	19
GO:000926	BP	GO:000926	cellular res	14/902	170/18614	0.037083	0.335742	0.301347	SESN3/PM	14
GO:006133	BP	GO:006133	renal tubul	8/902	79/18614	0.037179	0.335742	0.301347	GREM1/IRX	8
GO:200073	BP	GO:200073	regulation	8/902	79/18614	0.037179	0.335742	0.301347	ITCH/TGFB	8
GO:003250	BP	GO:003250	maintenan	7/902	65/18614	0.037288	0.335742	0.301347	HSPA5/KD	7
GO:001922	BP	GO:001922	cytokine-m	33/902	492/18614	0.037432	0.335742	0.301347	IRF1/ZNF6	33
GO:004352	BP	GO:004352	negative re	13/902	155/18614	0.038375	0.335742	0.301347	CCND1/NT	13
GO:002240	BP	GO:002240	molting cy	9/902	94/18614	0.038382	0.335742	0.301347	TGFB2/DLX	9
GO:002240	BP	GO:002240	hair cycle p	9/902	94/18614	0.038382	0.335742	0.301347	TGFB2/DLX	9
GO:004632	BP	GO:004632	negative re	5/902	39/18614	0.038904	0.335742	0.301347	ITCH/ZNF6	5
GO:004882	BP	GO:004882	erythrocyte	5/902	39/18614	0.038904	0.335742	0.301347	SH2B3/G6	5
GO:009007	BP	GO:009007	foam cell c	5/902	39/18614	0.038904	0.335742	0.301347	NR1H3/ST	5
GO:005160	BP	GO:005160	defense re	22/902	303/18614	0.038906	0.335742	0.301347	IRF1/ITCH/	22
GO:007233	BP	GO:007233	monocarbo	17/902	220/18614	0.039416	0.335742	0.301347	TRIB3/PTG	17
GO:000157	BP	GO:000157	ganglioside	4/902	27/18614	0.039573	0.335742	0.301347	ST6GALNA	4
GO:000209	BP	GO:000209	positive reg	4/902	27/18614	0.039573	0.335742	0.301347	GREM1/TB	4
GO:001812	BP	GO:001812	protein hyd	4/902	27/18614	0.039573	0.335742	0.301347	P4HA1/ASI	4
GO:002180	BP	GO:002180	cerebral co	4/902	27/18614	0.039573	0.335742	0.301347	SRGAP2/W	4
GO:002203	BP	GO:002203	telencepha	4/902	27/18614	0.039573	0.335742	0.301347	SRGAP2/W	4
GO:003195	BP	GO:003195	positive reg	4/902	27/18614	0.039573	0.335742	0.301347	GREM1/RA	4
GO:003414	BP	GO:003414	regulation	4/902	27/18614	0.039573	0.335742	0.301347	NR1H3/TN	4
GO:004283	BP	GO:004283	defense re	4/902	27/18614	0.039573	0.335742	0.301347	BCL3/MYD	4
GO:004849	BP	GO:004849	synaptic ve	4/902	27/18614	0.039573	0.335742	0.301347	SNAP23/CI	4
GO:009020	BP	GO:009020	positive reg	4/902	27/18614	0.039573	0.335742	0.301347	PMAIP1/BN	4
GO:001567	BP	GO:001567	oxygen tra	3/902	16/18614	0.03961	0.335742	0.301347	CYGB/HBB	3
GO:003020	BP	GO:003020	heparin me	3/902	16/18614	0.03961	0.335742	0.301347	SLC10A7/E	3
GO:004244	BP	GO:004244	progestero	3/902	16/18614	0.03961	0.335742	0.301347	STARD3/D	3
GO:004565	BP	GO:004565	positive reg	3/902	16/18614	0.03961	0.335742	0.301347	ID2/RB1/C	3
GO:004577	BP	GO:004577	negative re	3/902	16/18614	0.03961	0.335742	0.301347	TNFAIP3/C	3
GO:004838	BP	GO:004838	regulation	3/902	16/18614	0.03961	0.335742	0.301347	CYP26B1/	3
GO:006168	BP	GO:006168	chaperone	3/902	16/18614	0.03961	0.335742	0.301347	LAMP2/HS	3
GO:200034	BP	GO:200034	regulation	3/902	16/18614	0.03961	0.335742	0.301347	TNFAIP3/P	3
GO:200069	BP	GO:200069	regulation	3/902	16/18614	0.03961	0.335742	0.301347	STAT1/CTN	3
GO:007123	BP	GO:007123	cellular res	8/902	80/18614	0.039631	0.335742	0.301347	SESN3/CPE	8
GO:000690	BP	GO:000690	vesicle targ	7/902	66/18614	0.04004	0.335742	0.301347	STARD3/SI	7
GO:006067	BP	GO:006067	ureteric bu	7/902	66/18614	0.04004	0.335742	0.301347	GREM1/LA	7
GO:200123	BP	GO:200123	regulation	13/902	156/18614	0.040091	0.335742	0.301347	PMAIP1/TN	13
GO:014054	BP	GO:014054	defense re	22/902	304/18614	0.04011	0.335742	0.301347	IRF1/ITCH/	22
GO:200014	BP	GO:200014	negative re	27/902	390/18614	0.040333	0.335742	0.301347	GREM1/EN	27
GO:000275	BP	GO:000275	cytoplasmi	9/902	95/18614	0.040657	0.335742	0.301347	ITCH/TNFA	9
GO:004508	BP	GO:004508	regulation	26/902	373/18614	0.040676	0.335742	0.301347	IRF1/ITCH/	26
GO:007121	BP	GO:007121	cellular res	24/902	339/18614	0.041173	0.335742	0.301347	IRF1/ZFP36	24
GO:010400	BP	GO:010400	cellular res	24/902	339/18614	0.041173	0.335742	0.301347	IRF1/ZFP36	24
GO:004572	BP	GO:004572	positive reg	12/902	141/18614	0.041352	0.335742	0.301347	RPS27L/CY	12
GO:000222	BP	GO:000222	pattern rec	15/902	189/18614	0.041821	0.335742	0.301347	IRF1/ITCH/	15
GO:000703	BP	GO:000703	Golgi orga	13/902	157/18614	0.04186	0.335742	0.301347	LMAN1/ST	13
GO:000316	BP	GO:000316	His-Purkinj	2/902	7/18614	0.041874	0.335742	0.301347	IRX3/ID2	2
GO:000333	BP	GO:000333	regulation	2/902	7/18614	0.041874	0.335742	0.301347	STAT1/CTN	2
GO:000806	BP	GO:000806	Toll signali	2/902	7/18614	0.041874	0.335742	0.301347	MYD88/PE	2
GO:000962	BP	GO:000962	response t	2/902	7/18614	0.041874	0.335742	0.301347	PTGER2/RE	2
GO:001072	BP	GO:001072	positive reg	2/902	7/18614	0.041874	0.335742	0.301347	SNCA/SOD	2
GO:002154	BP	GO:002154	rhombome	2/902	7/18614	0.041874	0.335742	0.301347	MAFB/HOX	2
GO:002196	BP	GO:002196	anterior co	2/902	7/18614	0.041874	0.335742	0.301347	WDR47/TS	2
GO:003329	BP	GO:003329	secretion c	2/902	7/18614	0.041874	0.335742	0.301347	BLOC1S6/	2
GO:003475	BP	GO:003475	regulation	2/902	7/18614	0.041874	0.335742	0.301347	ISCU/TF	2
GO:003577	BP	GO:003577	interleukin-	2/902	7/18614	0.041874	0.335742	0.301347	PTPN2/IL4	2
GO:003579	BP	GO:003579	platelet-de	2/902	7/18614	0.041874	0.335742	0.301347	IFT20/PDG	2
GO:003814	BP	GO:003814	macropha	2/902	7/18614	0.041874	0.335742	0.301347	PTPN2/CSF	2
GO:004563	BP	GO:004563	positive reg	2/902	7/18614	0.041874	0.335742	0.301347	IL4R/RARA	2
GO:004671	BP	GO:004671	regulation	2/902	7/18614	0.041874	0.335742	0.301347	TBC1D20/	2
GO:006033	BP	GO:006033	negative re	2/902	7/18614	0.041874	0.335742	0.301347	NR1H3/PT	2
GO:006033	BP	GO:006033	negative re	2/902	7/18614	0.041874	0.335742	0.301347	NR1H3/PT	2
GO:006067	BP	GO:006067	ureteric bu	2/902	7/18614	0.041874	0.335742	0.301347	GREM1/HS	2
GO:006071	BP	GO:006071	chorio-alla	2/902	7/18614	0.041874	0.335742	0.301347	ZFP36L1/D	2
GO:006172	BP	GO:006172	lipophagy	2/902	7/18614	0.041874	0.335742	0.301347	AUP1/RAB	2
GO:007010	BP	GO:007010	interleukin-	2/902	7/18614	0.041874	0.335742	0.301347	OASL/STA	2
GO:007252	BP	GO:007252	pyridine-c	2/902	7/18614	0.041874	0.335742	0.301347	NUDT12/S	2
GO:009701	BP	GO:009701	renal prote	2/902	7/18614	0.041173	0.335742	0.301347	AMN/EDN	2
GO:011002	BP	GO:011002	cardiac mu	2/902	7/18614	0.041173	0.335742	0.301347	GATA6/MA	2
GO:011002	BP	GO:011002	regulation	2/902	7/18614	0.041874	0.335742	0.301347	GATA6/MA	2
GO:190109	BP	GO:190109	positive reg	2/902	7/18614	0.041874	0.335742	0.301347	TOM1/SMC	2
GO:190360	BP	GO:190360	protein loc	2/902	7/18614	0.041874	0.335742	0.301347	TIA1/SSB	2
GO:190568	BP	GO:190568	regulation	2/902	7/18614	0.041874	0.335742	0.301347	CASP7/RAI	2
GO:199079	BP	GO:199079	dorsal root	2/902	7/18614	0.041874	0.335742	0.301347	UNC5C/CT	2
GO:200048	BP	GO:200048	regulation	2/902	7/18614	0.041874	0.335742	0.301347	CYGB/GCL	2
GO:200064	BP	GO:200064	positive reg	2/902	7/18614	0.041874	0.335742	0.301347	ITCH/LAPT	2
GO:003432	BP	GO:003432	cell junctio	30/902	444/18614	0.042045	0.335742	0.301347	GREM1/M	30
GO:003101	BP	GO:003101	pancreas c	8/902	81/18614	0.042188	0.335742	0.301347	GATA6/SO	8
GO:000622	BP	GO:000622	pyrimidine	6/902	53/18614	0.042217	0.335742	0.301347	NTHL1/CM	6
GO:004001	BP	GO:004001	negative re	29/902	427/18614	0.042614	0.335742	0.301347	GREM1/EN	29
GO:003250	BP	GO:003250	cytokinetic	5/902	40/18614	0.042756	0.335742	0.301347	SNX18/CH	5
GO:003588	BP	GO:003588	vascular as	5/902	40/18614	0.042756	0.335742	0.301347	GATA6/EN	5
GO:004848	BP	GO:004848	synaptic ve	5/902	40/18614	0.042756	0.335742	0.301347	BLOC1S6/	5
GO:006038	BP	GO:006038	pathway-r	7/902	67/18614	0.042922	0.335742	0.301347	GREM1/EN	7
GO:007217	BP	GO:007217	mesoneph	7/902	67/18614	0.042922	0.335742	0.301347	GREM1/LA	7
GO:000664	BP	GO:000664	phospholip	26/902	375/18614	0.04293	0.335742	0.301347	GATA6/SO	26
GO:000716	BP	GO:000716	cell-matrix	18/902	239/18614	0.043014	0.335742	0.301347	ITGBL1/GR	18
GO:003367	BP	GO:003367	negative re	18/902	239/18614	0.043014	0.335742	0.301347	TRIB3/ZNF	18
GO:007208	BP	GO:007208	nephron tu	9/902	96/18614	0.04302	0.335742	0.301347	GREM1/IRX	9
GO:190351	BP	GO:190351	mucopolys	9/902	96/18614	0.04302	0.335742	0.301347	TNFAIP6/D	9
GO:001935	BP	GO:001935	nicotinami	4/902	28/18614	0.04446	0.335742	0.301347	NADK2/NA	4
GO:001936	BP	GO:001936	pyridine nu	4/902	28/18614	0.04446	0.335742	0.301347	NADK2/NA	4
GO:003158	BP	GO:003158	cell-substr	25/902	359/18614	0.04448	0.335742	0.301347	ITGBL1/GR	25
GO:004214	BP	GO:004214	retrograde	9/902	97/18614	0.045475	0.335742	0.301347	GOLT1B/TI	9
GO:001624	BP	GO:001624	regulation	13/902	159/18614	0.045562	0.335742	0.301347	SESN3/SN	13
GO:003953	BP	GO:003953	regulation	6/902	54/18614	0.045629	0.335742	0.301347	TNFAIP3/C	6
GO:004355	BP	GO:004355	regulation	6/902	54/18614	0.045629	0.335742	0.301347	NRBF2/RB1	6
GO:004668	BP	GO:004668	response t	6/902	54/18614	0.045629	0.335742	0.301347	SUMO1/CH	6
GO:007132	BP	GO:007132	cellular res	6/902	54/18614	0.045629	0.335742	0.301347	ZFP36L1/A	6
GO:001590	BP	GO:001590	long-chain	7/902	68/18614	0.045935	0.335742	0.301347	MFSD2A/S	7
GO:004567	BP	GO:004567	regulation	7/902	68/18614	0.045935	0.335742	0.301347	TNFAIP6/M	7
GO:007093	BP	GO:007093	protein K48	7/902	68/18614	0.045935	0.335742	0.301347	ITCH/SYVN	7
GO:200014	BP	GO:200014	positive reg	7/902	68/18614	0.045935	0.335742	0.301347	MED31/ME	7
GO:000157	BP	GO:000157	ganglioside	3/902	17/18614	0.046414	0.335742	0.301347	ST6GALNA	3
GO:000207	BP	GO:000207	osteoblast	3/902	17/18614	0.046414	0.335742	0.301347	PTHLH/RU	3
GO:003164	BP	GO:003164	heat gener	3/902	17/18614	0.046414	0.335742	0.301347	ARRDC3/P	3
GO:003251	BP	GO:003251	endosome	3/902	17/18614	0.046414	0.335742	0.301347	CHMP5/UE	3
GO:003318	BP	GO:003318	response t	3/902	17/18614	0.046414	0.335742	0.301347	CYP26B1/T	3
GO:003413	BP	GO:003413	toll-like rec	3/902	17/18614	0.046414	0.335742	0.301347	TNFAIP3/T	3
GO:003616	BP	GO:003616	phenotypic	3/902	17/18614	0.046414	0.335742	0.301347	SOD2/FGF	3
GO:004324	BP	GO:004324	erythrocyte	3/902	17/18614	0.046414	0.335742	0.301347	G6PD/ZBT	3
GO:004453	BP	GO:004453	long-chain	3/902	17/18614	0.046414	0.335742	0.301347	SLC27A2/F	3
GO:006115	BP	GO:006115	3'-UTR-me	3/902	17/18614	0.046414	0.335742	0.301347	ZFP36L1/C	3
GO:006154	BP	GO:006154	ganglion d	3/902	17/18614	0.046414	0.335742	0.301347	CYP1B1/UN	3
GO:000828	BP	GO:000828	insulin rece	11/902	128/18614	0.046588	0.335742	0.301347	SESN3/PDI	11
GO:190227	BP	GO:190227	regulation	5/902	41/18614	0.04683	0.335742	0.301347	TLK1/SAM	5
GO:005067	BP	GO:005067	positive reg	17/902	225/18614	0.047025	0.335742	0.301347	CCND1/FZ	17
GO:001921	BP	GO:001921	regulation	24/902	344/18614	0.04735	0.335742	0.301347	TRIB3/LAC	24
GO:004434	BP	GO:004434	cellular res	10/902	113/18614	0.047577	0.335742	0.301347	ZFP36L1/G	10
GO:005185	BP	GO:005185	modulation	8/902	83/18614	0.047625	0.335742	0.301347	PPIB/ZDHH	8
GO:007233	BP	GO:007233	intrinsic ap	8/902	83/18614	0.047625	0.335742	0.301347	PHLDA3/RI	8
GO:000166	BP	GO:000166	response t	21/902	293/18614	0.048036	0.335742	0.301347	GATA6/ZFI	21
GO:000169	BP	GO:000169	histamine	1/902	1/18614	0.048458	0.335742	0.301347	HNMT	1
GO:000171	BP	GO:000171	ectoderma	1/902	1/18614	0.048458	0.335742	0.301347	FZD7	1
GO:000219	BP	GO:000219	IRES-depe	1/902	1/18614	0.048458	0.335742	0.301347	RBM4	1
GO:000229	BP	GO:000229	gamma-d	1/902	1/18614	0.048458	0.335742	0.301347	LILRB1	1
GO:000238	BP	GO:000238	immune re	1/902	1/18614	0.048458	0.335742	0.301347	TREX1	1
GO:000306	BP	GO:000306	positive reg	1/902	1/18614	0.048458	0.335742	0.301347	TPM1	1
GO:000306	BP	GO:000306	regulation	1/902	1/18614	0.048458	0.335742	0.301347	SOD2	1
GO:000306	BP	GO:000306	acetylcholi	1/902	1/18614	0.048458	0.335742	0.301347	SOD2	1
GO:000307	BP	GO:000307	regulation	1/902	1/18614	0.048458	0.335742	0.301347	SOD2	1
GO:000334	BP	GO:000334	proepicard	1/902	1/18614	0.048458	0.335742	0.301347	FLRT3	1
GO:000336	BP	GO:000336	lamellipodi	1/902	1/18614	0.048458	0.335742	0.301347	SRGAP2	1
GO:000604	BP	GO:000604	UDP-N-ac	1/902	1/18614	0.048458	0.335742	0.301347	MGAT1	1
GO:000617	BP	GO:000617	dATP biosy	1/902	1/18614	0.048458	0.335742	0.301347	ADK	1
GO:000666	BP	GO:000666	3-keto-spl	1/902	1/18614	0.048458	0.335742	0.301347	KDSR	1
GO:000740	BP	GO:000740	glial cell fa	1/902	1/18614	0.048458	0.335742	0.301347	CTNNB1	1
GO:000968	BP	GO:000968	induced sy	1/902	1/18614	0.048458	0.335742	0.301347	MYD88	1
GO:000995	BP	GO:000995	radial patte	1/902	1/18614	0.048458	0.335742	0.301347	NR2F2	1
GO:001250	BP	GO:001250	induction c	1/902	1/18614	0.048458	0.335742	0.301347	LAPTM5	1
GO:001587	BP	GO:001587	acetyl-CoA	1/902	1/18614	0.048458	0.335742	0.301347	SLC33A1	1
GO:001611	BP	GO:001611	tetraterper	1/902	1/18614	0.048458	0.335742	0.301347	BCO2	1
GO:001611	BP	GO:001611	carotenoid	1/902	1/18614	0.048458	0.335742	0.301347	BCO2	1
GO:001612	BP	GO:001612	xanthophyl	1/902	1/18614	0.048458	0.335742	0.301347	BCO2	1
GO:001801	BP	GO:001801	N-termina	1/902	1/18614	0.048458	0.335742	0.301347	NTMT1	1
GO:001801	BP	GO:001801	N-termina	1/902	1/18614	0.048458	0.335742	0.301347	NTMT1	1
GO:001801	BP	GO:001801	N-termina	1/902	1/18614	0.048458	0.335742	0.301347	NTMT1	1
GO:001801	BP	GO:001801	N-termina	1/902	1/18614	0.048458	0.335742	0.301347	NTMT1	1
GO:001819	BP	GO:001819	peptidyl-a	1/902	1/18614	0.048458	0.335742	0.301347	NTMT1	1
GO:001829	BP	GO:001829	protein-FA	1/902	1/18614	0.048458	0.335742	0.301347	SDHAF2	1
GO:001936	BP	GO:001936	pyridine nu	1/902	1/18614	0.048458	0.335742	0.301347	NAPRT	1
GO:001991	BP	GO:001991	peptidyl-a	1/902	1/18614	0.048458	0.335742	0.301347	NDUFAF7	1
GO:002150	BP	GO:002150	neural fold	1/902	1/18614	0.048458	0.335742	0.301347	LUZP1	1
GO:002151	BP	GO:002151	spinal cord	1/902	1/18614	0.048458	0.335742	0.301347	GDF11	1

GO:190471	BP	GO:190471	regulation	1/1035	1/18614	0.055603	0.45089	0.432223	DKK1	1
GO:190472	BP	GO:190472	negative re	1/1035	1/18614	0.055603	0.45089	0.432223	DKK1	1
GO:190482	BP	GO:190482	positive re	1/1035	1/18614	0.055603	0.45089	0.432223	SP1	1
GO:190500	BP	GO:190500	regulation	1/1035	1/18614	0.055603	0.45089	0.432223	AGT	1
GO:190501	BP	GO:190501	positive re	1/1035	1/18614	0.055603	0.45089	0.432223	AGT	1
GO:190506	BP	GO:190506	negative re	1/1035	1/18614	0.055603	0.45089	0.432223	DKK1	1
GO:190557	BP	GO:190557	ganglioside	1/1035	1/18614	0.055603	0.45089	0.432223	PSAP	1
GO:190558	BP	GO:190558	positive re	1/1035	1/18614	0.055603	0.45089	0.432223	AGT	1
GO:190559	BP	GO:190559	positive re	1/1035	1/18614	0.055603	0.45089	0.432223	ANXA2	1
GO:190560	BP	GO:190560	negative re	1/1035	1/18614	0.055603	0.45089	0.432223	DKK1	1
GO:190586	BP	GO:190586	negative re	1/1035	1/18614	0.055603	0.45089	0.432223	METTL16	1
GO:199009	BP	GO:199009	calcium-de	1/1035	1/18614	0.055603	0.45089	0.432223	CAPN3	1
GO:199054	BP	GO:199054	mitochond	1/1035	1/18614	0.055603	0.45089	0.432223	SLC25A3	1
GO:199067	BP	GO:199067	histone H4	1/1035	1/18614	0.055603	0.45089	0.432223	HDAC9	1
GO:200018	BP	GO:200018	positive re	1/1035	1/18614	0.055603	0.45089	0.432223	ADM	1
GO:200053	BP	GO:200053	negative re	1/1035	1/18614	0.055603	0.45089	0.432223	GAS6	1
GO:200053	BP	GO:200053	regulation	1/1035	1/18614	0.055603	0.45089	0.432223	MUSK	1
GO:200054	BP	GO:200054	positive re	1/1035	1/18614	0.055603	0.45089	0.432223	MUSK	1
GO:200061	BP	GO:200061	regulation	1/1035	1/18614	0.055603	0.45089	0.432223	PAX8	1
GO:200068	BP	GO:200068	actomyosin	1/1035	1/18614	0.055603	0.45089	0.432223	PLEC	1
GO:200079	BP	GO:200079	negative re	1/1035	1/18614	0.055603	0.45089	0.432223	NFIB	1
GO:200085	BP	GO:200085	negative re	1/1035	1/18614	0.055603	0.45089	0.432223	TSPO	1
GO:200116	BP	GO:200116	negative re	1/1035	1/18614	0.055603	0.45089	0.432223	MLLT6	1
GO:000248	BP	GO:000248	antigen pro	3/1035	16/18614	0.055823	0.45089	0.432223	HLA-B/HL	3
GO:000673	BP	GO:000673	NADH rege	3/1035	16/18614	0.055823	0.45089	0.432223	ENO3/GCK	3
GO:003806	BP	GO:003806	collagen-a	3/1035	16/18614	0.055823	0.45089	0.432223	JAK2/DDR1	3
GO:004350	BP	GO:004350	negative re	3/1035	16/18614	0.055823	0.45089	0.432223	GSTP1/DU	3
GO:004398	BP	GO:004398	histone H4	3/1035	16/18614	0.055823	0.45089	0.432223	KANSL3/O	3
GO:004398	BP	GO:004398	histone H4	3/1035	16/18614	0.055823	0.45089	0.432223	KANSL3/O	3
GO:004683	BP	GO:004683	lipid phosp	3/1035	16/18614	0.055823	0.45089	0.432223	DGKG/DGK	3
GO:004748	BP	GO:004748	regulation	3/1035	16/18614	0.055823	0.45089	0.432223	MLC1/TIFA	3
GO:005101	BP	GO:005101	actin filam	3/1035	16/18614	0.055823	0.45089	0.432223	AVIL/CFL2	3
GO:006162	BP	GO:006162	canonical	3/1035	16/18614	0.055823	0.45089	0.432223	ENO3/GCK	3
GO:006171	BP	GO:006171	glucose ca	3/1035	16/18614	0.055823	0.45089	0.432223	ENO3/GCK	3
GO:007102	BP	GO:007102	RNA survei	3/1035	16/18614	0.055823	0.45089	0.432223	EXOSC10/	3
GO:200063	BP	GO:200063	positive re	3/1035	16/18614	0.055823	0.45089	0.432223	PUM1/STA	3
GO:200084	BP	GO:200084	regulation	3/1035	16/18614	0.055823	0.45089	0.432223	POMC/AGT	3
GO:005189	BP	GO:005189	membrane	8/1035	75/18614	0.056135	0.452889	0.43414	GPD1L/AN	8
GO:001839	BP	GO:001839	peptidyl-ly	15/1035	172/18614	0.056428	0.454729	0.435904	MUC1/RU	15
GO:002151	BP	GO:002151	spinal cord	10/1035	102/18614	0.057208	0.460487	0.441423	DAAM2/TC	10
GO:000989	BP	GO:000989	negative re	26/1035	337/18614	0.057528	0.462302	0.443163	RBM10/MT	26
GO:000600	BP	GO:000600	glucose me	16/1035	187/18614	0.057565	0.462302	0.443163	LCMT1/DC	16
GO:009010	BP	GO:009010	cochlea de	6/1035	50/18614	0.057693	0.462801	0.443641	SOX9/PAF	6
GO:003288	BP	GO:003288	regulation	5/1035	38/18614	0.05779	0.463042	0.443872	HAS2/SOR	5
GO:000918	BP	GO:000918	ribonucleo	11/1035	116/18614	0.058033	0.463932	0.444725	JMJD8/OG	11
GO:007200	BP	GO:007200	nephron ep	11/1035	116/18614	0.058033	0.463932	0.444725	MAGI2/FO	11
GO:007233	BP	GO:007233	signal trans	15/1035	173/18614	0.058764	0.469244	0.449818	MUC1/RRS	15
GO:000611	BP	GO:000611	energy res	9/1035	89/18614	0.058853	0.469422	0.449988	UGP2/POM	9
GO:000915	BP	GO:000915	purine ribo	18/1035	217/18614	0.058927	0.469479	0.450044	PPT1/NME	18
GO:003011	BP	GO:003011	regulation	26/1035	338/18614	0.059181	0.470763	0.451274	APCDD1/K	26
GO:000616	BP	GO:000616	purine nucl	21/1035	262/18614	0.059347	0.470763	0.451274	PPT1/NME	21
GO:000663	BP	GO:000663	fatty acid b	8/1035	76/18614	0.059829	0.470763	0.451274	ACADL/EC	8
GO:000720	BP	GO:000720	protein kin	4/1035	27/18614	0.060229	0.470763	0.451274	DGKG/DGK	4
GO:000906	BP	GO:000906	glutamine	4/1035	27/18614	0.060229	0.470763	0.451274	ASRGL1/D	4
GO:000965	BP	GO:000965	response t	4/1035	27/18614	0.060229	0.470763	0.451274	PAPPA2/O	4
GO:001081	BP	GO:001081	T cell chem	4/1035	27/18614	0.060229	0.470763	0.451274	CXCL16/PL	4
GO:003601	BP	GO:003601	protein loc	4/1035	27/18614	0.060229	0.470763	0.451274	MSN/PACS	4
GO:004213	BP	GO:004213	neurotrans	4/1035	27/18614	0.060229	0.470763	0.451274	SLC44A4/	4
GO:004594	BP	GO:004594	positive re	4/1035	27/18614	0.060229	0.470763	0.451274	NR1D1/FG	4
GO:004603	BP	GO:004603	GMP meta	4/1035	27/18614	0.060229	0.470763	0.451274	PAICS/PFA	4
GO:004675	BP	GO:004675	viral buddi	4/1035	27/18614	0.060229	0.470763	0.451274	VPS37B/V	4
GO:004694	BP	GO:004694	fatty-acyl-	4/1035	27/18614	0.060229	0.470763	0.451274	PPT1/PPT2	4
GO:004820	BP	GO:004820	vesicle targ	4/1035	27/18614	0.060229	0.470763	0.451274	TRAPPC2L	4
GO:004820	BP	GO:004820	COPII vesic	4/1035	27/18614	0.060229	0.470763	0.451274	TRAPPC2L	4
GO:007154	BP	GO:007154	cellular res	4/1035	27/18614	0.060229	0.470763	0.451274	SMYD3/JA	4
GO:190435	BP	GO:190435	negative re	4/1035	27/18614	0.060229	0.470763	0.451274	PINX1/EXC	4
GO:190307	BP	GO:190307	regulation	10/1035	103/18614	0.060358	0.471248	0.451738	VAMP8/AR	10
GO:000724	BP	GO:000724	I-kappaB k	23/1035	293/18614	0.060715	0.473507	0.453905	PRKD1/CA	23
GO:007110	BP	GO:007110	DNA confo	9/1035	90/18614	0.062326	0.48331	0.463302	GINS2/RU	9
GO:003287	BP	GO:003287	negative re	6/1035	51/18614	0.062454	0.48331	0.463302	PPIA/EZR/	6
GO:004576	BP	GO:004576	regulation	6/1035	51/18614	0.062454	0.48331	0.463302	ADRB2/GN	6
GO:005088	BP	GO:005088	neuromusc	6/1035	51/18614	0.062454	0.48331	0.463302	ALDH1A3/	6
GO:005148	BP	GO:005148	regulation	6/1035	51/18614	0.062454	0.48331	0.463302	DAAM2/AR	6
GO:006068	BP	GO:006068	regulation	6/1035	51/18614	0.062454	0.48331	0.463302	AR/PHB2/	6
GO:007030	BP	GO:007030	negative re	6/1035	51/18614	0.062454	0.48331	0.463302	PPIA/EZR/	6
GO:005071	BP	GO:005071	positive re	13/1035	146/18614	0.063269	0.485804	0.465692	JAK2/SYTL	13
GO:004240	BP	GO:004240	cellular bio	5/1035	39/18614	0.063389	0.485804	0.465692	SMOX/MO	5
GO:005265	BP	GO:005265	cyclic purin	5/1035	39/18614	0.063389	0.485804	0.465692	ADCY3/AD	5
GO:001635	BP	GO:001635	dendrite de	19/1035	234/18614	0.06342	0.485804	0.465692	COBL/TNIK	19
GO:000662	BP	GO:000662	protein targ	10/1035	104/18614	0.063618	0.485804	0.465692	SAMM50/	10
GO:190113	BP	GO:190113	carbohydra	15/1035	175/18614	0.063632	0.485804	0.465692	HYAL1/GP	15
GO:003250	BP	GO:003250	DNA duple	8/1035	77/18614	0.063672	0.485804	0.465692	GINS2/RU	8
GO:003254	BP	GO:003254	mitochond	12/1035	132/18614	0.063884	0.485804	0.465692	MRPL37/M	12
GO:005105	BP	GO:005105	negative re	7/1035	64/18614	0.063963	0.485804	0.465692	ACADL/MA	7
GO:007005	BP	GO:007005	neuron cell	7/1035	64/18614	0.063963	0.485804	0.465692	DCTN1/AT	7
GO:000193	BP	GO:000193	negative re	26/1035	341/18614	0.064342	0.485804	0.465692	CTDSP2/G	26
GO:001839	BP	GO:001839	internal pe	14/1035	161/18614	0.064952	0.485804	0.465692	MUC1/RU	14
GO:190285	BP	GO:190285	microtubul	14/1035	161/18614	0.064952	0.485804	0.465692	NSFL1C/D	14
GO:000209	BP	GO:000209	negative re	3/1035	17/18614	0.065077	0.485804	0.465692	MTMR2/A	3
GO:000247	BP	GO:000247	antigen pro	3/1035	17/18614	0.065077	0.485804	0.465692	HLA-B/HL	3
GO:000248	BP	GO:000248	antigen pro	3/1035	17/18614	0.065077	0.485804	0.465692	HLA-B/HL	3
GO:000608	BP	GO:000608	acetyl-CoA	3/1035	17/18614	0.065077	0.485804	0.465692	PDHB/ACL	3
GO:000610	BP	GO:000610	2-oxogluta	3/1035	17/18614	0.065077	0.485804	0.465692	OGDH/D2	3
GO:000647	BP	GO:000647	N-termina	3/1035	17/18614	0.065077	0.485804	0.465692	NAA30/NA	3
GO:000915	BP	GO:000915	purine deo	3/1035	17/18614	0.065077	0.485804	0.465692	NUDT18/G	3
GO:003413	BP	GO:003413	toll-like rec	3/1035	17/18614	0.065077	0.485804	0.465692	COLEC12/	3
GO:004683	BP	GO:004683	phosphory	3/1035	17/18614	0.065077	0.485804	0.465692	INPP1/MT	3
GO:004685	BP	GO:004685	inositol ph	3/1035	17/18614	0.065077	0.485804	0.465692	INPP1/MT	3
GO:005507	BP	GO:005507	copper ion	3/1035	17/18614	0.065077	0.485804	0.465692	COMMD1/	3
GO:006014	BP	GO:006014	positive re	3/1035	17/18614	0.065077	0.485804	0.465692	PUM1/STA	3
GO:006038	BP	GO:006038	regulation	3/1035	17/18614	0.065077	0.485804	0.465692	RUVBL2/IN	3
GO:006045	BP	GO:006045	negative re	3/1035	17/18614	0.065077	0.485804	0.465692	OXT/TIFAB	3
GO:190037	BP	GO:190037	positive re	3/1035	17/18614	0.065077	0.485804	0.465692	PUM1/STA	3
GO:190354	BP	GO:190354	regulation	3/1035	17/18614	0.065077	0.485804	0.465692	SDC4/PDC	3
GO:200126	BP	GO:200126	regulation	3/1035	17/18614	0.065077	0.485804	0.465692	JAK2/NLE1	3
GO:003304	BP	GO:003304	regulation	20/1035	250/18614	0.065508	0.485804	0.465692	RMI2/MAP	20
GO:006034	BP	GO:006034	bone deve	19/1035	235/18614	0.065581	0.485804	0.465692	PAPPA2/E	19
GO:003227	BP	GO:003227	regulation	16/1035	191/18614	0.066994	0.485804	0.465692	KANK1/DA	16
GO:000714	BP	GO:000714	male meio	4/1035	28/18614	0.067304	0.485804	0.465692	FOXJ3/FO	4
GO:001089	BP	GO:001089	negative re	4/1035	28/18614	0.067304	0.485804	0.465692	MALRD1/E	4
GO:001569	BP	GO:001569	quaternary	4/1035	28/18614	0.067304	0.485804	0.465692	SLC44A4/S	4
GO:003295	BP	GO:003295	inositol ph	4/1035	28/18614	0.067304	0.485804	0.465692	ITPKC/PLC	4
GO:003406	BP	GO:003406	stress gran	4/1035	28/18614	0.067304	0.485804	0.465692	EIF2S1/YT	4
GO:190367	BP	GO:190367	positive re	4/1035	28/18614	0.067304	0.485804	0.465692	JMJD8/FG	4
GO:000619	BP	GO:000619	purine nucl	6/1035	52/18614	0.067439	0.485804	0.465692	ACOT7/NU	6
GO:003531	BP	GO:003531	hair cell dif	6/1035	52/18614	0.067439	0.485804	0.465692	SLC44A4/	6
GO:004519	BP	GO:004519	establishm	6/1035	52/18614	0.067439	0.485804	0.465692	MSN/DLG	6
GO:004801	BP	GO:004801	ephrin rece	6/1035	52/18614	0.067439	0.485804	0.465692	TIAM1/EF	6
GO:005149	BP	GO:005149	positive re	6/1035	52/18614	0.067439	0.485804	0.465692	ARHGEF5/	6
GO:003272	BP	GO:003272	negative re	8/1035	78/18614	0.067666	0.485804	0.465692	AXL/POM	8
GO:004358	BP	GO:004358	ear develo	18/1035	221/18614	0.067757	0.485804	0.465692	SLC44A4/	18
GO:005133	BP	GO:005133	regulation	7/1035	65/18614	0.068414	0.485804	0.465692	ADRB2/GN	7
GO:000691	BP	GO:000691	nucleocyto	25/1035	328/18614	0.068822	0.485804	0.465692	PRKD1/JA	25
GO:005116	BP	GO:005116	nuclear tra	25/1035	328/18614	0.068822	0.485804	0.465692	PRKD1/JA	25
GO:000005	BP	GO:000005	citrulline m	2/1035	8/18614	0.069171	0.485804	0.465692	DDAH1/D	2
GO:000005	BP	GO:000005	ribosomal	2/1035	8/18614	0.069171	0.485804	0.465692	RRS1/NM	2
GO:000272	BP	GO:000272	positive re	2/1035	8/18614	0.069171	0.485804	0.465692	HLA-F/HL	2
GO:000309	BP	GO:000309	renal water	2/1035	8/18614	0.069171	0.485804	0.465692	HAS2/MLL	2
GO:000319	BP	GO:000319	atrioventric	2/1035	8/18614	0.069171	0.485804	0.465692	OLFM1/ZF	2
GO:000655	BP	GO:000655	leucine me	2/1035	8/18614	0.069171	0.485804	0.465692	BCAT2/MC	2
GO:000655	BP	GO:000655	L-phenylal	2/1035	8/18614	0.069171	0.485804	0.465692	GSTZ1/PA	2
GO:000655	BP	GO:000655	L-phenylal	2/1035	8/18614	0.069171	0.485804	0.465692	GSTZ1/PA	2
GO:000944	BP	GO:000944	gamma-ar	2/1035	8/18614	0.069171	0.485804	0.465692	ABAT/PHG	2
GO:001021	BP	GO:001021	maintenan	2/1035	8/18614	0.069171	0.485804	0.465692	HELLS/UH	2
GO:001405	BP	GO:001405	regulation	2/1035	8/18614	0.069171	0.485804	0.465692	ABAT/GAB	2
GO:002181	BP	GO:002181	cell motility	2/1035	8/18614	0.069171	0.485804	0.465692	SUN2/SYN	2
GO:003282	BP	GO:003282	positive re	2/1035	8/18614	0.069171	0.485804	0.465692	AXL/GAS6	2
GO:003545	BP	GO:003545	cellular res	2/1035	8/18614	0.069171	0.485804	0.465692	AXL/GAS6	2
GO:003575	BP	GO:003575	B cell chem	2/1035	8/18614	0.069171	0.485804	0.465692	GAS6/PIK3	2
GO:003808	BP	GO:003808	positive re	2/1035	8/18614	0.069171	0.485804	0.465692	PRKD1/PIK	2
GO:004275	BP	GO:004275	long-chain	2/1035	8/18614	0.069171	0.485804	0.465692	ACADL/AD	2
GO:004363	BP	GO:004363	polyadenyl	2/1035	8/18614	0.069171	0.485804	0.465692	EXOSC10/	2
GO:004420	BP	GO:004420	'de novo' A	2/1035	8/18614	0.069171	0.485804	0.465692	PAICS/PFA	2
GO:004595	BP	GO:004595	negative re	2/1035	8/18614	0.069171	0.485804	0.465692	C4BPA/CD	2
GO:004687	BP	GO:004687	regulation	2/1035	8/18614	0.069171	0.485804	0.465692	TIFAB/DCA	2
GO:005196	BP	GO:005196	negative re	2/1035	8/18614	0.069171	0.485804	0.465692	ROBO2/DK	2
GO:006039	BP	GO:006039	growth hor	2/1035	8/18614	0.069171	0.485804	0.465692	JAK2/STAT	2
GO:006050	BP	GO:006050	type I pneu	2/1035	8/18614	0.069171	0.485804	0.465692	SOX9/NFIB	2
GO:006067	BP	GO:006067	ureteric bu	2/1035	8/18614	0.069171	0.485804	0.465692	FGF1/KIF2	2
GO:006188	BP	GO:006188	regulation	2/1035	8/18614	0.069171	0.485804	0.465692	NR1D1/LD	2
GO:007047	BP	GO:007047	positive re	2/1035	8/18614	0.069171	0.485804	0.465692	OXT/ABAT	2
GO:007203	BP	GO:007203	renal vesicl	2/1035	8/18614	0.069171	0.485804	0.465692	SOX9/KIF2	2
GO:007228	BP	GO:007228	metanephr	2/1035	8/18614	0.069171	0.485804	0.465692	PAX8/SOX	2
GO:009011	BP	GO:009011	receptor-n	2/1035	8/18614	0.069171	0.485804	0.465692	ANXA2/LD	2
GO:009716	BP	GO:009716	mesenchyr	2/1035	8/18614	0.069171	0.485804	0.465692	LTBP3/CIT	2
GO:009890	BP	GO:009890	regulation	2/1035	8/18614	0.069171	0.485804	0.465692	FGF13/RAN	2
GO:190222	BP	GO:190222	erythrose	2/1035	8/18614	0.069171	0.485804	0.465692	GSTZ1/PA	2
GO:190222	BP	GO:190222	erythrose	2/1035	8/18614	0.069171	0.485804	0.465692	GSTZ1/PA	2
GO:190375	BP	GO:190375	negative re	2/1035	8/18614	0.069171	0.485804	0.465692	EZR/PTPN2	2
GO:190397	BP	GO:190397	negative re	2/1035	8/18614	0.069171	0.485804	0.465692	NR1D1/LD	2
GO:190439	BP	GO:190439	regulation	2/1035	8/18614	0.069171	0.485804	0.465692	DCTN1/MU	2
GO:190449	BP	GO:190449	protein loc	2/1035	8/18614	0.069171	0.485804	0.465692	TCTN1/SPA	2
GO:190583	BP	GO:190583	positive re	2/1035	8/18614	0.069171	0.485804	0.465692	NUMA1/G	2
GO:200060	BP	GO:200060	positive re	2/1035	8/18614	0.069171	0.485804	0.465692	WASF3/CY	2
GO:000247	BP	GO:000247	antigen pro	5/1035	40/18614	0.069282	0.485804	0.465692	TRAF6/DN	5
GO:000918	BP	GO:000918	cyclic nucle	5/1035	40/18614	0.069282	0.485804	0.465692	ADCY3/AD	5
GO:000930	BP	GO:000930	amine bios	5/1035	40/18614	0.069282	0.485804	0.465692	SMOX/MO	5
GO:003533	BP	GO:003533	fatty-acyl-	5/1035	40/18614	0.069282	0.485804	0.465692	PPT1/ACO	5
GO:004848	BP	GO:004848	synaptic ve	5/1035	40/18614	0.069282	0.485804	0.465692	LIN7A/DN	5
GO:004440	BP	GO:004440	biological	24/1035	313/18614	0.069636	0.48731	0.467136	VAMP8/LA	24
GO:006007	BP	GO:006007	canonical	24/1035	313/18614	0.069636	0.48731	0.467136	KANK1/DA	24
GO:190211	BP	GO:190211	regulation	17/1035	207/18614	0.06993	0.488881	0.468642	ARHGEF5/	17
GO:000647	BP	GO:000647	internal pro	14/1035	163/18614	0.070407	0.491695	0.471339	MUC1/RU	14
GO:190179	BP	GO:190179	regulation	10/1035	106/18614	0.070472	0.491695	0.471339	MUC1/RRS	10
GO:190290	BP	GO:190290	regulation	28/1035	376/18614	0.071406	0.497717	0.477112	ARHGEF5/	28
GO:000640	BP	GO:000640	mRNA cata	21/1035	268/18614	0.071612	0.498658	0.478014	RBM10/RB	21
GO:004238	CC	GO:004238	sarcolemm	21/1071	136/19518	1.63E-05	0.013892	0.01248	COL6A2/S	21
GO:003125	CC	GO:003125	cell leading	44/1071	422/19518	3.42E-05	0.014583	0.013101	TIAM2/CO	44
GO:000593	CC	GO:000593	cell cortex	34/1071	302/19518	5.80E-05	0.015063	0.013532	COBL/PRK	34
GO:001562	CC	GO:001562	actin cytos	48/1071	489/19518	7.06E-05	0.015063	0.013532	COBL/ARH	48
GO:199035	CC	GO:199035	terminal w	3/1071	3/19518	0.000165	0.023843	0.02142	COBL/KRT	3
GO:009743	CC	GO:009743	mitotic spi	9/1071	38/19518	0.000168	0.023843	0.02142	CNTRL/RA	9
GO:000592	CC	GO:000592	focal adhe	40/1071	422/19518	0.000556	0.067799	0.060909	ZNF185/P	40
GO:009885	CC	GO:009885	actin-base	25/1071	225/19518	0.000642	0.068414	0.061461	TIAM2/DC	25
GO:003005	CC	GO:003005	cell-substr	40/1071	432/19518	0.000877	0.082682	0.07428	ZNF185/P	40
GO:000172	CC	GO:000172	ruffle	21/1071	181/19518	0.000969	0.082682	0.07428	COBL/PAL	21
GO:009886	CC	GO:009886	cluster of a	19/1071	162/19518	0.001456	0.103231	0.092741	RSPH9/DC	19
GO:003086	CC	GO:003086	cortical cyt	14/1071	104/19518	0.001621	0.103231	0.092741	COBL/SHR	14
GO:000577	CC	GO:000577	vacuolar lu	20/1071	176/19518	0.001631	0.103231	0.092741	HYAL1/PPT	20
GO:003125	CC	GO:003125	cell project	33/1071	349/19518	0.001694	0.103231	0.092741	SGCE/KAN	33
GO:003086	CC	GO:003086	cortical act	11/1071	73/19518	0.002011	0.11157	0.100232	COBL/SHR	11
GO:003125	CC	GO:003125	leading ed	20/1071	180/19518	0.002134	0.11157	0.100232	SGCE/KAN	20
GO:004303	CC	GO:004303	costamere	5/1071	18/19518	0.002317	0.11157	0.100232	ANK3/SDC	5
GO:004264	CC	GO:004264	actomyosin	11/1071	76/19518	0.002789	0.11157	0.100232	PALLD/LIM	11
GO:003439	CC	GO:003439	nuclear per	17/1071	147/19518	0.002928	0.11157	0.100232	RUVBL2/A	17
GO:000558	CC	GO:000558	collagen ty	2/1071	2/19518	0.003008	0.11157	0.100232	COL6A3/C	2
GO:009757	CC	GO:009757	lateral cell	2/1071	2/19518	0.003008	0.11157	0.100232	NUMA1/G	2
GO:009864	CC	GO:009864	collagen b	2/1071	2/19518	0.003008	0.11157	0.100232	COL6A3/C	2
GO:199073	CC	GO:199073	VCP-NSFL	2/1071	2/19518	0.003008	0.11157	0.100232	NSFL1C/V	2
GO:190481	CC	GO:190481	ficolin-1-ri	15/1071	124/19518	0.003273	0.114069	0.102477	SERPINA1/	15
GO:003243	CC	GO:003243	actin filam	11/1071	78/19518	0.003429	0.114069	0.102477	PALLD/LIM	11
GO:004329	CC	GO:004329	apical junc	17/1071	150/19518	0.003614	0.114069	0.102477	ARHGAP17	17
GO:001652	CC	GO:001652	sarcoplasm	12/1071	90/19518	0.003676	0.114069	0.102477	SERPINB5/	12
GO:003002	CC	GO:003002	lamellipodi	21/1071	202/19518	0.003744	0.114069	0.102477	TIAM2/PAL	21
GO:001636	CC	GO:001636	nuclear ma	15/1071	127/19518	0.004113	0.116321	0.1045	RUVBL2/A	15
GO:000172	CC	GO:000172	stress fiber	10/1071	69/19518	0.004227	0.116321	0.1045	PALLD/LIM	10
GO:009751	CC	GO:009751	contractile	10/1071	69/19518	0.004227	0.116321	0.1045	PALLD/LIM	10
GO:009856	CC	GO:009856	cytoplasmi	20/1071	193/19518	0.004764	0.126998	0.114092	ESYT3/S10	20
GO:000210	CC	GO:000210	podosome	6/1071	30/19518	0.005138	0.132797	0.119302	ARHGEF5/	6
GO:003477	CC	GO:003477	secretory g	29/1071	322/19518	0.006183	0.151245	0.135875	SERPINA1/	29
GO:003017	CC	GO:003017	filopodium	13/1071	108/19518	0.006206	0.151245	0.135875	TIAM2/SPA	13
GO:000989	CC	GO:000989	cytoplasmi	17/1071	159/19518	0.006505	0.151817	0.136389	ESYT3/S10	17
GO:000575	CC	GO:000575	mitochond	40/1071	484/19518	0.006585	0.151817	0.136389	GSTZ1/MR	40
GO:006020	CC	GO:006020	cytoplasmi	29/1071	325/19518	0.007009	0.154512	0.13881	SERPINA1/	29
GO:003242	CC	GO:003242	stereociliur	9/1071	63/19518	0.007147	0.154512	0.13881	RSPH9/EPS	9
GO:002262	CC	GO:002262	proteasom	5/1071	23/19518	0.007246	0.154512	0.13881	PSMC5/PS	5
GO:003198	CC	GO:003198	vesicle lum	29/1071	327/19518	0.007608	0.158285	0.142199	SERPINA1/	29
GO:003141	CC	GO:003141	NatC comp	2/1071	3/19518	0.008695	0.168572	0.151442	NAA30/NA	2
GO:003318	CC	GO:003318	CAF-1 con	2/1071	3/19518	0.008695	0.168572	0.151442	CHAF1B/C	2
GO:009072	CC	GO:009072	central reg	2/1071	3/19518	0.008695	0.168572	0.151442	PAFAH1B1	2
GO:003258	CC	GO:003258	ruffle mem	12/1071	101/19518	0.009255	0.175426	0.157599	KANK1/PL	12
GO:190536	CC	GO:190536	endopeptic	11/1071	90/19518	0.010133	0.18526	0.166433	CRADD/PI	11
GO:003042	CC	GO:003042	growth cor	17/1071	167/19518	0.010434	0.18526	0.166433	TIAM2/CO	17
GO:004261	CC	GO:004261	MHC class	3/1071	9/19518	0.010784	0.18526	0.166433	HLA-B/HL	3
GO:003242	CC	GO:003242	stereociliur	8/1071	56/19518	0.010929	0.18526	0.166433	EPS8L2/M	8
GO:000576	CC	GO:000576	primary lys	16/1071	155/19518	0.011221	0.18526	0.166433	VAMP8/LA	16
GO:004258	CC	GO:004258	azurophil g	16/1071	155/19518	0.011221	0.18526	0.166433	VAMP8/LA	16
GO:003001	CC	GO:003001	Z disc	14/1071	129/19518	0.011296	0.18526	0.166433	PALLD/CA	14
GO:004512	CC	GO:004512	membrane	28/1071	323/19518	0.011511	0.18526	0.166433	PPT1/JAK2	28
GO:009885	CC	GO:009885	membrane	28/1071	324/19518	0.011964	0.185966	0.167068	PPT1/JAK2	28
GO:000568	CC	GO:000568	spliceosom	19/1071	197/19518	0.012208	0.185966	0.167068	PRPF19/SN	19
GO:000588	CC	GO:000588	actin filam	12/1071	105/19518	0.012427	0.185966	0.167068	COBL/PAL	12
GO:000590	CC	GO:000590	brush bord	12/1071	105/19518	0.012427	0.185966	0.167068	DCXR/CLI	12
GO:000591	CC	GO:000591	adherens ju	18/1071	185/19518	0.013297	0.195559	0.175685	BAIAP2L1/	18
GO:003042	CC	GO:003042	site of pola	17/1071	172/19518	0.013718	0.198224	0.17808	TIAM2/CO	17
GO:003577	CC	GO:003577	ribonucleo	24/1071	271/19518	0.014467	0.198224	0.17808	NCL/NFKB	24
GO:003190	CC	GO:003190	early endo	18/1071	187/19518	0.014718	0.198224	0.17808	VAMP8/CD	18
GO:003141	CC	GO:003141	N-termina	3/1071	10/19518	0.014784	0.198224	0.17808	NAA30/NA	3
GO:001601	CC	GO:001601	dystrophin	4/1071	18/19518	0.014873	0.198224	0.17808	SGCE/SSP	4
GO:003152	CC	GO:003152	filopodium	4/1071	18/19518	0.014873	0.198224	0.17808	SYNE2/GA	4
GO:000050	CC	GO:000050	proteasom	8/1071	60/19518	0.016261	0.213361	0.191679	PSMC5/PS	8
GO:003142	CC	GO:003142	RecQ famil	2/1071	4/19518	0.016759	0.213361	0.191679	RMI2/BLM	2
GO:004362	CC	GO:004362	delta DNA	2/1071	4/19518	0.016759	0.213361	0.191679	POLD4/PO	2
GO:000583	CC	GO:000583	proteasom	4/1071	19/19518	0.018038	0.226265	0.203271	PSMC5/PS	4
GO:004320	CC	GO:004320	lysosomal	11/1071	98/19518	0.018419	0.226601	0.203573	HYAL1/PPT	11
GO:009973	CC	GO:009973	cell cortex	6/1071	39/19518	0.018596	0.226601	0.203573	DCTN1/MY	6
GO:000568	CC	GO:000568	U12-type	5/1071	29/19518	0.0195	0.227981	0.204813	SNRPD1/R	5
GO:000081	CC	GO:000081	ESCRT I co	3/1071	11/19518	0.019511	0.227981	0.204813	VPS37B/V	3
GO:004454	CC	GO:004454	NSL compl	3/1071	11/19518	0.019511	0.227981	0.204813	KANSL3/O	3
GO:190536	CC	GO:190536	peptidase	13/1071	125/19518	0.019785	0.228062	0.204886	CRADD/PI	13
GO:000592	CC	GO:000592	bicellular ti	13/1071	126/19518	0.020994	0.235318	0.211405	ARHGAP17	13
GO:014053	CC	GO:014053	endoplasm	13/1071	126/19518	0.020994	0.235318	0.211405	FAF1/EMC	13
GO:000587	CC	GO:000587	microtubul	36/1071	461/19518	0.021321	0.235318	0.211405	EML6/ENK	36
GO:004517	CC	GO:004517	apical part	35/1071	446/19518	0.021518	0.235318	0.211405	MUC1/SLC	35
GO:003152	CC	GO:003152	microvillus	5/1071	30/19518	0.022373	0.237476	0.213343	MSN/EZR/	5
GO:003646	CC	GO:003646	cytoplasmi	22/1071	253/19518	0.022427	0.237476	0.213343	NCL/NFKB	22
GO:004485	CC	GO:004485	plasma me	12/1071	114/19518	0.022551	0.237476	0.213343	JAK2/HAS2	12
GO:003167	CC	GO:003167	I band	14/1071	141/19518	0.02299	0.23915	0.214847	PALLD/CA	14
GO:000031	CC	GO:000031	organellar	10/1071	89/19518	0.023795	0.241636	0.21708	MRPL37/M	10
GO:000576	CC	GO:000576	mitochond	10/1071	89/19518	0.023795	0.241636	0.21708	MRPL37/M	10
GO:000565	CC	GO:000565	nuclear lan	3/1071	12/19518	0.024971	0.24768	0.22251	EIF6/HLCS	3
GO:199006	CC	GO:199006	RPAP3/R2T	3/1071	12/19518	0.024971	0.24768	0.22251	RUVBL2/P	3
GO:003124	CC	GO:003124	protein ace	11/1071	103/19518	0.025674	0.249604	0.224238	NAA30/RU	11
GO:010100	CC	GO:010100	ficolin-1-ri	17/1071	185/19518	0.02607	0.249604	0.224238	SERPINA1/	17
GO:000024	CC	GO:000024	commitme	2/1071	5/19518	0.026921	0.249604	0.224238	SNRPD1/P	2
GO:000218	CC	GO:000218	ribose pho	2/1071	5/19518	0.026921	0.249604	0.224238	PRPS1/PRP	2
GO:004276	CC	GO:004276	GPI-ancho	2/1071	5/19518	0.026921	0.249604	0.224238	PIGT/PIGK	2
GO:009757	CC	GO:009757	lateral part	2/1071	5/19518	0.026921	0.249604	0.224238	NUMA1/G	2
GO:190249	CC	GO:190249	acetyltrans	11/1071	104/19518	0.027342	0.250786	0.2253	NAA30/RU	11
GO:007016	CC	GO:007016	tight juncti	13/1071	131/19518	0.027882	0.253017	0.227304	ARHGAP17	13
GO:000031	CC	GO:000031	organellar	5/1071	32/19518	0.028907	0.256848	0.230747	MRPS9/MF	5
GO:000576	CC	GO:000576	mitochond	5/1071	32/19518	0.028907	0.256848	0.230747	MRPS9/MF	5
GO:001632	CC	GO:001632	apical plas	30/1071	381/19518	0.030266	0.258097	0.231868	MUC1/SLC	30
GO:003049	CC	GO:003049	midbody	18/1071	203/19518	0.030769	0.258097	0.231868	CNTRL/VP	18
GO:003123	CC	GO:003123	extrinsic co	9/1071	80/19518	0.03084	0.258097	0.231868	ESYT3/S10	9
GO:000563	CC	GO:000563	nuclear env	37/1071	490/19518	0.031036	0.258097	0.231868	FAF1/SCR	37
GO:000140	CC	GO:000140	SAM comp	3/1071	13/19518	0.031165	0.258097	0.231868	SAMM50/	3
GO:014027	CC	GO:014027	MIB compl	3/1071	13/19518	0.031165	0.258097	0.231868	SAMM50/	3

GO:002157	BP	GO:002157	rhombome	1/902	1/18614	0.048458	0.335742	0.301347	MAFB	1
GO:002175	BP	GO:002175	caudate nu	1/902	1/18614	0.048458	0.335742	0.301347	FOXP2	1
GO:002175	BP	GO:002175	putamen d	1/902	1/18614	0.048458	0.335742	0.301347	FOXP2	1
GO:003096	BP	GO:003096	peptidyl-a	1/902	1/18614	0.048458	0.335742	0.301347	NDUFAF5	1
GO:003110	BP	GO:003110	dendrite re	1/902	1/18614	0.048458	0.335742	0.301347	PTN	1
GO:003165	BP	GO:003165	negative re	1/902	1/18614	0.048458	0.335742	0.301347	ARRDC3	1
GO:003261	BP	GO:003261	interleukin-	1/902	1/18614	0.048458	0.335742	0.301347	TIRAP	1
GO:003265	BP	GO:003265	regulation	1/902	1/18614	0.048458	0.335742	0.301347	TIRAP	1
GO:003273	BP	GO:003273	positive re	1/902	1/18614	0.048458	0.335742	0.301347	TIRAP	1
GO:003302	BP	GO:003302	negative re	1/902	1/18614	0.048458	0.335742	0.301347	KITLG	1
GO:003336	BP	GO:003336	protein loc	1/902	1/18614	0.048458	0.335742	0.301347	SRGN	1
GO:003336	BP	GO:003336	protease lo	1/902	1/18614	0.048458	0.335742	0.301347	SRGN	1
GO:003337	BP	GO:003337	maintenan	1/902	1/18614	0.048458	0.335742	0.301347	SRGN	1
GO:003337	BP	GO:003337	T cell secre	1/902	1/18614	0.048458	0.335742	0.301347	SRGN	1
GO:003337	BP	GO:003337	maintenan	1/902	1/18614	0.048458	0.335742	0.301347	SRGN	1
GO:003337	BP	GO:003337	protein loc	1/902	1/18614	0.048458	0.335742	0.301347	SRGN	1
GO:003337	BP	GO:003337	protease lo	1/902	1/18614	0.048458	0.335742	0.301347	SRGN	1
GO:003337	BP	GO:003337	maintenan	1/902	1/18614	0.048458	0.335742	0.301347	SRGN	1
GO:003337	BP	GO:003337	maintenan	1/902	1/18614	0.048458	0.335742	0.301347	SRGN	1
GO:003338	BP	GO:003338	granzyme	1/902	1/18614	0.048458	0.335742	0.301347	SRGN	1
GO:003338	BP	GO:003338	maintenan	1/902	1/18614	0.048458	0.335742	0.301347	SRGN	1
GO:003412	BP	GO:003412	negative re	1/902	1/18614	0.048458	0.335742	0.301347	SARM1	1
GO:003414	BP	GO:003414	toll-like rec	1/902	1/18614	0.048458	0.335742	0.301347	TNFAIP3	1
GO:003414	BP	GO:003414	regulation	1/902	1/18614	0.048458	0.335742	0.301347	TNFAIP3	1
GO:003414	BP	GO:003414	negative re	1/902	1/18614	0.048458	0.335742	0.301347	TNFAIP3	1
GO:003435	BP	GO:003435	NAD salva	1/902	1/18614	0.048458	0.335742	0.301347	NAPRT	1
GO:003522	BP	GO:003522	regulation	1/902	1/18614	0.048458	0.335742	0.301347	GCLM	1
GO:003522	BP	GO:003522	positive re	1/902	1/18614	0.048458	0.335742	0.301347	GCLM	1
GO:003554	BP	GO:003554	positive re	1/902	1/18614	0.048458	0.335742	0.301347	SNCA	1
GO:003556	BP	GO:003556	regulation	1/902	1/18614	0.048458	0.335742	0.301347	HNF1B	1
GO:003556	BP	GO:003556	N-termina	1/902	1/18614	0.048458	0.335742	0.301347	NTMT1	1
GO:003557	BP	GO:003557	N-termina	1/902	1/18614	0.048458	0.335742	0.301347	NTMT1	1
GO:003557	BP	GO:003557	N-termina	1/902	1/18614	0.048458	0.335742	0.301347	NTMT1	1
GO:003557	BP	GO:003557	N-termina	1/902	1/18614	0.048458	0.335742	0.301347	NTMT1	1
GO:003564	BP	GO:003564	enteric sm	1/902	1/18614	0.048458	0.335742	0.301347	EDNRB	1
GO:003566	BP	GO:003566	TIRAP-dep	1/902	1/18614	0.048458	0.335742	0.301347	TIRAP	1
GO:003566	BP	GO:003566	TIRAP-dep	1/902	1/18614	0.048458	0.335742	0.301347	TIRAP	1
GO:003569	BP	GO:003569	mitophagy	1/902	1/18614	0.048458	0.335742	0.301347	SPATA18	1
GO:003607	BP	GO:003607	ligamentou	1/902	1/18614	0.048458	0.335742	0.301347	RUNX2	1
GO:004227	BP	GO:004227	error-free	1/902	1/18614	0.048458	0.335742	0.301347	UBE2V2	1
GO:004266	BP	GO:004266	regulation	1/902	1/18614	0.048458	0.335742	0.301347	FZD7	1
GO:004266	BP	GO:004266	negative re	1/902	1/18614	0.048458	0.335742	0.301347	FZD7	1
GO:004277	BP	GO:004277	plasma me	1/902	1/18614	0.048458	0.335742	0.301347	COA6	1
GO:004433	BP	GO:004433	canonical	1/902	1/18614	0.048458	0.335742	0.301347	CTNNB1	1
GO:004460	BP	GO:004460	protein de	1/902	1/18614	0.048458	0.335742	0.301347	FICD	1
GO:004460	BP	GO:004460	protein dea	1/902	1/18614	0.048458	0.335742	0.301347	FICD	1
GO:004645	BP	GO:004645	hexadecan	1/902	1/18614	0.048458	0.335742	0.301347	ALDH3A2	1
GO:004670	BP	GO:004670	CDP metab	1/902	1/18614	0.048458	0.335742	0.301347	CMPK1	1
GO:004670	BP	GO:004670	CDP biosyn	1/902	1/18614	0.048458	0.335742	0.301347	CMPK1	1
GO:004672	BP	GO:004672	negative re	1/902	1/18614	0.048458	0.335742	0.301347	STAT1	1
GO:004877	BP	GO:004877	erythropho	1/902	1/18614	0.048458	0.335742	0.301347	SOD2	1
GO:005158	BP	GO:005158	negative re	1/902	1/18614	0.048458	0.335742	0.301347	SNCA	1
GO:005162	BP	GO:005162	negative re	1/902	1/18614	0.048458	0.335742	0.301347	SNCA	1
GO:005194	BP	GO:005194	negative re	1/902	1/18614	0.048458	0.335742	0.301347	SNCA	1
GO:005280	BP	GO:005280	imidazole-	1/902	1/18614	0.048458	0.335742	0.301347	HNMT	1
GO:006001	BP	GO:006001	Sertoli cell	1/902	1/18614	0.048458	0.335742	0.301347	RARA	1
GO:006034	BP	GO:006034	spleen trab	1/902	1/18614	0.048458	0.335742	0.301347	SLC40A1	1
GO:006061	BP	GO:006061	mammary	1/902	1/18614	0.048458	0.335742	0.301347	CSF1	1
GO:006068	BP	GO:006068	regulation	1/902	1/18614	0.048458	0.335742	0.301347	PDGFA	1
GO:006085	BP	GO:006085	establishm	1/902	1/18614	0.048458	0.335742	0.301347	MXRA8	1
GO:006090	BP	GO:006090	regulation	1/902	1/18614	0.048458	0.335742	0.301347	HSPA5	1
GO:006120	BP	GO:006120	mesoneph	1/902	1/18614	0.048458	0.335742	0.301347	HNF1B	1
GO:006121	BP	GO:006121	mesoneph	1/902	1/18614	0.048458	0.335742	0.301347	HNF1B	1
GO:006121	BP	GO:006121	negative re	1/902	1/18614	0.048458	0.335742	0.301347	HNF1B	1
GO:006122	BP	GO:006122	mesoneph	1/902	1/18614	0.048458	0.335742	0.301347	HNF1B	1
GO:006123	BP	GO:006123	mesenchyr	1/902	1/18614	0.048458	0.335742	0.301347	HNF1B	1
GO:006129	BP	GO:006129	regulation	1/902	1/18614	0.048458	0.335742	0.301347	HNF1B	1
GO:006129	BP	GO:006129	negative re	1/902	1/18614	0.048458	0.335742	0.301347	HNF1B	1
GO:006132	BP	GO:006132	canonical	1/902	1/18614	0.048458	0.335742	0.301347	CTNNB1	1
GO:006167	BP	GO:006167	evoked ne	1/902	1/18614	0.048458	0.335742	0.301347	RAB3A	1
GO:006176	BP	GO:006176	negative re	1/902	1/18614	0.048458	0.335742	0.301347	SMAD3	1
GO:006203	BP	GO:006203	sensory pe	1/902	1/18614	0.048458	0.335742	0.301347	WDR47	1
GO:006217	BP	GO:006217	lutein meta	1/902	1/18614	0.048458	0.335742	0.301347	BCO2	1
GO:006217	BP	GO:006217	lutein catal	1/902	1/18614	0.048458	0.335742	0.301347	BCO2	1
GO:007026	BP	GO:007026	oncosis	1/902	1/18614	0.048458	0.335742	0.301347	TMEM123	1
GO:007042	BP	GO:007042	negative re	1/902	1/18614	0.048458	0.335742	0.301347	TNFAIP3	1
GO:007104	BP	GO:007104	nuclear his	1/902	1/18614	0.048458	0.335742	0.301347	SSB	1
GO:007130	BP	GO:007130	cellular res	1/902	1/18614	0.048458	0.335742	0.301347	COL1A1	1
GO:007144	BP	GO:007144	cellular res	1/902	1/18614	0.048458	0.335742	0.301347	MGST1	1
GO:007152	BP	GO:007152	tRNA re-e	1/902	1/18614	0.048458	0.335742	0.301347	XPOT	1
GO:007230	BP	GO:007230	negative re	1/902	1/18614	0.048458	0.335742	0.301347	STAT1	1
GO:007270	BP	GO:007270	response t	1/902	1/18614	0.048458	0.335742	0.301347	TP53INP1	1
GO:007270	BP	GO:007270	cellular res	1/902	1/18614	0.048458	0.335742	0.301347	TP53INP1	1
GO:008005	BP	GO:008005	protein de	1/902	1/18614	0.048458	0.335742	0.301347	GLRX	1
GO:008501	BP	GO:008501	entry into	1/902	1/18614	0.048458	0.335742	0.301347	ITGAV	1
GO:009034	BP	GO:009034	negative re	1/902	1/18614	0.048458	0.335742	0.301347	NR1H3	1
GO:009728	BP	GO:009728	immune co	1/902	1/18614	0.048458	0.335742	0.301347	TREX1	1
GO:009732	BP	GO:009732	response t	1/902	1/18614	0.048458	0.335742	0.301347	PTGR1	1
GO:009803	BP	GO:009803	non-replica	1/902	1/18614	0.048458	0.335742	0.301347	PGBD5	1
GO:009897	BP	GO:009897	anterograd	1/902	1/18614	0.048458	0.335742	0.301347	TRAK2	1
GO:009916	BP	GO:009916	regulation	1/902	1/18614	0.048458	0.335742	0.301347	RAB3A	1
GO:009955	BP	GO:009955	trans-syna	1/902	1/18614	0.048458	0.335742	0.301347	NTRK2	1
GO:009957	BP	GO:009957	regulation	1/902	1/18614	0.048458	0.335742	0.301347	FBXL20	1
GO:011001	BP	GO:011001	cap-indep	1/902	1/18614	0.048458	0.335742	0.301347	RBM4	1
GO:012016	BP	GO:012016	detection	1/902	1/18614	0.048458	0.335742	0.301347	WDR47	1
GO:014032	BP	GO:014032	lysophosp	1/902	1/18614	0.048458	0.335742	0.301347	MFSD2A	1
GO:014049	BP	GO:014049	very long-	1/902	1/18614	0.048458	0.335742	0.301347	ACOX1	1
GO:015017	BP	GO:015017	regulation	1/902	1/18614	0.048458	0.335742	0.301347	MFSD2A	1
GO:015017	BP	GO:015017	regulation	1/902	1/18614	0.048458	0.335742	0.301347	MFSD2A	1
GO:190114	BP	GO:190114	mesenchyr	1/902	1/18614	0.048458	0.335742	0.301347	HNF1B	1
GO:190117	BP	GO:190117	lycopene n	1/902	1/18614	0.048458	0.335742	0.301347	BCO2	1
GO:190117	BP	GO:190117	lycopene c	1/902	1/18614	0.048458	0.335742	0.301347	BCO2	1
GO:190130	BP	GO:190130	regulation	1/902	1/18614	0.048458	0.335742	0.301347	PAOX	1
GO:190130	BP	GO:190130	positive re	1/902	1/18614	0.048458	0.335742	0.301347	PAOX	1
GO:190162	BP	GO:190162	positive re	1/902	1/18614	0.048458	0.335742	0.301347	GORAB	1
GO:190182	BP	GO:190182	zeaxanthin	1/902	1/18614	0.048458	0.335742	0.301347	BCO2	1
GO:190220	BP	GO:190220	regulation	1/902	1/18614	0.048458	0.335742	0.301347	PTPN2	1
GO:190220	BP	GO:190220	negative re	1/902	1/18614	0.048458	0.335742	0.301347	PTPN2	1
GO:190221	BP	GO:190221	negative re	1/902	1/18614	0.048458	0.335742	0.301347	PTPN2	1
GO:190222	BP	GO:190222	positive re	1/902	1/18614	0.048458	0.335742	0.301347	CSF1	1
GO:190223	BP	GO:190223	negative re	1/902	1/18614	0.048458	0.335742	0.301347	PTPN2	1
GO:190328	BP	GO:190328	positive re	1/902	1/18614	0.048458	0.335742	0.301347	SNCA	1
GO:190328	BP	GO:190328	positive re	1/902	1/18614	0.048458	0.335742	0.301347	SNCA	1
GO:190370	BP	GO:190370	substantia	1/902	1/18614	0.048458	0.335742	0.301347	TGFB2	1
GO:190398	BP	GO:190398	iron ion ex	1/902	1/18614	0.048458	0.335742	0.301347	SLC40A1	1
GO:190400	BP	GO:190400	negative re	1/902	1/18614	0.048458	0.335742	0.301347	GATA6	1
GO:190409	BP	GO:190409	positive re	1/902	1/18614	0.048458	0.335742	0.301347	DAPK1	1
GO:190421	BP	GO:190421	VCP-NPL4	1/902	1/18614	0.048458	0.335742	0.301347	SVIP	1
GO:190423	BP	GO:190423	regulation	1/902	1/18614	0.048458	0.335742	0.301347	SVIP	1
GO:190424	BP	GO:190424	negative re	1/902	1/18614	0.048458	0.335742	0.301347	SVIP	1
GO:190440	BP	GO:190440	cellular res	1/902	1/18614	0.048458	0.335742	0.301347	SLC5A5	1
GO:190443	BP	GO:190443	negative re	1/902	1/18614	0.048458	0.335742	0.301347	ISCU	1
GO:190479	BP	GO:190479	negative re	1/902	1/18614	0.048458	0.335742	0.301347	ZNF827	1
GO:190504	BP	GO:190504	negative re	1/902	1/18614	0.048458	0.335742	0.301347	DUSP10	1
GO:199026	BP	GO:199026	pre-mRNA	1/902	1/18614	0.048458	0.335742	0.301347	ZPR1	1
GO:199046	BP	GO:199046	protein aut	1/902	1/18614	0.048458	0.335742	0.301347	ZBED1	1
GO:200075	BP	GO:200075	regulation	1/902	1/18614	0.048458	0.335742	0.301347	SOX4	1
GO:200076	BP	GO:200076	positive re	1/902	1/18614	0.048458	0.335742	0.301347	SOX4	1
GO:200110	BP	GO:200110	regulation	1/902	1/18614	0.048458	0.335742	0.301347	CCPG1	1
GO:200114	BP	GO:200114	regulation	1/902	1/18614	0.048458	0.335742	0.301347	BAG4	1
GO:200114	BP	GO:200114	negative re	1/902	1/18614	0.048458	0.335742	0.301347	BAG4	1
GO:200119	BP	GO:200119	regulation	1/902	1/18614	0.048458	0.335742	0.301347	LILRB1	1
GO:200119	BP	GO:200119	positive re	1/902	1/18614	0.048458	0.335742	0.301347	LILRB1	1
GO:004254	BP	GO:004254	response t	11/902	129/18614	0.048807	0.33714	0.302602	CYP1B1/T	11
GO:200002	BP	GO:200002	regulation	11/902	129/18614	0.048807	0.33714	0.302602	GREM1/RS	11
GO:000608	BP	GO:000608	cellular ald	7/902	69/18614	0.04908	0.33714	0.302602	CYP1B1/A	7
GO:004209	BP	GO:004209	T-helper c	7/902	69/18614	0.04908	0.33714	0.302602	BCL3/FOX	7
GO:000322	BP	GO:000322	ventricular	6/902	55/18614	0.049208	0.33714	0.302602	IRX3/TGFB	6
GO:005237	BP	GO:005237	modulation	6/902	55/18614	0.049208	0.33714	0.302602	TRIM8/TRI	6
GO:001982	BP	GO:001982	stem cell p	14/902	177/18614	0.049217	0.33714	0.302602	TRIM8/FZD	14
GO:005114	BP	GO:005114	regulation	13/902	161/18614	0.049485	0.33714	0.302602	FZD7/CYP2	13
GO:000717	BP	GO:000717	transmemb	27/902	398/18614	0.049603	0.33714	0.302602	GREM1/EN	27
GO:004647	BP	GO:004647	glyceropho	16/902	210/18614	0.049658	0.33714	0.302602	SOCS6/MF	16
GO:000030	BP	GO:000030	response t	4/902	29/18614	0.049668	0.33714	0.302602	NQO1/SO	4
GO:000041	BP	GO:000041	protein pe	4/902	29/18614	0.049668	0.33714	0.302602	FKBP11/PP	4
GO:000156	BP	GO:000156	response t	4/902	29/18614	0.049668	0.33714	0.302602	BCL3/MYD	4
GO:000177	BP	GO:000177	plasma me	4/902	29/18614	0.049668	0.33714	0.302602	CHMP5/CA	4
GO:000320	BP	GO:000320	cardiac atr	4/902	29/18614	0.049668	0.33714	0.302602	TGFB2/SO	4
GO:000665	BP	GO:000665	phosphatic	4/902	29/18614	0.049668	0.33714	0.302602	MFSD2A/C	4
GO:000926	BP	GO:000926	deoxyribor	4/902	29/18614	0.049668	0.33714	0.302602	NTHL1/MB	4
GO:003144	BP	GO:003144	regulation	4/902	29/18614	0.049668	0.33714	0.302602	SAMD1/PI	4
GO:004638	BP	GO:004638	deoxyribos	4/902	29/18614	0.049668	0.33714	0.302602	NTHL1/MB	4
GO:012026	BP	GO:012026	regulation	4/902	29/18614	0.049668	0.33714	0.302602	SAMD1/PI	4
GO:006151	BP	GO:006151	myeloid ce	8/902	84/18614	0.050507	0.342473	0.307388	SH2B3/FO	8
GO:004866	BP	GO:004866	positive re	9/902	99/18614	0.050659	0.342473	0.307388	ID2/FOXP1	9
GO:006034	BP	GO:006034	bone morp	9/902	99/18614	0.050659	0.342473	0.307388	CYP26B1/	9
GO:200123	BP	GO:200123	negative re	9/902	99/18614	0.050659	0.342473	0.307388	TNFAIP3/E	9
GO:000020	BP	GO:000020	protein pol	18/902	244/18614	0.050777	0.342926	0.307795	ITCH/SYV	18
GO:005086	BP	GO:005086	regulation	11/902	130/18614	0.051096	0.343895	0.308665	ZFP36L1/A	11
GO:004512	BP	GO:004512	regulation	5/902	42/18614	0.051126	0.343895	0.308665	TNFAIP3/C	5
GO:004542	BP	GO:004542	positive re	5/902	42/18614	0.051126	0.343895	0.308665	HBB/HSP9	5
GO:006033	BP	GO:006033	regulation	5/902	42/18614	0.051126	0.343895	0.308665	PTPN2/ISG	5
GO:003510	BP	GO:003510	appendage	12/902	146/18614	0.051553	0.346068	0.310615	GREM1/TG	12
GO:003510	BP	GO:003510	limb morpl	12/902	146/18614	0.051553	0.346068	0.310615	GREM1/TG	12
GO:000222	BP	GO:000222	toll-like rec	7/902	70/18614	0.052358	0.347268	0.311692	IRF1/LY96/	7
GO:004311	BP	GO:004311	receptor m	7/902	70/18614	0.052358	0.347268	0.311692	ITCH/CHM	7
GO:005114	BP	GO:005114	negative re	7/902	70/18614	0.052358	0.347268	0.311692	FZD7/ID2/	7
GO:005134	BP	GO:005134	negative re	20/902	279/18614	0.052612	0.347268	0.311692	TRIB3/BAG	20
GO:007066	BP	GO:007066	leukocyte	24/902	348/18614	0.052756	0.347268	0.311692	GREM1/IR	24
GO:000038	BP	GO:000038	regulation	6/902	56/18614	0.052955	0.347268	0.311692	RBM20/TIA	6
GO:003051	BP	GO:003051	negative re	9/902	100/18614	0.053391	0.347268	0.311692	EMILIN1/H	9
GO:006132	BP	GO:006132	renal tubul	9/902	100/18614	0.053391	0.347268	0.311692	GREM1/IRX	9
GO:005086	BP	GO:005086	negative re	11/902	131/18614	0.053457	0.347268	0.311692	IRF1/ITCH/	11
GO:004586	BP	GO:004586	positive re	25/902	366/18614	0.053551	0.347268	0.311692	GREM1/CC	25
GO:000660	BP	GO:000660	protein imp	13/902	163/18614	0.053635	0.347268	0.311692	SUMO1/BC	13
GO:000697	BP	GO:000697	DNA dama	3/902	18/18614	0.053753	0.347268	0.311692	RPS27L/ZN	3
GO:003235	BP	GO:003235	response t	3/902	18/18614	0.053753	0.347268	0.311692	CYP1B1/G	3
GO:003649	BP	GO:003649	PERK-med	3/902	18/18614	0.053753	0.347268	0.311692	HSPA5/PTP	3
GO:004240	BP	GO:004240	cellular bio	3/902	18/18614	0.053753	0.347268	0.311692	SLC44A1/P	3
GO:004257	BP	GO:004257	retinal met	3/902	18/18614	0.053753	0.347268	0.311692	CYP1B1/A	3
GO:005074	BP	GO:005074	regulation	3/902	18/18614	0.053753	0.347268	0.311692	SVIP/ULK1	3
GO:006195	BP	GO:006195	midbody a	3/902	18/18614	0.053753	0.347268	0.311692	CHMP5/MI	3
GO:007107	BP	GO:007107	regulation	3/902	18/18614	0.053753	0.347268	0.311692	HTR2A/PD	3
GO:007207	BP	GO:007207	renal vesicl	3/902	18/18614	0.053753	0.347268	0.311692	GREM1/ST	3
GO:009008	BP	GO:009008	regulation	3/902	18/18614	0.053753	0.347268	0.311692	DNAJB6/H	3
GO:000665	BP	GO:000665	glyceropho	21/902	297/18614	0.054004	0.347268	0.311692	SOCS6/NR	21
GO:000223	BP	GO:000223	leukocyte	2/902	8/18614	0.054069	0.347268	0.311692	PTN/NINJ1	2
GO:000253	BP	GO:000253	respiratory	2/902	8/18614	0.054069	0.347268	0.311692	DUSP10/P	2
GO:000343	BP	GO:000343	chondrocy	2/902	8/18614	0.054069	0.347268	0.311692	SERPINH1/	2
GO:000672	BP	GO:000672	tetrahydrol	2/902	8/18614	0.054069	0.347268	0.311692	SPR/PCBD	2
GO:000725	BP	GO:000725	cytoplasmi	2/902	8/18614	0.054069	0.347268	0.311692	PSMD10/G	2
GO:001063	BP	GO:001063	negative re	2/902	8/18614	0.054069	0.347268	0.311692	VAT1/OMA	2
GO:001594	BP	GO:001594	nucleobase	2/902	8/18614	0.054069	0.347268	0.311692	CMPK1/GL	2
GO:003085	BP	GO:003085	negative re	2/902	8/18614	0.054069	0.347268	0.311692	INPP5D/RA	2
GO:004564	BP	GO:004564	negative re	2/902	8/18614	0.054069	0.347268	0.311692	MAFB/ZFP	2
GO:004565	BP	GO:004565	negative re	2/902	8/18614	0.054069	0.347268	0.311692	HOXA7/IN	2
GO:006048	BP	GO:006048	lung-assoc	2/902	8/18614	0.054069	0.347268	0.311692	FGF9/CTN	2
GO:006075	BP	GO:006075	regulation	2/902	8/18614	0.054069	0.347268	0.311692	VEGFC/VE	2
GO:007043	BP	GO:007043	regulation	2/902	8/18614	0.054069	0.347268	0.311692	TNFAIP3/H	2
GO:012018	BP	GO:012018	cell-substr	2/902	8/18614	0.054069	0.347268	0.311692	MAP4K4/D	2
GO:012018	BP	GO:012018	focal adhe	2/902	8/18614	0.054069	0.347268	0.311692	MAP4K4/D	2
GO:190374	BP	GO:190374	negative re	2/902	8/18614	0.054069	0.347268	0.311692	BAG4/LMA	2
GO:190375	BP	GO:190375	negative re	2/902	8/18614	0.054069	0.347268	0.311692	DUSP10/D	2
GO:190402	BP	GO:190402	regulation	2/902	8/18614	0.054069	0.347268	0.311692	EMILIN1/R	2
GO:190438	BP	GO:190438	response t	2/902	8/18614	0.054069	0.347268	0.311692	EDNRB/RU	2
GO:190582	BP	GO:190582	positive re	2/902	8/18614	0.054069	0.347268	0.311692	SMC4/SMC	2
GO:199026	BP	GO:199026	peptidyl-ty	2/902	8/18614	0.054069	0.347268	0.311692	DUSP10/D	2
GO:199070	BP	GO:199070	presynapti	2/902	8/18614	0.054069	0.347268	0.311692	RAB3A/CA	2
GO:200058	BP	GO:200058	regulation	2/902	8/18614	0.054069	0.347268	0.311692	PTPN2/PTP	2
GO:003444	BP	GO:003444	lipid oxidat	10/902	116/18614	0.055073	0.353037	0.31687	CYGB/MFS	10
GO:007200	BP	GO:007200	nephron ep	10/902	116/18614	0.055073	0.353037	0.31687	GREM1/IRX	10
GO:005105	BP	GO:005105	regulation	21/902	298/18614	0.055575	0.354445	0.318134	MAP4K4/T	21
GO:000628	BP	GO:000628	base-excis	5/902	43/18614	0.055646	0.354445	0.318134	NTHL1/MB	5
GO:001046	BP	GO:001046	mesenchyr	5/902	43/18614	0.055646	0.354445	0.318134	SHOX2/ST	5
GO:001626	BP	GO:001626	O-glycan p	5/902	43/18614	0.055646	0.354445	0.318134	ST6GALNA	5
GO:003195	BP	GO:003195	regulation	5/902	43/18614	0.055646	0.354445	0.318134	GREM1/RA	5
GO:003414	BP	GO:003414	toll-like rec	5/902	43/18614	0.055646	0.354445	0.318134	LY96/NR1H	5
GO:000229	BP	GO:000229	CD4-positi	7/902	71/18614	0.05577	0.354445	0.318134	BCL3/FOX	7
GO:004560	BP	GO:004560	positive re	7/902	71/18614	0.05577	0.354445	0.318134	ZFP36L1/ID	7
GO:004800	BP	GO:004800	antigen pro	7/902	71/18614	0.05577	0.354445	0.318134	ERAP1/HL	7
GO:006004	BP	GO:006004	heart contr	18/902	247/18614	0.055885	0.354529	0.318209	GATA6/IRX	18
GO:000224	BP	GO:000224	hematopoi	11/902	132/18614	0.055889	0.354529	0.318209	ITCH/SH2E	11
GO:003133	BP	GO:003133	negative re	12/902	148/18614	0.056074	0.355366	0.318961	HSPA5/CA	12
GO:004325	BP	GO:004325	regulation	9/902	101/18614	0.056217	0.355936	0.319472	DYRK2/PD	9
GO:000862	BP	GO:000862	extrinsic ap	8/902	86/18614	0.056602	0.357697	0.321053	PMAIP1/TN	8
GO:004804	BP	GO:004804	focal adhe	8/902	86/18614	0.056602	0.357697	0.321053	GREM1/M	8
GO:003386	BP	GO:003386	nucleoside	6/902	57/18614	0.056871	0.357705	0.32106	PDK2/DLA	6
GO:003403	BP	GO:003403	ribonucleo	6/902	57/18614	0.056871	0.357705	0.32106	PDK2/DLA	6
GO:003403	BP	GO:003403	purine nucl	6/902	57/18614	0.056871	0.357705	0.32106	PDK2/DLA	6
GO:003433	BP	GO:003433	adherens ju	6/902	57/18614	0.056871	0.357705	0.32106	CDH11/CD	6
GO:005043	BP	GO:005043	regulation	6/902	57/18614	0.056871	0.357705	0.32106	SDHD/SYT	6
GO:003010	BP	GO:003010	regulation	16/902	214/18614	0.05702	0.357966	0.321295	GREM1/RA	16
GO:190280	BP	GO:190280	regulation	16/902	214/18614	0.05702	0.357966	0.321295	CCND1/RP	16
GO:005109	BP	GO:005109	regulation	25/902	369/18614	0.057819	0.362645	0.325494	TRIB3/BAG	25
GO:000038	BP	GO:000038	alternative	7/902	72/18614	0.059317	0.370211	0.332285	RBM20/TIA	7
GO:000228	BP	GO:000228	alpha-beta	7/902	72/18614	0.059317	0.370211	0.332285	BCL3/FOX	7
GO:000229	BP	GO:000229	alpha-beta	7/902	72/18614	0.059317	0.370211	0.332285	BCL3/FOX	7
GO:003033	BP	GO:003033	DNA dama	7/902	72/18614	0.059317	0.370211	0.332285	RPS27L/PM	7
GO:190488	BP	GO:190488	cranial skel	7/902	72/18614	0.059317	0.370211	0.332285	IRX5/TGFB	7
GO:004348	BP	GO:004348	regulation	14/902	182/18614	0.059413	0.370211	0.332285	RBM20/CD	14
GO:004689	BP	GO:004689	regulation	14/902	182/18614	0.059413	0.370211	0.332285	TRIB3/NR1	14
GO:003300	BP	GO:003300	muscle cell	18/902	249/18614	0.059482	0.370297	0.332362	GATA6/IGF	18
GO:000196	BP	GO:000196	negative re	8/902	87/18614	0.059818	0.371347	0.333305	ZNF675/N	8
GO:001023	BP	GO:001023	vascular tr	8/902	87/18614	0.059818	0.371347	0.333305	MFSD2A/S	8
GO:015010	BP	GO:015010	transport a	8/902	87/18614	0.059818	0.371347	0.333305	MFSD2A/S	8
GO:000225	BP	GO:000225	activation	32/902	495/18614	0.06002	0.372051	0.333937	IRF1/ITCH/	32
GO:003196	BP	GO:003196	response t	13/902	166/18614	0.060294	0.372051	0.333937	CCND1/PT	13
GO:000170	BP	GO:000170	cell fate de	5/902	44/18614	0.060388	0.372051	0.333937	GATA6/CY	5
GO:000188	BP	GO:000188	receptor re	5/902	44/18614	0.060388	0.372051	0.333937	CHMP5/PT	5
GO:000234	BP	GO:000234	response t	5/902	44/18614	0.060388	0.372051	0.333937	AHR/TXNI	5
GO:190440	BP	GO:190440	positive re	5/902	44/18614	0.060388	0.372051	0.333937	HBB/HSP9	5
GO:200078	BP	GO:200078	regulation	5/902	44/18614	0.060388	0.372051	0.333937	SNX18/IFT	5
GO:200037	BP	GO:200037	regulation	12/902	150/18614	0.060854	0.372051	0.333937	CYP1B1/A	12
GO:000183	BP	GO:000183	release of	6/902	58/18614	0.060956	0.372051	0.333937	PMAIP1/BN	6
GO:001071	BP	GO:001071	positive re	6/902	58/18614	0.060956	0.372051	0.333937	TGFB2/EN	6
GO:003022	BP	GO:003022	macropha	6/902	58/18614	0.060956	0.372051	0.333937	ID2/PTPN2	6
GO:005073	BP	GO:005073	negative re	6/902	58/18614	0.060956	0.372051	0.333937	SH2B3/PTK	6
GO:006100	BP	GO:006100	cell differe	6/902	58/18614	0.060956	0.372051	0.333937	GREM1/GL	6
GO:009735	BP	GO:009735	autophago	6/902	58/18614	0.060956	0.372051	0.333937	CHMP5/LA	6
GO:001714	BP	GO:001714	stem cell d	4/902	31/18614	0.061044	0.372051	0.333937	TGFB2/FZD	4
GO:003368	BP	GO:003368	regulation	4/902	31/18614	0.061044	0.372051	0.333937	GREM1/NP	4
GO:003625	BP	GO:003625	multivesicu	4/902	31/18614	0.061044	0.372051	0.333937	CHMP5/ST	4
GO:007127	BP	GO:007127	cellular res	4/902	31/18614	0.061044	0.372051	0.333937	SUMO1/CH	4
GO:007252	BP	GO:007252	pyridine-c	4/902	31/18614	0.061044	0.372051	0.333937	NADK2/NA	4
GO:015011	BP	GO:015011	positive re	4/902	31/18614	0.061044	0.372051	0.333937	MAP4K4/L	4
GO:000166	BP	GO:000166	ameboidal	32/902	496/18614	0.061322	0.372107	0.333986	GREM1/M	32
GO:000679	BP	GO:000679	sulfur comp	22/902	319/18614	0.061577	0.372107	0.333986	DSE/DSEL/	22
GO:000719	BP	GO:000719	negative re	3/902	19/18614	0.06161	0.372107	0.333986	NPR3/GRM	3
GO:004277	BP	GO:004277	DNA dama	3/902	19/18614	0.06161	0.372107	0.333986	RPS27L/ZN	3
GO:005179	BP	GO:005179	regulation	3/902	19/18614	0.06161	0.372107	0.333986	TGFB2/MY	3
GO:007208	BP	GO:007208	renal vesicl	3/902	19/18614	0.06161	0.372107	0.333986	GREM1/ST	3
GO:009030	BP	GO:009030	regulation	3/902	19/18614	0.06161	0.372107	0.333986	SAMD1/ZN	3
GO:009720	BP	GO:009720	activation	3/902	19/18614	0.06161	0.372107	0.333986	CYFIP2/IFI1	3
GO:014035	BP	GO:014035	lipid impor	3/902	19/18614	0.06161	0.372107	0.333986	SLC27A2/F	3
GO:200078	BP	GO:200078	positive re	3/902	19/18614	0.06161	0.372107	0.333986	SNX18/UL	3
GO:000688	BP	GO:000688	regulation	9/902	103/18614	0.062156	0.375066	0.336642	PDK2/ATP	9
GO:001605	BP	GO:001605	Wnt signali	30/902	461/18614	0.062532	0.376996	0.338374	GREM1/CC	30
GO:000317	BP	GO:000317	heart valve	7/902	73/18614	0.063	0.378526	0.339748	EMILIN1/T	7

GO:199090	CC	GO:199090	beta-cater	3/1071	13/19518	0.031165	0.258097	0.231868	TCF7L1/TC	3
GO:190256	CC	GO:190256	H4 histone	6/1071	44/19518	0.031978	0.262279	0.235625	RUVBL2/K/	6
GO:000590	CC	GO:000590	microvillus	10/1071	94/19518	0.033215	0.268247	0.240987	DCXR/MSN	10
GO:006202	CC	GO:006202	collagen-c	32/1071	415/19518	0.033334	0.268247	0.240987	SERPINA1/	32
GO:009066	CC	GO:009066	glycoprote	4/1071	23/19518	0.034667	0.276365	0.24828	SGCE/SSPN	4
GO:009054	CC	GO:009054	Flemming I	5/1071	34/19518	0.036532	0.288538	0.259216	CNTRL/US	5
GO:000590	CC	GO:000590	caveola	9/1071	83/19518	0.037871	0.288742	0.259399	JAK2/PACS	9
GO:003067	CC	GO:003067	axolemma	3/1071	14/19518	0.038085	0.288742	0.259399	KCNC2/RC	3
GO:003108	CC	GO:003108	BLOC com	3/1071	14/19518	0.038085	0.288742	0.259399	BCAS4/BL(	3
GO:000565	CC	GO:000565	nucleolar ri	2/1071	6/19518	0.038928	0.288742	0.259399	POP7/POP:	2
GO:001601	CC	GO:001601	sarcoglyca	2/1071	6/19518	0.038928	0.288742	0.259399	SGCE/SGC	2
GO:008970	CC	GO:008970	U2AF com	2/1071	6/19518	0.038928	0.288742	0.259399	ZRSR2/SF1	2
GO:009742	CC	GO:009742	microtubul	2/1071	6/19518	0.038928	0.288742	0.259399	TPPP3/NUI	2
GO:004429	CC	GO:004429	axonal gro	4/1071	24/19518	0.039851	0.292581	0.262848	COBL/NEC	4
GO:000191	CC	GO:000191	photorece	8/1071	71/19518	0.040131	0.292581	0.262848	ASRGL1/M	8
GO:001049	CC	GO:001049	cytoplasmi	9/1071	85/19518	0.043122	0.305923	0.274834	EIF2S1/PAI	9
GO:004247	CC	GO:004247	melanosor	11/1071	112/19518	0.043588	0.305923	0.274834	LAMP1/AN	11
GO:004877	CC	GO:004877	pigment gi	11/1071	112/19518	0.043588	0.305923	0.274834	LAMP1/AN	11
GO:004261	CC	GO:004261	MHC prote	4/1071	25/19518	0.045452	0.305923	0.274834	HLA-B/HL/	4
GO:003101	CC	GO:003101	Ino80 com	3/1071	15/19518	0.045715	0.305923	0.274834	RUVBL2/IN	3
GO:003329	CC	GO:003329	eukaryotic	3/1071	15/19518	0.045715	0.305923	0.274834	EIF3D/EIF2	3
GO:001652	CC	GO:001652	sarcoplasm	8/1071	73/19518	0.046146	0.305923	0.274834	ANK3/ITPR	8
GO:005503	CC	GO:005503	recycling e	17/1071	200/19518	0.049028	0.305923	0.274834	VAMP8/TN	17
GO:001660	CC	GO:001660	nuclear spe	31/1071	415/19518	0.050921	0.305923	0.274834	RMI2/RBM	31
GO:009734	CC	GO:009734	INO80-typ	4/1071	26/19518	0.051468	0.305923	0.274834	RUVBL2/IN	4
GO:000050	CC	GO:000050	glycosylph	2/1071	7/19518	0.052546	0.305923	0.274834	PIGC/PIGH	2
GO:001601	CC	GO:001601	dystroglyca	2/1071	7/19518	0.052546	0.305923	0.274834	SGCE/SGC	2
GO:003139	CC	GO:003139	Ctf18 RFC-	2/1071	7/19518	0.052546	0.305923	0.274834	RFC4/CHTI	2
GO:003644	CC	GO:003644	microtubul	2/1071	7/19518	0.052546	0.305923	0.274834	NUMA1/C/	2
GO:007154	CC	GO:007154	eukaryotic	2/1071	7/19518	0.052546	0.305923	0.274834	EIF3D/EIF3	2
GO:015003	CC	GO:015003	distal axon	22/1071	277/19518	0.052815	0.305923	0.274834	TIAM2/CO	22
GO:000574	CC	GO:000574	mitochond	36/1071	497/19518	0.054676	0.305923	0.274834	MRPL37/E(	36
GO:001712	CC	GO:001712	protein N-	1/1071	1/19518	0.054872	0.305923	0.274834	OGT	1
GO:003069	CC	GO:003069	Noc1p-Nc	1/1071	1/19518	0.054872	0.305923	0.274834	NOC2L	1
GO:003069	CC	GO:003069	Noc2p-Nc	1/1071	1/19518	0.054872	0.305923	0.274834	NOC2L	1
GO:003147	CC	GO:003147	myosin V c	1/1071	1/19518	0.054872	0.305923	0.274834	DYNLL2	1
GO:003159	CC	GO:003159	nuclear prc	1/1071	1/19518	0.054872	0.305923	0.274834	PSMC5	1
GO:003214	CC	GO:003214	4-aminobu	1/1071	1/19518	0.054872	0.305923	0.274834	ABAT	1
GO:003477	CC	GO:003477	recycling e	1/1071	1/19518	0.054872	0.305923	0.274834	PDLIM4	1
GO:004420	CC	GO:004420	translation	1/1071	1/19518	0.054872	0.305923	0.274834	EIF2S1	1
GO:005502	CC	GO:005502	cortical mi	1/1071	1/19518	0.054872	0.305923	0.274834	NUMA1	1
GO:006147	CC	GO:006147	phagolyso:	1/1071	1/19518	0.054872	0.305923	0.274834	LAMP1	1
GO:007027	CC	GO:007027	RES compl	1/1071	1/19518	0.054872	0.305923	0.274834	BUD13	1
GO:007061	CC	GO:007061	Grb2-Sos	1/1071	1/19518	0.054872	0.305923	0.274834	KHDRBS1	1
GO:009072	CC	GO:009072	peripheral	1/1071	1/19518	0.054872	0.305923	0.274834	CYFIP1	1
GO:009747	CC	GO:009747	mossy fibe	1/1071	1/19518	0.054872	0.305923	0.274834	GRIK2	1
GO:009853	CC	GO:009853	cytoplasmi	1/1071	1/19518	0.054872	0.305923	0.274834	AP4B1	1
GO:009854	CC	GO:009854	cytoplasmi	1/1071	1/19518	0.054872	0.305923	0.274834	AP4B1	1
GO:009859	CC	GO:009859	mucin grar	1/1071	1/19518	0.054872	0.305923	0.274834	VAMP8	1
GO:190572	CC	GO:190572	cytoplasmi	1/1071	1/19518	0.054872	0.305923	0.274834	NUMA1	1
GO:199070	CC	GO:199070	MAD1 com	1/1071	1/19518	0.054872	0.305923	0.274834	MAD1L1	1
GO:003001	CC	GO:003001	myofibril	19/1071	233/19518	0.055258	0.306071	0.274968	PALLD/CAI	19
GO:003194	CC	GO:003194	filamentou	4/1071	27/19518	0.057896	0.318613	0.286235	SPECC1L/D	4
GO:003557	CC	GO:003557	azurophil g	9/1071	91/19518	0.061725	0.337509	0.30321	CYB5R3/AI	9
GO:003067	CC	GO:003067	phagocytic	8/1071	78/19518	0.063662	0.345885	0.310735	VAMP8/LA	8
GO:000022	CC	GO:000022	nuclear chr	18/1071	223/19518	0.065741	0.353574	0.317643	NIFK/PINX	18
GO:009879	CC	GO:009879	mitochond	23/1071	300/19518	0.066636	0.353574	0.317643	MRPL37/S/	23
GO:000586	CC	GO:000586	dynactin co	2/1071	8/19518	0.067565	0.353574	0.317643	ACTR1B/D	2
GO:003310	CC	GO:003310	cis-Golgi n	2/1071	8/19518	0.067565	0.353574	0.317643	BOK/HLA-	2
GO:003409	CC	GO:003409	VCP-NPL4	2/1071	8/19518	0.067565	0.353574	0.317643	FAF1/VCP	2
GO:009883	CC	GO:009883	presynapti	2/1071	8/19518	0.067565	0.353574	0.317643	SNAP91/D	2
GO:000577	CC	GO:000577	vacuolar m	33/1071	460/19518	0.070425	0.36476	0.327692	VAMP8/AB	33
GO:007268	CC	GO:007268	mitotic spi	15/1071	180/19518	0.070557	0.36476	0.327692	CNTRL/DC	15
GO:009855	CC	GO:009855	lumenal sic	4/1071	29/19518	0.071969	0.369814	0.332233	HLA-B/HL/	4
GO:007099	CC	GO:007099	translation	3/1071	18/19518	0.072659	0.371126	0.333411	EIF3D/EIF2	3
GO:004329	CC	GO:004329	contractile	19/1071	243/19518	0.076789	0.389887	0.350266	PALLD/CAI	19
GO:003001	CC	GO:003001	sarcomere	17/1071	213/19518	0.078195	0.393468	0.353482	PALLD/CAI	17
GO:010103	CC	GO:010103	chaperone	5/1071	42/19518	0.078417	0.393468	0.353482	RUVBL2/PI	5
GO:000573	CC	GO:000573	sno(s)RNA	4/1071	30/19518	0.079599	0.397062	0.356712	FBL/NHP2/	4
GO:003068	CC	GO:003068	preriboson	3/1071	19/19518	0.0829	0.399278	0.358702	RRS1/EIF6/	3
GO:003196	CC	GO:003196	nuclear me	23/1071	308/19518	0.083346	0.399278	0.358702	SCRN1/PH	23
GO:000854	CC	GO:000854	proteasom	2/1071	9/19518	0.083788	0.399278	0.358702	PSMC5/PSI	2
GO:001660	CC	GO:001660	flotillin con	2/1071	9/19518	0.083788	0.399278	0.358702	SORBS1/C(	2
GO:003108	CC	GO:003108	BLOC-1 co	2/1071	9/19518	0.083788	0.399278	0.358702	BCAS4/BL(	2
GO:004429	CC	GO:004429	dendritic g	2/1071	9/19518	0.083788	0.399278	0.358702	COBL/CYFI	2
GO:004517	CC	GO:004517	apical corte	2/1071	9/19518	0.083788	0.399278	0.358702	MYO5B/PR	2
GO:009883	CC	GO:009883	presynapti	2/1071	9/19518	0.083788	0.399278	0.358702	SNAP91/D	2
GO:004533	CC	GO:004533	phagocytic	12/1071	141/19518	0.08713	0.40465	0.363528	VAMP8/LA	12
GO:009898	CC	GO:009898	GABA-ergi	8/1071	84/19518	0.089491	0.40465	0.363528	PHB2/KCN	8
GO:199020	CC	GO:199020	oxidoreduc	11/1071	128/19518	0.093473	0.40465	0.363528	GPD1L/CYI	11
GO:003557	CC	GO:003557	azurophil g	6/1071	58/19518	0.097281	0.40465	0.363528	VAMP8/LA	6
GO:004429	CC	GO:004429	cell-cell co	7/1071	72/19518	0.099301	0.40465	0.363528	ANK3/TIAN	7
GO:000013	CC	GO:000013	Golgi trans	2/1071	10/19518	0.101038	0.40465	0.363528	ST6GAL1/C	2
GO:000017	CC	GO:000017	ribonuclea:	2/1071	10/19518	0.101038	0.40465	0.363528	POP7/POP:	2
GO:000034	CC	GO:000034	transcriptic	2/1071	10/19518	0.101038	0.40465	0.363528	CHTOP/TH	2
GO:000830	CC	GO:000830	caspase co	2/1071	10/19518	0.101038	0.40465	0.363528	PIGT/PIGK	2
GO:003068	CC	GO:003068	multimeric	2/1071	10/19518	0.101038	0.40465	0.363528	POP7/POP:	2
GO:007254	CC	GO:007254	EMC comp	2/1071	10/19518	0.101038	0.40465	0.363528	EMC10/EM	2
GO:010101	CC	GO:010101	nucleolar e	2/1071	10/19518	0.101038	0.40465	0.363528	EXOSC10/I	2
GO:012010	CC	GO:012010	centriolar s	2/1071	10/19518	0.101038	0.40465	0.363528	CNTRL/DC	2
GO:004258	CC	GO:004258	specific gra	13/1071	160/19518	0.101751	0.40465	0.363528	VAMP8/NI	13
GO:004517	CC	GO:004517	basal part	21/1071	285/19518	0.104659	0.40465	0.363528	MSN/ANK:	21
GO:000568	CC	GO:000568	U1 snRNP	4/1071	33/19518	0.104746	0.40465	0.363528	SNRPD1/SI	4
GO:003159	CC	GO:003159	neuromusc	7/1071	73/19518	0.104938	0.40465	0.363528	SYNGR3/A	7
GO:001251	CC	GO:001251	trans-Golg	3/1071	21/19518	0.105083	0.40465	0.363528	AP4B1/AP1	3
GO:003242	CC	GO:003242	stereociliur	3/1071	21/19518	0.105083	0.40465	0.363528	EPS8L2/M(	3
GO:000216	CC	GO:000216	3-methylc	1/1071	2/19518	0.106737	0.40465	0.363528	MCCC2	1
GO:000560	CC	GO:000560	classical-c	1/1071	2/19518	0.106737	0.40465	0.363528	CFB	1
GO:000596	CC	GO:000596	glycine cle	1/1071	2/19518	0.106737	0.40465	0.363528	GLDC	1
GO:001037	CC	GO:001037	perinucleol	1/1071	2/19518	0.106737	0.40465	0.363528	TINF2	1
GO:001717	CC	GO:001717	glucosidas	1/1071	2/19518	0.106737	0.40465	0.363528	PRKCSH	1
GO:003108	CC	GO:003108	BLOC-3 co	1/1071	2/19518	0.106737	0.40465	0.363528	HPS4	1
GO:003141	CC	GO:003141	NatB comp	1/1071	2/19518	0.106737	0.40465	0.363528	NAA25	1
GO:003151	CC	GO:003151	SUMO acti	1/1071	2/19518	0.106737	0.40465	0.363528	SAE1	1
GO:003239	CC	GO:003239	MHC class	1/1071	2/19518	0.106737	0.40465	0.363528	HLA-F	1
GO:003468	CC	GO:003468	integrin alp	1/1071	2/19518	0.106737	0.40465	0.363528	ITGA10	1
GO:003563	CC	GO:003563	mitochond	1/1071	2/19518	0.106737	0.40465	0.363528	PHB2	1
GO:003611	CC	GO:003611	hyaluranor	1/1071	2/19518	0.106737	0.40465	0.363528	HYAL1	1
GO:004361	CC	GO:004361	multi-eIF c	1/1071	2/19518	0.106737	0.40465	0.363528	EIF2S1	1
GO:009061	CC	GO:009061	meiotic spi	1/1071	2/19518	0.106737	0.40465	0.363528	CNTRL	1
GO:009705	CC	GO:009705	TRAF2-GS	1/1071	2/19518	0.106737	0.40465	0.363528	GSTP1	1
GO:009705	CC	GO:009705	CRLF-CLCI	1/1071	2/19518	0.106737	0.40465	0.363528	CLCF1	1
GO:009705	CC	GO:009705	CNTFR-CL	1/1071	2/19518	0.106737	0.40465	0.363528	CLCF1	1
GO:009867	CC	GO:009867	extrinsic co	1/1071	2/19518	0.106737	0.40465	0.363528	KIF1A	1
GO:009892	CC	GO:009892	extrinsic co	1/1071	2/19518	0.106737	0.40465	0.363528	KIF1A	1
GO:190256	CC	GO:190256	GMP reduc	1/1071	2/19518	0.106737	0.40465	0.363528	GMPR	1
GO:190271	CC	GO:190271	G protein-	1/1071	2/19518	0.106737	0.40465	0.363528	GABBR1	1
GO:190520	CC	GO:190520	methylcrot	1/1071	2/19518	0.106737	0.40465	0.363528	MCCC2	1
GO:199066	CC	GO:199066	AnxA2-p1:	1/1071	2/19518	0.106737	0.40465	0.363528	ANXA2	1
GO:199066	CC	GO:199066	PCSK9-LDI	1/1071	2/19518	0.106737	0.40465	0.363528	LDLR	1
GO:199066	CC	GO:199066	PCSK9-An:	1/1071	2/19518	0.106737	0.40465	0.363528	ANXA2	1
GO:199072	CC	GO:199072	mitotic spi	1/1071	2/19518	0.106737	0.40465	0.363528	MAD1L1	1
GO:199091	CC	GO:199091	ooplasm	1/1071	2/19518	0.106737	0.40465	0.363528	FAF1	1
GO:000588	CC	GO:000588	cytoplasmi	9/1071	102/19518	0.107445	0.405534	0.364323	ENKUR/FH	9
GO:000576	CC	GO:000576	lysosomal	29/1071	415/19518	0.108593	0.406273	0.364986	VAMP8/AB	29
GO:009885	CC	GO:009885	lytic vacuo	29/1071	415/19518	0.108593	0.406273	0.364986	VAMP8/AB	29
GO:001989	CC	GO:001989	extrinsic co	12/1071	147/19518	0.109788	0.408947	0.367388	ESYT3/S10	12
GO:000581	CC	GO:000581	lipid drople	9/1071	103/19518	0.112351	0.409838	0.368189	PRPF19/CY	9
GO:001989	CC	GO:001989	extrinsic co	21/1071	288/19518	0.113134	0.409838	0.368189	ESYT3/SYT	21
GO:004319	CC	GO:004319	terminal bo	5/1071	47/19518	0.113684	0.409838	0.368189	OXT/KCNC	5
GO:199075	CC	GO:199075	microtubul	4/1071	34/19518	0.113841	0.409838	0.368189	DCTN1/CL	4
GO:000574	CC	GO:000574	mitochond	3/1071	22/19518	0.116954	0.409838	0.368189	SAMM50/N	3
GO:003125	CC	GO:003125	lamellipodi	3/1071	22/19518	0.116954	0.409838	0.368189	SYNE2/CSI	3
GO:000023	CC	GO:000023	astral micro	2/1071	11/19518	0.119156	0.409838	0.368189	PAFAH1B1	2
GO:000217	CC	GO:000217	palmitoyltr	2/1071	11/19518	0.119156	0.409838	0.368189	SPTLC2/ZD	2
GO:000561	CC	GO:000561	interstitial r	2/1071	11/19518	0.119156	0.409838	0.368189	EGFLAM/V	2
GO:000581	CC	GO:000581	aster	2/1071	11/19518	0.119156	0.409838	0.368189	PAFAH1B1	2
GO:003499	CC	GO:003499	microtubul	2/1071	11/19518	0.119156	0.409838	0.368189	SUN2/SYN	2
GO:003499	CC	GO:003499	meiotic nu	2/1071	11/19518	0.119156	0.409838	0.368189	SUN2/SYN	2
GO:003563	CC	GO:003563	CD40 rece	2/1071	11/19518	0.119156	0.409838	0.368189	TRAF6/IKB	2
GO:006009	CC	GO:006009	kinocilium	2/1071	11/19518	0.119156	0.409838	0.368189	RSPH9/ELN	2
GO:007116	CC	GO:007116	CMG comp	2/1071	11/19518	0.119156	0.409838	0.368189	GINS2/MC	2
GO:009773	CC	GO:009773	9+2 non-n	2/1071	11/19518	0.119156	0.409838	0.368189	RSPH9/ELN	2
GO:009898	CC	GO:009898	cholinergic	2/1071	11/19518	0.119156	0.409838	0.368189	PLD1/DLG/	2
GO:010608	CC	GO:010608	nuclear me	2/1071	11/19518	0.119156	0.409838	0.368189	SUN2/SYN	2
GO:010609	CC	GO:010609	nuclear me	2/1071	11/19518	0.119156	0.409838	0.368189	SUN2/SYN	2
GO:003068	CC	GO:003068	preriboson	7/1071	76/19518	0.122854	0.419611	0.376969	FBL/RRS1/	7
GO:000581	CC	GO:000581	spindle	29/1071	421/19518	0.123146	0.419611	0.376969	PINX1/PRP	29
GO:003558	CC	GO:003558	specific gra	6/1071	62/19518	0.123608	0.419611	0.376969	NIT2/HPSE	6
GO:000579	CC	GO:000579	Golgi lume	9/1071	106/19518	0.127796	0.419611	0.376969	MUC1/SOI	9
GO:000576	CC	GO:000576	early endo:	29/1071	423/19518	0.128253	0.419611	0.376969	VAMP8/CI	29
GO:009879	CC	GO:009879	outer mito	3/1071	23/19518	0.129297	0.419611	0.376969	SAMM50/N	3
GO:003606	CC	GO:003606	ciliary basa	13/1071	167/19518	0.129331	0.419611	0.376969	CSNK1D/D	13
GO:000568	CC	GO:000568	U2-type sp	8/1071	92/19518	0.132027	0.419611	0.376969	PRPF19/SN	8
GO:009857	CC	GO:009857	lumenal sic	4/1071	36/19518	0.133019	0.419611	0.376969	HLA-B/HL/	4
GO:000578	CC	GO:000578	endoplasm	22/1071	312/19518	0.137202	0.419611	0.376969	SERPINA1/	22
GO:009897	CC	GO:009897	glutamater	28/1071	410/19518	0.137383	0.419611	0.376969	TNIK/JAK2	28
GO:001470	CC	GO:001470	intercalate	5/1071	50/19518	0.137909	0.419611	0.376969	ANK3/SCN	5
GO:000012	CC	GO:000012	histone ace	8/1071	93/19518	0.137968	0.419611	0.376969	RUVBL2/K/	8
GO:000017	CC	GO:000017	nuclear exc	2/1071	12/19518	0.137993	0.419611	0.376969	EXOSC10/I	2
GO:000078	CC	GO:000078	telomere c	2/1071	12/19518	0.137993	0.419611	0.376969	TINF2/CTC	2
GO:000078	CC	GO:000078	nuclear tel	2/1071	12/19518	0.137993	0.419611	0.376969	TINF2/CTC	2
GO:000584	CC	GO:000584	mRNA cap	2/1071	12/19518	0.137993	0.419611	0.376969	CYFIP1/EIF	2
GO:003012	CC	GO:003012	AP-1 adap	2/1071	12/19518	0.137993	0.419611	0.376969	AP1M1/AP	2
GO:003120	CC	GO:003120	SCAR com	2/1071	12/19518	0.137993	0.419611	0.376969	WASF3/CY	2
GO:003126	CC	GO:003126	DNA replic	2/1071	12/19518	0.137993	0.419611	0.376969	GINS2/MC	2
GO:004475	CC	GO:004475	autolysoso	2/1071	12/19518	0.137993	0.419611	0.376969	LAMP1/NC	2
GO:014026	CC	GO:014026	endoplasm	2/1071	12/19518	0.137993	0.419611	0.376969	ESYT3/ESY	2
GO:199007	CC	GO:199007	TRAPPII pr	2/1071	12/19518	0.137993	0.419611	0.376969	TRAPPC2L/	2
GO:199007	CC	GO:199007	TRAPPIII p	2/1071	12/19518	0.137993	0.419611	0.376969	TRAPPC2L/	2
GO:003537	CC	GO:003537	microtubul	3/1071	24/19518	0.142075	0.419611	0.376969	DCTN1/CL	3
GO:009868	CC	GO:009868	Schaffer co	8/1071	94/19518	0.144041	0.419611	0.376969	PAFAH1B1	8
GO:000992	CC	GO:000992	basal plasr	19/1071	266/19518	0.145392	0.419611	0.376969	MSN/ANK:	19
GO:001632	CC	GO:001632	basolatera	17/1071	234/19518	0.145671	0.419611	0.376969	MSN/ANK:	17
GO:009738	CC	GO:009738	glial cell pr	4/1071	38/19518	0.153411	0.419611	0.376969	EIF2S1/GPI	4
GO:003031	CC	GO:003031	T-tubule	5/1071	52/19518	0.155213	0.419611	0.376969	CAPN3/AN	5
GO:000575	CC	GO:000575	mitochond	3/1071	25/19518	0.155251	0.419611	0.376969	COX6B1/C	3
GO:003013	CC	GO:003013	clathrin ad:	3/1071	25/19518	0.155251	0.419611	0.376969	AP4B1/AP1	3
GO:000011	CC	GO:000011	nucleotide-	1/1071	3/19518	0.155757	0.419611	0.376969	XPA	1
GO:000012	CC	GO:000012	transcriptic	1/1071	3/19518	0.155757	0.419611	0.376969	BDP1	1
GO:000043	CC	GO:000043	core TFIIH	1/1071	3/19518	0.155757	0.419611	0.376969	GTF2H4	1
GO:000589	CC	GO:000589	interleukin-	1/1071	3/19518	0.155757	0.419611	0.376969	IL6R	1
GO:000824	CC	GO:000824	1-alkyl-2-	1/1071	3/19518	0.155757	0.419611	0.376969	PAFAH1B1	1
GO:000933	CC	GO:000933	glycerol-3-	1/1071	3/19518	0.155757	0.419611	0.376969	GPD1L	1
GO:003068	CC	GO:003068	Noc comp	1/1071	3/19518	0.155757	0.419611	0.376969	NOC2L	1
GO:003095	CC	GO:003095	glutamyl-t	1/1071	3/19518	0.155757	0.419611	0.376969	GATC	1
GO:003098	CC	GO:003098	cortical mi	1/1071	3/19518	0.155757	0.419611	0.376969	NUMA1	1
GO:003139	CC	GO:003139	Elg1 RFC-I	1/1071	3/19518	0.155757	0.419611	0.376969	RFC4	1
GO:003190	CC	GO:003190	early endo:	1/1071	3/19518	0.155757	0.419611	0.376969	PDLIM4	1
GO:003229	CC	GO:003229	ribonuclea:	1/1071	3/19518	0.155757	0.419611	0.376969	RNASEH2A	1
GO:003280	CC	GO:003280	DNA ligase	1/1071	3/19518	0.155757	0.419611	0.376969	XRCC4	1
GO:003293	CC	GO:003293	SREBP-SC/	1/1071	3/19518	0.155757	0.419611	0.376969	INSIG1	1
GO:003318	CC	GO:003318	dolichol-pl	1/1071	3/19518	0.155757	0.419611	0.376969	DPM3	1
GO:003467	CC	GO:003467	inhibin-bet	1/1071	3/19518	0.155757	0.419611	0.376969	TGFBR3	1
GO:003803	CC	GO:003803	G protein-	1/1071	3/19518	0.155757	0.419611	0.376969	GABBR1	1
GO:004202	CC	GO:004202	interleukin-	1/1071	3/19518	0.155757	0.419611	0.376969	JAK2	1
GO:007011	CC	GO:007011	ciliary neur	1/1071	3/19518	0.155757	0.419611	0.376969	IL6R	1
GO:009712	CC	GO:009712	cyclin D2-(	1/1071	3/19518	0.155757	0.419611	0.376969	CDKN2D	1
GO:009745	CC	GO:009745	glial limitin	1/1071	3/19518	0.155757	0.419611	0.376969	EIF2S1	1
GO:009751	CC	GO:009751	myosin II fi	1/1071	3/19518	0.155757	0.419611	0.376969	MYH10	1
GO:009884	CC	GO:009884	postsynapt	1/1071	3/19518	0.155757	0.419611	0.376969	RAB5A	1
GO:010100	CC	GO:010100	cytolytic gr	1/1071	3/19518	0.155757	0.419611	0.376969	LAMP1	1
GO:012000	CC	GO:012000	apical plas	1/1071	3/19518	0.155757	0.419611	0.376969	UPK1B	1
GO:012020	CC	GO:012020	photorece	1/1071	3/19518	0.155757	0.419611	0.376969	SPATA7	1
GO:015004	CC	GO:015004	cerebellar	1/1071	3/19518	0.155757	0.419611	0.376969	YWHAH	1
GO:190395	CC	GO:190395	nitric-oxide	1/1071	3/19518	0.155757	0.419611	0.376969	CYB5R3	1
GO:199011	CC	GO:199011	RQC comp	1/1071	3/19518	0.155757	0.419611	0.376969	TCF25	1
GO:199045	CC	GO:199045	Parkin-FBX	1/1071	3/19518	0.155757	0.419611	0.376969	CUL1	1
GO:199087	CC	GO:199087	CST compl	1/1071	3/19518	0.155757	0.419611	0.376969	CTC1	1
GO:000081	CC	GO:000081	Swr1 comp	2/1071	13/19518	0.157416	0.419611	0.376969	RUVBL2/K/	2
GO:000097	CC	GO:000097	Prp19 com	2/1071	13/19518	0.157416	0.419611	0.376969	PRPF19/TS	2
GO:000193	CC	GO:000193	uropod	2/1071	13/19518	0.157416	0.419611	0.376969	MSN/EZR	2
GO:000567	CC	GO:000567	transcriptic	2/1071	13/19518	0.157416	0.419611	0.376969	GTF2H4/M	2
GO:003451	CC	GO:003451	RNA cap b	2/1071	13/19518	0.157416	0.419611	0.376969	CYFIP1/EIF	2
GO:003603	CC	GO:003603	MKS comp	2/1071	13/19518	0.157416	0.419611	0.376969	TCTN1/B9I	2
GO:004429	CC	GO:004429	dendrite te	2/1071	13/19518	0.157416	0.419611	0.376969	COBL/CYFI	2
GO:009909	CC	GO:009909	postsynapt	2/1071	13/19518	0.157416	0.419611	0.376969	TNIK/DNM	2
GO:014044	CC	GO:014044	chromosor	2/1071	13/19518	0.157416	0.419611	0.376969	TINF2/CTC	2
GO:009880	CC	GO:009880	inner mitoc	12/1071	158/19518	0.159362	0.423475	0.38044	SAMM50/N	12
GO:003066	CC	GO:003066	secretory g	22/1071	319/19518	0.16037	0.42483	0.381658	VAMP8/LA	22
GO:000077	CC	GO:000077	kinetochor	12/1071	159/19518	0.16436	0.434054	0.389944	PINX1/DCT	12
GO:003028	CC	GO:003028	dynein con	5/1071	54/19518	0.173358	0.456402	0.410021	DCTN1/DY	5
GO:003067	CC	GO:003067	ribonuclea:	2/1071	14/19518	0.177304	0.461099	0.414241	POP7/POP:	2
GO:003125	CC	GO:003125	cell trailing	2/1071	14/19518	0.177304	0.461099	0.414241	MSN/EZR	2
GO:003506	CC	GO:003506	brahma co	2/1071	14/19518	0.177304	0.461099	0.414241	SMARCD2/	2
GO:004360	CC	GO:004360	nuclear rep	2/1071	14/19518	0.177304	0.461099	0.414241	POLD4/PO	2
GO:003013	CC	GO:003013	transport v	28/1071	425/19518	0.182284	0.467162	0.419687	PPT1/CRISI	28
GO:190472	CC	GO:190472	tertiary gra	5/1071	55/19518	0.182722	0.467162	0.419687	NIT2/CDA/	5
GO:000558	CC	GO:000558	collagen tr	7/1071	85/19518	0.184947	0.467162	0.419687	COL6A2/C	7
GO:004521	CC	GO:004521	postsynapt	19/1071	277/19518	0.187527	0.467162	0.419687	PRRT1/ANI	19
GO:000579	CC	GO:000579	endoplasm	10/1071	132/19518	0.188876	0.467162	0.419687	SERPINA1/	10
GO:003066	CC	GO:003066	endocytic	14/1071	196/19518	0.189842	0.467162	0.419687	VAMP8/AD	14
GO:000031	CC	GO:000031	organellar	5/1071	56/19518	0.192268	0.467162	0.419687	MRPL37/M	5
GO:000576	CC	GO:000576	mitochond	5/1071	56/19518	0.192268	0.467162	0.419687	MRPL37/M	5
GO:003013	CC	GO:003013	coated ves	21/1071	312/19518	0.195424	0.467162	0.419687	SERPINA1/	21
GO:003645	CC	GO:003645	ESCRT con	3/1071	28/19518	0.1968	0.467162	0.419687	VPS37B/VI	3
GO:004527	CC	GO:004527	respiratory	3/1071	28/19518	0.1968	0.467162	0.419687	COX6B1/C	3
GO:009899	CC	GO:009899	neuronal d	3/1071	28/19518	0.1968	0.467162	0.419687	ADRB2/KIF	3
GO:005503	CC	GO:005503	recycling e	8/1071	102/19518	0.197047	0.467162	0.419687	VAMP8/PA	8
GO:000017	CC	GO:000017	cytoplasmi	2/1071	15/19518	0.197549	0.467162	0.419687	EXOSC10/I	2
GO:000585	CC	GO:000585	eukaryotic	2/1071	15/19518	0.197549	0.467162	0.419687	EIF3D/EIF3	2
GO:001658	CC	GO:001658	RSC-type	2/1071	15/19518	0.197549	0.467162	0.419687	SMARCD2/	2
GO:009773	CC	GO:009773	non-motile	12/1071	166/19518	0.201457	0.467162	0.419687	RSPH9/MA	12
GO:000001	CC	GO:000001	phosphopy	1/1071	4/19518	0.20209	0.467162	0.419687	ENO3	1
GO:000081	CC	GO:000081	GINS comp	1/1071	4/19518	0.20209	0.467162	0.419687	GINS2	1
GO:000559	CC	GO:000559	collagen ty	1/1071	4/19518	0.20209	0.467162	0.419687	COL9A3	1
GO:000572	CC	GO:000572	perichroma	1/1071	4/19518	0.20209	0.467162	0.419687	TRIM24	1
GO:000582	CC	GO:000582	actomyosi	1/1071	4/19518	0.20209	0.467162	0.419687	RTKN	1
GO:000585	CC	GO:000585	eukaryotic	1/1071	4/19518	0.20209	0.467162	0.419687	EEF1D	1
GO:000595	CC	GO:000595	DNA-depe	1/1071	4/19518	0.20209	0.467162	0.419687	XRCC4	1
GO:001601	CC	GO:001601	syntrophin	1/1071	4/19518	0.20209	0.467162	0.419687	SNTG2	1
GO:001603	CC	GO:001603	zeta DNA	1/1071	4/19518	0.20209	0.467162	0.419687	POLD2	1
GO:003012	CC	GO:003012	AP-4 adap	1/1071	4/19518	0.20209	0.467162	0.419687	AP4B1	1
GO:003047	CC	GO:003047	actin cap	1/1071	4/19518	0.20209	0.467162	0.419687	GSN	1
GO:003052	CC	GO:003052	granulocyt	1/1071	4/19518	0.20209	0.467162	0.419687	JAK2	1
GO:003090	CC	GO:003090	retromer, c	1/1071	4/19518	0.20209	0.467162	0.419687	VPS26A	1
GO:003159	CC	GO:003159	cytosolic p	1/1071	4/19518	0.20209	0.467162	0.419687	PSMC5	1
GO:003202	CC	GO:003202	NELF comp	1/1071	4/19518	0.20209	0.467162	0.419687	NELFCD	1
GO:003277	CC	GO:003277	Piccolo Nu	1/1071	4/19518	0.20209	0.467162	0.419687	KAT5	1
GO:004354	CC	GO:004354	UDP-N-ac	1/1071	4/19518	0.20209	0.467162	0.419687	EXT2	1
GO:007253	CC	GO:007253	interleukin-	1/1071	4/19518	0.20209	0.467162	0.419687	JAK2	1
GO:007258	CC	GO:007258	box H/ACA	1/1071	4/19518	0.20209	0.467162	0.419687	NHP2	1
GO:009745	CC	GO:009745	GAIT comp	1/1071	4/19518	0.20209	0.467162	0.419687	SYNCRIP	1
GO:009754	CC	GO:009754	ciliary inve	1/1071	4/19518	0.20209	0.467162	0.419687	INVS	1
GO:009855	CC	GO:009855	cytoplasmi	1/1071	4/19518	0.20209	0.467162	0.419687	RAB5A	1
GO:009889	CC	GO:009889	extrinsic co	1/1071	4/19518	0.20209	0.467162	0.419687	SNAP91	1
GO:199078	CC	GO:199078	GLI-SUFU	1/1071	4/19518	0.20209	0.467162	0.419687	SUFU	1
GO:007101	CC	GO:007101	catalytic st	7/1071	88/19518	0.208085	0.479719	0.430969	PRPF19/SN	7
GO:000177	CC	GO:000177	immunolog	4/1071	43/19518	0.208841	0.480166	0.43137	VAV3/EZR/	4
GO:000590	CC	GO:000590	clathrin-co	6/1071	73/19518	0.210951	0.482993	0.43391	LDLR/SH3E	6
GO:000568	CC	GO:000568	U2 snRNP	3/1071	29/19518	0.211203	0.482993	0.43391	SNRPD1/SI	3
GO:003066	CC	GO:003066	Golgi-assc	5/1071	58/19518	0.211862	0.483204	0.434099	ZDHHC13/	5
GO:003066	CC	GO:003066	clathrin-co	10/1071	136/19518	0.213677	0.486043	0.43665	VAMP8/AD	10
GO:000589	CC	GO:000589	acetylcholi	2/1071	16/19518	0.21805	0.492055	0.442051	CHRNA1/C	2
GO:003089	CC	GO:003089	replisome	2/1071	16/19518	0.21805	0.492055	0.442051	POLD4/PO	2
GO:009909	CC	GO:009909	postsynapt	2/1071	16/19518	0.21805	0.492055	0.442051	TNIK/DNM	2
GO:001593	CC	GO:001593	small ribos	6/1071	74/19518	0.219793	0.494664	0.444395	MRPS9/MI	6
GO:003066	CC	GO:003066	coated ves	14/1071	202/19518	0.220426	0.494664	0.444395	VAMP8/AD	14

GO:004224	BP	GO:004224	tissue rege	7/902	73/18614	0.063	0.378526	0.339748	DUSP10/F/	7
GO:007126	BP	GO:007126	cellular res	7/902	73/18614	0.063	0.378526	0.339748	IRF1/ENG/	7
GO:000646	BP	GO:000646	negative re	16/902	217/18614	0.063012	0.378526	0.339748	TRIB3/ZNF	16
GO:004663	BP	GO:004663	alpha-beta	10/902	119/18614	0.063308	0.379825	0.340914	IRF1/BCL3/	10
GO:001082	BP	GO:001082	regulation	12/902	151/18614	0.063342	0.379825	0.340914	PMAIP1/BN	12
GO:000661	BP	GO:000661	protein tar	11/902	135/18614	0.063623	0.380829	0.341815	SEC61G/ZI	11
GO:004247	BP	GO:004247	odontoger	11/902	135/18614	0.063623	0.380829	0.341815	GATA6/TG	11
GO:004846	BP	GO:004846	cell matura	14/902	184/18614	0.063865	0.381191	0.34214	DEFB1/G6I	14
GO:007099	BP	GO:007099	neuron dea	25/902	373/18614	0.06388	0.381191	0.34214	CCND1/TG	25
GO:005117	BP	GO:005117	import intc	13/902	168/18614	0.065029	0.381191	0.34214	SUMO1/B(	13
GO:000834	BP	GO:000834	glial cell m	6/902	59/18614	0.065211	0.381191	0.34214	TGFB2/SR(	6
GO:005043	BP	GO:005043	catecholan	6/902	59/18614	0.065211	0.381191	0.34214	SDHD/SYT	6
GO:019873	BP	GO:019873	cell-cell sig	30/902	463/18614	0.065341	0.381191	0.34214	GREM1/C(	30
GO:000662	BP	GO:000662	protein tar	5/902	45/18614	0.065352	0.381191	0.34214	LAMP2/M(	5
GO:001401	BP	GO:001401	negative re	5/902	45/18614	0.065352	0.381191	0.34214	DUSP10/S(	5
GO:004848	BP	GO:004848	autonomic	5/902	45/18614	0.065352	0.381191	0.34214	SOX11/SO:	5
GO:004870	BP	GO:004870	embryonic	5/902	45/18614	0.065352	0.381191	0.34214	IRX5/SLC3!	5
GO:006220	BP	GO:006220	positive re	5/902	45/18614	0.065352	0.381191	0.34214	NR1H3/OA	5
GO:004400	BP	GO:004400	movement	14/902	185/18614	0.066172	0.381191	0.34214	ITCH/TRIM	14
GO:007177	BP	GO:007177	response t	10/902	120/18614	0.06622	0.381191	0.34214	ZFP36L1/G	10
GO:000110	BP	GO:000110	response t	11/902	136/18614	0.066349	0.381191	0.34214	SESN3/CPI	11
GO:004336	BP	GO:004336	CD4-positi	8/902	89/18614	0.066587	0.381191	0.34214	BCL3/FOXI	8
GO:005507	BP	GO:005507	iron ion ho	8/902	89/18614	0.066587	0.381191	0.34214	STEAP1/IS(	8
GO:002153	BP	GO:002153	telencepha	19/902	270/18614	0.066805	0.381191	0.34214	SRGAP2/W	19
GO:000206	BP	GO:000206	glandular e	7/902	74/18614	0.066819	0.381191	0.34214	GATA6/RH	7
GO:000758	BP	GO:000758	respiratory	7/902	74/18614	0.066819	0.381191	0.34214	MAFB/TRA	7
GO:000833	BP	GO:000833	endosome	7/902	74/18614	0.066819	0.381191	0.34214	CHMP5/PL	7
GO:005114	BP	GO:005114	smooth mu	7/902	74/18614	0.066819	0.381191	0.34214	GATA6/EN	7
GO:000683	BP	GO:000683	neurotrans	15/902	202/18614	0.06688	0.381191	0.34214	CADPS2/BI	15
GO:190165	BP	GO:190165	cellular res	25/902	375/18614	0.067071	0.381191	0.34214	TRIB3/ZFP:	25
GO:000151	BP	GO:000151	prostaglan	4/902	32/18614	0.067204	0.381191	0.34214	PTGDS/PT(	4
GO:000608	BP	GO:000608	acetyl-Co/	4/902	32/18614	0.067204	0.381191	0.34214	PDK2/DLA	4
GO:000634	BP	GO:000634	DNA meth	4/902	32/18614	0.067204	0.381191	0.34214	MBD2/SAN	4
GO:000662	BP	GO:000662	protein tar	4/902	32/18614	0.067204	0.381191	0.34214	LAMP2/SO	4
GO:003106	BP	GO:003106	hair follicle	4/902	32/18614	0.067204	0.381191	0.34214	TGFB2/DL)	4
GO:003625	BP	GO:003625	multivesicu	4/902	32/18614	0.067204	0.381191	0.34214	CHMP5/ST	4
GO:004645	BP	GO:004645	prostanoid	4/902	32/18614	0.067204	0.381191	0.34214	PTGDS/PT(	4
GO:004682	BP	GO:004682	regulation	4/902	32/18614	0.067204	0.381191	0.34214	SIRT6/UHN	4
GO:007135	BP	GO:007135	cellular res	4/902	32/18614	0.067204	0.381191	0.34214	HSPA5/PTI	4
GO:009886	BP	GO:009886	bone grow	4/902	32/18614	0.067204	0.381191	0.34214	RARB/TSKI	4
GO:000662	BP	GO:000662	protein ret	2/902	9/18614	0.067333	0.381191	0.34214	KDELR3/KI	2
GO:000665	BP	GO:000665	diacylglyce	2/902	9/18614	0.067333	0.381191	0.34214	PLA2G15/	2
GO:000915	BP	GO:000915	deoxyribor	2/902	9/18614	0.067333	0.381191	0.34214	ADK/TYMS	2
GO:000923	BP	GO:000923	cobalamin	2/902	9/18614	0.067333	0.381191	0.34214	AMN/ABCI	2
GO:001061	BP	GO:001061	negative re	2/902	9/18614	0.067333	0.381191	0.34214	FOXO1/SM	2
GO:001069	BP	GO:001069	regulation	2/902	9/18614	0.067333	0.381191	0.34214	TGFB2/SM.	2
GO:001487	BP	GO:001487	response t	2/902	9/18614	0.067333	0.381191	0.34214	PIK3CA/SC	2
GO:001588	BP	GO:001588	cobalamin	2/902	9/18614	0.067333	0.381191	0.34214	AMN/ABCI	2
GO:001609	BP	GO:001609	polyprenol	2/902	9/18614	0.067333	0.381191	0.34214	DPAGT1/A	2
GO:001937	BP	GO:001937	cyclooxyge	2/902	9/18614	0.067333	0.381191	0.34214	PTGDS/AK	2
GO:003162	BP	GO:003162	regulation	2/902	9/18614	0.067333	0.381191	0.34214	PTGES/EDI	2
GO:003413	BP	GO:003413	regulation	2/902	9/18614	0.067333	0.381191	0.34214	TNFAIP3/T	2
GO:003650	BP	GO:003650	ATF6-med	2/902	9/18614	0.067333	0.381191	0.34214	HSPA5/XBI	2
GO:004299	BP	GO:004299	regulation	2/902	9/18614	0.067333	0.381191	0.34214	ATP2C1/C!	2
GO:004482	BP	GO:004482	positive re	2/902	9/18614	0.067333	0.381191	0.34214	PPIB/TBC1	2
GO:004485	BP	GO:004485	hair cycle p	2/902	9/18614	0.067333	0.381191	0.34214	TGFB2/CTI	2
GO:004614	BP	GO:004614	tetrahydrol	2/902	9/18614	0.067333	0.381191	0.34214	SPR/PCBD:	2
GO:004664	BP	GO:004664	positive re	2/902	9/18614	0.067333	0.381191	0.34214	SOX4/LILR	2
GO:004828	BP	GO:004828	vesicle fusi	2/902	9/18614	0.067333	0.381191	0.34214	STX5/VTI1I	2
GO:004838	BP	GO:004838	negative re	2/902	9/18614	0.067333	0.381191	0.34214	CYP26B1/C	2
GO:004861	BP	GO:004861	embryonic	2/902	9/18614	0.067333	0.381191	0.34214	CTNNB1/S	2
GO:004881	BP	GO:004881	regulation	2/902	9/18614	0.067333	0.381191	0.34214	TGFB2/CTI	2
GO:005158	BP	GO:005158	negative re	2/902	9/18614	0.067333	0.381191	0.34214	SNCA/PNK	2
GO:006071	BP	GO:006071	chorion de	2/902	9/18614	0.067333	0.381191	0.34214	DNAJB6/TI	2
GO:006190	BP	GO:006190	autophagc	2/902	9/18614	0.067333	0.381191	0.34214	PIP4K2A/T(	2
GO:006204	BP	GO:006204	positive re	2/902	9/18614	0.067333	0.381191	0.34214	TGFB2/EN(	2
GO:007083	BP	GO:007083	dehydroas	2/902	9/18614	0.067333	0.381191	0.34214	SLC2A3/SL	2
GO:007128	BP	GO:007128	cellular res	2/902	9/18614	0.067333	0.381191	0.34214	SLC40A1/T	2
GO:007234	BP	GO:007234	rescue of s	2/902	9/18614	0.067333	0.381191	0.34214	ASCC3/LTI	2
GO:190324	BP	GO:190324	negative re	2/902	9/18614	0.067333	0.381191	0.34214	FOXO1/SM	2
GO:200034	BP	GO:200034	positive re	2/902	9/18614	0.067333	0.381191	0.34214	TIRAP/MBI	2
GO:000756	BP	GO:000756	aging	13/902	169/18614	0.067486	0.381734	0.342627	GPX4/CD6	13
GO:007082	BP	GO:007082	heterochro	9/902	105/18614	0.068479	0.387025	0.347376	CBX3/MBD	9
GO:003629	BP	GO:003629	response t	21/902	306/18614	0.069312	0.391132	0.351062	GATA6/ZFI	21
GO:009900	BP	GO:009900	vesicle-me	16/902	220/18614	0.069419	0.391132	0.351062	CADPS2/BI	16
GO:000165	BP	GO:000165	branching	6/902	60/18614	0.069635	0.391132	0.351062	GREM1/LA	6
GO:004562	BP	GO:004562	negative re	6/902	60/18614	0.069635	0.391132	0.351062	IRF1/ID2/P	6
GO:000178	BP	GO:000178	neutrophil	3/902	20/18614	0.069966	0.391132	0.351062	SH2B3/CSI	3
GO:000341	BP	GO:000341	growth pla	3/902	20/18614	0.069966	0.391132	0.351062	RARB/TSKI	3
GO:000648	BP	GO:000648	dolichol-lir	3/902	20/18614	0.069966	0.391132	0.351062	DPAGT1/A	3
GO:000931	BP	GO:000931	amine cata	3/902	20/18614	0.069966	0.391132	0.351062	SLC44A1/F	3
GO:001031	BP	GO:001031	regulation	3/902	20/18614	0.069966	0.391132	0.351062	SNCA/SOI	3
GO:003475	BP	GO:003475	iron ion tra	3/902	20/18614	0.069966	0.391132	0.351062	ISCU/SLC2	3
GO:004479	BP	GO:004479	positive re	3/902	20/18614	0.069966	0.391132	0.351062	PPIB/ZDHI	3
GO:006021	BP	GO:006021	definitive h	3/902	20/18614	0.069966	0.391132	0.351062	HIPK1/HO)	3
GO:190223	BP	GO:190223	negative re	3/902	20/18614	0.069966	0.391132	0.351062	SYVN1/XBI	3
GO:199009	BP	GO:199009	cellular res	5/902	46/18614	0.070537	0.393993	0.353631	HSPA5/RAI	5
GO:004269	BP	GO:004269	ovulation c	7/902	75/18614	0.070774	0.394661	0.35423	TNFAIP6/T	7
GO:007147	BP	GO:007147	cellular res	7/902	75/18614	0.070774	0.394661	0.35423	HSPA5/CC	7
GO:000941	BP	GO:000941	response t	12/902	154/18614	0.071205	0.39673	0.356087	CCND1/MI	12
GO:190189	BP	GO:190189	positive re	9/902	106/18614	0.071786	0.399636	0.358695	MAP4K4/IF	9
GO:001095	BP	GO:001095	positive re	13/902	171/18614	0.072581	0.403725	0.362366	CYFIP2/RP!	13
GO:001501	BP	GO:001501	heparan su	4/902	33/18614	0.073673	0.407054	0.365353	DSE/EXTL2	4
GO:003545	BP	GO:003545	vesicle carg	4/902	33/18614	0.073673	0.407054	0.365353	MIA2/TME	4
GO:005108	BP	GO:005108	chaperone	4/902	33/18614	0.073673	0.407054	0.365353	DNAJB14/I	4
GO:006079	BP	GO:006079	cell fate co	4/902	33/18614	0.073673	0.407054	0.365353	GATA6/FZI	4
GO:009018	BP	GO:009018	regulation	4/902	33/18614	0.073673	0.407054	0.365353	GREM1/ST	4
GO:190118	BP	GO:190118	negative re	4/902	33/18614	0.073673	0.407054	0.365353	DUSP3/PTI	4
GO:190489	BP	GO:190489	negative re	4/902	33/18614	0.073673	0.407054	0.365353	SH2B3/BCI	4
GO:003090	BP	GO:003090	midbrain d	8/902	91/18614	0.073809	0.407054	0.365353	DYNLL1/H!	8
GO:004247	BP	GO:004247	odontoger	8/902	91/18614	0.073809	0.407054	0.365353	GATA6/DL	8
GO:004390	BP	GO:004390	regulation	6/902	61/18614	0.074228	0.407054	0.365353	TRIM8/TRI	6
GO:003434	BP	GO:003434	response t	11/902	139/18614	0.074974	0.407054	0.365353	IRF1/NR1H	11
GO:001810	BP	GO:001810	peptidyl-se	21/902	309/18614	0.075016	0.407054	0.365353	DYRK2/NE	21
GO:003252	BP	GO:003252	response t	9/902	107/18614	0.075191	0.407054	0.365353	GATA6/FZI	9
GO:003430	BP	GO:003430	primary alc	9/902	107/18614	0.075191	0.407054	0.365353	GPD2/CYP	9
GO:001820	BP	GO:001820	peptidyl-se	22/902	327/18614	0.075834	0.407054	0.365353	DYRK2/NE	22
GO:001633	BP	GO:001633	calcium-de	5/902	47/18614	0.075942	0.407054	0.365353	ATP2C1/CI	5
GO:003101	BP	GO:003101	endocrine	5/902	47/18614	0.075942	0.407054	0.365353	GATA6/SO	5
GO:004327	BP	GO:004327	apoptotic	5/902	47/18614	0.075942	0.407054	0.365353	NR1H3/TR	5
GO:005091	BP	GO:005091	negative cl	5/902	47/18614	0.075942	0.407054	0.365353	FLRT2/UN(	5
GO:006138	BP	GO:006138	trabecula r	5/902	47/18614	0.075942	0.407054	0.365353	GREM1/TG	5
GO:009019	BP	GO:009019	regulation	5/902	47/18614	0.075942	0.407054	0.365353	PMAIP1/BN	5
GO:012016	BP	GO:012016	negative re	5/902	47/18614	0.075942	0.407054	0.365353	NR1H3/NP	5
GO:005170	BP	GO:005170	biological	15/902	206/18614	0.076265	0.407054	0.365353	ITCH/TRIM	15
GO:009028	BP	GO:009028	regulation	23/902	345/18614	0.076475	0.407054	0.365353	GREM1/EN	23
GO:006076	BP	GO:006076	negative re	8/902	92/18614	0.077591	0.407054	0.365353	ZNF675/NI	8
GO:004312	BP	GO:004312	regulation	18/902	258/18614	0.077648	0.407054	0.365353	TRIM8/NEI	18
GO:005085	BP	GO:005085	T cell recep	11/902	140/18614	0.077999	0.407054	0.365353	BTN3A3/TI	11
GO:190300	BP	GO:190300	organelle c	11/902	140/18614	0.077999	0.407054	0.365353	ASCC3/SP/	11
GO:004802	BP	GO:004802	regulation	9/902	108/18614	0.078693	0.407054	0.365353	RBM20/TI/	9
GO:002290	BP	GO:002290	respiratory	10/902	124/18614	0.078713	0.407054	0.365353	GPD2/NDU	10
GO:003428	BP	GO:003428	response t	15/902	207/18614	0.078739	0.407054	0.365353	ZNF236/S(	15
GO:000649	BP	GO:000649	oligosacch	3/902	21/18614	0.078802	0.407054	0.365353	DPAGT1/A	3
GO:000908	BP	GO:000908	branched-	3/902	21/18614	0.078802	0.407054	0.365353	HIBCH/BCI	3
GO:000912	BP	GO:000912	pyrimidine	3/902	21/18614	0.078802	0.407054	0.365353	CMPK1/TY	3
GO:002175	BP	GO:002175	striatum de	3/902	21/18614	0.078802	0.407054	0.365353	FOXP1/RAI	3
GO:003128	BP	GO:003128	negative re	3/902	21/18614	0.078802	0.407054	0.365353	NPR3/GRM	3
GO:003201	BP	GO:003201	ARF protei	3/902	21/18614	0.078802	0.407054	0.365353	MAP4K4/C	3
GO:003201	BP	GO:003201	regulation	3/902	21/18614	0.078802	0.407054	0.365353	MAP4K4/C	3
GO:004409	BP	GO:004409	positive re	3/902	21/18614	0.078802	0.407054	0.365353	SNX18/ULI	3
GO:004506	BP	GO:004506	T-helper 1	3/902	21/18614	0.078802	0.407054	0.365353	IL4R/RELB/	3
GO:005122	BP	GO:005122	cytoplasmi	3/902	21/18614	0.078802	0.407054	0.365353	NFKBIE/PS	3
GO:006097	BP	GO:006097	cell migrati	3/902	21/18614	0.078802	0.407054	0.365353	ENG/FLRT:	3
GO:000668	BP	GO:000668	glycosphin	6/902	62/18614	0.078989	0.407054	0.365353	ST6GALNA	6
GO:004542	BP	GO:004542	regulation	6/902	62/18614	0.078989	0.407054	0.365353	GLA/HBB/I	6
GO:006026	BP	GO:006026	positive re	6/902	62/18614	0.078989	0.407054	0.365353	MED31/MI	6
GO:006195	BP	GO:006195	establishm	6/902	62/18614	0.078989	0.407054	0.365353	RAB31/ATI	6
GO:000028	BP	GO:000028	nuclear-tra	4/902	34/18614	0.080445	0.407054	0.365353	TNRC6B/C	4
GO:000689	BP	GO:000689	intra-Golgi	4/902	34/18614	0.080445	0.407054	0.365353	GOSR2/CC	4
GO:000911	BP	GO:000911	nucleobase	4/902	34/18614	0.080445	0.407054	0.365353	CMPK1/AD	4
GO:000995	BP	GO:000995	proximal/d	4/902	34/18614	0.080445	0.407054	0.365353	GREM1/IR)	4
GO:004337	BP	GO:004337	positive re	4/902	34/18614	0.080445	0.407054	0.365353	IL4R/CD83	4
GO:004564	BP	GO:004564	positive re	4/902	34/18614	0.080445	0.407054	0.365353	ISG15/HSP	4
GO:005197	BP	GO:005197	positive re	4/902	34/18614	0.080445	0.407054	0.365353	GREM1/PA	4
GO:009917	BP	GO:009917	presynapti	4/902	34/18614	0.080445	0.407054	0.365353	FBXL20/HT	4
GO:003030	BP	GO:003030	negative re	14/902	191/18614	0.081176	0.407054	0.365353	GREM1/TG	14
GO:003227	BP	GO:003227	regulation	14/902	191/18614	0.081176	0.407054	0.365353	CYFIP2/BA	14
GO:005077	BP	GO:005077	negative re	14/902	191/18614	0.081176	0.407054	0.365353	AHR/DUSP	14
GO:003464	BP	GO:003464	cellular res	8/902	93/18614	0.081487	0.407054	0.365353	MFAP4/BM	8
GO:005178	BP	GO:005178	positive re	8/902	93/18614	0.081487	0.407054	0.365353	TGFB2/VE(	8
GO:000321	BP	GO:000321	cardiac ver	2/902	10/18614	0.081532	0.407054	0.365353	SOX11/SO.	2
GO:000744	BP	GO:000744	foregut mc	2/902	10/18614	0.081532	0.407054	0.365353	CTNNB1/S	2
GO:000829	BP	GO:000829	intracellula	2/902	10/18614	0.081532	0.407054	0.365353	ZFP36L1/FI	2
GO:001076	BP	GO:001076	negative re	2/902	10/18614	0.081532	0.407054	0.365353	CYGB/ARH	2
GO:001088	BP	GO:001088	negative re	2/902	10/18614	0.081532	0.407054	0.365353	NR1H3/TT(	2
GO:001840	BP	GO:001840	peptidyl-p	2/902	10/18614	0.081532	0.407054	0.365353	P4HA1/P4I	2
GO:003022	BP	GO:003022	neutrophil	2/902	10/18614	0.081532	0.407054	0.365353	LBR/INPP5	2
GO:003319	BP	GO:003319	response t	2/902	10/18614	0.081532	0.407054	0.365353	CCND1/C(	2
GO:003533	BP	GO:003533	positive re	2/902	10/18614	0.081532	0.407054	0.365353	SOX11/ARI	2
GO:003564	BP	GO:003564	endosome	2/902	10/18614	0.081532	0.407054	0.365353	BLOC1S6/(	2
GO:003587	BP	GO:003587	protein K1:	2/902	10/18614	0.081532	0.407054	0.365353	TNFAIP3/Y	2
GO:004335	BP	GO:004335	enucleate	2/902	10/18614	0.081532	0.407054	0.365353	ID2/RB1	2
GO:004348	BP	GO:004348	endosome	2/902	10/18614	0.081532	0.407054	0.365353	BLOC1S6/(	2
GO:004358	BP	GO:004358	tongue mc	2/902	10/18614	0.081532	0.407054	0.365353	CYP26B1/C	2
GO:004562	BP	GO:004562	regulation	2/902	10/18614	0.081532	0.407054	0.365353	IL4R/ANXA	2
GO:004565	BP	GO:004565	positive re	2/902	10/18614	0.081532	0.407054	0.365353	ZFP36L1/C	2
GO:004579	BP	GO:004579	positive re	2/902	10/18614	0.081532	0.407054	0.365353	TMEM123/	2
GO:004819	BP	GO:004819	Golgi vesic	2/902	10/18614	0.081532	0.407054	0.365353	TMED2/AR	2
GO:004825	BP	GO:004825	elastic fibe	2/902	10/18614	0.081532	0.407054	0.365353	EMILIN1/M	2
GO:004826	BP	GO:004826	determinat	2/902	10/18614	0.081532	0.407054	0.365353	GREM1/CT	2
GO:004866	BP	GO:004866	neuron fat	2/902	10/18614	0.081532	0.407054	0.365353	CTNNB1/C	2
GO:004867	BP	GO:004867	negative re	2/902	10/18614	0.081532	0.407054	0.365353	ULK1/IFRD	2
GO:004875	BP	GO:004875	pigment gr	2/902	10/18614	0.081532	0.407054	0.365353	BLOC1S6/(	2
GO:006034	BP	GO:006034	bone trabe	2/902	10/18614	0.081532	0.407054	0.365353	GREM1/C(	2
GO:006085	BP	GO:006085	establishm	2/902	10/18614	0.081532	0.407054	0.365353	MFSD2A/N	2
GO:006204	BP	GO:006204	regulation	2/902	10/18614	0.081532	0.407054	0.365353	TGFB2/EN(	2
GO:007029	BP	GO:007029	renal sodiu	2/902	10/18614	0.081532	0.407054	0.365353	EDNRB/SG	2
GO:007107	BP	GO:007107	positive re	2/902	10/18614	0.081532	0.407054	0.365353	HTR2A/FAI	2
GO:009016	BP	GO:009016	Golgi ribbc	2/902	10/18614	0.081532	0.407054	0.365353	VAMP4/TR	2
GO:015017	BP	GO:015017	regulation	2/902	10/18614	0.081532	0.407054	0.365353	MFSD2A/F	2
GO:190223	BP	GO:190223	positive re	2/902	10/18614	0.081532	0.407054	0.365353	PMAIP1/PT	2
GO:190489	BP	GO:190489	ESCRT con	2/902	10/18614	0.081532	0.407054	0.365353	CHMP5/CH	2
GO:190490	BP	GO:190490	ESCRT III c	2/902	10/18614	0.081532	0.407054	0.365353	CHMP5/CH	2
GO:199050	BP	GO:199050	dense core	2/902	10/18614	0.081532	0.407054	0.365353	CADPS2/R/	2
GO:000611	BP	GO:000611	regulation	5/902	48/18614	0.081564	0.407054	0.365353	HTR2A/SIR	5
GO:003585	BP	GO:003585	epithelial c	5/902	48/18614	0.081564	0.407054	0.365353	GREM1/ST	5
GO:004659	BP	GO:004659	regulation	5/902	48/18614	0.081564	0.407054	0.365353	TRIM8/TRI	5
GO:199008	BP	GO:199008	response t	5/902	48/18614	0.081564	0.407054	0.365353	HSPA5/RAI	5
GO:000669	BP	GO:000669	icosanoid r	10/902	125/18614	0.082049	0.407054	0.365353	PTGDS/CYI	10
GO:001939	BP	GO:001939	fatty acid c	9/902	109/18614	0.082293	0.407054	0.365353	CYGB/MFS	9
GO:004533	BP	GO:004533	cellular res	17/902	243/18614	0.082916	0.407054	0.365353	GPD2/NDU	17
GO:005511	BP	GO:005511	regulation	7/902	78/18614	0.083458	0.407054	0.365353	SUMO1/HI	7
GO:000275	BP	GO:000275	innate imm	15/902	209/18614	0.083843	0.407054	0.365353	IRF1/ITCH/	15
GO:007138	BP	GO:007138	cellular res	15/902	209/18614	0.083843	0.407054	0.365353	TFPI/ZFP36	15
GO:004312	BP	GO:004312	positive re	14/902	192/18614	0.083872	0.407054	0.365353	TRIM8/NEI	14
GO:003163	BP	GO:003163	zymogen a	6/902	63/18614	0.083918	0.407054	0.365353	CYFIP2/PG	6
GO:007054	BP	GO:007054	response t	6/902	63/18614	0.083918	0.407054	0.365353	AKR1C3/M	6
GO:004576	BP	GO:004576	regulation	23/902	349/18614	0.084204	0.407054	0.365353	GREM1/EN	23
GO:200105	BP	GO:200105	positive re	11/902	142/18614	0.084277	0.407054	0.365353	CYFIP2/RP!	11
GO:000323	BP	GO:000323	cardiac ver	10/902	126/18614	0.085471	0.407054	0.365353	TGFB2/SO)	10
GO:000176	BP	GO:000176	neuron mic	13/902	176/18614	0.08639	0.407054	0.365353	LRP12/SRG	13
GO:003296	BP	GO:003296	positive re	5/902	49/18614	0.087401	0.407054	0.365353	ELL2/CDK1	5
GO:000189	BP	GO:000189	maternal p	4/902	35/18614	0.087513	0.407054	0.365353	TMED2/PT	4
GO:004577	BP	GO:004577	positive re	4/902	35/18614	0.087513	0.407054	0.365353	ID2/TPM1/	4
GO:004587	BP	GO:004587	negative re	4/902	35/18614	0.087513	0.407054	0.365353	GLIS2/RB1	4
GO:004804	BP	GO:004804	embryonic	4/902	35/18614	0.087513	0.407054	0.365353	SOX11/HIF	4
GO:007067	BP	GO:007067	response t	4/902	35/18614	0.087513	0.407054	0.365353	HSPA5/PTI	4
GO:007142	BP	GO:007142	hematopoi	4/902	35/18614	0.087513	0.407054	0.365353	NKAP/ME(	4
GO:001021	BP	GO:001021	response t	11/902	143/18614	0.087529	0.407054	0.365353	CCND1/HS	11
GO:001407	BP	GO:001407	response t	11/902	143/18614	0.087529	0.407054	0.365353	GATA6/ZFI	11
GO:000191	BP	GO:000191	regulation	3/902	22/18614	0.088096	0.407054	0.365353	CHMP5/RA	3
GO:000308	BP	GO:000308	negative re	3/902	22/18614	0.088096	0.407054	0.365353	KCNK6/SC	3
GO:000674	BP	GO:000674	NADPH re	3/902	22/18614	0.088096	0.407054	0.365353	G6PD/NNT	3
GO:001566	BP	GO:001566	gas transp	3/902	22/18614	0.088096	0.407054	0.365353	CYGB/HBB	3
GO:003431	BP	GO:003431	diol biosyn	3/902	22/18614	0.088096	0.407054	0.365353	SPR/ASAH	3
GO:004202	BP	GO:004202	protein ref	3/902	22/18614	0.088096	0.407054	0.365353	HSPA5/HSI	3
GO:004562	BP	GO:004562	positive re	3/902	22/18614	0.088096	0.407054	0.365353	IL4R/RARA	3
GO:004848	BP	GO:004848	sympathet	3/902	22/18614	0.088096	0.407054	0.365353	SOX11/SO:	3
GO:007163	BP	GO:007163	positive re	3/902	22/18614	0.088096	0.407054	0.365353	GATA6/LU	3
GO:190247	BP	GO:190247	regulation	3/902	22/18614	0.088096	0.407054	0.365353	RAP1A/DL(	3
GO:200104	BP	GO:200104	regulation	3/902	22/18614	0.088096	0.407054	0.365353	LIMS1/CD1	3
GO:007138	BP	GO:007138	cellular res	6/902	64/18614	0.089014	0.407054	0.365353	ZFP36L1/C	6
GO:000150	BP	GO:000150	regulation	15/902	211/18614	0.089154	0.407054	0.365353	CADPS2/BI	15
GO:000602	BP	GO:000602	proteoglyc	8/902	95/18614	0.08962	0.407054	0.365353	DSE/DSEL/	8
GO:004484	BP	GO:004484	cell cycle G	19/902	281/18614	0.090282	0.407054	0.365353	CCND1/RF	19
GO:005196	BP	GO:005196	regulation	29/902	461/18614	0.091514	0.407054	0.365353	DUSP10/VI	29
GO:000663	BP	GO:000663	fatty acid b	12/902	161/18614	0.091913	0.407054	0.365353	TRIB3/PTG	12
GO:001063	BP	GO:001063	epithelial c	24/902	371/18614	0.092551	0.407054	0.365353	GREM1/M/	24
GO:000229	BP	GO:000229	T cell differ	7/902	80/18614	0.092592	0.407054	0.365353	BCL3/FOXI	7
GO:004592	BP	GO:004592	positive re	7/902	80/18614	0.092592	0.407054	0.365353	CADPS2/C	7
GO:200014	BP	GO:200014	regulation	7/902	80/18614	0.092592	0.407054	0.365353	MED31/MI	7
GO:000206	BP	GO:000206	columnar/c	5/902	50/18614	0.093449	0.407054	0.365353	RHEB/RAR	5
GO:001086	BP	GO:001086	positive re	5/902	50/18614	0.093449	0.407054	0.365353	GDF15/TGI	5
GO:004345	BP	GO:004345	regulation	5/902	50/18614	0.093449	0.407054	0.365353	ISCU/SNC/	5
GO:004558	BP	GO:004558	negative re	5/902	50/18614	0.093449	0.407054	0.365353	IRF1/PTPN	5
GO:004347	BP	GO:004347	pigmentati	9/902	112/18614	0.093677	0.407054	0.365353	BLOC1S6/Z	9
GO:000704	BP	GO:000704	cell-substr	8/902	96/18614	0.093857	0.407054	0.365353	GREM1/M/	8
GO:003150	BP	GO:003150	heterochro	8/902	96/18614	0.093857	0.407054	0.365353	CBX3/MBD	8
GO:004580	BP	GO:004580	positive re	8/902	96/18614	0.093857	0.407054	0.365353	GREM1/RA	8
GO:007066	BP	GO:007066	negative re	8/902	96/18614	0.093857	0.407054	0.365353	GREM1/IT(	8
GO:008016	BP	GO:008016	regulation	6/902	65/18614	0.094274	0.407054	0.365353	GLA/HBB/I	6
GO:190533	BP	GO:190533	regulation	6/902	65/18614	0.094274	0.407054	0.365353	GREM1/FC	6
GO:000171	BP	GO:000171	ectoderma	1/902	2/18614	0.094571	0.407054	0.365353	FZD7	1
GO:000188	BP	GO:000188	selenium c	1/902	2/18614	0.094571	0.407054	0.365353	SEPHS2	1
GO:000194	BP	GO:000194	sebaceous	1/902	2/18614	0.094571	0.407054	0.365353	GATA6	1
GO:000226	BP	GO:000226	follicular de	1/902	2/18614	0.094571	0.407054	0.365353	BCL3	1
GO:000284	BP	GO:000284	negative re	1/902	2/18614	0.094571	0.407054	0.365353	AHR	1
GO:000293	BP	GO:000293	trabecular	1/902	2/18614	0.094571	0.407054	0.365353	CYP1B1	1
GO:000317	BP	GO:000317	sinoatrial v	1/902	2/18614	0.094571	0.407054	0.365353	SHOX2	1
GO:000318	BP	GO:000318	aortic valve	1/902	2/18614	0.094571	0.407054	0.365353	CDH11	1
GO:000334	BP	GO:000334	proepicard	1/902	2/18614	0.094571	0.407054	0.365353	ZFP36L1	1
GO:000334	BP	GO:000334	septum tra	1/902	2/18614	0.094571	0.407054	0.365353	ZFP36L1	1
GO:000612	BP	GO:000612	glycerophc	1/902	2/18614	0.094571	0.407054	0.365353	GPD2	1
GO:000631	BP	GO:000631	transpositi	1/902	2/18614	0.094571	0.407054	0.365353	PGBD5	1
GO:000642	BP	GO:000642	cysteinyl-t	1/902	2/18614	0.094571	0.407054	0.365353	CARS2	1
GO:000648	BP	GO:000648	N-termina	1/902	2/18614	0.094571	0.407054	0.365353	NTMT1	1
GO:000652	BP	GO:000652	asparagine	1/902	2/18614	0.094571	0.407054	0.365353	ASNSD1	1
GO:000662	BP	GO:000662	vacuolar p	1/902	2/18614	0.094571	0.407054	0.365353	TMEM208	1
GO:000671	BP	GO:000671	sesquiterp	1/902	2/18614	0.094571	0.407054	0.365353	AKR1C3	1
GO:000674	BP	GO:000674	NADP bios	1/902	2/18614	0.094571	0.407054	0.365353	NADK2	1
GO:000699	BP	GO:000699	positive re	1/902	2/18614	0.094571	0.407054	0.365353	XBP1	1
GO:000717	BP	GO:000717	epidermal	1/902	2/18614	0.094571	0.407054	0.365353	RAB7A	1
GO:000749	BP	GO:000749	endoderma	1/902	2/18614	0.094571	0.407054	0.365353	GATA6	1
GO:000749	BP	GO:000749	posterior n	1/902	2/18614	0.094571	0.407054	0.365353	EDNRB	1
GO:000751	BP	GO:000751	myoblast f	1/902	2/18614	0.094571	0.407054	0.365353	IFRD1	1
GO:000920	BP	GO:000920	deoxyribor	1/902	2/18614	0.094571	0.407054	0.365353	ADK	1
GO:000921	BP	GO:000921	purine deo	1/902	2/18614	0.094571	0.407054	0.365353	ADK	1
GO:000944	BP	GO:000944	putrescine	1/902	2/18614	0.094571	0.407054	0.365353	PAOX	1
GO:001042	BP	GO:001042	DNA meth	1/902	2/18614	0.094571	0.407054	0.365353	DNMT1	1
GO:001051	BP	GO:001051	negative re	1/902	2/18614	0.094571	0.407054	0.365353	PDGFA	1
GO:001073	BP	GO:001073	protein glu	1/902	2/18614	0.094571	0.407054	0.365353	G6PD	1
GO:001073	BP	GO:001073	regulation	1/902	2/18614	0.094571	0.407054	0.365353	G6PD	1
GO:001073	BP	GO:001073	negative re	1/902	2/18614	0.094571	0.407054	0.365353	G6PD	1
GO:001096	BP	GO:001096	regulation	1/902	2/18614	0.094571	0.407054	0.365353	PAOX	1
GO:001578	BP	GO:001578	UDP-gluco	1/902	2/18614	0.094571	0.407054	0.365353	SLC35B1	1
GO:001591	BP	GO:001591	fatty-acyl-	1/902	2/18614	0.094571	0.407054	0.365353	SLC33A1	1
GO:001610	BP	GO:001610	sesquiterp	1/902	2/18614	0.094571	0.407054	0.365353	AKR1C3	1

GO:000077	CC	GO:000077	condensed	12/1071	170/19518	0.224186	0.494664	0.444395	PINX1/DCT	12
GO:000564	CC	GO:000564	nuclear out	3/1071	30/19518	0.225826	0.494664	0.444395	ITPR3/BOK	3
GO:003526	CC	GO:003526	NuA4 histo	3/1071	30/19518	0.225826	0.494664	0.444395	RUVBL2/K	3
GO:004318	CC	GO:004318	H4/H2A his	3/1071	30/19518	0.225826	0.494664	0.444395	RUVBL2/K	3
GO:007100	CC	GO:007100	U2-type ca	3/1071	30/19518	0.225826	0.494664	0.444395	PRPF19/SN	3
GO:000079	CC	GO:000079	condensed	18/1071	270/19518	0.229231	0.494664	0.444395	NIFK/PINX	18
GO:000092	CC	GO:000092	spindle pol	12/1071	171/19518	0.230027	0.494664	0.444395	CNTRL/DC	12
GO:003190	CC	GO:003190	late endos	11/1071	155/19518	0.231773	0.494664	0.444395	VAMP8/PL	11
GO:000079	CC	GO:000079	euchromat	5/1071	60/19518	0.232058	0.494664	0.444395	JAK2/RUVE	5
GO:003509	CC	GO:003509	histone me	5/1071	60/19518	0.232058	0.494664	0.444395	RUVBL2/IN	5
GO:009868	CC	GO:009868	chromosor	25/1071	390/19518	0.23795	0.494664	0.444395	PINX1/DCT	25
GO:000587	CC	GO:000587	microtubul	11/1071	156/19518	0.238007	0.494664	0.444395	DCTN1/CL	11
GO:001628	CC	GO:001628	eukaryotic	2/1071	17/19518	0.23872	0.494664	0.444395	EIF3D/EIF3	2
GO:003000	CC	GO:003000	TRAPP con	2/1071	17/19518	0.23872	0.494664	0.444395	TRAPPC2L	2
GO:003109	CC	GO:003109	platelet alp	2/1071	17/19518	0.23872	0.494664	0.444395	SYTL4/CD9	2
GO:007100	CC	GO:007100	U2-type pr	2/1071	17/19518	0.23872	0.494664	0.444395	PRPF39/PR	2
GO:007101	CC	GO:007101	prespliceos	2/1071	17/19518	0.23872	0.494664	0.444395	PRPF39/PR	2
GO:007133	CC	GO:007133	MLL1 comp	3/1071	31/19518	0.240635	0.494664	0.444395	RUVBL2/IN	3
GO:000207	CC	GO:000207	inner acros	1/1071	5/19518	0.245882	0.494664	0.444395	CD46	1
GO:000559	CC	GO:000559	collagen ty	1/1071	5/19518	0.245882	0.494664	0.444395	COL11A2	1
GO:000563	CC	GO:000563	lamin filam	1/1071	5/19518	0.245882	0.494664	0.444395	EIF6	1
GO:000566	CC	GO:000566	DNA replic	1/1071	5/19518	0.245882	0.494664	0.444395	RFC4	1
GO:000585	CC	GO:000585	eukaryotic	1/1071	5/19518	0.245882	0.494664	0.444395	EIF2S1	1
GO:000835	CC	GO:000835	katanin co	1/1071	5/19518	0.245882	0.494664	0.444395	KATNA1	1
GO:003089	CC	GO:003089	apolipopro	1/1071	5/19518	0.245882	0.494664	0.444395	RBM47	1
GO:003142	CC	GO:003142	box H/ACA	1/1071	5/19518	0.245882	0.494664	0.444395	NHP2	1
GO:003150	CC	GO:003150	mannosyltr	1/1071	5/19518	0.245882	0.494664	0.444395	DPM3	1
GO:003298	CC	GO:003298	kainate sel	1/1071	5/19518	0.245882	0.494664	0.444395	GRIK2	1
GO:004525	CC	GO:004525	oxoglutara	1/1071	5/19518	0.245882	0.494664	0.444395	OGDH	1
GO:005128	CC	GO:005128	cell tip	1/1071	5/19518	0.245882	0.494664	0.444395	RDX	1
GO:006170	CC	GO:006170	GATOR2 co	1/1071	5/19518	0.245882	0.494664	0.444395	MIOS	1
GO:007181	CC	GO:007181	MMXD cor	1/1071	5/19518	0.245882	0.494664	0.444395	MMS19	1
GO:007198	CC	GO:007198	Ragulator	1/1071	5/19518	0.245882	0.494664	0.444395	LAMTOR4	1
GO:009066	CC	GO:009066	box H/ACA	1/1071	5/19518	0.245882	0.494664	0.444395	NHP2	1
GO:009702	CC	GO:009702	MPP7-DLG	1/1071	5/19518	0.245882	0.494664	0.444395	LIN7A	1
GO:009725	CC	GO:009725	R2TP comp	1/1071	5/19518	0.245882	0.494664	0.444395	RUVBL2	1
GO:009736	CC	GO:009736	CIA compl	1/1071	5/19518	0.245882	0.494664	0.444395	MMS19	1
GO:009742	CC	GO:009742	tubular enc	1/1071	5/19518	0.245882	0.494664	0.444395	VPS26A	1
GO:009772	CC	GO:009772	9+0 motile	1/1071	5/19518	0.245882	0.494664	0.444395	ENKUR	1
GO:009859	CC	GO:009859	cytoplasmi	1/1071	5/19518	0.245882	0.494664	0.444395	EZR	1
GO:009888	CC	GO:009888	extrinsic cc	1/1071	5/19518	0.245882	0.494664	0.444395	SNAP91	1
GO:009901	CC	GO:009901	neuronal d	1/1071	5/19518	0.245882	0.494664	0.444395	KIF1A	1
GO:010600	CC	GO:010600	mCRD-me	1/1071	5/19518	0.245882	0.494664	0.444395	SYNCRIP	1
GO:012004	CC	GO:012004	stereociliur	1/1071	5/19518	0.245882	0.494664	0.444395	TRIOBP	1
GO:003013	CC	GO:003013	COPII-coa	7/1071	93/19518	0.248834	0.498252	0.447618	SERPINA1/	7
GO:007060	CC	GO:007060	SWI/SNF s	7/1071	93/19518	0.248834	0.498252	0.447618	RUVBL2/IN	7
GO:004454	MF	GO:004454	S100 prote	7/1068	14/18369	5.27E-06	0.00829	0.008144	ANXA2/S1	7
GO:000377	MF	GO:000377	actin bindi	46/1068	434/18369	6.12E-05	0.048185	0.047337	COBL/PALI	46
GO:004529	MF	GO:004529	cadherin b	36/1068	333/18369	0.000255	0.133591	0.131241	COBLL1/C	36
GO:001645	MF	GO:001645	C-acetyltra	4/1068	7/18369	0.000345	0.135815	0.133427	ACAT1/GC	4
GO:004515	MF	GO:004515	myosin II b	5/1068	13/18369	0.000572	0.180019	0.176852	SHROOM1	5
GO:001640	MF	GO:001640	C-acyltran	6/1068	20/18369	0.000729	0.180858	0.177677	SPTLC2/AC	6
GO:000550	MF	GO:000550	copper ion	11/1068	62/18369	0.000804	0.180858	0.177677	SOD3/SUN	11
GO:000842	MF	GO:000842	phosphatic	4/1068	9/18369	0.001131	0.222483	0.21857	ESYT3/ESY	4
GO:000554	MF	GO:000554	phospholip	44/1068	475/18369	0.001558	0.272459	0.267667	PPT1/PCYT	44
GO:004679	MF	GO:004679	virion bindi	4/1068	11/18369	0.002696	0.33765	0.331712	ANXA2/PP	4
GO:001640	MF	GO:001640	dimethylar	2/1068	2/18369	0.003377	0.33765	0.331712	DDAH1/DD	2
GO:015004	MF	GO:015004	G protein-	2/1068	2/18369	0.003377	0.33765	0.331712	GABRB1/G	2
GO:003069	MF	GO:003069	GTPase reg	44/1068	495/18369	0.003401	0.33765	0.331712	TIAM2/ARI	44
GO:006058	MF	GO:006058	nucleoside	44/1068	495/18369	0.003401	0.33765	0.331712	TIAM2/ARI	44
GO:000508	MF	GO:000508	guanyl-nuc	24/1068	227/18369	0.003411	0.33765	0.331712	TIAM2/ARI	24
GO:000398	MF	GO:000398	acetyl-CoA	3/1068	6/18369	0.003432	0.33765	0.331712	ACAT1/AC	3
GO:000801	MF	GO:000801	beta-cater	12/1068	88/18369	0.004837	0.365975	0.359538	KANK1/RU	12
GO:001708	MF	GO:001708	sodium cha	7/1068	37/18369	0.004885	0.365975	0.359538	GPD1L/FXY	7
GO:000398	MF	GO:000398	acetyl-CoA	3/1068	7/18369	0.005748	0.365975	0.359538	ACAT1/AC	3
GO:007194	MF	GO:007194	FAD bindin	7/1068	39/18369	0.006598	0.365975	0.359538	CYB5R2/C	7
GO:005101	MF	GO:005101	actin filam	21/1068	202/18369	0.00714	0.365975	0.359538	SHROOM3	21
GO:003572	MF	GO:003572	lysophosph	3/1068	8/18369	0.008801	0.365975	0.359538	PPT1/LPAR	3
GO:000483	MF	GO:000483	ubiquitin ac	2/1068	3/18369	0.00974	0.365975	0.359538	SAE1/UBA	2
GO:003255	MF	GO:003255	pyrimidine	2/1068	3/18369	0.00974	0.365975	0.359538	UGP2/MTP	2
GO:004399	MF	GO:004399	H2A histon	2/1068	3/18369	0.00974	0.365975	0.359538	KAT5/NAA	2
GO:000843	MF	GO:000843	JUN kinase	3/1068	9/18369	0.012636	0.365975	0.359538	GSTP1/DU	3
GO:000801	MF	GO:000801	microtubul	25/1068	268/18369	0.01351	0.365975	0.359538	EML6/MX1	25
GO:003142	MF	GO:003142	alkali meta	4/1068	17/18369	0.014712	0.365975	0.359538	CAPN3/DR	4
GO:000436	MF	GO:000436	glutathione	5/1068	26/18369	0.015585	0.365975	0.359538	GSTZ1/GS	5
GO:003050	MF	GO:003050	spectrin bi	5/1068	26/18369	0.015585	0.365975	0.359538	ANK3/USH	5
GO:001677	MF	GO:001677	phosphotra	6/1068	36/18369	0.016579	0.365975	0.359538	NME7/AK7	6
GO:003126	MF	GO:003126	small GTPa	25/1068	273/18369	0.016678	0.365975	0.359538	ALS2CL/D	25
GO:001665	MF	GO:001665	oxidoreduc	3/1068	10/18369	0.017281	0.365975	0.359538	CYB5R2/C	3
GO:007056	MF	GO:007056	cytidylyltra	3/1068	10/18369	0.017281	0.365975	0.359538	PCYT1B/P	3
GO:000486	MF	GO:000486	serine-type	11/1068	92/18369	0.017578	0.365975	0.359538	SERPINA1/	11
GO:000476	MF	GO:000476	steroid del	2/1068	4/18369	0.018731	0.365975	0.359538	EBP/GSTA	2
GO:001640	MF	GO:001640	palmitoyl-	2/1068	4/18369	0.018731	0.365975	0.359538	ACADL/AC	2
GO:003580	MF	GO:003580	deubiquitir	2/1068	4/18369	0.018731	0.365975	0.359538	TIFAB/VCP	2
GO:005050	MF	GO:005050	hyalurona	2/1068	4/18369	0.018731	0.365975	0.359538	HYAL1/HA	2
GO:005272	MF	GO:005272	inositol he	2/1068	4/18369	0.018731	0.365975	0.359538	IP6K1/PPIP	2
GO:005272	MF	GO:005272	inositol he	2/1068	4/18369	0.018731	0.365975	0.359538	IP6K1/PPIP	2
GO:010629	MF	GO:010629	NADPH ox	2/1068	4/18369	0.018731	0.365975	0.359538	FMO5/DU	2
GO:003559	MF	GO:003559	signaling a	10/1068	83/18369	0.021872	0.365975	0.359538	MAGI2/FR	10
GO:000414	MF	GO:000414	diacylglyce	3/1068	11/18369	0.02275	0.365975	0.359538	DGKG/DG	3
GO:000553	MF	GO:000553	glucose bir	3/1068	11/18369	0.02275	0.365975	0.359538	UGP2/PYG	3
GO:005066	MF	GO:005066	flavin aden	10/1068	85/18369	0.025386	0.365975	0.359538	CYB5R2/C	10
GO:001663	MF	GO:001663	oxidoreduc	4/1068	20/18369	0.026088	0.365975	0.359538	LOXL2/GL	4
GO:000178	MF	GO:000178	phosphatic	8/1068	62/18369	0.02653	0.365975	0.359538	AXL/ASAP	8
GO:000452	MF	GO:000452	endoribonu	9/1068	74/18369	0.027266	0.365975	0.359538	RCL1/KHN	9
GO:000454	MF	GO:000454	ribonuclea	13/1068	124/18369	0.028239	0.365975	0.359538	RCL1/KHN	13
GO:001984	MF	GO:001984	vitamin bin	15/1068	150/18369	0.028337	0.365975	0.359538	SPTLC2/AL	15
GO:000509	MF	GO:000509	GTPase inh	3/1068	12/18369	0.029047	0.365975	0.359538	CDC42SE1	3
GO:000817	MF	GO:000817	adenylate	3/1068	12/18369	0.029047	0.365975	0.359538	ADRB2/CA	3
GO:003095	MF	GO:003095	potassium	3/1068	12/18369	0.029047	0.365975	0.359538	DRG1/ACA	3
GO:004697	MF	GO:004697	histone me	3/1068	12/18369	0.029047	0.365975	0.359538	SETD4/SM	3
GO:004802	MF	GO:004802	monosacch	9/1068	75/18369	0.029447	0.365975	0.359538	UGP2/ALK	9
GO:000554	MF	GO:000554	calcium-de	7/1068	52/18369	0.029983	0.365975	0.359538	ESYT3/ANX	7
GO:003015	MF	GO:003015	signaling re	7/1068	52/18369	0.029983	0.365975	0.359538	MAGI2/FR	7
GO:005128	MF	GO:005128	NAD bindi	7/1068	52/18369	0.029983	0.365975	0.359538	GPD1L/ND	7
GO:000082	MF	GO:000082	inositol-1,3	2/1068	5/18369	0.030022	0.365975	0.359538	IP6K1/PPIP	2
GO:000082	MF	GO:000082	inositol he	2/1068	5/18369	0.030022	0.365975	0.359538	IP6K1/PPIP	2
GO:000082	MF	GO:000082	inositol hep	2/1068	5/18369	0.030022	0.365975	0.359538	IP6K1/PPIP	2
GO:000083	MF	GO:000083	inositol he	2/1068	5/18369	0.030022	0.365975	0.359538	IP6K1/PPIP	2
GO:000445	MF	GO:000445	lactate deh	2/1068	5/18369	0.030022	0.365975	0.359538	LDHB/LDH	2
GO:000463	MF	GO:000463	phospholip	2/1068	5/18369	0.030022	0.365975	0.359538	PLD1/PLD3	2
GO:000465	MF	GO:000465	procollage	2/1068	5/18369	0.030022	0.365975	0.359538	P4HA3/P4	2
GO:000472	MF	GO:000472	protein-lys	2/1068	5/18369	0.030022	0.365975	0.359538	LOXL2/LO	2
GO:000474	MF	GO:000474	ribose pho	2/1068	5/18369	0.030022	0.365975	0.359538	PRPS1/PRP	2
GO:000549	MF	GO:000549	androgen	2/1068	5/18369	0.030022	0.365975	0.359538	AR/TSPO	2
GO:000841	MF	GO:000841	CoA-trans	2/1068	5/18369	0.030022	0.365975	0.359538	ACAA1/AC	2
GO:000937	MF	GO:000937	biotin bind	2/1068	5/18369	0.030022	0.365975	0.359538	PC/HLCS	2
GO:001983	MF	GO:001983	phospholip	2/1068	5/18369	0.030022	0.365975	0.359538	ANXA2/AN	2
GO:004691	MF	GO:004691	acyltransfe	2/1068	5/18369	0.030022	0.365975	0.359538	HMGCS1/	2
GO:005025	MF	GO:005025	retinyl-pal	2/1068	5/18369	0.030022	0.365975	0.359538	PNPLA4/P	2
GO:009710	MF	GO:009710	neuroligin	2/1068	5/18369	0.030022	0.365975	0.359538	NRXN3/SN	2
GO:014044	MF	GO:014044	cytoskelet	2/1068	5/18369	0.030022	0.365975	0.359538	SUN2/SYN	2
GO:012022	MF	GO:012022	acyl-CoA	4/1068	21/18369	0.030784	0.365975	0.359538	ACADL/AC	4
GO:014059	MF	GO:014059	protein car	5/1068	31/18369	0.03173	0.365975	0.359538	EMC10/PW	5
GO:005102	MF	GO:005102	GTPase bir	26/1068	304/18369	0.031793	0.365975	0.359538	ALS2CL/D	26
GO:000438	MF	GO:000438	helicase ac	15/1068	153/18369	0.033021	0.365975	0.359538	RUVBL2/H	15
GO:007003	MF	GO:007003	telomerase	4/1068	22/18369	0.035944	0.365975	0.359538	PINX1/EXC	4
GO:003445	MF	GO:003445	ubiquitin-	3/1068	13/18369	0.036165	0.365975	0.359538	PRPF19/PE	3
GO:003970	MF	GO:003970	co-recepto	3/1068	13/18369	0.036165	0.365975	0.359538	NEO1/DKK	3
GO:004356	MF	GO:004356	insulin rece	3/1068	13/18369	0.036165	0.365975	0.359538	JAK2/NCL/	3
GO:009715	MF	GO:009715	pre-mRNA	3/1068	13/18369	0.036165	0.365975	0.359538	RNPC3/HN	3
GO:000551	MF	GO:000551	collagen bi	8/1068	66/18369	0.036866	0.365975	0.359538	COL6A2/D	8
GO:001920	MF	GO:001920	nucleobase	6/1068	43/18369	0.036893	0.365975	0.359538	NME7/AK7	6
GO:014009	MF	GO:014009	catalytic ac	32/1068	396/18369	0.037634	0.365975	0.359538	RCL1/FARS	32
GO:000817	MF	GO:000817	O-methyltr	4/1068	23/18369	0.041573	0.365975	0.359538	LCMT1/AS	4
GO:001677	MF	GO:001677	diphospho	2/1068	6/18369	0.043317	0.365975	0.359538	PRPS1/PRP	2
GO:001910	MF	GO:001910	pyrimidine	2/1068	6/18369	0.043317	0.365975	0.359538	UGP2/MTP	2
GO:003170	MF	GO:003170	type 1 ang	2/1068	6/18369	0.043317	0.365975	0.359538	JAK2/AGT	2
GO:003524	MF	GO:003524	protein-arg	2/1068	6/18369	0.043317	0.365975	0.359538	PRMT3/PR	2
GO:010641	MF	GO:010641	XMP 5'-nu	2/1068	6/18369	0.043317	0.365975	0.359538	NT5C3A/N	2
GO:199044	MF	GO:199044	U1 snRNP	2/1068	6/18369	0.043317	0.365975	0.359538	SNRPD1/S	2
GO:001686	MF	GO:001686	intramolec	3/1068	14/18369	0.044089	0.365975	0.359538	EBP/GSTA	3
GO:003297	MF	GO:003297	membrane	3/1068	14/18369	0.044089	0.365975	0.359538	EMC10/TIM	3
GO:004302	MF	GO:004302	ribosomal	3/1068	14/18369	0.044089	0.365975	0.359538	NMD3/EIF	3
GO:001563	MF	GO:001563	tubulin bin	30/1068	374/18369	0.046619	0.365975	0.359538	EML6/MX1	30
GO:009910	MF	GO:009910	ion channe	14/1068	147/18369	0.047271	0.365975	0.359538	VAMP8/GF	14
GO:000554	MF	GO:000554	phosphatic	9/1068	82/18369	0.048191	0.365975	0.359538	ASAP1/AN	9
GO:003067	MF	GO:003067	protein-ma	29/1068	361/18369	0.04898	0.365975	0.359538	VAMP8/SR	29
GO:000487	MF	GO:000487	nuclear rec	6/1068	46/18369	0.04903	0.365975	0.359538	AR/NR1D1	6
GO:009853	MF	GO:009853	ligand-acti	6/1068	46/18369	0.04903	0.365975	0.359538	AR/NR1D1	6
GO:003027	MF	GO:003027	clathrin bin	8/1068	70/18369	0.049612	0.365975	0.359538	EPN2/AP4	8
GO:000367	MF	GO:000367	DNA helica	7/1068	58/18369	0.05012	0.365975	0.359538	RUVBL2/H	7
GO:000017	MF	GO:000017	sphingosin	1/1068	1/18369	0.058141	0.365975	0.359538	DEGS2	1
GO:000022	MF	GO:000022	N-acetylgl	1/1068	1/18369	0.058141	0.365975	0.359538	PIGL	1
GO:000024	MF	GO:000024	delta24(24	1/1068	1/18369	0.058141	0.365975	0.359538	DHCR24	1
GO:000024	MF	GO:000024	C-8 sterol	1/1068	1/18369	0.058141	0.365975	0.359538	EBP	1
GO:000025	MF	GO:000025	lanosterol	1/1068	1/18369	0.058141	0.365975	0.359538	LSS	1
GO:000385	MF	GO:000385	2-hydroxy	1/1068	1/18369	0.058141	0.365975	0.359538	UGT8	1
GO:000386	MF	GO:000386	4-aminob	1/1068	1/18369	0.058141	0.365975	0.359538	ABAT	1
GO:000387	MF	GO:000387	ATP citrate	1/1068	1/18369	0.058141	0.365975	0.359538	ACLY	1
GO:000390	MF	GO:000390	alkylbase D	1/1068	1/18369	0.058141	0.365975	0.359538	MPG	1
GO:000397	MF	GO:000397	UDP-N-ac	1/1068	1/18369	0.058141	0.365975	0.359538	GALE	1
GO:000397	MF	GO:000397	UDP-gluc	1/1068	1/18369	0.058141	0.365975	0.359538	GALE	1
GO:000398	MF	GO:000398	UTP:glucos	1/1068	1/18369	0.058141	0.365975	0.359538	UGP2	1
GO:000401	MF	GO:000401	adenosylho	1/1068	1/18369	0.058141	0.365975	0.359538	AHCY	1
GO:000401	MF	GO:000401	adenosylm	1/1068	1/18369	0.058141	0.365975	0.359538	AMD1	1
GO:000405	MF	GO:000405	argininosu	1/1068	1/18369	0.058141	0.365975	0.359538	ASL	1
GO:000405	MF	GO:000405	aromatic-L	1/1068	1/18369	0.058141	0.365975	0.359538	DDC	1
GO:000407	MF	GO:000407	biotin-[ace	1/1068	1/18369	0.058141	0.365975	0.359538	HLCS	1
GO:000407	MF	GO:000407	biotin-[me	1/1068	1/18369	0.058141	0.365975	0.359538	HLCS	1
GO:000407	MF	GO:000407	biotin-[me	1/1068	1/18369	0.058141	0.365975	0.359538	HLCS	1
GO:000408	MF	GO:000408	biotin-[pro	1/1068	1/18369	0.058141	0.365975	0.359538	HLCS	1
GO:000416	MF	GO:000416	diphthine s	1/1068	1/18369	0.058141	0.365975	0.359538	DPH5	1
GO:000430	MF	GO:000430	ethanolam	1/1068	1/18369	0.058141	0.365975	0.359538	PCYT2	1
GO:000431	MF	GO:000431	farnesyl-di	1/1068	1/18369	0.058141	0.365975	0.359538	FDFT1	1
GO:000434	MF	GO:000434	gluconolac	1/1068	1/18369	0.058141	0.365975	0.359538	RGN	1
GO:000436	MF	GO:000436	glutaryl-Co	1/1068	1/18369	0.058141	0.365975	0.359538	GCDH	1
GO:000437	MF	GO:000437	glycine deh	1/1068	1/18369	0.058141	0.365975	0.359538	GLDC	1
GO:000444	MF	GO:000444	inositol-1,4	1/1068	1/18369	0.058141	0.365975	0.359538	INPP1	1
GO:000445	MF	GO:000445	D-lactate	1/1068	1/18369	0.058141	0.365975	0.359538	LDHD	1
GO:000446	MF	GO:000446	lactoylglut	1/1068	1/18369	0.058141	0.365975	0.359538	GLO1	1
GO:000456	MF	GO:000456	beta-mann	1/1068	1/18369	0.058141	0.365975	0.359538	MANBA	1
GO:000461	MF	GO:000461	phosphogl	1/1068	1/18369	0.058141	0.365975	0.359538	PHGDH	1
GO:000463	MF	GO:000463	phosphom	1/1068	1/18369	0.058141	0.365975	0.359538	PMVK	1
GO:000463	MF	GO:000463	phosphorib	1/1068	1/18369	0.058141	0.365975	0.359538	PAICS	1
GO:000463	MF	GO:000463	phosphorib	1/1068	1/18369	0.058141	0.365975	0.359538	PAICS	1
GO:000464	MF	GO:000464	phosphorib	1/1068	1/18369	0.058141	0.365975	0.359538	PFAS	1
GO:000473	MF	GO:000473	pyruvate c	1/1068	1/18369	0.058141	0.365975	0.359538	PC	1
GO:000488	MF	GO:000488	nuclear glu	1/1068	1/18369	0.058141	0.365975	0.359538	NR3C1	1
GO:000494	MF	GO:000494	beta2-adre	1/1068	1/18369	0.058141	0.365975	0.359538	ADRB2	1
GO:000845	MF	GO:000845	alpha-N-a	1/1068	1/18369	0.058141	0.365975	0.359538	NAGA	1
GO:000868	MF	GO:000868	2-octapren	1/1068	1/18369	0.058141	0.365975	0.359538	COQ6	1
GO:000872	MF	GO:000872	D-lactate	1/1068	1/18369	0.058141	0.365975	0.359538	LDHD	1
GO:000872	MF	GO:000872	DNA-3-me	1/1068	1/18369	0.058141	0.365975	0.359538	MPG	1
GO:000877	MF	GO:000877	acetate Co	1/1068	1/18369	0.058141	0.365975	0.359538	ACAA1	1
GO:000889	MF	GO:000889	glycine C-a	1/1068	1/18369	0.058141	0.365975	0.359538	GCAT	1
GO:000899	MF	GO:000899	ribonuclea	1/1068	1/18369	0.058141	0.365975	0.359538	PPP1R8	1
GO:001531	MF	GO:001531	phosphate	1/1068	1/18369	0.058141	0.365975	0.359538	SLC25A3	1
GO:001542	MF	GO:001542	ABC-type	1/1068	1/18369	0.058141	0.365975	0.359538	ABCB9	1
GO:001603	MF	GO:001603	maleylacet	1/1068	1/18369	0.058141	0.365975	0.359538	GSTZ1	1
GO:001623	MF	GO:001623	HNK-1 sul	1/1068	1/18369	0.058141	0.365975	0.359538	CHST10	1
GO:001664	MF	GO:001664	oxidoreduc	1/1068	1/18369	0.058141	0.365975	0.359538	GLDC	1
GO:001689	MF	GO:001689	oxidoreduc	1/1068	1/18369	0.058141	0.365975	0.359538	LDHD	1
GO:001711	MF	GO:001711	lipoyltransf	1/1068	1/18369	0.058141	0.365975	0.359538	LIPT1	1
GO:001717	MF	GO:001717	diphthine-	1/1068	1/18369	0.058141	0.365975	0.359538	DPH6	1
GO:001827	MF	GO:001827	biotin-prot	1/1068	1/18369	0.058141	0.365975	0.359538	HLCS	1
GO:001842	MF	GO:001842	protein C-	1/1068	1/18369	0.058141	0.365975	0.359538	LCMT1	1
GO:001872	MF	GO:001872	propionate	1/1068	1/18369	0.058141	0.365975	0.359538	ACSM3	1
GO:001915	MF	GO:001915	D-ribulokir	1/1068	1/18369	0.058141	0.365975	0.359538	FGGY	1
GO:001981	MF	GO:001981	putrescine	1/1068	1/18369	0.058141	0.365975	0.359538	AMD1	1
GO:003058	MF	GO:003058	[methionin	1/1068	1/18369	0.058141	0.365975	0.359538	MTRR	1
GO:003155	MF	GO:003155	oxidosqual	1/1068	1/18369	0.058141	0.365975	0.359538	LSS	1
GO:003170	MF	GO:003170	adrenomed	1/1068	1/18369	0.058141	0.365975	0.359538	ADM	1
GO:003170	MF	GO:003170	type 2 ang	1/1068	1/18369	0.058141	0.365975	0.359538	AGT	1
GO:003185	MF	GO:003185	oxytocin re	1/1068	1/18369	0.058141	0.365975	0.359538	OXT	1
GO:003203	MF	GO:003203	myosin II h	1/1068	1/18369	0.058141	0.365975	0.359538	LIMCH1	1
GO:003214	MF	GO:003214	succinate-	1/1068	1/18369	0.058141	0.365975	0.359538	ABAT	1
GO:003393	MF	GO:003393	endogalac	1/1068	1/18369	0.058141	0.365975	0.359538	HYAL1	1
GO:003420	MF	GO:003420	glycolipid f	1/1068	1/18369	0.058141	0.365975	0.359538	RFT1	1
GO:003438	MF	GO:003438	4-aminobu	1/1068	1/18369	0.058141	0.365975	0.359538	ABAT	1
GO:003460	MF	GO:003460	oxoglutara	1/1068	1/18369	0.058141	0.365975	0.359538	OGDH	1
GO:003460	MF	GO:003460	oxoglutara	1/1068	1/18369	0.058141	0.365975	0.359538	OGDH	1
GO:003472	MF	GO:003472	gamma-gl	1/1068	1/18369	0.058141	0.365975	0.359538	GGH	1
GO:003540	MF	GO:003540	histone kin	1/1068	1/18369	0.058141	0.365975	0.359538	JAK2	1
GO:003548	MF	GO:003548	adenine/gu	1/1068	1/18369	0.058141	0.365975	0.359538	MUTYH	1
GO:003573	MF	GO:003573	S-nitrosog	1/1068	1/18369	0.058141	0.365975	0.359538	GSTP1	1
GO:003573	MF	GO:003573	dinitrosyl-i	1/1068	1/18369	0.058141	0.365975	0.359538	GSTP1	1
GO:003621	MF	GO:003621	dTTP diph	1/1068	1/18369	0.058141	0.365975	0.359538	ASMTL	1
GO:003646	MF	GO:003646	5-hydroxy-	1/1068	1/18369	0.058141	0.365975	0.359538	DDC	1
GO:003646	MF	GO:003646	L-dopa de	1/1068	1/18369	0.058141	0.365975	0.359538	DDC	1
GO:004201	MF	GO:004201	interleukin-	1/1068	1/18369	0.058141	0.365975	0.359538	IL22RA2	1
GO:004260	MF	GO:004260	riboflavin r	1/1068	1/18369	0.058141	0.365975	0.359538	BLVRB	1
GO:004372	MF	GO:004372	5-amino-4	1/1068	1/18369	0.058141	0.365975	0.359538	PAICS	1
GO:004373	MF	GO:004373	DNA-3-me	1/1068	1/18369	0.058141	0.365975	0.359538	MPG	1
GO:004375	MF	GO:004375	methylbuta	1/1068	1/18369	0.058141	0.365975	0.359538	ACSM3	1
GO:004384	MF	GO:004384	Ras palmit	1/1068	1/18369	0.058141	0.365975	0.359538	ZDHHC9	1
GO:004391	MF	GO:004391	DNA-7-me	1/1068	1/18369	0.058141	0.365975	0.359538	MPG	1
GO:004399	MF	GO:004399	histone ac	1/1068	1/18369	0.058141	0.365975	0.359538	KAT5	1
GO:004471	MF	GO:004471	8-hydroxy-	1/1068	1/18369	0.058141	0.365975	0.359538	NUDT18	1
GO:004512	MF	GO:004512	N-acetylgl	1/1068	1/18369	0.058141	0.365975	0.359538	NAGK	1
GO:004650	MF	GO:004650	hydrolase a	1/1068	1/18369	0.058141	0.365975	0.359538	AHCY	1
GO:004710	MF	GO:004710	aminomuc	1/1068	1/18369	0.058141	0.365975	0.359538	ALDH8A1	1
GO:004726	MF	GO:004726	N-acylsphi	1/1068	1/18369	0.058141	0.365975	0.359538	UGT8	1
GO:004729	MF	GO:004729	(S)-3-amir	1/1068	1/18369	0.058141	0.365975	0.359538	ABAT	1
GO:004798	MF	GO:004798	hydroxyaci	1/1068	1/18369	0.058141	0.365975	0.359538	ADHFE1	1
GO:004804	MF	GO:004804	UDP-gluc	1/1068	1/18369	0.058141	0.365975	0.359538	UXS1	1
GO:005003	MF	GO:005003	L-xylulose	1/1068	1/18369	0.058141	0.365975	0.359538	DCXR	1
GO:005010	MF	GO:005010	L-gulonate	1/1068	1/18369	0.058141	0.365975	0.359538	CRYL1	1
GO:005014	MF	GO:005014	nucleoside	1/1068	1/18369	0.058141	0.365975	0.359538	NT5C2	1
GO:005015	MF	GO:005015	omega-am	1/1068	1/18369	0.058141	0.365975	0.359538	NIT2	1
GO:005061	MF	GO:005061	delta24-ste	1/1068	1/18369	0.058141	0.365975	0.359538	DHCR24	1
GO:005174	MF	GO:005174	UTP-mon	1/1068	1/18369	0.058141	0.365975	0.359538	UGP2	1
GO:005199	MF	GO:005199	(R)-2-hydr	1/1068	1/18369	0.058141	0.365975	0.359538	D2HGDH	1
GO:005199	MF	GO:005199	squalene s	1/1068	1/18369	0.058141	0.365975	0.359538	FDFT1	1
GO:005275	MF	GO:005275	chondroitir	1/1068	1/18369	0.058141	0.365975	0.359538	HYAL1	1
GO:005282	MF	GO:005282	DNA-7-me	1/1068	1/18369	0.058141	0.365975	0.359538	MPG	1
GO:005282	MF	GO:005282	DNA-3-me	1/1068	1/18369	0.058141	0.365975	0.359538	MPG	1
GO:005282	MF	GO:005282	inositol-1,3	1/1068	1/18369	0.058141	0.365975	0.359538	INPP1	1
GO:005289	MF	GO:005289	norspermir	1/1068	1/18369	0.058141	0.365975	0.359538	SMOX	1
GO:005289	MF	GO:005289	N1-acetyls	1/1068	1/18369	0.058141	0.365975	0.359538	SMOX	1
GO:005290	MF	GO:005290	23S rRNA (	1/1068	1/18369	0.058141	0.365975	0.359538	METTL16	1
GO:005290	MF	GO:005290	18S rRNA (	1/1068	1/18369	0.058141	0.365975	0.359538	DIMT1	1
GO:005510	MF	GO:005510	ligase regu	1/1068	1/18369	0.058141	0.365975	0.359538	CAPN3	1
GO:006007	MF	GO:006007	large cond	1/1068	1/18369	0.058141	0.365975	0.359538	KCNMA1	1
GO:006070	MF	GO:006070	deoxyribor	1/1068	1/18369	0.058141	0.365975	0.359538	DFFA	1
GO:006192	MF	GO:006192	gamma-gl	1/1068	1/18369	0.058141	0.365975	0.359538	GGACT	1
GO:006210	MF	GO:006210	pumilio-re	1/1068	1/18369	0.058141	0.365975	0.359538	PUM2	1
GO:007011	MF	GO:007011	ciliary neur	1/1068	1/18369	0.058141	0.365975	0.359538	IL6R	1
GO:007012	MF	GO:007012	transformir	1/1068	1/18369	0.058141	0.365975	0.359538	TGFBR3	1
GO:007028	MF	GO:007028	pyridoxal b	1/1068	1/18369	0.058141	0.365975	0.359538	GLDC	1
GO:007053	MF	GO:007053	linoleic aci	1/1068	1/18369	0.058141	0.365975	0.359538	PPARD	1
GO:007097	MF	GO:007097	POU doma	1/1068	1/18369	0.058141	0.365975	0.359538	AR	1
GO:010600	MF	GO:010600	2-oxogluta	1/1068	1/18369	0.058141	0.365975	0.359538	NIT2	1
GO:010625	MF	GO:010625	L-serine-p	1/1068	1/18369	0.058141	0.365975	0.359538	PTDSS1	1
GO:010636	MF	GO:010636	4-hydroxy-	1/1068	1/18369	0.058141	0.365975	0.359538	COQ6	1
GO:012004	MF	GO:012004	U6 snRNA	1/1068	1/18369	0.058141	0.365975	0.359538	METTL16	1
GO:012014	MF	GO:012014	Formylglyc	1/1068	1/18369	0.058141	0.365975	0.359538	SUMF1	1
GO:014034	MF	GO:014034	triglyceride	1/1068	1/18369	0.058141	0.365975	0.359538	MTTP	1

GO:001611	BP	GO:001611	carotene m	1/902	2/18614	0.094571	0.407054	0.365353	BCO2	1
GO:001612	BP	GO:001612	carotene c	1/902	2/18614	0.094571	0.407054	0.365353	BCO2	1
GO:001612	BP	GO:001612	xanthophyl	1/902	2/18614	0.094571	0.407054	0.365353	BCO2	1
GO:001626	BP	GO:001626	selenocyst	1/902	2/18614	0.094571	0.407054	0.365353	SEPHS2	1
GO:001648	BP	GO:001648	farnesol m	1/902	2/18614	0.094571	0.407054	0.365353	AKR1C3	1
GO:001648	BP	GO:001648	farnesol ca	1/902	2/18614	0.094571	0.407054	0.365353	AKR1C3	1
GO:001811	BP	GO:001811	protein ad	1/902	2/18614	0.094571	0.407054	0.365353	FICD	1
GO:001817	BP	GO:001817	protein nu	1/902	2/18614	0.094571	0.407054	0.365353	FICD	1
GO:001952	BP	GO:001952	aldonic aci	1/902	2/18614	0.094571	0.407054	0.365353	PGD	1
GO:001952	BP	GO:001952	D-glucona	1/902	2/18614	0.094571	0.407054	0.365353	PGD	1
GO:002156	BP	GO:002156	abducens	1/902	2/18614	0.094571	0.407054	0.365353	MAFB	1
GO:002157	BP	GO:002157	rhombome	1/902	2/18614	0.094571	0.407054	0.365353	HOXB1	1
GO:002157	BP	GO:002157	hindbrain s	1/902	2/18614	0.094571	0.407054	0.365353	HSPA5	1
GO:002158	BP	GO:002158	cerebellum	1/902	2/18614	0.094571	0.407054	0.365353	HSPA5	1
GO:002159	BP	GO:002159	abducens	1/902	2/18614	0.094571	0.407054	0.365353	MAFB	1
GO:002159	BP	GO:002159	abducens	1/902	2/18614	0.094571	0.407054	0.365353	MAFB	1
GO:002175	BP	GO:002175	facial nucle	1/902	2/18614	0.094571	0.407054	0.365353	HOXB1	1
GO:002181	BP	GO:002181	extension	1/902	2/18614	0.094571	0.407054	0.365353	SRGAP2	1
GO:003201	BP	GO:003201	positive reg	1/902	2/18614	0.094571	0.407054	0.365353	MAP4K4	1
GO:003243	BP	GO:003243	activation	1/902	2/18614	0.094571	0.407054	0.365353	ANG	1
GO:003246	BP	GO:003246	positive reg	1/902	2/18614	0.094571	0.407054	0.365353	ISG15	1
GO:003246	BP	GO:003246	Golgi calci	1/902	2/18614	0.094571	0.407054	0.365353	ATP2C1	1
GO:003247	BP	GO:003247	Golgi calci	1/902	2/18614	0.094571	0.407054	0.365353	ATP2C1	1
GO:003248	BP	GO:003248	regulation	1/902	2/18614	0.094571	0.407054	0.365353	RGL2	1
GO:003274	BP	GO:003274	positive reg	1/902	2/18614	0.094571	0.407054	0.365353	FOXP1	1
GO:003291	BP	GO:003291	negative re	1/902	2/18614	0.094571	0.407054	0.365353	GATA6	1
GO:003291	BP	GO:003291	positive reg	1/902	2/18614	0.094571	0.407054	0.365353	SMAD3	1
GO:003336	BP	GO:003336	mast cell s	1/902	2/18614	0.094571	0.407054	0.365353	SRGN	1
GO:003412	BP	GO:003412	regulation	1/902	2/18614	0.094571	0.407054	0.365353	SARM1	1
GO:003439	BP	GO:003439	regulation	1/902	2/18614	0.094571	0.407054	0.365353	SLC40A1	1
GO:003475	BP	GO:003475	negative re	1/902	2/18614	0.094571	0.407054	0.365353	ISCU	1
GO:003476	BP	GO:003476	negative re	1/902	2/18614	0.094571	0.407054	0.365353	ISCU	1
GO:003598	BP	GO:003598	tongue mu	1/902	2/18614	0.094571	0.407054	0.365353	CYP26B1	1
GO:003637	BP	GO:003637	protein loc	1/902	2/18614	0.094571	0.407054	0.365353	ASB3	1
GO:003637	BP	GO:003637	opsin trans	1/902	2/18614	0.094571	0.407054	0.365353	IFT20	1
GO:003902	BP	GO:003902	pronephric	1/902	2/18614	0.094571	0.407054	0.365353	HNF1B	1
GO:004001	BP	GO:004001	positive reg	1/902	2/18614	0.094571	0.407054	0.365353	RFTN1	1
GO:004226	BP	GO:004226	peptidyl-a	1/902	2/18614	0.094571	0.407054	0.365353	ASPH	1
GO:004248	BP	GO:004248	positive reg	1/902	2/18614	0.094571	0.407054	0.365353	CSF1	1
GO:004263	BP	GO:004263	positive reg	1/902	2/18614	0.094571	0.407054	0.365353	TSKU	1
GO:004270	BP	GO:004270	uterine wal	1/902	2/18614	0.094571	0.407054	0.365353	TGFB2	1
GO:004433	BP	GO:004433	canonical W	1/902	2/18614	0.094571	0.407054	0.365353	CTNNB1	1
GO:004486	BP	GO:004486	modulatior	1/902	2/18614	0.094571	0.407054	0.365353	PTX3	1
GO:004486	BP	GO:004486	modulatior	1/902	2/18614	0.094571	0.407054	0.365353	PTX3	1
GO:004486	BP	GO:004486	negative re	1/902	2/18614	0.094571	0.407054	0.365353	PTX3	1
GO:004487	BP	GO:004487	modulatior	1/902	2/18614	0.094571	0.407054	0.365353	PTX3	1
GO:004487	BP	GO:004487	negative re	1/902	2/18614	0.094571	0.407054	0.365353	PTX3	1
GO:004558	BP	GO:004558	positive reg	1/902	2/18614	0.094571	0.407054	0.365353	TNFSF9	1
GO:004565	BP	GO:004565	negative re	1/902	2/18614	0.094571	0.407054	0.365353	INPP5D	1
GO:004582	BP	GO:004582	negative re	1/902	2/18614	0.094571	0.407054	0.365353	AKR1C3	1
GO:004597	BP	GO:004597	negative re	1/902	2/18614	0.094571	0.407054	0.365353	CTNNB1	1
GO:004617	BP	GO:004617	aldonic aci	1/902	2/18614	0.094571	0.407054	0.365353	PGD	1
GO:004617	BP	GO:004617	D-glucona	1/902	2/18614	0.094571	0.407054	0.365353	PGD	1
GO:004620	BP	GO:004620	spermidine	1/902	2/18614	0.094571	0.407054	0.365353	PAOX	1
GO:004620	BP	GO:004620	spermine c	1/902	2/18614	0.094571	0.407054	0.365353	PAOX	1
GO:004624	BP	GO:004624	terpene ca	1/902	2/18614	0.094571	0.407054	0.365353	BCO2	1
GO:004825	BP	GO:004825	iron import	1/902	2/18614	0.094571	0.407054	0.365353	SLC25A37	1
GO:004868	BP	GO:004868	regulation	1/902	2/18614	0.094571	0.407054	0.365353	TNR	1
GO:004868	BP	GO:004868	negative re	1/902	2/18614	0.094571	0.407054	0.365353	TNR	1
GO:004869	BP	GO:004869	regulation	1/902	2/18614	0.094571	0.407054	0.365353	TNR	1
GO:004869	BP	GO:004869	negative re	1/902	2/18614	0.094571	0.407054	0.365353	TNR	1
GO:005162	BP	GO:005162	regulation	1/902	2/18614	0.094571	0.407054	0.365353	SNCA	1
GO:005247	BP	GO:005247	modulatior	1/902	2/18614	0.094571	0.407054	0.365353	SAP30	1
GO:006101	BP	GO:006101	hepatoblas	1/902	2/18614	0.094571	0.407054	0.365353	HNF1B	1
GO:006103	BP	GO:006103	endoderma	1/902	2/18614	0.094571	0.407054	0.365353	ID2	1
GO:006104	BP	GO:006104	positive reg	1/902	2/18614	0.094571	0.407054	0.365353	CTNNB1	1
GO:006138	BP	GO:006138	closure of	1/902	2/18614	0.094571	0.407054	0.365353	SOX11	1
GO:006202	BP	GO:006202	positive reg	1/902	2/18614	0.094571	0.407054	0.365353	G3BP2	1
GO:006204	BP	GO:006204	negative re	1/902	2/18614	0.094571	0.407054	0.365353	TGFB2	1
GO:007056	BP	GO:007056	negative re	1/902	2/18614	0.094571	0.407054	0.365353	KANK2	1
GO:007100	BP	GO:007100	response t	1/902	2/18614	0.094571	0.407054	0.365353	SOD2	1
GO:007133	BP	GO:007133	hair follicle	1/902	2/18614	0.094571	0.407054	0.365353	DLX3	1
GO:007138	BP	GO:007138	cellular res	1/902	2/18614	0.094571	0.407054	0.365353	CYP1B1	1
GO:007157	BP	GO:007157	shelterin co	1/902	2/18614	0.094571	0.407054	0.365353	ZNF827	1
GO:007205	BP	GO:007205	renal inner	1/902	2/18614	0.094571	0.407054	0.365353	CTNNB1	1
GO:007205	BP	GO:007205	renal outer	1/902	2/18614	0.094571	0.407054	0.365353	CTNNB1	1
GO:007211	BP	GO:007211	pronephro	1/902	2/18614	0.094571	0.407054	0.365353	HNF1B	1
GO:007218	BP	GO:007218	mesoneph	1/902	2/18614	0.094571	0.407054	0.365353	HNF1B	1
GO:007218	BP	GO:007218	negative re	1/902	2/18614	0.094571	0.407054	0.365353	STAT1	1
GO:007257	BP	GO:007257	tolerance i	1/902	2/18614	0.094571	0.407054	0.365353	TNFAIP3	1
GO:009018	BP	GO:009018	regulation	1/902	2/18614	0.094571	0.407054	0.365353	NR1H3	1
GO:009032	BP	GO:009032	negative re	1/902	2/18614	0.094571	0.407054	0.365353	ARRDC3	1
GO:009716	BP	GO:009716	lens epithe	1/902	2/18614	0.094571	0.407054	0.365353	SOX11	1
GO:009721	BP	GO:009721	positive reg	1/902	2/18614	0.094571	0.407054	0.365353	LAPTM5	1
GO:009733	BP	GO:009733	response t	1/902	2/18614	0.094571	0.407054	0.365353	PRKAR2B	1
GO:009733	BP	GO:009733	response t	1/902	2/18614	0.094571	0.407054	0.365353	PRKAR2B	1
GO:009740	BP	GO:009740	cellular res	1/902	2/18614	0.094571	0.407054	0.365353	ZFP36L1	1
GO:009862	BP	GO:009862	trans-Golg	1/902	2/18614	0.094571	0.407054	0.365353	ATP2C1	1
GO:009893	BP	GO:009893	dendritic tr	1/902	2/18614	0.094571	0.407054	0.365353	TRAK2	1
GO:009904	BP	GO:009904	vesicle teth	1/902	2/18614	0.094571	0.407054	0.365353	STARD3	1
GO:009954	BP	GO:009954	trans-syna	1/902	2/18614	0.094571	0.407054	0.365353	NTRK2	1
GO:009964	BP	GO:009964	retrograde	1/902	2/18614	0.094571	0.407054	0.365353	MAP1A	1
GO:010621	BP	GO:010621	tRNA C3-c	1/902	2/18614	0.094571	0.407054	0.365353	METTL8	1
GO:190005	BP	GO:190005	negative re	1/902	2/18614	0.094571	0.407054	0.365353	AKR1C3	1
GO:190015	BP	GO:190015	regulation	1/902	2/18614	0.094571	0.407054	0.365353	GREM1	1
GO:190015	BP	GO:190015	negative re	1/902	2/18614	0.094571	0.407054	0.365353	GREM1	1
GO:190023	BP	GO:190023	regulation	1/902	2/18614	0.094571	0.407054	0.365353	SH2B3	1
GO:190023	BP	GO:190023	negative re	1/902	2/18614	0.094571	0.407054	0.365353	SH2B3	1
GO:190024	BP	GO:190024	negative re	1/902	2/18614	0.094571	0.407054	0.365353	CPEB3	1
GO:190133	BP	GO:190133	thioester tr	1/902	2/18614	0.094571	0.407054	0.365353	SLC33A1	1
GO:190154	BP	GO:190154	response t	1/902	2/18614	0.094571	0.407054	0.365353	ZFP36L1	1
GO:190182	BP	GO:190182	zeaxanthin	1/902	2/18614	0.094571	0.407054	0.365353	BCO2	1
GO:190189	BP	GO:190189	positive reg	1/902	2/18614	0.094571	0.407054	0.365353	HRC	1
GO:190195	BP	GO:190195	regulation	1/902	2/18614	0.094571	0.407054	0.365353	MAP2	1
GO:190195	BP	GO:190195	positive reg	1/902	2/18614	0.094571	0.407054	0.365353	MAP2	1
GO:190196	BP	GO:190196	regulation	1/902	2/18614	0.094571	0.407054	0.365353	CTNNB1	1
GO:190261	BP	GO:190261	cellular res	1/902	2/18614	0.094571	0.407054	0.365353	COL1A1	1
GO:190263	BP	GO:190263	negative re	1/902	2/18614	0.094571	0.407054	0.365353	SOD2	1
GO:190280	BP	GO:190280	positive reg	1/902	2/18614	0.094571	0.407054	0.365353	MAP2	1
GO:190301	BP	GO:190301	negative re	1/902	2/18614	0.094571	0.407054	0.365353	PTX3	1
GO:190305	BP	GO:190305	regulation	1/902	2/18614	0.094571	0.407054	0.365353	ZEB2	1
GO:190328	BP	GO:190328	regulation	1/902	2/18614	0.094571	0.407054	0.365353	SNCA	1
GO:190338	BP	GO:190338	regulation	1/902	2/18614	0.094571	0.407054	0.365353	HYOU1	1
GO:190338	BP	GO:190338	negative re	1/902	2/18614	0.094571	0.407054	0.365353	HYOU1	1
GO:190348	BP	GO:190348	positive reg	1/902	2/18614	0.094571	0.407054	0.365353	XBP1	1
GO:190354	BP	GO:190354	response t	1/902	2/18614	0.094571	0.407054	0.365353	PIK3CA	1
GO:190374	BP	GO:190374	positive reg	1/902	2/18614	0.094571	0.407054	0.365353	MAP2	1
GO:190400	BP	GO:190400	regulation	1/902	2/18614	0.094571	0.407054	0.365353	GATA6	1
GO:190409	BP	GO:190409	negative re	1/902	2/18614	0.094571	0.407054	0.365353	LAPTM5	1
GO:190416	BP	GO:190416	DNA synth	1/902	2/18614	0.094571	0.407054	0.365353	TREX1	1
GO:190420	BP	GO:190420	iodide tran	1/902	2/18614	0.094571	0.407054	0.365353	SLC5A5	1
GO:190431	BP	GO:190431	response t	1/902	2/18614	0.094571	0.407054	0.365353	HSPA5	1
GO:190440	BP	GO:190440	response t	1/902	2/18614	0.094571	0.407054	0.365353	SLC5A5	1
GO:190452	BP	GO:190452	negative re	1/902	2/18614	0.094571	0.407054	0.365353	MAP2	1
GO:190454	BP	GO:190454	regulation	1/902	2/18614	0.094571	0.407054	0.365353	AMBRA1	1
GO:190454	BP	GO:190454	positive reg	1/902	2/18614	0.094571	0.407054	0.365353	AMBRA1	1
GO:190455	BP	GO:190455	L-tryptoph	1/902	2/18614	0.094571	0.407054	0.365353	SLC36A4	1
GO:190461	BP	GO:190461	cellular res	1/902	2/18614	0.094571	0.407054	0.365353	AHR	1
GO:190479	BP	GO:190479	regulation	1/902	2/18614	0.094571	0.407054	0.365353	ZNF827	1
GO:190480	BP	GO:190480	regulation	1/902	2/18614	0.094571	0.407054	0.365353	MAP2	1
GO:190481	BP	GO:190481	positive reg	1/902	2/18614	0.094571	0.407054	0.365353	MAP2	1
GO:190497	BP	GO:190497	negative re	1/902	2/18614	0.094571	0.407054	0.365353	RAB7A	1
GO:190500	BP	GO:190500	negative re	1/902	2/18614	0.094571	0.407054	0.365353	TGFB2	1
GO:190536	BP	GO:190536	regulation	1/902	2/18614	0.094571	0.407054	0.365353	RAB7A	1
GO:190536	BP	GO:190536	negative re	1/902	2/18614	0.094571	0.407054	0.365353	RAB7A	1
GO:190537	BP	GO:190537	ceramide	1/902	2/18614	0.094571	0.407054	0.365353	SAMD8	1
GO:190537	BP	GO:190537	ceramide	1/902	2/18614	0.094571	0.407054	0.365353	SAMD8	1
GO:190539	BP	GO:190539	response t	1/902	2/18614	0.094571	0.407054	0.365353	NQO1	1
GO:190559	BP	GO:190559	fibronectin	1/902	2/18614	0.094571	0.407054	0.365353	TNFAIP6	1
GO:199040	BP	GO:199040	embryonic	1/902	2/18614	0.094571	0.407054	0.365353	PDGFA	1
GO:199078	BP	GO:199078	response t	1/902	2/18614	0.094571	0.407054	0.365353	RIOK3	1
GO:199078	BP	GO:199078	cellular res	1/902	2/18614	0.094571	0.407054	0.365353	RIOK3	1
GO:199083	BP	GO:199083	response t	1/902	2/18614	0.094571	0.407054	0.365353	EDNRB	1
GO:200001	BP	GO:200001	positive reg	1/902	2/18614	0.094571	0.407054	0.365353	CTNNB1	1
GO:200034	BP	GO:200034	negative re	1/902	2/18614	0.094571	0.407054	0.365353	TNFAIP3	1
GO:200047	BP	GO:200047	positive reg	1/902	2/18614	0.094571	0.407054	0.365353	SNCA	1
GO:200053	BP	GO:200053	negative re	1/902	2/18614	0.094571	0.407054	0.365353	ITGAV	1
GO:200064	BP	GO:200064	negative re	1/902	2/18614	0.094571	0.407054	0.365353	SNX12	1
GO:200103	BP	GO:200103	regulation	1/902	2/18614	0.094571	0.407054	0.365353	CYP26B1	1
GO:200103	BP	GO:200103	positive reg	1/902	2/18614	0.094571	0.407054	0.365353	CYP26B1	1
GO:200110	BP	GO:200110	regulation	1/902	2/18614	0.094571	0.407054	0.365353	SOX11	1
GO:200111	BP	GO:200111	positive reg	1/902	2/18614	0.094571	0.407054	0.365353	SOX11	1
GO:000206	BP	GO:000206	glandular e	4/902	36/18614	0.094871	0.407054	0.365353	RHEB/RAR	4
GO:000247	BP	GO:000247	antigen pr	4/902	36/18614	0.094871	0.407054	0.365353	ERAP1/TAP	4
GO:001061	BP	GO:001061	negative re	4/902	36/18614	0.094871	0.407054	0.365353	FOXP1/G6	4
GO:002179	BP	GO:002179	cerebral cc	4/902	36/18614	0.094871	0.407054	0.365353	SRGAP2/W	4
GO:004873	BP	GO:004873	epidermis	4/902	36/18614	0.094871	0.407054	0.365353	TGFB2/DLX	4
GO:190372	BP	GO:190372	regulation	4/902	36/18614	0.094871	0.407054	0.365353	MFSD2A/H	4
GO:190533	BP	GO:190533	positive reg	4/902	36/18614	0.094871	0.407054	0.365353	GREM1/SIF	4
GO:004259	BP	GO:004259	glucose ho	17/902	248/18614	0.0954	0.407054	0.365353	SESN3/ZN	17
GO:000165	BP	GO:000165	temperatu	13/902	179/18614	0.095415	0.407054	0.365353	NR1H3/NP	13
GO:001907	BP	GO:001907	viral genor	10/902	129/18614	0.096247	0.407054	0.365353	PPIB/OASL	10
GO:002170	BP	GO:002170	developme	21/902	319/18614	0.09628	0.407054	0.365353	GREM1/BL	21
GO:000154	BP	GO:000154	antral ovar	2/902	11/18614	0.096547	0.407054	0.365353	TNFAIP6/P	2
GO:000166	BP	GO:000166	fever gene	2/902	11/18614	0.096547	0.407054	0.365353	PTGES/ED	2
GO:000335	BP	GO:000335	noradrener	2/902	11/18614	0.096547	0.407054	0.365353	SOX11/SO	2
GO:001026	BP	GO:001026	response t	2/902	11/18614	0.096547	0.407054	0.365353	SOD2/TXN	2
GO:001474	BP	GO:001474	negative re	2/902	11/18614	0.096547	0.407054	0.365353	FOXO1/SM	2
GO:002260	BP	GO:002260	mammalia	2/902	11/18614	0.096547	0.407054	0.365353	TNFAIP6/P	2
GO:003134	BP	GO:003134	positive reg	2/902	11/18614	0.096547	0.407054	0.365353	SNCA/ANX	2
GO:003226	BP	GO:003226	purine nucl	2/902	11/18614	0.096547	0.407054	0.365353	NAPRT/AD	2
GO:003242	BP	GO:003242	regulation	2/902	11/18614	0.096547	0.407054	0.365353	ANG/ANXA	2
GO:004248	BP	GO:004248	regulation	2/902	11/18614	0.096547	0.407054	0.365353	CSF1/RUN	2
GO:004484	BP	GO:004484	biological	2/902	11/18614	0.096547	0.407054	0.365353	TGFB2/CTN	2
GO:004664	BP	GO:004664	regulation	2/902	11/18614	0.096547	0.407054	0.365353	SOX4/LILR	2
GO:004826	BP	GO:004826	determinat	2/902	11/18614	0.096547	0.407054	0.365353	GREM1/CT	2
GO:005146	BP	GO:005146	vesicle fusi	2/902	11/18614	0.096547	0.407054	0.365353	CHMP5/CH	2
GO:006017	BP	GO:006017	limb bud fo	2/902	11/18614	0.096547	0.407054	0.365353	SOX11/SO	2
GO:006027	BP	GO:006027	embryonic	2/902	11/18614	0.096547	0.407054	0.365353	SHOX2/CT	2
GO:006062	BP	GO:006062	regulation	2/902	11/18614	0.096547	0.407054	0.365353	SMC4/SMC	2
GO:006114	BP	GO:006114	lung secret	2/902	11/18614	0.096547	0.407054	0.365353	GATA6/RB	2
GO:006176	BP	GO:006176	multivesicu	2/902	11/18614	0.096547	0.407054	0.365353	CHMP5/CH	2
GO:007060	BP	GO:007060	centromeri	2/902	11/18614	0.096547	0.407054	0.365353	NAA50/CT	2
GO:007094	BP	GO:007094	neutrophil-	2/902	11/18614	0.096547	0.407054	0.365353	TREM1/MY	2
GO:007271	BP	GO:007271	cellular res	2/902	11/18614	0.096547	0.407054	0.365353	SPIDR/TRE	2
GO:008610	BP	GO:008610	endothelin	2/902	11/18614	0.096547	0.407054	0.365353	EDNRB/CD	2
GO:009048	BP	GO:009048	pyrimidine	2/902	11/18614	0.096547	0.407054	0.365353	SLC35A3/S	2
GO:009869	BP	GO:009869	regulation	2/902	11/18614	0.096547	0.407054	0.365353	RAP1A/GP	2
GO:190036	BP	GO:190036	regulation	2/902	11/18614	0.096547	0.407054	0.365353	CPEB3/CPS	2
GO:190120	BP	GO:190120	positive reg	2/902	11/18614	0.096547	0.407054	0.365353	EMILIN1/S	2
GO:190184	BP	GO:190184	regulation	2/902	11/18614	0.096547	0.407054	0.365353	IRX3/HRC	2
GO:190305	BP	GO:190305	regulation	2/902	11/18614	0.096547	0.407054	0.365353	SVIP/ULK1	2
GO:190389	BP	GO:190389	regulation	2/902	11/18614	0.096547	0.407054	0.365353	HSPA5/PTP	2
GO:200064	BP	GO:200064	regulation	2/902	11/18614	0.096547	0.407054	0.365353	ITCH/LAPT	2
GO:200067	BP	GO:200067	negative re	2/902	11/18614	0.096547	0.407054	0.365353	ZPR1/CRLF	2
GO:200122	BP	GO:200122	negative re	2/902	11/18614	0.096547	0.407054	0.365353	SRGAP2/A	2
GO:004578	BP	GO:004578	positive reg	30/902	482/18614	0.096556	0.407054	0.365353	EMILIN1/M	30
GO:007124	BP	GO:007124	cellular res	16/902	231/18614	0.096558	0.407054	0.365353	SUMO1/SY	16
GO:190134	BP	GO:190134	regulation	23/902	355/18614	0.096743	0.407574	0.36582	GREM1/EM	23
GO:003646	BP	GO:003646	synaptic ve	7/902	81/18614	0.09736	0.409812	0.367829	SYT8/DGK	7
GO:000665	BP	GO:000665	phosphatic	3/902	23/18614	0.097825	0.409812	0.367829	MFSD2A/P	3
GO:001943	BP	GO:001943	removal of	3/902	23/18614	0.097825	0.409812	0.367829	NQO1/SO	3
GO:004482	BP	GO:004482	modulatior	3/902	23/18614	0.097825	0.409812	0.367829	PPIB/TBC1	3
GO:005145	BP	GO:005145	maintenan	3/902	23/18614	0.097825	0.409812	0.367829	MORC3/AF	3
GO:007257	BP	GO:007257	hepatocyte	3/902	23/18614	0.097825	0.409812	0.367829	TNFAIP3/P	3
GO:007257	BP	GO:007257	epithelial c	3/902	23/18614	0.097825	0.409812	0.367829	TNFAIP3/P	3
GO:009715	BP	GO:009715	neuronal st	3/902	23/18614	0.097825	0.409812	0.367829	WDR47/HC	3
GO:190167	BP	GO:190167	regulation	3/902	23/18614	0.097825	0.409812	0.367829	CHMP5/HS	3
GO:003350	BP	GO:003350	carbohydra	17/902	249/18614	0.098032	0.410121	0.368106	SESN3/ZN	17
GO:000741	BP	GO:000741	synapse as	14/902	197/18614	0.098202	0.410121	0.368106	RAP2A/SR	14
GO:000004	BP	GO:000004	transition	8/902	97/18614	0.098206	0.410121	0.368106	ISCU/ATP2	8
GO:000694	BP	GO:000694	regulation	8/902	97/18614	0.098206	0.410121	0.368106	SUMO1/HI	8
GO:190332	BP	GO:190332	negative re	8/902	97/18614	0.098206	0.410121	0.368106	BAG2/SOX	8
GO:002191	BP	GO:002191	neural tube	12/902	163/18614	0.098442	0.410851	0.368761	ZFP36L1/T	12
GO:009013	BP	GO:009013	epithelium	24/902	374/18614	0.098897	0.412491	0.370233	GREM1/M	24
GO:005109	BP	GO:005109	regulation	29/902	465/18614	0.099053	0.412886	0.370588	GREM1/IT	29
GO:005077	BP	GO:005077	negative re	6/902	66/18614	0.099697	0.414833	0.372336	ULK1/IFRD	6
GO:001587	BP	GO:001587	dopamine	5/902	51/18614	0.099706	0.414833	0.372336	SYT8/HTR2	5
GO:005118	BP	GO:005118	vitamin tra	5/902	51/18614	0.099706	0.414833	0.372336	AMN/SLC2	5
GO:000164	BP	GO:000164	osteoblast	17/902	250/18614	0.100709	0.418744	0.375845	GREM1/TN	17
GO:000854	BP	GO:000854	epidermis	24/902	375/18614	0.101073	0.419995	0.376969	GATA6/MA	24
GO:000717	BP	GO:000717	epidermal	9/902	114/18614	0.101753	0.421546	0.378361	PIK3C2A/D	9
GO:003355	BP	GO:003355	unsaturate	9/902	114/18614	0.101753	0.421546	0.378361	PTGDS/CY	9
GO:004663	BP	GO:004663	regulation	9/902	114/18614	0.101753	0.421546	0.378361	IRF1/ITCH/	9
GO:005196	BP	GO:005196	regulation	9/902	114/18614	0.101753	0.421546	0.378361	RAP2A/SLI	9
GO:007066	BP	GO:007066	regulation	18/902	268/18614	0.101761	0.421546	0.378361	GREM1/IR	18
GO:000690	BP	GO:000690	vesicle buc	7/902	82/18614	0.10226	0.422028	0.378793	CHMP5/TM	7
GO:004692	BP	GO:004692	regulation	7/902	82/18614	0.10226	0.422028	0.378793	FBXL20/RA	7
GO:000323	BP	GO:000323	cardiac atr	4/902	37/18614	0.102509	0.422028	0.378793	TGFB2/SO	4
GO:000820	BP	GO:000820	C21-steroi	4/902	37/18614	0.102509	0.422028	0.378793	STARD3/D	4
GO:001093	BP	GO:001093	macropha	4/902	37/18614	0.102509	0.422028	0.378793	TGFB2/MY	4
GO:001093	BP	GO:001093	regulation	4/902	37/18614	0.102509	0.422028	0.378793	TGFB2/MY	4
GO:003314	BP	GO:003314	negative re	4/902	37/18614	0.102509	0.422028	0.378793	FOXP1/ZB	4
GO:003588	BP	GO:003588	enteroend	4/902	37/18614	0.102509	0.422028	0.378793	GATA6/RH	4
GO:004351	BP	GO:004351	regulation	4/902	37/18614	0.102509	0.422028	0.378793	PMAIP1/SO	4
GO:009734	BP	GO:009734	mitochond	4/902	37/18614	0.102509	0.422028	0.378793	BID/SLC35	4
GO:004327	BP	GO:004327	response t	8/902	98/18614	0.102667	0.422418	0.379144	HSPA5/HT	8
GO:200011	BP	GO:200011	regulation	16/902	234/18614	0.104972	0.431639	0.38742	CYFIP2/RP	16
GO:003571	BP	GO:003571	CD4-positi	9/902	115/18614	0.105935	0.435076	0.390505	ITCH/BCL3	9
GO:004519	BP	GO:004519	establishm	5/902	52/18614	0.106168	0.435076	0.390505	ARF4/IFT2	5
GO:004663	BP	GO:004663	positive reg	5/902	52/18614	0.106168	0.435076	0.390505	NKAP/IL4R	5
GO:200067	BP	GO:200067	regulation	5/902	52/18614	0.106168	0.435076	0.390505	ZNF675/SO	5
GO:004592	BP	GO:004592	negative re	17/902	252/18614	0.106199	0.435076	0.390505	GREM1/GD	17
GO:009730	BP	GO:009730	response t	17/902	252/18614	0.106199	0.435076	0.390505	CCND1/AH	17
GO:200124	BP	GO:200124	negative re	8/902	99/18614	0.107239	0.439019	0.394044	SYVN1/BID	8
GO:014002	BP	GO:014002	exocytic pr	7/902	83/18614	0.107292	0.439019	0.394044	BLOC1S6/	7
GO:190303	BP	GO:190303	regulation	24/902	378/18614	0.107785	0.439086	0.394104	IRF1/ITCH/	24
GO:000704	BP	GO:000704	lysosomal	10/902	132/18614	0.107789	0.439086	0.394104	CHMP5/PL	10
GO:000232	BP	GO:000232	lymphoid	3/902	24/18614	0.107966	0.439086	0.394104	SOX4/ZNH	3
GO:000916	BP	GO:000916	deoxyribor	3/902	24/18614	0.107966	0.439086	0.394104	ADK/TYMS	3
GO:005135	BP	GO:005135	negative re	3/902	24/18614	0.107966	0.439086	0.394104	NPR3/GRM	3
GO:006090	BP	GO:006090	positive reg	3/902	24/18614	0.107966	0.439086	0.394104	MYD88/LIL	3
GO:007220	BP	GO:007220	cell differe	3/902	24/18614	0.107966	0.439086	0.394104	GREM1/ST	3
GO:007257	BP	GO:007257	liver morph	3/902	24/18614	0.107966	0.439086	0.394104	TNFAIP3/P	3
GO:012025	BP	GO:012025	olefinic co	3/902	24/18614	0.107966	0.439086	0.394104	STARD3/D	3
GO:190241	BP	GO:190241	mitotic cyt	3/902	24/18614	0.107966	0.439086	0.394104	CHMP5/MI	3
GO:004340	BP	GO:004340	negative re	13/902	183/18614	0.108316	0.440242	0.395141	EMILIN1/IT	13
GO:005116	BP	GO:005116	nuclear exp	12/902	166/18614	0.108746	0.440593	0.395456	PHAX/SIRT	12
GO:009013	BP	GO:009013	tissue migr	24/902	379/18614	0.110083	0.440593	0.395456	GREM1/M	24
GO:000181	BP	GO:000181	positive reg	30/902	489/18614	0.110194	0.440593	0.395456	GATA6/IRF	30
GO:000918	BP	GO:000918	ribonucleo	9/902	116/18614	0.110214	0.440593	0.395456	HTR2A/PG	9
GO:003200	BP	GO:003200	regulation	9/902	116/18614	0.110214	0.440593	0.395456	SESN3/DG	9
GO:004275	BP	GO:004275	regulation	9/902	116/18614	0.110214	0.440593	0.395456	PTGDS/NR	9
GO:004864	BP	GO:004864	negative re	9/902	116/18614	0.110214	0.440593	0.395456	GDF15/DU	9
GO:007134	BP	GO:007134	cellular res	9/902	116/18614	0.110214	0.440593	0.395456	IRF1/NR1H	9
GO:001074	BP	GO:001074	macropha	4/902	38/18614	0.110419	0.440593	0.395456	NR1H3/ST	4
GO:001474	BP	GO:001474	negative re	4/902	38/18614	0.110419	0.440593	0.395456	FOXP1/G6	4
GO:002195	BP	GO:002195	central ner	4/902	38/18614	0.110419	0.440593	0.395456	WDR47/CD	4
GO:003080	BP	GO:003080	regulation	4/902	38/18614	0.110419	0.440593	0.395456	PDK2/SNC	4
GO:003272	BP	GO:003272	positive reg	4/902	38/18614	0.110419	0.440593	0.395456	IRF1/ISG15	4
GO:004300	BP	GO:004300	Golgi to pl	4/902	38/18614	0.110419	0.440593	0.395456	RAB31/ATF	4
GO:005108	BP	GO:005108	'de novo' p	4/902	38/18614	0.110419	0.440593	0.395456	DNAJB14/	4
GO:007252	BP	GO:007252	pyrimidine	4/902	38/18614	0.110419	0.440593	0.395456	NTHL1/ME	4
GO:190037	BP	GO:190037	regulation	4/902	38/18614	0.110419	0.440593	0.395456	PDK2/SNC	4
GO:000690	BP	GO:000690	phagocyto	16/902	236/18614	0.110824	0.440593	0.395456	RAB31/NR	16
GO:000028	BP	GO:000028	nuclear-tra	6/902	68/18614	0.111025	0.440593	0.395456	ZFP36L1/T	6
GO:004866	BP	GO:004866	neuron fate	6/902	68/18614	0.111025	0.440593	0.395456	ID2/NOTC	6
GO:005105	BP	GO:005105	positive reg	6/902	68/18614	0.111025	0.440593	0.395456	MAP4K4/L	6

GO:014048	MF	GO:014048	ABC-type	1/1068	1/18369	0.058141	0.365975	0.359538	ABCB7	1
GO:190557	MF	GO:190557	ganglioside	1/1068	1/18369	0.058141	0.365975	0.359538	PSAP	1
GO:190557	MF	GO:190557	ganglioside	1/1068	1/18369	0.058141	0.365975	0.359538	PSAP	1
GO:190557	MF	GO:190557	ganglioside	1/1068	1/18369	0.058141	0.365975	0.359538	PSAP	1
GO:199093	MF	GO:199093	mRNA N1-	1/1068	1/18369	0.058141	0.365975	0.359538	ALKBH3	1
GO:007234	MF	GO:007234	modified a	9/1068	85/18369	0.058203	0.365975	0.359538	AXL/ASAP1	9
GO:001687	MF	GO:001687	ligase activ	6/1068	48/18369	0.058336	0.365975	0.359538	PAICS/PFA	6
GO:000391	MF	GO:000391	DNA topoi	2/1068	7/18369	0.058344	0.365975	0.359538	TOP1MT/S	2
GO:000399	MF	GO:000399	acyl-CoA c	2/1068	7/18369	0.058344	0.365975	0.359538	ACADL/AC	2
GO:000412	MF	GO:000412	cytochrom	2/1068	7/18369	0.058344	0.365975	0.359538	CYB5R2/CY	2
GO:000814	MF	GO:000814	oxysterol b	2/1068	7/18369	0.058344	0.365975	0.359538	RORC/INSI	2
GO:001522	MF	GO:001522	choline tra	2/1068	7/18369	0.058344	0.365975	0.359538	SLC44A4/S	2
GO:001592	MF	GO:001592	galactosida	2/1068	7/18369	0.058344	0.365975	0.359538	GLB1L2/NA	2
GO:001684	MF	GO:001684	amidine-ly	2/1068	7/18369	0.058344	0.365975	0.359538	GGACT/AS	2
GO:001688	MF	GO:001688	ligase activ	2/1068	7/18369	0.058344	0.365975	0.359538	PC/MCCC2	2
GO:003170	MF	GO:003170	angiotensi	2/1068	7/18369	0.058344	0.365975	0.359538	JAK2/AGT	2
GO:003500	MF	GO:003500	1-phospha	2/1068	7/18369	0.058344	0.365975	0.359538	PIK3C2G/P	2
GO:004697	MF	GO:004697	TAP bindin	2/1068	7/18369	0.058344	0.365975	0.359538	HLA-B/HLA	2
GO:001662	MF	GO:001662	oxidoreduc	7/1068	60/18369	0.058361	0.365975	0.359538	ACADL/BLV	7
GO:001624	MF	GO:001624	channel re	14/1068	152/18369	0.0594	0.371017	0.364492	VAMP8/GP	14
GO:000816	MF	GO:000816	methyltran	18/1068	208/18369	0.059752	0.37174	0.365202	SETD4/SM	18
GO:000196	MF	GO:000196	G-protein	4/1068	26/18369	0.061264	0.378383	0.371728	NUCB1/LP	4
GO:004216	MF	GO:004216	telomeric D	5/1068	37/18369	0.061361	0.378383	0.371728	NCL/BLM/	5
GO:000817	MF	GO:000817	N-methyltr	10/1068	99/18369	0.061792	0.378383	0.371728	SETD4/SM	10
GO:000469	MF	GO:000469	calcium-de	3/1068	16/18369	0.062263	0.378383	0.371728	PRKD1/PKN	3
GO:001664	MF	GO:001664	oxidoreduc	3/1068	16/18369	0.062263	0.378383	0.371728	LOXL2/LOX	3
GO:007040	MF	GO:007040	NAD+ bind	3/1068	16/18369	0.062263	0.378383	0.371728	ALDH1A3/	3
GO:001676	MF	GO:001676	transferase	7/1068	61/18369	0.062776	0.379454	0.37278	GSTZ1/GST	7
GO:000809	MF	GO:000809	ATP-depen	12/1068	126/18369	0.062921	0.379454	0.37278	RUVBL2/H	12
GO:004830	MF	GO:004830	calcium-de	7/1068	62/18369	0.067389	0.403309	0.396215	VPS37B/AN	7
GO:009863	MF	GO:009863	cell adhesi	7/1068	62/18369	0.067389	0.403309	0.396215	ROBO3/PA	7
GO:000412	MF	GO:000412	cytochrom	3/1068	17/18369	0.072452	0.41619	0.40887	COX6B1/C	3
GO:000469	MF	GO:000469	protein kin	3/1068	17/18369	0.072452	0.41619	0.40887	PRKD1/PKN	3
GO:001629	MF	GO:001629	palmitoyl-	3/1068	17/18369	0.072452	0.41619	0.40887	PPT1/ACOT	3
GO:004260	MF	GO:004260	peptide an	5/1068	39/18369	0.073819	0.41619	0.40887	HLA-B/SLC	5
GO:000374	MF	GO:000374	translation	6/1068	51/18369	0.074143	0.41619	0.40887	EIF3D/EIF2	6
GO:009863	MF	GO:009863	cell-cell ad	6/1068	51/18369	0.074143	0.41619	0.40887	ROBO3/PA	6
GO:000175	MF	GO:000175	retinal deh	2/1068	8/18369	0.074859	0.41619	0.40887	ALDH8A1/	2
GO:000188	MF	GO:000188	nucleoside	2/1068	8/18369	0.074859	0.41619	0.40887	OARD1/CD	2
GO:000459	MF	GO:000459	peptide alp	2/1068	8/18369	0.074859	0.41619	0.40887	NAA30/NA	2
GO:000825	MF	GO:000825	tRNA-spec	2/1068	8/18369	0.074859	0.41619	0.40887	ADAT1/AD	2
GO:001643	MF	GO:001643	rRNA (ade	2/1068	8/18369	0.074859	0.41619	0.40887	DIMT1/ME	2
GO:001979	MF	GO:001979	procollage	2/1068	8/18369	0.074859	0.41619	0.40887	P4HA3/P4H	2
GO:003154	MF	GO:003154	peptidyl-p	2/1068	8/18369	0.074859	0.41619	0.40887	P4HA3/P4H	2
GO:200106	MF	GO:200106	glycogen b	2/1068	8/18369	0.074859	0.41619	0.40887	PPP1R3C/P	2
GO:001662	MF	GO:001662	oxidoreduc	4/1068	28/18369	0.076678	0.41619	0.40887	BLVRB/TM	4
GO:004354	MF	GO:004354	phosphatic	4/1068	28/18369	0.076678	0.41619	0.40887	AXL/JAK2/	4
GO:003509	MF	GO:003509	phosphatic	22/1068	275/18369	0.080839	0.41619	0.40887	ESYT3/ASA	22
GO:000006	MF	GO:000006	fatty-acyl-	3/1068	18/18369	0.08333	0.41619	0.40887	ACADL/AC	3
GO:001565	MF	GO:001565	quaternary	3/1068	18/18369	0.08333	0.41619	0.40887	SLC44A4/S	3
GO:001667	MF	GO:001667	oxidoreduc	3/1068	18/18369	0.08333	0.41619	0.40887	COX6B1/C	3
GO:009864	MF	GO:009864	cadherin b	3/1068	18/18369	0.08333	0.41619	0.40887	BAIAP2L1/	3
GO:000827	MF	GO:000827	protein me	9/1068	92/18369	0.086458	0.41619	0.40887	SETD4/SM	9
GO:003002	MF	GO:003002	extracellula	5/1068	41/18369	0.087546	0.41619	0.40887	COL6A2/C	5
GO:000368	MF	GO:000368	DNA clamp	2/1068	9/18369	0.092636	0.41619	0.40887	RFC4/CHTI	2
GO:000409	MF	GO:000409	carbonyl re	2/1068	9/18369	0.092636	0.41619	0.40887	DCXR/DHR	2
GO:000522	MF	GO:000522	volume-se	2/1068	9/18369	0.092636	0.41619	0.40887	TTYH2/LRR	2
GO:000552	MF	GO:000552	tropomyos	2/1068	9/18369	0.092636	0.41619	0.40887	S100A6/NE	2
GO:001617	MF	GO:001617	NAD(P)H c	2/1068	9/18369	0.092636	0.41619	0.40887	FMO5/DUO	2
GO:001662	MF	GO:001662	oxidoreduc	2/1068	9/18369	0.092636	0.41619	0.40887	OGDH/PDH	2
GO:001920	MF	GO:001920	nucleoside	2/1068	9/18369	0.092636	0.41619	0.40887	TK2/UCK2	2
GO:003235	MF	GO:003235	oxidized pu	2/1068	9/18369	0.092636	0.41619	0.40887	BLM/MUTY	2
GO:004279	MF	GO:004279	histone me	2/1068	9/18369	0.092636	0.41619	0.40887	SETD4/NSI	2
GO:007099	MF	GO:007099	snRNP bind	2/1068	9/18369	0.092636	0.41619	0.40887	SNRPD1/SI	2
GO:001674	MF	GO:001674	transferase	18/1068	221/18369	0.093624	0.41619	0.40887	SETD4/SM	18
GO:000518	MF	GO:000518	neuropepti	4/1068	30/18369	0.093835	0.41619	0.40887	OXT/CCK/	4
GO:000033	MF	GO:000033	RNA cap b	3/1068	19/18369	0.094857	0.41619	0.40887	EIF3D/CYF	3
GO:004228	MF	GO:004228	MHC class	3/1068	19/18369	0.094857	0.41619	0.40887	CD8B/PILR	3
GO:004280	MF	GO:004280	histone me	3/1068	19/18369	0.094857	0.41619	0.40887	SETD4/SM	3
GO:005142	MF	GO:005142	peptide ho	3/1068	19/18369	0.094857	0.41619	0.40887	JAK2/CCK/	3
GO:190156	MF	GO:190156	fatty acid c	3/1068	19/18369	0.094857	0.41619	0.40887	ACADL/AC	3
GO:000830	MF	GO:000830	structural c	5/1068	42/18369	0.094873	0.41619	0.40887	CAPN3/KR	5
GO:004313	MF	GO:004313	ubiquitin b	9/1068	94/18369	0.095804	0.41619	0.40887	FAF1/NSFL	9
GO:000451	MF	GO:000451	endonucle	11/1068	122/18369	0.098109	0.41619	0.40887	RCL1/KHN	11
GO:004815	MF	GO:004815	tau protein	5/1068	43/18369	0.102502	0.41619	0.40887	DCTN1/AA	5
GO:004342	MF	GO:004342	bHLH trans	4/1068	31/18369	0.103036	0.41619	0.40887	SOX9/HAN	4
GO:003017	MF	GO:003017	pyridoxal p	6/1068	56/18369	0.105349	0.41619	0.40887	SPTLC2/GL	6
GO:001650	MF	GO:001650	protein-ho	3/1068	20/18369	0.106993	0.41619	0.40887	LGR6/PAX8	3
GO:003141	MF	GO:003141	L-ascorbic	3/1068	20/18369	0.106993	0.41619	0.40887	ALKBH3/P4	3
GO:007030	MF	GO:007030	phosphatic	3/1068	20/18369	0.106993	0.41619	0.40887	PACSIN2/C	3
GO:003802	MF	GO:003802	cargo rece	8/1068	83/18369	0.108961	0.41619	0.40887	LOXL2/CXC	8
GO:001702	MF	GO:001702	myosin bin	7/1068	70/18369	0.11138	0.41619	0.40887	AXL/SHRO	7
GO:000109	MF	GO:000109	TFIID-class	2/1068	10/18369	0.111475	0.41619	0.40887	FBL/RUVBL	2
GO:000809	MF	GO:000809	5S rRNA bi	2/1068	10/18369	0.111475	0.41619	0.40887	RRS1/RPF2	2
GO:000864	MF	GO:000864	ubiquitin-li	2/1068	10/18369	0.111475	0.41619	0.40887	SAE1/UBA	2
GO:001527	MF	GO:001527	store-oper	2/1068	10/18369	0.111475	0.41619	0.40887	ORAI1/TRP	2
GO:001630	MF	GO:001630	1-phospha	2/1068	10/18369	0.111475	0.41619	0.40887	PIK3C2G/P	2
GO:001663	MF	GO:001663	oxidoreduc	2/1068	10/18369	0.111475	0.41619	0.40887	ACADL/AC	2
GO:001681	MF	GO:001681	hydrolase	2/1068	10/18369	0.111475	0.41619	0.40887	DDAH1/DD	2
GO:001688	MF	GO:001688	carbon-nit	2/1068	10/18369	0.111475	0.41619	0.40887	PFAS/GAT	2
GO:003205	MF	GO:003205	clathrin he	2/1068	10/18369	0.111475	0.41619	0.40887	LDLR/SNA	2
GO:003235	MF	GO:003235	oxidized D	2/1068	10/18369	0.111475	0.41619	0.40887	BLM/MUTY	2
GO:003320	MF	GO:003320	ribonuclea	2/1068	10/18369	0.111475	0.41619	0.40887	POP7/POP	2
GO:009859	MF	GO:009859	palmitoyl h	2/1068	10/18369	0.111475	0.41619	0.40887	PPT1/PPT2	2
GO:199024	MF	GO:199024	N6-methyl	2/1068	10/18369	0.111475	0.41619	0.40887	YTHDF2/YT	2
GO:007027	MF	GO:007027	vitamin B6	6/1068	57/18369	0.112297	0.41619	0.40887	SPTLC2/GL	6
GO:000021	MF	GO:000021	tRNA 2'-ph	1/1068	2/18369	0.112905	0.41619	0.40887	NT5C3A	1
GO:000070	MF	GO:000070	purine-spe	1/1068	2/18369	0.112905	0.41619	0.40887	MUTYH	1
GO:000173	MF	GO:000173	mRNA (N6	1/1068	2/18369	0.112905	0.41619	0.40887	METTL16	1
GO:000188	MF	GO:000188	purine nucl	1/1068	2/18369	0.112905	0.41619	0.40887	OARD1	1
GO:000213	MF	GO:000213	UTP bindin	1/1068	2/18369	0.112905	0.41619	0.40887	MTPAP	1
GO:000383	MF	GO:000383	beta-galac	1/1068	2/18369	0.112905	0.41619	0.40887	ST6GAL1	1
GO:000388	MF	GO:000388	protein C-t	1/1068	2/18369	0.112905	0.41619	0.40887	LCMT1	1
GO:000392	MF	GO:000392	GMP reduc	1/1068	2/18369	0.112905	0.41619	0.40887	GMPR	1
GO:000392	MF	GO:000392	GPI-ancho	1/1068	2/18369	0.112905	0.41619	0.40887	PIGK	1
GO:000404	MF	GO:000404	amidase ac	1/1068	2/18369	0.112905	0.41619	0.40887	FAAH	1
GO:000406	MF	GO:000406	asparagina	1/1068	2/18369	0.112905	0.41619	0.40887	ASRGL1	1
GO:000407	MF	GO:000407	biliverdin r	1/1068	2/18369	0.112905	0.41619	0.40887	BLVRB	1
GO:000408	MF	GO:000408	branched-	1/1068	2/18369	0.112905	0.41619	0.40887	BCAT2	1
GO:000410	MF	GO:000410	choline-ph	1/1068	2/18369	0.112905	0.41619	0.40887	PCYT1B	1
GO:000413	MF	GO:000413	deoxycytid	1/1068	2/18369	0.112905	0.41619	0.40887	TK2	1
GO:000416	MF	GO:000416	dimethylall	1/1068	2/18369	0.112905	0.41619	0.40887	FDPS	1
GO:000433	MF	GO:000433	geranyltrar	1/1068	2/18369	0.112905	0.41619	0.40887	FDPS	1
GO:000442	MF	GO:000442	hydroxyme	1/1068	2/18369	0.112905	0.41619	0.40887	HMGCS1	1
GO:000448	MF	GO:000448	methylcrot	1/1068	2/18369	0.112905	0.41619	0.40887	MCCC2	1
GO:000455	MF	GO:000455	alpha-gala	1/1068	2/18369	0.112905	0.41619	0.40887	NAGA	1
GO:000456	MF	GO:000456	alpha-L-fu	1/1068	2/18369	0.112905	0.41619	0.40887	FUCA2	1
GO:000474	MF	GO:000474	pyruvate ki	1/1068	2/18369	0.112905	0.41619	0.40887	PKM	1
GO:000479	MF	GO:000479	thymidine	1/1068	2/18369	0.112905	0.41619	0.40887	TK2	1
GO:000483	MF	GO:000483	tyrosine-tR	1/1068	2/18369	0.112905	0.41619	0.40887	YARS2	1
GO:000491	MF	GO:000491	interleukin-	1/1068	2/18369	0.112905	0.41619	0.40887	IL6R	1
GO:000499	MF	GO:000499	thyroid-sti	1/1068	2/18369	0.112905	0.41619	0.40887	PAX8	1
GO:000518	MF	GO:000518	neurohypo	1/1068	2/18369	0.112905	0.41619	0.40887	OXT	1
GO:000523	MF	GO:000523	extracellula	1/1068	2/18369	0.112905	0.41619	0.40887	GRIK2	1
GO:000829	MF	GO:000829	calcium- a	1/1068	2/18369	0.112905	0.41619	0.40887	ADCY1	1
GO:000846	MF	GO:000846	glycogenin	1/1068	2/18369	0.112905	0.41619	0.40887	GYG1	1
GO:000851	MF	GO:000851	secondary	1/1068	2/18369	0.112905	0.41619	0.40887	SLC22A4	1
GO:000879	MF	GO:000879	beta-aspa	1/1068	2/18369	0.112905	0.41619	0.40887	ASRGL1	1
GO:001592	MF	GO:001592	fucosidase	1/1068	2/18369	0.112905	0.41619	0.40887	FUCA2	1
GO:001688	MF	GO:001688	acid-amm	1/1068	2/18369	0.112905	0.41619	0.40887	DPH6	1
GO:001915	MF	GO:001915	mannokina	1/1068	2/18369	0.112905	0.41619	0.40887	GCK	1
GO:001994	MF	GO:001994	SUMO acti	1/1068	2/18369	0.112905	0.41619	0.40887	SAE1	1
GO:001998	MF	GO:001998	interleukin-	1/1068	2/18369	0.112905	0.41619	0.40887	IL6R	1
GO:003030	MF	GO:003030	heparanase	1/1068	2/18369	0.112905	0.41619	0.40887	HPSE	1
GO:003035	MF	GO:003035	melanin-co	1/1068	2/18369	0.112905	0.41619	0.40887	PMCHL2	1
GO:003062	MF	GO:003062	U12 snRNA	1/1068	2/18369	0.112905	0.41619	0.40887	RNPC3	1
GO:003062	MF	GO:003062	U6 snRNA	1/1068	2/18369	0.112905	0.41619	0.40887	METTL16	1
GO:003171	MF	GO:003171	calcitonin r	1/1068	2/18369	0.112905	0.41619	0.40887	CALCB	1
GO:003175	MF	GO:003175	D5 dopam	1/1068	2/18369	0.112905	0.41619	0.40887	GNA13	1
GO:003177	MF	GO:003177	melanin-co	1/1068	2/18369	0.112905	0.41619	0.40887	PMCHL2	1
GO:003177	MF	GO:003177	type 1 mel	1/1068	2/18369	0.112905	0.41619	0.40887	PMCHL2	1
GO:003187	MF	GO:003187	thromboxa	1/1068	2/18369	0.112905	0.41619	0.40887	RPGRIP1L	1
GO:003202	MF	GO:003202	myosin hea	1/1068	2/18369	0.112905	0.41619	0.40887	LIMCH1	1
GO:003385	MF	GO:003385	diphospho	1/1068	2/18369	0.112905	0.41619	0.40887	PPIP5K1	1
GO:003403	MF	GO:003403	8-oxo-7,8-	1/1068	2/18369	0.112905	0.41619	0.40887	MUTYH	1
GO:003540	MF	GO:003540	histone tyr	1/1068	2/18369	0.112905	0.41619	0.40887	JAK2	1
GO:003622	MF	GO:003622	UTP dipho	1/1068	2/18369	0.112905	0.41619	0.40887	ASMTL	1
GO:003806	MF	GO:003806	protein tyr	1/1068	2/18369	0.112905	0.41619	0.40887	DDR1	1
GO:004201	MF	GO:004201	interleukin-	1/1068	2/18369	0.112905	0.41619	0.40887	IL22RA2	1
GO:004228	MF	GO:004228	sphingolipi	1/1068	2/18369	0.112905	0.41619	0.40887	DEGS2	1
GO:004232	MF	GO:004232	heparan su	1/1068	2/18369	0.112905	0.41619	0.40887	EXT2	1
GO:004471	MF	GO:004471	8-oxo-GD	1/1068	2/18369	0.112905	0.41619	0.40887	NUDT18	1
GO:004513	MF	GO:004513	keratan sul	1/1068	2/18369	0.112905	0.41619	0.40887	CHST1	1
GO:004517	MF	GO:004517	glutathione	1/1068	2/18369	0.112905	0.41619	0.40887	GSTO2	1
GO:004685	MF	GO:004685	oxidoreduc	1/1068	2/18369	0.112905	0.41619	0.40887	GMPR	1
GO:004775	MF	GO:004775	cholesteno	1/1068	2/18369	0.112905	0.41619	0.40887	EBP	1
GO:005004	MF	GO:005004	L-leucine:2	1/1068	2/18369	0.112905	0.41619	0.40887	BCAT2	1
GO:005019	MF	GO:005019	[phosphory	1/1068	2/18369	0.112905	0.41619	0.40887	PPP1R3B	1
GO:005048	MF	GO:005048	GMP 5'-nu	1/1068	2/18369	0.112905	0.41619	0.40887	NT5C2	1
GO:005050	MF	GO:005050	N-acetylgl	1/1068	2/18369	0.112905	0.41619	0.40887	EXT2	1
GO:005061	MF	GO:005061	methylarsc	1/1068	2/18369	0.112905	0.41619	0.40887	GSTO2	1
GO:005061	MF	GO:005061	delta14-ste	1/1068	2/18369	0.112905	0.41619	0.40887	TM7SF2	1
GO:005149	MF	GO:005149	D-aminoac	1/1068	2/18369	0.112905	0.41619	0.40887	DTD1	1
GO:005150	MF	GO:005150	D-tyrosyl-	1/1068	2/18369	0.112905	0.41619	0.40887	DTD1	1
GO:005259	MF	GO:005259	tryptamine	1/1068	2/18369	0.112905	0.41619	0.40887	AOC3	1
GO:005265	MF	GO:005265	L-leucine t	1/1068	2/18369	0.112905	0.41619	0.40887	BCAT2	1
GO:005265	MF	GO:005265	L-valine tra	1/1068	2/18369	0.112905	0.41619	0.40887	BCAT2	1
GO:005265	MF	GO:005265	L-isoleucin	1/1068	2/18369	0.112905	0.41619	0.40887	BCAT2	1
GO:005271	MF	GO:005271	tRNA-spec	1/1068	2/18369	0.112905	0.41619	0.40887	ADAT2	1
GO:005290	MF	GO:005290	spermine:c	1/1068	2/18369	0.112905	0.41619	0.40887	SMOX	1
GO:006146	MF	GO:006146	O-acetyl-A	1/1068	2/18369	0.112905	0.41619	0.40887	OARD1	1
GO:006174	MF	GO:006174	forked DNA	1/1068	2/18369	0.112905	0.41619	0.40887	BLM	1
GO:006176	MF	GO:006176	magnesium	1/1068	2/18369	0.112905	0.41619	0.40887	SLC41A3	1
GO:006184	MF	GO:006184	telomeric C	1/1068	2/18369	0.112905	0.41619	0.40887	BLM	1
GO:007069	MF	GO:007069	type II acti	1/1068	2/18369	0.112905	0.41619	0.40887	MAGI2	1
GO:007097	MF	GO:007097	FHA doma	1/1068	2/18369	0.112905	0.41619	0.40887	XRCC4	1
GO:007235	MF	GO:007235	histone kin	1/1068	2/18369	0.112905	0.41619	0.40887	VRK1	1
GO:007254	MF	GO:007254	tyrosine bi	1/1068	2/18369	0.112905	0.41619	0.40887	YARS2	1
GO:009042	MF	GO:009042	thiamine p	1/1068	2/18369	0.112905	0.41619	0.40887	SLC44A4	1
GO:009048	MF	GO:009048	small RNA	1/1068	2/18369	0.112905	0.41619	0.40887	BCDIN3D	1
GO:009863	MF	GO:009863	collagen fil	1/1068	2/18369	0.112905	0.41619	0.40887	CHADL	1
GO:009880	MF	GO:009880	mRNA cap	1/1068	2/18369	0.112905	0.41619	0.40887	EIF3D	1
GO:009957	MF	GO:009957	G protein-	1/1068	2/18369	0.112905	0.41619	0.40887	GABBR1	1
GO:010275	MF	GO:010275	UDP-alpha	1/1068	2/18369	0.112905	0.41619	0.40887	GYG1	1
GO:010622	MF	GO:010622	peptide 2-	1/1068	2/18369	0.112905	0.41619	0.40887	KAT5	1
GO:010624	MF	GO:010624	L-serine-p	1/1068	2/18369	0.112905	0.41619	0.40887	PTDSS1	1
GO:010627	MF	GO:010627	biliberdin r	1/1068	2/18369	0.112905	0.41619	0.40887	BLVRB	1
GO:010627	MF	GO:010627	biliverdin r	1/1068	2/18369	0.112905	0.41619	0.40887	BLVRB	1
GO:010629	MF	GO:010629	NADH oxic	1/1068	2/18369	0.112905	0.41619	0.40887	DUOX1	1
GO:010634	MF	GO:010634	snRNA me	1/1068	2/18369	0.112905	0.41619	0.40887	METTL16	1
GO:012022	MF	GO:012022	coenzyme	1/1068	2/18369	0.112905	0.41619	0.40887	ACAT1	1
GO:014006	MF	GO:014006	peptide cr	1/1068	2/18369	0.112905	0.41619	0.40887	KAT5	1
GO:014006	MF	GO:014006	peptide bu	1/1068	2/18369	0.112905	0.41619	0.40887	KAT5	1
GO:199025	MF	GO:199025	histone-glu	1/1068	2/18369	0.112905	0.41619	0.40887	FBL	1
GO:199088	MF	GO:199088	H4K20me3	1/1068	2/18369	0.112905	0.41619	0.40887	PWP1	1
GO:001920	MF	GO:001920	carbohydra	3/1068	21/18369	0.119694	0.439159	0.431435	GCK/FGGY	3
GO:005101	MF	GO:005101	microtubul	3/1068	21/18369	0.119694	0.439159	0.431435	CLIP2/PAF	3
GO:003140	MF	GO:003140	carboxylic	16/1068	200/18369	0.122083	0.446881	0.439021	ALKBH3/PI	16
GO:014009	MF	GO:014009	catalytic ac	19/1068	245/18369	0.123189	0.449884	0.441971	RUVBL2/A	19
GO:003218	MF	GO:003218	ubiquitin-li	10/1068	114/18369	0.12675	0.453422	0.445447	FAF1/NSFL	10
GO:003293	MF	GO:003293	sterol bind	6/1068	59/18369	0.126867	0.453422	0.445447	RORC/TSP	6
GO:004432	MF	GO:004432	transmemb	11/1068	129/18369	0.130528	0.453422	0.445447	GPD1L/AN	11
GO:000216	MF	GO:000216	dystroglyca	2/1068	11/18369	0.131192	0.453422	0.445447	AGR3/PLE	2
GO:000401	MF	GO:000401	adenylate	2/1068	11/18369	0.131192	0.453422	0.445447	ADCY3/AD	2
GO:000524	MF	GO:000524	voltage-ga	2/1068	11/18369	0.131192	0.453422	0.445447	CLCN6/CL	2
GO:001666	MF	GO:001666	oxidoreduc	2/1068	11/18369	0.131192	0.453422	0.445447	CYB5R3/GI	2
GO:001667	MF	GO:001667	oxidoreduc	2/1068	11/18369	0.131192	0.453422	0.445447	SUMF1/QS	2
GO:003203	MF	GO:003203	myosin hea	2/1068	11/18369	0.131192	0.453422	0.445447	AXL/LIMCH	2
GO:007041	MF	GO:007041	co-SMAD	2/1068	11/18369	0.131192	0.453422	0.445447	PAX6/CITE	2
GO:007051	MF	GO:007051	death dom	2/1068	11/18369	0.131192	0.453422	0.445447	DAP/CRAD	2
GO:009952	MF	GO:009952	G protein-	2/1068	11/18369	0.131192	0.453422	0.445447	GABRB1/G	2
GO:199045	MF	GO:199045	transferrin	2/1068	11/18369	0.131192	0.453422	0.445447	RGMA/IKB	2
GO:001665	MF	GO:001665	oxidoreduc	8/1068	87/18369	0.132734	0.453422	0.445447	CYB5R2/C	8
GO:000460	MF	GO:000460	glutathione	3/1068	22/18369	0.132918	0.453422	0.445447	GSTZ1/GST	3
GO:001678	MF	GO:001678	phosphotra	3/1068	22/18369	0.132918	0.453422	0.445447	PIGO/PTDS	3
GO:003168	MF	GO:003168	G-protein	3/1068	22/18369	0.132918	0.453422	0.445447	GNG7/RAS	3
GO:004761	MF	GO:004761	acyl-CoA h	3/1068	22/18369	0.132918	0.453422	0.445447	PPT1/ACOT	3
GO:005032	MF	GO:005032	tau-protei	3/1068	22/18369	0.132918	0.453422	0.445447	CSNK1D/M	3
GO:012002	MF	GO:012002	cholesterol	3/1068	22/18369	0.132918	0.453422	0.445447	TSPO/STAR	3
GO:001683	MF	GO:001683	carboxy-ly	4/1068	34/18369	0.13294	0.453422	0.445447	PAICS/UXS	4
GO:001689	MF	GO:001689	endoribonu	4/1068	34/18369	0.13294	0.453422	0.445447	POP7/POP	4
GO:014010	MF	GO:014010	molecular	8/1068	88/18369	0.139061	0.453422	0.445447	EMC10/PW	8
GO:000509	MF	GO:000509	GTPase act	21/1068	280/18369	0.139575	0.453422	0.445447	TIAM2/ALS	21
GO:001681	MF	GO:001681	hydrolase a	11/1068	131/18369	0.14075	0.453422	0.445447	ASRGL1/N	11
GO:004302	MF	GO:004302	ribonucleo	13/1068	161/18369	0.144578	0.453422	0.445447	SNRPD1/E	13
GO:000504	MF	GO:000504	scavenger	5/1068	48/18369	0.144899	0.453422	0.445447	LOXL2/CXC	5
GO:001686	MF	GO:001686	intramolec	5/1068	48/18369	0.144899	0.453422	0.445447	EBP/PIGK/	5
GO:001690	MF	GO:001690	oxidoreduc	5/1068	48/18369	0.144899	0.453422	0.445447	FMO5/OGD	5
GO:000864	MF	GO:000864	rRNA meth	3/1068	23/18369	0.14662	0.453422	0.445447	FBL/DIMT1	3
GO:000993	MF	GO:000993	calcium-de	3/1068	23/18369	0.14662	0.453422	0.445447	PRKD1/PKN	3
GO:001711	MF	GO:001711	single-stra	3/1068	23/18369	0.14662	0.453422	0.445447	RFC4/MCM	3
GO:003503	MF	GO:003503	histone ace	3/1068	23/18369	0.14662	0.453422	0.445447	PAX6/SP1/	3
GO:004327	MF	GO:004327	phospholip	3/1068	23/18369	0.14662	0.453422	0.445447	BANK1/PA	3
GO:012001	MF	GO:012001	sterol trans	3/1068	23/18369	0.14662	0.453422	0.445447	TSPO/STAR	3
GO:000875	MF	GO:000875	S-adenosy	13/1068	162/18369	0.149377	0.453422	0.445447	SETD4/SM	13
GO:000451	MF	GO:000451	nuclease a	16/1068	207/18369	0.150353	0.453422	0.445447	RCL1/KHN	16
GO:000034	MF	GO:000034	RNA 7-me	2/1068	12/18369	0.151621	0.453422	0.445447	CYFIP1/EIF	2
GO:000399	MF	GO:000399	acyl-CoA c	2/1068	12/18369	0.151621	0.453422	0.445447	ACADL/GC	2
GO:000452	MF	GO:000452	ribonuclea	2/1068	12/18369	0.151621	0.453422	0.445447	POP7/POP	2
GO:000485	MF	GO:000485	phospholip	2/1068	12/18369	0.151621	0.453422	0.445447	ANXA2/AN	2
GO:001627	MF	GO:001627	arginine N-	2/1068	12/18369	0.151621	0.453422	0.445447	PRMT3/PR	2
GO:001627	MF	GO:001627	protein-arg	2/1068	12/18369	0.151621	0.453422	0.445447	PRMT3/PR	2
GO:003154	MF	GO:003154	peptidyl-p	2/1068	12/18369	0.151621	0.453422	0.445447	P4HA3/P4H	2
GO:003500	MF	GO:003500	phosphatic	2/1068	12/18369	0.151621	0.453422	0.445447	PIK3C2G/P	2
GO:004603	MF	GO:004603	inositol tris	2/1068	12/18369	0.151621	0.453422	0.445447	INPP1/INP	2
GO:005289	MF	GO:005289	oxidoreduc	2/1068	12/18369	0.151621	0.453422	0.445447	ACADL/GC	2
GO:007067	MF	GO:007067	inositol 1,4	2/1068	12/18369	0.151621	0.453422	0.445447	ITPR3/TRP	2
GO:009749	MF	GO:009749	structural r	2/1068	12/18369	0.151621	0.453422	0.445447	EMILIN3/A	2
GO:014071	MF	GO:014071	histone cha	2/1068	12/18369	0.151621	0.453422	0.445447	PWP1/VPS	2
GO:000372	MF	GO:000372	RNA helica	7/1068	76/18369	0.15226	0.453422	0.445447	AQR/DHX1	7
GO:001524	MF	GO:001524	sterol trans	4/1068	36/18369	0.154617	0.453422	0.445447	TSPO/STAR	4
GO:004353	MF	GO:004353	ADP bindin	4/1068	36/18369	0.154617	0.453422	0.445447	RUVBL2/M	4
GO:000370	MF	GO:000370	nuclear ste	3/1068	24/18369	0.160755	0.453422	0.445447	NR1D1/NR	3
GO:001085	MF	GO:001085	calcium-de	3/1068	24/18369	0.160755	0.453422	0.445447	PRKD1/PKN	3
GO:001510	MF	GO:001510	bicarbonat	3/1068	24/18369	0.160755	0.453422	0.445447	SLC4A2/SL	3
GO:014010	MF	GO:014010	catalytic ac	3/1068	24/18369	0.160755	0.453422	0.445447	FBL/DIMT1	3
GO:001802	MF	GO:001802	histone-lys	5/1068	50/18369	0.163669	0.453422	0.445447	SETD4/SM	5
GO:001687	MF	GO:001687	ligase activ	13/1068	165/18369	0.164271	0.453422	0.445447	FARS2/PAI	13
GO:000082	MF	GO:000082	inositol hex	1/1068	3/18369	0.164488	0.453422	0.445447	ITPR3	1
GO:000115	MF	GO:000115	TFIIIC-clas	1/1068	3/18369	0.164488	0.453422	0.445447	BDP1	1
GO:000206	MF	GO:000206	purine nucl	1/1068	3/18369	0.164488	0.453422	0.445447	PYGL	1
GO:000391	MF	GO:000391	DNA topoi	1/1068	3/18369	0.164488	0.453422	0.445447	SPO11	1
GO:000395	MF	GO:000395	NADPH-he	1/1068	3/18369	0.164488	0.453422	0.445447	MTRR	1
GO:000403	MF	GO:000403	aldehyde c	1/1068	3/18369	0.164488	0.453422	0.445447	FMO5	1
GO:000414	MF	GO:000414	diacylglyce	1/1068	3/18369	0.164488	0.453422	0.445447	CDS1	1
GO:000416	MF	GO:000416	delta(3)-de	1/1068	3/18369	0.164488	0.453422	0.445447	ECI1	1
GO:000431	MF	GO:000431	farnesyltra	1/1068	3/18369	0.164488	0.453422	0.445447	FDFT1	1
GO:000450	MF	GO:000450	dopamine	1/1068	3/18369	0.164488	0.453422	0.445447	MOXD1	1
GO:000450	MF	GO:000450	phenylalan	1/1068	3/18369	0.164488	0.453422	0.445447	PAH	1
GO:000456	MF	GO:000456	beta-glucu	1/1068	3/18369	0.164488	0.453422	0.445447	HPSE	1
GO:000459	MF	GO:000459	pantothena	1/1068	3/18369	0.164488	0.453422	0.445447	PANK1	1
GO:000460	MF	GO:000460	phosphatic	1/1068	3/18369	0.164488	0.453422	0.445447	CDS1	1
GO:00047[illegible]	MF	GO:00047[illegible]	pyruvate [illegible]	1/1068	3/18369	0.164488	0.453422	0.445447	PDHB	1
GO:00049[illegible]	MF	GO:00049[illegible]	interleukin-	1/1068	3/18369	0.164488	0.453422	0.445447	IL6R	1
GO:00049[illegible]	MF	GO:00049[illegible]	beta-adren	1/1068	3/18369	0.164488	0.453422	0.445447	ADRB2	1
GO:00049[illegible]	MF	GO:00049[illegible]	G protein-	1/1068	3/18369	0.164488	0.453422	0.445447	GABBR1	1
GO:00049[illegible]	MF	GO:00049[illegible]	melanocyt	1/1068	3/18369	0.164488	0.453422	0.445447	MC1R	1
GO:00052[illegible]	MF	GO:00052[illegible]	inositol 1,4	1/1068	3/18369	0.164488	0.453422	0.445447	ITPR3	1

GO:002154	BP	GO:002154	pallium de	13/902	184/18614	0.111695	0.440593	0.395456	SRGAP2/W	13
GO:004277	BP	GO:004277	signal trans	13/902	184/18614	0.111695	0.440593	0.395456	CCND1/RP	13
GO:000192	BP	GO:000192	positive re	2/902	12/18614	0.112263	0.440593	0.395456	RAB29/SN	2
GO:000206	BP	GO:000206	columnar/c	2/902	12/18614	0.112263	0.440593	0.395456	GDF11/TYM	2
GO:000255	BP	GO:000255	mast cell c	2/902	12/18614	0.112263	0.440593	0.395456	VEGFC/VE	2
GO:000316	BP	GO:000316	sinoatrial n	2/902	12/18614	0.112263	0.440593	0.395456	GATA6/SH	2
GO:000917	BP	GO:000917	pyrimidine	2/902	12/18614	0.112263	0.440593	0.395456	TYMS/TYM	2
GO:001063	BP	GO:001063	regulation	2/902	12/18614	0.112263	0.440593	0.395456	VAT1/OMA	2
GO:001485	BP	GO:001485	response t	2/902	12/18614	0.112263	0.440593	0.395456	PIK3CA/SC	2
GO:001578	BP	GO:001578	nucleotide	2/902	12/18614	0.112263	0.440593	0.395456	SLC35A3/S	2
GO:001932	BP	GO:001932	pentose m	2/902	12/18614	0.112263	0.440593	0.395456	G6PD/PGD	2
GO:003163	BP	GO:003163	regulation	2/902	12/18614	0.112263	0.440593	0.395456	CPLX2/RAB	2
GO:003413	BP	GO:003413	regulation	2/902	12/18614	0.112263	0.440593	0.395456	TNFAIP3/T	2
GO:003581	BP	GO:003581	positive re	2/902	12/18614	0.112263	0.440593	0.395456	NPR3/EDN	2
GO:003629	BP	GO:003629	cellular res	2/902	12/18614	0.112263	0.440593	0.395456	ATP6V0A2	2
GO:004241	BP	GO:004241	dopamine	2/902	12/18614	0.112263	0.440593	0.395456	TGFB2/SN	2
GO:004324	BP	GO:004324	proteasom	2/902	12/18614	0.112263	0.440593	0.395456	PSMD10/P	2
GO:004347	BP	GO:004347	pigment ac	2/902	12/18614	0.112263	0.440593	0.395456	BLOC1S6/	2
GO:004348	BP	GO:004348	cellular pig	2/902	12/18614	0.112263	0.440593	0.395456	BLOC1S6/	2
GO:004854	BP	GO:004854	regulation	2/902	12/18614	0.112263	0.440593	0.395456	NR1H3/CD	2
GO:005154	BP	GO:005154	positive re	2/902	12/18614	0.112263	0.440593	0.395456	MAP4K4/M	2
GO:005179	BP	GO:005179	positive re	2/902	12/18614	0.112263	0.440593	0.395456	TGFB2/TRA	2
GO:006039	BP	GO:006039	negative re	2/902	12/18614	0.112263	0.440593	0.395456	GREM1/EM	2
GO:007094	BP	GO:007094	neutrophil-	2/902	12/18614	0.112263	0.440593	0.395456	TREM1/MY	2
GO:007137	BP	GO:007137	cellular res	2/902	12/18614	0.112263	0.440593	0.395456	GCLM/GCL	2
GO:007147	BP	GO:007147	cellular res	2/902	12/18614	0.112263	0.440593	0.395456	ZFP36L1/S	2
GO:007218	BP	GO:007218	regulation	2/902	12/18614	0.112263	0.440593	0.395456	STAT1/CTN	2
GO:007271	BP	GO:007271	response t	2/902	12/18614	0.112263	0.440593	0.395456	SPIDR/TRE	2
GO:009030	BP	GO:009030	positive re	2/902	12/18614	0.112263	0.440593	0.395456	ZNF304/DI	2
GO:009043	BP	GO:009043	protein loc	2/902	12/18614	0.112263	0.440593	0.395456	SUMO1/TC	2
GO:009757	BP	GO:009757	vacuole fus	2/902	12/18614	0.112263	0.440593	0.395456	CHMP5/CH	2
GO:014005	BP	GO:014005	cellular res	2/902	12/18614	0.112263	0.440593	0.395456	CD68/MYD	2
GO:190163	BP	GO:190163	regulation	2/902	12/18614	0.112263	0.440593	0.395456	CPLX2/RAB	2
GO:190233	BP	GO:190233	regulation	2/902	12/18614	0.112263	0.440593	0.395456	TGFB2/HN	2
GO:190386	BP	GO:190386	extraembry	2/902	12/18614	0.112263	0.440593	0.395456	DNAJB6/TN	2
GO:190466	BP	GO:190466	negative re	2/902	12/18614	0.112263	0.440593	0.395456	BAG2/RPL	2
GO:200121	BP	GO:200121	positive re	2/902	12/18614	0.112263	0.440593	0.395456	RAP1A/RIN	2
GO:200102	BP	GO:200102	positive re	12/902	167/18614	0.112316	0.440593	0.395456	PMAIP1/EY	12
GO:190303	BP	GO:190303	negative re	11/902	150/18614	0.112416	0.440593	0.395456	IRF1/ITCH/	11
GO:000028	BP	GO:000028	mitotic cyt	7/902	84/18614	0.112455	0.440593	0.395456	SNX18/CH	7
GO:190121	BP	GO:190121	negative re	15/902	219/18614	0.112488	0.440593	0.395456	CCND1/NT	15
GO:001082	BP	GO:001082	negative re	5/902	53/18614	0.112829	0.440899	0.395731	VAT1/OMA	5
GO:004826	BP	GO:004826	positive re	5/902	53/18614	0.112829	0.440899	0.395731	GREM1/TF	5
GO:006007	BP	GO:006007	Wnt signali	5/902	53/18614	0.112829	0.440899	0.395731	RSPO3/FZD	5
GO:190571	BP	GO:190571	positive re	5/902	53/18614	0.112829	0.440899	0.395731	BID/SLC35	5
GO:000930	BP	GO:000930	amine met	9/902	117/18614	0.114587	0.447504	0.40166	TGFB2/SLC	9
GO:000865	BP	GO:000865	phospholip	17/902	255/18614	0.114772	0.447967	0.402075	SOCS6/MF	17
GO:001095	BP	GO:001095	positive re	13/902	185/18614	0.115136	0.449123	0.403113	CYFIP2/RP	13
GO:003249	BP	GO:003249	response t	22/902	345/18614	0.115613	0.450723	0.404549	LY96/DUSP	22
GO:000704	BP	GO:000704	lysosome c	8/902	101/18614	0.116713	0.451993	0.405689	CHMP5/HC	8
GO:004870	BP	GO:004870	oligodendr	8/902	101/18614	0.116713	0.451993	0.405689	DUSP10/SC	8
GO:008017	BP	GO:008017	lytic vacuo	8/902	101/18614	0.116713	0.451993	0.405689	CHMP5/HC	8
GO:000931	BP	GO:000931	oligosacch	6/902	69/18614	0.116925	0.451993	0.405689	ST6GALNA	6
GO:000176	BP	GO:000176	morphoge	14/902	203/18614	0.117267	0.451993	0.405689	GREM1/RS	14
GO:000609	BP	GO:000609	glycolytic p	7/902	85/18614	0.117746	0.451993	0.405689	HTR2A/PG	7
GO:003139	BP	GO:003139	negative re	7/902	85/18614	0.117746	0.451993	0.405689	BAG2/SOX	7
GO:000314	BP	GO:000314	outflow tra	3/902	25/18614	0.118493	0.451993	0.405689	GATA6/TG	3
GO:001404	BP	GO:001404	glutamate	3/902	25/18614	0.118493	0.451993	0.405689	NTRK2/GR	3
GO:004265	BP	GO:004265	regulation	3/902	25/18614	0.118493	0.451993	0.405689	FZD7/RBB	3
GO:004315	BP	GO:004315	entrainmer	3/902	25/18614	0.118493	0.451993	0.405689	PPP1CB/ID	3
GO:005086	BP	GO:005086	negative re	3/902	25/18614	0.118493	0.451993	0.405689	DUSP3/PT	3
GO:007084	BP	GO:007084	inclusion b	3/902	25/18614	0.118493	0.451993	0.405689	DNAJB6/H	3
GO:007145	BP	GO:007145	cellular res	3/902	25/18614	0.118493	0.451993	0.405689	NQO1/SO	3
GO:007145	BP	GO:007145	cellular res	3/902	25/18614	0.118493	0.451993	0.405689	NQO1/SO	3
GO:007227	BP	GO:007227	metanephr	3/902	25/18614	0.118493	0.451993	0.405689	GREM1/ST	3
GO:009963	BP	GO:009963	protein loc	3/902	25/18614	0.118493	0.451993	0.405689	RAP1A/DL	3
GO:009964	BP	GO:009964	neurotrans	3/902	25/18614	0.118493	0.451993	0.405689	RAP1A/DL	3
GO:190107	BP	GO:190107	glucosamir	3/902	25/18614	0.118493	0.451993	0.405689	CHI3L1/EX	3
GO:200029	BP	GO:200029	regulation	3/902	25/18614	0.118493	0.451993	0.405689	GATA6/CT	3
GO:003085	BP	GO:003085	granulocyt	4/902	39/18614	0.118592	0.451993	0.405689	NKAP/LBR	4
GO:004230	BP	GO:004230	positive re	4/902	39/18614	0.118592	0.451993	0.405689	HSP90AA1	4
GO:004240	BP	GO:004240	cellular bio	4/902	39/18614	0.118592	0.451993	0.405689	TGFB2/SN	4
GO:004582	BP	GO:004582	positive re	4/902	39/18614	0.118592	0.451993	0.405689	TGFB2/HR	4
GO:006025	BP	GO:006025	regulation	4/902	39/18614	0.118592	0.451993	0.405689	VEGFC/SO	4
GO:003425	BP	GO:003425	positive re	12/902	169/18614	0.119658	0.451993	0.405689	RPS27L/CY	12
GO:009010	BP	GO:009010	negative re	12/902	169/18614	0.119658	0.451993	0.405689	GREM1/EM	12
GO:003085	BP	GO:003085	negative re	5/902	54/18614	0.119687	0.451993	0.405689	CCND1/HC	5
GO:003111	BP	GO:003111	regulation	5/902	54/18614	0.119687	0.451993	0.405689	SNCA/SLA	5
GO:009747	BP	GO:009747	synaptic ve	5/902	54/18614	0.119687	0.451993	0.405689	BLOC1S6/	5
GO:000189	BP	GO:000189	placenta d	11/902	152/18614	0.120202	0.451993	0.405689	ZFP36L1/R	11
GO:007132	BP	GO:007132	cellular res	11/902	152/18614	0.120202	0.451993	0.405689	ZFP36L1/Z	11
GO:007200	BP	GO:007200	nephron de	11/902	152/18614	0.120202	0.451993	0.405689	GREM1/IRX	11
GO:190331	BP	GO:190331	regulation	21/902	329/18614	0.121079	0.451993	0.405689	ZFP36L1/R	21
GO:000616	BP	GO:000616	nucleoside	8/902	102/18614	0.121613	0.451993	0.405689	HTR2A/PG	8
GO:004277	BP	GO:004277	ATP synthe	8/902	102/18614	0.121613	0.451993	0.405689	NDUFA5/IS	8
GO:004277	BP	GO:004277	mitochond	8/902	102/18614	0.121613	0.451993	0.405689	NDUFA5/IS	8
GO:199083	BP	GO:199083	cellular res	8/902	102/18614	0.121613	0.451993	0.405689	VEGFC/B3	8
GO:001081	BP	GO:001081	regulation	15/902	222/18614	0.122097	0.451993	0.405689	GREM1/EM	15
GO:004424	BP	GO:004424	cellular lipi	15/902	222/18614	0.122097	0.451993	0.405689	CYP1B1/M	15
GO:000606	BP	GO:000606	alcohol me	23/902	366/18614	0.122724	0.451993	0.405689	GPD2/DPA	23
GO:004684	BP	GO:004684	filopodium	6/902	70/18614	0.122978	0.451993	0.405689	SRGAP2/C	6
GO:004848	BP	GO:004848	synaptic ve	6/902	70/18614	0.122978	0.451993	0.405689	SYT8/DGK	6
GO:005188	BP	GO:005188	regulation	6/902	70/18614	0.122978	0.451993	0.405689	PMAIP1/BI	6
GO:200037	BP	GO:200037	positive re	6/902	70/18614	0.122978	0.451993	0.405689	CYP1B1/CD	6
GO:000675	BP	GO:000675	ATP genera	7/902	86/18614	0.123163	0.451993	0.405689	HTR2A/PG	7
GO:001584	BP	GO:001584	monoamin	7/902	86/18614	0.123163	0.451993	0.405689	SDHD/SYT	7
GO:003307	BP	GO:003307	T cell differ	7/902	86/18614	0.123163	0.451993	0.405689	MAFB/ZFP	7
GO:003410	BP	GO:003410	regulation	7/902	86/18614	0.123163	0.451993	0.405689	GREM1/TN	7
GO:003027	BP	GO:003027	regulation	9/902	119/18614	0.123615	0.451993	0.405689	GREM1/SC	9
GO:001604	BP	GO:001604	lipid catab	21/902	330/18614	0.123755	0.451993	0.405689	CYP1B1/M	21
GO:000716	BP	GO:000716	negative re	20/902	312/18614	0.124056	0.451993	0.405689	MAP4K4/IF	20
GO:002240	BP	GO:002240	negative re	14/902	205/18614	0.124072	0.451993	0.405689	IRF1/ITCH/	14
GO:004671	BP	GO:004671	viral entry i	11/902	153/18614	0.124205	0.451993	0.405689	ITCH/TRIM	11
GO:007133	BP	GO:007133	cellular res	10/902	136/18614	0.124352	0.451993	0.405689	ZNF236/SC	10
GO:006113	BP	GO:006113	morphoge	13/902	188/18614	0.125824	0.451993	0.405689	GREM1/RS	13
GO:015011	BP	GO:015011	cell-substr	8/902	103/18614	0.12662	0.451993	0.405689	GREM1/MA	8
GO:199082	BP	GO:199082	response t	8/902	103/18614	0.12662	0.451993	0.405689	VEGFC/B3	8
GO:004337	BP	GO:004337	regulation	5/902	55/18614	0.126734	0.451993	0.405689	IL4R/JUNB	5
GO:006146	BP	GO:006146	protein loc	5/902	55/18614	0.126734	0.451993	0.405689	LAMP2/SO	5
GO:003294	BP	GO:003294	mononucle	20/902	313/18614	0.126852	0.451993	0.405689	IRF1/ITCH/	20
GO:000930	BP	GO:000930	amine bios	4/902	40/18614	0.127018	0.451993	0.405689	TGFB2/SN	4
GO:003086	BP	GO:003086	cortical act	4/902	40/18614	0.127018	0.451993	0.405689	DLG1/RAC	4
GO:004209	BP	GO:004209	type 2 imm	4/902	40/18614	0.127018	0.451993	0.405689	BCL3/IL4R	4
GO:190153	BP	GO:190153	regulation	4/902	40/18614	0.127018	0.451993	0.405689	ITCH/ZNH	4
GO:190352	BP	GO:190352	positive re	4/902	40/18614	0.127018	0.451993	0.405689	TGFB2/HR	4
GO:000275	BP	GO:000275	immune re	26/902	423/18614	0.127531	0.451993	0.405689	IRF1/ITCH/	26
GO:000700	BP	GO:000700	mitochond	9/902	120/18614	0.128269	0.451993	0.405689	PMAIP1/BI	9
GO:003032	BP	GO:003032	embryonic	9/902	120/18614	0.128269	0.451993	0.405689	GREM1/TG	9
GO:003511	BP	GO:003511	embryonic	9/902	120/18614	0.128269	0.451993	0.405689	GREM1/TG	9
GO:000168	BP	GO:000168	tRNA 5'-le	2/902	13/18614	0.128579	0.451993	0.405689	RPP25/SSB	2
GO:000309	BP	GO:000309	renal sodiu	2/902	13/18614	0.128579	0.451993	0.405689	EDNRB/SG	2
GO:000653	BP	GO:000653	cysteine m	2/902	13/18614	0.128579	0.451993	0.405689	GCLM/GCL	2
GO:000673	BP	GO:000673	NADH met	2/902	13/18614	0.128579	0.451993	0.405689	ME1/NUD	2
GO:000718	BP	GO:000718	SMAD prot	2/902	13/18614	0.128579	0.451993	0.405689	EID2/SMA	2
GO:001074	BP	GO:001074	negative re	2/902	13/18614	0.128579	0.451993	0.405689	NR1H3/ITC	2
GO:003296	BP	GO:003296	negative re	2/902	13/18614	0.128579	0.451993	0.405689	EMILIN1/C	2
GO:003433	BP	GO:003433	adherens ju	2/902	13/18614	0.128579	0.451993	0.405689	HIPK1/CTN	2
GO:003498	BP	GO:003498	mitochond	2/902	13/18614	0.128579	0.451993	0.405689	YME1L1/O	2
GO:003600	BP	GO:003600	cellular res	2/902	13/18614	0.128579	0.451993	0.405689	PTPN2/CSF	2
GO:003630	BP	GO:003630	atrioventric	2/902	13/18614	0.128579	0.451993	0.405689	GATA6/EN	2
GO:004211	BP	GO:004211	monocyte	2/902	13/18614	0.128579	0.451993	0.405689	FOXP1/CSF	2
GO:004255	BP	GO:004255	pteridine-c	2/902	13/18614	0.128579	0.451993	0.405689	SPR/PCBD	2
GO:004356	BP	GO:004356	positive re	2/902	13/18614	0.128579	0.451993	0.405689	IGFBP3/IGF	2
GO:006105	BP	GO:006105	negative re	2/902	13/18614	0.128579	0.451993	0.405689	FOXP1/G6I	2
GO:006143	BP	GO:006143	bone trabe	2/902	13/18614	0.128579	0.451993	0.405689	GREM1/CC	2
GO:006179	BP	GO:006179	Golgi lume	2/902	13/18614	0.128579	0.451993	0.405689	ATP6V0A2	2
GO:007049	BP	GO:007049	thrombin-a	2/902	13/18614	0.128579	0.451993	0.405689	DGKQ/SN	2
GO:007091	BP	GO:007091	UV-damag	2/902	13/18614	0.128579	0.451993	0.405689	TREX1/CUI	2
GO:007123	BP	GO:007123	cellular res	2/902	13/18614	0.128579	0.451993	0.405689	HSPA5/RPL	2
GO:190010	BP	GO:190010	positive re	2/902	13/18614	0.128579	0.451993	0.405689	PTPN2/XBP	2
GO:190326	BP	GO:190326	positive re	2/902	13/18614	0.128579	0.451993	0.405689	HSPA1B/LA	2
GO:190474	BP	GO:190474	regulation	2/902	13/18614	0.128579	0.451993	0.405689	TGFB2/HN	2
GO:190506	BP	GO:190506	negative re	2/902	13/18614	0.128579	0.451993	0.405689	FGF9/DNM	2
GO:190506	BP	GO:190506	positive re	2/902	13/18614	0.128579	0.451993	0.405689	ENG/SOD2	2
GO:200104	BP	GO:200104	positive re	2/902	13/18614	0.128579	0.451993	0.405689	LIMS1/CD6	2
GO:000208	BP	GO:000208	lens develo	7/902	87/18614	0.128706	0.451993	0.405689	SOX11/HIP	7
GO:003362	BP	GO:003362	cell adhesi	7/902	87/18614	0.128706	0.451993	0.405689	ITGBL1/EM	7
GO:000320	BP	GO:000320	cardiac ven	6/902	71/18614	0.129182	0.451993	0.405689	TGFB2/SO	6
GO:007026	BP	GO:007026	necrotic ce	6/902	71/18614	0.129182	0.451993	0.405689	TMEM123/	6
GO:000651	BP	GO:000651	protein deg	3/902	26/18614	0.129382	0.451993	0.405689	SYVN1/RN	3
GO:000692	BP	GO:000692	substrate-c	3/902	26/18614	0.129382	0.451993	0.405689	ITGA11/VE	3
GO:000727	BP	GO:000727	neuromusc	3/902	26/18614	0.129382	0.451993	0.405689	CHAT/RAB	3
GO:000943	BP	GO:000943	NAD biosy	3/902	26/18614	0.129382	0.451993	0.405689	NAPRT/NU	3
GO:001482	BP	GO:001482	vascular as	3/902	26/18614	0.129382	0.451993	0.405689	PIK3C2A/H	3
GO:003533	BP	GO:003533	regulation	3/902	26/18614	0.129382	0.451993	0.405689	SOX11/AR	3
GO:004434	BP	GO:004434	fibroblast a	3/902	26/18614	0.129382	0.451993	0.405689	BID/CASP7	3
GO:005123	BP	GO:005123	sequesterin	3/902	26/18614	0.129382	0.451993	0.405689	LMAN1/FT	3
GO:005189	BP	GO:005189	positive re	3/902	26/18614	0.129382	0.451993	0.405689	MAP4K4/LI	3
GO:006034	BP	GO:006034	trabecula f	3/902	26/18614	0.129382	0.451993	0.405689	GREM1/SL	3
GO:006057	BP	GO:006057	intestinal e	3/902	26/18614	0.129382	0.451993	0.405689	GATA6/PTI	3
GO:190342	BP	GO:190342	regulation	3/902	26/18614	0.129382	0.451993	0.405689	DGKQ/SN	3
GO:006082	BP	GO:006082	regulation	17/902	260/18614	0.129961	0.451993	0.405689	GREM1/RS	17
GO:007190	BP	GO:007190	regulation	23/902	369/18614	0.130483	0.451993	0.405689	TRIB3/CCN	23
GO:007145	BP	GO:007145	cellular res	12/902	172/18614	0.131171	0.451993	0.405689	GATA6/ZF	12
GO:004693	BP	GO:004693	nucleotide	8/902	104/18614	0.131732	0.451993	0.405689	HTR2A/PG	8
GO:001612	BP	GO:001612	sterol meta	11/902	155/18614	0.132432	0.451993	0.405689	STARD3/C	11
GO:003294	BP	GO:003294	regulation	16/902	243/18614	0.132823	0.451993	0.405689	IRF1/ITCH/	16
GO:007133	BP	GO:007133	cellular res	10/902	138/18614	0.133127	0.451993	0.405689	ZNF236/SC	10
GO:000195	BP	GO:000195	positive re	5/902	56/18614	0.133968	0.451993	0.405689	MAP4K4/LI	5
GO:000275	BP	GO:000275	cell surface	5/902	56/18614	0.133968	0.451993	0.405689	LY96/NR1H	5
GO:001936	BP	GO:001936	arachidoni	5/902	56/18614	0.133968	0.451993	0.405689	PTGDS/CY	5
GO:003508	BP	GO:003508	establishm	5/902	56/18614	0.133968	0.451993	0.405689	ARF4/IFT2	5
GO:004507	BP	GO:004507	negative re	5/902	56/18614	0.133968	0.451993	0.405689	OASL/ISG1	5
GO:006124	BP	GO:006124	establishm	5/902	56/18614	0.133968	0.451993	0.405689	ARF4/IFT2	5
GO:000639	BP	GO:000639	mRNA pro	30/902	500/18614	0.134003	0.451993	0.405689	ZFP36L1/R	30
GO:001610	BP	GO:001610	diterpenoi	7/902	88/18614	0.134372	0.451993	0.405689	CYP1B1/CY	7
GO:003111	BP	GO:003111	regulation	7/902	88/18614	0.134372	0.451993	0.405689	MAP1A/WI	7
GO:006056	BP	GO:006056	epithelial t	21/902	334/18614	0.134809	0.451993	0.405689	GREM1/IRX	21
GO:200124	BP	GO:200124	regulation	12/902	173/18614	0.13514	0.451993	0.405689	PMAIP1/SY	12
GO:000226	BP	GO:000226	lymphocyte	6/902	72/18614	0.135533	0.451993	0.405689	PMAIP1/TN	6
GO:004566	BP	GO:004566	negative re	6/902	72/18614	0.135533	0.451993	0.405689	IRX3/NOT	6
GO:004662	BP	GO:004662	regulation	6/902	72/18614	0.135533	0.451993	0.405689	SESN3/PTP	6
GO:006026	BP	GO:006026	regulation	6/902	72/18614	0.135533	0.451993	0.405689	MED31/ME	6
GO:014023	BP	GO:014023	presynapti	6/902	72/18614	0.135533	0.451993	0.405689	SYT8/DGK	6
GO:005115	BP	GO:005115	negative re	4/902	41/18614	0.135687	0.451993	0.405689	FZD7/FOX	4
GO:009875	BP	GO:009875	bone cell c	4/902	41/18614	0.135687	0.451993	0.405689	SH2B3/FO	4
GO:000184	BP	GO:000184	neural tube	8/902	105/18614	0.136949	0.451993	0.405689	TGFB2/SO	8
GO:000667	BP	GO:000667	ceramide r	8/902	105/18614	0.136949	0.451993	0.405689	ST6GALNA	8
GO:000676	BP	GO:000676	vitamin me	8/902	105/18614	0.136949	0.451993	0.405689	CYP26B1/	8
GO:007147	BP	GO:007147	cellular res	13/902	191/18614	0.137055	0.451993	0.405689	MFAP4/HS	13
GO:004215	BP	GO:004215	lipoprotein	10/902	139/18614	0.137635	0.451993	0.405689	APOL3/ZD	10
GO:009010	BP	GO:009010	positive re	9/902	122/18614	0.137851	0.451993	0.405689	GATA6/GD	9
GO:005105	BP	GO:005105	negative re	29/902	483/18614	0.137901	0.451993	0.405689	ISCU/SUM	29
GO:000032	BP	GO:000032	re-entry in	1/902	3/18614	0.138451	0.451993	0.405689	CCND1	1
GO:000170	BP	GO:000170	ectoderm f	1/902	3/18614	0.138451	0.451993	0.405689	FZD7	1
GO:000205	BP	GO:000205	osteoblast	1/902	3/18614	0.138451	0.451993	0.405689	RUNX2	1
GO:000226	BP	GO:000226	follicular de	1/902	3/18614	0.138451	0.451993	0.405689	BCL3	1
GO:000303	BP	GO:000303	detection c	1/902	3/18614	0.138451	0.451993	0.405689	SOD2	1
GO:000316	BP	GO:000316	Purkinje my	1/902	3/18614	0.138451	0.451993	0.405689	IRX3	1
GO:000316	BP	GO:000316	atrioventric	1/902	3/18614	0.138451	0.451993	0.405689	IRX3	1
GO:000321	BP	GO:000321	cardiac righ	1/902	3/18614	0.138451	0.451993	0.405689	SHOX2	1
GO:000327	BP	GO:000327	apoptotic p	1/902	3/18614	0.138451	0.451993	0.405689	TGFB2	1
GO:000604	BP	GO:000604	glucosamir	1/902	3/18614	0.138451	0.451993	0.405689	PGM3	1
GO:000617	BP	GO:000617	dAMP bios	1/902	3/18614	0.138451	0.451993	0.405689	ADK	1
GO:000623	BP	GO:000623	dTMP bios	1/902	3/18614	0.138451	0.451993	0.405689	TYMS	1
GO:000657	BP	GO:000657	tyrosine bi	1/902	3/18614	0.138451	0.451993	0.405689	PCBD2	1
GO:000674	BP	GO:000674	NADP cata	1/902	3/18614	0.138451	0.451993	0.405689	NUDT12	1
GO:000718	BP	GO:000718	transformir	1/902	3/18614	0.138451	0.451993	0.405689	EID2	1
GO:000720	BP	GO:000720	phospholip	1/902	3/18614	0.138451	0.451993	0.405689	HTR2A	1
GO:000829	BP	GO:000829	acetylcholi	1/902	3/18614	0.138451	0.451993	0.405689	CHAT	1
GO:000909	BP	GO:000909	aromatic a	1/902	3/18614	0.138451	0.451993	0.405689	PCBD2	1
GO:000917	BP	GO:000917	purine deo	1/902	3/18614	0.138451	0.451993	0.405689	ADK	1
GO:000922	BP	GO:000922	nucleotide	1/902	3/18614	0.138451	0.451993	0.405689	MGAT1	1
GO:001051	BP	GO:001051	positive re	1/902	3/18614	0.138451	0.451993	0.405689	HTR2A	1
GO:001069	BP	GO:001069	negative re	1/902	3/18614	0.138451	0.451993	0.405689	TGFB2	1
GO:001090	BP	GO:001090	positive re	1/902	3/18614	0.138451	0.451993	0.405689	CTNNB1	1
GO:001404	BP	GO:001404	negative re	1/902	3/18614	0.138451	0.451993	0.405689	EDNRB	1
GO:001609	BP	GO:001609	polyprenol	1/902	3/18614	0.138451	0.451993	0.405689	AKR1C3	1
GO:001610	BP	GO:001610	tetraterpen	1/902	3/18614	0.138451	0.451993	0.405689	BCO2	1
GO:001611	BP	GO:001611	carotenoid	1/902	3/18614	0.138451	0.451993	0.405689	BCO2	1
GO:001618	BP	GO:001618	synaptic ve	1/902	3/18614	0.138451	0.451993	0.405689	VAMP4	1
GO:001625	BP	GO:001625	selenocyst	1/902	3/18614	0.138451	0.451993	0.405689	SEPHS2	1
GO:001718	BP	GO:001718	peptidyl-g	1/902	3/18614	0.138451	0.451993	0.405689	GGCX	1
GO:001821	BP	GO:001821	protein car	1/902	3/18614	0.138451	0.451993	0.405689	GGCX	1
GO:001924	BP	GO:001924	methylglyc	1/902	3/18614	0.138451	0.451993	0.405689	PNKD	1
GO:001927	BP	GO:001927	UDP-N-ac	1/902	3/18614	0.138451	0.451993	0.405689	EXTL2	1
GO:001929	BP	GO:001929	tyrosine bi	1/902	3/18614	0.138451	0.451993	0.405689	PCBD2	1
GO:001986	BP	GO:001986	uracil meta	1/902	3/18614	0.138451	0.451993	0.405689	TYMS	1
GO:003057	BP	GO:003057	Cajal body	1/902	3/18614	0.138451	0.451993	0.405689	ZPR1	1
GO:003099	BP	GO:003099	regulation	1/902	3/18614	0.138451	0.451993	0.405689	CTNNB1	1
GO:003113	BP	GO:003113	sister chror	1/902	3/18614	0.138451	0.451993	0.405689	RB1	1
GO:003150	BP	GO:003150	pericentric	1/902	3/18614	0.138451	0.451993	0.405689	SIRT6	1
GO:003205	BP	GO:003205	negative re	1/902	3/18614	0.138451	0.451993	0.405689	RBM4	1
GO:003245	BP	GO:003245	regulation	1/902	3/18614	0.138451	0.451993	0.405689	ISG15	1
GO:003302	BP	GO:003302	mast cell a	1/902	3/18614	0.138451	0.451993	0.405689	KITLG	1
GO:003302	BP	GO:003302	regulation	1/902	3/18614	0.138451	0.451993	0.405689	KITLG	1
GO:003314	BP	GO:003314	negative re	1/902	3/18614	0.138451	0.451993	0.405689	GADD45A	1
GO:003322	BP	GO:003322	dsRNA trar	1/902	3/18614	0.138451	0.451993	0.405689	RFTN1	1
GO:003402	BP	GO:003402	response t	1/902	3/18614	0.138451	0.451993	0.405689	SOD2	1
GO:003414	BP	GO:003414	negative re	1/902	3/18614	0.138451	0.451993	0.405689	TNFAIP3	1
GO:003443	BP	GO:003443	glycoprote	1/902	3/18614	0.138451	0.451993	0.405689	SLC10A7	1
GO:003443	BP	GO:003443	lipoprotein	1/902	3/18614	0.138451	0.451993	0.405689	SAMD1	1
GO:003528	BP	GO:003528	brain segm	1/902	3/18614	0.138451	0.451993	0.405689	MAFB	1
GO:003555	BP	GO:003555	oxidative s	1/902	3/18614	0.138451	0.451993	0.405689	ALKBH5	1
GO:003578	BP	GO:003578	CD86 biosy	1/902	3/18614	0.138451	0.451993	0.405689	TREX1	1
GO:003579	BP	GO:003579	positive re	1/902	3/18614	0.138451	0.451993	0.405689	PDGFA	1
GO:003602	BP	GO:003602	embryonic	1/902	3/18614	0.138451	0.451993	0.405689	CTNNB1	1
GO:003648	BP	GO:003648	neuron intr	1/902	3/18614	0.138451	0.451993	0.405689	HYOU1	1
GO:003652	BP	GO:003652	astrocyte-	1/902	3/18614	0.138451	0.451993	0.405689	CTNNB1	1
GO:003901	BP	GO:003901	pronephric	1/902	3/18614	0.138451	0.451993	0.405689	HNF1B	1
GO:004216	BP	GO:004216	lipoprotein	1/902	3/18614	0.138451	0.451993	0.405689	SAMD1	1
GO:004216	BP	GO:004216	lipoprotein	1/902	3/18614	0.138451	0.451993	0.405689	SAMD1	1
GO:004221	BP	GO:004221	terpene me	1/902	3/18614	0.138451	0.451993	0.405689	BCO2	1
GO:004253	BP	GO:004253	hypotonic	1/902	3/18614	0.138451	0.451993	0.405689	SLC12A6	1
GO:004393	BP	GO:004393	ossificatior	1/902	3/18614	0.138451	0.451993	0.405689	PTN	1
GO:004402	BP	GO:004402	hypomethy	1/902	3/18614	0.138451	0.451993	0.405689	PIK3CA	1
GO:004433	BP	GO:004433	canonical	1/902	3/18614	0.138451	0.451993	0.405689	CTNNB1	1
GO:004486	BP	GO:004486	modulatior	1/902	3/18614	0.138451	0.451993	0.405689	PTX3	1
GO:004558	BP	GO:004558	regulation	1/902	3/18614	0.138451	0.451993	0.405689	TNFSF9	1
GO:004562	BP	GO:004562	negative re	1/902	3/18614	0.138451	0.451993	0.405689	IL4R	1
GO:004565	BP	GO:004565	regulation	1/902	3/18614	0.138451	0.451993	0.405689	INPP5D	1
GO:004572	BP	GO:004572	positive re	1/902	3/18614	0.138451	0.451993	0.405689	TGFB2	1
GO:004606	BP	GO:004606	dATP meta	1/902	3/18614	0.138451	0.451993	0.405689	ADK	1
GO:004607	BP	GO:004607	dTMP cata	1/902	3/18614	0.138451	0.451993	0.405689	TYMP	1
GO:004629	BP	GO:004629	formaldehy	1/902	3/18614	0.138451	0.451993	0.405689	ADH5	1
GO:004810	BP	GO:004810	establishm	1/902	3/18614	0.138451	0.451993	0.405689	FZD6	1
GO:004810	BP	GO:004810	establishm	1/902	3/18614	0.138451	0.451993	0.405689	FZD6	1
GO:004867	BP	GO:004867	axon exten	1/902	3/18614	0.138451	0.451993	0.405689	TNR	1
GO:004868	BP	GO:004868	sprouting c	1/902	3/18614	0.138451	0.451993	0.405689	TNR	1
GO:005043	BP	GO:005043	positive re	1/902	3/18614	0.138451	0.451993	0.405689	MDFIC	1
GO:005158	BP	GO:005158	negative re	1/902	3/18614	0.138451	0.451993	0.405689	SNCA	1
GO:005161	BP	GO:005161	negative re	1/902	3/18614	0.138451	0.451993	0.405689	SNCA	1
GO:00517[illegible]	BP	GO:00517[illegible]	positive re	1/902	3/18614	0.138451	0.451993	0.405689	TGFB2	1
GO:00519[illegible]	BP	GO:00519[illegible]	lysophosph	1/902	3/18614	0.138451	0.451993	0.405689	MFSD2A	1
GO:00602[illegible]	BP	GO:00602[illegible]	negative re	1/902	3/18614	0.138451	0.451993	0.405689	TOB1	1
GO:00603[illegible]	BP	GO:00603[illegible]	foregut reg	1/902	3/18614	0.138451	0.451993	0.405689	HIPK1	1
GO:00604[illegible]	BP	GO:00604[illegible]	lung field s	1/902	3/18614	0.138451	0.451993	0.405689	CTNNB1	1

GO:000815	MF	GO:000815	hedgehog	1/1068	3/18369	0.164488	0.453422	0.445447	B9D1	1
GO:000818	MF	GO:000818	glycogen p	1/1068	3/18369	0.164488	0.453422	0.445447	PYGL	1
GO:000838	MF	GO:000838	IkappaB ki	1/1068	3/18369	0.164488	0.453422	0.445447	IKBKB	1
GO:000842	MF	GO:000842	protein kin	1/1068	3/18369	0.164488	0.453422	0.445447	SFN	1
GO:000848	MF	GO:000848	UDP-galac	1/1068	3/18369	0.164488	0.453422	0.445447	UGT8	1
GO:000853	MF	GO:000853	oxidized pu	1/1068	3/18369	0.164488	0.453422	0.445447	MUTYH	1
GO:000898	MF	GO:000898	rRNA (ade	1/1068	3/18369	0.164488	0.453422	0.445447	METTL16	1
GO:001526	MF	GO:001526	urea chanr	1/1068	3/18369	0.164488	0.453422	0.445447	SLC14A1	1
GO:001667	MF	GO:001667	oxidoreduc	1/1068	3/18369	0.164488	0.453422	0.445447	GSTO2	1
GO:001697	MF	GO:001697	flavin-linke	1/1068	3/18369	0.164488	0.453422	0.445447	QSOX2	1
GO:001702	MF	GO:001702	myosin pho	1/1068	3/18369	0.164488	0.453422	0.445447	PPP1R16A	1
GO:001916	MF	GO:001916	trans-2-er	1/1068	3/18369	0.164488	0.453422	0.445447	MECR	1
GO:001980	MF	GO:001980	polyamine	1/1068	3/18369	0.164488	0.453422	0.445447	AMD1	1
GO:001997	MF	GO:001997	interleukin-	1/1068	3/18369	0.164488	0.453422	0.445447	IL6R	1
GO:003061	MF	GO:003061	oxidoreduc	1/1068	3/18369	0.164488	0.453422	0.445447	GSTO2	1
GO:003061	MF	GO:003061	oxidoreduc	1/1068	3/18369	0.164488	0.453422	0.445447	GSTO2	1
GO:003153	MF	GO:003153	thyrotropir	1/1068	3/18369	0.164488	0.453422	0.445447	PSMC5	1
GO:003169	MF	GO:003169	beta-1 adr	1/1068	3/18369	0.164488	0.453422	0.445447	MAGI2	1
GO:003174	MF	GO:003174	D2 dopam	1/1068	3/18369	0.164488	0.453422	0.445447	DNM2	1
GO:003186	MF	GO:003186	prostanoid	1/1068	3/18369	0.164488	0.453422	0.445447	RPGRIP1L	1
GO:003187	MF	GO:003187	proteinase	1/1068	3/18369	0.164488	0.453422	0.445447	PDCD6IP	1
GO:003203	MF	GO:003203	myosin II h	1/1068	3/18369	0.164488	0.453422	0.445447	LIMCH1	1
GO:003276	MF	GO:003276	copper-de	1/1068	3/18369	0.164488	0.453422	0.445447	ATOX1	1
GO:003463	MF	GO:003463	retinol tran	1/1068	3/18369	0.164488	0.453422	0.445447	RBP4	1
GO:003517	MF	GO:003517	histone kin	1/1068	3/18369	0.164488	0.453422	0.445447	VRK1	1
GO:003600	MF	GO:003600	protein-glu	1/1068	3/18369	0.164488	0.453422	0.445447	FBL	1
GO:003631	MF	GO:003631	phosphatic	1/1068	3/18369	0.164488	0.453422	0.445447	GSN	1
GO:003955	MF	GO:003955	RIG-I bindi	1/1068	3/18369	0.164488	0.453422	0.445447	SEC14L1	1
GO:004261	MF	GO:004261	CD8 recep	1/1068	3/18369	0.164488	0.453422	0.445447	HLA-G	1
GO:004373	MF	GO:004373	DNA-N1-r	1/1068	3/18369	0.164488	0.453422	0.445447	ALKBH3	1
GO:004377	MF	GO:004377	cytidine kir	1/1068	3/18369	0.164488	0.453422	0.445447	UCK2	1
GO:004471	MF	GO:004471	8-oxo-dGl	1/1068	3/18369	0.164488	0.453422	0.445447	NUDT18	1
GO:004659	MF	GO:004659	polyamine	1/1068	3/18369	0.164488	0.453422	0.445447	SMOX	1
GO:004697	MF	GO:004697	TAP2 bindi	1/1068	3/18369	0.164488	0.453422	0.445447	HLA-F	1
GO:004737	MF	GO:004737	all-trans-re	1/1068	3/18369	0.164488	0.453422	0.445447	PNPLA4	1
GO:005050	MF	GO:005050	glucuronos	1/1068	3/18369	0.164488	0.453422	0.445447	EXT2	1
GO:005056	MF	GO:005056	glutaminyl-	1/1068	3/18369	0.164488	0.453422	0.445447	GATC	1
GO:005063	MF	GO:005063	acetyl-CoA	1/1068	3/18369	0.164488	0.453422	0.445447	ACAA1	1
GO:005103	MF	GO:005103	nucleic aci	1/1068	3/18369	0.164488	0.453422	0.445447	SIDT2	1
GO:005103	MF	GO:005103	RNA transr	1/1068	3/18369	0.164488	0.453422	0.445447	SIDT2	1
GO:005126	MF	GO:005126	mono-olei	1/1068	3/18369	0.164488	0.453422	0.445447	PNPLA4	1
GO:005126	MF	GO:005126	diolein trar	1/1068	3/18369	0.164488	0.453422	0.445447	PNPLA4	1
GO:005283	MF	GO:005283	inositol 5-c	1/1068	3/18369	0.164488	0.453422	0.445447	IP6K1	1
GO:005283	MF	GO:005283	inositol dip	1/1068	3/18369	0.164488	0.453422	0.445447	IP6K1	1
GO:005510	MF	GO:005510	adiponecti	1/1068	3/18369	0.164488	0.453422	0.445447	ADIPOR2	1
GO:006215	MF	GO:006215	C5-methyl	1/1068	3/18369	0.164488	0.453422	0.445447	YTHDF2	1
GO:007029	MF	GO:007029	N-acylpho	1/1068	3/18369	0.164488	0.453422	0.445447	PLD1	1
GO:009054	MF	GO:009054	MIT domai	1/1068	3/18369	0.164488	0.453422	0.445447	IST1	1
GO:009736	MF	GO:009736	protein O-	1/1068	3/18369	0.164488	0.453422	0.445447	OGT	1
GO:010225	MF	GO:010225	linear malt	1/1068	3/18369	0.164488	0.453422	0.445447	PYGL	1
GO:010249	MF	GO:010249	SHG alpha	1/1068	3/18369	0.164488	0.453422	0.445447	PYGL	1
GO:014029	MF	GO:014029	ADP-ribos	1/1068	3/18369	0.164488	0.453422	0.445447	OARD1	1
GO:014058	MF	GO:014058	promoter-	1/1068	3/18369	0.164488	0.453422	0.445447	RUVBL2	1
GO:014058	MF	GO:014058	chromatin	1/1068	3/18369	0.164488	0.453422	0.445447	RUVBL2	1
GO:014067	MF	GO:014067	ATP-deper	1/1068	3/18369	0.164488	0.453422	0.445447	VPS72	1
GO:190412	MF	GO:190412	phosphatic	1/1068	3/18369	0.164488	0.453422	0.445447	MTTP	1
GO:190550	MF	GO:190550	acetyl-CoA	1/1068	3/18369	0.164488	0.453422	0.445447	PANK1	1
GO:190557	MF	GO:190557	ganglioside	1/1068	3/18369	0.164488	0.453422	0.445447	PSAP	1
GO:190557	MF	GO:190557	ganglioside	1/1068	3/18369	0.164488	0.453422	0.445447	PSAP	1
GO:190577	MF	GO:190577	8-hydroxy-	1/1068	3/18369	0.164488	0.453422	0.445447	BLM	1
GO:199018	MF	GO:199018	peptide-se	1/1068	3/18369	0.164488	0.453422	0.445447	NAA40	1
GO:001640	MF	GO:001640	palmitoyltr	4/1068	37/18369	0.16592	0.45498	0.446978	SPTLC2/ZD	4
GO:003519	MF	GO:003519	miRNA bin	4/1068	37/18369	0.16592	0.45498	0.446978	RBM10/ZN	4
GO:009952	MF	GO:009952	neurotrans	4/1068	37/18369	0.16592	0.45498	0.446978	GRIK2/GAE	4
GO:000818	MF	GO:000818	ATP-deper	7/1068	78/18369	0.16725	0.457828	0.449776	AQR/DHX1	7
GO:190293	MF	GO:190293	phosphatic	9/1068	107/18369	0.169834	0.464096	0.455933	ASAP1/AN	9
GO:004317	MF	GO:004317	organic aci	16/1068	212/18369	0.172549	0.464959	0.456782	ALKBH3/PI	16
GO:000400	MF	GO:000400	adenosine	2/1068	13/18369	0.172613	0.464959	0.456782	ADAT1/AD	2
GO:000825	MF	GO:000825	5'-nucleoti	2/1068	13/18369	0.172613	0.464959	0.456782	NT5C3A/N	2
GO:001620	MF	GO:001620	AMP bindir	2/1068	13/18369	0.172613	0.464959	0.456782	PYGL/PRPS	2
GO:001684	MF	GO:001684	carbon-nit	2/1068	13/18369	0.172613	0.464959	0.456782	GGACT/AS	2
GO:001910	MF	GO:001910	DNA N-gly	2/1068	13/18369	0.172613	0.464959	0.456782	MPG/MUTY	2
GO:003423	MF	GO:003423	protein kin	2/1068	13/18369	0.172613	0.464959	0.456782	SOX9/EZR	2
GO:005101	MF	GO:005101	microtubul	2/1068	13/18369	0.172613	0.464959	0.456782	NUMA1/CA	2
GO:000166	MF	GO:000166	G protein-	21/1068	289/18369	0.172939	0.464959	0.456782	OXT/JAK2/	21
GO:005101	MF	GO:005101	protein kin	5/1068	51/18369	0.1734	0.464959	0.456782	RDX/SOX9	5
GO:006198	MF	GO:006198	regulatory	5/1068	51/18369	0.1734	0.464959	0.456782	FAM172A/	5
GO:001628	MF	GO:001628	CoA hydro	3/1068	25/18369	0.175281	0.468371	0.460133	PPT1/ACOT	3
GO:007084	MF	GO:007084	dynein con	3/1068	25/18369	0.175281	0.468371	0.460133	PAFAH1B1	3
GO:004205	MF	GO:004205	histone me	6/1068	65/18369	0.175565	0.468371	0.460133	SETD4/SMY	6
GO:001682	MF	GO:001682	lyase activi	15/1068	198/18369	0.17836	0.474744	0.466394	GGACT/AS	15
GO:001712	MF	GO:001712	SH3 doma	10/1068	123/18369	0.178557	0.474744	0.466394	ARHGAP17	10
GO:003329	MF	GO:003329	monocarbo	7/1068	80/18369	0.18286	0.48518	0.476647	PC/GSTP1/	7
GO:001685	MF	GO:001685	isomerase	12/1068	154/18369	0.185699	0.48518	0.476647	GSTZ1/GA	12
GO:001662	MF	GO:001662	oxidoreduc	4/1068	39/18369	0.189358	0.48518	0.476647	OGDH/PDH	4
GO:001689	MF	GO:001689	endonuclea	4/1068	39/18369	0.189358	0.48518	0.476647	POP7/POP	4
GO:003033	MF	GO:003033	nuclear est	4/1068	39/18369	0.189358	0.48518	0.476647	PHB2/PAG	4
GO:000109	MF	GO:000109	RNA polym	3/1068	26/18369	0.190152	0.48518	0.476647	FBL/RUVBL	3
GO:000814	MF	GO:000814	poly(A) bin	3/1068	26/18369	0.190152	0.48518	0.476647	PABPC4/SY	3
GO:000857	MF	GO:000857	plus-end-c	3/1068	26/18369	0.190152	0.48518	0.476647	KIF1A/DYN	3
GO:004802	MF	GO:004802	mRNA 5'-(	3/1068	26/18369	0.190152	0.48518	0.476647	NCL/SYNC	3
GO:000203	MF	GO:000203	p53 bindin	6/1068	67/18369	0.193282	0.48518	0.476647	MUC1/TRII	6
GO:005099	MF	GO:005099	quaternary	5/1068	53/18369	0.193496	0.48518	0.476647	PCYT1B/ES	5
GO:014029	MF	GO:014029	general tra	5/1068	53/18369	0.193496	0.48518	0.476647	FBL/RUVBL	5
GO:000819	MF	GO:000819	phosphatic	2/1068	14/18369	0.194033	0.48518	0.476647	LPIN3/LPIN	2
GO:001630	MF	GO:001630	phosphatic	2/1068	14/18369	0.194033	0.48518	0.476647	PIK3C2G/P	2
GO:004313	MF	GO:004313	3'-5' DNA	2/1068	14/18369	0.194033	0.48518	0.476647	BLM/MCM	2
GO:007183	MF	GO:007183	HMG box c	2/1068	14/18369	0.194033	0.48518	0.476647	PAX6/SP1	2
GO:007193	MF	GO:007193	Arp2/3 cor	2/1068	14/18369	0.194033	0.48518	0.476647	AVIL/WASI	2
GO:001679	MF	GO:001679	thiolester h	4/1068	40/18369	0.20145	0.48518	0.476647	PPT1/ACOT	4
GO:004848	MF	GO:004848	beta-tubul	4/1068	40/18369	0.20145	0.48518	0.476647	EML4/UXT	4
GO:002302	MF	GO:002302	MHC class	3/1068	27/18369	0.205328	0.48518	0.476647	ANXA11/H	3
GO:000017	MF	GO:000017	rRNA (ade	1/1068	4/18369	0.213074	0.48518	0.476647	DIMT1	1
GO:000040	MF	GO:000040	Y-form DN	1/1068	4/18369	0.213074	0.48518	0.476647	BLM	1
GO:000070	MF	GO:000070	mismatch	1/1068	4/18369	0.213074	0.48518	0.476647	MUTYH	1
GO:000070	MF	GO:000070	oxidized ba	1/1068	4/18369	0.213074	0.48518	0.476647	MUTYH	1
GO:000102	MF	GO:000102	RNA polym	1/1068	4/18369	0.213074	0.48518	0.476647	BDP1	1
GO:000205	MF	GO:000205	nucleobase	1/1068	4/18369	0.213074	0.48518	0.476647	PYGL	1
GO:000391	MF	GO:000391	DNA topoi	1/1068	4/18369	0.213074	0.48518	0.476647	TOP1MT	1
GO:000404	MF	GO:000404	aminoacyl-	1/1068	4/18369	0.213074	0.48518	0.476647	PTRHD1	1
GO:000412	MF	GO:000412	cytidylate	1/1068	4/18369	0.213074	0.48518	0.476647	AK7	1
GO:000445	MF	GO:000445	L-lactate c	1/1068	4/18369	0.213074	0.48518	0.476647	LDHB	1
GO:000446	MF	GO:000446	long-chain	1/1068	4/18369	0.213074	0.48518	0.476647	ACADL	1
GO:000459	MF	GO:000459	oxoglutara	1/1068	4/18369	0.213074	0.48518	0.476647	OGDH	1
GO:000463	MF	GO:000463	phosphopy	1/1068	4/18369	0.213074	0.48518	0.476647	ENO3	1
GO:000473	MF	GO:000473	pyrroline-5	1/1068	4/18369	0.213074	0.48518	0.476647	PYCR1	1
GO:000478	MF	GO:000478	superoxide	1/1068	4/18369	0.213074	0.48518	0.476647	SOD3	1
GO:000482	MF	GO:000482	phenylalan	1/1068	4/18369	0.213074	0.48518	0.476647	FARS2	1
GO:000484	MF	GO:000484	uridine kina	1/1068	4/18369	0.213074	0.48518	0.476647	UCK2	1
GO:000489	MF	GO:000489	ciliary neur	1/1068	4/18369	0.213074	0.48518	0.476647	IL6R	1
GO:000547	MF	GO:000547	ATP:ADP a	1/1068	4/18369	0.213074	0.48518	0.476647	SLC25A6	1
GO:000553	MF	GO:000553	galactose	1/1068	4/18369	0.213074	0.48518	0.476647	COLEC12	1
GO:000843	MF	GO:000843	vitamin E b	1/1068	4/18369	0.213074	0.48518	0.476647	SEC14L2	1
GO:000844	MF	GO:000844	inositol-1,4	1/1068	4/18369	0.213074	0.48518	0.476647	ITPKC	1
GO:001052	MF	GO:001052	telomerase	1/1068	4/18369	0.213074	0.48518	0.476647	PINX1	1
GO:001518	MF	GO:001518	L-cystine t	1/1068	4/18369	0.213074	0.48518	0.476647	SLC7A9	1
GO:001544	MF	GO:001544	ABC-type	1/1068	4/18369	0.213074	0.48518	0.476647	ABCB9	1
GO:001566	MF	GO:001566	alcohol tra	1/1068	4/18369	0.213074	0.48518	0.476647	RBP4	1
GO:001662	MF	GO:001662	oxidoreduc	1/1068	4/18369	0.213074	0.48518	0.476647	FMO5	1
GO:001664	MF	GO:001664	oxidoreduc	1/1068	4/18369	0.213074	0.48518	0.476647	SMOX	1
GO:001672	MF	GO:001672	oxidoreduc	1/1068	4/18369	0.213074	0.48518	0.476647	SOD3	1
GO:001706	MF	GO:001706	fatty acid a	1/1068	4/18369	0.213074	0.48518	0.476647	FAAH	1
GO:003006	MF	GO:003006	L-malate c	1/1068	4/18369	0.213074	0.48518	0.476647	PHGDH	1
GO:003019	MF	GO:003019	extracellula	1/1068	4/18369	0.213074	0.48518	0.476647	MUC17	1
GO:003022	MF	GO:003022	very-low-c	1/1068	4/18369	0.213074	0.48518	0.476647	LDLR	1
GO:003178	MF	GO:003178	type 3 mel	1/1068	4/18369	0.213074	0.48518	0.476647	POMC	1
GO:003178	MF	GO:003178	type 4 mel	1/1068	4/18369	0.213074	0.48518	0.476647	POMC	1
GO:003184	MF	GO:003184	neuropepti	1/1068	4/18369	0.213074	0.48518	0.476647	NPY	1
GO:003405	MF	GO:003405	estrogen re	1/1068	4/18369	0.213074	0.48518	0.476647	TRIM24	1
GO:003424	MF	GO:003424	mitochond	1/1068	4/18369	0.213074	0.48518	0.476647	FOXO3	1
GO:003445	MF	GO:003445	3'-5' RNA	1/1068	4/18369	0.213074	0.48518	0.476647	AQR	1
GO:003473	MF	GO:003473	histone dea	1/1068	4/18369	0.213074	0.48518	0.476647	HDAC9	1
GO:003524	MF	GO:003524	protein-arg	1/1068	4/18369	0.213074	0.48518	0.476647	PRMT9	1
GO:003536	MF	GO:003536	selenocyst	1/1068	4/18369	0.213074	0.48518	0.476647	SECISBP2	1
GO:004353	MF	GO:004353	angiostatir	1/1068	4/18369	0.213074	0.48518	0.476647	AMOT	1
GO:004454	MF	GO:004454	DNA topoi	1/1068	4/18369	0.213074	0.48518	0.476647	NCL	1
GO:004514	MF	GO:004514	single-stra	1/1068	4/18369	0.213074	0.48518	0.476647	PLD3	1
GO:004697	MF	GO:004697	TAP1 bindi	1/1068	4/18369	0.213074	0.48518	0.476647	HLA-F	1
GO:004769	MF	GO:004769	beta-adrer	1/1068	4/18369	0.213074	0.48518	0.476647	GRK5	1
GO:005021	MF	GO:005021	propionate	1/1068	4/18369	0.213074	0.48518	0.476647	ACSM3	1
GO:005048	MF	GO:005048	IMP 5'-nuc	1/1068	4/18369	0.213074	0.48518	0.476647	NT5C2	1
GO:005137	MF	GO:005137	mannose-(	1/1068	4/18369	0.213074	0.48518	0.476647	PIGO	1
GO:005138	MF	GO:005138	norepineph	1/1068	4/18369	0.213074	0.48518	0.476647	ADRB2	1
GO:005259	MF	GO:005259	aliphatic-a	1/1068	4/18369	0.213074	0.48518	0.476647	AOC3	1
GO:006182	MF	GO:006182	telomeric D	1/1068	4/18369	0.213074	0.48518	0.476647	BLM	1
GO:006189	MF	GO:006189	calcium ior	1/1068	4/18369	0.213074	0.48518	0.476647	EFHD1	1
GO:007002	MF	GO:007002	nitric oxide	1/1068	4/18369	0.213074	0.48518	0.476647	GSTP1	1
GO:007005	MF	GO:007005	thrombosp	1/1068	4/18369	0.213074	0.48518	0.476647	SDC4	1
GO:007069	MF	GO:007069	activin rece	1/1068	4/18369	0.213074	0.48518	0.476647	MAGI2	1
GO:007099	MF	GO:007099	type 1 mel	1/1068	4/18369	0.213074	0.48518	0.476647	POMC	1
GO:007232	MF	GO:007232	volume-se	1/1068	4/18369	0.213074	0.48518	0.476647	TTYH2	1
GO:009700	MF	GO:009700	adipokinet	1/1068	4/18369	0.213074	0.48518	0.476647	ADIPOR2	1
GO:009701	MF	GO:009701	L27 domai	1/1068	4/18369	0.213074	0.48518	0.476647	LIN7A	1
GO:009951	MF	GO:009951	voltage-ga	1/1068	4/18369	0.213074	0.48518	0.476647	CACNB4	1
GO:009953	MF	GO:009953	G protein-	1/1068	4/18369	0.213074	0.48518	0.476647	GABBR1	1
GO:009962	MF	GO:009962	voltage-ga	1/1068	4/18369	0.213074	0.48518	0.476647	CACNB4	1
GO:010215	MF	GO:010215	very-long-	1/1068	4/18369	0.213074	0.48518	0.476647	HACD4	1
GO:010234	MF	GO:010234	3-hydroxy-	1/1068	4/18369	0.213074	0.48518	0.476647	HACD4	1
GO:010234	MF	GO:010234	3-hydroxy-	1/1068	4/18369	0.213074	0.48518	0.476647	HACD4	1
GO:010234	MF	GO:010234	3-hydroxy-	1/1068	4/18369	0.213074	0.48518	0.476647	HACD4	1
GO:010626	MF	GO:010626	DNA-DNA	1/1068	4/18369	0.213074	0.48518	0.476647	RUVBL2	1
GO:010626	MF	GO:010626	1-acylglyc	1/1068	4/18369	0.213074	0.48518	0.476647	MBOAT2	1
GO:010626	MF	GO:010626	1-acylglyc	1/1068	4/18369	0.213074	0.48518	0.476647	MBOAT2	1
GO:014026	MF	GO:014026	mRNA cap	1/1068	4/18369	0.213074	0.48518	0.476647	SRRT	1
GO:014072	MF	GO:014072	nuclease ir	1/1068	4/18369	0.213074	0.48518	0.476647	DFFA	1
GO:190428	MF	GO:190428	BAT3 comp	1/1068	4/18369	0.213074	0.48518	0.476647	VCP	1
GO:190439	MF	GO:190439	heparan su	1/1068	4/18369	0.213074	0.48518	0.476647	PPIA	1
GO:004216	MF	GO:004216	SH2 doma	4/1068	41/18369	0.213764	0.48518	0.476647	JAK2/SYNC	4
GO:001659	MF	GO:001659	amino acic	5/1068	55/18369	0.214358	0.48518	0.476647	DDAH1/GL	5
GO:000408	MF	GO:000408	carbonate	2/1068	15/18369	0.215762	0.48518	0.476647	CA5B/CA9	2
GO:000504	MF	GO:000504	low-densit	2/1068	15/18369	0.215762	0.48518	0.476647	CXCL16/LD	2
GO:000824	MF	GO:000824	omega pep	2/1068	15/18369	0.215762	0.48518	0.476647	ASRGL1/G	2
GO:000825	MF	GO:000825	nucleotida	2/1068	15/18369	0.215762	0.48518	0.476647	NT5C3A/N	2
GO:001018	MF	GO:001018	FMN bindi	2/1068	15/18369	0.215762	0.48518	0.476647	NDUFV1/N	2
GO:001592	MF	GO:001592	hexosamin	2/1068	15/18369	0.215762	0.48518	0.476647	HYAL1/NA	2
GO:003100	MF	GO:003100	filamin bin	2/1068	15/18369	0.215762	0.48518	0.476647	DPYSL3/NE	2
GO:005137	MF	GO:005137	muscle alp	2/1068	15/18369	0.215762	0.48518	0.476647	PALLD/PDL	2
GO:010299	MF	GO:010299	myristoyl-(	2/1068	15/18369	0.215762	0.48518	0.476647	ACOT7/AC	2
GO:014003	MF	GO:014003	ubiquitin-c	2/1068	15/18369	0.215762	0.48518	0.476647	VCP/ANKR	2
GO:000813	MF	GO:000813	translation	7/1068	84/18369	0.215773	0.48518	0.476647	EIF3D/EIF2	7
GO:000549	MF	GO:000549	steroid bin	8/1068	99/18369	0.217692	0.488799	0.480201	AR/NR3C1	8
GO:001664	MF	GO:001664	oxidoreduc	3/1068	28/18369	0.220766	0.494994	0.486288	BLVRB/SM	3
GO:190168	MF	GO:190168	sulfur com	19/1068	269/18369	0.221377	0.495659	0.486941	SOD3/CRIS	19

GO:006047	BP	GO:006047	positive re	1/902	3/18614	0.138451	0.451993	0.405689	DEFB1	1
GO:006049	BP	GO:006049	lung induct	1/902	3/18614	0.138451	0.451993	0.405689	CTNNB1	1
GO:006071	BP	GO:006071	syncytiotro	1/902	3/18614	0.138451	0.451993	0.405689	DNAJB6	1
GO:006076	BP	GO:006076	mammary	1/902	3/18614	0.138451	0.451993	0.405689	CSF1	1
GO:006076	BP	GO:006076	positive re	1/902	3/18614	0.138451	0.451993	0.405689	CTNNB1	1
GO:006083	BP	GO:006083	lymphatic e	1/902	3/18614	0.138451	0.451993	0.405689	NR2F2	1
GO:006119	BP	GO:006119	fungiform	1/902	3/18614	0.138451	0.451993	0.405689	CTNNB1	1
GO:006151	BP	GO:006151	macrophag	1/902	3/18614	0.138451	0.451993	0.405689	CSF1	1
GO:006152	BP	GO:006152	Langerhan	1/902	3/18614	0.138451	0.451993	0.405689	ITGB8	1
GO:006174	BP	GO:006174	protein tar	1/902	3/18614	0.138451	0.451993	0.405689	LAMP2	1
GO:006175	BP	GO:006175	substrate l	1/902	3/18614	0.138451	0.451993	0.405689	TOM1	1
GO:007042	BP	GO:007042	negative re	1/902	3/18614	0.138451	0.451993	0.405689	TNFAIP3	1
GO:007043	BP	GO:007043	negative re	1/902	3/18614	0.138451	0.451993	0.405689	TNFAIP3	1
GO:007049	BP	GO:007049	regulation	1/902	3/18614	0.138451	0.451993	0.405689	SNCA	1
GO:007049	BP	GO:007049	negative re	1/902	3/18614	0.138451	0.451993	0.405689	SNCA	1
GO:007068	BP	GO:007068	proteasom	1/902	3/18614	0.138451	0.451993	0.405689	PSMD10	1
GO:007116	BP	GO:007116	DNA replic	1/902	3/18614	0.138451	0.451993	0.405689	GMNN	1
GO:007133	BP	GO:007133	cellular res	1/902	3/18614	0.138451	0.451993	0.405689	XBP1	1
GO:007140	BP	GO:007140	cellular res	1/902	3/18614	0.138451	0.451993	0.405689	ABCG4	1
GO:007146	BP	GO:007146	cellular res	1/902	3/18614	0.138451	0.451993	0.405689	PIK3CA	1
GO:007147	BP	GO:007147	cellular hyp	1/902	3/18614	0.138451	0.451993	0.405689	SLC12A6	1
GO:007193	BP	GO:007193	negative re	1/902	3/18614	0.138451	0.451993	0.405689	RB1	1
GO:007213	BP	GO:007213	kidney mes	1/902	3/18614	0.138451	0.451993	0.405689	STAT1	1
GO:007213	BP	GO:007213	metanephr	1/902	3/18614	0.138451	0.451993	0.405689	STAT1	1
GO:008604	BP	GO:008604	membrane	1/902	3/18614	0.138451	0.451993	0.405689	SCN5A	1
GO:008604	BP	GO:008604	membrane	1/902	3/18614	0.138451	0.451993	0.405689	SCN5A	1
GO:008610	BP	GO:008610	endothelin	1/902	3/18614	0.138451	0.451993	0.405689	CDC42	1
GO:009020	BP	GO:009020	protein loc	1/902	3/18614	0.138451	0.451993	0.405689	SUMO1	1
GO:009021	BP	GO:009021	negative re	1/902	3/18614	0.138451	0.451993	0.405689	PIP4K2A	1
GO:009721	BP	GO:009721	regulation	1/902	3/18614	0.138451	0.451993	0.405689	LAPTM5	1
GO:009729	BP	GO:009729	activation (	1/902	3/18614	0.138451	0.451993	0.405689	SMAD3	1
GO:009891	BP	GO:009891	membrane	1/902	3/18614	0.138451	0.451993	0.405689	SCN5A	1
GO:009918	BP	GO:009918	trans-syna	1/902	3/18614	0.138451	0.451993	0.405689	NTRK2	1
GO:010638	BP	GO:010638	purine deo	1/902	3/18614	0.138451	0.451993	0.405689	ADK	1
GO:010638	BP	GO:010638	dAMP salva	1/902	3/18614	0.138451	0.451993	0.405689	ADK	1
GO:011007	BP	GO:011007	negative re	1/902	3/18614	0.138451	0.451993	0.405689	GPX4	1
GO:011015	BP	GO:011015	NAD-cap (	1/902	3/18614	0.138451	0.451993	0.405689	NUDT12	1
GO:012018	BP	GO:012018	positive re	1/902	3/18614	0.138451	0.451993	0.405689	SIRT6	1
GO:012025	BP	GO:012025	hydrocarbo	1/902	3/18614	0.138451	0.451993	0.405689	BCO2	1
GO:014021	BP	GO:014021	positive re	1/902	3/18614	0.138451	0.451993	0.405689	FABP3	1
GO:014046	BP	GO:014046	HRI-media	1/902	3/18614	0.138451	0.451993	0.405689	OMA1	1
GO:014057	BP	GO:014057	ascorbate	1/902	3/18614	0.138451	0.451993	0.405689	CYB561D2	1
GO:190005	BP	GO:190005	regulation	1/902	3/18614	0.138451	0.451993	0.405689	AKR1C3	1
GO:190008	BP	GO:190008	positive re	1/902	3/18614	0.138451	0.451993	0.405689	GREM1	1
GO:190010	BP	GO:190010	positive re	1/902	3/18614	0.138451	0.451993	0.405689	XBP1	1
GO:190020	BP	GO:190020	mesenchyr	1/902	3/18614	0.138451	0.451993	0.405689	HNF1B	1
GO:190021	BP	GO:190021	regulation	1/902	3/18614	0.138451	0.451993	0.405689	HNF1B	1
GO:190021	BP	GO:190021	negative re	1/902	3/18614	0.138451	0.451993	0.405689	HNF1B	1
GO:190023	BP	GO:190023	regulation	1/902	3/18614	0.138451	0.451993	0.405689	PDGFA	1
GO:190062	BP	GO:190062	acetate est	1/902	3/18614	0.138451	0.451993	0.405689	CHAT	1
GO:190107	BP	GO:190107	positive re	1/902	3/18614	0.138451	0.451993	0.405689	HRC	1
GO:190139	BP	GO:190139	positive re	1/902	3/18614	0.138451	0.451993	0.405689	GATA6	1
GO:190148	BP	GO:190148	regulation	1/902	3/18614	0.138451	0.451993	0.405689	SIRT6	1
GO:190148	BP	GO:190148	positive re	1/902	3/18614	0.138451	0.451993	0.405689	SIRT6	1
GO:190149	BP	GO:190149	positive re	1/902	3/18614	0.138451	0.451993	0.405689	VEGFC	1
GO:190150	BP	GO:190150	positive re	1/902	3/18614	0.138451	0.451993	0.405689	DENND5B	1
GO:190221	BP	GO:190221	regulation	1/902	3/18614	0.138451	0.451993	0.405689	PTPN2	1
GO:190222	BP	GO:190222	negative re	1/902	3/18614	0.138451	0.451993	0.405689	PTPN2	1
GO:190223	BP	GO:190223	regulation	1/902	3/18614	0.138451	0.451993	0.405689	PTPN2	1
GO:190225	BP	GO:190225	regulation	1/902	3/18614	0.138451	0.451993	0.405689	TGFB2	1
GO:190226	BP	GO:190226	negative re	1/902	3/18614	0.138451	0.451993	0.405689	OAZ1	1
GO:190263	BP	GO:190263	regulation	1/902	3/18614	0.138451	0.451993	0.405689	SOD2	1
GO:190263	BP	GO:190263	1-phospha	1/902	3/18614	0.138451	0.451993	0.405689	PIP4K2A	1
GO:190263	BP	GO:190263	1-phospha	1/902	3/18614	0.138451	0.451993	0.405689	PIP4K2A	1
GO:190281	BP	GO:190281	positive re	1/902	3/18614	0.138451	0.451993	0.405689	CYP26B1	1
GO:190287	BP	GO:190287	negative re	1/902	3/18614	0.138451	0.451993	0.405689	GDF11	1
GO:190295	BP	GO:190295	negative re	1/902	3/18614	0.138451	0.451993	0.405689	SNCA	1
GO:190295	BP	GO:190295	positive re	1/902	3/18614	0.138451	0.451993	0.405689	ISCU	1
GO:190301	BP	GO:190301	regulation	1/902	3/18614	0.138451	0.451993	0.405689	PTX3	1
GO:190307	BP	GO:190307	negative re	1/902	3/18614	0.138451	0.451993	0.405689	SVIP	1
GO:190348	BP	GO:190348	regulation	1/902	3/18614	0.138451	0.451993	0.405689	XBP1	1
GO:190365	BP	GO:190365	negative re	1/902	3/18614	0.138451	0.451993	0.405689	SNX12	1
GO:190374	BP	GO:190374	regulation	1/902	3/18614	0.138451	0.451993	0.405689	MAP2	1
GO:190402	BP	GO:190402	negative re	1/902	3/18614	0.138451	0.451993	0.405689	EMILIN1	1
GO:190423	BP	GO:190423	positive re	1/902	3/18614	0.138451	0.451993	0.405689	ISCU	1
GO:190442	BP	GO:190442	positive re	1/902	3/18614	0.138451	0.451993	0.405689	CLN5	1
GO:190443	BP	GO:190443	regulation	1/902	3/18614	0.138451	0.451993	0.405689	ISCU	1
GO:190458	BP	GO:190458	positive re	1/902	3/18614	0.138451	0.451993	0.405689	ZFP36L1	1
GO:190458	BP	GO:190458	cellular res	1/902	3/18614	0.138451	0.451993	0.405689	SLC5A5	1
GO:190461	BP	GO:190461	response to	1/902	3/18614	0.138451	0.451993	0.405689	AHR	1
GO:190469	BP	GO:190469	midbrain m	1/902	3/18614	0.138451	0.451993	0.405689	FZD6	1
GO:190471	BP	GO:190471	negative re	1/902	3/18614	0.138451	0.451993	0.405689	SNCA	1
GO:190487	BP	GO:190487	positive re	1/902	3/18614	0.138451	0.451993	0.405689	G6PD	1
GO:190488	BP	GO:190488	response to	1/902	3/18614	0.138451	0.451993	0.405689	NQO1	1
GO:190497	BP	GO:190497	lymphatic e	1/902	3/18614	0.138451	0.451993	0.405689	LGALS8	1
GO:190504	BP	GO:190504	regulation	1/902	3/18614	0.138451	0.451993	0.405689	DUSP10	1
GO:190533	BP	GO:190533	regulation	1/902	3/18614	0.138451	0.451993	0.405689	TYMP	1
GO:190541	BP	GO:190541	regulation	1/902	3/18614	0.138451	0.451993	0.405689	RAB3A	1
GO:190544	BP	GO:190544	negative re	1/902	3/18614	0.138451	0.451993	0.405689	SNCA	1
GO:190545	BP	GO:190545	positive re	1/902	3/18614	0.138451	0.451993	0.405689	ZNHIT1	1
GO:190555	BP	GO:190555	positive re	1/902	3/18614	0.138451	0.451993	0.405689	SIRT6	1
GO:190588	BP	GO:190588	positive re	1/902	3/18614	0.138451	0.451993	0.405689	DENND5B	1
GO:190591	BP	GO:190591	positive re	1/902	3/18614	0.138451	0.451993	0.405689	SOD2	1
GO:190593	BP	GO:190593	positive re	1/902	3/18614	0.138451	0.451993	0.405689	SOD2	1
GO:199010	BP	GO:199010	protein line	1/902	3/18614	0.138451	0.451993	0.405689	SPATA2	1
GO:199012	BP	GO:199012	messenger	1/902	3/18614	0.138451	0.451993	0.405689	CPSF6	1
GO:199039	BP	GO:199039	epithelium	1/902	3/18614	0.138451	0.451993	0.405689	DUSP10	1
GO:199041	BP	GO:199041	response to	1/902	3/18614	0.138451	0.451993	0.405689	XBP1	1
GO:199065	BP	GO:199065	sebum sec	1/902	3/18614	0.138451	0.451993	0.405689	GATA6	1
GO:200018	BP	GO:200018	regulation	1/902	3/18614	0.138451	0.451993	0.405689	DGKQ	1
GO:200022	BP	GO:200022	regulation	1/902	3/18614	0.138451	0.451993	0.405689	AKR1C3	1
GO:200029	BP	GO:200029	regulation	1/902	3/18614	0.138451	0.451993	0.405689	SNCA	1
GO:200059	BP	GO:200059	positive re	1/902	3/18614	0.138451	0.451993	0.405689	PDGFA	1
GO:200118	BP	GO:200118	negative re	1/902	3/18614	0.138451	0.451993	0.405689	LILRB1	1
GO:000189	BP	GO:000189	embryonic	7/902	89/18614	0.140158	0.456362	0.40961	ZFP36L1/R	7
GO:006019	BP	GO:006019	regulation	7/902	89/18614	0.140158	0.456362	0.40961	NR1H3/AR	7
GO:200017	BP	GO:200017	regulation	7/902	89/18614	0.140158	0.456362	0.40961	VEGFC/ID2	7
GO:000196	BP	GO:000196	synaptic tra	3/902	27/18614	0.140608	0.456362	0.40961	RASD2/SN	3
GO:000661	BP	GO:000661	SRP-deper	3/902	27/18614	0.140608	0.456362	0.40961	SRP9/SEC6	3
GO:000908	BP	GO:000908	branched-	3/902	27/18614	0.140608	0.456362	0.40961	HIBCH/BCI	3
GO:000965	BP	GO:000965	response to	3/902	27/18614	0.140608	0.456362	0.40961	ZFP36L1/S	3
GO:001080	BP	GO:001080	negative re	3/902	27/18614	0.140608	0.456362	0.40961	ZNF675/PT	3
GO:002154	BP	GO:002154	subpallium	3/902	27/18614	0.140608	0.456362	0.40961	FOXP1/RAI	3
GO:003311	BP	GO:003311	negative re	3/902	27/18614	0.140608	0.456362	0.40961	RBM20/SR	3
GO:004633	BP	GO:004633	diacylglyce	3/902	27/18614	0.140608	0.456362	0.40961	DGKQ/PLA	3
GO:009955	BP	GO:009955	maintenan	3/902	27/18614	0.140608	0.456362	0.40961	SORT1/DL	3
GO:000167	BP	GO:000167	cellular glu	11/902	157/18614	0.140947	0.456874	0.410069	ZNF236/S	11
GO:004859	BP	GO:004859	eye morph	11/902	157/18614	0.140947	0.456874	0.410069	MFSD2A/S	11
GO:000293	BP	GO:000293	response to	5/902	57/18614	0.141381	0.456874	0.410069	NPR3/NQ	5
GO:000674	BP	GO:000674	glutathione	5/902	57/18614	0.141381	0.456874	0.410069	GPX4/G6PI	5
GO:000682	BP	GO:000682	iron ion tra	5/902	57/18614	0.141381	0.456874	0.410069	ISCU/SLC2	5
GO:001067	BP	GO:001067	positive re	5/902	57/18614	0.141381	0.456874	0.410069	DYRK2/PM	5
GO:003112	BP	GO:003112	mRNA 3'-e	5/902	57/18614	0.141381	0.456874	0.410069	ZFP36L1/C	5
GO:004347	BP	GO:004347	regulation	5/902	57/18614	0.141381	0.456874	0.410069	HTR2A/SIR	5
GO:009017	BP	GO:009017	regulation	5/902	57/18614	0.141381	0.456874	0.410069	RSPO3/FZD	5
GO:004663	BP	GO:004663	regulation	6/902	73/18614	0.142029	0.458413	0.411451	NKAP/IL4R	6
GO:005193	BP	GO:005193	catecholan	6/902	73/18614	0.142029	0.458413	0.411451	SDHD/SYT	6
GO:007132	BP	GO:007132	cellular res	10/902	140/18614	0.142222	0.458413	0.411451	ZNF236/S	10
GO:000913	BP	GO:000913	purine nucl	8/902	106/18614	0.142269	0.458413	0.411451	HTR2A/PG	8
GO:000917	BP	GO:000917	purine ribo	8/902	106/18614	0.142269	0.458413	0.411451	HTR2A/PG	8
GO:190179	BP	GO:190179	regulation	8/902	106/18614	0.142269	0.458413	0.411451	DYRK2/PM	8
GO:004505	BP	GO:004505	regulated e	15/902	228/18614	0.142701	0.458558	0.411581	PTGDS/CA	15
GO:007190	BP	GO:007190	negative re	9/902	123/18614	0.142776	0.458558	0.411581	ZNF675/DU	9
GO:004663	BP	GO:004663	alpha-beta	12/902	175/18614	0.143272	0.458558	0.411581	IRF1/ITCH/	12
GO:001598	BP	GO:001598	energy der	21/902	337/18614	0.143468	0.458558	0.411581	GPD2/NDU	21
GO:000645	BP	GO:000645	'de novo' p	4/902	42/18614	0.144588	0.458558	0.411581	DNAJB14/H	4
GO:001059	BP	GO:001059	regulation	4/902	42/18614	0.144588	0.458558	0.411581	CAPZB/HS	4
GO:004562	BP	GO:004562	regulation	4/902	42/18614	0.144588	0.458558	0.411581	IL4R/JUNB	4
GO:005069	BP	GO:005069	regulation	4/902	42/18614	0.144588	0.458558	0.411581	TNFAIP3/H	4
GO:000246	BP	GO:000246	germinal c	2/902	14/18614	0.1454	0.458558	0.411581	TNFAIP3/B	2
GO:000320	BP	GO:000320	cardiac cha	2/902	14/18614	0.1454	0.458558	0.411581	SOX11/SO	2
GO:000322	BP	GO:000322	ventricular	2/902	14/18614	0.1454	0.458558	0.411581	TGFB2/ENG	2
GO:000646	BP	GO:000646	signal pept	2/902	14/18614	0.1454	0.458558	0.411581	SPCS3/CLN	2
GO:000659	BP	GO:000659	polyamine	2/902	14/18614	0.1454	0.458558	0.411581	PAOX/OAZ	2
GO:000721	BP	GO:000721	G protein-	2/902	14/18614	0.1454	0.458558	0.411581	GRM2/HO	2
GO:001071	BP	GO:001071	negative re	2/902	14/18614	0.1454	0.458558	0.411581	EMILIN1/C	2
GO:001567	BP	GO:001567	carbon dio	2/902	14/18614	0.1454	0.458558	0.411581	HBB/HBD	2
GO:002181	BP	GO:002181	layer forma	2/902	14/18614	0.1454	0.458558	0.411581	ADGRG1/C	2
GO:003111	BP	GO:003111	negative re	2/902	14/18614	0.1454	0.458558	0.411581	SNCA/MAF	2
GO:003126	BP	GO:003126	pseudopoc	2/902	14/18614	0.1454	0.458558	0.411581	SRGAP2/CI	2
GO:003166	BP	GO:003166	negative re	2/902	14/18614	0.1454	0.458558	0.411581	TNFAIP3/P	2
GO:003248	BP	GO:003248	Rap proteir	2/902	14/18614	0.1454	0.458558	0.411581	RAP2A/RAI	2
GO:003268	BP	GO:003268	negative re	2/902	14/18614	0.1454	0.458558	0.411581	LILRB1/REL	2
GO:003556	BP	GO:003556	positive re	2/902	14/18614	0.1454	0.458558	0.411581	MBD2/GM	2
GO:003584	BP	GO:003584	photorecep	2/902	14/18614	0.1454	0.458558	0.411581	MFSD2A/IF	2
GO:004254	BP	GO:004254	hemoglobi	2/902	14/18614	0.1454	0.458558	0.411581	SLC25A37/	2
GO:004320	BP	GO:004320	response to	2/902	14/18614	0.1454	0.458558	0.411581	SESN3/PIK	2
GO:004479	BP	GO:004479	negative re	2/902	14/18614	0.1454	0.458558	0.411581	PTX3/ZBED	2
GO:004618	BP	GO:004618	aldehyde c	2/902	14/18614	0.1454	0.458558	0.411581	PNKD/ADH	2
GO:004676	BP	GO:004676	viral buddi	2/902	14/18614	0.1454	0.458558	0.411581	CHMP5/CH	2
GO:004871	BP	GO:004871	negative re	2/902	14/18614	0.1454	0.458558	0.411581	DUSP10/CT	2
GO:005125	BP	GO:005125	spindle mic	2/902	14/18614	0.1454	0.458558	0.411581	LZTS2/RAC	2
GO:005154	BP	GO:005154	regulation	2/902	14/18614	0.1454	0.458558	0.411581	MAP4K4/M	2
GO:005164	BP	GO:005164	Golgi local	2/902	14/18614	0.1454	0.458558	0.411581	HOOK3/CD	2
GO:005501	BP	GO:005501	ventricular	2/902	14/18614	0.1454	0.458558	0.411581	RARB/RAR	2
GO:006104	BP	GO:006104	regulation	2/902	14/18614	0.1454	0.458558	0.411581	TNFAIP3/X	2
GO:007014	BP	GO:007014	response to	2/902	14/18614	0.1454	0.458558	0.411581	CCND1/MI	2
GO:007094	BP	GO:007094	neutrophil	2/902	14/18614	0.1454	0.458558	0.411581	TREM1/MY	2
GO:007136	BP	GO:007136	cellular res	2/902	14/18614	0.1454	0.458558	0.411581	TP53INP1/	2
GO:007216	BP	GO:007216	nephron tu	2/902	14/18614	0.1454	0.458558	0.411581	STAT1/CTN	2
GO:190102	BP	GO:190102	negative re	2/902	14/18614	0.1454	0.458558	0.411581	SLC35F6/G	2
GO:190294	BP	GO:190294	regulation	2/902	14/18614	0.1454	0.458558	0.411581	RB1/HSP90	2
GO:190372	BP	GO:190372	positive re	2/902	14/18614	0.1454	0.458558	0.411581	HTR2A/FAI	2
GO:190432	BP	GO:190432	response to	2/902	14/18614	0.1454	0.458558	0.411581	AHR/SLC5/	2
GO:190432	BP	GO:190432	cellular res	2/902	14/18614	0.1454	0.458558	0.411581	AHR/SLC5/	2
GO:190442	BP	GO:190442	regulation	2/902	14/18614	0.1454	0.458558	0.411581	CCPG1/CLI	2
GO:002240	BP	GO:002240	membrane	7/902	90/18614	0.146064	0.460434	0.413265	BLOC1S6/S	7
GO:007169	BP	GO:007169	anatomica	16/902	247/18614	0.146437	0.461394	0.414126	GREM1/DE	16
GO:000173	BP	GO:000173	establishm	6/902	74/18614	0.148665	0.467133	0.419277	RSPO3/FZD	6
GO:000328	BP	GO:000328	ventricular	6/902	74/18614	0.148665	0.467133	0.419277	TGFB2/SOX	6
GO:000716	BP	GO:000716	establishm	6/902	74/18614	0.148665	0.467133	0.419277	RSPO3/FZD	6
GO:200057	BP	GO:200057	positive re	6/902	74/18614	0.148665	0.467133	0.419277	GREM1/CY	6
GO:000276	BP	GO:000276	immune re	27/902	450/18614	0.148687	0.467133	0.419277	IRF1/ITCH/	27
GO:000974	BP	GO:000974	response to	13/902	194/18614	0.148817	0.467133	0.419277	ZNF236/S	13
GO:001715	BP	GO:001715	regulation	13/902	194/18614	0.148817	0.467133	0.419277	CADPS2/FE	13
GO:004566	BP	GO:004566	regulation	13/902	194/18614	0.148817	0.467133	0.419277	IRX3/MMD	13
GO:000641	BP	GO:000641	translation	5/902	58/18614	0.148968	0.467167	0.419308	ASCC3/CPI	5
GO:000676	BP	GO:000676	water-solu	5/902	58/18614	0.148968	0.467167	0.419308	AMN/ABCI	5
GO:002241	BP	GO:002241	cellular cor	29/902	488/18614	0.150119	0.470555	0.422349	MAP4K4/A	29
GO:001072	BP	GO:001072	positive re	26/902	432/18614	0.150428	0.471302	0.423019	ZFP36L1/D	26
GO:006002	BP	GO:006002	roof of mo	7/902	91/18614	0.152085	0.473359	0.424865	TGFB2/SUI	7
GO:200011	BP	GO:200011	negative re	7/902	91/18614	0.152085	0.473359	0.424865	DNAJB6/BI	7
GO:000030	BP	GO:000030	response to	3/902	28/18614	0.152146	0.473359	0.424865	NQO1/SOI	3
GO:000651	BP	GO:000651	protein qua	3/902	28/18614	0.152146	0.473359	0.424865	YME1L1/O	3
GO:001654	BP	GO:001654	protein aut	3/902	28/18614	0.152146	0.473359	0.424865	OMA1/FAN	3
GO:003166	BP	GO:003166	regulation	3/902	28/18614	0.152146	0.473359	0.424865	LY96/TNFA	3
GO:003270	BP	GO:003270	negative re	3/902	28/18614	0.152146	0.473359	0.424865	TNFAIP3/H	3
GO:004205	BP	GO:004205	negative re	3/902	28/18614	0.152146	0.473359	0.424865	DUSP3/PTI	3
GO:004568	BP	GO:004568	negative re	3/902	28/18614	0.152146	0.473359	0.424865	DUSP10/CT	3
GO:004633	BP	GO:004633	phosphatic	3/902	28/18614	0.152146	0.473359	0.424865	MFSD2A/A	3
GO:005187	BP	GO:005187	killing by h	3/902	28/18614	0.152146	0.473359	0.424865	TREM1/AP	3
GO:006047	BP	GO:006047	lung cell di	3/902	28/18614	0.152146	0.473359	0.424865	GATA6/CT	3
GO:006048	BP	GO:006048	lung epithe	3/902	28/18614	0.152146	0.473359	0.424865	GATA6/CT	3
GO:190305	BP	GO:190305	positive re	3/902	28/18614	0.152146	0.473359	0.424865	EMILIN1/R	3
GO:190582	BP	GO:190582	positive re	3/902	28/18614	0.152146	0.473359	0.424865	SMC4/RB1	3
GO:001081	BP	GO:001081	positive re	9/902	125/18614	0.15289	0.475132	0.426457	EMILIN1/M	9
GO:003296	BP	GO:003296	collagen m	8/902	108/18614	0.153209	0.475132	0.426457	EMILIN1/M	8
GO:000801	BP	GO:000801	regulation	14/902	213/18614	0.153489	0.475132	0.426457	IRX3/TGFB	14
GO:005087	BP	GO:005087	positive re	16/902	249/18614	0.153522	0.475132	0.426457	DUSP10/TI	16
GO:000690	BP	GO:000690	vesicle doc	4/902	43/18614	0.153711	0.475132	0.426457	BLOC1S6/V	4
GO:001090	BP	GO:001090	positive re	4/902	43/18614	0.153711	0.475132	0.426457	DYRK2/PM	4
GO:001660	BP	GO:001660	Rac proteir	4/902	43/18614	0.153711	0.475132	0.426457	ELMO1/DE	4
GO:003167	BP	GO:003167	cellular res	4/902	43/18614	0.153711	0.475132	0.426457	PDK2/XBP1	4
GO:004871	BP	GO:004871	regulation	4/902	43/18614	0.153711	0.475132	0.426457	DUSP10/RI	4
GO:005115	BP	GO:005115	regulation	4/902	43/18614	0.153711	0.475132	0.426457	ENG/SOD2	4
GO:006042	BP	GO:006042	lung epithe	4/902	43/18614	0.153711	0.475132	0.426457	GATA6/CT	4
GO:007176	BP	GO:007176	nuclear me	4/902	43/18614	0.153711	0.475132	0.426457	NEK6/CHM	4
GO:009011	BP	GO:009011	COPII-coa	4/902	43/18614	0.153711	0.475132	0.426457	TMED2/TB	4
GO:190211	BP	GO:190211	positive re	4/902	43/18614	0.153711	0.475132	0.426457	BID/SLC35	4
GO:004440	BP	GO:004440	entry into h	11/902	160/18614	0.154247	0.47657	0.427748	ITCH/TRIM	11
GO:000715	BP	GO:000715	leukocyte (	25/902	415/18614	0.154905	0.478381	0.429373	IRF1/ITCH/	25
GO:004205	BP	GO:004205	regulation	6/902	75/18614	0.155438	0.479583	0.430452	DUSP3/PTI	6
GO:004866	BP	GO:004866	negative re	6/902	75/18614	0.155438	0.479583	0.430452	IGFBP3/TN	6
GO:190390	BP	GO:190390	regulation	10/902	143/18614	0.156451	0.482214	0.432814	TRIM8/TRI	10
GO:000276	BP	GO:000276	positive re	5/902	59/18614	0.156723	0.482214	0.432814	ZFP36L1/ID	5
GO:000661	BP	GO:000661	protein exp	5/902	59/18614	0.156723	0.482214	0.432814	SIRT6/UHN	5
GO:001025	BP	GO:001025	NADH deh	5/902	59/18614	0.156723	0.482214	0.432814	NDUFA5/N	5
GO:003298	BP	GO:003298	mitochond	5/902	59/18614	0.156723	0.482214	0.432814	NDUFA5/N	5
GO:007053	BP	GO:007053	protein K63	5/902	59/18614	0.156723	0.482214	0.432814	ITCH/TRAF	5
GO:000906	BP	GO:000906	aerobic res	13/902	196/18614	0.156948	0.482684	0.433236	NDUFA5/A	13
GO:002240	BP	GO:002240	regulation	29/902	491/18614	0.157742	0.484904	0.435228	EMILIN1/IF	29
GO:004547	BP	GO:004547	response to	9/902	126/18614	0.158076	0.485255	0.435543	CCND1/G6	9
GO:000042	BP	GO:000042	autophagy	7/902	92/18614	0.158219	0.485255	0.435543	SPATA18/(	7
GO:000165	BP	GO:000165	metanephr	7/902	92/18614	0.158219	0.485255	0.435543	GREM1/IRX	7
GO:000979	BP	GO:000979	post-embr	7/902	92/18614	0.158219	0.485255	0.435543	ATF5/SERP	7
GO:006172	BP	GO:006172	mitochond	7/902	92/18614	0.158219	0.485255	0.435543	SPATA18/(	7
GO:003214	BP	GO:003214	activation (	8/902	109/18614	0.158826	0.486044	0.436251	TGFB2/CHI	8
GO:004581	BP	GO:004581	negative re	8/902	109/18614	0.158826	0.486044	0.436251	CBX3/MBD	8
GO:003025	BP	GO:003025	lipid modif	13/902	197/18614	0.161099	0.486044	0.436251	CYGB/MFS	13
GO:004665	BP	GO:004665	lymphocyte	19/902	306/18614	0.16137	0.486044	0.436251	IRF1/ITCH/	19
GO:000691	BP	GO:000691	activation (	6/902	76/18614	0.162344	0.486044	0.436251	RPS27L/PM	6
GO:000916	BP	GO:000916	nucleotide	6/902	76/18614	0.162344	0.486044	0.436251	NTHL1/MB	6
GO:000267	BP	GO:000267	respiratory	2/902	15/18614	0.162637	0.486044	0.436251	DUSP10/PF	2
GO:000913	BP	GO:000913	pyrimidine	2/902	15/18614	0.162637	0.486044	0.436251	CMPK1/TY	2
GO:000926	BP	GO:000926	deoxyribor	2/902	15/18614	0.162637	0.486044	0.436251	ADK/TYMS	2
GO:000926	BP	GO:000926	2'-deoxyrib	2/902	15/18614	0.162637	0.486044	0.436251	ADK/TYMS	2
GO:001064	BP	GO:001064	regulation	2/902	15/18614	0.162637	0.486044	0.436251	IRX3/HRC	2
GO:001086	BP	GO:001086	positive re	2/902	15/18614	0.162637	0.486044	0.436251	NR1H3/MF	2
GO:001951	BP	GO:001951	peptidyl-p	2/902	15/18614	0.162637	0.486044	0.436251	P4HA1/P4H	2
GO:003126	BP	GO:003126	pseudopoc	2/902	15/18614	0.162637	0.486044	0.436251	SRGAP2/CI	2
GO:003145	BP	GO:003145	positive re	2/902	15/18614	0.162637	0.486044	0.436251	ZNF304/DI	2
GO:003368	BP	GO:003368	negative re	2/902	15/18614	0.162637	0.486044	0.436251	GREM1/SM	2
GO:003431	BP	GO:003431	primary alc	2/902	15/18614	0.162637	0.486044	0.436251	AKR1C3/SU	2
GO:004310	BP	GO:004310	purine-cor	2/902	15/18614	0.162637	0.486044	0.436251	NAPRT/AD	2
GO:004332	BP	GO:004332	protein tra	2/902	15/18614	0.162637	0.486044	0.436251	STAM2/STA	2
GO:004583	BP	GO:004583	positive re	2/902	15/18614	0.162637	0.486044	0.436251	BID/GLRX	2
GO:004634	BP	GO:004634	amino suga	2/902	15/18614	0.162637	0.486044	0.436251	CHI3L1/MC	2
GO:004638	BP	GO:004638	deoxyribos	2/902	15/18614	0.162637	0.486044	0.436251	ADK/TYMS	2
GO:004675	BP	GO:004675	non-lytic v	2/902	15/18614	0.162637	0.486044	0.436251	CHMP5/CH	2
GO:004826	BP	GO:004826	clathrin coa	2/902	15/18614	0.162637	0.486044	0.436251	PIK3C2A/A	2
GO:004882	BP	GO:004882	hair follicle	2/902	15/18614	0.162637	0.486044	0.436251	TGFB2/CTN	2
GO:006041	BP	GO:006041	atrial septu	2/902	15/18614	0.162637	0.486044	0.436251	TGFB2/SOX	2
GO:006076	BP	GO:006076	negative re	2/902	15/18614	0.162637	0.486044	0.436251	FOXP1/ZBT	2
GO:007036	BP	GO:007036	hepatocyte	2/902	15/18614	0.162637	0.486044	0.436251	HNF1B/AN	2
GO:007088	BP	GO:007088	negative re	2/902	15/18614	0.162637	0.486044	0.436251	DYRK2/HO	2
GO:007214	BP	GO:007214	epithelial c	2/902	15/18614	0.162637	0.486044	0.436251	RARA/NR2	2
GO:007249	BP	GO:007249	embryonic	2/902	15/18614	0.162637	0.486044	0.436251	SHOX2/CT	2
GO:009011	BP	GO:009011	COPII-coa	2/902	15/18614	0.162637	0.486044	0.436251	TBC1D20/S	2
GO:010605	BP	GO:010605	negative re	2/902	15/18614	0.162637	0.486044	0.436251	DYRK2/HO	2
GO:011015	BP	GO:011015	methylgua	2/902	15/18614	0.162637	0.486044	0.436251	CNOT7/NU	2
GO:012026	BP	GO:012026	positive re	2/902	15/18614	0.162637	0.486044	0.436251	ZNF304/DI	2
GO:190010	BP	GO:190010	negative re	2/902	15/18614	0.162637	0.486044	0.436251	HSPA5/DN	2
GO:190354	BP	GO:190354	positive re	2/902	15/18614	0.162637	0.486044	0.436251	STAM/RAB	2
GO:190438	BP	GO:190438	endoplasm	2/902	15/18614	0.162637	0.486044	0.436251	SYVN1/RN	2
GO:200067	BP	GO:200067	regulation	2/902	15/18614	0.162637	0.486044	0.436251	ZPR1/CRLF	2
GO:003532	BP	GO:003532	hippo signa	4/902	44/18614	0.163043	0.486044	0.436251	MOB1A/SO	4
GO:004218	BP	GO:004218	ketone bio	4/902	44/18614	0.163043	0.486044	0.436251	STARD3/D	4
GO:004502	BP	GO:004502	early endo	4/902	44/18614	0.163043	0.486044	0.436251	HOOK3/SN	4

GO:005085	BP	GO:005085	regulation	4/902	44/18614	0.163043	0.486044	0.436251	DUSP3/PTI	4
GO:005160	BP	GO:005160	response t	4/902	44/18614	0.163043	0.486044	0.436251	NQO1/SOI	4
GO:009014	BP	GO:009014	membrane	4/902	44/18614	0.163043	0.486044	0.436251	CHMP5/ST	4
GO:200051	BP	GO:200051	positive re	4/902	44/18614	0.163043	0.486044	0.436251	IL4R/CD83	4
GO:000170	BP	GO:000170	formation	9/902	127/18614	0.163346	0.486044	0.436251	GATA6/FZI	9
GO:003560	BP	GO:003560	protein de	9/902	127/18614	0.163346	0.486044	0.436251	RCOR1/ME	9
GO:004859	BP	GO:004859	camera-ty	9/902	127/18614	0.163346	0.486044	0.436251	MFSD2A/S	9
GO:000341	BP	GO:000341	endochonc	3/902	29/18614	0.16397	0.486044	0.436251	RARB/TSKU	3
GO:000964	BP	GO:000964	photoperic	3/902	29/18614	0.16397	0.486044	0.436251	PPP1CB/ID	3
GO:001059	BP	GO:001059	positive re	3/902	29/18614	0.16397	0.486044	0.436251	HSP90AA1	3
GO:001907	BP	GO:001907	viral releas	3/902	29/18614	0.16397	0.486044	0.436251	CHMP5/CH	3
GO:002195	BP	GO:002195	central ner	3/902	29/18614	0.16397	0.486044	0.436251	WDR47/CD	3
GO:003157	BP	GO:003157	mitotic G1	3/902	29/18614	0.16397	0.486044	0.436251	CCND1/RP	3
GO:003450	BP	GO:003450	centromere	3/902	29/18614	0.16397	0.486044	0.436251	RB1/SIRT6/	3
GO:003589	BP	GO:003589	exit from h	3/902	29/18614	0.16397	0.486044	0.436251	CHMP5/CH	3
GO:004434	BP	GO:004434	type B pan	3/902	29/18614	0.16397	0.486044	0.436251	IGFBP3/DA	3
GO:004481	BP	GO:004481	mitotic G1/	3/902	29/18614	0.16397	0.486044	0.436251	CCND1/RP	3
GO:190364	BP	GO:190364	regulation	3/902	29/18614	0.16397	0.486044	0.436251	SNX12/RAI	3
GO:000167	BP	GO:000167	long-chain	8/902	110/18614	0.164538	0.486044	0.436251	PTGDS/CYI	8
GO:001071	BP	GO:001071	regulation	8/902	110/18614	0.164538	0.486044	0.436251	GREM1/TG	8
GO:001481	BP	GO:001481	muscle cell	8/902	110/18614	0.164538	0.486044	0.436251	IGFBP3/CY	8
GO:000317	BP	GO:000317	heart valve	5/902	60/18614	0.16464	0.486044	0.436251	EMILIN1/T	5
GO:003200	BP	GO:003200	negative re	5/902	60/18614	0.16464	0.486044	0.436251	SESN3/TBC	5
GO:004657	BP	GO:004657	positive re	5/902	60/18614	0.16464	0.486044	0.436251	MAP4K4/LI	5
GO:007170	BP	GO:007170	membrane	5/902	60/18614	0.16464	0.486044	0.436251	MAFB/CHN	5
GO:005134	BP	GO:005134	negative re	21/902	345/18614	0.168067	0.486044	0.436251	TFPI/FICD/	21
GO:005110	BP	GO:005110	negative re	11/902	163/18614	0.168162	0.486044	0.436251	BAG2/ZNF	11
GO:001604	BP	GO:001604	cell growth	29/902	495/18614	0.168244	0.486044	0.436251	GREM1/IGI	29
GO:004341	BP	GO:004341	positive re	29/902	495/18614	0.168244	0.486044	0.436251	IGFBP3/GD	29
GO:004308	BP	GO:004308	regulation	22/902	364/18614	0.16936	0.486044	0.436251	MAP4K4/C	22
GO:004568	BP	GO:004568	regulation	6/902	77/18614	0.169379	0.486044	0.436251	DUSP10/ID	6
GO:009055	BP	GO:009055	regulation	6/902	77/18614	0.169379	0.486044	0.436251	PMAIP1/BI	6
GO:200051	BP	GO:200051	regulation	6/902	77/18614	0.169379	0.486044	0.436251	ITCH/IL4R/	6
GO:000974	BP	GO:000974	response t	13/902	199/18614	0.169567	0.486044	0.436251	ZNF236/SC	13
GO:004206	BP	GO:004206	wound hea	26/902	439/18614	0.169767	0.486044	0.436251	EMILIN1/T	26
GO:005125	BP	GO:005125	positive re	20/902	327/18614	0.169834	0.486044	0.436251	BLOC1S6/I	20
GO:190121	BP	GO:190121	regulation	20/902	327/18614	0.169834	0.486044	0.436251	CCND1/TG	20
GO:000307	BP	GO:000307	regulation	7/902	94/18614	0.170819	0.486044	0.436251	KCNK6/SC	7
GO:000663	BP	GO:000663	acyl-CoA r	7/902	94/18614	0.170819	0.486044	0.436251	PDK2/DLA	7
GO:001657	BP	GO:001657	histone de	7/902	94/18614	0.170819	0.486044	0.436251	RCOR1/ME	7
GO:003538	BP	GO:003538	thioester m	7/902	94/18614	0.170819	0.486044	0.436251	PDK2/DLA	7
GO:004603	BP	GO:004603	ADP metal	7/902	94/18614	0.170819	0.486044	0.436251	HTR2A/PG	7
GO:004663	BP	GO:004663	negative re	4/902	45/18614	0.172574	0.486044	0.436251	ITCH/IL4R/	4
GO:014071	BP	GO:014071	facultative	4/902	45/18614	0.172574	0.486044	0.436251	MBD2/SAN	4
GO:190268	BP	GO:190268	mitochond	4/902	45/18614	0.172574	0.486044	0.436251	BID/SLC35	4
GO:002202	BP	GO:002202	telencepha	5/902	61/18614	0.172713	0.486044	0.436251	SRGAP2/W	5
GO:003278	BP	GO:003278	positive re	5/902	61/18614	0.172713	0.486044	0.436251	ELL2/CDK1	5
GO:004566	BP	GO:004566	negative re	5/902	61/18614	0.172713	0.486044	0.436251	GREM1/TN	5
GO:004645	BP	GO:004645	icosanoid	5/902	61/18614	0.172713	0.486044	0.436251	PTGDS/PT	5
GO:004685	BP	GO:004685	phosphatic	5/902	61/18614	0.172713	0.486044	0.436251	SOCS6/PIK	5
GO:004639	BP	GO:004639	carboxylic	20/902	328/18614	0.173171	0.486044	0.436251	TRIB3/PTG	20
GO:000694	BP	GO:000694	striated mu	12/902	182/18614	0.173709	0.486044	0.436251	SUMO1/HI	12
GO:007124	BP	GO:007124	cellular res	13/902	200/18614	0.173883	0.486044	0.436251	SUMO1/SY	13
GO:000223	BP	GO:000223	response t	22/902	366/18614	0.175697	0.486044	0.436251	LY96/DUSI	22
GO:000706	BP	GO:000706	mitotic sist	3/902	30/18614	0.176056	0.486044	0.436251	PDS5A/NA	3
GO:000964	BP	GO:000964	entrainmer	3/902	30/18614	0.176056	0.486044	0.436251	PPP1CB/ID	3
GO:003136	BP	GO:003136	N-termina	3/902	30/18614	0.176056	0.486044	0.436251	NAA50/NT	3
GO:003406	BP	GO:003406	protein loc	3/902	30/18614	0.176056	0.486044	0.436251	ARL5A/ARI	3
GO:007049	BP	GO:007049	interleukin	3/902	30/18614	0.176056	0.486044	0.436251	ZNF675/M	3
GO:190179	BP	GO:190179	positive re	3/902	30/18614	0.176056	0.486044	0.436251	PMAIP1/ZN	3
GO:000007	BP	GO:000007	regulation	8/902	112/18614	0.176242	0.486044	0.436251	CCND1/TN	8
GO:004217	BP	GO:004217	negative re	8/902	112/18614	0.176242	0.486044	0.436251	MAP1A/SV	8
GO:007037	BP	GO:007037	negative re	6/902	78/18614	0.176539	0.486044	0.436251	EMILIN1/D	6
GO:004648	BP	GO:004648	glycerolipi	23/902	385/18614	0.176637	0.486044	0.436251	SOCS6/NR	23
GO:000762	BP	GO:000762	locomotor	13/902	201/18614	0.178253	0.486044	0.436251	CSTB/WDF	13
GO:000926	BP	GO:000926	response t	12/902	183/18614	0.178298	0.486044	0.436251	SUMO1/HS	12
GO:000183	BP	GO:000183	trophectoc	1/902	4/18614	0.180207	0.486044	0.436251	ZPR1	1
GO:000192	BP	GO:000192	B-1 B cell	1/902	4/18614	0.180207	0.486044	0.436251	TNFAIP3	1
GO:000219	BP	GO:000219	cap-indep	1/902	4/18614	0.180207	0.486044	0.436251	RBM4	1
GO:000239	BP	GO:000239	MHC class	1/902	4/18614	0.180207	0.486044	0.436251	CALR	1
GO:000250	BP	GO:000250	peptide an	1/902	4/18614	0.180207	0.486044	0.436251	CALR	1
GO:000260	BP	GO:000260	negative re	1/902	4/18614	0.180207	0.486044	0.436251	CD68	1
GO:000327	BP	GO:000327	cell migrati	1/902	4/18614	0.180207	0.486044	0.436251	ENG	1
GO:000340	BP	GO:000340	regulation	1/902	4/18614	0.180207	0.486044	0.436251	SAR1B	1
GO:000622	BP	GO:000622	UDP biosy	1/902	4/18614	0.180207	0.486044	0.436251	CMPK1	1
GO:000623	BP	GO:000623	dTTP biosy	1/902	4/18614	0.180207	0.486044	0.436251	TYMS	1
GO:000640	BP	GO:000640	snRNA exp	1/902	4/18614	0.180207	0.486044	0.436251	PHAX	1
GO:000657	BP	GO:000657	valine cata	1/902	4/18614	0.180207	0.486044	0.436251	HIBCH	1
GO:000659	BP	GO:000659	polyamine	1/902	4/18614	0.180207	0.486044	0.436251	PAOX	1
GO:000829	BP	GO:000829	spermidine	1/902	4/18614	0.180207	0.486044	0.436251	PAOX	1
GO:000859	BP	GO:000859	regulation	1/902	4/18614	0.180207	0.486044	0.436251	PELI3	1
GO:000907	BP	GO:000907	aromatic a	1/902	4/18614	0.180207	0.486044	0.436251	PCBD2	1
GO:000917	BP	GO:000917	pyrimidine	1/902	4/18614	0.180207	0.486044	0.436251	TYMS	1
GO:000919	BP	GO:000919	pyrimidine	1/902	4/18614	0.180207	0.486044	0.436251	CMPK1	1
GO:000921	BP	GO:000921	pyrimidine	1/902	4/18614	0.180207	0.486044	0.436251	TYMS	1
GO:000975	BP	GO:000975	response t	1/902	4/18614	0.180207	0.486044	0.436251	AKR1C3	1
GO:000979	BP	GO:000979	regulation	1/902	4/18614	0.180207	0.486044	0.436251	CTNNB1	1
GO:001002	BP	GO:001002	wax biosyn	1/902	4/18614	0.180207	0.486044	0.436251	FAR1	1
GO:001004	BP	GO:001004	response t	1/902	4/18614	0.180207	0.486044	0.436251	G6PD	1
GO:001016	BP	GO:001016	wax metab	1/902	4/18614	0.180207	0.486044	0.436251	FAR1	1
GO:001062	BP	GO:001062	negative re	1/902	4/18614	0.180207	0.486044	0.436251	SUMO1	1
GO:001090	BP	GO:001090	regulation	1/902	4/18614	0.180207	0.486044	0.436251	CTNNB1	1
GO:001482	BP	GO:001482	vein smoot	1/902	4/18614	0.180207	0.486044	0.436251	EDNRB	1
GO:001483	BP	GO:001483	myoblast r	1/902	4/18614	0.180207	0.486044	0.436251	ANXA1	1
GO:001591	BP	GO:001591	long-chain	1/902	4/18614	0.180207	0.486044	0.436251	ABCD4	1
GO:001610	BP	GO:001610	diterpenoi	1/902	4/18614	0.180207	0.486044	0.436251	CYP26B1	1
GO:001807	BP	GO:001807	N-termina	1/902	4/18614	0.180207	0.486044	0.436251	SOX4	1
GO:001820	BP	GO:001820	peptidyl-g	1/902	4/18614	0.180207	0.486044	0.436251	NTMT1	1
GO:001974	BP	GO:001974	regulation	1/902	4/18614	0.180207	0.486044	0.436251	AKR1C3	1
GO:002202	BP	GO:002202	interkinetic	1/902	4/18614	0.180207	0.486044	0.436251	HOOK3	1
GO:003248	BP	GO:003248	Ral protein	1/902	4/18614	0.180207	0.486044	0.436251	RGL2	1
GO:003249	BP	GO:003249	detection	1/902	4/18614	0.180207	0.486044	0.436251	LY96	1
GO:003290	BP	GO:003290	transformi	1/902	4/18614	0.180207	0.486044	0.436251	SMAD3	1
GO:003291	BP	GO:003291	regulation	1/902	4/18614	0.180207	0.486044	0.436251	SMAD3	1
GO:003302	BP	GO:003302	mast cell h	1/902	4/18614	0.180207	0.486044	0.436251	KITLG	1
GO:003303	BP	GO:003303	positive re	1/902	4/18614	0.180207	0.486044	0.436251	ANXA1	1
GO:003313	BP	GO:003313	positive re	1/902	4/18614	0.180207	0.486044	0.436251	PFKFB1	1
GO:003336	BP	GO:003336	protein loc	1/902	4/18614	0.180207	0.486044	0.436251	SRGN	1
GO:003412	BP	GO:003412	regulation	1/902	4/18614	0.180207	0.486044	0.436251	IRF1	1
GO:003455	BP	GO:003455	respiratory	1/902	4/18614	0.180207	0.486044	0.436251	SDHAF2	1
GO:003455	BP	GO:003455	mitochond	1/902	4/18614	0.180207	0.486044	0.436251	SDHAF2	1
GO:003465	BP	GO:003465	retinoic aci	1/902	4/18614	0.180207	0.486044	0.436251	CYP26B1	1
GO:003475	BP	GO:003475	regulation	1/902	4/18614	0.180207	0.486044	0.436251	ISCU	1
GO:003524	BP	GO:003524	peptidyl-a	1/902	4/18614	0.180207	0.486044	0.436251	NDUFAF7	1
GO:003528	BP	GO:003528	central ner	1/902	4/18614	0.180207	0.486044	0.436251	MAFB	1
GO:003578	BP	GO:003578	metanephr	1/902	4/18614	0.180207	0.486044	0.436251	PDGFA	1
GO:003588	BP	GO:003588	embryonic	1/902	4/18614	0.180207	0.486044	0.436251	FZD6	1
GO:003610	BP	GO:003610	leukotriene	1/902	4/18614	0.180207	0.486044	0.436251	PTGR1	1
GO:003621	BP	GO:003621	response t	1/902	4/18614	0.180207	0.486044	0.436251	SH2B3	1
GO:003621	BP	GO:003621	cellular res	1/902	4/18614	0.180207	0.486044	0.436251	SH2B3	1
GO:003802	BP	GO:003802	insulin rece	1/902	4/18614	0.180207	0.486044	0.436251	PIK3CA	1
GO:003809	BP	GO:003809	sequesteri	1/902	4/18614	0.180207	0.486044	0.436251	GREM1	1
GO:003810	BP	GO:003810	Kit signalin	1/902	4/18614	0.180207	0.486044	0.436251	SH2B3	1
GO:003818	BP	GO:003818	neuropilin	1/902	4/18614	0.180207	0.486044	0.436251	CDC42	1
GO:003953	BP	GO:003953	negative re	1/902	4/18614	0.180207	0.486044	0.436251	RIOK3	1
GO:004242	BP	GO:004242	choline cat	1/902	4/18614	0.180207	0.486044	0.436251	SLC44A1	1
GO:004263	BP	GO:004263	catagen	1/902	4/18614	0.180207	0.486044	0.436251	TGFB2	1
GO:004299	BP	GO:004299	negative re	1/902	4/18614	0.180207	0.486044	0.436251	CSK	1
GO:004331	BP	GO:004331	positive re	1/902	4/18614	0.180207	0.486044	0.436251	CD177	1
GO:004362	BP	GO:004362	cortical mi	1/902	4/18614	0.180207	0.486044	0.436251	DLG1	1
GO:004506	BP	GO:004506	cytotoxic T	1/902	4/18614	0.180207	0.486044	0.436251	TNFSF9	1
GO:004544	BP	GO:004544	mitotic cell	1/902	4/18614	0.180207	0.486044	0.436251	CTNNB1	1
GO:004562	BP	GO:004562	negative re	1/902	4/18614	0.180207	0.486044	0.436251	ANXA1	1
GO:004576	BP	GO:004576	negative re	1/902	4/18614	0.180207	0.486044	0.436251	BCKDK	1
GO:004607	BP	GO:004607	dTTP meta	1/902	4/18614	0.180207	0.486044	0.436251	TYMS	1
GO:004629	BP	GO:004629	formaldeh	1/902	4/18614	0.180207	0.486044	0.436251	ADH5	1
GO:004633	BP	GO:004633	phosphatic	1/902	4/18614	0.180207	0.486044	0.436251	PLA2G15	1
GO:004672	BP	GO:004672	positive re	1/902	4/18614	0.180207	0.486044	0.436251	TBC1D20	1
GO:004696	BP	GO:004696	cytosol to	1/902	4/18614	0.180207	0.486044	0.436251	TAP1	1
GO:005074	BP	GO:005074	positive re	1/902	4/18614	0.180207	0.486044	0.436251	SVIP	1
GO:005097	BP	GO:005097	sensory pe	1/902	4/18614	0.180207	0.486044	0.436251	RAB3A	1
GO:005159	BP	GO:005159	methylglyc	1/902	4/18614	0.180207	0.486044	0.436251	PNKD	1
GO:005161	BP	GO:005161	regulation	1/902	4/18614	0.180207	0.486044	0.436251	SNCA	1
GO:005179	BP	GO:005179	regulation	1/902	4/18614	0.180207	0.486044	0.436251	TGFB2	1
GO:005189	BP	GO:005189	positive re	1/902	4/18614	0.180207	0.486044	0.436251	TGFB2	1
GO:005240	BP	GO:005240	negative re	1/902	4/18614	0.180207	0.486044	0.436251	PTX3	1
GO:006024	BP	GO:006024	negative re	1/902	4/18614	0.180207	0.486044	0.436251	DACH1	1
GO:006043	BP	GO:006043	primary lur	1/902	4/18614	0.180207	0.486044	0.436251	CTNNB1	1
GO:006046	BP	GO:006046	pharynx de	1/902	4/18614	0.180207	0.486044	0.436251	EDNRB	1
GO:006048	BP	GO:006048	club cell di	1/902	4/18614	0.180207	0.486044	0.436251	GATA6	1
GO:006093	BP	GO:006093	His-Purkinj	1/902	4/18614	0.180207	0.486044	0.436251	IRX3	1
GO:006103	BP	GO:006103	epithelial c	1/902	4/18614	0.180207	0.486044	0.436251	ID2	1
GO:006107	BP	GO:006107	ciliary body	1/902	4/18614	0.180207	0.486044	0.436251	TSKU	1
GO:006118	BP	GO:006118	negative re	1/902	4/18614	0.180207	0.486044	0.436251	PTHLH	1
GO:006132	BP	GO:006132	cell prolife	1/902	4/18614	0.180207	0.486044	0.436251	CTNNB1	1
GO:006172	BP	GO:006172	methylglyc	1/902	4/18614	0.180207	0.486044	0.436251	PNKD	1
GO:007042	BP	GO:007042	regulation	1/902	4/18614	0.180207	0.486044	0.436251	TNFAIP3	1
GO:007060	BP	GO:007060	regulation	1/902	4/18614	0.180207	0.486044	0.436251	CTNNB1	1
GO:007066	BP	GO:007066	positive re	1/902	4/18614	0.180207	0.486044	0.436251	KITLG	1
GO:007067	BP	GO:007067	intralumen	1/902	4/18614	0.180207	0.486044	0.436251	RAB7A	1
GO:007121	BP	GO:007121	protein tar	1/902	4/18614	0.180207	0.486044	0.436251	LAMP2	1
GO:007122	BP	GO:007122	cellular res	1/902	4/18614	0.180207	0.486044	0.436251	TXNIP	1
GO:007139	BP	GO:007139	cellular res	1/902	4/18614	0.180207	0.486044	0.436251	AKR1C3	1
GO:007160	BP	GO:007160	endothelia	1/902	4/18614	0.180207	0.486044	0.436251	CYP1B1	1
GO:007179	BP	GO:007179	response t	1/902	4/18614	0.180207	0.486044	0.436251	AKR1C3	1
GO:007179	BP	GO:007179	cellular res	1/902	4/18614	0.180207	0.486044	0.436251	AKR1C3	1
GO:007196	BP	GO:007196	mitotic sist	1/902	4/18614	0.180207	0.486044	0.436251	NAA50	1
GO:007203	BP	GO:007203	regulation	1/902	4/18614	0.180207	0.486044	0.436251	HNF1B	1
GO:007204	BP	GO:007204	negative re	1/902	4/18614	0.180207	0.486044	0.436251	HNF1B	1
GO:007208	BP	GO:007208	specificatic	1/902	4/18614	0.180207	0.486044	0.436251	IRX3	1
GO:007209	BP	GO:007209	regulation	1/902	4/18614	0.180207	0.486044	0.436251	HNF1B	1
GO:007218	BP	GO:007218	mesoneph	1/902	4/18614	0.180207	0.486044	0.436251	HNF1B	1
GO:007242	BP	GO:007242	response t	1/902	4/18614	0.180207	0.486044	0.436251	EME1	1
GO:007256	BP	GO:007256	type B pan	1/902	4/18614	0.180207	0.486044	0.436251	GDF11	1
GO:007271	BP	GO:007271	cellular res	1/902	4/18614	0.180207	0.486044	0.436251	RPL23	1
GO:007275	BP	GO:007275	cellular res	1/902	4/18614	0.180207	0.486044	0.436251	SPIDR	1
GO:008601	BP	GO:008601	Purkinje m	1/902	4/18614	0.180207	0.486044	0.436251	SCN5A	1
GO:009018	BP	GO:009018	negative re	1/902	4/18614	0.180207	0.486044	0.436251	NR1H3	1
GO:009021	BP	GO:009021	regulation	1/902	4/18614	0.180207	0.486044	0.436251	PIP4K2A	1
GO:009023	BP	GO:009023	regulation	1/902	4/18614	0.180207	0.486044	0.436251	RB1	1
GO:009701	BP	GO:009701	renal albur	1/902	4/18614	0.180207	0.486044	0.436251	EDNRB	1
GO:009708	BP	GO:009708	methyl-bra	1/902	4/18614	0.180207	0.486044	0.436251	SLC27A2	1
GO:009711	BP	GO:009711	gamma-ar	1/902	4/18614	0.180207	0.486044	0.436251	GLRB	1
GO:009716	BP	GO:009716	circadian r	1/902	4/18614	0.180207	0.486044	0.436251	RBM4	1
GO:009770	BP	GO:009770	ferroptosis	1/902	4/18614	0.180207	0.486044	0.436251	GPX4	1
GO:009917	BP	GO:009917	regulation	1/902	4/18614	0.180207	0.486044	0.436251	LRFN3	1
GO:009919	BP	GO:009919	trans-syna	1/902	4/18614	0.180207	0.486044	0.436251	NTRK2	1
GO:011007	BP	GO:011007	regulation	1/902	4/18614	0.180207	0.486044	0.436251	GPX4	1
GO:012022	BP	GO:012022	regulation	1/902	4/18614	0.180207	0.486044	0.436251	TTLL4	1
GO:014015	BP	GO:014015	ammoniun	1/902	4/18614	0.180207	0.486044	0.436251	SLC12A6	1
GO:015009	BP	GO:015009	glial cell-n	1/902	4/18614	0.180207	0.486044	0.436251	CTNNB1	1
GO:190008	BP	GO:190008	regulation	1/902	4/18614	0.180207	0.486044	0.436251	GREM1	1
GO:190013	BP	GO:190013	negative re	1/902	4/18614	0.180207	0.486044	0.436251	ANXA1	1
GO:190036	BP	GO:190036	positive re	1/902	4/18614	0.180207	0.486044	0.436251	CPEB3	1
GO:190114	BP	GO:190114	insulin cata	1/902	4/18614	0.180207	0.486044	0.436251	CTSD	1
GO:190114	BP	GO:190114	mesenchyr	1/902	4/18614	0.180207	0.486044	0.436251	HNF1B	1
GO:190150	BP	GO:190150	regulation	1/902	4/18614	0.180207	0.486044	0.436251	DENND5B	1
GO:190161	BP	GO:190161	positive re	1/902	4/18614	0.180207	0.486044	0.436251	MAP2	1
GO:190220	BP	GO:190220	regulation	1/902	4/18614	0.180207	0.486044	0.436251	PTPN2	1
GO:190280	BP	GO:190280	regulation	1/902	4/18614	0.180207	0.486044	0.436251	CYP26B1	1
GO:190294	BP	GO:190294	negative re	1/902	4/18614	0.180207	0.486044	0.436251	RB1	1
GO:190295	BP	GO:190295	positive re	1/902	4/18614	0.180207	0.486044	0.436251	TBC1D20	1
GO:190306	BP	GO:190306	positive re	1/902	4/18614	0.180207	0.486044	0.436251	SVIP	1
GO:190323	BP	GO:190323	regulation	1/902	4/18614	0.180207	0.486044	0.436251	RAB3A	1
GO:190345	BP	GO:190345	regulation	1/902	4/18614	0.180207	0.486044	0.436251	RPL23	1
GO:190354	BP	GO:190354	negative re	1/902	4/18614	0.180207	0.486044	0.436251	RAB7A	1
GO:190385	BP	GO:190385	regulation	1/902	4/18614	0.180207	0.486044	0.436251	OMA1	1
GO:190389	BP	GO:190389	regulation	1/902	4/18614	0.180207	0.486044	0.436251	HSPA5	1
GO:190389	BP	GO:190389	positive re	1/902	4/18614	0.180207	0.486044	0.436251	PTPN2	1
GO:190392	BP	GO:190392	response t	1/902	4/18614	0.180207	0.486044	0.436251	DNMT1	1
GO:190392	BP	GO:190392	cellular res	1/902	4/18614	0.180207	0.486044	0.436251	DNMT1	1
GO:190402	BP	GO:190402	positive re	1/902	4/18614	0.180207	0.486044	0.436251	RB1	1
GO:190423	BP	GO:190423	regulation	1/902	4/18614	0.180207	0.486044	0.436251	ISCU	1
GO:190458	BP	GO:190458	regulation	1/902	4/18614	0.180207	0.486044	0.436251	ZFP36L1	1
GO:190477	BP	GO:190477	response t	1/902	4/18614	0.180207	0.486044	0.436251	NQO1	1
GO:190484	BP	GO:190484	response t	1/902	4/18614	0.180207	0.486044	0.436251	NQO1	1
GO:190495	BP	GO:190495	canonical	1/902	4/18614	0.180207	0.486044	0.436251	CTNNB1	1
GO:190506	BP	GO:190506	allantois de	1/902	4/18614	0.180207	0.486044	0.436251	TMED2	1
GO:190522	BP	GO:190522	atrioventric	1/902	4/18614	0.180207	0.486044	0.436251	ENG	1
GO:190555	BP	GO:190555	regulation	1/902	4/18614	0.180207	0.486044	0.436251	SIRT6	1
GO:190568	BP	GO:190568	positive re	1/902	4/18614	0.180207	0.486044	0.436251	CASP7	1
GO:190588	BP	GO:190588	regulation	1/902	4/18614	0.180207	0.486044	0.436251	DENND5B	1
GO:190593	BP	GO:190593	negative re	1/902	4/18614	0.180207	0.486044	0.436251	PTGDS	1
GO:199004	BP	GO:199004	protein loc	1/902	4/18614	0.180207	0.486044	0.436251	AUP1	1
GO:199016	BP	GO:199016	protein K2	1/902	4/18614	0.180207	0.486044	0.436251	YOD1	1
GO:199096	BP	GO:199096	ATP gener	1/902	4/18614	0.180207	0.486044	0.436251	PARG	1
GO:200001	BP	GO:200001	regulation	1/902	4/18614	0.180207	0.486044	0.436251	CTNNB1	1
GO:200025	BP	GO:200025	negative re	1/902	4/18614	0.180207	0.486044	0.436251	PTGDS	1
GO:200046	BP	GO:200046	regulation	1/902	4/18614	0.180207	0.486044	0.436251	SNCA	1
GO:200058	BP	GO:200058	regulation	1/902	4/18614	0.180207	0.486044	0.436251	PDGFA	1
GO:200130	BP	GO:200130	lipoxin A4	1/902	4/18614	0.180207	0.486044	0.436251	PTGR1	1
GO:000675	BP	GO:000675	glutathione	2/902	16/18614	0.180213	0.486044	0.436251	GCLM/GCL	2
GO:001814	BP	GO:001814	keratan sul	2/902	16/18614	0.180213	0.486044	0.436251	B3GNT2/B	2
GO:002153	BP	GO:002153	cell prolife	2/902	16/18614	0.180213	0.486044	0.436251	ATF5/GPR3	2
GO:002192	BP	GO:002192	cell prolife	2/902	16/18614	0.180213	0.486044	0.436251	ATF5/GPR3	2
GO:002193	BP	GO:002193	cerebellar	2/902	16/18614	0.180213	0.486044	0.436251	ATF5/GPR3	2
GO:003057	BP	GO:003057	nuclear bo	2/902	16/18614	0.180213	0.486044	0.436251	SUMO1/ZF	2
GO:003085	BP	GO:003085	regulation	2/902	16/18614	0.180213	0.486044	0.436251	INPP5D/RA	2
GO:003413	BP	GO:003413	toll-like re	2/902	16/18614	0.180213	0.486044	0.436251	TNFAIP3/T	2
GO:003806	BP	GO:003806	collagen-a	2/902	16/18614	0.180213	0.486044	0.436251	ITGA11/CC	2
GO:004215	BP	GO:004215	lipoprotein	2/902	16/18614	0.180213	0.486044	0.436251	CTSD/LYPL	2
GO:004350	BP	GO:004350	negative re	2/902	16/18614	0.180213	0.486044	0.436251	ZNF675/DU	2
GO:004351	BP	GO:004351	positive re	2/902	16/18614	0.180213	0.486044	0.436251	PMAIP1/ZN	2
GO:004415	BP	GO:004415	histone H3	2/902	16/18614	0.180213	0.486044	0.436251	MBIP/DR1	2
GO:004484	BP	GO:004484	estrous cyc	2/902	16/18614	0.180213	0.486044	0.436251	CYP1B1/PT	2
GO:004557	BP	GO:004557	positive re	2/902	16/18614	0.180213	0.486044	0.436251	XBP1/INPP	2
GO:004572	BP	GO:004572	negative re	2/902	16/18614	0.180213	0.486044	0.436251	SIRT6/LEPI	2
GO:004853	BP	GO:004853	anatomica	2/902	16/18614	0.180213	0.486044	0.436251	HSPA5/HO	2
GO:005509	BP	GO:005509	phospholip	2/902	16/18614	0.180213	0.486044	0.436251	TLCD1/FAE	2
GO:007043	BP	GO:007043	nucleotide	2/902	16/18614	0.180213	0.486044	0.436251	TNFAIP3/H	2
GO:009709	BP	GO:009709	synaptic ve	2/902	16/18614	0.180213	0.486044	0.436251	CTNNB1/R	2
GO:009911	BP	GO:009911	tRNA 5'-er	2/902	16/18614	0.180213	0.486044	0.436251	RPP25/SSB	2
GO:190174	BP	GO:190174	positive re	2/902	16/18614	0.180213	0.486044	0.436251	GDF15/IL4	2
GO:190526	BP	GO:190526	positive re	2/902	16/18614	0.180213	0.486044	0.436251	ZNF304/DI	2
GO:200063	BP	GO:200063	positive re	2/902	16/18614	0.180213	0.486044	0.436251	FXR1/WTIF	2
GO:200121	BP	GO:200121	regulation	2/902	16/18614	0.180213	0.486044	0.436251	RAP1A/RIN	2
GO:200125	BP	GO:200125	regulation	2/902	16/18614	0.180213	0.486044	0.436251	SARAF/HO	2
GO:004690	BP	GO:004690	regulation	5/902	62/18614	0.180934	0.487793	0.437821	PMAIP1/BI	5
GO:000760	BP	GO:000760	visual perc	14/902	220/18614	0.181991	0.489676	0.439511	IRX5/RP2/(	14
GO:001071	BP	GO:001071	regulation	4/902	46/18614	0.182292	0.489676	0.439511	EMILIN1/M	4
GO:001083	BP	GO:001083	regulation	4/902	46/18614	0.182292	0.489676	0.439511	CYP26B1/S	4
GO:003051	BP	GO:003051	negative re	4/902	46/18614	0.182292	0.489676	0.439511	IFRD1/MAI	4
GO:003312	BP	GO:003312	positive re	4/902	46/18614	0.182292	0.489676	0.439511	RBM20/ZP	4
GO:003433	BP	GO:003433	cell junctio	4/902	46/18614	0.182292	0.489676	0.439511	CD177/SO	4
GO:004208	BP	GO:004208	T-helper 1	4/902	46/18614	0.182292	0.489676	0.439511	BCL3/IL4R/	4
GO:005501	BP	GO:005501	ventricular	4/902	46/18614	0.182292	0.489676	0.439511	TGFB2/EN	4
GO:200122	BP	GO:200122	regulation	4/902	46/18614	0.182292	0.489676	0.439511	SRGAP2/AI	4
GO:001605	BP	GO:001605	organic aci	20/902	331/18614	0.183388	0.492422	0.441976	TRIB3/PTG	20
GO:004591	BP	GO:004591	positive re	6/902	79/18614	0.18382	0.493039	0.44253	DYRK2/PM	6
GO:005068	BP	GO:005068	regulation	6/902	79/18614	0.18382	0.493039	0.44253	ITCH/TNFA	6
GO:004853	BP	GO:004853	hematopoi	7/902	96/18614	0.183839	0.493039	0.44253	MAFB/BCL	7
GO:005067	BP	GO:005067	regulation	15/902	239/18614	0.185073	0.495994	0.445182	IRF1/ITCH/	15
GO:003812	BP	GO:003812	ERBB signa	9/902	131/18614	0.185237	0.495994	0.445182	PIK3C2A/D	9
GO:007232	BP	GO:007232	monocarbo	9/902	131/18614	0.185237	0.495994	0.445182	MFSD2A/C	9
GO:009873	BP	GO:009873	macromole	9/902	131/18614	0.185237	0.495994	0.445182	RCOR1/ME	9
GO:004211	BP	GO:004211	B cell activ	17/902	276/18614	0.185859	0.497458	0.446496	ZFP36L1/A	17
GO:001593	BP	GO:001593	nucleobase	14/902	221/18614	0.186262	0.498339	0.447287	ZFP36L1/S	14
GO:007145	BP	GO:007145	cellular res	10/902	149/18614	0.186908	0.499868	0.448659	GATA6/ZFI	10

GO:014053	CC	GO:014053	endoplasm	22/931	126/19518	1.20E-07	9.64E-05	8.81E-05	EMC2/SPC	22
GO:000579	CC	GO:000579	endoplasm	22/931	132/19518	2.80E-07	0.000112	0.000103	ZDHHC20/	22
GO:003466	CC	GO:003466	endoplasm	6/931	11/19518	4.36E-06	0.001163	0.001062	PPIB/DNAJ	6
GO:000578	CC	GO:000578	endoplasm	34/931	312/19518	6.52E-06	0.001306	0.001193	IGFBP3/ER	34
GO:000576	CC	GO:000576	lysosomal	40/931	415/19518	2.02E-05	0.002505	0.002288	MMD/STAI	40
GO:009885	CC	GO:009885	lytic vacuo	40/931	415/19518	2.02E-05	0.002505	0.002288	MMD/STAI	40
GO:003311	CC	GO:003311	endoplasm	14/931	80/19518	2.28E-05	0.002505	0.002288	ZDHHC20/	14
GO:000825	CC	GO:000825	oligosacch	6/931	14/19518	2.50E-05	0.002505	0.002288	RPN1/OST	6
GO:000577	CC	GO:000577	vacuolar m	41/931	460/19518	9.46E-05	0.00842	0.00769	MMD/STAI	41
GO:000577	CC	GO:000577	autophagc	16/931	117/19518	0.000138	0.011052	0.010094	CHMP5/TN	16
GO:001250	CC	GO:001250	ER to Golg	11/931	63/19518	0.000173	0.012608	0.011515	CNIH3/LM	11
GO:001660	CC	GO:001660	nuclear spe	37/931	415/19518	0.000203	0.013545	0.012371	DUSP10/SN	37
GO:003557	CC	GO:003557	azurophil g	10/931	58/19518	0.000377	0.02322	0.021207	CD68/LAM	10
GO:000579	CC	GO:000579	smooth en	7/931	32/19518	0.000648	0.036442	0.033284	SYVN1/PPI	7
GO:003066	CC	GO:003066	coated ves	21/931	202/19518	0.000682	0.036442	0.033284	CNIH3/LM	21
GO:004247	CC	GO:004247	melanosor	14/931	112/19518	0.000887	0.041812	0.038188	RPN1/PPIB	14
GO:004877	CC	GO:004877	pigment gı	14/931	112/19518	0.000887	0.041812	0.038188	RPN1/PPIB	14
GO:004432	CC	GO:004432	endoplasm	6/931	25/19518	0.00094	0.041826	0.038201	SYVN1/ERI	6
GO:190472	CC	GO:190472	tertiary gra	9/931	55/19518	0.001083	0.043255	0.039506	TNFAIP6/C	9
GO:000576	CC	GO:000576	primary lys	17/931	155/19518	0.001188	0.043255	0.039506	FRK/CD68/	17
GO:003190	CC	GO:003190	late endos	17/931	155/19518	0.001188	0.043255	0.039506	MMD/STAI	17
GO:004258	CC	GO:004258	azurophil g	17/931	155/19518	0.001188	0.043255	0.039506	FRK/CD68/	17
GO:004258	CC	GO:004258	specific gra	17/931	160/19518	0.001682	0.058592	0.053514	FRK/CHI3L	17
GO:001710	CC	GO:001710	glutamate-	2/931	2/19518	0.002273	0.070023	0.063955	GCLM/GCL	2
GO:003468	CC	GO:003468	integrin alp	2/931	2/19518	0.002273	0.070023	0.063955	ITGB8/ITG/	2
GO:015000	CC	GO:015000	primary de	2/931	2/19518	0.002273	0.070023	0.063955	MAP1A/M/	2
GO:009057	CC	GO:009057	RNA polym	23/931	254/19518	0.002503	0.074258	0.067822	MAFB/ASC	23
GO:000042	CC	GO:000042	autophagc	8/931	51/19518	0.002673	0.076462	0.069835	CHMP5/TN	8
GO:003066	CC	GO:003066	secretory g	27/931	319/19518	0.002924	0.080773	0.073773	RAB31/DYI	27
GO:003557	CC	GO:003557	specific gra	11/931	91/19518	0.003972	0.10605	0.096859	RAP1A/CD	11
GO:003013	CC	GO:003013	COPII-coa	11/931	93/19518	0.004696	0.121339	0.110823	CNIH3/LM	11
GO:003356	CC	GO:003356	ESCRT-0 c	2/931	3/19518	0.006602	0.1511	0.138004	STAM2/ST/	2
GO:006179	CC	GO:006179	chromatin	2/931	3/19518	0.006602	0.1511	0.138004	CBX3/RB1	2
GO:007072	CC	GO:007072	ISGF3 com	2/931	3/19518	0.006602	0.1511	0.138004	STAT1/IRF	2
GO:190158	CC	GO:190158	dendritic m	2/931	3/19518	0.006602	0.1511	0.138004	BAG2/MAF	2
GO:003120	CC	GO:003120	SNARE cor	7/931	48/19518	0.007323	0.162945	0.148822	GOSR2/ST	7
GO:003013	CC	GO:003013	coated ves	25/931	312/19518	0.008098	0.171464	0.156604	SNX18/CN	25
GO:000577	CC	GO:000577	late endos	24/931	296/19518	0.008134	0.171464	0.156604	MMD/STAI	24
GO:004319	CC	GO:004319	dendritic sl	6/931	38/19518	0.008654	0.17775	0.162344	MAP1A/CN	6
GO:000580	CC	GO:000580	cis-Golgi n	9/931	75/19518	0.009191	0.179879	0.164289	ATP2C1/IF	9
GO:003066	CC	GO:003066	COPI-coat	4/931	18/19518	0.009207	0.179879	0.164289	COPA/TME	4
GO:001989	CC	GO:001989	extrinsic cc	23/931	288/19518	0.011188	0.204074	0.186387	EMC2/BLO	23
GO:000592	CC	GO:000592	focal adhe	31/931	422/19518	0.011691	0.204074	0.186387	ITGBL1/M/	31
GO:003477	CC	GO:003477	secretory g	25/931	322/19518	0.011769	0.204074	0.186387	TGFB2/FRK	25
GO:000577	CC	GO:000577	peroxisom	8/931	65/19518	0.011828	0.204074	0.186387	FAR1/SLC2	8
GO:003190	CC	GO:003190	microbody	8/931	65/19518	0.011828	0.204074	0.186387	FAR1/SLC2	8
GO:007082	CC	GO:007082	tertiary gra	15/931	164/19518	0.011974	0.204074	0.186387	TNFAIP6/D	15
GO:004430	CC	GO:004430	dendritic b	2/931	4/19518	0.012787	0.209866	0.191677	MAP1A/M/	2
GO:003152	CC	GO:003152	microvillus	5/931	30/19518	0.012893	0.209866	0.191677	AMN/CTNI	5
GO:006020	CC	GO:006020	cytoplasmi	25/931	325/19518	0.0131	0.209866	0.191677	TGFB2/FRK	25
GO:003198	CC	GO:003198	vesicle lum	25/931	327/19518	0.014053	0.21675	0.197965	TGFB2/FRK	25
GO:003109	CC	GO:003109	platelet alp	8/931	67/19518	0.014071	0.21675	0.197965	TGFB2/VE(	8
GO:000830	CC	GO:000830	integrin co	5/931	31/19518	0.014784	0.223431	0.204067	ITGBL1/EN	5
GO:190481	CC	GO:190481	ficolin-1-ri	12/931	124/19518	0.01542	0.228723	0.2089	TNFAIP6/C	12
GO:003005	CC	GO:003005	cell-substr	31/931	432/19518	0.01584	0.230683	0.21069	ITGBL1/M/	31
GO:003196	CC	GO:003196	organelle c	19/931	236/19518	0.018389	0.26142	0.238763	ARMCX1/F	19
GO:000563	CC	GO:000563	nuclear env	34/931	490/19518	0.018805	0.26142	0.238763	CCND1/G/	34
GO:003013	CC	GO:003013	COPI-coat	4/931	22/19518	0.018929	0.26142	0.238763	COPA/TME	4
GO:001986	CC	GO:001986	outer mem	19/931	238/19518	0.019899	0.270158	0.246744	ARMCX1/F	19
GO:190273	CC	GO:190273	dendritic fi	2/931	5/19518	0.020641	0.275564	0.251681	FXR1/MAP	2
GO:003445	CC	GO:003445	centriolar s	11/931	115/19518	0.021468	0.276228	0.252288	NEK6/SDC	11
GO:004429	CC	GO:004429	dendrite te	3/931	13/19518	0.02161	0.276228	0.252288	IFT20/HSP	3
GO:000574	CC	GO:000574	mitochond	17/931	208/19518	0.021775	0.276228	0.252288	ARMCX1/F	17
GO:007168	CC	GO:007168	endocytic	4/931	23/19518	0.022071	0.276228	0.252288	HBB/HSP9	4
GO:000590	CC	GO:000590	clathrin-co	8/931	73/19518	0.022644	0.279048	0.254863	LRP12/AMI	8
GO:003001	CC	GO:003001	CCR4-NO	3/931	14/19518	0.026547	0.316097	0.288701	CPEB3/CN	3
GO:001660	CC	GO:001660	PML body	10/931	105/19518	0.028279	0.316097	0.288701	TRIM8/SUI	10
GO:003042	CC	GO:003042	growth cor	14/931	167/19518	0.029105	0.316097	0.288701	WDR47/C(	14
GO:003109	CC	GO:003109	platelet alp	9/931	91/19518	0.029426	0.316097	0.288701	TGFB2/VE(	9
GO:000083	CC	GO:000083	Hrd1p ubic	2/931	6/19518	0.029992	0.316097	0.288701	SYVN1/DE	2
GO:003108	CC	GO:003108	platelet de	2/931	6/19518	0.029992	0.316097	0.288701	LAMP2/CD	2
GO:007120	CC	GO:007120	histone pre	2/931	6/19518	0.029992	0.316097	0.288701	SLBP/ERI1	2
GO:007125	CC	GO:007125	translocon	2/931	6/19518	0.029992	0.316097	0.288701	SEC61G/SE	2
GO:009716	CC	GO:009716	nuclear stre	2/931	6/19518	0.029992	0.316097	0.288701	SUMO1/TI/	2
GO:009953	CC	GO:009953	synapse-a	2/931	6/19518	0.029992	0.316097	0.288701	LAMA5/TN	2
GO:199031	CC	GO:199031	Atg1/ULK1	2/931	6/19518	0.029992	0.316097	0.288701	ULK1/SMC	2
GO:004423	CC	GO:004423	organelle r	6/931	50/19518	0.030852	0.318145	0.290572	STARD3/C	6
GO:003065	CC	GO:003065	transport v	18/931	234/19518	0.031732	0.318145	0.290572	CNIH3/LM	18
GO:010100	CC	GO:010100	ficolin-1-ri	15/931	185/19518	0.031746	0.318145	0.290572	TNFAIP6/D	15
GO:009057	CC	GO:009057	RNA polym	3/931	15/19518	0.032033	0.318145	0.290572	CTNNB1/D	3
GO:003067	CC	GO:003067	phagocytic	8/931	78/19518	0.032172	0.318145	0.290572	RAB31/ATF	8
GO:000590	CC	GO:000590	microvillus	9/931	94/19518	0.035269	0.344519	0.31466	IFT20/AMN	9
GO:003042	CC	GO:003042	site of pola	14/931	172/19518	0.036095	0.348334	0.318145	WDR47/C(	14
GO:004533	CC	GO:004533	phagocytic	12/931	141/19518	0.037412	0.354508	0.323783	RAB31/SR(	12
GO:009885	CC	GO:009885	membrane	23/931	324/19518	0.037818	0.354508	0.323783	TFPI/LAMP	23
GO:000167	CC	GO:000167	female ger	3/931	16/19518	0.038062	0.354508	0.323783	MARCKS/D	3
GO:003196	CC	GO:003196	nuclear me	22/931	308/19518	0.039222	0.356725	0.325808	CCND1/G/	22
GO:007085	CC	GO:007085	cell body fi	2/931	7/19518	0.040678	0.356725	0.325808	HTR2A/SA(	2
GO:000577	CC	GO:000577	peroxisom	12/931	143/19518	0.040987	0.356725	0.325808	HSDL2/FAF	12
GO:004257	CC	GO:004257	microbody	12/931	143/19518	0.040987	0.356725	0.325808	HSDL2/FAF	12
GO:006202	CC	GO:006202	collagen-c	28/931	415/19518	0.041644	0.356725	0.325808	GREM1/EN	28
GO:009885	CC	GO:009885	actin-base	17/931	225/19518	0.04166	0.356725	0.325808	IFT20/AMN	17
GO:003283	CC	GO:003283	dendrite cy	4/931	28/19518	0.042351	0.356725	0.325808	TRAK2/UH	4
GO:003645	CC	GO:003645	ESCRT con	4/931	28/19518	0.042351	0.356725	0.325808	STAM2/ST/	4
GO:003013	CC	GO:003013	endocytic	24/931	347/19518	0.044381	0.356725	0.325808	RAB31/SR(	24
GO:003204	CC	GO:003204	guanyl-nu	3/931	17/19518	0.044624	0.356725	0.325808	RAP1A/ELN	3
GO:000594	CC	GO:000594	phosphatic	4/931	29/19518	0.04734	0.356725	0.325808	SOCS6/PIK	4
GO:000559	CC	GO:000559	collagen ty	1/931	1/19518	0.0477	0.356725	0.325808	COL12A1	1
GO:003299	CC	GO:003299	Bcl3-Bcl10	1/931	1/19518	0.0477	0.356725	0.325808	BCL3	1
GO:003475	CC	GO:003475	cytosolic a	1/931	1/19518	0.0477	0.356725	0.325808	AHR	1
GO:004354	CC	GO:004354	6-phosphc	1/931	1/19518	0.0477	0.356725	0.325808	PFKFB1	1
GO:005512	CC	GO:005512	striated mu	1/931	1/19518	0.0477	0.356725	0.325808	FRG1	1
GO:007126	CC	GO:007126	Ssh1 transl	1/931	1/19518	0.0477	0.356725	0.325808	SEC61G	1
GO:009896	CC	GO:009896	extracellula	1/931	1/19518	0.0477	0.356725	0.325808	LAMA5	1
GO:015001	CC	GO:015001	apical dista	1/931	1/19518	0.0477	0.356725	0.325808	MAP2	1
GO:199072	CC	GO:199072	DAPK1-cal	1/931	1/19518	0.0477	0.356725	0.325808	DAPK1	1
GO:000576	CC	GO:000576	early endo	28/931	423/19518	0.050739	0.356725	0.325808	ITCH/RAB3	28
GO:000566	CC	GO:000566	RNA polym	3/931	18/19518	0.051708	0.356725	0.325808	POLR2K/P(	3
GO:000079	CC	GO:000079	condensin	2/931	8/19518	0.052551	0.356725	0.325808	SMC4/SM(	2
GO:000083	CC	GO:000083	Hrd1p ubic	2/931	8/19518	0.052551	0.356725	0.325808	SYVN1/DE	2
GO:000596	CC	GO:000596	mitochond	2/931	8/19518	0.052551	0.356725	0.325808	PDK2/DLA	2
GO:003123	CC	GO:003123	extrinsic cc	2/931	8/19518	0.052551	0.356725	0.325808	TF/ANXA1	2
GO:003645	CC	GO:003645	growth fac	2/931	8/19518	0.052551	0.356725	0.325808	IGFBP3/PD	2
GO:004238	CC	GO:004238	paraspeckl	2/931	8/19518	0.052551	0.356725	0.325808	CPSF6/PSP	2
GO:004282	CC	GO:004282	MHC class	2/931	8/19518	0.052551	0.356725	0.325808	TAP1/CALF	2
GO:004525	CC	GO:004525	pyruvate d	2/931	8/19518	0.052551	0.356725	0.325808	PDK2/DLA	2
GO:006120	CC	GO:006120	clathrin-sc	2/931	8/19518	0.052551	0.356725	0.325808	RAB3A/DN	2
GO:006120	CC	GO:006120	clathrin-sc	2/931	8/19518	0.052551	0.356725	0.325808	RAB3A/DN	2
GO:003013	CC	GO:003013	transport v	28/931	425/19518	0.053223	0.356788	0.325866	BLOC1S6/(	28
GO:009863	CC	GO:009863	protein cor	6/931	57/19518	0.053451	0.356788	0.325866	ITGBL1/EN	6
GO:004512	CC	GO:004512	membrane	22/931	323/19518	0.059971	0.397	0.362593	TFPI/BAAL	22
GO:000083	CC	GO:000083	ER ubiquiti	2/931	9/19518	0.065475	0.422945	0.38629	SYVN1/DE	2
GO:003301	CC	GO:003301	sarcoplasm	2/931	9/19518	0.065475	0.422945	0.38629	HRC/CALR	2
GO:004429	CC	GO:004429	dendritic g	2/931	9/19518	0.065475	0.422945	0.38629	HSP90AA1	2
GO:003017	CC	GO:003017	filopodium	9/931	108/19518	0.072927	0.430131	0.392853	MYO1B/FX	9
GO:003558	CC	GO:003558	specific gra	6/931	62/19518	0.074456	0.430131	0.392853	FRK/CHI3L	6
GO:000166	CC	GO:000166	acrosomal	11/931	141/19518	0.074461	0.430131	0.392853	FNDC3A/S	11
GO:004423	CC	GO:004423	mitochond	3/931	21/19518	0.075925	0.430131	0.392853	CANX/CLC	3
GO:001705	CC	GO:001705	transcriptic	7/931	78/19518	0.078242	0.430131	0.392853	CCND1/R(	7
GO:000577	CC	GO:000577	vacuolar lu	13/931	176/19518	0.078703	0.430131	0.392853	FRK/LAMP	13
GO:000022	CC	GO:000022	vacuolar pı	2/931	10/19518	0.079321	0.430131	0.392853	ATP6V0A2	2
GO:003131	CC	GO:003131	extrinsic cc	2/931	10/19518	0.079321	0.430131	0.392853	WDR81/AN	2
GO:001658	CC	GO:001658	Sin3 comp	3/931	22/19518	0.084925	0.430131	0.392853	SAP30/BRN	3
GO:007082	CC	GO:007082	Sin3-type	3/931	22/19518	0.084925	0.430131	0.392853	SAP30/BRN	3
GO:000030	CC	GO:000030	cyclin-dep	5/931	50/19518	0.088787	0.430131	0.392853	CCND1/CD	5
GO:001470	CC	GO:001470	intercalate	5/931	50/19518	0.088787	0.430131	0.392853	PIK3CA/DL	5
GO:000165	CC	GO:000165	fibrillar cen	11/931	146/19518	0.09001	0.430131	0.392853	ZNRF2/DD	11
GO:001503	CC	GO:001503	Cajal body	7/931	81/19518	0.091434	0.430131	0.392853	TRIM22/DI	7
GO:000030	CC	GO:000030	cytoplasmi	1/931	2/19518	0.093126	0.430131	0.392853	CDK14	1
GO:000032	CC	GO:000032	storage va	1/931	2/19518	0.093126	0.430131	0.392853	CDC42	1
GO:000294	CC	GO:000294	cyclin K-CI	1/931	2/19518	0.093126	0.430131	0.392853	CDK12	1
GO:000558	CC	GO:000558	collagen ty	1/931	2/19518	0.093126	0.430131	0.392853	COL1A1	1
GO:000804	CC	GO:000804	intracellula	1/931	2/19518	0.093126	0.430131	0.392853	FTL	1
GO:001622	CC	GO:001622	procollage	1/931	2/19518	0.093126	0.430131	0.392853	P4HA1	1
GO:001693	CC	GO:001693	glycine-ga	1/931	2/19518	0.093126	0.430131	0.392853	GLRB	1
GO:001705	CC	GO:001705	negative c	1/931	2/19518	0.093126	0.430131	0.392853	DR1	1
GO:003120	CC	GO:003120	endoplasm	1/931	2/19518	0.093126	0.430131	0.392853	SEC61B	1
GO:003137	CC	GO:003137	UBC13-MI	1/931	2/19518	0.093126	0.430131	0.392853	UBE2V2	1
GO:003231	CC	GO:003231	angiogenir	1/931	2/19518	0.093126	0.430131	0.392853	ANG	1
GO:003325	CC	GO:003325	Bcl3/NF-ka	1/931	2/19518	0.093126	0.430131	0.392853	BCL3	1
GO:003468	CC	GO:003468	integrin alp	1/931	2/19518	0.093126	0.430131	0.392853	ITGA11	1
GO:003468	CC	GO:003468	integrin alp	1/931	2/19518	0.093126	0.430131	0.392853	ITGAV	1
GO:003468	CC	GO:003468	integrin alp	1/931	2/19518	0.093126	0.430131	0.392853	ITGAV	1
GO:003475	CC	GO:003475	Scrib-APC	1/931	2/19518	0.093126	0.430131	0.392853	CTNNB1	1
GO:003475	CC	GO:003475	nuclear ary	1/931	2/19518	0.093126	0.430131	0.392853	AHR	1
GO:003618	CC	GO:003618	early phag	1/931	2/19518	0.093126	0.430131	0.392853	RAB31	1
GO:004282	CC	GO:004282	TAP compl	1/931	2/19518	0.093126	0.430131	0.392853	TAP1	1
GO:006207	CC	GO:006207	histone mF	1/931	2/19518	0.093126	0.430131	0.392853	SLBP	1
GO:007028	CC	GO:007028	ferritin con	1/931	2/19518	0.093126	0.430131	0.392853	FTL	1
GO:007055	CC	GO:007055	synaptobre	1/931	2/19518	0.093126	0.430131	0.392853	CPLX2	1
GO:007100	CC	GO:007100	U2-type p	1/931	2/19518	0.093126	0.430131	0.392853	GCFC2	1
GO:009705	CC	GO:009705	CRLF-CLCI	1/931	2/19518	0.093126	0.430131	0.392853	CRLF1	1
GO:009712	CC	GO:009712	cyclin D1-(	1/931	2/19518	0.093126	0.430131	0.392853	CCND1	1
GO:009763	CC	GO:009763	extrinsic cc	1/931	2/19518	0.093126	0.430131	0.392853	ULK1	1
GO:009911	CC	GO:009911	chromosor	1/931	2/19518	0.093126	0.430131	0.392853	SIRT6	1
GO:012004	CC	GO:012004	stereociliur	1/931	2/19518	0.093126	0.430131	0.392853	FCHSD2	1
GO:015000	CC	GO:015000	distal denc	1/931	2/19518	0.093126	0.430131	0.392853	MAP2	1
GO:199026	CC	GO:199026	platelet-de	1/931	2/19518	0.093126	0.430131	0.392853	PDGFA	1
GO:199056	CC	GO:199056	HSP90-CD	1/931	2/19518	0.093126	0.430131	0.392853	CDC37	1
GO:199068	CC	GO:199068	CSF1-CSF1	1/931	2/19518	0.093126	0.430131	0.392853	CSF1	1
GO:199071	CC	GO:199071	beta-cater	1/931	2/19518	0.093126	0.430131	0.392853	CTNNB1	1
GO:199097	CC	GO:199097	EMILIN cor	1/931	2/19518	0.093126	0.430131	0.392853	EMILIN1	1
GO:003183	CC	GO:003183	haptoglobi	2/931	11/19518	0.093974	0.430131	0.392853	HBB/HBD	2
GO:006009	CC	GO:006009	kinocilium	2/931	11/19518	0.093974	0.430131	0.392853	IFT20/DCD	2
GO:009773	CC	GO:009773	9+2 non-n	2/931	11/19518	0.093974	0.430131	0.392853	IFT20/DCD	2
GO:003131	CC	GO:003131	extrinsic cc	5/931	51/19518	0.094785	0.431378	0.393991	EMC2/ULK	5
GO:000579	CC	GO:000579	rough endo	7/931	82/19518	0.096092	0.433881	0.396277	PTGDS/RPI	7
GO:006169	CC	GO:006169	transferase	20/931	306/19518	0.096418	0.433881	0.396277	CCND1/SC	20
GO:003086	CC	GO:003086	rough endo	3/931	24/19518	0.104187	0.46579	0.42542	SEC61G/SI	3
GO:000558	CC	GO:000558	fibrillar coll	2/931	12/19518	0.109324	0.46579	0.42542	LUM/COL1	2
GO:000583	CC	GO:000583	hemoglobi	2/931	12/19518	0.109324	0.46579	0.42542	HBB/HBD	2
GO:003090	CC	GO:003090	retromer c	2/931	12/19518	0.109324	0.46579	0.42542	SNX12/TB(	2
GO:003317	CC	GO:003317	proton-tra	2/931	12/19518	0.109324	0.46579	0.42542	ATP6V0A2	2
GO:004475	CC	GO:004475	autolysoso	2/931	12/19518	0.109324	0.46579	0.42542	LAMP2/FTI	2
GO:006019	CC	GO:006019	clathrin-sc	2/931	12/19518	0.109324	0.46579	0.42542	RAB3A/DN	2
GO:009750	CC	GO:009750	Gemini of	2/931	12/19518	0.109324	0.46579	0.42542	ZPR1/NPA	2
GO:009864	CC	GO:009864	banded co	2/931	12/19518	0.109324	0.46579	0.42542	LUM/COL1	2
GO:190493	CC	GO:190493	amphisom	2/931	12/19518	0.109324	0.46579	0.42542	CHMP5/CH	2
GO:003198	CC	GO:003198	Golgi ciste	9/931	118/19518	0.110983	0.470354	0.42959	ZDHHC14/	9
GO:003190	CC	GO:003190	early endo	13/931	187/19518	0.112064	0.47244	0.431494	ITCH/RAB3	13
GO:001645	CC	GO:001645	myosin cor	5/931	54/19518	0.113968	0.477949	0.436526	BMF/MYO1	5
GO:190255	CC	GO:190255	serine/thre	9/931	121/19518	0.124211	0.479153	0.437626	CCND1/CD	9
GO:009897	CC	GO:009897	glutamater	25/931	410/19518	0.125257	0.479153	0.437626	CADPS2/SI	25
GO:000015	CC	GO:000015	cytoplasmi	2/931	13/19518	0.125272	0.479153	0.437626	SYVN1/DE	2
GO:000567	CC	GO:000567	chromatin	2/931	13/19518	0.125272	0.479153	0.437626	CBX3/RB1	2
GO:000573	CC	GO:000573	RNA polym	2/931	13/19518	0.125272	0.479153	0.437626	POLR2K/P(	2
GO:004419	CC	GO:004419	cytolytic gı	2/931	13/19518	0.125272	0.479153	0.437626	SRGN/CAL	2
GO:012011	CC	GO:012011	neuron prc	7/931	89/19518	0.132228	0.479153	0.437626	BLOC1S6/	7
GO:000853	CC	GO:000853	proteasom	1/931	3/19518	0.136388	0.479153	0.437626	PSME1	1
GO:000933	CC	GO:000933	glycerol-3	1/931	3/19518	0.136388	0.479153	0.437626	GPD2	1
GO:002001	CC	GO:002001	ciliary pock	1/931	3/19518	0.136388	0.479153	0.437626	SNAP29	1
GO:002001	CC	GO:002001	ciliary pock	1/931	3/19518	0.136388	0.479153	0.437626	SNAP29	1
GO:003093	CC	GO:003093	anchoring	1/931	3/19518	0.136388	0.479153	0.437626	COL12A1	1
GO:003318	CC	GO:003318	CAF-1 con	1/931	3/19518	0.136388	0.479153	0.437626	RBBP4	1
GO:003466	CC	GO:003466	integrin alp	1/931	3/19518	0.136388	0.479153	0.437626	EMILIN1	1
GO:003468	CC	GO:003468	integrin alp	1/931	3/19518	0.136388	0.479153	0.437626	ITGAV	1
GO:003500	CC	GO:003500	subapical c	1/931	3/19518	0.136388	0.479153	0.437626	CRB3	1
GO:003506	CC	GO:003506	interchrom	1/931	3/19518	0.136388	0.479153	0.437626	CPSF6	1
GO:003586	CC	GO:003586	alphav-bet	1/931	3/19518	0.136388	0.479153	0.437626	ITGAV	1
GO:003586	CC	GO:003586	alphav-bet	1/931	3/19518	0.136388	0.479153	0.437626	ITGAV	1
GO:004262	CC	GO:004262	mast cell g	1/931	3/19518	0.136388	0.479153	0.437626	SRGN	1
GO:004325	CC	GO:004325	laminin-10	1/931	3/19518	0.136388	0.479153	0.437626	LAMA5	1
GO:004326	CC	GO:004326	laminin-11	1/931	3/19518	0.136388	0.479153	0.437626	LAMA5	1
GO:004669	CC	GO:004669	SLIK (SAG	1/931	3/19518	0.136388	0.479153	0.437626	TAF6	1
GO:004847	CC	GO:004847	Holliday ju	1/931	3/19518	0.136388	0.479153	0.437626	EME1	1
GO:006020	CC	GO:006020	clathrin-sc	1/931	3/19518	0.136388	0.479153	0.437626	RAB3A	1
GO:006020	CC	GO:006020	clathrin-sc	1/931	3/19518	0.136388	0.479153	0.437626	RAB3A	1
GO:007003	CC	GO:007003	synaptobre	1/931	3/19518	0.136388	0.479153	0.437626	CPLX2	1
GO:007036	CC	GO:007036	beta-cater	1/931	3/19518	0.136388	0.479153	0.437626	CTNNB1	1
GO:007181	CC	GO:007181	BAT3 comp	1/931	3/19518	0.136388	0.479153	0.437626	UBL4A	1
GO:007256	CC	GO:007256	endothelia	1/931	3/19518	0.136388	0.479153	0.437626	ENG	1
GO:009712	CC	GO:009712	cyclin D2-(	1/931	3/19518	0.136388	0.479153	0.437626	CCND2	1
GO:009714	CC	GO:009714	centralspin	1/931	3/19518	0.136388	0.479153	0.437626	RACGAP1	1
GO:009745	CC	GO:009745	glial limitin	1/931	3/19518	0.136388	0.479153	0.437626	ADGRG1	1
GO:009762	CC	GO:009762	extrinsic cc	1/931	3/19518	0.136388	0.479153	0.437626	ULK1	1
GO:009905	CC	GO:009905	activating	1/931	3/19518	0.136388	0.479153	0.437626	ASCC3	1
GO:190263	CC	GO:190263	kinociliary	1/931	3/19518	0.136388	0.479153	0.437626	IFT20	1
GO:199011	CC	GO:199011	RQC comp	1/931	3/19518	0.136388	0.479153	0.437626	LTN1	1
GO:010103	CC	GO:010103	chaperone	4/931	42/19518	0.138792	0.485468	0.443394	BAG2/DN/	4
GO:190291	CC	GO:190291	protein kin	10/931	142/19518	0.141303	0.487222	0.444995	CCND1/CD	10
GO:004475	CC	GO:004475	amphisom	2/931	14/19518	0.141726	0.487222	0.444995	CHMP5/CH	2
GO:009752	CC	GO:009752	sperm plas	2/931	14/19518	0.141726	0.487222	0.444995	HSP90AA1	2
GO:014067	CC	GO:014067	ATAC com	2/931	14/19518	0.141726	0.487222	0.444995	MBIP/DR1	2
GO:003001	CC	GO:003001	myofibril	15/931	233/19518	0.147727	0.499117	0.455859	FLNC/LMA	15
GO:000592	CC	GO:000592	bicellular ti	9/931	126/19518	0.148035	0.499117	0.455859	CCND1/CY	9
GO:003577	CC	GO:003577	ribonucleo	17/931	271/19518	0.152383	0.499117	0.455859	ZFP36L1/R	17
GO:003646	CC	GO:003646	cytoplasmi	16/931	253/19518	0.153798	0.499117	0.455859	ZFP36L1/R	16
GO:003258	CC	GO:003258	Golgi ciste	7/931	93/19518	0.155505	0.499117	0.455859	ZDHHC14/	7
GO:199020	CC	GO:199020	oxidoreduc	9/931	128/19518	0.158161	0.499117	0.455859	GPD2/NDU	9
GO:003012	CC	GO:003012	COPII vesic	2/931	15/19518	0.158602	0.499117	0.455859	SEC23A/S/	2
GO:009744	CC	GO:009744	apical denc	2/931	15/19518	0.158602	0.499117	0.455859	CPEB3/MA	2
GO:003258	CC	GO:003258	multivesicu	3/931	29/19518	0.158635	0.499117	0.455859	CHMP5/CH	3
GO:009855	CC	GO:009855	lumenal sic	3/931	29/19518	0.158635	0.499117	0.455859	HLA-DQA	3
GO:009868	CC	GO:009868	Schaffer cc	7/931	94/19518	0.161602	0.499117	0.455859	RAP2A/FB)	7
GO:001989	CC	GO:001989	extrinsic cc	10/931	147/19518	0.164919	0.499117	0.455859	SNX18/FRI	10
GO:000210	CC	GO:000210	podosome	3/931	30/19518	0.170412	0.499117	0.455859	DBNL/CTT	3
GO:000572	CC	GO:000572	pericentric	3/931	30/19518	0.170412	0.499117	0.455859	CBX3/SIRT	3
GO:007100	CC	GO:007100	U2-type ca	3/931	30/19518	0.170412	0.499117	0.455859	BCAS2/CW	3
GO:015003	CC	GO:015003	distal axon	17/931	277/19518	0.173457	0.499117	0.455859	WDR47/NT	17
GO:007016	CC	GO:007016	tight juncti	9/931	131/19518	0.173958	0.499117	0.455859	CCND1/CY	9
GO:000011	CC	GO:000011	histone de	6/931	79/19518	0.17492	0.499117	0.455859	RCOR1/SA	6
GO:000578	CC	GO:000578	signal reco	2/931	16/19518	0.175822	0.499117	0.455859	SRP9/SRP7	2
GO:003013	CC	GO:003013	clathrin co	2/931	16/19518	0.175822	0.499117	0.455859	AP2S1/EPS	2
GO:004850	CC	GO:004850	signal reco	2/931	16/19518	0.175822	0.499117	0.455859	SRP9/SRP7	2
GO:000208	CC	GO:000208	outer acros	1/931	4/19518	0.177588	0.499117	0.455859	TRIP11	1
GO:000294	CC	GO:000294	tumor necı	1/931	4/19518	0.177588	0.499117	0.455859	TRADD	1
GO:000572	CC	GO:000572	perichroma	1/931	4/19518	0.177588	0.499117	0.455859	CPSF6	1
GO:000574	CC	GO:000574	mitochond	1/931	4/19518	0.177588	0.499117	0.455859	SDHD	1
GO:000578	CC	GO:000578	signal reco	1/931	4/19518	0.177588	0.499117	0.455859	SRP9	1
GO:000586	CC	GO:000586	muscle thir	1/931	4/19518	0.177588	0.499117	0.455859	TPM1	1
GO:000594	CC	GO:000594	phosphatic	1/931	4/19518	0.177588	0.499117	0.455859	PIK3CA	1
GO:003325	CC	GO:003325	I-kappaB/I	1/931	4/19518	0.177588	0.499117	0.455859	BCL3	1
GO:003326	CC	GO:003326	internode r	1/931	4/19518	0.177588	0.499117	0.455859	MBP	1
GO:003359	CC	GO:003359	TSC1-TSC	1/931	4/19518	0.177588	0.499117	0.455859	TBC1D7	1
GO:003518	CC	GO:003518	Rb-E2F co	1/931	4/19518	0.177588	0.499117	0.455859	RB1	1
GO:003586	CC	GO:003586	alphav-bet	1/931	4/19518	0.177588	0.499117	0.455859	ITGAV	1
GO:004256	CC	GO:004256	insulin-like	1/931	4/19518	0.177588	0.499117	0.455859	IGFBP3	1
GO:004258	CC	GO:004258	germinal v	1/931	4/19518	0.177588	0.499117	0.455859	MARCKS	1
GO:004321	CC	GO:004321	lateral loop	1/931	4/19518	0.177588	0.499117	0.455859	DLG1	1
GO:004525	CC	GO:004525	succinate c	1/931	4/19518	0.177588	0.499117	0.455859	SDHD	1
GO:004527	CC	GO:004527	respiratory	1/931	4/19518	0.177588	0.499117	0.455859	SDHD	1
GO:004528	CC	GO:004528	succinate c	1/931	4/19518	0.177588	0.499117	0.455859	SDHD	1
GO:004528	CC	GO:004528	fumarate r	1/931	4/19518	0.177588	0.499117	0.455859	SDHD	1
GO:006019	CC	GO:006019	clathrin-sc	1/931	4/19518	0.177588	0.499117	0.455859	RAB3A	1
GO:006020	CC	GO:006020	clathrin-sc	1/931	4/19518	0.177588	0.499117	0.455859	RAB3A	1
GO:007003	CC	GO:007003	synaptobre	1/931	4/19518	0.177588	0.499117	0.455859	SNAP23	1
GO:007166	CC	GO:007166	catenin-TC	1/931	4/19518	0.177588	0.499117	0.455859	CTNNB1	1
GO:007253	CC	GO:007253	perineuron	1/931	4/19518	0.177588	0.499117	0.455859	TNR	1

GO:009073	CC	GO:009073	tenascin cc	1/931	4/19518	0.177588	0.499117	0.455859	TNR	1
GO:009866	CC	GO:009866	G protein-	1/931	4/19518	0.177588	0.499117	0.455859	HTR2A	1
GO:009912	CC	GO:009912	mitochond	1/931	4/19518	0.177588	0.499117	0.455859	ISCU	1
GO:014028	CC	GO:014028	endoplasm	1/931	4/19518	0.177588	0.499117	0.455859	STARD3	1
GO:190334	CC	GO:190334	omegasom	1/931	4/19518	0.177588	0.499117	0.455859	ULK1	1
GO:190534	CC	GO:190534	endodeoxy	1/931	4/19518	0.177588	0.499117	0.455859	EME1	1
GO:199076	CC	GO:199076	proximal n	1/931	4/19518	0.177588	0.499117	0.455859	MAP2	1
GO:005108	MF	GO:005108	unfolded p	19/932	122/18369	1.21E-05	0.007885	0.007609	RP2/HSPB6	19
GO:001620	MF	GO:001620	antioxidant	15/932	82/18369	1.39E-05	0.007885	0.007609	CYGB/GPX	15
GO:004438	MF	GO:004438	ubiquitin-li	35/932	320/18369	1.64E-05	0.007885	0.007609	TRIB3/BAG	35
GO:001668	MF	GO:001668	oxidoreduc	12/932	57/18369	2.31E-05	0.008337	0.008045	SESN3/CY	12
GO:000474	MF	GO:000474	pyruvate d	4/932	5/18369	3.16E-05	0.009114	0.008795	PDK2/BCK	4
GO:000460	MF	GO:000460	glutathione	7/932	22/18369	7.37E-05	0.017089	0.016491	GPX4/PTG	7
GO:000460	MF	GO:000460	peroxidase	11/932	55/18369	8.30E-05	0.017089	0.016491	CYGB/GPX	11
GO:003162	MF	GO:003162	ubiquitin p	31/932	301/18369	0.00015	0.027027	0.026082	TRIB3/BAG	31
GO:000371	MF	GO:000371	transcriptic	21/932	190/18369	0.000671	0.107589	0.103827	TRIB3/CCN	21
GO:005108	MF	GO:005108	chaperone	14/932	104/18369	0.000766	0.110399	0.106539	BAG2/HSP	14
GO:000551	MF	GO:000551	collagen bi	10/932	66/18369	0.001714	0.211418	0.204025	ITGA11/PP	10
GO:006059	MF	GO:006059	ATPase reg	8/932	46/18369	0.001995	0.211418	0.204025	BAG2/BAG	8
GO:000070	MF	GO:000070	oxidized py	2/932	2/18369	0.002572	0.211418	0.204025	NTHL1/SM	2
GO:000435	MF	GO:000435	glutamate-	2/932	2/18369	0.002572	0.211418	0.204025	GCLM/GCL	2
GO:003255	MF	GO:003255	adenyl dec	2/932	2/18369	0.002572	0.211418	0.204025	HSP90AA1	2
GO:004775	MF	GO:004775	chondroitir	2/932	2/18369	0.002572	0.211418	0.204025	DSE/DSEL	2
GO:007120	MF	GO:007120	histone pre	2/932	2/18369	0.002572	0.211418	0.204025	SLBP/ERI1	2
GO:003281	MF	GO:003281	tumor necr	8/932	48/18369	0.002639	0.211418	0.204025	TNFSF9/BI	8
GO:005178	MF	GO:005178	misfolded	6/932	29/18369	0.00292	0.214092	0.206606	DNAJB11/	6
GO:000014	MF	GO:000014	SNARE bin	13/932	107/18369	0.002969	0.214092	0.206606	BLOC1S6/	13
GO:000504	MF	GO:000504	signal reco	3/932	7/18369	0.003907	0.268261	0.258881	SRP9/SRP7	3
GO:005066	MF	GO:005066	NADP bind	8/932	53/18369	0.004962	0.320045	0.308855	ME1/G6PD	8
GO:001685	MF	GO:001685	isomerase	16/932	154/18369	0.005154	0.320045	0.308855	PTGDS/DS	16
GO:003014	MF	GO:003014	manganese	9/932	65/18369	0.005327	0.320045	0.308855	DYRK2/ME	9
GO:000466	MF	GO:000466	prostaglan	2/932	3/18369	0.007455	0.335924	0.324178	PTGDS/PT	2
GO:000504	MF	GO:000504	KDEL sequ	2/932	3/18369	0.007455	0.335924	0.324178	KDELR3/KD	2
GO:000545	MF	GO:000545	UDP-galac	2/932	3/18369	0.007455	0.335924	0.324178	SLC35A3/S	2
GO:001511	MF	GO:001511	iodide tran	2/932	3/18369	0.007455	0.335924	0.324178	SLC5A5/SL	2
GO:003203	MF	GO:003203	myosin II h	2/932	3/18369	0.007455	0.335924	0.324178	MYL4/MYL	2
GO:003255	MF	GO:003255	purine deo	2/932	3/18369	0.007455	0.335924	0.324178	HSP90AA1	2
GO:003281	MF	GO:003281	sterol resp	2/932	3/18369	0.007455	0.335924	0.324178	NR1H3/SM	2
GO:014067	MF	GO:014067	ATP-deper	2/932	3/18369	0.007455	0.335924	0.324178	ZNHIT1/M	2
GO:190539	MF	GO:190539	retromer c	3/932	9/18369	0.008686	0.343203	0.331202	TBC1D5/S	3
GO:000532	MF	GO:000532	long-chain	4/932	17/18369	0.009221	0.343203	0.331202	MFSD2A/S	4
GO:001686	MF	GO:001686	intramolec	7/932	48/18369	0.010115	0.343203	0.331202	PTGDS/PD	7
GO:000202	MF	GO:000202	protease b	14/932	138/18369	0.010443	0.343203	0.331202	SYVN1/TH	14
GO:000375	MF	GO:000375	protein dis	4/932	18/18369	0.011388	0.343203	0.331202	PDIA6/ERP	4
GO:001686	MF	GO:001686	intramolec	4/932	18/18369	0.011388	0.343203	0.331202	PDIA6/ERP	4
GO:003054	MF	GO:003054	Hsp70 prot	7/932	50/18369	0.012578	0.343203	0.331202	FICD/DNA	7
GO:001503	MF	GO:001503	disulfide o	6/932	39/18369	0.013056	0.343203	0.331202	PDIA6/DN	6
GO:000070	MF	GO:000070	oxidized ba	2/932	4/18369	0.014408	0.343203	0.331202	NTHL1/SM	2
GO:000369	MF	GO:000369	satellite DI	2/932	4/18369	0.014408	0.343203	0.331202	MBD2/MB	2
GO:000478	MF	GO:000478	superoxide	2/932	4/18369	0.014408	0.343203	0.331202	NQO1/SO	2
GO:000517	MF	GO:000517	stem cell fa	2/932	4/18369	0.014408	0.343203	0.331202	SH2B3/KIT	2
GO:001672	MF	GO:001672	oxidoreduc	2/932	4/18369	0.014408	0.343203	0.331202	NQO1/SO	2
GO:003566	MF	GO:003566	Toll-like re	2/932	4/18369	0.014408	0.343203	0.331202	LY96/TIRA	2
GO:000486	MF	GO:000486	protein kin	6/932	40/18369	0.014719	0.343203	0.331202	TRIB3/MBI	6
GO:000512	MF	GO:000512	death rece	4/932	20/18369	0.01664	0.343203	0.331202	BID/MADD	4
GO:001983	MF	GO:001983	growth fac	13/932	132/18369	0.0167	0.343203	0.331202	IGFBP3/LIF	13
GO:001675	MF	GO:001675	glycosyltra	22/932	266/18369	0.016923	0.343203	0.331202	DPAGT1/R	22
GO:000512	MF	GO:000512	cytokine re	22/932	270/18369	0.01973	0.343203	0.331202	GREM1/IT	22
GO:199038	MF	GO:199038	ubiquitin-s	4/932	21/18369	0.019748	0.343203	0.331202	SYVN1/SU	4
GO:199078	MF	GO:199078	protein tyr	12/932	121/18369	0.019953	0.343203	0.331202	SH2B3/ELN	12
GO:000077	MF	GO:000077	adenyl-nu	3/932	12/18369	0.020302	0.343203	0.331202	BAG2/BAG	3
GO:000849	MF	GO:000849	UDP-galac	3/932	12/18369	0.020302	0.343203	0.331202	B3GNT2/B	3
GO:005505	MF	GO:005505	D-glucose	3/932	12/18369	0.020302	0.343203	0.331202	SLC2A12/S	3
GO:000122	MF	GO:000122	transcriptic	6/932	43/18369	0.020577	0.343203	0.331202	GATA6/AH	6
GO:004313	MF	GO:004313	ubiquitin b	10/932	94/18369	0.020724	0.343203	0.331202	UBE2L6/C	10
GO:001990	MF	GO:001990	syntaxin bi	8/932	68/18369	0.021346	0.343203	0.331202	BLOC1S6/	8
GO:001921	MF	GO:001921	kinase inhil	6/932	44/18369	0.022836	0.343203	0.331202	TRIB3/MBI	6
GO:000204	MF	GO:000204	opsin bindi	2/932	5/18369	0.02321	0.343203	0.331202	IFT20/PRC	2
GO:000457	MF	GO:000457	dolichyl-di	2/932	5/18369	0.02321	0.343203	0.331202	RPN1/OST	2
GO:000465	MF	GO:000465	procollage	2/932	5/18369	0.02321	0.343203	0.331202	P4HA1/P4	2
GO:000503	MF	GO:000503	neurotroph	2/932	5/18369	0.02321	0.343203	0.331202	NTRK2/SO	2
GO:000853	MF	GO:000853	N-acetylla	2/932	5/18369	0.02321	0.343203	0.331202	B3GNT2/B	2
GO:003172	MF	GO:003172	hemoglobi	2/932	5/18369	0.02321	0.343203	0.331202	HBB/HBD	2
GO:003255	MF	GO:003255	deoxyribor	2/932	5/18369	0.02321	0.343203	0.331202	HSP90AA1	2
GO:004692	MF	GO:004692	ER retentio	2/932	5/18369	0.02321	0.343203	0.331202	KDELR3/KD	2
GO:005102	MF	GO:005102	GDP-disso	2/932	5/18369	0.02321	0.343203	0.331202	HSP90AA1	2
GO:199093	MF	GO:199093	splicing fac	2/932	5/18369	0.02321	0.343203	0.331202	RBM20/UH	2
GO:000837	MF	GO:000837	acetylgluc	6/932	45/18369	0.025256	0.343203	0.331202	MGAT2/B3	6
GO:000122	MF	GO:000122	transcriptic	11/932	111/18369	0.025318	0.343203	0.331202	GATA6/AH	11
GO:001910	MF	GO:001910	DNA N-gly	3/932	13/18369	0.025416	0.343203	0.331202	NTHL1/MB	3
GO:003138	MF	GO:003138	protein tag	3/932	13/18369	0.025416	0.343203	0.331202	SUMO1/IS	3
GO:003199	MF	GO:003199	insulin-like	3/932	13/18369	0.025416	0.343203	0.331202	IGFBP3/IG	3
GO:003532	MF	GO:003532	Toll-like re	3/932	13/18369	0.025416	0.343203	0.331202	LY96/TIRA	3
GO:001503	MF	GO:001503	protein-dis	5/932	34/18369	0.027262	0.343203	0.331202	PDIA6/DN	5
GO:003033	MF	GO:003033	cyclin bind	5/932	34/18369	0.027262	0.343203	0.331202	CDK12/CD	5
GO:000487	MF	GO:000487	nuclear rec	6/932	46/18369	0.027841	0.343203	0.331202	AHR/NR1H	6
GO:009853	MF	GO:009853	ligand-acti	6/932	46/18369	0.027841	0.343203	0.331202	AHR/NR1H	6
GO:003107	MF	GO:003107	heat shock	12/932	128/18369	0.029447	0.343203	0.331202	BAG2/AHR	12
GO:003218	MF	GO:003218	ubiquitin-li	11/932	114/18369	0.030079	0.343203	0.331202	UBE2L6/C	11
GO:000462	MF	GO:000462	lysophosp	4/932	24/18369	0.03111	0.343203	0.331202	ABHD4/PL	4
GO:000517	MF	GO:000517	vascular er	3/932	14/18369	0.031153	0.343203	0.331202	GREM1/VE	3
GO:000534	MF	GO:000534	oxygen car	3/932	14/18369	0.031153	0.343203	0.331202	CYGB/HBB	3
GO:001630	MF	GO:001630	phosphatic	3/932	14/18369	0.031153	0.343203	0.331202	PIK3C2A/P	3
GO:006157	MF	GO:006157	Lys63-spe	3/932	14/18369	0.031153	0.343203	0.331202	TNFAIP3/Y	3
GO:000808	MF	GO:000808	growth fac	14/932	160/18369	0.033204	0.343203	0.331202	GDF15/TG	14
GO:000166	MF	GO:000166	alpha-N-a	2/932	6/18369	0.033655	0.343203	0.331202	ST6GALNA	2
GO:000457	MF	GO:000457	oligosacch	2/932	6/18369	0.033655	0.343203	0.331202	RPN1/OST	2
GO:000512	MF	GO:000512	ciliary neur	2/932	6/18369	0.033655	0.343203	0.331202	LIFR/CRLF1	2
GO:001509	MF	GO:001509	ferrous iror	2/932	6/18369	0.033655	0.343203	0.331202	SLC25A37/	2
GO:003091	MF	GO:003091	TPR domai	2/932	6/18369	0.033655	0.343203	0.331202	HSP90AA1	2
GO:004432	MF	GO:004432	retinoic aci	2/932	6/18369	0.033655	0.343203	0.331202	NR2F1/RA	2
GO:007008	MF	GO:007008	chromo sh	2/932	6/18369	0.033655	0.343203	0.331202	LBR/SP100	2
GO:010627	MF	GO:010627	NAD+-prc	2/932	6/18369	0.033655	0.343203	0.331202	ARF4/SIRT	2
GO:190148	MF	GO:190148	oleate tran	2/932	6/18369	0.033655	0.343203	0.331202	MFSD2A/S	2
GO:199043	MF	GO:199043	extracellula	2/932	6/18369	0.033655	0.343203	0.331202	ITGB8/ITG	2
GO:014010	MF	GO:014010	molecular	9/932	88/18369	0.034213	0.343203	0.331202	EMC2/CYG	9
GO:004302	MF	GO:004302	ribonucleo	14/932	161/18369	0.034742	0.343203	0.331202	ITCH/SEC6	14
GO:000535	MF	GO:000535	glucose tra	4/932	25/18369	0.035594	0.343203	0.331202	SLC2A12/S	4
GO:001514	MF	GO:001514	hexose trar	4/932	25/18369	0.035594	0.343203	0.331202	SLC2A12/S	4
GO:000552	MF	GO:000552	lamin bindi	3/932	15/18369	0.037508	0.343203	0.331202	NARF/LBR/	3
GO:004853	MF	GO:004853	beta-1,3-g	3/932	15/18369	0.037508	0.343203	0.331202	B3GNT2/B	3
GO:000548	MF	GO:000548	SNAP rece	5/932	37/18369	0.037747	0.343203	0.331202	GOSR2/ST	5
GO:003097	MF	GO:003097	receptor ty	8/932	76/18369	0.038393	0.343203	0.331202	SH2B3/ELN	8
GO:001675	MF	GO:001675	hexosyltrar	16/932	195/18369	0.040004	0.343203	0.331202	RPN1/MGA	16
GO:003592	MF	GO:003592	mRNA 3'-l	4/932	26/18369	0.040432	0.343203	0.331202	ZFP36L1/C	4
GO:000375	MF	GO:000375	peptidyl-p	5/932	38/18369	0.041719	0.343203	0.331202	FKBP11/PP	5
GO:000090	MF	GO:000090	mRNA regi	3/932	16/18369	0.04447	0.343203	0.331202	CPEB3/TYN	3
GO:000105	MF	GO:000105	RNA polym	3/932	16/18369	0.04447	0.343203	0.331202	POLR2K/PO	3
GO:000831	MF	GO:000831	7S RNA bir	2/932	7/18369	0.045555	0.343203	0.331202	SRP9/SRP7	2
GO:003500	MF	GO:003500	1-phospha	2/932	7/18369	0.045555	0.343203	0.331202	PIK3C2A/P	2
GO:004693	MF	GO:004693	phosphatic	2/932	7/18369	0.045555	0.343203	0.331202	PIK3C2A/P	2
GO:009863	MF	GO:009863	cell-matrix	2/932	7/18369	0.045555	0.343203	0.331202	EMILIN1/IT	2
GO:000167	MF	GO:000167	ATPase act	4/932	27/18369	0.045625	0.343203	0.331202	TOR1AIP2/	4
GO:000819	MF	GO:000819	ferrous iror	4/932	27/18369	0.045625	0.343203	0.331202	ISCU/SNC	4
GO:001662	MF	GO:001662	oxidoreduc	5/932	39/18369	0.045934	0.343203	0.331202	DLAT/FAR1	5
GO:001982	MF	GO:001982	oxygen bin	5/932	39/18369	0.045934	0.343203	0.331202	CYGB/NOX	5
GO:003329	MF	GO:003329	monocarbo	8/932	80/18369	0.049612	0.343203	0.331202	PTGDS/CY	8
GO:004691	MF	GO:004691	transition r	5/932	40/18369	0.050394	0.343203	0.331202	ATP2C1/SL	5
GO:000213	MF	GO:000213	CTP bindin	1/932	1/18369	0.050738	0.343203	0.331202	HSP90AA1	1
GO:000382	MF	GO:000382	alpha-1,3-	1/932	1/18369	0.050738	0.343203	0.331202	MGAT1	1
GO:000386	MF	GO:000386	3-hydroxyi	1/932	1/18369	0.050738	0.343203	0.331202	HIBCH	1
GO:000394	MF	GO:000394	(N-acetyln	1/932	1/18369	0.050738	0.343203	0.331202	B4GALNT1	1
GO:000395	MF	GO:000395	NAD(P)+ tı	1/932	1/18369	0.050738	0.343203	0.331202	NNT	1
GO:000397	MF	GO:000397	UDP-N-ac	1/932	1/18369	0.050738	0.343203	0.331202	DPAGT1	1
GO:000400	MF	GO:000400	adenosine	1/932	1/18369	0.050738	0.343203	0.331202	ADK	1
GO:000410	MF	GO:000410	choline O-	1/932	1/18369	0.050738	0.343203	0.331202	CHAT	1
GO:000439	MF	GO:000439	heparan su	1/932	1/18369	0.050738	0.343203	0.331202	HS2ST1	1
GO:000451	MF	GO:000451	nicotinate	1/932	1/18369	0.050738	0.343203	0.331202	NAPRT	1
GO:000457	MF	GO:000457	Glc3Man9	1/932	1/18369	0.050738	0.343203	0.331202	MOGS	1
GO:000461	MF	GO:000461	phosphoac	1/932	1/18369	0.050738	0.343203	0.331202	PGM3	1
GO:000461	MF	GO:000461	phosphogl	1/932	1/18369	0.050738	0.343203	0.331202	PGD	1
GO:000474	MF	GO:000474	dihydrolipc	1/932	1/18369	0.050738	0.343203	0.331202	DLAT	1
GO:000475	MF	GO:000475	sepiapterir	1/932	1/18369	0.050738	0.343203	0.331202	SPR	1
GO:000479	MF	GO:000479	thymidylat	1/932	1/18369	0.050738	0.343203	0.331202	TYMS	1
GO:000504	MF	GO:000504	netrin rece	1/932	1/18369	0.050738	0.343203	0.331202	UNC5C	1
GO:000512	MF	GO:000512	Toll bindin	1/932	1/18369	0.050738	0.343203	0.331202	MYD88	1
GO:000546	MF	GO:000546	UDP-gluco	1/932	1/18369	0.050738	0.343203	0.331202	SLC35B1	1
GO:000812	MF	GO:000812	quercetin 2	1/932	1/18369	0.050738	0.343203	0.331202	PIR	1
GO:000839	MF	GO:000839	testosteror	1/932	1/18369	0.050738	0.343203	0.331202	CYP2B6	1
GO:000845	MF	GO:000845	alpha-1,6-	1/932	1/18369	0.050738	0.343203	0.331202	MGAT2	1
GO:000848	MF	GO:000848	gamma-gl	1/932	1/18369	0.050738	0.343203	0.331202	GGCX	1
GO:000850	MF	GO:000850	sodium:iod	1/932	1/18369	0.050738	0.343203	0.331202	SLC5A5	1
GO:000852	MF	GO:000852	acetyl-Co	1/932	1/18369	0.050738	0.343203	0.331202	SLC33A1	1
GO:000874	MF	GO:000874	NAD(P)+ tı	1/932	1/18369	0.050738	0.343203	0.331202	NNT	1
GO:001043	MF	GO:001043	9,10 (9', 10	1/932	1/18369	0.050738	0.343203	0.331202	BCO2	1
GO:001085	MF	GO:001085	calcium-de	1/932	1/18369	0.050738	0.343203	0.331202	CAST	1
GO:001615	MF	GO:001615	pyrimidine	1/932	1/18369	0.050738	0.343203	0.331202	TYMP	1
GO:001665	MF	GO:001665	oxidoreduc	1/932	1/18369	0.050738	0.343203	0.331202	NNT	1
GO:001704	MF	GO:001704	galactosylg	1/932	1/18369	0.050738	0.343203	0.331202	GLA	1
GO:001706	MF	GO:001706	single-stra	1/932	1/18369	0.050738	0.343203	0.331202	SMUG1	1
GO:001846	MF	GO:001846	formaldehy	1/932	1/18369	0.050738	0.343203	0.331202	ADH5	1
GO:001978	MF	GO:001978	FAT10 activ	1/932	1/18369	0.050738	0.343203	0.331202	UBA6	1
GO:003010	MF	GO:003010	HLA-A spe	1/932	1/18369	0.050738	0.343203	0.331202	LILRB1	1
GO:003037	MF	GO:003037	neurotensi	1/932	1/18369	0.050738	0.343203	0.331202	SORT1	1
GO:003196	MF	GO:003196	nuclear mir	1/932	1/18369	0.050738	0.343203	0.331202	SMAD3	1
GO:003244	MF	GO:003244	2-alkenal r	1/932	1/18369	0.050738	0.343203	0.331202	PTGR1	1
GO:003256	MF	GO:003256	dATP bindi	1/932	1/18369	0.050738	0.343203	0.331202	HSP90AA1	1
GO:003392	MF	GO:003392	mannosyl-	1/932	1/18369	0.050738	0.343203	0.331202	ENGASE	1
GO:003522	MF	GO:003522	glutamate-	1/932	1/18369	0.050738	0.343203	0.331202	GCLM	1
GO:003524	MF	GO:003524	alpha-1,4-	1/932	1/18369	0.050738	0.343203	0.331202	EXTL2	1
GO:003579	MF	GO:003579	2-alkenal r	1/932	1/18369	0.050738	0.343203	0.331202	PTGR1	1
GO:003613	MF	GO:003613	prostaglan	1/932	1/18369	0.050738	0.343203	0.331202	AKR1C3	1
GO:003618	MF	GO:003618	13-lipoxin	1/932	1/18369	0.050738	0.343203	0.331202	PTGR1	1
GO:003631	MF	GO:003631	tyrosyl-RN	1/932	1/18369	0.050738	0.343203	0.331202	TDP2	1
GO:003802	MF	GO:003802	leptin rece	1/932	1/18369	0.050738	0.343203	0.331202	LEPR	1
GO:004208	MF	GO:004208	5,10-meth	1/932	1/18369	0.050738	0.343203	0.331202	TYMS	1
GO:004395	MF	GO:004395	3-hydroxy	1/932	1/18369	0.050738	0.343203	0.331202	ECHS1	1
GO:004460	MF	GO:004460	protein ade	1/932	1/18369	0.050738	0.343203	0.331202	FICD	1
GO:004570	MF	GO:004570	ketoreduct	1/932	1/18369	0.050738	0.343203	0.331202	AKR1C3	1
GO:004652	MF	GO:004652	xylosylprot	1/932	1/18369	0.050738	0.343203	0.331202	B4GALT7	1
GO:004653	MF	GO:004653	histamine	1/932	1/18369	0.050738	0.343203	0.331202	HNMT	1
GO:004657	MF	GO:004657	long-chain	1/932	1/18369	0.050738	0.343203	0.331202	ALDH3A2	1
GO:004702	MF	GO:004702	7-beta-hy	1/932	1/18369	0.050738	0.343203	0.331202	HSD11B1	1
GO:004732	MF	GO:004732	[3-methyl-	1/932	1/18369	0.050738	0.343203	0.331202	BCKDK	1
GO:004756	MF	GO:004756	3-dehydro	1/932	1/18369	0.050738	0.343203	0.331202	KDSR	1
GO:004788	MF	GO:004788	fatty acid	1/932	1/18369	0.050738	0.343203	0.331202	CYGB	1
GO:004791	MF	GO:004791	ganglioside	1/932	1/18369	0.050738	0.343203	0.331202	B3GALT4	1
GO:005006	MF	GO:005006	long-chain	1/932	1/18369	0.050738	0.343203	0.331202	ALDH3A2	1
GO:005011	MF	GO:005011	myosin-lig	1/932	1/18369	0.050738	0.343203	0.331202	PPP1CB	1
GO:005197	MF	GO:005197	lysophosp	1/932	1/18369	0.050738	0.343203	0.331202	MFSD2A	1
GO:005259	MF	GO:005259	sn-glycero	1/932	1/18369	0.050738	0.343203	0.331202	GPD2	1
GO:005259	MF	GO:005259	sn-glycero	1/932	1/18369	0.050738	0.343203	0.331202	GPD2	1
GO:005269	MF	GO:005269	raffinose a	1/932	1/18369	0.050738	0.343203	0.331202	GLA	1
GO:005275	MF	GO:005275	GDP-manr	1/932	1/18369	0.050738	0.343203	0.331202	NUDT22	1
GO:005281	MF	GO:005281	medium-c	1/932	1/18369	0.050738	0.343203	0.331202	ALDH3A2	1
GO:005285	MF	GO:005285	peptidyl-ly	1/932	1/18369	0.050738	0.343203	0.331202	NAA50	1
GO:005289	MF	GO:005289	N(1),N(12)	1/932	1/18369	0.050738	0.343203	0.331202	PAOX	1
GO:005290	MF	GO:005290	spermidine	1/932	1/18369	0.050738	0.343203	0.331202	PAOX	1
GO:005290	MF	GO:005290	N1-acetyls	1/932	1/18369	0.050738	0.343203	0.331202	PAOX	1
GO:005290	MF	GO:005290	N1-acetyls	1/932	1/18369	0.050738	0.343203	0.331202	PAOX	1
GO:005292	MF	GO:005292	dol-P-Mar	1/932	1/18369	0.050738	0.343203	0.331202	ALG3	1
GO:006017	MF	GO:006017	brain-deriv	1/932	1/18369	0.050738	0.343203	0.331202	NTRK2	1
GO:007026	MF	GO:007026	5'-tyrosyl-	1/932	1/18369	0.050738	0.343203	0.331202	TDP2	1
GO:007052	MF	GO:007052	11-beta-h	1/932	1/18369	0.050738	0.343203	0.331202	HSD11B1	1
GO:007097	MF	GO:007097	TIR domair	1/932	1/18369	0.050738	0.343203	0.331202	MYD88	1
GO:008606	MF	GO:008606	voltage-ga	1/932	1/18369	0.050738	0.343203	0.331202	SCN5A	1
GO:008606	MF	GO:008606	voltage-ga	1/932	1/18369	0.050738	0.343203	0.331202	SCN5A	1
GO:008606	MF	GO:008606	voltage-ga	1/932	1/18369	0.050738	0.343203	0.331202	SCN5A	1
GO:009725	MF	GO:009725	leukotriene	1/932	1/18369	0.050738	0.343203	0.331202	PTGR1	1
GO:009757	MF	GO:009757	glutathione	1/932	1/18369	0.050738	0.343203	0.331202	GLRX	1
GO:010207	MF	GO:010207	beta,beta-	1/932	1/18369	0.050738	0.343203	0.331202	BCO2	1
GO:010219	MF	GO:010219	cortisol del	1/932	1/18369	0.050738	0.343203	0.331202	HSD11B1	1
GO:010632	MF	GO:010632	S-(hydroxy	1/932	1/18369	0.050738	0.343203	0.331202	ADH5	1
GO:010632	MF	GO:010632	S-(hydroxy	1/932	1/18369	0.050738	0.343203	0.331202	ADH5	1
GO:010637	MF	GO:010637	protein-L-	1/932	1/18369	0.050738	0.343203	0.331202	METTL9	1
GO:014034	MF	GO:014034	lysophosp	1/932	1/18369	0.050738	0.343203	0.331202	MFSD2A	1
GO:014076	MF	GO:014076	NAD-depe	1/932	1/18369	0.050738	0.343203	0.331202	SIRT6	1
GO:199014	MF	GO:199014	talin bindin	1/932	1/18369	0.050738	0.343203	0.331202	CORO2B	1
GO:199044	MF	GO:199044	exon-exon	1/932	1/18369	0.050738	0.343203	0.331202	CPSF6	1
GO:001514	MF	GO:001514	monosaccl	4/932	28/18369	0.051171	0.343203	0.331202	SLC2A12/S	4
GO:001662	MF	GO:001662	oxidoreduc	4/932	28/18369	0.051171	0.343203	0.331202	AKR1C3/LE	4
GO:003525	MF	GO:003525	UDP-galac	4/932	28/18369	0.051171	0.343203	0.331202	B3GNT2/B	4
GO:004418	MF	GO:004418	protein fol	7/932	67/18369	0.052657	0.351533	0.339242	HSPB6/HS	7
GO:001666	MF	GO:001666	oxidoreduc	6/932	54/18369	0.054835	0.364384	0.351643	PDIA6/DN	6
GO:001685	MF	GO:001685	cis-trans is	5/932	41/18369	0.055098	0.364454	0.35171	FKBP11/PP	5
GO:000184	MF	GO:000184	compleme	2/932	8/18369	0.058734	0.37642	0.363258	PTX3/CALF	2
GO:001979	MF	GO:001979	procollage	2/932	8/18369	0.058734	0.37642	0.363258	P4HA1/P4	2
GO:002302	MF	GO:002302	MHC class	2/932	8/18369	0.058734	0.37642	0.363258	TAP1/LILR	2
GO:003154	MF	GO:003154	peptidyl-p	2/932	8/18369	0.058734	0.37642	0.363258	P4HA1/P4	2
GO:003199	MF	GO:003199	insulin-like	2/932	8/18369	0.058734	0.37642	0.363258	IGFBP3/IG	2
GO:003330	MF	GO:003330	dehydroas	2/932	8/18369	0.058734	0.37642	0.363258	SLC2A3/SL	2
GO:005192	MF	GO:005192	peroxiredo	2/932	8/18369	0.058734	0.37642	0.363258	PRDX6/PR	2
GO:000197	MF	GO:000197	retinoic aci	3/932	18/18369	0.06015	0.382096	0.368736	CYP26B1/	3
GO:003354	MF	GO:003354	MAP kinas	3/932	18/18369	0.06015	0.382096	0.368736	DUSP10/D	3
GO:000516	MF	GO:000516	tumor necr	4/932	30/18369	0.063313	0.39289	0.379152	TNFSF9/ST	4
GO:005111	MF	GO:005111	sugar trans	4/932	30/18369	0.063313	0.39289	0.379152	SLC2A12/S	4
GO:001514	MF	GO:001514	carbohydra	5/932	43/18369	0.06524	0.39289	0.379152	SLC2A12/S	5
GO:004815	MF	GO:004815	tau protein	5/932	43/18369	0.06524	0.39289	0.379152	BAG2/MA	5
GO:000552	MF	GO:000552	insulin-like	3/932	19/18369	0.06883	0.39289	0.379152	IGFBP3/IG	3
GO:000196	MF	GO:000196	fibronectin	4/932	31/18369	0.069901	0.39289	0.379152	TNFAIP6/I	4
GO:000837	MF	GO:000837	galactosylt	4/932	31/18369	0.069901	0.39289	0.379152	B3GNT2/B	4
GO:005111	MF	GO:005111	ATPase bir	8/932	86/18369	0.070081	0.39289	0.379152	SYVN1/TO	8
GO:000813	MF	GO:000813	protein tyr	5/932	44/18369	0.070677	0.39289	0.379152	DUSP10/P	5
GO:000511	MF	GO:000511	type II tran	2/932	9/18369	0.073033	0.39289	0.379152	TGFB2/EN	2
GO:000538	MF	GO:000538	iron ion tra	2/932	9/18369	0.073033	0.39289	0.379152	SLC25A37/	2
GO:000843	MF	GO:000843	selenium b	2/932	9/18369	0.073033	0.39289	0.379152	GPX4/GPX	2
GO:001516	MF	GO:001516	pyrimidine	2/932	9/18369	0.073033	0.39289	0.379152	SLC35A3/S	2
GO:001920	MF	GO:001920	nucleoside	2/932	9/18369	0.073033	0.39289	0.379152	CMPK1/AD	2
GO:003049	MF	GO:003049	hemoglobi	2/932	9/18369	0.073033	0.39289	0.379152	HBB/HBD	2
GO:003143	MF	GO:003143	mitogen-a	2/932	9/18369	0.073033	0.39289	0.379152	TRIB3/DLG	2
GO:004749	MF	GO:004749	calcium-in	2/932	9/18369	0.073033	0.39289	0.379152	PRDX6/PL	2
GO:005065	MF	GO:005065	3'-phosph	2/932	9/18369	0.073033	0.39289	0.379152	CHST12/SU	2
GO:005281	MF	GO:005281	phosphatic	2/932	9/18369	0.073033	0.39289	0.379152	PIK3C2A/P	2
GO:004317	MF	GO:004317	organic aci	16/932	212/18369	0.073656	0.39289	0.379152	PTGDS/SE	16
GO:001661	MF	GO:001661	oxidoreduc	11/932	133/18369	0.075522	0.39289	0.379152	GPD2/KDS	11
GO:000122	MF	GO:000122	transcriptic	5/932	45/18369	0.076354	0.39289	0.379152	NEK6/ZBT	5
GO:001989	MF	GO:001989	kinesin bin	5/932	45/18369	0.076354	0.39289	0.379152	NEK6/RAB	5
GO:001900	MF	GO:001900	GDP bindir	7/932	73/18369	0.076454	0.39289	0.379152	RAP2A/RA	7
GO:001031	MF	GO:001031	phosphatic	3/932	20/18369	0.07804	0.39289	0.379152	PHLDA3/P	3
GO:001601	MF	GO:001601	cyclosporir	3/932	20/18369	0.07804	0.39289	0.379152	PPIB/PPIL1	3
GO:001707	MF	GO:001707	syntaxin-1	3/932	20/18369	0.07804	0.39289	0.379152	SNAP23/C	3
GO:000369	MF	GO:000369	single-stra	10/932	119/18369	0.080471	0.39289	0.379152	SMC4/FUB	10
GO:000517	MF	GO:000517	integrin bir	12/932	151/18369	0.082717	0.39289	0.379152	ITGBL1/EM	12
GO:001662	MF	GO:001662	oxidoreduc	6/932	60/18369	0.082796	0.39289	0.379152	SDHD/AKR	6
GO:000520	MF	GO:000520	extracellula	13/932	167/18369	0.082874	0.39289	0.379152	EMILIN1/M	13
GO:004802	MF	GO:004802	monosaccl	7/932	75/18369	0.085582	0.39289	0.379152	LMAN1/EN	7
GO:000550	MF	GO:000550	iron ion bir	12/932	152/18369	0.085893	0.39289	0.379152	CYGB/ACC	12
GO:000184	MF	GO:000184	opsonin bi	3/932	21/18369	0.087755	0.39289	0.379152	PTX3/ITGA	3
GO:004302	MF	GO:004302	cysteine-ty	3/932	21/18369	0.087755	0.39289	0.379152	BIRC3/SNC	3
GO:000833	MF	GO:000833	protein tyr	2/932	10/18369	0.088305	0.39289	0.379152	DUSP10/D	2
GO:001524	MF	GO:001524	fatty acid t	2/932	10/18369	0.088305	0.39289	0.379152	MFSD2A/S	2
GO:001630	MF	GO:001630	1-phospha	2/932	10/18369	0.088305	0.39289	0.379152	PIK3C2A/P	2
GO:003172	MF	GO:003172	haptoglobi	2/932	10/18369	0.088305	0.39289	0.379152	HBB/HBD	2
GO:003355	MF	GO:003355	MAP kinas	2/932	10/18369	0.088305	0.39289	0.379152	DUSP10/D	2
GO:004312	MF	GO:004312	neurotroph	2/932	10/18369	0.088305	0.39289	0.379152	NTRK2/SO	2
GO:004713	MF	GO:004713	protein-dis	2/932	10/18369	0.088305	0.39289	0.379152	PGK1/TXN	2
GO:005281	MF	GO:005281	phosphatic	2/932	10/18369	0.088305	0.39289	0.379152	PIK3C2A/P	2
GO:007085	MF	GO:007085	growth fac	11/932	137/18369	0.088772	0.39289	0.379152	GREM1/ER	11

GO:005101	MF	GO:005101	actin filam	15/932	202/18369	0.090303	0.39289	0.379152	BLOC1S6/	15
GO:000472	MF	GO:000472	protein tyr	8/932	91/18369	0.090563	0.39289	0.379152	DUSP10/P	8
GO:001687	MF	GO:001687	ligase activ	5/932	48/18369	0.094806	0.39289	0.379152	GCLM/NA	5
GO:001690	MF	GO:001690	oxidoreduc	5/932	48/18369	0.094806	0.39289	0.379152	DLAT/FAR	5
GO:003069	MF	GO:003069	GTPase reg	32/932	495/18369	0.095598	0.39289	0.379152	RP2/TBC1D	32
GO:006058	MF	GO:006058	nucleoside	32/932	495/18369	0.095598	0.39289	0.379152	RP2/TBC1D	32
GO:001688	MF	GO:001688	acid-amin	3/932	22/18369	0.097949	0.39289	0.379152	GCLM/TTL	3
GO:003501	MF	GO:003501	phosphatic	3/932	22/18369	0.097949	0.39289	0.379152	SOCS6/WD	3
GO:000164	MF	GO:000164	group II m	1/932	2/18369	0.098904	0.39289	0.379152	GRM2	1
GO:000187	MF	GO:000187	(1->3)-be	1/932	2/18369	0.098904	0.39289	0.379152	PTX3	1
GO:000188	MF	GO:000188	glucuronyl-	1/932	2/18369	0.098904	0.39289	0.379152	EXTL2	1
GO:000213	MF	GO:000213	UTP bindin	1/932	2/18369	0.098904	0.39289	0.379152	HSP90AA1	1
GO:000384	MF	GO:000384	11-beta-h	1/932	2/18369	0.098904	0.39289	0.379152	HSD11B1	1
GO:000387	MF	GO:000387	5-aminole	1/932	2/18369	0.098904	0.39289	0.379152	ALAS1	1
GO:000395	MF	GO:000395	NAD+ kina	1/932	2/18369	0.098904	0.39289	0.379152	NADK2	1
GO:000406	MF	GO:000406	asparagine	1/932	2/18369	0.098904	0.39289	0.379152	ASNSD1	1
GO:000434	MF	GO:000434	glucose-6-	1/932	2/18369	0.098904	0.39289	0.379152	G6PD	1
GO:000436	MF	GO:000436	glycerol-3-	1/932	2/18369	0.098904	0.39289	0.379152	GPD2	1
GO:000455	MF	GO:000455	alpha-gala	1/932	2/18369	0.098904	0.39289	0.379152	GLA	1
GO:000457	MF	GO:000457	N-acetylgl	1/932	2/18369	0.098904	0.39289	0.379152	ALG13	1
GO:000461	MF	GO:000461	phosphogl	1/932	2/18369	0.098904	0.39289	0.379152	PGK1	1
GO:000464	MF	GO:000464	poly(ADP-	1/932	2/18369	0.098904	0.39289	0.379152	PARG	1
GO:000472	MF	GO:000472	prenylated	1/932	2/18369	0.098904	0.39289	0.379152	PTP4A2	1
GO:000475	MF	GO:000475	selenide, w	1/932	2/18369	0.098904	0.39289	0.379152	SEPHS2	1
GO:000480	MF	GO:000480	transposas	1/932	2/18369	0.098904	0.39289	0.379152	PGBD5	1
GO:000481	MF	GO:000481	cysteine-tR	1/932	2/18369	0.098904	0.39289	0.379152	CARS2	1
GO:000491	MF	GO:000491	interleukin-	1/932	2/18369	0.098904	0.39289	0.379152	IL4R	1
GO:000492	MF	GO:000492	leukemia in	1/932	2/18369	0.098904	0.39289	0.379152	LIFR	1
GO:000504	MF	GO:000504	netrin rece	1/932	2/18369	0.098904	0.39289	0.379152	UNC5C	1
GO:000515	MF	GO:000515	interleukin-	1/932	2/18369	0.098904	0.39289	0.379152	ERAP1	1
GO:000812	MF	GO:000812	4-alpha-hy	1/932	2/18369	0.098904	0.39289	0.379152	PCBD2	1
GO:000814	MF	GO:000814	structural c	1/932	2/18369	0.098904	0.39289	0.379152	MGP	1
GO:000816	MF	GO:000816	protein tyro	1/932	2/18369	0.098904	0.39289	0.379152	IGFBP3	1
GO:000826	MF	GO:000826	pyrimidine	1/932	2/18369	0.098904	0.39289	0.379152	MBD4	1
GO:000876	MF	GO:000876	UDP-sugar	1/932	2/18369	0.098904	0.39289	0.379152	NUDT22	1
GO:000903	MF	GO:000903	thymidine	1/932	2/18369	0.098904	0.39289	0.379152	TYMP	1
GO:001043	MF	GO:001043	carotenoid	1/932	2/18369	0.098904	0.39289	0.379152	BCO2	1
GO:001046	MF	GO:001046	nerve grow	1/932	2/18369	0.098904	0.39289	0.379152	SORT1	1
GO:001542	MF	GO:001542	ABC-type	1/932	2/18369	0.098904	0.39289	0.379152	ABCD4	1
GO:001545	MF	GO:001545	protein-tra	1/932	2/18369	0.098904	0.39289	0.379152	SEC61B	1
GO:001641	MF	GO:001641	S-acetyltra	1/932	2/18369	0.098904	0.39289	0.379152	DLAT	1
GO:001674	MF	GO:001674	N-succinyl	1/932	2/18369	0.098904	0.39289	0.379152	ALAS1	1
GO:001678	MF	GO:001678	phosphotra	1/932	2/18369	0.098904	0.39289	0.379152	SEPHS2	1
GO:001690	MF	GO:001690	oxidoreduc	1/932	2/18369	0.098904	0.39289	0.379152	GPD2	1
GO:001709	MF	GO:001709	sulfonylure	1/932	2/18369	0.098904	0.39289	0.379152	HSP90AA1	1
GO:003010	MF	GO:003010	HLA-B spe	1/932	2/18369	0.098904	0.39289	0.379152	LILRB1	1
GO:003169	MF	GO:003169	beta-3 adr	1/932	2/18369	0.098904	0.39289	0.379152	ARRDC3	1
GO:003212	MF	GO:003212	histone dea	1/932	2/18369	0.098904	0.39289	0.379152	SIRT6	1
GO:003242	MF	GO:003242	GBD doma	1/932	2/18369	0.098904	0.39289	0.379152	CDC42	1
GO:003386	MF	GO:003386	UMP kinas	1/932	2/18369	0.098904	0.39289	0.379152	CMPK1	1
GO:003387	MF	GO:003387	hormone-s	1/932	2/18369	0.098904	0.39289	0.379152	ABHD2	1
GO:003404	MF	GO:003404	ABC-type	1/932	2/18369	0.098904	0.39289	0.379152	ABCG4	1
GO:003613	MF	GO:003613	prostaglan	1/932	2/18369	0.098904	0.39289	0.379152	AKR1C3	1
GO:003643	MF	GO:003643	CMP kinas	1/932	2/18369	0.098904	0.39289	0.379152	CMPK1	1
GO:003643	MF	GO:003643	dCMP kina	1/932	2/18369	0.098904	0.39289	0.379152	CMPK1	1
GO:003650	MF	GO:003650	prosaposin	1/932	2/18369	0.098904	0.39289	0.379152	GPR37L1	1
GO:004318	MF	GO:004318	vascular en	1/932	2/18369	0.098904	0.39289	0.379152	VEGFC	1
GO:004555	MF	GO:004555	geranylger	1/932	2/18369	0.098904	0.39289	0.379152	AKR1C3	1
GO:004696	MF	GO:004696	NAD-depe	1/932	2/18369	0.098904	0.39289	0.379152	SIRT6	1
GO:004701	MF	GO:004701	prostaglan	1/932	2/18369	0.098904	0.39289	0.379152	AKR1C3	1
GO:004702	MF	GO:004702	15-hydroxy	1/932	2/18369	0.098904	0.39289	0.379152	AKR1C3	1
GO:004706	MF	GO:004706	phospholip	1/932	2/18369	0.098904	0.39289	0.379152	GPX4	1
GO:004729	MF	GO:004729	(alpha-N-a	1/932	2/18369	0.098904	0.39289	0.379152	ST6GALNA	1
GO:004774	MF	GO:004774	cholate-Co	1/932	2/18369	0.098904	0.39289	0.379152	SLC27A2	1
GO:004778	MF	GO:004778	citrate deh	1/932	2/18369	0.098904	0.39289	0.379152	ACO2	1
GO:004778	MF	GO:004778	delta4-3-o	1/932	2/18369	0.098904	0.39289	0.379152	AKR1C3	1
GO:005019	MF	GO:005019	phytanate-	1/932	2/18369	0.098904	0.39289	0.379152	SLC27A2	1
GO:005061	MF	GO:005061	delta14-ste	1/932	2/18369	0.098904	0.39289	0.379152	LBR	1
GO:005190	MF	GO:005190	S-(hydroxy	1/932	2/18369	0.098904	0.39289	0.379152	ADH5	1
GO:005290	MF	GO:005290	spermine:c	1/932	2/18369	0.098904	0.39289	0.379152	PAOX	1
GO:006165	MF	GO:006165	NEDD8 co	1/932	2/18369	0.098904	0.39289	0.379152	UBE2F	1
GO:006176	MF	GO:006176	ribosylnico	1/932	2/18369	0.098904	0.39289	0.379152	NMRK1	1
GO:006210	MF	GO:006210	peptidyl-a	1/932	2/18369	0.098904	0.39289	0.379152	ASPH	1
GO:006218	MF	GO:006218	testosteron	1/932	2/18369	0.098904	0.39289	0.379152	CYP2B6	1
GO:007025	MF	GO:007025	pristanate-	1/932	2/18369	0.098904	0.39289	0.379152	SLC27A2	1
GO:007025	MF	GO:007025	tyrosyl-DN	1/932	2/18369	0.098904	0.39289	0.379152	TDP2	1
GO:007073	MF	GO:007073	protein ade	1/932	2/18369	0.098904	0.39289	0.379152	FICD	1
GO:007188	MF	GO:007188	N-termina	1/932	2/18369	0.098904	0.39289	0.379152	NTMT1	1
GO:007188	MF	GO:007188	1-(4-iodo-	1/932	2/18369	0.098904	0.39289	0.379152	HTR2A	1
GO:008001	MF	GO:008001	fatty-acyl-	1/932	2/18369	0.098904	0.39289	0.379152	FAR1	1
GO:008606	MF	GO:008606	voltage-ga	1/932	2/18369	0.098904	0.39289	0.379152	SCN5A	1
GO:009715	MF	GO:009715	pre-mRNA	1/932	2/18369	0.098904	0.39289	0.379152	RBM4	1
GO:009737	MF	GO:009737	NAD-depe	1/932	2/18369	0.098904	0.39289	0.379152	SIRT6	1
GO:009864	MF	GO:009864	integrin bin	1/932	2/18369	0.098904	0.39289	0.379152	EMILIN1	1
GO:010296	MF	GO:010296	alcohol-fo	1/932	2/18369	0.098904	0.39289	0.379152	FAR1	1
GO:011015	MF	GO:011015	RNA NAD-	1/932	2/18369	0.098904	0.39289	0.379152	NUDT12	1
GO:012009	MF	GO:012009	crotonyl-C	1/932	2/18369	0.098904	0.39289	0.379152	ECHS1	1
GO:014061	MF	GO:014061	P-type ma	1/932	2/18369	0.098904	0.39289	0.379152	ATP2C1	1
GO:014077	MF	GO:014077	NAD-depe	1/932	2/18369	0.098904	0.39289	0.379152	SIRT6	1
GO:014077	MF	GO:014077	NAD-depe	1/932	2/18369	0.098904	0.39289	0.379152	SIRT6	1
GO:199017	MF	GO:199017	phosphodi	1/932	2/18369	0.098904	0.39289	0.379152	NUDT12	1
GO:199093	MF	GO:199093	mRNA N6-	1/932	2/18369	0.098904	0.39289	0.379152	ALKBH5	1
GO:199084	MF	GO:199084	promoter-	6/932	63/18369	0.099258	0.393215	0.379466	STAT1/GA	6
GO:009048	MF	GO:009048	vitamin tra	4/932	35/18369	0.099543	0.393263	0.379512	SLC22A14/	4
GO:190150	MF	GO:190150	carbohydra	5/932	49/18369	0.101418	0.398322	0.384394	MFSD2A/S	5
GO:000028	MF	GO:000028	magnesium	16/932	223/18369	0.103293	0.398322	0.384394	RP2/RAP2	16
GO:000533	MF	GO:000533	nucleotide	2/932	11/18369	0.104415	0.398322	0.384394	SLC35A3/S	2
GO:000553	MF	GO:000553	glucose bin	2/932	11/18369	0.104415	0.398322	0.384394	G6PD/SLC	2
GO:000819	MF	GO:000819	ferric iron	2/932	11/18369	0.104415	0.398322	0.384394	TF/FTL	2
GO:001685	MF	GO:001685	racemase	2/932	11/18369	0.104415	0.398322	0.384394	DSE/DSEL	2
GO:003199	MF	GO:003199	thioesteras	2/932	11/18369	0.104415	0.398322	0.384394	TRAF4/CD	2
GO:003203	MF	GO:003203	myosin hea	2/932	11/18369	0.104415	0.398322	0.384394	MYL4/MYL	2
GO:004840	MF	GO:004840	platelet-de	2/932	11/18369	0.104415	0.398322	0.384394	PDGFA/CC	2
GO:005273	MF	GO:005273	phosphatic	2/932	11/18369	0.104415	0.398322	0.384394	PLA2G15/	2
GO:010254	MF	GO:010254	phosphatic	2/932	11/18369	0.104415	0.398322	0.384394	PLA2G15/	2
GO:014066	MF	GO:014066	annealing	2/932	11/18369	0.104415	0.398322	0.384394	FXR1/REC	2
GO:199045	MF	GO:199045	transferrin	2/932	11/18369	0.104415	0.398322	0.384394	CHUK/TF	2
GO:004353	MF	GO:004353	ADP bindir	4/932	36/18369	0.107734	0.406737	0.392515	ME1/TAP1/	4
GO:009771	MF	GO:009771	disordered	4/932	36/18369	0.107734	0.406737	0.392515	RB1/HSP90	4
GO:000516	MF	GO:000516	transformi	3/932	23/18369	0.108595	0.406737	0.392515	TGFB2/EN	3
GO:001529	MF	GO:001529	anion:catio	3/932	23/18369	0.108595	0.406737	0.392515	SLC5A5/SL	3
GO:005043	MF	GO:005043	transformi	3/932	23/18369	0.108595	0.406737	0.392515	ENG/TSKU	3
GO:005069	MF	GO:005069	WW doma	3/932	23/18369	0.108595	0.406737	0.392515	TREX1/WB	3
GO:014010	MF	GO:014010	catalytic ac	3/932	23/18369	0.108595	0.406737	0.392515	MGAT2/M	3
GO:006198	MF	GO:006198	regulatory	5/932	51/18369	0.115307	0.430759	0.415697	SOX4/MBD	5
GO:000508	MF	GO:000508	guanyl-nu	16/932	227/18369	0.115663	0.43081	0.415747	CYTH1/AR	16
GO:003519	MF	GO:003519	miRNA bin	4/932	37/18369	0.116217	0.43081	0.415747	SOX4/ZC3	4
GO:006165	MF	GO:006165	ubiquitin-li	4/932	37/18369	0.116217	0.43081	0.415747	UBE2F/UB	4
GO:009711	MF	GO:009711	scaffold pr	6/932	66/18369	0.117313	0.433759	0.418592	TREM1/CH	6
GO:001920	MF	GO:001920	kinase regu	15/932	211/18369	0.118379	0.436578	0.421313	GREM1/TR	15
GO:000105	MF	GO:000105	RNA polym	2/932	12/18369	0.121237	0.440362	0.424964	POLR2K/P	2
GO:000472	MF	GO:000472	non-meml	2/932	12/18369	0.121237	0.440362	0.424964	PTPN2/PT	2
GO:001666	MF	GO:001666	oxidoreduc	2/932	12/18369	0.121237	0.440362	0.424964	PGK1/TXN	2
GO:003154	MF	GO:003154	peptidyl-p	2/932	12/18369	0.121237	0.440362	0.424964	P4HA1/P4	2
GO:003500	MF	GO:003500	phosphatic	2/932	12/18369	0.121237	0.440362	0.424964	PIK3C2A/P	2
GO:014071	MF	GO:014071	histone cha	2/932	12/18369	0.121237	0.440362	0.424964	ZNHIT1/M	2
GO:004529	MF	GO:004529	cadherin b	22/932	333/18369	0.124846	0.440917	0.4255	TBC1D2/C	22
GO:000550	MF	GO:000550	retinoid bir	4/932	38/18369	0.124982	0.440917	0.4255	PTGDS/CY	4
GO:001679	MF	GO:001679	hydrolase	4/932	38/18369	0.124982	0.440917	0.4255	NTHL1/MB	4
GO:001984	MF	GO:001984	isoprenoid	4/932	38/18369	0.124982	0.440917	0.4255	PTGDS/CY	4
GO:000510	MF	GO:000510	fibroblast	3/932	25/18369	0.131128	0.440917	0.4255	FLRT2/FLR	3
GO:001702	MF	GO:001702	TBP-class	3/932	25/18369	0.131128	0.440917	0.4255	AHR/DR1/	3
GO:000486	MF	GO:000486	cysteine-ty	5/932	54/18369	0.137723	0.440917	0.4255	CSTB/BIRC	5
GO:000486	MF	GO:000486	protein ser	2/932	13/18369	0.138659	0.440917	0.4255	PPP1R14C/	2
GO:001701	MF	GO:001701	MAP kinas	2/932	13/18369	0.138659	0.440917	0.4255	DUSP10/D	2
GO:003423	MF	GO:003423	protein kin	2/932	13/18369	0.138659	0.440917	0.4255	CSK/PRKA	2
GO:004515	MF	GO:004515	myosin II b	2/932	13/18369	0.138659	0.440917	0.4255	MYL4/MYL	2
GO:009715	MF	GO:009715	pre-mRNA	2/932	13/18369	0.138659	0.440917	0.4255	RBM20/RB	2
GO:003140	MF	GO:003140	carboxylic	14/932	200/18369	0.139627	0.440917	0.4255	PTGDS/SE	14
GO:005102	MF	GO:005102	GTPase bir	20/932	304/18369	0.142081	0.440917	0.4255	CYFIP2/SR	20
GO:000419	MF	GO:000419	aspartic-ty	3/932	26/18369	0.142958	0.440917	0.4255	CTSD/CAS	3
GO:000436	MF	GO:000436	glutathione	3/932	26/18369	0.142958	0.440917	0.4255	PTGES/MG	3
GO:005068	MF	GO:005068	nuclear and	3/932	26/18369	0.142958	0.440917	0.4255	FOXP1/ZB	3
GO:001988	MF	GO:001988	protein kin	13/932	184/18369	0.143255	0.440917	0.4255	GREM1/TR	13
GO:000554	MF	GO:000554	phosphatic	4/932	40/18369	0.143307	0.440917	0.4255	PHLDA3/M	4
GO:001702	MF	GO:001702	myosin bin	6/932	70/18369	0.143728	0.440917	0.4255	TRAK2/TO	6
GO:000003	MF	GO:000003	alpha-1,3-	1/932	3/18369	0.144628	0.440917	0.4255	ALG3	1
GO:000021	MF	GO:000021	NAD+ dipl	1/932	3/18369	0.144628	0.440917	0.4255	NUDT12	1
GO:000158	MF	GO:000158	Gq/11-cou	1/932	3/18369	0.144628	0.440917	0.4255	HTR2A	1
GO:000295	MF	GO:000295	ceramide	1/932	3/18369	0.144628	0.440917	0.4255	SAMD8	1
GO:000383	MF	GO:000383	beta-carot	1/932	3/18369	0.144628	0.440917	0.4255	BCO2	1
GO:000397	MF	GO:000397	UDP-N-ac	1/932	3/18369	0.144628	0.440917	0.4255	DPAGT1	1
GO:000399	MF	GO:000399	aconitate	1/932	3/18369	0.144628	0.440917	0.4255	ACO2	1
GO:000409	MF	GO:000409	catalase ac	1/932	3/18369	0.144628	0.440917	0.4255	CYGB	1
GO:000413	MF	GO:000413	deoxyader	1/932	3/18369	0.144628	0.440917	0.4255	ADK	1
GO:000416	MF	GO:000416	delta(3)-de	1/932	3/18369	0.144628	0.440917	0.4255	ECHS1	1
GO:000441	MF	GO:000441	hydroxyacy	1/932	3/18369	0.144628	0.440917	0.4255	PNKD	1
GO:000447	MF	GO:000447	malate del	1/932	3/18369	0.144628	0.440917	0.4255	ME1	1
GO:000447	MF	GO:000447	malate del	1/932	3/18369	0.144628	0.440917	0.4255	ME1	1
GO:000450	MF	GO:000450	phenylalan	1/932	3/18369	0.144628	0.440917	0.4255	PCBD2	1
GO:000492	MF	GO:000492	oncostatin	1/932	3/18369	0.144628	0.440917	0.4255	LIFR	1
GO:000496	MF	GO:000496	endothelin	1/932	3/18369	0.144628	0.440917	0.4255	EDNRB	1
GO:000500	MF	GO:000500	epidermal	1/932	3/18369	0.144628	0.440917	0.4255	EFEMP1	1
GO:000515	MF	GO:000515	macrophag	1/932	3/18369	0.144628	0.440917	0.4255	CSF1	1
GO:000536	MF	GO:000536	taurine:soc	1/932	3/18369	0.144628	0.440917	0.4255	SLC6A6	1
GO:000807	MF	GO:000807	ornithine d	1/932	3/18369	0.144628	0.440917	0.4255	OAZ1	1
GO:000817	MF	GO:000817	succinate c	1/932	3/18369	0.144628	0.440917	0.4255	SDHD	1
GO:000838	MF	GO:000838	IkappaB ki	1/932	3/18369	0.144628	0.440917	0.4255	CHUK	1
GO:000853	MF	GO:000853	oxidized pu	1/932	3/18369	0.144628	0.440917	0.4255	NTHL1	1
GO:000875	MF	GO:000875	NADPH de	1/932	3/18369	0.144628	0.440917	0.4255	NQO1	1
GO:000904	MF	GO:000904	uridylate ki	1/932	3/18369	0.144628	0.440917	0.4255	CMPK1	1
GO:001519	MF	GO:001519	L-tryptoph	1/932	3/18369	0.144628	0.440917	0.4255	SLC36A4	1
GO:001543	MF	GO:001543	ABC-type	1/932	3/18369	0.144628	0.440917	0.4255	TAP1	1
GO:001630	MF	GO:001630	1-phospha	1/932	3/18369	0.144628	0.440917	0.4255	PIP4K2A	1
GO:001674	MF	GO:001674	succinyltra	1/932	3/18369	0.144628	0.440917	0.4255	ALAS1	1
GO:001677	MF	GO:001677	phosphotra	1/932	3/18369	0.144628	0.440917	0.4255	PGK1	1
GO:001689	MF	GO:001689	oxidoreduc	1/932	3/18369	0.144628	0.440917	0.4255	ALDH3A2	1
GO:001694	MF	GO:001694	natriuretic	1/932	3/18369	0.144628	0.440917	0.4255	NPR3	1
GO:001863	MF	GO:001863	phenanthre	1/932	3/18369	0.144628	0.440917	0.4255	AKR1C3	1
GO:001916	MF	GO:001916	trans-2-er	1/932	3/18369	0.144628	0.440917	0.4255	PECR	1
GO:003255	MF	GO:003255	pyrimidine	1/932	3/18369	0.144628	0.440917	0.4255	HSP90AA1	1
GO:003318	MF	GO:003318	sphingomy	1/932	3/18369	0.144628	0.440917	0.4255	SAMD8	1
GO:003359	MF	GO:003359	RNA strand	1/932	3/18369	0.144628	0.440917	0.4255	FXR1	1
GO:003471	MF	GO:003471	type III tra	1/932	3/18369	0.144628	0.440917	0.4255	TGFB2	1
GO:003498	MF	GO:003498	iron chape	1/932	3/18369	0.144628	0.440917	0.4255	TF	1
GO:003613	MF	GO:003613	13-prostag	1/932	3/18369	0.144628	0.440917	0.4255	PTGR1	1
GO:004318	MF	GO:004318	vascular en	1/932	3/18369	0.144628	0.440917	0.4255	VEGFB	1
GO:004659	MF	GO:004659	polyamine	1/932	3/18369	0.144628	0.440917	0.4255	PAOX	1
GO:004697	MF	GO:004697	TAP2 bindi	1/932	3/18369	0.144628	0.440917	0.4255	TAP1	1
GO:004723	MF	GO:004723	glucuronyl	1/932	3/18369	0.144628	0.440917	0.4255	EXTL2	1
GO:004749	MF	GO:004749	ceramide c	1/932	3/18369	0.144628	0.440917	0.4255	SAMD8	1
GO:004752	MF	GO:004752	15-oxopro	1/932	3/18369	0.144628	0.440917	0.4255	PTGR1	1
GO:004789	MF	GO:004789	flavonol 3-	1/932	3/18369	0.144628	0.440917	0.4255	SULT1A1	1
GO:005022	MF	GO:005022	prostaglan	1/932	3/18369	0.144628	0.440917	0.4255	PTGES	1
GO:005026	MF	GO:005026	ribosylnico	1/932	3/18369	0.144628	0.440917	0.4255	NMRK1	1
GO:005153	MF	GO:005153	3 iron, 4 su	1/932	3/18369	0.144628	0.440917	0.4255	ACO2	1
GO:006218	MF	GO:006218	anandamic	1/932	3/18369	0.144628	0.440917	0.4255	CYP2B6	1
GO:006218	MF	GO:006218	anandamic	1/932	3/18369	0.144628	0.440917	0.4255	CYP2B6	1
GO:007053	MF	GO:007053	oleic acid	1/932	3/18369	0.144628	0.440917	0.4255	FABP3	1
GO:007085	MF	GO:007085	myosin VI	1/932	3/18369	0.144628	0.440917	0.4255	TOM1	1
GO:009768	MF	GO:009768	intracellula	1/932	3/18369	0.144628	0.440917	0.4255	TPCN1	1
GO:199076	MF	GO:199076	osmolarity	1/932	3/18369	0.144628	0.440917	0.4255	TMEM63B	1
GO:003149	MF	GO:003149	chromatin	9/932	118/18369	0.145295	0.442016	0.426561	NR1H3/ME	9
GO:001665	MF	GO:001665	oxidoreduc	7/932	87/18369	0.15242	0.462054	0.445898	NDUFA5/N	7
GO:003002	MF	GO:003002	extracellula	4/932	41/18369	0.152843	0.462054	0.445898	COL12A1/	4
GO:014066	MF	GO:014066	ATP-deper	4/932	41/18369	0.152843	0.462054	0.445898	HSPA5/HS	4
GO:003126	MF	GO:003126	small GTPa	18/932	273/18369	0.15502	0.466994	0.450665	CYFIP2/SR	18
GO:007000	MF	GO:007000	aspartic-ty	3/932	27/18369	0.155125	0.466994	0.450665	CTSD/CAS	3
GO:004302	MF	GO:004302	ribosomal	2/932	14/18369	0.156577	0.467461	0.451116	CPSF6/LTN	2
GO:004313	MF	GO:004313	3'-5' DNA	2/932	14/18369	0.156577	0.467461	0.451116	ASCC3/RE	2
GO:007041	MF	GO:007041	I-SMAD bi	2/932	14/18369	0.156577	0.467461	0.451116	CTNNB1/S	2
GO:199082	MF	GO:199082	sequence-	2/932	14/18369	0.156577	0.467461	0.451116	TYMS/SSB	2
GO:002003	MF	GO:002003	heme bind	10/932	137/18369	0.158392	0.471903	0.455402	CYGB/CYP	10
GO:001665	MF	GO:001665	oxidoreduc	5/932	57/18369	0.161886	0.480503	0.463702	NDUFA5/A	5
GO:000392	MF	GO:000392	G protein a	4/932	42/18369	0.162611	0.480503	0.463702	RAP2A/RA	4
GO:000830	MF	GO:000830	structural c	4/932	42/18369	0.162611	0.480503	0.463702	ASPH/TPM	4
GO:004301	MF	GO:004301	alpha-tubu	4/932	42/18369	0.162611	0.480503	0.463702	FAM110C/	4
GO:004302	MF	GO:004302	ribosome b	6/932	73/18369	0.165163	0.485925	0.468934	SEC61G/C	6
GO:000451	MF	GO:000451	endonucle	9/932	122/18369	0.167072	0.485925	0.468934	RNASE4/N	9
GO:004302	MF	GO:004302	cysteine-ty	4/932	43/18369	0.172598	0.485925	0.468934	RPS27L/BI	4
GO:000850	MF	GO:000850	anion trans	9/932	123/18369	0.172744	0.485925	0.468934	SLC5A5/G	9
GO:001661	MF	GO:001661	oxidoreduc	9/932	123/18369	0.172744	0.485925	0.468934	KDSR/ME1	9
GO:001712	MF	GO:001712	SH3 doma	9/932	123/18369	0.172744	0.485925	0.468934	ELMO2/EL	9
GO:000402	MF	GO:000402	aldehyde c	2/932	15/18369	0.174895	0.485925	0.468934	ALDH3A2/	2
GO:007074	MF	GO:007074	C2H2 zinc	2/932	15/18369	0.174895	0.485925	0.468934	EBF1/MBD	2
GO:199022	MF	GO:199022	histone me	2/932	15/18369	0.174895	0.485925	0.468934	CBX3/CTN	2
GO:199038	MF	GO:199038	Lys48-spe	2/932	15/18369	0.174895	0.485925	0.468934	YOD1/DES	2
GO:005121	MF	GO:005121	dioxygenas	7/932	91/18369	0.178897	0.485925	0.468934	PIR/P4HA1	7
GO:000486	MF	GO:000486	protein ph	4/932	44/18369	0.18279	0.485925	0.468934	PPP1R14C/	4
GO:000010	MF	GO:000010	succinate c	1/932	4/18369	0.188035	0.485925	0.468934	SDHD	1
GO:000070	MF	GO:000070	mismatch	1/932	4/18369	0.188035	0.485925	0.468934	MBD4	1
GO:000383	MF	GO:000383	beta-N-ac	1/932	4/18369	0.188035	0.485925	0.468934	B4GALT7	1
GO:000387	MF	GO:000387	6-phospho	1/932	4/18369	0.188035	0.485925	0.468934	PFKFB1	1
GO:000388	MF	GO:000388	DNA (cytos	1/932	4/18369	0.188035	0.485925	0.468934	DNMT1	1
GO:000396	MF	GO:000396	NADPH:qu	1/932	4/18369	0.188035	0.485925	0.468934	CRYZ	1
GO:000412	MF	GO:000412	cytidylate	1/932	4/18369	0.188035	0.485925	0.468934	CMPK1	1
GO:000447	MF	GO:000447	malic enzy	1/932	4/18369	0.188035	0.485925	0.468934	ME1	1
GO:000452	MF	GO:000452	ribonuclea	1/932	4/18369	0.188035	0.485925	0.468934	RNASE4	1
GO:000479	MF	GO:000479	thiosulfate	1/932	4/18369	0.188035	0.485925	0.468934	UBA6	1
GO:000484	MF	GO:000484	uracil DNA	1/932	4/18369	0.188035	0.485925	0.468934	SMUG1	1
GO:000484	MF	GO:000484	uridine kin	1/932	4/18369	0.188035	0.485925	0.468934	CMPK1	1
GO:000489	MF	GO:000489	ciliary neur	1/932	4/18369	0.188035	0.485925	0.468934	LIFR	1
GO:000503	MF	GO:000503	osmosens	1/932	4/18369	0.188035	0.485925	0.468934	TMEM63B	1
GO:000535	MF	GO:000535	galactose	1/932	4/18369	0.188035	0.485925	0.468934	SLC2A3	1
GO:000536	MF	GO:000536	taurine tra	1/932	4/18369	0.188035	0.485925	0.468934	SLC6A6	1
GO:000546	MF	GO:000546	UDP-N-ac	1/932	4/18369	0.188035	0.485925	0.468934	SLC35A3	1
GO:000547	MF	GO:000547	ATP:ADP a	1/932	4/18369	0.188035	0.485925	0.468934	SLC35B1	1
GO:000553	MF	GO:000553	galactose	1/932	4/18369	0.188035	0.485925	0.468934	ENG	1
GO:000845	MF	GO:000845	beta-galac	1/932	4/18369	0.188035	0.485925	0.468934	B3GNT2	1
GO:000894	MF	GO:000894	oxaloaceta	1/932	4/18369	0.188035	0.485925	0.468934	ME1	1
GO:001544	MF	GO:001544	ABC-type	1/932	4/18369	0.188035	0.485925	0.468934	TAP1	1
GO:001640	MF	GO:001640	palmitoyl-	1/932	4/18369	0.188035	0.485925	0.468934	ACOX1	1
GO:001663	MF	GO:001663	oxidoreduc	1/932	4/18369	0.188035	0.485925	0.468934	SDHD	1
GO:001664	MF	GO:001664	oxidoreduc	1/932	4/18369	0.188035	0.485925	0.468934	PAOX	1
GO:001693	MF	GO:001693	extracellula	1/932	4/18369	0.188035	0.485925	0.468934	GLRB	1
GO:001693	MF	GO:001693	extracellula	1/932	4/18369	0.188035	0.485925	0.468934	GLRB	1
GO:001706	MF	GO:001706	fatty acid a	1/932	4/18369	0.188035	0.485925	0.468934	ASAH1	1
GO:002302	MF	GO:002302	MHC class	1/932	4/18369	0.188035	0.485925	0.468934	LILRB1	1
GO:003419	MF	GO:003419	apolipopro	1/932	4/18369	0.188035	0.485925	0.468934	CDC42	1
GO:003473	MF	GO:003473	histone dea	1/932	4/18369	0.188035	0.485925	0.468934	SIRT6	1
GO:003524	MF	GO:003524	protein-arg	1/932	4/18369	0.188035	0.485925	0.468934	NDUFAF7	1
GO:003536	MF	GO:003536	selenocyste	1/932	4/18369	0.188035	0.485925	0.468934	SECISBP2L	1
GO:003541	MF	GO:003541	dihydrotes	1/932	4/18369	0.188035	0.485925	0.468934	AKR1C3	1
GO:003552	MF	GO:003552	NADH pyro	1/932	4/18369	0.188035	0.485925	0.468934	NUDT12	1
GO:003566	MF	GO:003566	Toll-like re	1/932	4/18369	0.188035	0.485925	0.468934	TIRAP	1
GO:003580	MF	GO:003580	deubiquitir	1/932	4/18369	0.188035	0.485925	0.468934	WDR48	1
GO:004229	MF	GO:004229	ISG15 trans	1/932	4/18369	0.188035	0.485925	0.468934	UBE2L6	1
GO:004295	MF	GO:004295	alkanesulfo	1/932	4/18369	0.188035	0.485925	0.468934	SLC6A6	1
GO:004697	MF	GO:004697	TAP1 bindi	1/932	4/18369	0.188035	0.485925	0.468934	TAP1	1
GO:004775	MF	GO:004775	chondroitir	1/932	4/18369	0.188035	0.485925	0.468934	CHST12	1
GO:004840	MF	GO:004840	brain-deriv	1/932	4/18369	0.188035	0.485925	0.468934	NTRK2	1
GO:004840	MF	GO:004840	epidermal	1/932	4/18369	0.188035	0.485925	0.468934	SEC61B	1
GO:005029	MF	GO:005029	steroid sulf	1/932	4/18369	0.188035	0.485925	0.468934	SULT1A1	1
GO:005043	MF	GO:005043	microfibril	1/932	4/18369	0.188035	0.485925	0.468934	LTBP2	1
GO:005102	MF	GO:005102	Rho GDP-c	1/932	4/18369	0.188035	0.485925	0.468934	HSP90AA1	1
GO:005273	MF	GO:005273	tRNA (cyto	1/932	4/18369	0.188035	0.485925	0.468934	METTL8	1
GO:006218	MF	GO:006218	anandamic	1/932	4/18369	0.188035	0.485925	0.468934	CYP2B6	1
GO:006218	MF	GO:006218	anandamic	1/932	4/18369	0.188035	0.485925	0.468934	CYP2B6	1

GO:00712(	MF	GO:00712(	histone pre	1/932	4/18369	0.188035	0.485925	0.468934	SLBP	1
GO:009701	MF	GO:009701	L27 domai	1/932	4/18369	0.188035	0.485925	0.468934	DLG1	1
GO:00975(	MF	GO:00975(	deaminate	1/932	4/18369	0.188035	0.485925	0.468934	SMUG1	1
GO:009891	MF	GO:009891	structural c	1/932	4/18369	0.188035	0.485925	0.468934	DLG1	1
GO:012032	MF	GO:012032	NuRD com	1/932	4/18369	0.188035	0.485925	0.468934	ZNF827	1
GO:01402(	MF	GO:01402(	mRNA cap	1/932	4/18369	0.188035	0.485925	0.468934	PHAX	1
GO:014057	MF	GO:014057	transmemb	1/932	4/18369	0.188035	0.485925	0.468934	CYB561D2	1
GO:014057	MF	GO:014057	transmemb	1/932	4/18369	0.188035	0.485925	0.468934	CYB561D2	1
GO:190484	MF	GO:190484	TORC2 cor	1/932	4/18369	0.188035	0.485925	0.468934	SIRT6	1
GO:00081(	MF	GO:00081(	UDP-glycc	10/932	143/18369	0.19056	0.491314	0.474134	MGAT2/GA	10
GO:000851	MF	GO:000851	organic an	15/932	230/18369	0.19242	0.491314	0.474134	MFSD2A/S	15
GO:001641	MF	GO:001641	S-acyltrans	3/932	30/18369	0.193357	0.491314	0.474134	ZDHHC20/	3
GO:00150(	MF	GO:00150(	magnesiun	2/932	16/18369	0.193527	0.491314	0.474134	MAGT1/CN	2
GO:001537	MF	GO:001537	anion:sodiu	2/932	16/18369	0.193527	0.491314	0.474134	SLC5A5/SL	2
GO:00170(	MF	GO:00170(	chloride ch	2/932	16/18369	0.193527	0.491314	0.474134	VTI1B/SGK	2
GO:00228(	MF	GO:00228(	alanine tra	2/932	16/18369	0.193527	0.491314	0.474134	SLC36A4/S	2
GO:00302(	MF	GO:00302(	transmemb	2/932	16/18369	0.193527	0.491314	0.474134	GREM1/DG	2
GO:004281	MF	GO:004281	Wnt-activa	2/932	16/18369	0.193527	0.491314	0.474134	FZD7/FZD6	2
GO:00704(	MF	GO:00704(	NADPH bir	2/932	16/18369	0.193527	0.491314	0.474134	LBR/CRYZ	2
GO:00080(	MF	GO:00080(	monocarb	5/932	61/18369	0.196492	0.497964	0.480552	MFSD2A/S	5
